# Contribution of cryptochromes and photolyases for insect life under sunlight

**DOI:** 10.1007/s00359-022-01607-5

**Published:** 2023-01-06

**Authors:** Peter Deppisch, Valentina Kirsch, Charlotte Helfrich-Förster, Pingkalai R. Senthilan

**Affiliations:** Neurobiology and Genetics, Theodor-Boveri Institute, Biocenter, Julius-Maximilians-University Würzburg, 97074 Würzburg, Germany

**Keywords:** Cryptochrome, Photolyase, CRY/PL family, *Drosophila melanogaster*, Circadian clock

## Abstract

The cryptochrome/photolyase (CRY/PL) family is essential for life under sunlight because photolyases repair UV-damaged DNA and cryptochromes are normally part of the circadian clock that controls the activity–sleep cycle within the 24-h day. In this study, we aim to understand how the lineage and habitat of an insect affects its CRY/PL composition. To this end, we searched the large number of annotated protein sequences of 340 insect species already available in databases for CRY/PLs. Using phylogenetic tree and motif analyses, we identified four frequent CRY/PLs in insects: the photolyases 6-4 PL and CPDII PL, as well as the mammalian-type cryptochrome (MCRY) and Drosophila-type cryptochrome (DCRY). Assignment of CRY/PLs to the corresponding insects confirmed that light-exposed insects tend to have more CRY/PLs than insects with little light exposure. Nevertheless, even insects with greatly reduced CRY/PLs still possess MCRY, which can be regarded as the major insect cryptochrome. Only flies of the genus Schizophora, which includes *Drosophila melanogaster*, lost MCRY. Moreover, we found that MCRY and CPDII PL as well as DCRY and 6-4 PL occur very frequently together, suggesting an interaction between the two pairs.

**Supplementary Information:**

The online version contains supplementary material available at 10.1007/s00359-022-01607-5.

## Introduction

Sunlight is challenging for all organisms as it contains high-energy UV light that can cause DNA damage. On the other hand, sunlight enables visual orientation and color vision, which helps animals to find nutritious food, avoid predators, and interact socially. Light is also the most important zeitgeber for synchronizing their circadian clock. Therefore, all organisms have evolved mechanisms to protect themselves from UV light, but at the same time have multiple photoreceptors to perceive the different wavelengths of light and optimally synchronize their circadian clock and time their activity.

### The cryptochrome/photolyase family: a key driver for life under sunlight

The cryptochrome/photolyase family, also known as CRY/PL family or as CPF, appears crucial for a life under sunlight. Photolyases repair UV-damaged DNA and cryptochromes are involved in circadian clocks, either as core clock members that determine the timing of the sleep–wake cycle or as photoreceptors that synchronize the core clock to external light–dark cycles. Both roles of cryptochromes can help avoid or search for light, depending on the permanent or momentary needs of a species. Consistent with the various roles played by CPF members, the CRY/PL repertoire in different organisms appears to be highly variable and, in addition to phylogenetic causes, environmental factors appear to play a major role in this (Haug et al. 2015; Deppisch et al. 2022).

### Photolyases: blue light-dependent restoration of DNA

Photolyases are the ancestors of cryptochromes and are divided into 6-4 and CPD photolyases, depending on whether they repair pyrimidine-pyrimidone (6-4) photoproducts or cyclobutane-pyrimidine dimers (CPD). The CPD photolyases are further subdivided into three subgroups, called CPDI, CPDII, or CPDIII photolyases. 6-4 photolyases (6-4 PL) and the CPDII photolyases (CPDII PL) have so far been identified in insects. While 6-4 PLs are most likely the ancestors of the animal CRYs, CPDII PLs have very little similarity to them and are also quite different to the other CPD PLs (Mei and Dvornyk 2015; Michael et al. 2017; Deppisch et al. 2022).

### Cryptochromes and their involvement in the circadian clock

The best-known CRY/PL representatives in insects are the two cryptochromes DCRY (*Drosophila*-type CRY, also called CRY1, dCRY, d-CRY, or CRY-d) and MCRY (Mammalian type CRY, also called CRY2, mCRY, m-CRY, or CRY-m). Both are active in the circadian clock but modulate it in different ways. The function of DCRY depends on the *Drosophila* type TIMELESS (DTIM, also called dTIM or TIM-d). All animals that have a DCRY also have the DTIM (Kotwica-Rolinska et al. 2021). DTIM is part of the core circadian clock and forms a heterodimer with the clock protein PERIOD (PER). The PER-DTIM heterodimer binds and inhibits the CLOCK-CYCLE heterodimer, which acts as transcriptional activators of the *period* and *timeless* genes (Darlington et al. 1998; Lee et al. 1999). By this means, DTIM and PER block their own expression in a negative feedback loop, which leads to an oscillation. Further proteins, especially kinases, extend the period of this oscillation to circa 24 h (Bae and Edery 2006; Rosbash et al. 2007). The light-sensitive DCRY plays an exclusive role in this process by adjusting it to the 24-h day–night cycle. Light-activated DCRY leads to the degradation of DTIM, which then can no longer inhibit its own transcription and thus *dtim* can be transcribed again; in other words, the molecular cycle is reset every morning by the action of DCRY (Ceriani et al. 1999; Peschel et al. 2009; Ozturk et al. 2013). In contrast, the light-insensitive MCRY does not act on DTIM but assumes the role of DTIM itself as an interaction partner of PER (Thresher et al. 1998; Kume et al. 1999; Vitaterna et al. 1999; Griffin et al. 1999). In addition to MCRY and DCRY-based clocks, many insect circadian clocks rely on both (Zhu et al. 2005, 2008; Yuan et al. 2007). MCRY is more original and widespread (Deppisch et al. 2022), thus MCRY based clocks are probably also the more original clocks.

### Insects: a versatile metazoan class to study CRY/PLs

Insects are especially interesting for exploring the various roles of CRY/PLs. Insects contribute significantly to animal biodiversity. About one million insect species are known worldwide. However, the number of species that have not yet been discovered and described is probably five times as high (Stork 2018). As great as their biodiversity is, so are their living conditions. Not only are their ways of life (whether solitary, social or parasitic) very different, but also their habitats. For example, while termites live in hidden, dark places, aphids are exposed to sunlight most of the day. The way of life may even change during insect development. Tobacco hornworms *Manduca sexta* for example, are exposed to high levels of sunlight in their larval and pupal stages but become nocturnal as adults. Thus, it is expected that different CRY/PL family members are present in various insects and that a thorough analysis of them will help to better understand their function and evolution. So far, besides MCRY, DCRY, the two photolyases 6-4 PL and CPDII PL, also DASH-CRY, whose function is not yet fully understood (Kiontke et al. 2020), have been reported in insects (Kotwica-Rolinska et al. 2021; Deppisch et al. 2022). Both studies indicate a large variety in CRY/PL distribution in insects. In this work, we aim to understand the contribution of ancestry and the way of life of an insect on its CRY/PL composition.

## Methods

### Finding putative insect CRY/PLs

We used the NCBI’s BLASTP program (https://blast.ncbi.nlm.nih.gov/Blast.cgi?PAGE=Proteins, accessed in October 2022) to find putative CRY/PL homologs in insects. For this purpose, the amino acid sequences of *Drosophila melanogaster* CRYPTOCHROME (NP_732407) and *Drosophila melanogaster* PHOTOREPAIR (NP_523653) were used as reference sequences. For BLASTP search, we selected the non-redundant protein sequences (nr) as the search set and BLASTP (protein–protein BLAST) as the algorithm with its default parameters (BLOSUM62, Gap Costs 11/1, Conditional compositional score matrix adjustment). To obtain only insect-specific sequences, the search was restricted to the NCBI TaxID insects (6960) and the maximum number of results per search (Max target sequences) was set to 5000 sequences. In order not to miss any sequence, the search was subsequently narrowed down to single orders and performed again. Then, duplicated protein sequences were deleted using their accession numbers. The remaining sequences had different origins; some sequences came from complete or partial genomes, some from transcriptomes, and some from studies that examined, sequenced, and annotated CRY/PLs in specific organisms. We collected the analysis results in MS-Excel, customized the sequence labels with species name and accession number and transferred the amino acid sequences with their adjusted sequence labels into Geneious Prime software 2022.2.2. In this process, we took only the sequences that had an *e*-value of less than *e*-15. This cutoff value was adopted from Deppisch et al. (2022) as we aimed to compare both studies. Furthermore, this cutoff value proved to be well suited to avoid overlooking CRY/PLs while keeping false results to a minimum. We filtered out the few spurious values by further analyses, especially back-BLAST and motif analyses. Despite the cutoff of *e*-15, most CRY/PL homologs in our study had an e value between *e*-50 and 0. At the end of this process, we obtained 1514 protein sequences from 342 insects, but they also contained erroneous and multiple annotations, which we cleaned up in the next step. The final 912 purified sequences (sum of sequences identified in this study and Deppisch et al. (2022)) and their accession numbers are listed in Supplementary file 1.

### Taxonomy

In this step, we determined the taxonomy of all insects in which we found CRY/PLs. For this purpose, we chose the NCBI taxonomy tool (www.ncbi.nlm.nih.gov/taxonomy, accessed in October 2022) and assigned the found species according to their scientific classifications. We have listed all organisms studied and their taxonomic assignment on Supplementary file 2. The exact number of organisms examined per taxonomic level is further indicated in the respective results. To keep the degrees of relationship between the insects fairly accurate, we also consulted Lifemap (https://lifemap-ncbi.univ-lyon1.fr/) and One Zoom (https://www.onezoom.org/), both accessed in October 2022.

### Motif analyses

For all sequences found, we performed a motif analysis using the Annotate & Predict tool of the Geneious Prime software 2022.2.2. We screened the retrieved sequences for all motifs described in Deppisch et al. (2022) with the same parameters.

### Phylogenetic tree

Based on the previously described protein sequences from Deppisch et al. (2022), we constructed an unrooted phylogenetic tree to assign the identified insect CRY/PL protein sequences to their appropriate subfamilies. The phylogenetic tree was created using the Geneious Prime software 2022.2.2. We applied the Alignment type: Global alignment with the Cost Matrix PAM 100 and the gap costs 10/1 and selected Jukes-Cantor as Genetic Distance Model and Neighbor-Joining as Tree Build Method. The initial tree containing 3763 protein sequences (1514 new and 2249 from Deppisch et al. 2022) is given in Supplementary file 3. We then manually verified the sequences in the tree, and incorrect/multiple annotations as well isoforms were sequentially removed (727 in total were removed). The main factor considered in the decision to delete or keep a sequence was the CRY/PL motifs present. Isoforms with the most motifs were prioritized. The final tree was then created using the same parameters with the remaining 3036 sequences (787 sequences identified in this study and 2249 from Deppisch et al. 2022). The phylogenetic tree was further processed with FigTree v1.4.4. The detailed final phylogenetic tree is given in unrooted and rooted version in the Supplementary files 4 and 5.

### Classification of the CRY/PL subfamilies

Based on the unrooted phylogenetic tree (Supplementary file 4), we identified the subfamilies of the CRY/PL family. For this purpose, we used the corresponding reference protein sequences of the CRY/PL subfamily described in Deppisch et al. (2022). To ensure this, we constantly checked the CRY/PL motifs of individual protein sequences.

### Assignment of CRY/PL subfamily members to their organisms

We assigned the protein sequences to the respective CRY/PL subfamilies based on tree branches and protein motifs. Next, we examined which CRY/PL subfamilies were possessed by the insects studied. This distribution was then inserted into the phylogenetic taxon of each organism, which is listed in Supplementary file 2. Here, we noticed that some CRY/PL sequences were derived solely from clock-associated studies and, therefore, might reveal little about the other CRY/PLs, especially photolyases. Thus, we examined the genome quality and its completeness for the insects studied. For this purpose, we selected only species with a full annotation report at NCBI (https://www.ncbi.nlm.nih.gov/genome/annotation_euk/all/) or species where conceptual translation was applied on a genome assembly to predict the proteome. We also checked the available annotations and the quality of the assembly using BUSCO scores (Simão et al. 2015), N50 values (Earl et al. 2011) and the sequencing coverage (Supplementary file 9). Moreover, we performed protein sequence alignments with their respective reference proteins for organisms with doubtful CRY/PL distributions. The alignments were performed with Geneious Prime software 2022.2.2 (Global alignment with free end gaps, the Cost Matrix PAM 100 and the gap costs 10/1 and selected Jukes-Cantor as Genetic Distance Model).

Insects not fulfilling our criteria were excluded from our final analysis in the results section. Finally, the overall distribution and frequency of presence of each CRY/PL family member within a taxon were calculated. The data of all, including the incompletely sequenced insects, can be found in Supplementary files 6 and 10. Insects that were not included due to incompleteness are shown in gray in the Supplementary file 6.

### Figures

Figures 3, 5, and 6 were created entirely or in part using BioRender.com.

## Results

### CRY/PL subfamilies in insect

**Fig. 1:**
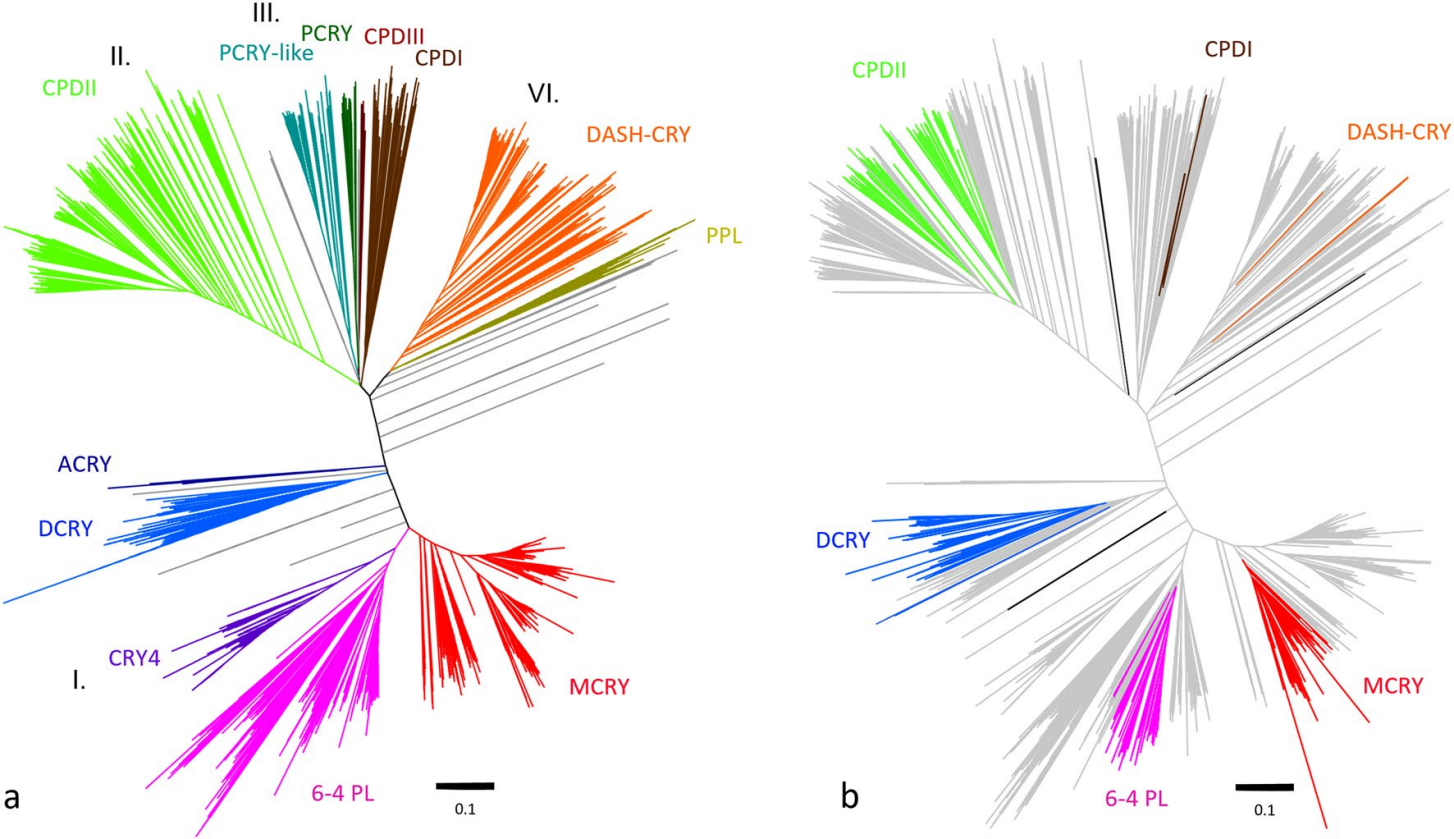
Unrooted phylogenetic trees representing all major CRY/PL subfamilies. **a** Unrooted tree representing the known CRY/PL subfamilies (adapted from Deppisch et al. 2022). ACRYs (dark blue), DCRYs (blue), CRY4s (purple), 6-4 PLs (pink), and MCRYs (red) belong to the 6-4 PL cluster (I). The CPDII PLs (light green) are the only CRY/PLs in the CPDII PL cluster (II). The CPDI (dark brown) as well as CPDIII photolyases (light brown), the plant cryptochrome PCRY (dark green) and the PCRY-like (turquoise) belong to the CPDI/III cluster (III). DASH-CRYs (orange) and the plant photolyase PPLs (ochre green) belong to the DASH-CRY cluster (IV). **b** Unrooted tree to which all so far sequenced and annotated insect CRY/PL sequences were added and shown in color. Insect-specific sequences were present in the CPDII PL, 6-4 PL, MCRY, and DCRY clusters. A small number of insect sequences clustered with DASH-CRY and CPDI PLs, while few sequences (dark grey) were not assignable to the known CRY/PLs

To highlight the variety of CRY/PL subfamilies and classify insect CRY/PLs in their general phylogeny, we used the unrooted phylogenetic tree generated in Deppisch et al. (2022) and added 787 insects CRY/PL sequences from the present study. The former tree contained the sequences from maximally five species per order and two species per family, among which were just 125 insect sequences, making the tree phylogenetically quite balanced (Fig. 1a; Deppisch et al. 2022). The new tree contains 912 insect sequences (from 340 insect species), which were highlighted in color, and 2124 non-insect sequences (Fig. 1b). Due to the high number of insect sequences, this tree is not balanced and only of limited use for phylogenetic statements classification. However, when comparing it with the original tree in Deppisch et al. (2022) (Fig. 1a), it clearly demonstrates that insects possess mainly members of the subfamilies MCRY (red), 6-4 photolyase (pink), DCRY (blue), and CPDII photolyase (light green), while the other subfamilies are underrepresented (Fig. 1a). None of the insect sequences clusters with the plant photolyase (PPL, ochre green), plant CRY (dark green), the marine organism specific PCRY-like (turquoise), the CPDIII photolyase (brown), the chordate-specific CRY4 cluster (purple) and the cnidarian-specific ACRY cluster (dark blue). Also, CPDI (dark brown) and DASH-CRY (orange) hardly occur in insects. Only three insect sequences cluster with DASH-CRY and two with CPDI photolyases. Five sequences do not cluster with any known CRYPL subfamily and remain unexplored (dark grey in Fig. 1b). These belong to the cat flea *Ctenocephalides felis,* the bug *Apolygus lucorum,* and several walking stick species (*Timema*).

Most insect CRY/PLs are members of the CPDII photolyase cluster, followed by the MCRY cluster. DCRY and 6-4 photolyases are clustered about equally. Interestingly, some insects (*Ischnura elegans*, *Ladona fulva*, and all flies of the superfamily Tephridoidea) appear to possess two CPDII photolyases. While repeated annotations and protein isoforms usually cluster together in the phylogenetic tree, these duplicates were located nearby but in different branches (Supplementary files 4 and 5).

### Motif analysis

**Fig. 2:**
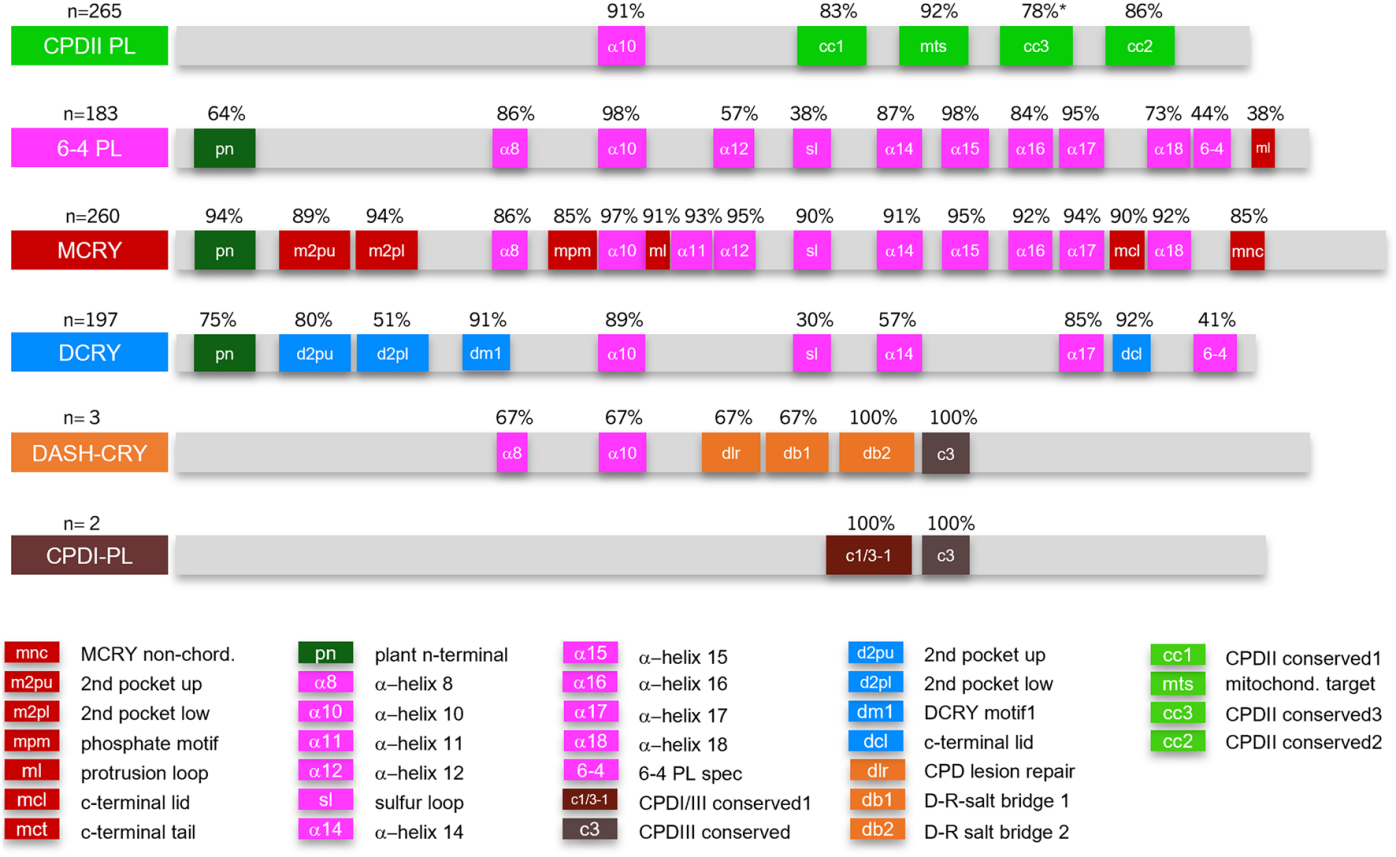
Schematical presentation of the insect CRY/PL subfamilies with their most frequent motifs. Only motifs that occur with a frequency of at least 30% are shown. We used the same motifs and search parameters as described in Deppisch et al. (2022). All protein sequences including their motifs are listed in Supplementary file 7. The number of sequences studied is indicated above the subfamily name. In the case of DCRY of walking sticks (*Timema spec.*), we included sequences that clustered differently but had DCRY motifs. In the case of the cc3 motif of CPDII PL (*), both plant (9%) and microorganism/animal (69%) motifs were combined

To verify the accuracy of the phylogenetic tree, we performed a motif analysis using the Annotate & Predict tool in Geneious Prime software, applying all previously known CRY/PL motifs summarized by Deppisch et al. (2022). We found that almost all identified CRY/PLs exhibit their typical subfamilial motifs (Fig. 2). All protein sequences with their protein motifs are listed in Supplementary file 7. Based on the motif analysis, we could assign the three unclassifiable sequences from walking sticks (*Timema* spec.) to DCRY. Apparently, they were annotated as smaller fragments, but all possess DCRY specific motifs (Supplementary file 7). Protein sequence alignment of all putative *Timema* DCRY sequences compared to DCRY from *Drosophila melanogaster* (Supplementary file 8) showed that the different fragments belong to the same DCRY sequence. Therefore, we added these *Timema* sequences to DCRY in Fig. 2. The unclassifiable sequence from cat flea *Ctenocephalides felis* (XP_026474410.1) carries 6-4 PL cluster-typical motifs that are common in 6-4 PLs as well as in MCRYs and DCRYs. As we cannot assign it more precisely, we did not include it in Fig. 2 and disregarded it for further analyses. We did the same for the unclassified sequence of the bug, *Apolygus lucorum* (KAF6198968.1), because we did not find any CRY/PL motifs, suggesting that this sequence is not a CRY/PL or at least not a hitherto known CRY/PL.

Due to the large number of sequences examined, we can make precise statements about the occurrence and frequency of individual motifs in CPDII PL, 6-4 PL, MCRY, and DCRY, while we cannot do so for the rarely occurring CRY/PLs (DASH-CRY and CPDI-PL) (Fig. 2). In the following, we will describe the distribution of the individual motifs in more detail.

CPDII photolyases are characterized by the alpha helix motif α10, the CPDII-PL-specific motifs cc1 (CPDII conserved1), cc2 (CPDII conserved2), cc3 (CPDII conserved3), and mts (mitochondrial target sequence) (Fig. 2). The α10 motif is very abundant in all CRY/PLs except for the CPDI photolyases, which have completely different motifs. The 6-4 photolyases are characterized by several additional alpha helixes (α8, α10, α12, α14, α15, α16, α17, and α18), the sulfur loop (sl), and the plant n-terminal (pn) motifs. These motifs are very common not only in the 6-4 photolyase itself, but also in MCRY and DCRY, which are derived from it. In addition, the 6-4 photolyases carry the 6-4 motif (6-4) and the MCRY specific protrusion loop (ml) (Fig. 2). The 6-4 motif is only found with a frequency of 44% in insect 6-4 photolyase, whereas it is almost twice as frequent (81%) in other organisms (Deppisch et al. 2022). In contrast, the MCRY specific protrusion loop (ml) occurs in 38% of insect 6-4 photolyases while it was only present in 17% of all 6-4 photolyases studied in Deppisch et al. (2022) and was, therefore, not considered. The ml motif is in different places in 6-4 photolyases and MCRYs (Fig. 2): in 6-4 photolyases, it is usually located at the C-terminal end, while it is between the alpha helices α10 and α11 in MCRY. Exceptions are the 6-4 PLs of the cotton bag worm *Eumeta japonica* where it also occurs between α10 and α11, that of the marine midge *Clunio marinus* where the motif occurs twice in succession between α10 and α11, and that of *Drosophila innubila*, where it occurs in the N-terminus between plant N-terminus (pn) and α10 (Supplementary file 7). *Drosophila innubila* is the only Muscomorpha in which the ml motif occurs. The ml motif is generally rare in flies (Brachycera). We found another ml motif in the 6-4 PL of black soldier flies (*Hermetia illucens*), and there it occurs at the C-terminal end, as in all other insects (Supplementary files 6 and 7). The 38% frequency of the ml motif in Fig. 2 was calculated regardless of its position.

MCRYs carry an additional alpha helix, α11, besides the already mentioned alpha helices of 6-4 photolyases (Fig. 2). With a percentage of 93%, α11 is very frequent in MCRYs while it occurs in only 13% of the 6-4 PLs and is, therefore, not included in Fig. 2. Furthermore, MCRYs possess the MCRY-specific motifs m2pu (MCRY 2nd pocket up), m2pl (MCRY 2nd pocket low), mpm (MCRY phosphate motif), mnc (MCRY-non-chordates), and the already mentioned protrusion loop, ml. Similarly, DCRYs are characterized by the additional DCRY motifs d2pu (DCRY 2nd pocket up), d2pl (DCRY 2nd pocket low), dm1 (DCRY motif1), and dcl (DCRY c-terminal lid).

In DASH-CRY sequences, we find in addition to the alpha helices 8 and 10, the DASH-CRY-specific dlr (CPD lesion repair), db1 (D-R-salt bridge 1), and db2 (D-R-salt bridge 2), as well as the c3 motif (CPDIII conserved). In the two CPDI photolyases, the c1/3-1 (CPDI/III conserved1) and c3 motifs are present. Both motif analyses reveal that the DASH-CRYs and CPDI photolyases found in insects do indeed belong to these subfamilies and are not resulting from incorrect clustering in the phylogenetic tree.

### CRY/PL distribution

**Fig. 3:**
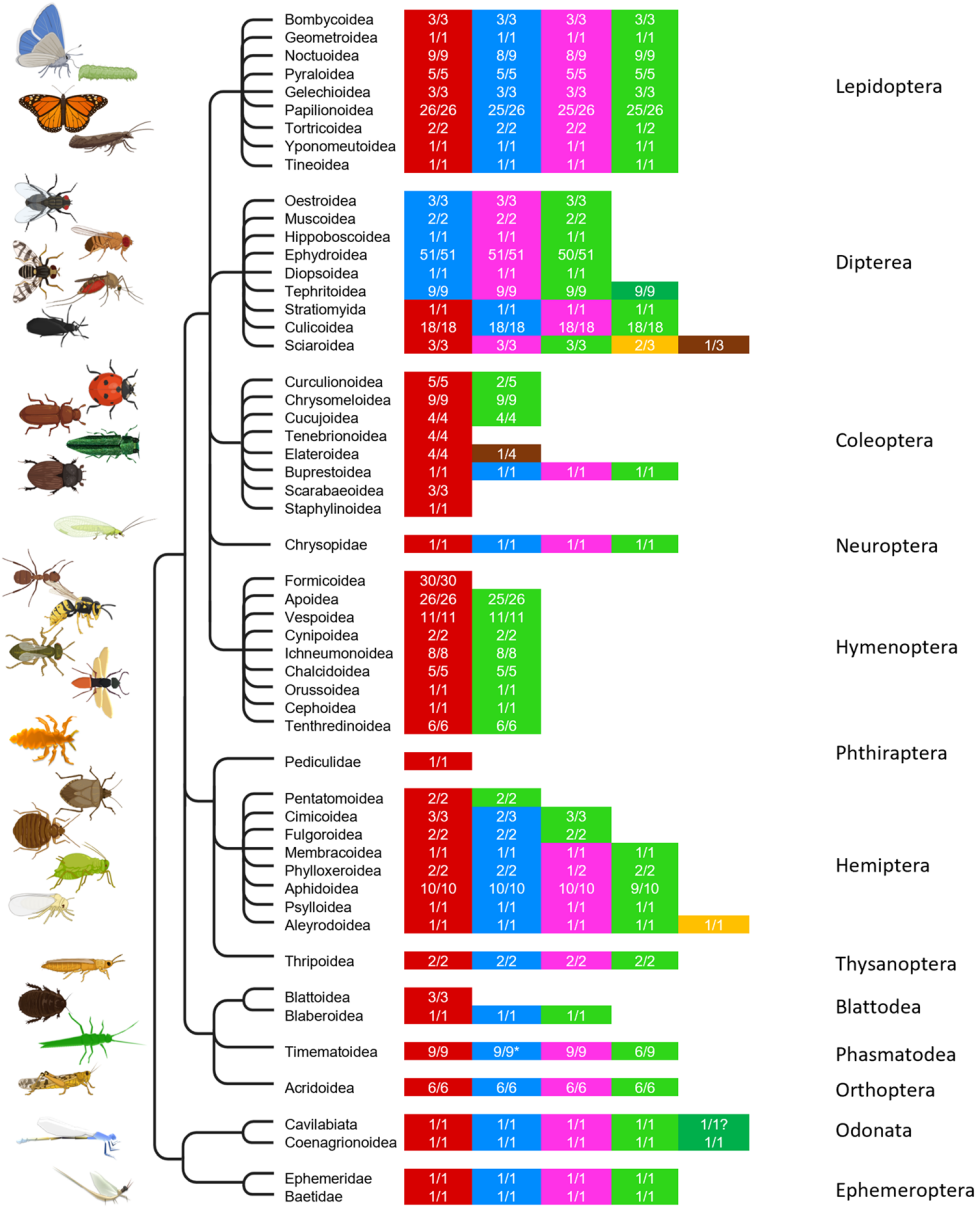
Distribution of CRY/PL subfamilies within insect orders and superfamilies (MCRY dark red, DCRY blue, 6-4 PL light magenta, CPDII light green, CPDII duplication green, CPDI brown, DASH-CRY orange). The prevalence of a CRY/PL within an insect superfamily is shown as a fraction, with the denominator representing the total number of animals examined in a superfamily and the numerator representing the number of animals with the respective CRY/PL. MCRY, DCRY, 6-4 PL, and CPDII PL are the most common CRY/PLs in insects. All studied insects belonging to the orders Ephemeroptera, Odonata, Phasmatodea, Thysanoptera, and Neuroptera possess them. Most Lepidoptera and many insects belonging to Hemiptera (depending on the superfamily) also have all of them. In Hymenoptera, the CRY/PLs are greatly reduced, and some groups retain only MCRY. Such reductions are also observed in insects belonging to other orders. Many superfamilies of Diptera lack MCRY and possess only DCRY, 6-4 PL, and CPDII PL. Some individual insects also have DASH-CRY or CPDI photolyase and others possess a CPDII photolyase duplication. DCRY is only fragmentarily sequenced and annotated in most Timematoidea (*). But in all studied *Timema* species we could find DCRY fragments

To obtain an overview of the distribution of CRY/PL family members in the different insect groups, we subdivided the individual insects into orders and superfamilies (if subdivision into superfamilies was not given, into families) and examined the CRY/PL distribution in these (Fig. 3). As already evident in the unrooted tree (Fig. 1), MCRY, DCRY and the two photolyases 6-4 and CPDII are the CRY/PLs most abundant in insects. In the following, we will refer to them as the four main insect CRY/PLs or even as the four CRY/PLs. Nevertheless, the four CRY/PLs are not present in all insects. Furthermore, few insects possess in addition or instead the DASH-CRY and CPDI photolyases, and few other insects carry a CPDII duplication (Fig. 3).

### All four CRY/PLs in Palaeoptera

**Fig. 4:**
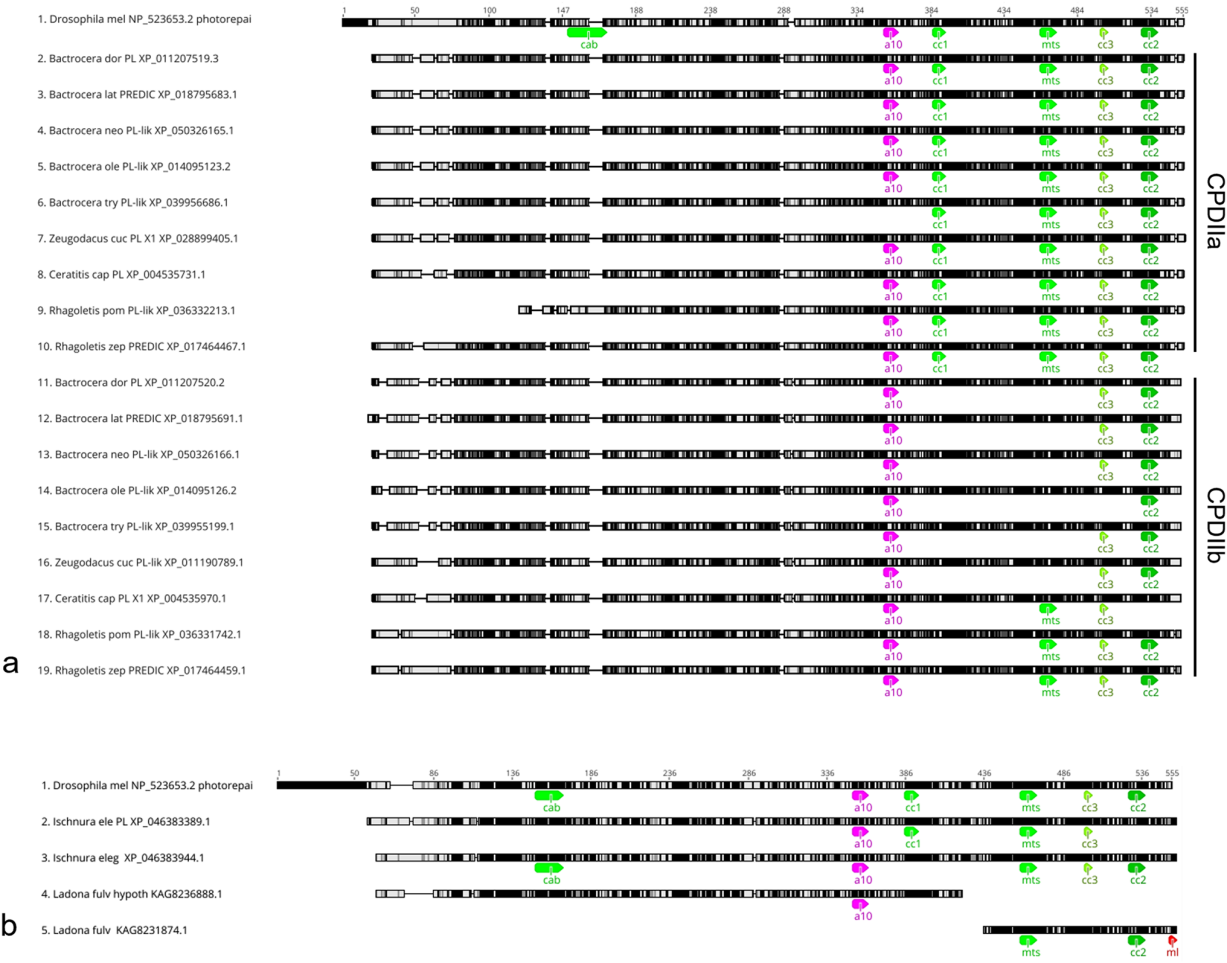
Protein sequence alignment of CPDII photolyase duplicates from Tephritoidea with Drosophila melanogaster sequences indicates a CPDII PL duplication in Tephritoidea and dragon flies resulting in CPDIIa and CPDIIb sequences. **a** All Tephritoidea sequences lack the CPDII antenna binding (cab) motif that is present in Drosophila. The duplicates b additionally lack the cc1 (1) motif and some b duplicates lack also the mitochondrial targeting motif (m) of CPDII. **b** Protein sequence alignment of the two CPDII photolyases from *Ischnura elegans* and *Ladona fulva* compared with the CPDII PL from *Drosophila melanogaster*. While XP_046383944.1 has a cab motif but no cc1 motif, XP_046383389.1 carries a cc1 motif and no cab motif. The two *Ladona* sequences are incomplete and therefore can be derived from the same sequence as well as from two different CPDII-PLs

**Fig. 5:**
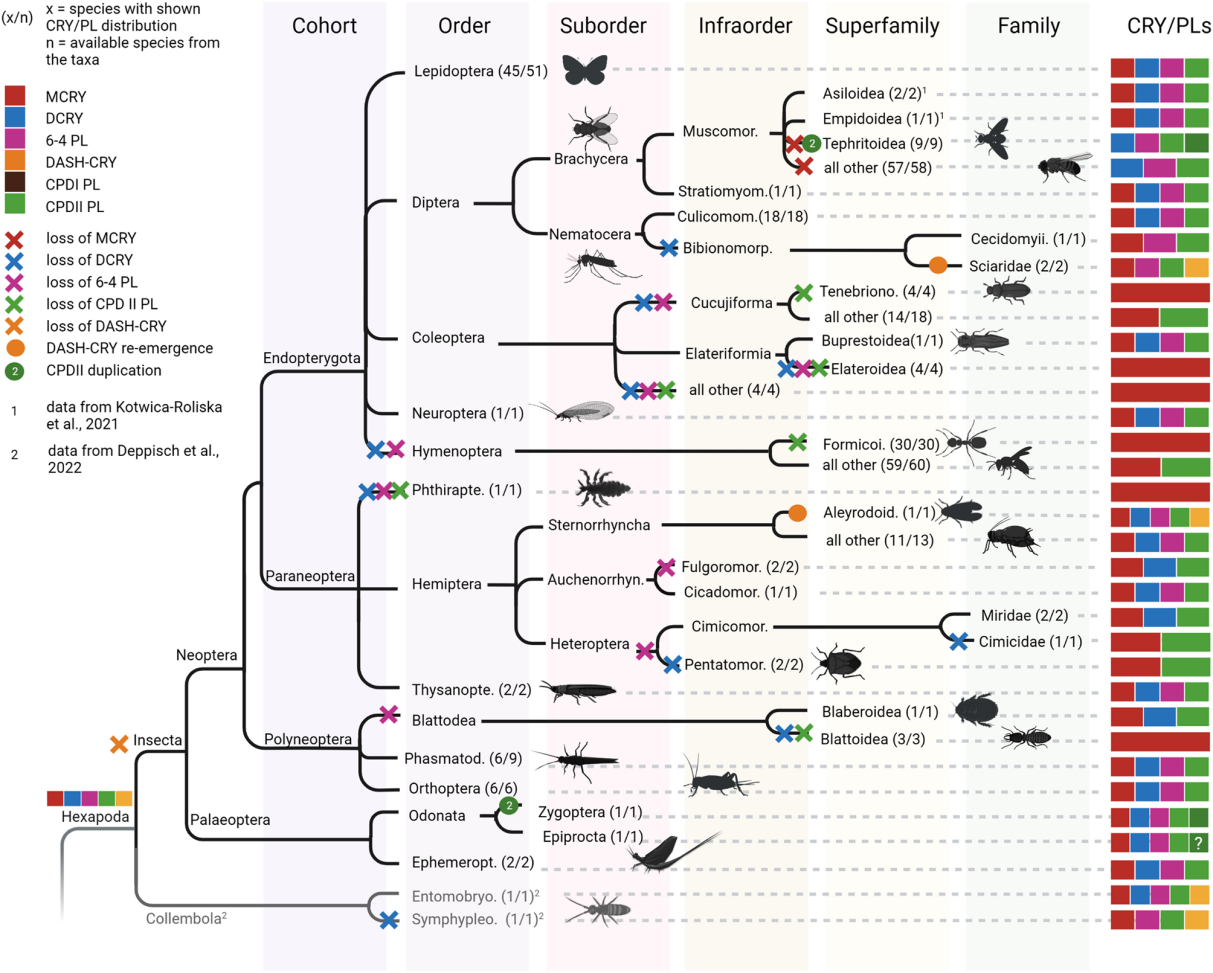
Distribution of CRY/PL subfamilies in hexapoda down to the resolution of certain insect species in order to demonstrate the loss and rare reappearance of CRY/PL family members. The total number of animals examined within a taxon (denominator) and the number of animals with CRY/PL distribution shown (numerator) are given in parentheses next to the taxonomic name. Data labeled 1 (in superscript) are from the publication by Kotwica-Rolinska et al. (2021) and data labeled 2 (in superscript) are from Deppisch et al. (2022). The ancestors of insects probably had five members of the CRY/PL family-MCRY, DCRY, 6-4 PL, CPDII PL, and DASH-CRY. The more primitive hexapods, which belong to the class Collembola, possess these five CRY/PLs. However, DASH-CRY is lost in most insects. The insects of the superfamily Sciaroidea and Aleyrodoidea again possess DASH-CRYs. In Zygotera (suborder) and Tephritoidea (superfamily) we see a CPDII duplication. In Epiprocta (suborder), we cannot yet confirm this duplication. Other CRY/PL members of the insects have been lost at different phylogenetic levels. In the Hymenoptera, DCRY and 6-4 PL have already been lost at the order level. CPDII has been lost only at the level of the superfamily Formicoidea. Similarly, MCRY has been lost in the Muscomorpha only in some superfamilies

The mayflies (order: Ephemeroptera) and dragonflies (order: Odonata) belong to the more primitive infraclass Palaeoptera (Fig. 5). A characteristic of this infraclass is the inability of the insects to fold their wings over the abdomen as the insects of the Neoptera do. In our analysis, we found only two mayflies and two dragonflies with a sequenced and annotated genome. All of them contain the four insect CRY/PLs: MCRY, DCRY, 6-4 and CPDII photolyases (Fig. 3). The two dragonflies, *Ischnura elegans* and *Ladona fulva,* appear furthermore to possess a CPDII duplication (Fig. 4). While the blue-tailed damselfly *Ischnura elegans*, clearly has two CDPII photolyases (CPDIIa and CPDIIb), this is less certain in the scarce chaser *Ladona fulva* due to incomplete sequences (Fig. 4, Supplementary file 8).

All other insects studied belong to the Infraclass Neoptera that can fold their wings over the abdomen and that can be further divided into Polyneoptera (orders: Orthoptera, Phasmatodea, and Blattodea), Paraneoptera (orders: Hemiptera, Thysanoptera, and Phthiraptera), and Endopterygota (orders: Hymenoptera, Neuroptera, Coleoptera, Diptera, and Lepidoptera) (Fig. 5). In the following, we will describe the CRY/PL member composition in the different orders briefly.

#### Orthoptera

 We examined nine species belonging to the order Orthoptera, three of which belong to the suborder Grilloidea (crickets) and are not considered in the final calculation as their genomes are not fully sequenced (Supplementary file 9). All other six Orthoptera belong to the suborder Acrididae (locusts) and possess all four CRY/PLs (Fig. 3).

#### Phasmatodea

The nine species sequenced belong all to the already discussed walking sticks (Timematoidea). The majority of these species appear to possess all 4 CRY/PL members. Only the CPDII photolyases were found in 67% of the species (Fig. 3). Since the *Timema* sequences are more likely to be fragments and, therefore, have smaller *e*-values than complete sequences, their CPDII PLs may have been missed in the BLASTP analysis with our applied parameters.

#### Blattodea

Though we found a total of eight Blattodea in our analysis, only four of them are fully sequenced and were considered for further analyses. Of these only the German cockroach *Blattella germanica* possesses DCRY and CPDII photolyase in addition to MCRY (Fig. 3). The other three sequenced termites seem to retain only MCRY (Fig. 3).

#### Thysanoptera

The two thrips examined again possess all four CRY/PLs.

#### Hemiptera

The eight investigated superfamilies of the order Hemiptera show a heterogeneous CRY/PL composition (Fig. 3). While nearly all species of the suborder Sternorrhyncha (consisting of the superfamilies Aleyrodoidea, Psylloiidae, Aphidoidea, Phylloxeroidea, and Membracoidea) have all four CRY/PLs, species from the other suborders (Auchenorrhyncha and Heteroptera) have significantly fewer (Fig. 3). The whitefly *Bemisia tabaci* from the superfamily Aleyrodoidea has an additional DASH-CRY. The Cimicoidea and Fulgoroidea lack the 6-4 photolyase, and within the Cimicoidea, the bed bug, *Cimex lectularis* lacks also DCRY. The same reduction (no 6-4 photolyase and no DCRY) is found in the Pentatomoidea. Although the fire bug from the superfamily *Pyrrhocoris apterus* was not included in our analysis due to incomplete sequencing (Supplementary file 6, 10), it also appears to possess MCRY and CPDII PL only (Kotwica-Rolinska et al. 2021).

#### Phiraptera

The body louse *Pediculus humanus corporis* is the only Phthiraptera studied and possesses only MCRY (Figs. 3, 5).

#### Hymenoptera

Of all the orders studied, the order Hymenoptera is probably the most conserved. All Hymenoptera examined lack DCRY and the 6-4 photolyase (Fig. 3). Almost all of them (59 out of 60), except those belonging to the superfamily Formicoidea (ants), carry the MCRY and the CPDII photolyase (Figs. 3, 5, Supplementary file 6). All 30 ants examined lost the CPDII photolyase and retain only MCRY (Fig. 5).

#### Neuroptera

The common green lacewing *Chrysoperla carnea*, the only Neuroptera studied, again possesses all four CRY/PLs.

#### Coleoptera

Most insects of the order Coleoptera (beetles) have rather a reduced CRY/PL composition (Fig. 3). The only exception is the emerald ash borer *Agrilus planipennis* (superfamily: Buprestoidea), which carries all four CRY/PLs. All other beetles studied have MCRY as their major CRY/PL family member and in most beetles, this is also the only one. Many beetles of the superfamily Chrysomeloidea (8/9) and all of the superfamily Cucujoidea (4/4) additionally possess the CPDII photolyase (Supplementary file 6). The firefly *Abscondita terminalis* (formerly also called *Luciola terminalis; s*uperfamily Elateroidea) is exceptional because it possesses a CPDI photolyase and no CPDII photolyase besides MCRY. (Figs. 3, 5). CPDI photolyases are rarely found in insects but are abundant in bacteria. Since fireflies are known to have an extensive microbiome because microbial endosymbionts such as *Tenericutes spec.* contribute to the metabolism and biosynthesis of luciferin (Fallon et al. 2018).Thus, it is quite likely that the found CPDI is a contamination from them.

#### Diptera

Diptera are classically divided into Nematocera and Brachycera (Fig. 5). Of the Nematocera, 18 species belonging to the infraorder Culicomorpha and three species belonging to the infraorder Bibionomorpha (superfamily Sciaroidea) have been sequenced (Fig. 3). The latter three are all gnats (fungus or turnip gnats) having a special position among all diptereans. The gnats lack DCRY, while most Culicomorpha possess the main insect CRY/PLs. Furthermore, the two fungus gnats *Bradysia coprophila* & *Bradysia odoriphaga* possess DASH-CRY. *B. odoriphaga* additionally seems to possess a CPDI photolyase, However, since this clusters with the CPDI photolyases of bacteria, including those of *Escherichia coli* and since the sister species *Bradysia coprophila* does not have any CPDI photolyase, we assume that it is more likely a bacterial contamination of the sequenced DNA.

Of the Brachycera, 67 species of the infraorder Muscomorpha and 1 species of the infraorder Stratiomyomorpha (the black soldier fly *Hermetia illucens*) have been sequenced (Fig. 5). While the black soldier fly possesses all four CRY/PL members, all sequenced species from the Muscomorpha have lost MCRY (Figs. 3, 5). The following superfamilies belong to the Muscomorpha: Oestoidea, Muscuoidea, Hippoboscoidea, Ephydroidea, Diopsoidea and Tephritoidea (Fig. 3). They all have DCRY as their main cryptochrome and the great majority have in addition the photolyases 6-4 and CPDII (Fig. 3). The CPDII photolyase was not detected in the fly *Drosophila immigrans* (superfamily Ephydroidea), but since it is present in its sister species *D. albomicans,* this speaks for an incomplete annotation. Thus, we can assume that 6-4 and CPDII photolyases are likely present in all Muscomorpha. The only flies that appear exceptional are the Tephritoidea (9 sequenced species), which have two CPDII photolyases, the CPDIIa and CPDIIb duplicates (Figs. 3, 5).

#### Lepidoptera

Almost all lepidopterans examined possess the four CRY/PLs. However, in some species one or the other member is missing. In some cases, this is probably due to sequencing and annotation errors.

### Protein and nucleotide sequence alignments of CPDII to verify gene duplications

To verify whether the CPDII PL duplicates found in the fruit flies of the superfamily Tephritoidea and the two dragon flies (Odonata) were gene duplications or alternatively spliced mRNAs, we aligned their protein sequences with the CPDII photolyase of *Drosophila melanogaster* (Fig. 4).

In the nine sequenced Tephritiodea, both duplicates differ mainly in their CPDII conserved1 motif (cc1, Fig. 5a), which consists of the amino acid sequence EEAVVRREL. A cc1 motif is only present in the CPDIIa sequence but absent in its CPDIIb duplication (Fig. 4a). Furthermore, some CPDIIb duplicates lack in addition the CPDII mitochondrial target motif (mts), which consists of the sequence IHGFLRMYWAK (Fig. 4a). Nucleotide alignments of the CPDIIa and CPDIIb duplicates of the different species further confirm that they are true duplicates because the associated nucleotides of each duplicate align better between the species than between each other (Supplementary file 8). For example, the *B. dorsalis* CPDIIa sequence XP_011207519.3, aligns better with the *B. oleae* CPDIIa sequence XP_014095123.2 than with the *B. dorsalis* CPDIIb duplicate XP_011207520.2. In addition, we analyzed the coding regions of the photolyases CPDIIa and CPDIIb of *B. dorsalis* for their composition and found that both sequences are located directly adjacent to each other on chromosome 3. This shows that these are actual duplications and not isoforms of a gene where they would share the same gene span.

Also, in the case of the blue-tailed damselfly *Ischnura elegans*, we searched for the coding regions of CPDIIa and CPDIIb photolyases on their assembly. In this case, both photolyases are found inverted on the second chromosome more than 30 million base pairs apart, proving that they are true duplicates. These two protein sequences differ from each other not only in the cc1 motif, but additionally in the CPDII antenna binding (cab) motif, which is present in XP_046383389.1, but not in XP_046383944.1 (Fig. 4b). In the dragonfly *Ladona fulva,* the situation looks similar but is less clear because the two *Ladona* sequences found are incomplete. While KAG8236888.1 lacks the C-terminal end, KAG8231874.1 seems to consist only of this (Fig. 4b). Therefore, we cannot completely exclude that these are only two partial sequences from a single CPDII photolyase. All alignments are shown in more detail in Supplementary file 8.

### A closer look at rarely found CRY/PLs in insects

Our study discovered three DASH-CRYs and two CPDI PLs that are rarely found in insects. By further investigation of their protein and nucleotide sequences, we aimed to determine whether they were true insect genes, possibly the result of HGT (Horizontal Gene Transfer), or contaminants. Interestingly, both CPDI PLs are derived from predictions of whole genome shotgun analyses, and both seem to map in the microbiomes of both species. Thus, the whole genome shotgun sequence JABVZW010000398 from which the *Abscondita* CPDI PL is derived has 13 other protein sequences. All these protein sequences yielded results from the bacterium *Acinetobacter* in addition to *Abscondita* in BLASTP searches. Since *Acinetobacter* is a component of the microbiome of the firefly *Photuris versicolor*, we assume that it is also present in the microbiome of *Abscondita terminalis*. Similarly, the whole genome shotgun sequence of *Bradysia odoriphaga* has two more protein sequences, all of them showing hits from the bacterium *Serratia* in addition to *Bradysia* in a BLASTP analysis. *Serratia marcescens* is an intestinal microorganism that has already been identified in insects. Therefore, we conclude that with certainty neither *Bradysia* nor *Abscondita*, and thus probably also all insects, do not possess CPDI photolyase (Fig. 5).

The *Bradysia odoriphaga* DASH-CRY was also predicted by whole genome shotgun analyses (JAFDOW010000828.1). In addition to the DASH-CRY protein, 68 other proteins were identified in the same fragment. BLASTP analyses of the other proteins revealed insect hits in addition to *Bradysia* itself, suggesting that these are in fact genes present in the *Bradysia* genome. BLASTP analyses of DASH-CRY revealed DASH-CRY results from other arthropods (*Orchesella, Allacma, Daphnia, Nematostella*, etc.) in addition to the two *Bradysia* DASHs. In the case of *Bradysia coprophila* DASH, As the chromosome in which the gene is localized is also known (Chr4), this additionally is, a clear indication for no microbiome contamination. Again, BLASTP analysis of *Bradysia coprophila* DASH-CRY revealed similar results as that of *Bradysia odoriphaga* and thus fits our phylogenetic tree (Table 1).

**Table 1:** COUSIN_59_ scores of CRY/PL nucleotide sequences of *Bemisia tabacci* are given

	COUSIN_59_ with *Bemisia tabacci* CUPref	COUSIN_59_ with *Xanthoria parietina* CUPref	COUSIN_59_ with *Nicotiana tabacum* CUPref
*Bemisia tab 6-4 PL *	1.345	−0.583	0.819
*Bemisia tab CPDII PL*	0.909	−0.492	0.547
*Bemisia tab DCRY*	1.095	−0.268	1.098
*Bemisia tab MCRY*	0.571	−0.359	0.544
*Bemisia tab DASH-CRY*	0.473	−0.169	0.236

Here, the codon preferences of the CRY/PLs were compared with those of *Bemisia* itself, *Xanthoria*, and *Nicotiana*. Comparing codon usage with *Bemisia*, 6-4 PL, CPDII PL, and DCRY have an index of about 1 or above 1, predicting a CUPreference. MCRY has a slightly lower value of 0.571, but DASH-CRY has the lowest value of only 0.473, which again is closer to zero. Compared to codon usage in *Nicotiana*, the Cousin_59_ value of DCRY remains similar at 1.098. The MCRY value is also 0.544, which is only slightly lower than the comparison with *Bemisia*. The Cousin_59_ value of DASH-CRY is close to zero. Obviously, the codon usage of tobacco plant and *Bemisia* is quite similar. The Cousin_59_ values of 6-4 PL and CPDII PL range from 0.547 to 0.829, but both times significantly lower than compared to *Bemisia* CUPrefs. We found that all genes matched least with the codon usage of *Xanthoria parietina*, resulting in a Cousin_59_ of negative numbers, with DASH-CRY closest to 0. When the Cousin_59_ indexes are 0, the genes under study are considered to have no CUPref. When the Cousin_59_ indices are above 1, the genes are considered to have a clear preference, and when the values are negative, the CUPrefs in the query are even opposite to those in the reference (Bourret et al. 2019)

We used the Codon Usage Similarity Index Cousin_59_ (https://cousin.ird.fr/) to evaluate whether DASH-CRY from *Bemisia tabacci* had a specific codon usage preference that could indicate HGT (Bourret et al. 2019). Because we did not know the exact donor of DASH-CRY, we compared its nucleotide sequence with the CUPrefs (Codon Usage Preferences) of *Bemisia* itself (generated via https://cousin.ird.fr/) and with those of the yellow spruce *Xanthoria parietina*, as well as with those of the tobacco plant *Nicotiana tabacum* (available via https://www.kazusa.or.jp/codon/). *Nicotina tabacum* is one of the major hosts of *Bemisia tabacci* and thus the potential candidate host, while the DASH-CRYs of several *Xanthoria* species are similar to the DASH-CRYs of *Bemisia* according to BLASTP. We compared the distribution of Cousin_59_ indices of all cryptochrome genes of *Bemisia*. We detect negative Cousin_59_ values when comparing the CRY/PL sequences with CUPrefs of *Xanthoria*. Consequently, we tend to exclude *Xanthoria* as a donor. The highest Cousin_59_ values are obtained when comparing the sequences with the CUPref of *Bemisia*, whereby the Cousin_59_ values with *Nicotiana* are also quite high. The Cousin_59_ values of DASH-CRY are all three times rather towards 0. Therefore, we assume that *Bemisia* DASH-CRY does not have a unique CUPref, although it is closest to *Bemisia* CUPrefs.

## Discussion

As expected, insects show high variability in the presence of CRY/PL members. Besides the typical four insect CRY/PLs (MCRY, DCRY, 6-4 and CPDII photolyases) we found occasionally DASH-CRY and CPDI photolyase as well as a duplication in the CPDII photolyase. Furthermore, we found that different CRY/PL members were lost several times during insect evolution and that in rare cases some were regained by horizontal gene transfer (HGT), which is consistent with the results for all metazoans (Deppisch et al. 2022). With the large number of sequenced and annotated animal genomes belonging to the class Insecta, we were able to resolve the CRY/PL composition down to the individual genera and to analyze the loss of different CRY/PL family members in detail. Although this type of in silico analysis carries the risk of being erroneous or incomplete, the performed motif analyses and alignments appear to underline the accuracy of our results. As already outlined in the results section, in several cases, the lack of specific CRY/PL members is most likely due to incompleteness in sequencing and annotation, but in other cases certain CRY/PL members have indeed been lost. Here we will discuss the evolutionary reasons for certain CRY/PL losses and the conditions that may have made these losses and sometimes recoveries possible. We will also discuss whether the life history of individual species led to a certain CRY/PL composition and how specific CRY/PL members interact with each other. Special emphasis will be laid on flies (Brachycera), since in this order we find the only species that have lost MCRY, while other species possess DASH-CRYs and CPDI photolyases in addition to the main insect CRY/PLs.

### Loss of CRY/PLs

As previously described in Deppisch et al. (2022), primitive metazoans such as cnidarians, mollusks, echinoderms have very many CRY/PLs that appear to have been lost during evolution and during specialization of individual species. In contrast to *C. elegans*, in which no CRY/PLs were found (Romanowski et al. 2014; Deppisch et al. 2022), we did not detect any insect in our study that had no CRY/PLs at all. This may be due in part to our study design, in which we first searched for CRY/PLs and then assigned insects to them. However, to our awareness, there is not a single insect that is fully sequenced and annotated but did not appear in our study (Supplementary file 6). Therefore, based on current knowledge, we assume that all insects have at least one CRY/PL.

Even insects that have lost almost all CRY/PLs seem to retain at least MCRY. Several Insects living in dark niches appear to have lost the light sensitive DCRY in addition to both photolyases. In particular, the larval developmental site appears to be more important than the adult habitat for the loss of photolyases. For example, nocturnal moths such as the tobacco hawk moth *Manduca sexta* still possess all four CRY/PLs (Broadhead et al. 2017) probably because the larval stages can be exposed to strong sunlight while foraging on plants. On the other hand, termites and hymenoptera can afford the loss of most CRY/PL members, because their brood is well protected from light. The honeybee *Apis mellifera* is a long-standing social model insect in chronobiology that allows direct comparisons to mammals due to its almost mammalian-like clock (Rubin et al. 2006; Beer and Helfrich-Förster 2020). Nevertheless, as other Hymenoptera, honeybees still retain the CPDII photolyase that is absent in Eutherian mammals such as mice and humans. In this respect, the CRY/PL repertoire of ants is even more like that of Eutherian mammals. The loss of both photolyases in ants is possibly caused by the circumstance that ant queens, responsible for the reproduction, live isolated from light in the formicary and thus are not exposed to DNA damaging UV-radiation. The male ants, which have a short life span, are also exposed to little light. The ant workers, which are more likely to be subjected to daylight, do not transmit their genes. Due to their high numbers in an ant colony, they are probably also more dispensable, which means that their DNA damages do not affect the next generation. Therefore, we assume that at least the ants examined in our study, have lost the CPDII photolyase genes during evolution.

Another species that is barely exposed to sunlight at any developmental stage is the human body louse *Pediculus humanus corporis* (Phthiraptera). Like ants, this species has lost all CRY/PL members except MCRY. The body louse shows a putative co-evolution with humans (Kittler et al. 2003). The parasitic lifestyle and its hiding places in hairy areas and under human clothing seem to have facilitated the loss of most CRY/PL members. It would be most interesting to analyze the possible ancestor of *Pediculus humanus*, or even earlier ancestors that may not have lived parasitically, at least not with humans. However, *Pediculus humanus corporis* is the only representative of this order that has been sequenced and annotated so far. The bed bug *Cimex lectularius,* is another example for a lifestyle without bright light exposure. This bug has lost DCRY and the 6-4 photolyase, retaining MCRY and the CPDII photolyase.

More difficult to understand is the loss of most CRY/PLs in beetles because many species live under bright light. All sequenced species of the Tenebrionoidea, Elateroidea, Scarabaeoidea and Staphylinoidea retain only MCRY, and all other sequenced beetle species (except *Agrilus planipennis*) possess MCRY and the CPDII photolyase. One reason why they can survive in the sunlight might be the unique thick carapace of adults, which is often dark-pigmented and light-tight. Furthermore, the larvae of Tenebrionoidea, Elateroidea, Scarabaeoidea and Staphylinoidea develop underground completely shielded from the light. Most interestingly, the emerald ash borer, *Agrilus planipennis* differs greatly from all other beetles in that it has all four CRY/PLs. Perhaps this is due to the lifestyle of the invasive beetle, which feeds on ash leaves and is mainly active on warm and sunny days. Furthermore, it lays its eggs in bark crevices of ash trees on their sunny side. Therefore, the rather unpigmented eggs, which hatch only after 12–19 days depending on the temperature, must be protected from the sun. After hatching, however, *Agrilus planipennis* spends most of its life as a larva, feeding on the phloem of the ash tree.

### Gain of CRY/PLs

While CRY/PL loss happens very frequently, their recovery seems to occur only in rare cases. In our study, a total of 340 insects were examined, of which only four species showed recovery of DASH-CRYs and/or CPDI photolyases. DASH-CRYs were found in the whitefly *Bemisia tabaci* (Aleyrodoidea, Hemiptera) and the fungus gnats *Bradysia coprophila* and *Bradysia odoriphaga* (Sciaroidea, Nematocera), while CPDI photolyases were present in *Bradysia odoriphaga* and in the firefly *Abscondita terminalis* (Coleoptera). The nucleotide analysis of both CPDI photolyases revealed that they are contaminations derived from microbiome bacteria.

In the case of DASH-CRY the situation appears different. The original arthropods had DASH-CRY in addition to the four CRY/PLs. DASH-CRY still occurs in many crustaceans as well as in Collembola, a sister clade of insects (Fig. 5). In Fig. 5, we suggest that DASH-CRY has been lost in insects. However, the presence of DASH-CRY in the white fly and fungus gnats challenges our hypothesis. Could these insect species have retained the original DASH-CRY? If so, then DASH-CRY should have been lost later in individual orders, superfamilies, and even families, all except the Aleyrodoidea and Sciaridae themselves (Supplementary file 6). This scenario is rather unlikely as *Bradysia* is evolutionarily relatively recent (ca. 120 Ma) and the common ancestor of Collembola is ca. 500 Ma distant (Misof et al. 2014). Interestingly, all three insects that possess a DASH-CRY are known plant pest species. The close coexistence with their host plant seems to favor HGT and the uptake of extrinsic genes via HGT has already been described in several studies with *Bemisia* (Lapadula et al. 2020; Méteignier et al. 2021; Gilbert and Maumus 2022). However, the DASH-CRY of *Bemisia* clusters with that of a plant pathogenic fungus (next to an endophyte), which might indicate an infection with a fungus leading to a gene exchange (Kotwica-Rolinska et al. 2021; Deppisch et al. 2022). However, CUPref analyses indicate opposite codon usages of DASH-CRY nucleotide sequence when compared to the CUPrefs of fungi. But the Cousin_59_ scores are very similar when compared to *Bemisia* itself, but also when compared to *Nicotiana*, and both times close to 0. A similar finding was obtained in Gilbert and Maumus (2022). They too found that many possible transferred genes had a COUSIN index around 0 and suggested that the absence of CUPref may have facilitated the uptake of these genes. They further discuss whether these genes already lack CUPref in the donor species or whether the lack of CUPref developed only after transfer into the genome (Gilbert and Maumus 2022).

The DASH-CRYs of *Bradysia* cluster with those of arthropods and especially with those of Collembola, the sister class of insects (Supplementary files 4 and 5). *Bradysia* probably requires a hitherto unknown vector (bacterium, protists, fungi, etc.) that transferred the DASH-CRY sequence from Collembola to the fungus gnats. This hypothesis requires further investigation. Most interestingly, both fungus gnats are the only dipterans besides the turnip gnat *Contarinia nasturtii* (also belonging to Bibionomorpha) that do not possess DCRY highlighting their special position among dipterans. Perhaps the loss of DCRY made the reintroduction of DASH-CRY possible. The exact role of DASH-CRY in these insects and its link to DCRY is an intriguing question that should be also investigated in more detail in the future. Possibly, DASH-CRY provides some protection from light, seems to be needed in these insects that live on plants and are highly exposed to light. Recently, a possible HGT has also been reported for Plant CRY-like (PCRY-like) putatively for the same reason (Deppisch et al. 2022).

Nevertheless, we must emphasize that the common insect CRY/PLs MCRY, DCRY, 6-4 PL, and CPDII PL were never regained in any animals studied. Once lost, they appear to be truly gone. Possibly, the loss of a particular CRY/PL can also be compensated by gene duplications of another CRY/PL, such as that of CPDII photolyase that we will discuss in the next chapter.

### CPDII duplication

Flies of the superfamily Tephritoidea and two dragon flies carry a CPDII duplication (CPDIIa and CPDIIb). The second CPDII photolyase could serve as an additional protection against UV damage during the long developmental time of dragon flies. The females usually lay their eggs on floating plant parts at the water surface. The larvae take about a year to develop and must be protected from UV damage in the DNA during this time. In the case of fruit flies, the reason for the additional protection is less clear because they lay their eggs inside fruits, where they are more protected from sunlight. Also, pupation takes place underground, so mainly adult flies are exposed to UV radiation. The distant relationship of these species suggests an analogous and independent duplication. Obviously, gene duplications and deletions tend to occur randomly, and whenever these random mutations are of evolutionary advantage, they are retained and passed on. So far, the evolutionary advantage of the CPDII duplication in Tephritoid fruit flies is not completely clear. The Tephritoidea belong to the Schizophora that lost MCRY, but they split off relatively early. Considering that non-Schizophora flies still have MCRY, could it be that the Tephritoidea CPDII duplicate is a potential MCRY replacement? Evolutionarily, MCRY evolved from 6-4 photolyase and CPDII photolyase is very distant from it. Nevertheless, at least in the case of the CPDII photolyase of the long-nosed kangaroo *Potorous tridactylus*, it has been demonstrated that it cannot only repair DNA but also replace MCRY in MCRY-deficient mice (Chaves et al. 2011). Interestingly, *P. tridactylus* is also the only mammal seeming to lack MCRY (Deppisch et al. 2022). To understand the role of this CPDII duplication, further research is needed.

### Brachycera (Diptera): it works also without MCRY

DCRY is certainly the main cryptochrome of dipterans. Besides *Drosophila melanogaster*, we found 48 of 66 other species of the family Muscomorpha sharing the same CRY/PL distributions (Fig. 5, Supplementary file 6). The only Brachycera in our study that does not belong to the Infraorder Muscomorpha but to the Infraorder Stratiomyomorpha is the black soldier fly *Hermetia illucens*. Interestingly, it is also the only Brachycera that has a MCRY alongside DCRY, 6-4 and CPDII photolyases. Thus, it has a CRY/PL distribution more similar to that of Nematocera than Brachycera. Recently, also other flies belonging to the Infraorder Muscomorpha, (the robber fly *Machimus arthriticus*, the long-legged fly *Heteropsilopus ingenuus,* and the large bee-fly *Bombylius major*) were identified to possess MCRY (Kotwica-Rolinska et al. 2021). Since the genome of these flies are not annotated yet, they are missing in our study. This finding clearly indicates that MCRY is lost only in certain superfamilies of Muscomorpha.

### The standard clock: MCRY or DCRY?

Already in Deppisch et al (2022) it became clear that MCRY is more important for the circadian clock than DCRY. This insect-focused study further supports this assumption. This is because all insects studied (except for a few incompletely sequenced genomes) have MCRY, and very often MCRY is the only cryptochrome. Exceptions are only made by *Drosophila melanogaster* and its relatives. The fruit flies, in which especially the circadian clock has been extensively studied and still is, demonstrate that MCRY is not necessary for a functional clock. However, fruit flies also make clear that MCRY loss is a recent evolutionary invention rather than an original mechanism.

Not long ago, it seemed clear that there were two distinct groups in the Diptera. The Brachycera, which do not have MCRY, and the Nematocera, which have both DCRY and MCRY. This study and the studies by Kotwica-Rolinska et al. (2021) and Deppisch et al. (2022) clearly demonstrate that it is not as simple. Insects of the suborder Bibionomorpha (Nematocera) even lack DCRY, which seemed to be peculiar to dipterans. At the same time, the Brachycera *Hermetia illucens* (Brachycera, Suborder Stratiomyomorpha) seems still to possess the MCRY.

**Fig. 6:**
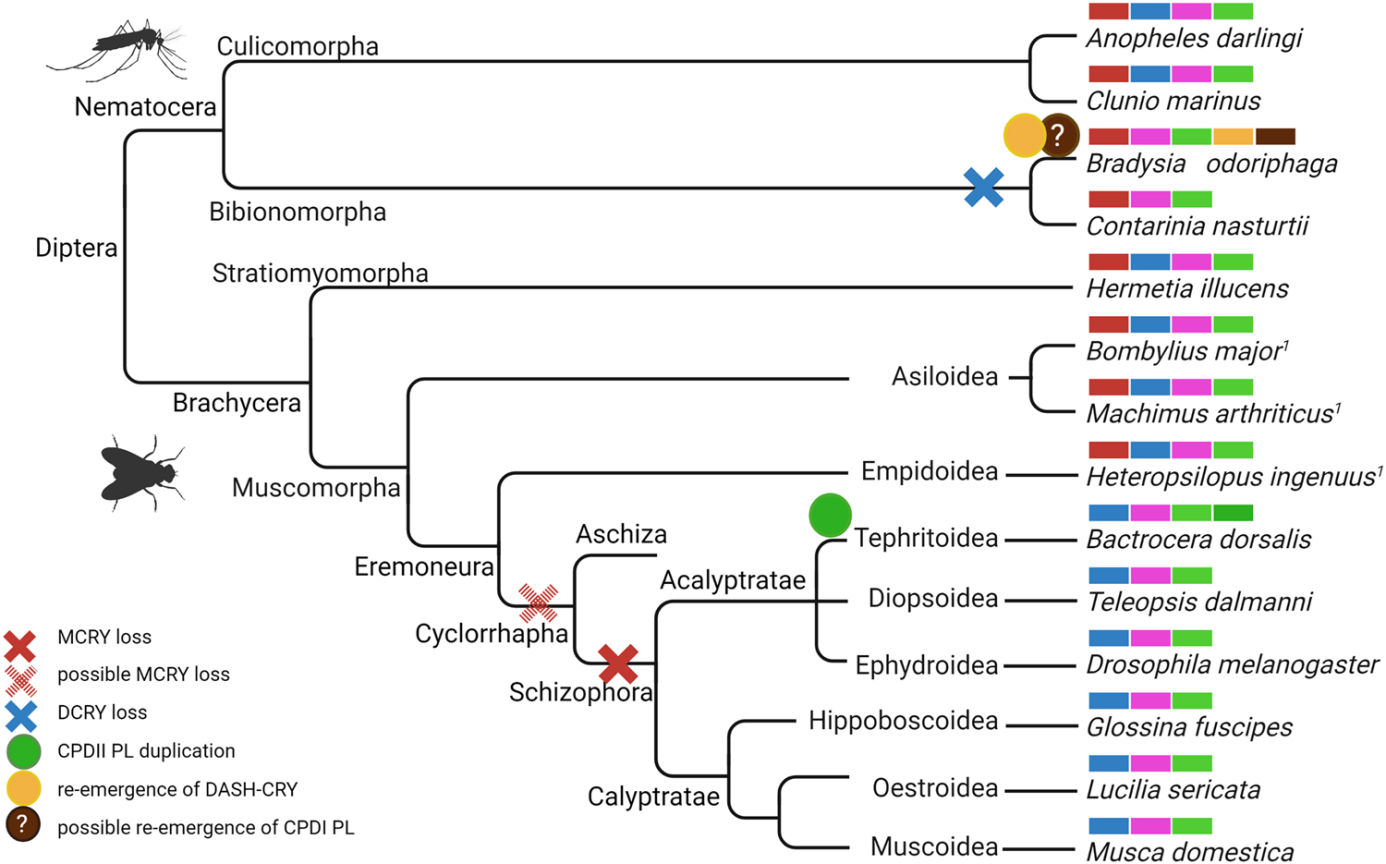
The phylogeny of dipterans. Diptera are divided into Brachycera (flies) and Nematocera (mosquitoes). While all nematocerans have MCRY, some brachycerans have lost it. However, this loss is only seen in the flies that belong to the section Schizophora. It is possible that the loss occurred one level earlier in Cyclorrhapha. However, since no fly of the sister section Aschiza has been studied so far, we cannot make any conclusion here

These results clearly indicate that flies that have only DCRY and no MCRY are obvious exceptions in insects, even in the whole animal kingdom (Deppisch et al. 2022). Having only DCRY seems even an exception among dipterans. So far only flies belonging to the clade Schizophora seem to lack MCRY (Fig. 6). The Schizophora comprise flies that hatch from their puparium by ejecting an inverted air sac, the ptilinium. This is a rather modern trait that evolved only about 65 Ma ago (Yeates and Wiegmann 1999; Wiegmann et al. 2011). Thus, we conclude that the loss of MCRY was also recent in evolution and that the well-known *Drosophila* clock is a quite modern achievement. It is also possible that MCRY has already been lost one taxonomic level higher in Cyclorrhapha. However, since all Cyclorrhapha studied so far belong to the subgroup Schizipohora and none to Aschiza (the second subgroup), we cannot make a definite statement here. But even in Schizophora, the fruit flies of the superfamily Tephritoidea having a CPDII photolyase duplication raise new questions. Most circadian clocks outside of Schiziphora appear to rely primarily on MCRY, whereas DCRY seems dispensable.

It is a remarkable coincidence that initial studies on the circadian clock have been conducted in this exceptional animal (Konopka and Benzer 1971). Clearly, this case highlights the need not to rely on just one model organism, but to take a broader view. Nevertheless, *Drosophila melanogaster* continues to be an indispensable member for circadian clock research. What molecular, neuronal, anatomical modifications make it possible for Schizophora to establish an intact circadian clock entirely without MCRY? Or is it their way of life that makes them the only ones in the entire animal kingdom to have a clock without MCRY? These questions bring the exceptional animal *Drosophila* back to the forefront and further research in it is essential.

### The interplay between CRY/PLs

Apparently, there is a yet unknown interplay between DCRY and the 6-4 photolyase and MCRY and the CPDII photolyase. The only insects in our study that have a DCRY, but no 6-4 PL are a few hemipterans and the German cockroach *Blattella germanica*, whereby incomplete sequencing cannot be ruled out in any of them. Even though there are many examples in the animal kingdom where MCRY (as in ants and in humans) and CPDII photolyase likewise (especially in nematodes and platyhelminths) may function alone, both seem to cooperate with each other as well. In species where we only detect MCRY, their related species often seem to have MCRY and CPDII photolyase. It seems that the other CRY/PLs are lost first and only at the end the CPDII photolyase. For example, all other hymenopterans still have MCRY and CPDII photolyases, while ants have lost CPDII in addition. Similarly, while Prototheria still have CPDII photolyase and MCRY, the Eutheria, to which we humans also belong, only have MCRY (Deppisch et al. 2022). This indicates a stepwise loss of CRY/PLs during evolution. The fact that DCRY occurs very frequently with 6-4 PL could possibly be attributed to its direct or indirect origin from 6-4 PL. However, why MCRY prefers to cooperate with the CPDII-PL rather than the 6-4-PL from which it is derived, and which is most similar to it in terms of sequence and motifs is not yet clear. In this context, we also admit that we are not aware of any molecular mechanisms that would explain an interplay between specific cryptochromes and photolyases. Nevertheless, we would like to emphasize the finding that especially the combination of MCRY and CPDII PL is very common across species. The fact that the *Potorous* CPDII photolyase can under certain circumstances take over the MCRY functions might give a hint. Also, the CPDII photolyase duplication of the Tephritoidea could add another hint.

## Conclusion

Our study highlights the importance of CRY/PLs for life under sunlight. While insects exposed to sunlight have at least one photolyase, some dark-adapted insects (e.g., the queen ant) can live entirely without one. Most notably, photolyases appear to be important for larval stages, which are exposed to sunlight during development. For circadian clocks, MCRY seems to be more important than DCRY. DCRY together with DTIM appears to play a not yet fully understood additional role. The *Drosophila* clock that works entirely without MCRY is a unique phenomenon. Furthermore, although a relatively large amount of research has been done on the four CRY/PLs found in insects, so far photolyases and cryptochromes have been seen as two independent protein groups, apart from their lineage history. The data from this study and from Deppisch et al. (2022) indicate that preferential partnerships exist between cryptochromes and photolyases, with reasons and advantages still unexplored.

## Supplementary Information

Below is the link to the electronic supplementary material.

Supplementary file1 (DOCX 337 KB)

Supplementary file2 (XLSX 80 KB)

Supplementary file3 (PDF 242 KB)

Supplementary file4 (PDF 202 KB)

Supplementary file5 (PDF 194 KB)

Supplementary file6 (XLSX 55 KB)

Supplementary file7 (ZIP 16298 KB)

Supplementary file8 (PDF 1261 KB)

Supplementary file9 (XLSX 77 KB)

Supplementary file10 (DOCX 762 KB)

## Data Availability

All data generated or analyzed during this study are included in this published article and its supplementary information files.
