# Supplementary material for: Contribution of cryptochromes and photolyases for insect life under sunlight
Source: J Comp Physiol A Neuroethol Sens Neural Behav Physiol. 2023 Jan 6;209(3):373–89. doi: 10.1007/s00359-022-01607-5 (PMC10102093; doi:10.1007/s00359-022-01607-5)
Supplement: Supplementary file 1 — Supplementary file1 (DOCX 337 KB) [file 359_2022_1607_MOESM1_ESM.docx]

>Abscondita_ter_hypoth_KAF5305339.1 KAF5305339.1

MQKQLIWFRQDLRIHDHAALWHARQSGTVIALVVLSPQQWQKHDDAPIKIDFYLRQIEVLQHALTKLNIPLIIQTIPLWE

NIATEVTQLCQKLNIENIHANIECGVNELNRDFDVQKRLNKNGHDLILYHDRTLFPVGSIRNKSNQPYQVFGAFKKYCYE

QLSISVPQCYPAPEVQSLDMSIYKDDLNPLPTTIQKSARSWRCVDRKKLPKTQWGKVRRKAHAWHLLDEFLDNHIENYQV

HRDFPNLEATSQLSAYLNIGIISIRQCLNALFRAQHGQFTIENIGQQTWMDELLWREFYQHILFDFPRVSKHLPFKQTTQ

NSIADAPKGSRSAWQMESRQAHPDWEQWFMQHLIDGDLAANNGGWQWCASTGTDSVPYFRIFNPVSQSQKFDAEGEYIRK

WVPELADLDAKTIHEPYAKNSEIQLNYPQPIVDLKQSRIRAIEAFKQI

>Abscondita_ter_hypoth_KAF5307814.1 KAF5307814.1

MSVVSGCAGPVSKGPEKHTVHWFRKGLRFHDNPALREDPWFAGSSNVGINKWRFLLQCLEDLDRSLRKMNSRLFVVRGQP

ADALPKLFKEWGTTSLTFEEDPEPFGRVRDHNIITICEELGITVVQRVSHTLYYLDRIIERNGGKAPLTYHQFQAVIASM

DPPPYAEPPVSNKDVVNITTPLNEDHDEKFGVPTLEELGFDTEGLLPPVWQGGESEALARLERHLERKAWVASFGRPKMT

PQSLLASQTGLSPYLRFGCLSTRLFYYQLTDLYKKIKKSFPPLSLHGQLLWREFFYCAATNNTNFDKMSGNPICVQIPWD

KNAKALAKWANGHTGFPWIDAIMTQLREEGWIHHLARHAVVCFLTRGDLWLSWEEGMRVFEELLLDADWSVNAGMWMWLS

CSSFFQQFFHYYCPVKFGRKADPNGDYIKKYLPVLKNYPTQYIHEPWMAPENVQRASKCVIGKDYPLPIVNHAIASRINI

QRMKKVYQNLTKYRNVDQCNVPQKYNDPFENTISNVDSKNC

>Acromyrmex_cha_CRY1___KAG5329508.1 KAG5329508.1

MTGSSKNEMGQAVTSGVRGDGGKHTVHWFRKGLRLHDNPSLKEGLAGASTFRCVFVLDPWFAGSTNVGINKWRFLLQCLE

DLDCSLRKLNSRLFVIRGQPADALPKLFKEWGTTDLTFEEDPEPFGRVRDHNISALCKELGISVIQRVSHTLYRLDEIIE

RNSGKPPLTYHQFQNVVAGMDPPEPPVPTVTAACIGSAYTPLKDDHDDHYGVPTLEELGFDTENLLPPVWVGGESEALAR

LERHLERKAWVASFGRPKMTPQSLLPSQTGLSPYLRFGCLSTRLFYYQLTDLYKKIKKAVPPLSLHGQLLWREFFYCAAT

KNPNFDKMQGNPICVQIPWDKNVEALAKWANGQTGFPWIDAIMTQLREEGWIHHLARHAVACFLTRGDLWISWEEGMKVF

DELLLDADWSVNAGMWMWLSCSSFFQQFFHCYCPVRFGRKADPNGDYISRRYLPVLKNFPTRYIHEPWNAPLSIQHAAKC

IIGKEYSLPMVNHNKSSRINIERMKQVYQQLNKYRDNVCFAQGLLNALLAPPTKDGDEEKRKQDSPNRENEQKMETISSP

TQQQ

>Acromyrmex_ech_CRY-1_EGI61895.1 EGI61895.1

MTGSSKNEMGQAVTSGVRGDGGKHTVHWFRKGLRLHDNPSLKEGLAGASTFRCVFVLDPWFAGSTNVGINKWRFLLQCLE

DLDCSLRKLNSRLFVIRGQPADALPKLFKEWGTTDLTFEEDPEPFGRVRDHNISALCKELGISVVQRVSHTLYRLDEIIE

RNSGKPPLTYHQFQNVVAGMDPPEPPAPTVTAACIGSAYTPLKDDHDDHYGVPTLEELGFDTESLLPPVWVGGESEALAR

LERHLERKAWVASFGRPKMTPQSLLPSQTGLSPYLRFGCLSTRLFYYQLTDLYKKIKKAVPPLSLHGQLLWREFFYCAAT

KNPNFDKMQGNPICVQIPWDKNVEALAKWANGQTGFPWIDAIMTQLREEGWIHHLARHAVACFLTRGDLWISWEEGMKVF

DELLLDADWSVNAGMWMWLSCSSFFQQFFHCYCPVRFGRKADPNGDYIRRYLPVLKNFPTRYIHEPWNAPLSIQHAAKCI

IGKEYSLPMVNHNKSSRINIERMKQVYQQLNKYRDNGTSFKGENIDVSEAIDLFVDRVSFNEDIADSNKSGYSDLGFRVA

DVAGIGIPLVVMKLPGKHRSSELYSVTYTPHLPAVDVTSGEIQWPWQSVTTARFRNGLIALCGKSTQRSKIVSKFLLDNW

TNGEEKRLEGWPNLGNFLTLHRTIATTTNLT

>Acromyrmex_hey_CRY1___KAG5344869.1 KAG5344869.1

MTGSSKNEMGQAVTSGVRGDGGKHTVHWFRKGLRLHDNPSLKEGLAGASTFRCVFVLDPWFAGSTNVGINKWRFLLQCLE

DLDCSLRKLNSRLFVIRGQPADALPKLFKEWGTTDLTFEEDPEPFGRVRDHNISALCKELGISVVQRVSHTLYRLDEIIE

RNSGKPPLTYHQFQNVVAGMDPPEPPVPTVTAACIGSAYTPLKDDHDDHYGVPTLEELGFDTENLLPPVWVGGESEALAR

LERHLERKAWVASFGRPKMTPQSLLPSQTGLSPYLRFGCLSTRLFYYQLTDLYKKIKKAVPPLSLHGQLLWREFFYCAAT

KNPNFDKMQGNPICVQIPWDKNVEALAKWANGQTGFPWIDAIMTQLREEGWIHHLARHAVACFLTRGDLWISWEEGMKVF

DELLLDADWSVNAGMWMWLSCSSFFQQFFHCYCPVRFGRKADPNGDYISRRYLPVLKNFPTRYIHEPWNAPLSIQHAAKC

IIGKEYSLPMVNHNKSSRINIERMKQVYQQLNKYRDNVCFAQGLLNALLAPPTKDGDEEKRKQDSPNRENEQKMETISSP

TQQQ

>Acromyrmex_ins_CRY1___KAG5308993.1 KAG5308993.1

MTGSSKNEMGQAVTSGVRGDGGKHTVHWFRKGLRLHDNPSLKEGLAGASTFRCVFVLDPWFAGSTNVGINKWRFLLQCLE

DLDCSLRKLNSRLFVIRGQPADALPKLFKEWGTTDLTFEEDPEPFGRVRDHNISALCKELGISVVQRVSHTLYRLDEIIE

RNSGKPPLTYHQFQNVVAGMDPPEPPAPTVTAACIGSAYTPLKDDHDDHYGVPTLEELGFDTESLLPPVWVGGESEALAR

LERHLERKAWVASFGRPKMTPQSLLPSQTGLSPYLRFGCLSTRLFYYQLTDLYKKIKKAVPPLSLHGQLLWREFFYCAAT

KNPNFDKMQGNPICVQIPWDKNVEALAKWANGQTGFPWIDAIMTQLREEGWIHHLARHAVACFLTRGDLWISWEEGMKVF

DELLLDADWSVNAGMWMWLSCSSFFQQFFHCYCPVRFGRKADPNGDYIRRYLPVLKNFPTRYIHEPWNAPLSIQHAAKCI

IGKEYSLPMVNHNKSSRINIERMKQVYQQLNKYRDNVCFAQGLLNALLAPPTKDGDEEKRKQDSPNRENEQKMETISSPT

QQQ

>Acyrthosiphon_pis_NP_001164532.2_chryptochr NP_001164532.2

MTVAVHWFRNGLRLHDNPALIEAHNNAEKLITLFIFDETTFNPKWYGYNPMRFLLESLIDLNNNLALVGGRLYILQGNPV

NIFKMIKEKIGLHFITYEQDCAHLGRTRDEKVKSFCDENDIKCIETVSHTLWNPKSIIEKNGGVPPFTFKQFQNTAKQIG

HPPSPVGNVDWLSVIFEELPASIQDEFKNLHNPTPETFGIYPEVPENLTSTYRWYGGETRALEQLKERLEYEKEAFVNGF

YLPNQVNPDLLSPPSSLSAALRYGCLSIRKFYWELTKLFINKFEGDLLPMYSVTSQLIWRDYFYTMSIDNKNFGQIEDNP

ACISIPWNDIKIPENKKMLECWKTGKTGYPFIDAGMRQLMQEGWIHHVVRNSLASFLTRGDLWISWVEGLNHFMKYLLDA

DFSVCSGNWIWVSSSTFEQLLDCPLCVCPVSYGLRLDPSGEYIKRYVPELKNMPAQFLYEPWKCPESVQKQVGCIIGKDY

PNCIVDHTIASKGNRKKMLALRVSMTNENRVPHCCPSDREEVQKFMYLPDECMQQLLPLENQDSKAYDIYKSH

>Acyrthosiphon_pis_NP_001164573.1_chryptochr NP_001164573.1

MQNKHTVHWFRKGLRIHDNPSLREGLINAKTFRCIFILDPWFAGASNVGINKWRFLLQCLSDLDNSLKKLNSRLFVIKGQ

PAEALPKLFRQWGTTNFTFEEDPEPFGRVRDQNIKVMCSEMGISVITRCSHTLYQLDKIINVNGGKAPLTYHLFQKLLEC

IDPPERAVPSIDKEFLGNAFTPIKYDHDEIFGVPTLEELGFKEINNITRHVWVGGETEALIRLQCHLERKAFIASFGKPK

MTPQSLVASPTGLAPYLKFGCLSTRLFFSELNELYKKIRKSQPPLSLHGQLLWRDFFYCASTNNPNFDRMVGNPICVQIP

WDKNPRALSKWANGQTGYPWIDAIMIQLRQEGWIHCIARHAVACFLTRGDLWLSWEEGMKVFDELLLDADWSVNAGYWMW

YSCSSFYQEFIHCYCPVRFGRKVDPNGDYIRRYIPALNNMPNQYIHEPWLAPESIQFSANCIIGIDYPLPIVNHINASKI

NLERMKLAYQQLSNCQPQLENGKLILISSLRR

>Acyrthosiphon_pis_XP_001946012.1_cry-2 XP_001946012.1

MDKNDADVGRETTVHWFRKGMRLHDNPAFKLSCEAKNSNGERYKLRPIYILDPYFRKYIRAGANRWRFLQQSLVDLDTTL

RKLGTRLYVIRGLPHEVFPDLFAKWNVKLLTFELDTEPYARERDNQVEQLARKHGVKVEQKVSHTIYNTELVLRANGGSV

PMTYQKFVSVVGSMPTPRRPIPAPDMLPSECLLDDDLNNPEFDVPTLDELLTLKGFNPAELKPCLYPGGEKEALRRLEEY

MKNKTWVCKFEKPNTSPNSLKPSTTVLSPYMKFGCLSASHFYYRLKEVIGNSPHSKPPVSLIGQLYWREFYYTVGASTPN

FDKMVGNSICCQVPWDNNPDALEAWTNGKTGYPFIDAIMRQLRDEGWIHHLARHAVACFLTRGDLWISWEKGLAVFEELL

LDADWSMNAGNWMWLSASAFFHQFFRVYSPVAFGKKTDKSGDYIRKYIPELAKYPDQYIYEPWSAPKSIQERAGCVVGVH

YPKRVVVHEDVYKNNIAKMSLAYKSTKAGKSSNTKKSRDMSTSPDKKNIKKPKLK

>Acyrthosiphon_pis_XP_001949151.1_photolyase XP_001949151.1

MSGNEPLTKKFKKTISEVGTSKTSNFLNDVAAERNKTASSIMEFKFNKKRVRVLSEQKEVPEWAEGVIYWTFRDERIHDN

WALLYAQKLAIKNKVSLHITFCRLKQFLDCSLRHYKHIFQGLEELETECKSLDIQFHFLIGCAADILPDFVKKHKLGAII

VDFMPVREHISWTKQLADRIGSEVPVIQVDAHNIVPCWVASDKQEYSARTIRNKINNKLPEFLTEFPPVIKHPFRSTFKA

KPTNWDEADKTLEVDRSVVSVPGLKAGFKAGMSELEQFLKKRLPKYSTDRNNPVKDGLSKLSPWLHFGQISAQRCILEVS

KLSKQYPDSVAAYREEAIVRRELSDNFCFYNPKYDKIEGAPNWAQTSLNEHRKDKRMFVYTREELESSRTHDDLWNSAQI

QLVKEGKMHGFLRMYWAKKILEWTDTPDRALADAIYLNDKYSMDGRDPSGFVGCMWSICGIHDQGWKEREIFGKIRYMNY

AGCKRKFDINAFIARYGGMVHKYTKK

>Adelges_coo_CRY-1-_XP_050419660.1 XP_050419660.1

MDFEQPSKHTVHWFRKGLRLHDNPSLRDGLIGAKTFRCIFILDPWFAGASNVGINKWRFLLQCLEDLDNSLKELNSRLFV

IRGQPAEALPKLFKMWRTTNFTFEEDPEPYGRVRDENITAMCREMDISVITRVSHTLYKLDKIINMNGGKAPLTYHLFQK

LLEYIDPPDEAVPKVDKDFLADAITPVDFDHDDKFGVPTLEELGFEDPDTKARYIWKGGETEALIRLQRHLERKAYIASY

GKPNMTPQSLLASRTGLTPYLRFGCLSTRLFFSELNNLYRKVTTDGSLSTMRLVPSPDLSPRIRKARPPLSLHGQLLWRD

FFYCASTNNPNFDKMIDNPICVQIPWNSNIQALSKWANGQTGYPWIDAIMIQLRKEGWIHCIARHAVACFLTRGDMWLSW

EEGMKVFEELLLDADWSVNAGSWMWYSCSSFFQEFIHCYCPVRFGRKADPNGDYIRRYIPVLKNIPNRYIHEPWLAPQSV

QIAANCVIGNDYPLPIVNHVVASKINLERMKLAYQQLSNCQPRLENGKLILITNTTSYCGKGLQQ

>Adelges_coo_CRY-1-_XP_050432639.1 XP_050432639.1

MMSCLSSKCQQSTISVVNMTVGVHWYRHGLRLHDNPSLLEASKRSSKLIVVFIFDIINEDPELTGYNRMRFLLESLKDLD

ENLKVRGGRLYVLQGDPVAIFKRIQQEIGLDLITFEQDCEPIWKSRDQSVKAFCEENNVEWVEKVSHTLWDPKLIIQTNG

GVPPLTYESFQIIANKIGQPPRPVPNVDWMSVNFQTLPANILDEFNKELKDLTPEYFNIRPETAGLSNPHNRWYGGETKA

LEQLQNRLEFEKEAFVKGFYLPNQVNPDLLAPPTSMSAALRLGCLSIRKFYWELSKLFTKSFETNLLPQYSATSQLIWRD

YFYVMSVNNDNFNKVDGNPACIAIKWNDLDQGDNRKLLDCWINGQTGYPFIDAGMRQLMQEGWTHHVVRNAVACFLTRGD

LWISWTEGMKHFLKYLLDADWAVCSGNWIWVSSSTFEQLLDCPLCVCPVSYGMRLDPTGEYIRRYVPELRKLPGRYLYEP

WKCPKEVQNEVGCVIGQDYPERCVDHQQASCENRKKMQELRYSLMNEGDVPHCRPANAAEVQKFMCLSNDCMQELLANLQ

SEYGDMY

>Adelges_coo_CRY-2_XP_050432117.1 XP_050432117.1

MSNEETSIHWFRKGMRLHDNPSFKLAYESKNSAGQYYKLRPIYILDPYFRKHIRAGMNRWRFLQQSLVDLDLSLRKLGTR

LYVVRGLPEDVFPGLFTKWNVKLLTFELDTEPYARKRDEHVENLARQHKVKVEQKVSHTLYNTELVLRANKGSVPMTYQK

FVSVVSSMPEPRRPLSAPTILPAECLLNDDLENEEYDVPTLDELLTLKGFNPEDIKPCLYPGGEVEALRRLEEYMKNKTW

VCKFEKPNTAPNSLKPSTTVLSPYMKFGCLSSSLFYYRLKEVIGNSAHSKPPVSLIGQLYWREFYYTVGAATPNFDKMLG

NPICCQVPWDSNQEALEAWANGKTGYPFIDAIMRQLNDEGWVHHLARHAVACFLTRGDLWISWEQGLAVFEELLLDADWS

MNAGNWMWMSASAFFHQFYRVYSPVAFGKKTDKSGDYIRKYVPELIKYPDEYIYEPWSAPKSVQYRAGCVIGVDYPKRIV

VHEEVYKINITKMSLAYKNTKAIKEKNVHPAPSPEEKQKKKRKFK

>Adelges_coo_PL_XP_050437580.1 XP_050437580.1

MADESPPIKKSKLSTSDAGSSAQSNFLSTVSAQRKKTAPSVMEFNFNKKRVRVLSDVKEVPEWADGIIYWTFRDERIHDN

WAFLYAQKLALKNKVSLHITFCRLQKFLDCSLRHYKHIFEGLEELETECNSLNVQFHFLIGCAADVLPDFVRKHKLGAVV

VDFMPVREHMLWTKQLAQRLGSEVPVVQVDAHNIVPCWVASDKQEYAARTIRNKINSKLSEFLTEFPPVIKHPYGGKLKA

KPTDWNEADKTLEVDRSVGPVAGLKAGYKAGMNELEMFLKKRLPNYATARNNPVKDGLSKLSPWLHFGQISAQRCILEVS

KFSGKYKDSVAAYREEAIVRRELSDNFCFYNPKYDSIEGAPNWARTTLNDHKKDKRKYVYTREELEKSRTHDDLWNSAQL

QLVNQGKMHGFLRMYWAKKILEWTDTPERALADSIYLNDKYSMDGRDPSGFVGCMWSVCGIHDQGWKEREIFGKIRYMNY

EGCKRKFDINAFIVRYGGKIHKYTKK

>Aedes_aeg_CRY-1_XP_001648498.2 XP_001648498.2

MTVNNILWFRHGLRLHDNPSLLEALRNDGTGSESVRLYPIFIFDGESAGTKLVGFNRMKFLLESLADLDRQLREIGGQLY

VFRGNAVNVMRRLFEELNIQKLCFEQDCEPIWKARDDAIQNLCRMMDVKCVEKVSHTLWDPQQIIRTNGGIPPLTYQMFL

HTVDIIGKPPRPVAAPSFEFVEFGTIPSILAQEVKLQQVRNLSPEDFGIYYEGNPDISHQQWMGGETKALECLGHRLKQE

EEAFLGGYFLPTQAKPEFLVPATSMSAALRFGCLSVRMFYWCVHDLYEKVQANNQYRNPGGQHITGQLIWREYFYTMSVH

NPHYAEMEANPICLNIPWYEPKDDSLDRWKEGRTGFPMIDAAMRQLLAEGWLHHILRNITATFLTRGALWISWEAGVQHF

LKYLLDADWSVCAGNWMWVSSSAFEKLLDSSSCTSPIALARRLDPKGEYVRRYLPELKNLPTLYVHEPWKAPLDVQKECG

CIVGRDYPAPMIDLAAASRANANTMNSIRQKLMERGGSTPPHCRPSDVEEIRNFFWLPEDVVADC

>Aedes_aeg_CRY-1_XP_001655778.2 XP_001655778.2

MGMTKQHNLVHQQKTTNTATSTIGSGGTGTGTNSSIGTQKQQHQQKHTVHWFRKGLRLHDNPALREGLKDAVSFRCVFVI

DPWFAGSSNVGINKWRFLLQCLEDLDRNLRKLNSRLFVIRGQPADALPKLFKEWGTTCLTFEEDPEPFGKVRDHNISEMC

KELGIEVISAVSHTLYKLERIIEKNGGRAPLTYHQFQAIIASMDAPPQPEPAITLDTIANATTPQYEDHDDKYGVPTLEE

LGFETEGLKPPIWVGGETEALARLERHLERKAWVASFGRPKMTPQSLLASQTGLSPYLRFGCLSTRLFYYQLTDLYKKIK

KACPPLSLHGQLLWREFFYCAATKNPNFDKMAGNPICVQIPWDRNAEALAKWASGQTGFPWIDAIMTQLREEGWIHHLAR

HAVACFLTRGDLWISWEEGMKVFEELLLDADWSVNAGMWMWLSCSSFFQQFFHCYCPVKFGRKADPNGDYIRRYLPVLKN

FPTRYIHEPWNAPETVQRTAKCIIGKEYPLPMVNHAIASRANMERIKQVYQQLAKYRSPSSSFDSECPEKGGSAIAGVMT

AAKVQHMNASSLNDSPSPTTIMTNLNSSGNYMCRTIPPAQTDPNAKIITYHQNREGQQQLSQQQHRLRNQHQLVGHQPPG

SDQYSNLQGSAYVTIKPESPSMTVSNNNNDLINAHFNTLQDQLNNSMIPLGSTGSTKIETDSFEYERNQENLYNNQFKVE

YTDNYNSGYGMRNADFYGRGREDDSVEQPMKSSLIQPGNLPNQTKQLSENQMHIHHHNHLNNHQQRSAIADNHRRQQQQQ

QQPMQQPMQQSPISEEKLTSGD

>Aedes_aeg_CRY-2_XP_001658195.1 XP_001658195.1

MSSQQTVVHWFRKGLRLHDNPALLAAIRRCRDSPQQYALRPVFILDPAIVQWLRVGPNRWRFLQQTLADLDRNLRKINSR

LYVVRGNPVDVFPKLFQEWNVSFLTFEHDIEPYSIKRDATVEEQAKKHGVKIQIEKSLTIYDPDAILKMNAGRPPLTYQK

YGSLASTLKIPEPVATPKEIPSDCVPKQDSNERKRANCYDPPLLVELGVKEEDLGECKFPGGETEALRRLEDHMKRKSWV

CSFEKPNTSPNSLEPSTTVLSPYVKFGCLSARLFMRELQTVIKGQKHSQPPVSLIGQLMWREFYYCAAADEPNFDKMVGN

SICLHVPWDSNKEFLEAWTHGRTGYPFIDAIMRQLRQEGWIHHLARHAVACFLTRGDLWISWEEGQRVFEEFLLDADWAL

NAGNWMWLSASAFFHQYFRVYSPVAFGKKTDPEGKFIKKYVPELAKYPSGIIYEPWKASVDVQKKLGCIIGKDYPNRIVI

HEEIHKKNIQKMTEAYRKNKAVKEGIMKGDKDPEAPNAGGKRKSSSSSSPSSSKPTAKKPKLRETLIKYLKKK

>Aedes_aeg_PL_X2_XP_001653955.1 XP_001653955.1

MKKASSSKADGPAEKKPKVEGGPIKVDDFVEKFKTIRKETAKSILDFDFKKKRVRILSDAKEVEENKKGVIYWMSRDARV

QDNWAFLFAQKLALKNELPLHVCFSLVPKFLDATIRHYKFMLKGLEEVAKECESLNINFHMLTGMAKDTIPKFVKTHNIG

AVVCDFSPLRVPMKWVEDVRKSLPAEVPLCQVDAHNIVPLWVTSEKQEYAARTIRNKVNNNLNTYLTQFPPVIKHPHKAS

FKADSIDWVKLLDTIEVDRTVDEVDWAVPGYTGGIGVLQSFVEKRLRKFNAKRNDPTDDALSNLSPWFHFGQISVQRCVL

AVKKYGKGYSEGVAAFCEESIVRRELSDNFCYYNKNYDNLKGAYDWAQKTLDDHRKDKRTYVYSRDQLEQARTHDDLWNS

AQIQMVKEGKMHGFLRMYWAKKILEWTKTPEEALETAIYLNDRYQLDGRDPNGYVGCMWSIAGIHDQGWREREVFGKIRY

MNYEGCKRKFDVAAFVARYGGKVYKK

>Aedes_alb_CRY-1_XP_019553042.2 XP_019553042.2

MTVNNVLWFRHGLRLHDNPSLLEALRNDGGRSESVRLYPIFIFDGESAGTKLVGFNRMKFLLESLADLDRQLREIGGQLY

VFRGNAVNVMRRLFEELNIRKLCFEQDCEPIWKPRDDAIVNLCHSMDVKCVEKVSHTLWDPEQVIRTNGGIPPLTYQMFL

HTVNIIGKPPRSVAAPSFEFVEFGTIPSILAQEVKLQPIQNLSPEDFGIYYEGNPDISHQQWIGGETKALECLGRRLKQE

EEAFLNGYFLPTQAKPEFLGPATSMSAALRFGCLSVRMFYWCVHDLYEKVQANNQYRHPGGQHITGQLIWREYFYTMSVH

NPNYAEMEANPICLNIPWYEPKGDSLERWKEGRTGFPMIDAAMRQLLAEGWLHHILRNITATFLTRGALWISWEEGVQHF

LKYLLDADWSVCAGNWMWVSSSAFEKLLDSSSCTSPIALARRLDPKGEYVRRYLPELKNLPTLYVHEPWKAPLDVQKECG

CIVGRDYPAPMIDLARASNDNANKMNSIRQTLMKSGGDTPPHCRPSDVEEIRNFFWLPEDVVADC

>Aedes_alb_CRY-1-_XP_029724400.1 XP_029724400.1

MGMTKQQQSLGHQQKSNSTTTSVIGSGGGGGPGSGTNSSSATLKQQQTKHTVHWFRKGLRLHDNPALREGLRDAASFRCV

FVIDPWFAGSSNVGINKWRFLLQCLEDLDRNLRKLNSRLFVIRGQPADALPKLFKEWGTTCLTFEEDPEPFGKVRDHNIS

EMCKELGIEVISAVSHTLYKLERIIEKNGGRAPLTYHQFQAIIASMDAPPQPEPAITLDTIANASTPQCEDHDDKYGVPT

LEELGFETEGLKPPIWVGGETEALARLERHLERKAWVASFGRPKMTPQSLLASQTGLSPYLRFGCLSTRLFYYQLTDLYK

KIKKACPPLSLHGQLLWREFFYCAATKNPNFDKMAGNPICVQIPWDRNAEALAKWASGQTGFPWIDAIMTQLREEGWIHH

LARHAVACFLTRGDLWISWEEGMKVFEELLLDADWSVNAGMWMWLSCSSFFQQFFHCYCPVKFGRKADPNGDYIRRYLPV

LKNFPTRYIHEPWNAPETVQRTAKCIIGKDYPLPMVNHAIASRANMERIKQVYQQLAKYRSPSSSFDSECPEKGGSAIAG

VMTPAKVQHMNASSLNDSPSPTTIMTNLNSSGNYMCRTIPPAQSDPNAKIITYHQNNREVQQQQHRLQQQTQQHLVGGHQ

TPGSDQFTNLQGSAYVTIKPESPSMAVSNNNNNNNNDLINAHFNTLQDQLNNSMNPLGGTGSTKIETDSFEYERNQENLY

NNQFKVEYTDNYNSGYGMRNADFYGRGREDEPSVEQTIKSSLIQPATLQPNQAKQLGENQMHIHHHNHQNHHNHHQQRSA

IADNHRRQHQQHHHQQQQQPMQQSPSGEEKLTSGD

>Aedes_alb_CRY-2__XP_019549386.2 XP_019549386.2

MSSQQSTVIHWFRKGLRVHDNPALVAAIRRCYDSPQQHALRPIFILDPGIVQWLRVGPNRWRFLQQSLADLDKNLRKINS

RLYVVRGNPEDVFPRLFKEWKVSFLTFEHDIEPYSLKRDATVEKQAKEHGVKILIEKSLTIYDPDAILKMNSGRPPLTYQ

KYGSLAATLKIPQPVNAPQVPLPSESVPKQDVNERKMASCYDPPSLDELGVKEEDLGECKFPGGETEALRRLEEQMKRKS

WVCSFEKPNTSPNSLEPSTTVLSPYVKFGCLSARLFMRDLQSVLKGQKHSQPPVSLVGQLMWREFYYCAAAAEPNFDKMA

GNSICLQVPWDTNKEFLEAWTYGRTGYPFIDAIMRQLRQEGWIHHLARHAVACFLTRGDLWISWEEGQRVFEELLLDADW

ALNAGNWLWLSASAFFHQYFRVYSPVAFGKKTDPEGKYIKKYVPELAKYPSGIIYEPWKASLDVQKKLGCIIGKDYPKRI

VIHEEIHKKNIQKMTEAYRKNKAVKEGVMKGDKDPEAPSSSGKRKLSTPSSSATKKPKLRETLTKYLKKK

>Aedes_alb_PL-lik_XP_019563527.2 XP_019563527.2

MKKASSSKADGPAEKKAKVEGGPIKVDDFVEKFKTTRKETAKSILDFDFKKKRVRILSDAKEVDENKKGVVYWMSRDARV

QDNWAFLFAQKLALKNELPLHVCFSLVPKFLDATIRHYKFMLKGLEEVAKECESLNINFHMLTGLAKDTIPKFVKTHDIG

AVVCDFSPLRVPMKWVEDVHKSLPAEVPLCQVDAHNIVPLWVTSEKQEYAARTIRNKVNGNLGTYLTQFPPVIKHPHKAS

FKANPIDWVKLLDTIEVDRSVDEVDWATPGYTGGVGMLQSFVEKRLRKFNAKRNDPTDDALSNLSPWFHFGQISVQRCIL

AVKKYGKGFSEGVAAFCEESIVRRELSDNFCYYNKNYDNLKGAYDWAQKTLNDHRKDKRTYVYSREQLEQARTHDDLWNS

AQIQMVKEGKMHGFLRMYWAKKILEWTKTPEEALETAIYLNDRYQLDGRDPNGYVGCMWSIAGIHDQGWREREVFGKIRY

MNYEGCKRKFDVAAFVARYGGKVYKK

>Aethina_tum_CRY-1_XP_019869775.2 XP_019869775.2

MSGAGGCRSGGGTAAPKGGGLHRQQDKHMVHWFRKGLRLHDNPSLREGLRGAKTFRCVFVLDPWFAGSSNVGINKWRFLL

QCLEDLDRSLRKLNSRLFVIRGQPADALPKLFKEWGTTALTFEEDPEPFGRVRDHNITTLCQELGISVVQKVSHTLYHLQ

HIIDKNNGKAPLTYHQFLAVIACMKAPPKPELPVTANSLNGAHTPLSEDHDEKYGVPTLEELGFDTEGLNPPVWQGGESE

ALARLERHLERKAWVASFGRPKMTPQSLLPSQTGLSPYLRFGCLSTRLFYYQLTDLYKKIKKAFPPLSLHGQLLWREFFY

CAATKNPNFDKMLGNPICVQIPWDKNAEALAKWANGQTGFPWIDAIMAQLRQEGWIHHLARHAVACFLTRGDLWLSWEEG

MKVFEELLLDADWSVNAGMWMWLSCSSFFQQFFHCYCPIKFGRKADPNGDYIRKYLPVLKNMPIQYIHEPWTAPENVQRA

AKCIIGKDYPLPMVNHASASRINIQRMKQVYQQLANYRTLENVNCTSQESFKDGYQHQPPMATAFQQFEFLDPYLHATPD

DNFDDEFHMS

>Aethina_tum_PL_XP_019880902.1 XP_019880902.1

MTSAGSRFLNTVTKDIFLRSISRSRLGEGKSVVYYCFNVSRRRILSLNEDLVHKNGVVYLMSRDCRIDDNWAFLYSQKLA

LQNELPLFVCVLTKDFSSLYPTKRQLEFAMGGFRWLRGQCECKNVGFYFLNANVEDFIGVVLENDVSGVVIDFSPLRKPV

EWRDYLIRNLPKDVPLIEVDAHNIVPAWLASDKQELMARSIRSKIQKKLPEYLTGFPEVSIHEYSGKLSTPLNLVDIDDV

YEFYKPLQDVSEVDWAEPGSDSAVEVLLNFIKHRLYYYGIKSNDPSNEYTSDLSPWIHFGQISTQRVALEVTRLREVVKR

QVDKFLEELIVRRELAENYCLYNPNYDNINGAADWAIKTLNDHRSDERPYVYTKEQFEKALTHDEMWNSAQIQLVQTGKM

HGYMRMYWCKKILEWTETPEEAIEIALCLNDMYSLDGTDPNGFVGVMWSICCVHDQGWKERKIFGKIRYMVDYSLRKKFN

MESYCARFGRRLPENNNSTKKGAKRKRK

>Agrilus_pla_XP_018319593.2_photolyase XP_018319593.2

MASITPRVGSKVDWTKFTKENFISAIKESRSKEGERVEDFKFNKNRIRILSDCKEVKESSDGILYWMCRDCRVQDNWAML

FAQRLARKNKIPLFVCYFTKNGSELVPTRRHNNFLINGLKEVAEECSRLNIKFHLINGDPCKSLVDIVIRNNIGSVICEL

NPLRKPKKWQQTVKGMLPNDVSLVQVDAHNIVPVWITSSKQELAARTIRPKINRNLEEYLTGFPMVSKHEYAGELNLNNM

EYSFEDAIKSFESEKDVDLPEIKWAKPSGTTAGIEMLHLFLTTRLKNYATTSGDPSKDNLSRMSSWVNFGQLSAQRIALE

VENFKNSYKESVERYLEELIVRRELSDNYCFYNENYDNIEGADKWARTTLDIHRKDKREYIYTKEQFAQAKTHDEIWNAA

QVQVINEGRMHGYIRMYWCKKILEWTKSPEEAIEIGLHLNDTFALDGNNPNGFVGVMWSICGVHDHGWTERPIFGKIRFM

VFWSMRKKFDADAFCEQYGRRIDNATAPKAKKQSVSNIKQTYLNKKQSTKSHSESDNEEKVQEKKSRPRRNKNETSRLQP

KKKTKLK

>Agrilus_pla_XP_018321938.1_cry-2_X1 XP_018321938.1

MEPRQATVIHWFRKGLRLHDNPALLEAIQVAVSQQAFLRPLFILDPNIPKWMKIGPNRWRFLIQSLKNLNNSLKAVNSKL

YVVRGKPEKVFRRILLEWSVTLITFESDIEPFAKERDTKIEKLAKELNIKVIQRISHTIYNPDLILTKNFGNPPLTYQKF

ISLIEQFKKPINLAKHPPNVPDYCKPFADLLEKNNSNCYEIPTLQELGVNEEGLGENLYSGGETEALARMEKYLENAQWI

CKFEKPNTSPNSLEPSTTVLSPYLKFGCLSSRLFYNRLNDVIRGAYCTKPPVSLIGQLLWREFYYTVAAATPNFDKMVGN

SICYQIDWDTNERHLQAWKTGKTGYPFIDAIMRQLRTEGWIHHLARHAVACFLTRGDLWISWEEGLKVFEEFLLDADWAL

NAGNWLWMSASAFFHQFYRVYSPTAFGKKTDKKGDYIRKYVPELRKFPEAYIYEPWLASKNVQKEAGCVVGVDYPERIVI

HETVYKINIGRMSAAYKRNKKVNDNGKSAKSSNCSSNLKRKRK

>Agrilus_pla_XP_018331212.1_cry-1-like XP_018331212.1

MTGTIKHPSNQSPKKPVKHMVHWFRKGLRLHDNPALREGLRDATTFRCVFVLDPWFAGSSNVGINKWRFLLQCLEDLDSS

LKKLNSRLFVIRGQPADALPKLFKEWGINALTFEEDPEPFGRVRDHNISALCKEMGITVIQKTSHTLYNLDAIISRNGGR

APFTYHQFQTVIAGMDPPPTAEAAVTDKTLNGAVTPVNEDHDDRFGVPTLDELGFDTEGLLPPVWQGGETEALARLERHL

ERKAWVASFGRPKMTPQSLLASQTGLSPYLRFGCLSTRLFYYQLTDLYKKIKKAFPPLSLHGQLLWREFFYCAATKNPNF

DKMVGNPICVQIPWDKNPEALAKWANGQTGFPWIDAIMTQLREEGWIHHLARHAAACFLTRGDLWISWEEGMKVFEELLL

DADWSVNAGMWMWLSCSSFFQQFFHCYCPVKFGRKADPNGDYIRKYLPVLKNMPTQYIHEPWNASDAVQKAAKCIIGKDY

PLPIVNHTLASKINMERMKQVYQQLSKYRSFDTQKLNCISSSKLKDYQPSVVTVGNLKNLQI

>Agrilus_pla_XP_025837056.1_cry-1 XP_025837056.1

MHFIQYCTDIYVLAPLTDILGKLFISQSNTKSTIKLEVFAMQVSILWFRHGLRLHDNPALHAALKKKQPFLPIFIFDGET

AGTQLIGYNRMRFLCEALYDLDRQLKQLGGRLYYAKGAVVQIFTKIWQELGIGRLCFEEDCEPIWRARDDSVKIMCKDLG

IECVECVSHTLWDPKKIIETNGGQPPLTYQMFLYTVDILGDPPRPKDEPRWSRVKFSNPFPLLEKVLYSFQTLPNPEDFG

VFPEITETPLVRWIGGETQALKKLEERLKLEERAFRQGYYLPSQNTPDLLGPPASQSAALRHGCLSVRKFYWSIQDLFKV

IHGDRLPTVHSITGQLIWREYFYTMSVKNPNYAVMKENPICLDINWIQSTSTDYNIRLTSWKEGLTGYPFIDAIMRQLKA

EGWIHHMARNMVACFLTRGDLWISWEEGLQHFLKYLIDADWSVCAGNWMWVSSSAFEQLLDCSTCICPINFGKHFDPSGE

FIKRYVPELKKFPSTLIYEPWKASLEQQIAAECIVGKDYPNRIINHKEAAERNRLAMKTLREDLTKPPTHCCPSNENEVR

QFMWLQDTCLKHQYVSI

>Agrotis_ips_CRY_1_AFJ22638.1 AFJ22638.1

MLGGSVLWFRHGLRLHDNPSLHAALEEKGFPFFPIFIFDGETAGTKLVGYNRMRYLLEALDDLDNQFKKYGGRLIMLKGK

PNVVFRRLWEEFGIRKLCFEQDCEPVWRARDDSVKAACKEIGVVCKEHVSHTLWEPDTVIKANGGIPPLTYQMFLHTVTT

IGDPPRPVHDIDLRRVKFGSLPECFYNEFTVFDKAPKPEDLGVFLENEDIRMIRWVGGESTALKQMQQRLAVEHETFLRG

SYLPTHGNPDLLGPPISLSPALRFGCLSVRSFYWSVQDLFRKVHQGRLTTQSASHFITGQLIWREYFHTMSVNNPNYGQM

SGNPICLDIPWKNPEGDELKRWEDGRTGFPFVDATMRQLKTEGWLHHAARNTVASFLTRGTLWLSWEHGLNHFLKYLLDV

DWSVCAGNWMWVSSSAFEALLDSGECACPVRLGQRLDPSGEYVRRYVPELACMPVEYIYEPWKAPIDIQGRANCIIGKDY

PAPVVNHLVAAQRNKNTMKELRHILQKAPPHCCPSSEEEIRQFMWLNE

>Agrotis_ips_CRY_2_AFJ22639.1 AFJ22639.1

MEIDRDIQSNLFHTRRNVCKYMKMSASAETLPAPTVRPRAPLAAAAAPHASPAARRPGGKHIVHWFRKGLRLHDNPALKD

GLVDAATFRCVFIIDPWFASSSNVGINKWRFLLQCLEDLDSSLRKLNSRLFVVRGQPADALPKLFREWGTTALSFEEDPE

PYGRVRDHNIMSKCREVGITVTSRVSHTLYKLDQIIERNGGKAPLTYHQFQALIASMPPPPPAEAPISAKTLNGATTPVT

DDHDDRFGVPTLEELGFETEGLKPPVWIGGESEALARLERHLERKAWVASFGRPKMTPQSLLASQTGLSPYLRFGCLSTR

LFYYQLTELYKRVKRVRPPLSLHGQILWREFFYCAATRNPNFDRMEGNPICVQIPWEKNQEALAKWASGQSGFPWIDAIM

IQLREEGWIHHLARHAVACFLTRGDLWISWEEGMKVFDELLLDADWSVNAGMWMWLSCSSFFQQFFHCYCPVRFGRKTDP

NGDFIRRYIPALKNMPTRYIHEPWVAPESVQQAARCIIGRDYPMPMVDHAKASQVNIERIKQVYAQLAKFKPQGTLNPNA

VQRPNVMQSSPSPNSIITSINQSNFLCSQAPDQPATTPDIIPYKDNDVVFQKPMSHRSMKPSFKQVVIVQKKQSTNVIQT

VSQTKENYVVNRQLDVAFKMPRNDHLQPAKQENYDFKNLIINNYAQGYANSPEIFENDQTTKSELFAQQTLKINSFNYEK

QKFFLSSYPDNGVRKAAGHNEAPAPPPYASGIDHDTNMSFNNENKIENVKDEANDGTCIHPMSINDDGIISNENQINTSN

ECDNYSENENQK

>Agrotis_seg_CRY_1_AUG44605.1 AUG44605.1

MLGGSVLWFRHGLRLHDNPSLHAALEEKGFPFFPIFIFDGETAGTKLVGYNRMRYLLEALDDLDNQFKKYGGRLIMVKGK

PNVVFRRLWEEFGIRKLCFEQDCEPVWRARDDSVKSACKEIGVVCKEHVSHTLWEPDTVIKANGGIPPLTYQMFLHTVTT

IGDPPRPVDDIDFRRVKFGSLPECFYNEFTVFDKTPKPEDLGVFLENEDIRMIRWVGGETTALKQMQQRLAVEHDTFLRG

SYLPTHGNPDLLGPPISLSPALRFGCLSVRSFYWSVQDLFRKVHQGRLTTQSASHFITGQLIWREYFYTMSVNNPNYGQM

SGNPICLDIPWKNPEGDELQRWEEGRTGFPFVDAAMRQLKTEGWLHHAARNTVASFLTRGTLWLSWEHGLNHFLKYLLDA

DWSVCAGNWMWVSSSAFEALLDSGECACPVRLGQRLDPSGEYVRRYVPELARMPVEYIYEPWKAPIDIQERATCIIGKDY

PAPVVNHLAAAQRNKNAMKELRQILQKPPPHCCPSSEEEIRQFMWLNE

>Agrotis_seg_CRY_2_AUG44606.1 AUG44606.1

MKMSASAETLPAPTVRERAPPAPHASPAARRPGGKHIVHWFRKGLRLHDNPALKDGLVDAATFRCVFIIDPWFASSSNVG

INKWRFLLQCLEDLDSSLRKLNSRLFVVRGQPADALPKLFREWGTTALSFEEDPEPYGRVRDHNIMSKCREVGITVTSRV

SHTLYKLDQIIERNGGKAPLTYHQFQALIASMPPPPPAEAPISAKTLNGATTPVTDDHDDRFGVPTLEELGFETEGLKPP

VWIGGESEALARLERHLERKAWVASFGRPKMTPQSLLASQTGLSPYLRFGCLSTRLFYYQLTELYKRVKRVRPPLSLHGQ

ILWREFFYCAATRNPNFDRMEGNPICVQIPWEKNQEALAKWASGQSGFPWIDAIMIQLREEGWIHHLARHAVACFLTRGD

LWISWEEGMKVFDELLLDADWSVNAGMWMWLSCSSFFQQFFHCYCPVRFGRKTDPNGDFIRRYIPALKNMPTRYIHEPWV

APESVQQAARCIIGRDYPMPMVDHAKASQVNIERIKQVYAQLAKYKPQGTLNPNAVQRPNVMQSSPSPNSIITSINQSNF

LCSQAPDQPSTTPDIIPYKDNDVVFQKPMNHRSMKPSFKQVVIVQKKQSTNVIQTVSQTKENYVVNRQLDVAFKMPRNDN

LQSAKQENYDFKNLIINNYAQGYANSPEIFENDQTTKNELFAQQTLKINSFNYEKQKFYLSSFSDNGVRKAAGHNEAPAP

PPYSAVIDHDTNMTFNNENKIQNVKDEANDGTCIHPMLINDDGIISNENQNNTSKECENYSANENQK

>Ampulex_com_hypoth_KAG7199484.1 KAG7199484.1

MTGSRKSELGQGVAIHGDGRKHTVHWFRKGLRLHDNPSLKEGLVGASTFRCVFVLDPWFAGSTNVGINKWRFLLQCLDDL

DSSLRKLNSRLFVIRGQPTDALPKLFKEWGTTNLTFEEDPEPFGRVRDHNMTALCKELGISVVQRVSHTLYKLDEIIEKN

GGKPPLTYHQFQNVVASMDPPEPPVTTVTSACIGSAYTPLRDDHDDQYGIPTLEELGFDTEGLLPPVWVGGESEALARLE

RHLERKAWVASFGRPKMTPQSLLPSQTGLSPYLRFGCLSTRLFYYQLTDLYKKIKKAVPPLSLHGQLLWREFFYCAATKN

PNFDRMHGNPICVQIPWDKNAEALAKWATGQTGFPWIDAIMTQLREEGWIHHLARHAVACFLTRGDLWISWEEGMKVFDE

LLLDADWSVNAGMWMWLSCSSFFQQFFHCYCPVRFGRKADPNGDYIRRYLPVLKNFPTRYIHEPWNAPLSVQRVSKCIIG

KEYSLPMVNHSKSSRINIERMKQVYRQLNKYRDNGKCLLNSTVPASAKEKIEEPLMESRIGQENHQNVESINLSAQQQKQ

QQQQQSQ

>Amyelois_tra_PREDIC_XP_013183955.1 XP_013183955.1

MRIFSQSLLLRNTLVKMASAPKRIKLSSSSTSTENETKTDVEGFMKSIQDKREETAESILKYKFNKKRVRIISQEQLVLD

NCEGIVYWMSRDSRVQDNWAFLFAQKLALKNEVPLHVCFCIIAKYLDASVRQFHFLLRGLEKVAADCQKLNISFHLLEGS

GADALPQWVEKHNIGAVVCDFNPLRVPLGWLDGVKKKMKKDVPLIQVDAHNVVPCWVASDKQEYSARTIRKKITSKLDEF

LTEFPPVIKHPYTSKFKPEPIDWAEAIESREADKTVGPVDWAKPGYDEAIKMLKSFLDKRLKIFASKRNDPTVDALSNLS

PWFHFGQISVQRVALCVQEYKSKHTESVNSFLEEAIVRRELADNFCFYSEHYDSIKGASAWAQKTLDDHRKDKRTHLYTL

EELSNAKTHDDLWNSAQLQLAKEGKMHGFLRMYWCKKILEWTPTPEDALKYAIYLNDHYSIDGRDPNGYVGCMWSICGIH

DQGWAERAVFGKVRYMNYDGCKRKFNIHAFIARYGGKAHKYVPKKNSDGKKSDK

>Amyelois_tra_PREDIC_XP_013195022.1 XP_013195022.1

MSAAPETLPASSSRLPAPPGPNGRHIVHWFRKGLRLHDNPALREGLNGAITFRCVFIIDPWFASSTNVGINKWRFLLQCL

EDLDSSLKKLNSRLFVVRGQPADALPKLFREWGTTALTFEEDPEPYGRVRDHNIMSKCREVGITVISRVSHTLYKLDKII

ERNGGKAPLTYHQFQALIASMPPPPPAEAAITAQMLNGATTPLTEDHDDRFGVPTLEELGFDVEGLKPPVWMGGESEALA

RLERHLERKAWVASFGRPKMTPQSLLASQTGLSPYLRFGCLSTRLFYYQLTELYKRVKQVRPPLSLHGQILWREFFYCAA

TRNPNFDRMEGNPICVQIPWEKNQEALVKWAGGKTGFPWIDAIMIQLREEGWIHHLARHAVACFLTRGDLWISWEEGMKV

FDELLLDADWSVNAGMWMWLSCSSFFQQFFHCYCPVRFGRKTDPNGDFIRRYIPALKNMPTRYIHEPWVAPESVQQAARC

IIGRDYPLPMVDHNKVSQINIERIKQVYSQLAKYKPQANINNAQLQRPNVMQSSPSPTSIIASINQSNYLCAQTPESTPH

IVQYKNAAIFQQPAKVLPRADSKQQQFKQVVIVQQIQNSNVSPATITSCAQTLDNYVINGQLATCKSNNTRIDPMKPPSK

QENYDFKNLAINNYTQEYANKEVYRNEQSNKNEDYQTSVKNSNYGYIKPKFYITAFPDKKNLQNSHSDSSQTFVVPGRTY

TATDNDNKEKRETNSCLITDGTYADREDKNKSASNENQK

>Amyelois_tra_PREDIC_XP_013199862.1 XP_013199862.1

MLGGSVLWFRHGLRLHDNPSLLSAIEDRGVPFFPIFIFDGETAGTKLVGYNRMRYLLEALDDLDGQFKKYGGRLIMVKGT

PNVVIRRLWEEFGIRKLCFEQDCEPVWRARDDSVKRACREIGVTCREHVSHTLWEPDTVIRANGGIPPLTYQMFLHTVAT

IGEPPRPVADADLHGIKFGTLPECFYEEFVVFDKVPKPEDLGIFLENEDIRMIRWVGGETAALKQMEQRLNVEYETFCRG

SYLPTHGNPDLLGPPISLSPALRFGCLSVRRFYWAVQDLFRKVHQGRLSSTHFITGQLIWREYFYTMSVNNPNYGQMAGN

PICLDIPWRNPEGDELQRWTEGRTGFPFVDAAMRQLRTEGWLHHAVRNTVASFLTRGTLWLSWEHGLNHFLKYLLDADWS

VCAGNWMWVSSSAFEALLDSGECACPVRLGRRLDPSGEYVRRYVPELARMPVDYIYEPWKAPIDVQERANCVIGKDYPGP

VVNHLAAAQTNRNAMKELRNILQKAPPHCCPSSEDEIRQFMWINEDQPVGTA

>Amyelois_tra_PREDIC_XP_013201110.1 XP_013201110.1

MAKSPSVIHWFRLDLRIHDNLALRNAINEAENRKHLLRPVYVLDPDITSKVGVNRLRFLLQSLQDLDANLRKLNTCLFII

RGKASEELPKLFDKWQVKYLTTQIDIDPEIIKEEELIEKIANEKDVFIVKRVQHTVYDAHSVLKKNNGSVPLTYQKFLSL

AQDIQVKDTIEITKQISDHCKPQDLSEACNVPTLSDLGIDESELKPSKYPGGETEGLKRLHLYMAKKQWVCKFEKPNSSP

NSIEPSTTVLSPYISHGCVSAKLFYHKLKEVESGLPHTMPPVSLMGQLMWREFYYTAGTGTENFDKMVGNKLCTQIPWGK

NDDHLKAWAQGRTGYPFVDAIMRQLKEEGWIHHLARHMVACFLTRGDLWISWEEGAKVFEDYLLDYDWSLNAGNWMWLSA

SAFFYKYFRVYSPVAFGKKTDKEGLYIRKYVPELKKYPAEYIYEPWKAPKNVQKAAGCIIGEGYPQRIVDHDKIHKENMQ

KMSIAYKVNKEKKSLKRPRS

>Anopheles_alb_CRY-1_XP_035782382.1 XP_035782382.1

MTINNILWFRHGLRLHDNPSRLEALKSDCQSSSGEPVKLFPVFIFDGESAGTRIVGYNRMKFLLESLADLDRQFRELGGQ

LLVFRGDSETVLRRLFEELNIKKLCFEQDCEPIWRERDERVTRLCETMDVKCVENVSHTLWNPNEVIQTNGDIPPLTYQM

FLHTVDIIGEPPRPVGAPDFEFVEFGRIPAILASELKLFQQSHMPGPEEFGLTYDGNADIAFQKWFGGETRALESLGARL

KQEEEAFREGYYLPTQAKPEILGPATSMSAALRFGCLSVRMFYWCVHDLFAKVQASSQFKCPIGQHITGQLIWREYFYTM

SVRNPHYGEMERNPICLNIPWYEPVDDSLVRWKEGRTGFPLIDAAMRQLLAEGWLHHILRNITATFLTRGGLWISWEAGL

QHFLKYLLDADWSVCAGNWMWVSSSAFERLLDSSKCTCPIALAYRLDPTGDYVKRYVPELANFPAHLVHEPWKATKEEQL

EYGCTIGQHYPAPMVDLNVVAKRNAHAMAKLREQLVNNGGSTPPHCRPSDIDEIRQFFWLADDVVPET

>Anopheles_alb_CRY-1-_XP_035774976.1 XP_035774976.1

MAKKETIIHWFRKGLRIHDNPALSVAVDKVRQNPTKYCLRPIFVLDPAIRKWLRVGPNRWRFLQQTLANLDENLRTINTR

LYVVRGNPVQIFPDLFQRWNVSLLTYEHDIEPYAVQRDKTVQEQARKHNVATHIEVSHTIFDPETIVKRNGGKPPLTYQK

YATVASSCKIPIPLPAPQKLPEKGIEPEKDSQERKNQSCYDPPTMSELDVDEGSLQECKFPGGETEALRRMEQCLSRKAW

ICSFEKPNTSPNSLEPSTTVLSPYLKFGCLSARLFYSRIQETIKGQKHSQPPVSLIGQIMWREFYYCVAAVTPNFDKMPG

NEICIQVDWDTNKEYLDAWTEGRTGYPFIDAIMRQLRQEGWIHHLARHAVACFLTRGDLWISWEEGQRVFEELLLDADWA

LNAGNWMWLSASAFFHQYFRVYSPVAFGKKTDPEGKYIKKYVPELARFPSGIIYEPWKANLETQKKLGCIIGKDYPKRIV

VHEEISKTNIQRMSAAYRRNKALKEGGQSEASVGEGAGSASPSKPSSKRTSSTGSSKTKPSPKKRKLEATITKFLKKK

>Anopheles_alb_CRY-1-_XP_035782470.1 XP_035782470.1

MGMTKQTSSGVGHYSGSGGHTGMNSMATGSGNADKQQQASSNSSLQQQQQQTSRHGQGSKGQAQHHLHQPLHAHHQLHHH

LQQQQHPTAGKLRDKHTVHWFRKGLRLHDNPALREGVRGATSFRCVFVIDPWFAGSSNVGINKWRFLLQCLDDLDRNLRK

LNSRLFVIRGQPADALPKLFKEWGTTCLTFEEDPEPFGRVRDHNISEMCKELGIEVISAASHTLYNLERIIEKNGGRAPL

TYHQFQAIIASMDAPPQPETTITLEAIGGATTPLYDDHDDKYGVPTLEELGFETEALRPPVWIGGETEALARLERHLERK

AWVASFGRPKMTPQSLLASQTGLSPYLRFGCLSTRLFYYQLTDLYKKIKKACPPLSLHGQLLWREFFYCAATKNPTFDKM

AGNPICVQIPWDRNSEALAKWASGQTGFPWIDAIMTQLREEGWIHHLARHAVACFLTRGDLWISWEEGMKVFEELLLDAD

WSVNAGMWMWLSCSSFFQQFFHCYCPVKFGRKADPNGDYIRRYLPVLKNFPTRFIHEPWNAPENVQRAAKCLIGKDYPLP

MVNHAIASRANMERIKQVYQHLAKYRTPGTCYEGDCVEKGGSAIAGVMTAAKMQHMNASSMNDSPSPTTILTSVNSSGNY

MCRSNPSAQSDPNAKVLTYHPLAMDGPASIEGRARSGSVVSGLGGGDNANDGSGNTMDGKGRVLTMPESALQQQQQQQRQ

QQQQQQHLQHHQQQQRHALQDGRSSNTVQEAYGNLPTSDYPTLKTDSGLATMIRGGNLTAGRFTNLQDQLSNSLLSLECE

VMATKLEPNNYEYERSQNMYNSQFKVEYSDNFNSGYGLRNAVFYGRKREDEQDNEQDEDEGTGDDDNDREESYNDEDDDR

LATIAMSPLQDTVAARSLDHYQPGEPHIKQEAKLLRSDMFREPKLLKGSSLRTRTEHAQQQQRAEQEERQCQHVCPQVSA

NERAEDKHTKRSAEQPSNSMLTDCDYDSDSHKMVTDYSRQQQQQHQQQQQQRQ

>Anopheles_alb_PL-lik_XP_035784326.1 XP_035784326.1

MKKAARPSSSGGKSSVEPPAKKPKKDEANNGSSTSSPLVDAGSSSNKSDDFVSLFRAERAATAKSILDFDFKKKRVRVLS

DAKVIEEGKEGVLYWMSRDVRVQDNWAFLFAQKLALKNELPLHVCFNLVPRFLEATIRHFKFMLKGLEEVATECEKLNIH

FHLLRGNAAQNVPAFVKKHRIGGVVCDFSPLRVPMQWVDDVRKTLPMEVPLCQVDAHNIVPVWVTSEKLEYAARTIRNKV

NNNLGTFLTPFPPVIKHPHKASFEAEPIDWPQVIDSLEVDRTVDEVKWATPGYTGGVATLQSFVEKRLGKFNGKRNDPTE

NALSNLSPWFHFGQIAVQRAILAVKSHGKRHTESVASFCEEAIVRRELSDNFCFYNKNYDNLKGAYDWARKTLDDHRKDR

RVYCYSREELETAKTHDDLWNSAQLQMVKEGKMHGFLRMYWAKKILEWTKSPEEALETAIYLNDRYSLDGRDPNGYVGCM

WSIAGIHDQGWKEREIFGKIRYMNYEGCKRKFDVNAFVMRYGGKVHRRK

>Anopheles_aqu_CRY-1_XP_050081660.1 XP_050081660.1

MTINNILWFRHGLRLHDNPSLLEALKSDCQSSSGEPVKLFPVFIFDGESAGTRVVGYNRMKFLLESLADLDRQFRELGGQ

LLVFRGDSETVLRRLFEELNIKKLCFEQDCEPIWRERDERVAKLCETMDVKCVENVSHTLWNPNEVIQTNGDIPPLTYQM

FLHTVDIIGEPPRPVGAPDFEFIEFGRIPAILASELKLFQQHHMPGPEEFGLAYDGNADIAFQKWFGGETRALESLGARL

KLEEEAFREGYYLPTQAKPEILGPATSMSAALRFGCLSVRMFYWCVHDLFAKVQASSQFKCPIGQHITGQLIWREYFYTM

SVRNPHYGEMERNPICLNIPWYQPVDDSLVRWKEGRTGFPLIDAAMRQLLAEGWLHHILRNITATFLTRGGLWISWEAGL

QHFLKYLLDADWSVCAGNWMWVSSSAFERLLDSSKCTCPIALAYRLDPTGDYVKRYVPELANYPAHLVHEPWKATKEEQL

EYGCTIGQQYPAPMVDLNVVAKRNAHAMAKLREELVNSGGSTPPHCRPSDIDEIRQFFWLADDVVPET

>Anopheles_aqu_CRY-1-_XP_050099989.1 XP_050099989.1

MGMTKQTSSGVGHYSGSGGHTGMNSMATGSGNADKQQQPSSNLPHHQQQQQQQQTSRHGQGSKGQAQHHLLQPLHAHHSL

HHHLQQQQQQHPVAGKLRDKHTVHWFRKGLRLHDNPALREGVRGATSFRCVFVIDPWFAGSSNVGINKWRFLLQCLDDLD

RNLRKLNSRLFVIRGQPADALPKLFKEWGTTCLTFEEDPEPFGRVRDHNISEMCKELGIEVISAASHTLYNLERIIEKNG

GRAPLTYHQFQAIIASMDAPPQPETTITLEAIGGATTPLYDDHDDKYGVPTLEELGFETEALRPPVWIGGETEALARLER

HLERKAWVASFGRPKMTPQSLLASQTGLSPYLRFGCLSTRLFYYQLTDLYKKIKKACPPLSLHGQLLWREFFYCAATKNP

TFDKMAGNPICVQIPWDRNSEALAKWASGQTGFPWIDAIMTQLREEGWIHHLARHAVACFLTRGDLWISWEEGMKVFEEL

LLDADWSVNAGMWMWLSCSSFFQQFFHCYCPVKFGRKADPNGDYIRRYLPVLKNFPTRFIHEPWNAPENVQRAAKCLIGK

DYPLPMVNHAIASRANMERIKQVYQHLAKYRSPGTCYEGDCVEKGGSAIAGVMTAAKMQHMNASSMNDSPSPTTILTSVN

SSGNYMCRSNPSAQSDPNAKVLTYHPLAMDGPASIDGRARSGSAVSGLAGGDNANDGSSNRLDGKGRLLTMQESVLQQQH

QRQQQQHQQQHLQQHQQQQRHALQDGRPSNKVQEAYGNLSTSDYPHLKTDSGLATMIRGGNLTAGRFTNLQDQLSNSLLS

LECEVMATKLEPNNYEYERNQNMYNSQFKVEYSDNFNSGYGLRNAVFYGRKREDEQDNEQDEDEGTGDDNNDREESYNDE

DDDRLATIAMSPLQDAVAARSLDHYQPSESQIKQEATPLHSDMFREPKLLKGSSLRTRTEQAQQQQRAEQEERQCENVHR

QPSTSERDEDKHTKRSVELPSNSMLTDCDYDSGSHKLVTNYSRQQQQQQQEQQHQSEVQLQNDQPMQDTAAKEEKLDCAD

>Anopheles_aqu_CRY-2_XP_050101182.1 XP_050101182.1

MAKKETIIHWFRKGLRIHDNPALSLAVDKVRQNPNKYCLRPIFVLDPAIRKWLRVGPNRWRFLQQTLVNLDENLRTINTR

LYVVRGNPVQIFPDLFDRWNVSLLTYEHDIEPYAVQRDKTVQEQARKHNVATHVEVSHTIFDPETIVKRNGGKPPLTYQK

YASVAAACKIPLPLPAPQKLPETGVEPEKDSQELKNHSCYEPPTMSELDVDEESLQDCKFPGGETEALRRMEQCLSRKAW

ICSFEKPNTSPNSLEPSTTVLSPYLKFGCLSARLFYSRIQEIIKGQKHSQPPVSLIGQVMWREFYYCVAAVTPNFDKMSG

NEVCIQVDWDTNKEYLDAWTEGRTGYPFIDAIMRQLRQEGWIHHLARHAVACFLTRGDLWISWEEGQRVFEELLLDADWA

LNAGNWMWLSASAFFHQYFRVYSPVAFGKKTDPEGKYIKKYVPELARFPSGIIYEPWKANLETQKKLGCIIGKDYPKRIV

VHEEISKTNIQRMSAAYRRNKALKEGGQSEASVSEDAGSATPSKSSSKRTSSNGSSKTKPSPKKRKLEATITKFLKKK

>Anopheles_aqu_PL_XP_050093347.1 XP_050093347.1

MCSRAFLINRVFVEQFIRNSLANMKKAARPSSSGGKSSVEPPAKKPKKDEPSTSSPPVDASGSSSKSDDFVALFRAERAA

TAKSILDFDFKKKRVRVLSDAKVIEEGKEGVLYWMSRDVRVQDNWAFLFAQKLALKNELPLHVCFNLVPRFLEATIRHFK

FMLNGLEEVAKECEKLNIHFHLLRGNAGQNVPAFVKKHRIGGVVCDFSPLRVPMQWVEDVREALPMEVPLCQVDAHNIVP

VWVTSEKLEYAARTIRNKVNNNLGTFLTPFPPVIKHPHKASFAAEPIDWQQVIDSLEVDRTVDEVKWATPGYTGGVATLQ

SFVEKRLGKFNAKRNDPTENALSNLSPWFHFGQIAVQRAILAVKSHGKRHTESVASFCEEAIVRRELSDNFCFYNKNYDN

LKGAYDWARKTLDDHRKDRRVYCYTREELETAKTHDDLWNSAQLQMVKEGKMHGFLRMYWAKKILEWTKSPEEALETAIY

LNDRYNLDGRDPNGYVGCMWSIAGIHDQGWKEREIFGKIRYMNYEGCKRKFDVNAFVMRYGGKVYRRK

>Anopheles_ara_CRY-1_XP_040162237.1 XP_040162237.1

MTINNILWFRHGLRLHDNPSLLEALKSDCVNQSSEAVKLFPIFIFDGESAGTRIVGYNRMKFLLESLADLDRQFRDLGGQ

LLVFRGDSVTVLRRLFEELNIKKLCYEQDCEPIWKERDDAVAKLCRTMDVRCVENVSHTLWNPIEVIQTNGDIPPLTYQM

FLHTVNIIGDPPRPVGAPNFEYVEFGRVPALLASELKLCQQMPAPDDFGIHYDGNARIAFQKWIGGETRALEALGARLKQ

EEEAFREGYYLPTQAKPEILGPATSMSAALRFGCLSVRMFYWCVHDLFAKVQSNSQFKYPGGHHITGQLIWREYFYTMSV

QNPHYGEMERNPICLNIPWYKPEDDSLTRWKEGRTGFPMIDAAMRQLLAEGWLHHILRNITATFLTRGGLWLSWEEGLQH

FLKYLLDADWSVCAGNWMWVSSSAFERLLDSSKCTCPIALARRLDPKGDYVKRYLPELANYPAQFVHEPWKASREQQIEF

GCVIGEKYPAPMVDLAIVSKRNAHTMASLREKLVDGGSTPPHCRPSDIEEIRQFFWLADDAATEA

>Anopheles_ara_CRY-1_XP_040165625.1 XP_040165625.1

MAKRETIVHWFRKGLRIHDNPALTVAVDKVRANPAKYCLRPIFVLDPGIRKWLRVGPNRWRFLQQTLANLDENLRSINSR

LYVVRGNPVEVFPKLFADWNVSLLTYEHDIEPYAVKRDSTVEEQARKHRVEVHIEKSHTIFDPEGIVKKNGGKPPLTYQR

YATLASAFKIPQPLPVPQKLPAKETSPEADKEERKNPSCYDPPTMEELDVEEASMGHCKFPGGESEALRRMNEILSRKAW

VCKFEKPNTSPNSLEPSTTVLSPYLKFGCLSVRLFYSRIAETIKGQKHSQPPVSLIGQVMWREFYYCVAAATPNYDKMVG

NGICTQIDWDTNKDYLEAWTHGRTGYPFIDAIMRQLRQEGWIHHLARHAVACFLTRGDLWISWEEGQRVFEELLLDADWA

LNAGNWMWLSASAFFHQYFRVYSPVAFGKKTDPEGKFIKKYVPELARFPAGIIYEPWKANLETQKKLGCIIGKDYPNRIV

VHEDISKVNIQRMSAAYKRNKAQKDGGTEEDDSVSSGSGGTPGKKRPSSTGSTAKGKAAPKKRKIEATIEKFLKKK

>Anopheles_ara_CRY-1-_XP_040158288.1 XP_040158288.1

MTKKTSTIGHFSASTSGTVGQTGGMSNFGTGTSGMESTDKQALSSSGQQAHQHHEHPSGIGGKGSEQQRPHQHQSQHHSQ

HHHHHSHHVQHSVGKMRDKHTVHWFRKGLRLHDNPALREGLRGARTFRCVFIIDPWFAGSSNVGINKWRFLLQCLDDLDR

NLRKLNSRLFVIRGQPADALPKLFKEWGTTCLTFEEDPEPFGRVRDHNISEMCKELGIEVISAASHTLYNLERIIEKNGG

RAPLTYHQFQAIIASMDAPPQPEAAITLDVIGNANTPQYDDHDDKYGVPTLEELGFETEALRPPVWIGGETEALARLERH

LERKAWVASFGRPKMTPQSLLASQTGLSPYLRFGCLSTRLFYYQLTDLYKKIKKACPPLSLHGQLLWREFFYCAATKNPT

FDKMAGNPICVQIPWDRNAEALAKWASGQTGFPWIDAIMTQLREEGWIHHLARHAVACFLTRGDLWISWEEGMKVFEELL

LDADWSVNAGMWMWLSCSSFFQQFFHCYCPVKFGRKADPNGDYIRRYLPVLKNFPTRFIHEPWNASESVQRAAKCLIGKD

YPLPMVNHAIASRANMERIKQVYQHLAKYRTPSGGCYEGDCTEKGGSAIAGVMTAAKVQHMNTSSMNDSPSPTTILTSVN

SSGNYMCRSNPPAQSDHNGKVITYHQLLSGPDPRSALGGNGSGGDRGNAGAPDDTKHGDGTDGSNHLMAGKESISQHQQS

QLQHSHQAQSLDQQSQENSNSVANSEQRYGTLPVAEYAAIKPDSLSTLIRAGNLTSAGTRFSNLQDQLSNSLMSLECDAL

ATKLKPNNYEYERNQNIYNSQFKVEYSDNFNSGYGLRNAVFYGGKREDENEDTKADNMNNNHSDQLAGDNSADERAVPND

TDDDDESTMTTLQATDAHSAPHALLFAANKSKIKQESLVQQSSRMFLEPPTHHASRGAKPHRSRCEATEARSHLADSQVK

KEQQITSQALPPHSMHTDCDYEPESHKLLAENYRQQHQQQQQQQQQQQQQHEQEQQQQQNSMLATQQRLEASQMDQGTDQ

PMQESPCNEEKIGAGD

>Anopheles_ara_PL_XP_040155359.1 XP_040155359.1

MFLVRASRFSIKCSLNHVPCRSLLLTGPNMKKSASSSSSTSSLEPAAKKQKPNDDPPKPSDKGSAAGREDYVAMLKAERK

ATAKSILDFDFNKKRVRILSDAKTIEDGKQGVLYWMSRDARVQDNWAFLFAQKLALKNDLPLHVCFNLVPKFLDATIRHF

KFMLKGLEEVAEECRKLNIQFHLLRGSAGENVPAFVKKHKIGGVVCDFSPLRVPMKWVDEVRKALPMEIPLCQVDAHNIV

PVWVTSDKLEYAARTIRTKVNKNLPTYLTPFPPLVKHPFTADFEADPINWTQVLDTLQVDRTVEAVEWATPGYTGGVKTL

QTFVEKRLGKFNDKRNDPTENALSNLSPWFHFGQIAVQRAVLTVKKHGKRYSESVASFCEEAIVRRELSDNFCFHNKNYD

NLQGAYDWARKTLDDHRKDKRVYCYSREELETAKTHDDLWNSAQLQMVKEGKMHGFLRMYWAKKILEWTKTPEEALETAI

YLNDRYSLDGRDPNGYVGCMWSIAGIHDQGWKEREIFGKIRYMNYDGCKRKFNVNAFVVRYGGKVHRRK

>Anopheles_col_CRY-1_XP_040234734.2 XP_040234734.2

MAKRETIVHWFRKGLRIHDNPALTVAVDKVRANPAKYCLRPIFVLDPGIRKWLRVGPNRWRFLQQTLANLDENLRSINSR

LYVVRGNPVEVFPKLFADWNVSLLTYEHDIEPYAVKRDSTVEEQARKHRVEVHIEKSHTIFDPEGIVKKNGGKPPLTYQR

YATLASACKIPQPLPVPQKLPAKETSPEADKEERKNPSCYDPPTMEELDIEEASMGQCKFPGGESEALRRMNEILSRKAW

VCKFEKPNTSPNSLEPSTTVLSPYLKFGCLSVRLFYSRIAETIKGQKHSQPPVSLIGQVMWREFYYCVAAATPNYDKMVG

NGICTQIDWDTNKDHLEAWTHGRTGYPFIDAIMRQLRQEGWIHHLARHAVACFLTRGDLWISWEEGQRVFEELLLDADWA

LNAGNWMWLSASAFFHQYFRVYSPVAFGKKTDPEGKFIKKYVPELARFPAGIIYEPWKANLETQKKLGCIIGKDYPNRIV

VHEDISKVNIQRMSAAYKRNKAQKDGGTEEDDSVSSGSGGTPGKKRPSSTGSTAKGKAAPKKRKIEATIEKFLKKK

>Anopheles_col_CRY-1_XP_040241143.1 XP_040241143.1

MTINNILWFRHGLRLHDNPSLLEALKSDCVNQSSEAVKLFPIFIFDGESAGTRIVGYNRMKFLLESLADLDRQFRDLGGQ

LLVFRGDSVTVLRRLFEELNIKKLCYEQDCEPIWKERDDAVAKLCRTMDVRCVENVSHTLWNPIEVIQTNGDIPPLTYQM

FLHTVNIIGDPPRPVGAPNFEYVEFGRVPALLASELKLCQQMPAPDDFGIHYDGNARIAFQKWIGGETRALEALGARLKQ

EEEAFREGYYLPTQAKPEILGPATSMSAALRFGCLSVRMFYWCVHDLFAKVQSNSQFKYPGGHHITGQLIWREYFYTMSV

QNPHYGEMERNPICLNIPWYKPEDDSLTRWKEGRTGFPMIDAAMRQLLAEGWLHHILRNITATFLTRGGLWLSWEEGLQH

FLKYLLDADWSVCAGNWMWVSSSAFERLLDSSKCTCPIALARRLDPKGDYVKRYLPELANYPAQFVHEPWKASREQQIEY

GCVIGEKYPAPMVDLAIVSKRNAHTMASLREKLVDGGSTPPHCRPSDIEEIRQFFWLADDAATEA

>Anopheles_col_CRY-1-_XP_040221177.2 XP_040221177.2

MTKKTSTIGHFSASTSGTVGQTGGMSNFGTGTSGMESIDKQALSSSGQQAHQHHEHPSGIGGKSSEQQRPHQHQSQHHHH

HSHQVQQSVGKMRDKHTVHWFRKGLRLHDNPALREGLRGARTFRCVFIIDPWFAGSSNVGINKWRFLLQCLDDLDRNLRK

LNSRLFVIRGQPADALPKLFKEWGTTCLTFEEDPEPFGRVRDHNISEMCKELGIEVISAASHTLYNLERIIEKNGGRAPL

TYHQFQAIIASMDAPPQPEAAITLDVIGNANTPQYDDHDDKYGVPTLEELGFETEALRPPVWIGGETEALARLERHLERK

AWVASFGRPKMTPQSLLASQTGLSPYLRFGCLSTRLFYYQLTDLYKKIKKACPPLSLHGQLLWREFFYCAATKNPTFDKM

AGNPICVQIPWDRNAEALAKWASGQTGFPWIDAIMTQLREEGWIHHLARHAVACFLTRGDLWISWEEGMKVFEELLLDAD

WSVNAGMWMWLSCSSFFQQFFHCYCPVKFGRKADPNGDYIRRYLPVLKNFPTRFIHEPWNASESVQRAAKCLIGKDYPLP

MVNHAIASRANMERIKQVYQHLAKYRTPSGGCYEGDCTEKGGSAIAGVMTAAKVQHMNTSSMNDSPSPTTILTSVNSSGN

YMCRSNPPAQSDHNGKVITYHQLLSGPDPRSALGGNGSGGDRGNAGAPDDTKHGDGTDGSNHLMAGKESISQHQQSQLQH

SHQAQSLDQQSQENSNSVANSEQRYGTLPVAEYAAIKPDSLSTLIRAGNLTSAGTRFSNLQDQLSNSLMSLECDALATKL

KPNNYEYERNQNIYNSQFKVEYSDNFNSGYGLRNAVFYGGKREDENEDTKADNMNNNHSDQLTGDNSADERAVPNDADDD

DESTMTTLQATDAHSAPHALLFAANKSKIKQESLVQQSSRMFLEPPKHHASRGAKPHRSRCEATEARSHLVDSQVKKEQQ

ITSQALPPHSMHTDCDYEPESHKLLAENYRQQHQQQQQQQQQQQQQHEHEQQQQQNSMLATQQRLEASQMDQGTDQPMQE

SPCNEEKIGAGD

>Anopheles_col_PL_XP_040226535.2 XP_040226535.2

MFLVRASRFSIKCSLNHAPCRFLLLTGPNMEKSASSSSSTSSLEPAAKKQKPNDDPPKPSDKGSAAGREDYVAMLKADRK

ATAKSILDFDFNKKRVRILSDAKTIEDGKQGVLYWMSRDARVQDNWAFLFAQKLALKNDLPLHVCFNLVPKFLDATIRHF

KFMLKGLEEVAEECRKLNIQFHLLRGSAGENVPAFVKKHKIGGVVCDFSPLRVPMKWVDEVRKALPMEIPLCQVDAHNIV

PVWVTSDKLEYAARTIRTKVNKNLPTYLTPFPPLVKHPFTADFEADPINWKQVLDTLQVDRTVEAVEWATPGYTGGVKTL

QTFVEKRLGKFNDKRNDPTENALSNLSPWFHFGQIAVQRAVLTVKKHGKRYSESVASFCEEAIVRRELSDNFCFHNKNYD

NLQGAYDWARKTLDDHRKDKRVYCYSREELETAKTHDDLWNSAQLQMVKEGKMHGFLRMYWAKKILEWTKTPEEALETAI

YLNDRYSLDGRDPNGYVGCMWSIAGIHDQGWKEREIFGKIRYMNYDGCKRKFNVNAFVVRYGGKVHRRK

>Anopheles_dar_CRY-1_XP_049533792.1 XP_049533792.1

MTINNILWFRHGLRLHDNPSLLEALKSDCQSSNEPVKLFPVFIFDGESAGTRVVGYNRMKFLLESLADLDRQFRELGGQL

LVFRGDSETVLRRLFEELNIKKLCFEQDCEPIWRERDERVTKLCETMDVKCVENVSHTLWNPNEVIQTNGDIPPLTYQMF

LHTVDIIGEPPRPVGAPDFEFIEFGRIPAILASELKLYQQQHMPGPEEFGLTYDGNADIAFQKWIGGETRALESLGARLK

QEEEAFREGYYLPTQAKPEILGPATSMSAALRFGCLSVRMFYWCVHDLFAKVQASSQFKCPIGQHITGQLIWREYFYTMS

VRNPHYGEMDRNPICLNIPWYEPVDDSLVRWKEGRTGFPLIDAAMRQLLAEGWLHHILRNITATFLTRGGLWISWEAGLQ

HFLKYLLDADWSVCAGNWMWVSSSAFERLLDSSKCTCPVALAYRLDPTGDYVKRYVPELVNYPAHLVHEPWKATKEEQLE

YGCTIGQQYPAPMVDLNVVAKRNAHAMSKLREELINNGGSTPPHCRPSDIDEIRQFFWLADDVVPEA

>Anopheles_dar_CRY-1_XP_049535035.1 XP_049535035.1

MAKKETIIHWFRKGLRIHDNPALSLAVDKVRQNPTKYCLRPIFVLDPAIRKWLRVGPNRWRFLQQTLVNLDENLRTINSR

LYVVRGNPIQIFPELFDRWNVSLLTYEHDIEPYAVQRDKTVEEHAREHNVTTHVEVSHTIFHPETIVKRNGGKPPLTYQK

YASLASTCTIPLPLPAPQKLPGKGVEPEKDSQERKDQSCYDPPTMNELDVDEGSLLECKFPGGETEALSRMEQCLSRKAW

ICSFEKPNTSPNSLEPSTTVLSPYLKFGCLSARLFYSRIQETIKGQKHSQPPVSLIGQMMWREFYYCVAAVTPNFDKMSG

NDVCIQVDWDTNKEYLDAWTEGRTGYPFIDAIMRQLRQEGWIHHLARHAVACFLTRGDLWISWEEGQRVFEELLLDADWA

LNAGNWMWLSASAFFHQYFRVYSPVAFGKKTDPEGKYIKKYVPELARFPSGIIYEPWKANLETQKKLGCIIGKDYPKRIV

VHEEISKTNIQRMSAAYRRNKALKEGGQSEASVSEEVGSGSTSKLSSKRTSSTVSSKTKPSPKKRKLEATITKFLKKK

>Anopheles_dar_CRY-1-_XP_049534686.1 XP_049534686.1

MGMTKQTSSGVSGHYSGSGGHTGMNSMATGSGNAEKQQQPSSNLPHQQQQQASRHGQGSKGQTQHQQHHLHQPLHAHHPL

HHHLQQQQQQQQQQQQQQHPTAGKLRDKHTVHWFRKGLRLHDNPALREGVRGATSFRCVFVIDPWFAGSSNVGINKWRFL

LQCLDDLDRNLRKLNSRLFVIRGQPADALPKLFKEWGTTCLTFEEDPEPFGRVRDHNISEMCKELGIEVISAASHTLYNL

ERIIEKNGGRAPLTYHQFQAIIASMDAPPQPETTITLEAIGGATTPLYDDHDDKYGVPTLEELGFETEALRPPVWIGGET

EALARLERHLERKAWVASFGRPKMTPQSLLASQTGLSPYLRFGCLSTRLFYYQLTDLYKKIKKACPPLSLHGQLLWREFF

YCAATKNPTFDKMAGNPICVQIPWDRNSEALAKWASGQTGFPWIDAIMTQLREEGWIHHLARHAVACFLTRGDLWISWEE

GMKVFEELLLDADWSVNAGMWMWLSCSSFFQQFFHCYCPVKFGRKADPNGDYIRRYLPVLKNFPTRFIHEPWNAPENVQR

AAKCLIGKDYPLPMVNHAIASRANMERIKQVYQHLAKYRSPGVCYEGDCVEKGGSAIAGVMTAAKMQHMNASSMNDSPSP

TTILTSVNSSGNYMCRSNPSAQSDPNAKVLTYHPLAMDGPTSIDGRARSGSVVSGLGTGDNANDGSSNTMDAKGRMLTMQ

ESVLQQQQQQHQRQQQLQRQQRHALQDGRPSNTVQEAYGNLPTSDYASLKTDSGLGTMIRGGNLTGGRFTNLQDQLSNSL

LSLECEVMATKLEPNNYDFERNQNMYNSQFKVEYSDNFNSGYGLRNAVFYGRKREDEQDNEQDEDEGTGDDNNDQEESYN

DEDDDRLATIAMSPLQDGVATRSLDHYQPGAPQIKQEATPLHSDMFREPKLLKGSSLRTRTEEHQQQRAEQEERQCEDVV

RPQASTNEHGEDKHTKRSAELPSNSMLTDCDYDSESHKMVTDYSRQQQQQQQQQRQSAVQLQNDQPMQDTAAKEEKLDCA

D

>Anopheles_dar_PL_XP_049542178.1 XP_049542178.1

MFGVCSTRALLINRVFLEQVISSARMMKKAARASSSGGKTSMEPPAKKPKKDETGNGSSTSGPVVEASGSTNKSDDFVAL

FRAERAATAKSILDFDFKKKRVRVLSDAKVIEEGKEGVLYWMSRDVRVQDNWAFLFAQKLALKNELPLHVCFNLVPRFLD

ATIRHFKFMLNGLEEVAKECEKLNIHFHLLRGNAGQNVPAFVKKHRIGGVVCDFSPLRVPMQWVEDVRKALPMEVPLCQV

DAHNIVPVWVTSEKLEYAARTIRNKVNNNLGTFLTPFPPVIKHPHKASFQAEPIDWPQVIDSLEVDRTVDEVKWATPGYT

GGVATLQSFVEKRLGKFNAKRNDPTENALSNLSPWFHFGQIAVQRAVLAVKSHGKRHTESVASFCEEAIVRRELADNFCF

YNKNYDNLKGAYDWARKTLDDHRKDRRVYCYTREELETAKTHDDLWNSAQLQMVKEGKMHGFLRMYWAKKILEWTKSPEE

ALETAIYLNDRFNLDGRDPNGYVGCMWSIAGIHDQGWKEREIFGKIRYMNYEGCKRKFDVNAFVVRYGGKVYRRK

>Anopheles_fun_CRY-1_XP_049278114.1 XP_049278114.1

MTINNILWFRHGLRLHDNPSLLEALKSDCINHSSEAVKLFPIFIFDGESAGTRVVGYNRMKFLLESLADLDRQFRDLGGQ

LLVFRGDSVTVLRRLFEELDIKKLCFEQDCEPIWKERDDGVTELCQKMDVRCVENVSHTLWNPNEVIQTNGDIPPLTYQM

FLHTVNIIGDPPRPVGAPNFEYIEFGRVPAILSSELKLCQQIPAPEDFGIFYEGNANIAFQKWIGGETRALEALGARLKQ

EEEAFREGYYLPTQAKPEILGPATSMSAALRFGCLSVRMFYWCVHDLFERVQSHSQFKYPGGHHITGQLIWREYFYTMSV

QNPYYGEMERNPICLNIPWYSPEDDSLVRWKEGRTGFPMIDAAMRQLMAEGWLHHILRNITATFLTRGGLWLSWEAGLQH

FLKYLLDADWSVCAGNWMWVSSSAFERLLDSSKCTCPIALARRLDPKGDYVKRYLPELANYPAQFVHEPWKASREQQIEY

GCVIGEHYPTPMIDLAVVSKRNAHTMASLRDKLVDGGSTPPHCRPSDIDEIRQFFWLADDAVTEA

>Anopheles_fun_CRY-1-_XP_049288542.1 XP_049288542.1

MTKKTSSIGHFSASTSGTVGGGTGGMSNFGTGTGGTDGSEKQQLSSSGSSHQQQQSQHHDGGKSSAQEQQRHHSSQHAHQ

HHHHQHHLQQGSGGKMRDKHTVHWFRKGLRLHDNPALREGLRGAKTFRCVFIIDPWFAGSSNVGINKWRFLLQCLDDLDR

NLRKLNSRLFVIRGQPADALPKLFKEWGTTCLTFEEDPEPFGRVRDHNISEMCKELGIEVISAASHTLYNLERIIEKNGG

RAPLTYHQFQAIIASMDAPPQPESSITLDVIGKSSTPQYDDHDDKYGVPTLEELGFETEALRPPIWIGGETEALARLERH

LERKAWVASFGRPKMTPQSLLASQTGLSPYLRFGCLSTRLFYYQLTDLYKKIKKACPPLSLHGQLLWREFFYCAATKNPT

FDKMAGNPICVQIPWDRNAEALAKWASGQTGFPWIDAIMTQLREEGWIHHLARHAVACFLTRGDLWISWEEGMKVFEELL

LDADWSVNAGMWMWLSCSSFFQQFFHCYCPVKFGRKADPNGDYIRRYLPVLKNFPTRFIHEPWNASESVQRAAKCLIGKD

YPLPMVNHAIASRANMERIKQVYQHLAKYRTPGGGCYEADCNEKGGSAIAGVMTAAKVQHMNTSSMNDSPSPTTILTSVN

SSGNYMCRSNPSAQSDPNGKIITYHQLLSGPDTRSAHGGNGSGDRANVGAAETTKHGDSRPTGNNHIMVGKESVPQQPQT

QHQLQHPAQSLDQQSQENSNSVANSEQRFGSMAVADYAAIKPDSLSTLIRAGNLSTVGTRFSNLQDQLSNSLMSLECDVM

ANKLKPNNYEYERSQNIYNSQFKVEYSDNFNSGYGLRNAVFYGGKREDENEDTKADNINNNIHHGDQLASNNRGEGQTMR

NAIDDDDESAMTTLEDTGTHPAPHALLFGTNKAKIKQESLVQTTRMFLEPPKHHPSRGTKVHQQRTRCESTDQQSPTLHT

QLDAQIKKEQQITSQPLPPQSMHTDCDYEPESHKQLLTDIHRQQQQQQQQQQQQQQQASILAAQRLEASQMDQGTDQPMQ

ESPCSEEKIGATD

>Anopheles_fun_CRY-2_XP_049280195.1 XP_049280195.1

MAKRETIVHWFRKGLRIHDNPALSIAVDKVRENPAKYYLRPVFVLDPGIRKWLRVGPNRWRFLQQTLVNLDENLRSINSR

LYVVRGNPVEIFPKLFTEWNVSLLTFEHDIEPYAVKRDKTVEDLAKKHNVKIHVEKSHTIFDPDTIVRKNAGKPPLTYQK

YATLASACKIPQALPVPKKLPANCGVPEPDKEERKNPTCYDPPTMKELGVKEGTISECKFPGGETEALRRMDEALSRKTW

VCNFEKPNTSPNSLEPSTTVLSPYLKFGCLSVRLFYSRVSEIIKGQKHSQPPASLIGQIMWREFYYCVAAVTPSFDKMAG

NNVCLQVDWDTNQDHLDAWTNGRTGYPFIDAIMRQLRQEGWIHHLARHAVACFLTRGDLWISWEEGQRVFEELLLDADWA

LNAGNWMWLSASAFFHQFFRVYSPVAFGKKTDPEGKFIRKYVPELARFPAGIIYEPWKANLETQKKLGCIIGKDYPQRIV

VHEEISKVNIQRMSAAYKRNKAQKEHDLDGDSAPGASSAGKKRPSSTKANAAPKKRKLEATIEKFLKKK

>Anopheles_fun_PL_XP_049296244.1 XP_049296244.1

MFCVRLIPFPLIECSSNPTSFRLLYPFAVSPSMKKSASSSSNSMEPATKKHKPNDGPPNDSKAVGGSSEDYAALFKADRK

ATAKSILDFDFSKKRVRILSDAKTIEDGKRGVLYWMSRDVRTQDNWAFLFAQKLALKNDLPLHVCFNLVPKFLDATIRHF

KFMLAGLEEVANECGKLNIQFHLLRGNACENVPEFVRKHKIGSVVCDFSPLRVPMKWVDDVKRSLPVEVPLCQVDAHNIV

PVWVTSDKLEYAARTIRNKVNNNLPTYLTPFPPLVKHPFTANFEANPINWTKVLDTLQVDRSVDAIEWATPGYIGGVKTL

QSFVEKRLGKFNDKRNDPTENALSNLSPWFHFGQISVQRAILTVKKYGKRYSESVASFCEEAIVRRELSDNFCFHNENYD

NLKGAYEWARKTLNDHRKDKRVYCYSRDELETAKTHDDLWNSAQLQMVKEGKMHGFLRMYWAKKILEWTKTPEEALETAI

YLNDRYSLDGRDPNGYVGCMWSIAGIHDQGWKEREIFGKIRYMNYEGCKRKFNVNAFVVRYGGKAHHRK

>Anopheles_gam_str.PEST_AGAP00_XP_321104.4 XP_321104.4

MTINNILWFRHGLRLHDNPSLLEALKSDCVNQSSEAVKLFPIFIFDGESAGTRIVGYNRMKFLLESLADLDRQFRDLGGQ

LLVFRGDSVTVLRRLFEELNIKKLCYEQDCEPIWKERDDAVAKLCRTMDVRCVENVSHTLWNPIEVIQTNGDIPPLTYQM

FLHTVNIIGDPPRPVGAPNFEYVEFGRVPALLASELKLCQQMPAPDDFGIHYDGNARIAFQKWIGGETRALEALGARLKQ

EEEAFREGYYLPTQAKPEILGPATSMSAALRFGCLSVRMFYWCVHDLFAKVQSNSQFKYPGGHHITGQLIWREYFYTMSV

QNPHYGEMERNPICLNIPWYKPEDDSLTRWKEGRTGFPMIDAAMRQLLAEGWLHHILRNITATFLTRGGLWLSWEEGLQH

FLKYLLDADWSVCAGNWMWVSSSAFERLLDSSKCTCPIALARRLDPKGDYVKRYLPELANYPAQFVHEPWKASREQQIEY

GCVIGEKYPAPMVDLAIVSKRNAHTMASLREKLVDGGSTPPHCRPSDIEEIRQFFWLADDAATEA

>Anopheles_gam_str_AGAP00_XP_313925.3 XP_313925.3

MFLVRAFRSSIKCSLNHAPCRFLLLTGPNMEKSASSSSSTSSLEPTAKKQKPNDDPPKPSDKGSAAGREDYVAMLKADRK

ATAKSILDFDFNKKRVRILSDAKTIEDGKQGVLYWMSRDARVQDNWAFLFAQKLALKNDLPLHVCFNLVPKFLDATIRHF

KFMLKGLEEVAEECRRLNIQFHLLRGSAGENVPAFVKKHKIGGVVCDFSPLRVPMKWVDEVRKALPMEIPLCQVDAHNIV

PVWVTSDKLEYAARTIRTKVNKNLPTYLTPFPPLVKHPFTANFEADPINWTQVLDTLQVDRTVEAVEWATPGYAGGVKTL

QTFVEKRLGKFNDKRNDPTENALSNLSPWFHFGQIAVQRAVLTVKKHGKRYSESVASFCEEAIVRRELSDNFCFHNKNYD

NLQGAYDWARKTLDDHRKDKRVYCYSREELETAKTHDDLWNSAQLQMVKEGKMHGFLRMYWAKKILEWTKTPEEALETAI

YLNDRYSLDGRDPNGYVGCMWSIAGIHDQGWKEREIFGKIRYMNYDGCKRKFNVNAFVVRYGGKVHRRK

>Anopheles_gam_str_AGAP00_XP_314748.3 XP_314748.3

MAKRETIVHWFRKGLRIHDNPALTVAVDKVRANPAKYCLRPIFVLDPGIRKWLRVGPNRWRFLQQTLANLDENLRSINSR

LYVVRGNPVEVFPKLFADWNVSLLTYEHDIEPYAVKRDSTVEEQARKHWVEVHIEKSHTIFDPEGIVKKNGGKPPLTYQR

YATLASACKIPQPLPVPQKLPAKETSPEADKEERKNPSCYDPPTMEELDIEEASMGQCKFPGGESEALRRMNEILSRKAW

VCKFEKPNTSPNSLEPSTTVLSPYLKFGCLSVRLFYSRIAETIKGQKHSQPPVSLIGQVMWREFYYCVAAATPNYDKMVG

NGICTQIDWDTNKDYLEAWTHGRTGYPFIDAIMRQLRQEGWIHHLARHAVACFLTRGDLWISWEEGQRVFEELLLDADWA

LNAGNWMWLSASAFFHQYFRVYSPVAFGKKTDPEGKFIKKYVPELARFPAGIIYEPWKANLETQKKLGCIIGKDYPMRIV

VHEDISKVNIQRMSAAYKRNKAQKDGGTEQDDSVSSGSGGTPGKKRPTSTGSTAKGKAAPKKRKIEANIEKFLKKK

>Anopheles_gam_str_AGAP00_XP_003436763.1 XP_003436763.1

MTKKTSTIGHFSASTSGTVGQTGGMSNFGTGTSGMESTDKQALSSSGQQAHQHHEHPSGIGGKSSEQQRPHQHQSQHQSQ

HHHHHSHHVQQSVGKMRDKHTVHWFRKGLRLHDNPALREGLRGARTFRCVFIIDPWFAGSSNVGINKWRFLLQCLDDLDR

NLRKLNSRLFVIRGQPADALPKLFKEWGTTCLTFEEDPEPFGRVRDHNISEMCKELGIEVISAASHTLYNLERIIEKNGG

RAPLTYHQFQAIIASMDAPPQPEAAITLDVIGNANTPQYDDHDDKYGVPTLEELGFETEALRPPVWIGGETEALARLERH

LERKAWVASFGRPKMTPQSLLASQTGLSPYLRFGCLSTRLFYYQLTDLYKKIKKACPPLSLHGQLLWREFFYCAATKNPT

FDKMAGNPICVQIPWDRNAEALAKWASGQTGFPWIDAIMTQLREEGWIHHLARHAVACFLTRGDLWISWEEGMKVFEELL

LDADWSVNAGMWMWLSCSSFFQQFFHCYCPVKFGRKADPNGDYIRRYLPVLKNFPTRFIHEPWNASESVQRAAKCLIGKD

YPLPMVNHAIASRANMERIKQVYQHLAKYRTPSGGCYEGDCTEKGGSAIAGVMTAAKVQHMNTSSMNDSPSPTTILTSVN

SSGNYMCRSNPPAQSDHNGKVITYHQLLSGPDPRSALGGNGSGGDRGNAGVPDDTKHGDGTDGSNHLMAGKESISQHQQS

QLQHSHQAQSLDQQSQENSNSVANSEQRYGTLPVAEYAAIKPDSLSTLIRAGNLTSAGTRFSNLQDQLSNSLMSLECDAL

ATKLKPNNYEYERNQNIYNSQFKVEYSDNFNSGYGLRNAVFYGGKREDENEDTKADNMNNNHSDQLTGDNSADERAVPND

ADDDDESTMTTLQATDAHSAPHALLFAANKSKIKQESLVQQSSRMFLEPPKHHASRGAKSHRSRCEATEARSHLADSQVK

KEQQITSQALPPHSMHTDCDYEPESHKLLAENYRQQHQQQQQQQQQQQQQHEHEQQQQQNSMLATQQRLEASQMDQGTDQ

PMQESPCNEEKIGAGD

>Anopheles_mac_CRY-1_XP_050069464.1 XP_050069464.1

MTINNILWFRHGLRLHDNPSLLEALRSDCMNQSSEAVKLFPIFIFDGESAGTRIVGYNRMKFLLESLADLDRQFRDLGGQ

LLVFRGDSVTVLRRLFEELNIKKLCFEQDCEPIWKERDDGVAQLCQKMDVRCVENVSHTLWNPTEVIQTNGDIPPLTYQM

FLHTVNIIGEPPRPVGAPNFEYVEFGRIPAILASELKLCHKIPVPEEFDIFYEGNANIAFQKWVGGETRALEALGARLKQ

EEEAFREGYYLPTQAKPEILGPATSMSAALRFGCLSVRMFYWCVHDLFEKVQAHSQFKYPGGHHITGQLIWREYFYTMSV

QNPYYGEMKRNPICLNIPWYTPEDDSLARWKEGRTGFPMIDAAMRQLLAEGWLHHILRNITATFLTRGGLWLSWEEGLQH

FLKYLLDADWSVCAGNWMWVSSSAFERLLDSSKCTCPIALARRLDPKGDYVKRYLPELTNYPVQFVHEPWKASREQQIEY

GCVIGEHYPAPMVDLASVSKRNAHTMASLRDKLVDGGSTPPHCRPSDIDEIRQFFWLADDAVTEA

>Anopheles_mac_CRY-1_XP_050077460.1 XP_050077460.1

MAKRETIVHWFRKGLRIHDNPALTVAVDRVRENPTKYSLRPIFVLDPAIRKWLRVGSNRWRFLQQTLVDLDENLRSINSR

LYVVRGNPVEVFPKLFTEWNVSLLTYEHDIEPYAVKRDATVEQQANKHHVQIHVEKSHTIFDPEVIVKKNAGKPPLTYQR

YATLASACKIPPALPAPQKLPAKCTAPASDNEERKNSACYDPPTTKELGVDENTLSSCKFPGGETEALRRMEEVLGRKAW

VCNFEKPNTSPNSLEPSTTVLSPYLKFGCLSVRLFYTRISDIIKGQKHSQPPVSLIGQIMWREFYYCVAAVTPNFDKMVG

NSVCMQIDWDTNKEHLEAWTHGRTGYPFIDAIMRQLRQEGWIHHLARHAVACFLTRGDLWISWEEGQRVFEELLLDADWA

LNAGNWMWLSASAFFHQFFRVYSPVAFGKKTDPEGKFIRKYVPELARFPAGIIYEPWKANLETQKKLGCIIGKDYPKRIV

MHEEISKANIQRMSAAYKRNKAQKETDLDGDSSPPVASTTGKKRPAASSSATKSGATPKKRKLENTIEKFLKKK

>Anopheles_mac_CRY-1-_XP_050068402.1 XP_050068402.1

MTKKTSSIGHFAATSTSGGAVGGTGGVNHFGTGTCGADGSDKQQIPSSGSNQPQQHHDGGKCTAQDQQRQHHHQHHHHQQ

HHLQQSGSGKLRDKHTVHWFRKGLRLHDNPALREGLRGTKTFRCVFIIDPWFAGSSNVGINKWRFLLQCLDDLDRNLRKL

NSRLFVIRGQPADALPKLFKEWGTTCLTFEEDPEPFGRVRDHNISEMCKELGIEVISAASHTLYNLERIIEKNGGRAPLT

YHQFQAIIASMDAPPQPEASITLDVIGKATTPQYDDHDDKYGVPTLEELGFETEALRPPVWIGGETEALARLERHLERKA

WVASFGRPKMTPQSLLASQTGLSPYLRFGCLSTRLFYYQLTDLYKKIKKACPPLSLHGQLLWREFFYCAATKNPTFDKMA

GNPICVQIPWDRNAEALAKWASGQTGFPWIDAIMTQLREEGWIHHLARHAVACFLTRGDLWISWEEGMKVFEELLLDADW

SVNAGMWMWLSCSSFFQQFFHCYCPVKFGRKADPNGDYIRRYLPVLKNFPTRFIHEPWNASESVQRAAKCLIGKDYPLPM

VNHAIASRANMERIKQVYQHLAKYRTPGSGCYEADCNEKGGSAIAGVMTAAKVQHMNTSSMNDSPSPTTILTSVNSSGNY

MCRSNPPAQSDPNGKIITYHQMLSGSAARSSLGGNGSGERSSAGAGEGNKHGDGTVGHNHLMTGKESIPQQQQQTQHHSH

QAQNLDQQSQENSNCLANSDQRFGSMADYAAIKPDSLSTLIRAGNLTTAGTRFSNLQDQLSNSLMSLECDVMATKLKPNN

YEYERNQNIYNSQFKVEYSDNFNSGYGLRNAVFYGGKHEDENEDTKADSMNNNNHSDELVGDNCGDEQGVHNDAEDDDDE

SPMTTLEDAGTHSAPHALLFGTDKSKIKQESLMSQSTRMFLEPPKHQSGREAKVHRARCESTDQQSNALHSSSLDAQIKK

EQQQITSQQLPAHSMHTDCDYEPDSHKLLADIHRHQQHHQQQQHEQQQHQQQQASLLAAQRLESSQMDQGTDQPMQESPC

SEDKIGATD

>Anopheles_mac_PL_XP_050074423.1 XP_050074423.1

MKKSASTTSNSSEPAAKKHKPNDTPPKQTVDKGDGGGHEDYVALFKADRKATAKSILDFDFSKKRVRILSDAKSVEDGKC

GVLYWMSRDVRVQDNWAFLFAQKLALKNDLPLHVCFNLVPKFLDATIRHFKFMLKGLEEVADECRKLNIQFHLLRGNAGD

NVPAFVKKHKIGGVVCDFSPLRVPMKWVDDVRQALPVEVPLCQVDAHNIVPVWVTSDKLEYAARTIRNKVNNNLPTYLTP

FPPLVKHPFAAKFEAEPINWTKVLDTLQVDRSVDAVEWATPGYVGGVKTLQSFVEKRLGKFNDKRNDPTENALSNLSPWF

HFGQISVQRAILTVKKFGKRYSESVASFCEEAIVRRELSDNFCFHNKNYDNLKGAYDWARKTLDDHRKDKRVYCYTREEL

ETAKTHDDLWNSAQLQMVKEGKMHGFLRMYWAKKILEWTKSPEEALETAIYLNDRYSLDGRDPNGYVGCMWSIAGIHDQG

WKEREIFGKIRYMNYEGCKRKFNVNAFVVRHGGKVHRRK

>Anopheles_mer_CRY-1_XP_041766708.1 XP_041766708.1

MTINNILWFRHGLRLHDNPSLLEALKSDCVNQSSEAVKLFPIFIFDGESAGTRIVGYNRMKFLLESLADLDRQFRDLGGQ

LLVFRGDSVTVLRRLFEELNIKKLCYEQDCEPIWKERDDAVAKLCRTMDVRCVENVSHTLWNPIEVIQTNGDIPPLTYQM

FLHTVNIIGDPPRPVGAPNFEYVEFGRVPALLASELKLCQQMPAPDDFGIHYDGNARIAFQKWIGGETHALEALGARLKQ

EEEAFREGYYLPTQAKPEILGPATSMSAALRFGCLSVRMFYWCVHDLFAKVQSNSQFKYPGGHHITGQLIWREYFYTMSV

QNPHYGEMERNPICLNIPWYKPEDDSLTRWKEGRTGFPMIDAAMRQLLAEGWLHHILRNITATFLTRGGLWLSWEEGLQH

FLKYLLDADWSVCAGNWMWVSSSAFERLLDSSKCTCPIALARRLDPKGDYVKRYLPELANYPAQFVHEPWKASREQQIEY

GCVIGEKYPAPMVDLAIVSKRNAHTMASLREKLVDGGSTPPHCRPSDIEEIRQFFWLADDAATEA

>Anopheles_mer_CRY-1-_XP_041763205.1 XP_041763205.1

MTKKTSTIGHFSASTSGTVGQTGGMSSFGTGTSGMESSDKQPLSSSGQQAHQHHEHPSGIGGKSSEQQRPHQHQSQHHQH

HHHHSHHVQQSVGKMRDKHTVHWFRKGLRLHDNPALREGLRGARTFRCVFIIDPWFAGSSNVGINKWRFLLQCLDDLDRN

LRKLNSRLFVIRGQPADALPKLFKEWGTTCLTFEEDPEPFGRVRDHNISEMCKELGIEVISAASHTLYNLERIIEKNGGR

APLTYHQFQAIIASMDAPPQPEAAITLDVIGNANTPQYDDHDDKYGVPTLEELGFETEALRPPVWIGGETEALARLERHL

ERKAWVASFGRPKMTPQSLLASQTGLSPYLRFGCLSTRLFYYQLTDLYKKIKKACPPLSLHGQLLWREFFYCAATKNPTF

DKMAGNPICVQIPWDRNAEALAKWASGQTGFPWIDAIMTQLREEGWIHHLARHAVACFLTRGDLWISWEEGMKVFEELLL

DADWSVNAGMWMWLSCSSFFQQFFHCYCPVKFGRKADPNGDYIRRYLPVLKNFPTRFIHEPWNASESVQRAAKCLIGKDY

PLPMVNHAIASRANMERIKQVYQHLAKYRTPNGGCYEGDCTEKGGSAIAGVMTAAKVQHMNTSSMNDSPSPTTILTSVNS

SGNYMCRSNPPAQSDHNGKVITYHQLLSGPEPRSALGGNGSGGDRGNAGAPDDTKHGDGTDGSNHLMAGKESISQHQQSQ

LQHSHQAQSMDQQSQENSNSVANSEQRYGTLPVAEYAAIKPDSLSTLIRAGNLTSAGTRFSNLQDQLSNSLMSLECDALA

TKLKPNNYEYERNQNIYNSQFKVEYSDNFNSGYGLRNAVFYGGKREDENEDTKADNMNNNHSDQLAGDNSADERAVPNDA

DDNDESTMTTLQATDAHSAPHALLFAANKSKIKQESLVQQSSRMFLEPPKHHASRGAKPHRSRCEAAEARSHLADSQVKK

EQQMTSQALPPHSMHTDCDYEPESHKLLAENYRQQHQQQQQQQQQQQHEHEHEQQQQQNSMLATQQRLEASQMDQGTDQP

MQESPCNEEKIGAGD

>Anopheles_mer_CRY-2_XP_041778502.1 XP_041778502.1

MAKRETIVHWFRKGLRIHDNPALTVAVDKVRANPAKYCLRPIFVLDPGIRKWLRVGPNRWRFLQQTLANLDENLRSINSR

LYVVRGNPVEVFPKLFADWNVSLLTYEHDIEPYAVKRDSTVEEQARKHRVEVHIEKSHTIFDPEAIVKKNGGKPPLTYQR

YATLASACKIPQPLPVPQKLPAQATAPEEDEEERKNPSCYDPPTMEELEVEEASMGRCKFPGGESEALRRMNEILSRKAW

VCKFEKPNTSPNSLEPSTTVLSPYLKFGCLSVRLFYSRIAETIKGQKHSQPPVSLIGQIMWREFYYCVAAVTPNYDKMVG

NSICTQIDWDTNKDHLEAWTHGRTGYPFIDAIMRQLRQEGWIHHLARHAVACFLTRGDLWISWEEGQRVFEELLLDADWA

LNAGNWMWLSASAFFHQYFRVYSPVAFGKKTDPEGKFIKKYVPELARFPAGIIYEPWKANLETQKKLGCIIGKDYPNRIV

VHEDISKVNIQRMSAAYKRNKAQKDGGTEEDDSVSSGSGGTPGKKRPSSTGSTAKGKAAPKKRKIEATIEKFLKKK

>Anopheles_mer_PL_XP_041769754.1 XP_041769754.1

MFLVRASRFSIKCSLNHAPYRFLLLIGPTMEKSASSSSSSSSLEPAAKKQKPNDDPPKPSDKGSAVGREDYVAMLKADRK

ATAKSILDFDFNKKRVRILSDAKTIEDGKQGVLYWMSRDARVQDNWAFLFAQKLALKNDLPLHVCFNLVPKFLDATIRHF

KFMLKGLEEVAEECRKLNIQFHLLRGSAGENVPAFVKKHKIGGVVCDFSPLRVPMKWVDEVRKALPMEIPLCQVDAHNIV

PVWVTSDKQEYAARTIRAKVNKNLPTYLTPFPPLVKHPFTADFEADPISWTQVLDTLQVDRTVEAVEWATPGYAGGVKTL

QTFVEKRLGKFNDKRNDPTENALSNLSPWFHFGQIAVQRAVLTVKKHGKRYSESVASFCEEAIVRRELSDNFCFHNKNYD

NLQGAYDWARKTLDDHRKDKRVYCYSREELETAKTHDDLWNSAQLQMVKEGKMHGFLRMYWAKKILEWTKTPEEALETAI

YLNDRYSLDGRDPNGYVGCMWSIAGIHDQGWKEREIFGKIRYMNYDGCKRKFNVNAFVVRYGGKVHRRK

>Anopheles_sin_AGAP00_KFB40799.1 KFB40799.1

MTINNILWFRHGLRLHDNPSLLEALKNDCANHSSETVKLFPVFIFDGESAGTKIVGYNRMKFLLESLADLDRQFRELGGQ

LLVFRGDSVAVLRRLFEELNVKKLCFEQDCEPIWKDRDDRVAKLCQSMDVRCVEHVSHTLWNPLEVIQTNGDIPPLTYQM

FLHTVNIIGEPPRPVGAPNFEYIEFGRIPAILSSELKLFTKIPTPEDFGIRYDGLADIAYQKWIGGETRALSALGSRLKQ

EEEAFREGYYLPTQANPEILGPATSMSAALRFGCLSVRLFYWCVHDLFAKVQSDSQFKYPGGQHITGQLIWREYFYTMSV

QNPYYGEMERNPICLNIPWYEPVDDSLARWKEGRTGFPMIDAAMRQLLAEGWLHHILRNITATFLTRGGLWISWEAGLQH

FLKYLLDADWSVCAGNWMWVSSSAFERLLDSSKCTDPISLGRRLDPKGSYVKRYLPELASYPPELIHEPWKATREQQIEY

GCVIGEHYPAPMVDLAVVGKRNAQTMASLRENLVADGSSTPPHCRPSDIDEIRQFFWLSDDVVPEA

>Anopheles_sin_AGAP00_KFB40969.1 KFB40969.1

MAKKETIVHWFRKGLRTHDNPALSVAVDRVRENPNKYFLRPIFVLDPGIRKWLRVGPNRWRFLQQTLANLDENLRLINSR

LYVVRGNPIEVFPKLFEEWNVSLLTYEYDIEPYAVKRDKTVEEQATKHKVEVHVAKSHTIFDPELILKRNGGKPPLTYQK

YASVSSVFKIPQALPVPEKLPANCVHPEADKQERKNPSCYDVPTLDELGIAGNELGECKFPGGETEALRRMEKCLARKAW

ICSFEKPNTSPNSLEPSTTVLSPYLKFGCLSARLFYNRISETIKGQKHSQPPVSLIGQLMWREFYYCAAAGTPNFDKMVG

NNVCLQVDWDTNREFLDAWTHGRTGYPFIDACMRQLRQEGWIHHLARHAVACFLTRGDLWISWEEGQRVFEELLLDADWA

LNAGNWMWLSASAFFHQYFRVYSPVAFGKKTDPEGKFIRKYVPEVARFPAGIIYEPWKASIETQKKLGCIIGKDYPNRIV

VHEEISKVNIQRMSAAYKRNKGLKDEVTGDDSSAAQSKPTNKRGSTASSSSKSSPKAKPAPKRQKLENTIKKFLSKK

>Anopheles_sin_AGAP00_KFB51941.1 KFB51941.1

MTKKMSAIGHYKSSSSGTVGAAGMSGFSGGGSSEREQNASGSSSQLKMQQDMCGGGKGPSKDQNHHHHSHHNHYHSQQHR

QQLTSKPRDKHTVHWFRKGLRLHDSPALREGLRGATTFRCVFIIDPWFAGSSNVGINKWRFLLQCLDDLDRNLRKLNSRL

FVIRGQPADALPKLFKEWDTTCLTFEEDPEPFGRVRDHNISEMCKELGIEVISAASHTLYNLERIIEKNRGRAPLTYHQF

QAIIASMDAPPPPEAAITLEVIGNATTPQYDDHDDKYGVPTLEELGFETEALRPPVWIGGETEALARLERHLERKAWVAS

FGRPKMTPQSLLASQTGLSPYLRFGCLSTRLFYYQLTDLYKKIKKACPPLSLHGQLLWREFFYCAATKNPTFDKMVGNPI

CVQIPWDRNAEALAKWASGQTGFPWIDAIMTQLREEGWIHHLARHAVACFLTRGDLWISWEEGMKVFEELLLDADWSVNA

GMWMWLSCSSFFQQFFHCYCPVKFGRKADPNGDYIRRYLPVLKNFPTRFIHEPWNASESVQRAAKCLVGKDYPLPMVNHA

IASRANMERIKQVYQHLAKYRTPTGPTSYEGAEGAERGGSAIAGVMTAAKVHHMNTSSMNDSPSPTTILTSVNSSGNYMC

RSNPSAQSDPNVNYLSLHGGPSPGGVAHTKPGGDTGEGGTGGGGGSEKRVTDGMENHNHLAHSMQKNARPHQTSEHHFEE

PSVAEYAAIKPDSLSTLIRAGSLGNAAGRYSNLQDQLSNSLMSLECDVLAAKLKPNNYEYERNHNMYNNQFKVEYSDNFN

SGYGLRNAVFYGGKREDENDEGQHLDTVTDSVNNNNNNNSNNHSQNSAEERNVGDEQELGQARSESVGTRLIVAMSTLQD

PTAAGHPARAQVKQEPPVQTQADKSYHRQRQEALQRQQQPETRNQSQPMPVKNEQSVPQLQASPAMHTDCDYEPERHNLL

ADMQSQQQQQHHQRKPMMQTQAEPTLMEQGNDQPMPDALASTDKRGAAD

>Anopheles_sin_hypoth_KFB48037.1 KFB48037.1

MKKSASASSSSIGPAAKKQKPNEPEPPVEAASASKTNDNYVALFKADRKATAKSVLDFDFKKKRVRVLSDAKEVEEGKKG

VVYWMSRDVRVQDNWAFLFAQKLALKNELPLHVCFNLVPKFLDATIRHFKFMLKGLEEVAQECGQLNIQFHLLRGNAAQN

VPEFVQQHNIGAVVCDFSPLRVPAKWVEDVRKALPLEVPLCQVDAHNIVPVWVTSDKLEYAARTIRNKVNNNLSTYLTPF

PPVVKHPYTASSQANPIDWAKVLDTLEVDRSVDEVTWATPGYEGGVRTLQSFVEKRLVKFNSKRNDPTQNALSNLSPWFH

FGQLSVQRAILTVKSYGKRFNESVASFCEEAIVRRELSDNFCFYNKNYDNLQGAYDWARKTLDDHRKDKRVYCYSRDELE

TAKTHDDLWNAAQLQMVKEGKMHGFLRMYWAKKILEWSKTPEEALEAAIYLNDRYSLDGRDPNGYVGCMWSIAGIHDQGW

KERDIFGKIRYMNYEGCKRKFDVNAFVIRYGGKVHRRK

>Anopheles_ste_CRY-1_XP_035899755.1 XP_035899755.1

MTINNILWFRHGLRLHDNPSLLEALRSDCMSQSSEAVKLFPIFIFDGESAGTRIVGYNRMKFLLESLADLDRQFRDLGGQ

LLVFRGDSVTVLRRLFEELNIKKLCFEQDCEPIWKERDDGVSQLCQKMDVRCVESVSHTLWNPNEVIQTNGDIPPLTYQM

FLHTVNIIGEPPRPVGAPNFEYIEFGRIPAILGSELKLCHQIPAPEDFGIYYEGNAKIAFQKWIGGETRALEALGARLKQ

EEEAFREGYYLPTQAKPEILGPATSMSAALRFGCLSVRMFYWCVHDLFERVQSHSQFKYPGGHHITGQLIWREYFYTMSV

QNPYYGEMERNPICLNIPWYAPEDDSLARWKEGRTGFPMIDAAMRQLLAEGWLHHILRNITATFLTRGGLWLSWEAGLQH

FLKYLLDADWSVCAGNWMWVSSSAFERLLDSSKCTCPIALARRLDPKGDYVKRYLPELANYPVQFVHEPWKASREQQIEY

GCVIGQHYPAPMIDLAIVSKRNAHTMATLRDKLVDGGASTPPHCRPSDIDEIRQFFWLADDAVTEA

>Anopheles_ste_CRY-1_XP_035909958.1 XP_035909958.1

MAKRETIVHWFRKGLRIHDNPALTVAVDKVRENPAKYCLRPIFMLDPGIRKWLRVGSNRWRFLQQTLVDLDKNLRSINSR

LYVVRGNPVEVFPKLFTEWNVSLLTYEHDIEPYAVKRDKTVEDQAKKHRVEIHVEKSHTIFDPEAIVKKNAGKPPLTYQR

YATLASACKIPPVLPAPSKLPAKCAAPESDKEERKNPACYDPPTMKELGVDEGTMSECKFPGGETESLRRMEEVLGRKAW

VCNFEKPNTSPNSLEPSTTVLSPYLKFGCLSVRLFYSRISDTIKGQKHSQPPVSLIGQIMWREFYYCVAAVTPNFDKMVG

NGVCMQIDWDTNKDHLEAWTHGRTGYPFIDAIMRQLRQEGWIHHLARHAVACFLTRGDLWISWEEGQRVFEELLLDADWA

LNAGNWMWLSASAFFHQFFRVYSPVAFGKKTDPEGKFIRKYVPELARFPAGIIYEPWKANLETQKKLGCIIGKDYPKRIV

VHEDISKVNIQRMSAAYKRNKAQKETDLDGDSAPDASTPGKKRPASSSSSAKSSAAPKKRKLEATIEKFLKKK

>Anopheles_ste_CRY-1-_XP_035903937.1 XP_035903937.1

MTKKTSSIGHFAASTSGAGAGGMNHFGTGTCGTDSSDKQQLSSSGSNQQQQHHDGGKSSSQDRQHHHQHHHHQHHLQQQQ

QQQQQSGGKLRDKHTVHWFRKGLRLHDNPALREGLRGTKTFRCVFIIDPWFAGSSNVGINKWRFLLQCLDDLDRNLRKLN

SRLFVIRGQPADALPKLFKEWGTTCLTFEEDPEPFGRVRDHNISEMCKELGIEVISAASHTLYNLERIIEKNGGRAPLTY

HQFQAIIASMDAPPQPEASITLDVIGKATTPQYDDHDDKYGVPTLEELGFETEALRPPVWIGGETEALARLERHLERKAW

VASFGRPKMTPQSLLASQTGLSPYLRFGCLSTRLFYYQLTDLYKKIKKACPPLSLHGQLLWREFFYCAATKNPTFDKMAG

NPICVQIPWDRNAEALAKWASGQTGFPWIDAIMTQLREEGWIHHLARHAVACFLTRGDLWISWEEGMKVFEELLLDADWS

VNAGMWMWLSCSSFFQQFFHCYCPVKFGRKADPNGDYIRRYLPVLKNFPTRFIHEPWNASESVQRAAKCLIGKDYPLPMV

NHAIASRANMERIKQVYQHLAKYRTPGGGCYEADCNEKGGSAIAGVMTAAKVQHMNTSSMNDSPSPTTILTSVNSSGNYM

CRSNPPAQSDPNGKIITYHQMLGGPEPRSTLGGNGSGERGGTGASEGNKHGDHGTVGSNHMMSGKDSGVSQHQQQHHQTQ

HSHQAQNLDQQSQENSNSVANSEQQRFGSMADYAAIKPDSLSTLIRAGNLSTVGARFSNLQDQLSNSLMSLECDVMATKL

KPNNYEYERNQNIYNSQFKVEYSDNFNSGYGLRNAVFYGGKHEDENEESLKADSINNNNHSDQLGGNNVDGEGQAVHNEA

DEDDGDESPMSTLEDTGTNQAPHALLFGTNKSKQIKQESGLVSQSTRMFLEPPKHYPGRGSKVHRARCDDQPEQQSHALH

SSSLDAQIKKEQQITSQQLPPHSMHTDCDYEPESHKLMANIHRQQQQQQQQQQQQHDQQQQQQQQQASMMAAQRLEASQM

DQGTDQPMQESPCSDEKIGAAD

>Anopheles_ste_PL_X2_XP_035906634.1 XP_035906634.1

MKKSASSSSSSSEPAAKKHKPNDGPPAPAVDKVDGCPEDYVALFKADRKATAKSILDFDFSKKRVRILSDAKSIEDGKRG

VLYWMSRDVRVQDNWAFLFAQKLALKNDLPLHVCFNLVPKFLDATIRHFKFMLKGLEEVADECRKLNIPFHLLRGNAGDN

VPEFVRKHKIGGVVCDFSPLRVPMGWVDEVKQALPMEVPLCQVDAHNIVPVWVTSDKLEYAARTIRTKVNKNLPTYLTPF

PPLVKHPFAATFEADPINWTKVLDTLQVDRSVDAVEWATPGYVGGVKTLQSFVEKRLGKFNDKRNDPTENALSNLSPWFH

FGQLSVQRAILTVKKYGKRYSESVASFCEEAIVRRELSDNFCFHNKNYDNLKGAYDWARKTLDDHRKDKRVYCYSRGELE

AAKTHDDLWNAAQLQMVKEGKMHGFLRMYWAKKILEWTKSPEEALETAIYLNDRYSLDGRDPNGYVGCMWSIAGIHDQGW

KERAIFGKIRYMNYEGCKRKFNVNAFVVRHGGKAHHRK

>Anoplophora_gla_CRY-1-_XP_018561357.1 XP_018561357.1

MSGSAYLTSNGQEKHTVHWFRKGLRLHDNPSLKEGLKGASTFRCVFVLDPWFAGASNVGINKWRFLLQCLEDLDRNLRKL

NSRLFVIRGQPADALPKLFKEWGTTALTFEEDPEPFGRVRDDNITALCKELGISVIQKVSHTLYHLQHIIDRNAGQAPLT

YHQFLAVVACMGPPPKPELPVNASSLNGAVTPLSEDHDEKYGVPTLEELGFDTDGLNPPVWQGGETESLARLERHLERKA

WVASFGRPKMTPQSLLPSQTGLSPYLRFGCLSTRLFYYQLTDLYKKIKKAFPPLSLHGQLLWREFFYCAATKNPNFDKML

GNPICVQIPWDKNLEALAKWANGQTGFPWIDAIMTQLRQEGWIHHLARHAVACFLTRGDLWISWEEGMKVFEELLLDADW

SVNAGMWMWLSCSSFFQQFFHCYCPVKFGRKADPNGDYIRKYLPVLKNMPIQYIHEPWAAPENVQRAAKCMIGKDYPLPM

VNHASASRINIQRMKQVYHQLANYRPLEHCKCNGPENHKDGFQHQANAVTVGNHNSNNQSLN

>Antheraea_per_CRY_2_ABO38435.1 ABO38435.1

MSAAAETLSAPRARSHVPAASLTSAPTPRRPHTKHTVHWFRKGLRLHDNPALREGLVNATTFRCVFIIDPWFASSSNVGI

NKWRFLLQCLEDLDSSLKKLNSRLFVVRGQPADALPKLFREWGTTALTFEEDPEPYGRVRDHNITTKCREVGINVISRVS

HTLYKLDKIIERNGGKAPLTYHQFQALIASMPPPQPAEAPISIETLNGAKTPVSVDHDDRFGVPTLEELGFETEDLKPPM

WMGGESEALARLDRHLERKAWVASFGRPKMTPQSLLASQTGLSPYLRFGCLSTRLFYYQLTELYKKIKRVRPPLSLHGQI

LWREFFYCAATRNPNFDRMEGNPICVQIPWEKNQEALSKWANGQTGYPWIDAIMIQLREEGWIHHLARHAVACFLTRGDL

WISWEEGMKVFDELMLDADWSVNAGMWMWLSCSSFFQQFFHCYCPVRFGRKTDPNGDFIRKYIPALKNMPTRYIHEPWVA

PESVQQAAQCVVGCDYPLPMLDHTKASQINLERIKQVYAQLAKYKPQVTLQTVQRPNVMQSSPSPTSIIASINQSNLLCS

TTPESQSAATQMIYKDPSGIFQRPTKNAVHADTKNTKFKKVLIVHQVKHSEITEERVSQTNSHHQTPKENYIINSRQSIE

YKTCSDNNLQPPNKHNNYDFKNLVINNHIQGYNNHIIYQCQKGNQNEIYSQQGVKTNAYGYENTKFFLSNFADNNIFVSG

SPQLYIPGQVNEESPLQSNSETNNESTKEKTVDLHCINIQSEESFSMENIQNSTTAESAVNPTNENEN

>Antheraea_per_CRY_AAK11644.1 AAK11644.1

MLGGSVLWFRHGLRLHDNPSLHAALEDRSVPFFPIFVFDGETAGTKLVGYNRMRYLLEALEDLDNQFKKYGGKLIMLKGK

PSDVFRRLWEEFGIRKLCFEQDCEPLWRARDDGVKSACREIGVACREYVSHTLWEPDTVIRANGGIPPLTYQMFLHTVTI

VGDPPRPVPDVDMSGITFGTLPDCFYQEFTVFDKTPKPEDLGVFLENEDIRMIRWVGGETAALKQMQQRLAVEHETFRKG

SYLPTHGSPDLLGPPISLSPALRFGCLSVRSFYWSVQDLFRQVHQGRLSSAHFITGQLIWREYFYTMSVNNPNYGQMADN

PICLDIPWKHPEGDELQRWIEGRTGFPFIDAAMRQLRAEGWLHHAVRNTVASFLTRGTLWLSWEHGLNHFLKYLLDADWS

VCAGNWMWVSSSAFEALLDSGECACPVRLGQRLDPSGEYVRRYVPEIARMPVDYIYEPWKAPLDVQTRASCIIGKHYPAP

LVNHIVAAQRNRNAMKELRQILQKAPPHCCPSSEEEIRQFMWVTE

>Anthonomus_gra_gra_CRY-1-_XP_050306949.1 XP_050306949.1

MSCAELDNAARREGKHAVHWFRKGLRLHDNPSLREGLQGATTFRCVFVLDPWFAGSSNVGINKWRFLLQCLEDLDRSLRK

LNSRLFVIRGQPADTLPKLFREWGTTALTFEEDPEPFGRVRDHNITALCKELGITVVQNVSHTLYQLQNIIDRNGGKAPL

TYHQFLAVIACMGPPPQPEPPVTGSTVELAHTPLAEDHDEKFGVPTLEELGFDTEGLNPPVWQGGETEALARLERHLERK

AWVASFGRPKMTPQSLLPSQTGLSPYLRFGCLSTRLFYYQLTDLYKKIKKAFPPLSLHGQLLWREFFYCAATKNPNFDKM

LGNPICVQIPWDRNAEALAKWANGQTGFPWIDAIMTQLRQEGWIHHLARHAVACFLTRGDLWISWEEGMKVFEELLLDAD

WSVNAGMWMWLSCSSFFQQFFHCYCPVKFGRKADPNGDYIRKYLPILKNMPTQYIHEPWMSPENVQKAAKCIVGTDYPLP

MVNHISVSKINIQRMKQVYQQLSNYRQHEANYYSNNLKRYDQGPYRDNYHKGILTVNYNNNSRNAQ

>Anthonomus_gra_gra_PL_XP_050295997.1 XP_050295997.1

MASLKPRDAKGKISLENFTVENFLGAIENSRQSQGTSIENYDFNKSRCRILSANDQISEGSKGVLYWMTRDCRIHDNWGI

LFSQRLALKNKLPLLICFSLFEAHHYYPTRRHQDFLIKGLELVKNDCEALNINFYLINQSPRELVKSVVKNKLGGVVCDF

SPLKKRREWIDTLCKALPDDIPIVQVDAHNIVPAWIASEKQEIMAKTLRPKINKKLSEYLTGFPAVIKHPYKGIFNIETI

SFDKAFSVLKTTHDVGPVEEAGAKRGLEMLFKFMQKGLKHYGITSNDPSKEHTSNLSFWINFGHISAQRVALEVKSLESL

YKEQVDKYLEELIVRRELAENYCFYNENYDNLMGAADWARKSLELHAGDKRSYLYSKEEFEKGLTHDDCWNAAQFQLKKE

GKIHGYMRMYWCKKILEWSSGPKEALDTALWLNDTFALDGNDPNGFVGVMWSICGVHDQGWREREVFGKIRFMVEYSLRR

KFDMEAYCARYGVVGKKEQKKGGSKRKNSN

>Aphidius_gif_CRY-1_XP_044018980.1 XP_044018980.1

MTGSRHHGCDQGVSSQVDNVPKAKHSVHWFRKGLRLHDNPSLRDCLADACTFRCVFVIDPWLAGSKSVGVNKWRFLLQCL

EDLDVSLRKLNSRLFVIRGQPTDALPKLFREWGTTNLTFEEDPEPFGRARDHNITTLCKEIGISVVQVASHTLYKMDEIL

ERNGGKSPLTYHQFQSIIAGMDSPLPPVPTITADCIGAAYTPLQADHDEKYGVPTLDELGFDIEKLKPPVWIGGESEALA

RLERHLERKAWVASFERPKMTPQSLLPSQTSLSPYLRFGCLSTRLFYYKLCELYKQIKKAMPPLSLHGQLLWREFFYCAA

TKNQNFDRMKGNPICLQIPWDTNVEALSKWANGTTGFPWIDAIMTQLREEGWIHHLARHAVACFLTRGDLWISWEEGMKV

FDEFLLDADWSINAGMWMWLSCSSFFQQFFHCYCPVKFGRKADPNGEYIRRYLPVLKNYPTRYIHAPWMAPLSIQRAAKC

IIGHDYSMPMVNHSKNSRINMERMKQVYQRFNKYHNNDEIKLLII

>Aphidius_gif_PL-lik_XP_044004454.1 XP_044004454.1

MSRKNSSEPPIKKQKLTSLFDELTEKRKNVADSITNFKFNKKRVKILSQATEVVKGSKGILYWMFRDGRIQDNWAMLFAQ

KLALKNRIPLHVCYCILPKFLDATLRHYKFLVESLEEVSMDAKKLNINFHLLHGEPNTVVLEFIRKHNMGALITDFFPLR

VPMAWVESLKMDLPQDIPICQVDAHNIVPCWVTSDQLEYSARTIRNKINSKLQEYLTEYPPVIEHPYKSSFEVPEIDWLN

CLNDVEIDRSVDKVEWCKPGYRAALIQLEKFIYECLPHYCEKRNNPHLEMTSGLSPWFHFGMISVQRVILEVQKYKQKYL

RSVNDFMEEAIVRRELSDNFCLHNKNYDKIDGARQWAIETLNTHKKDKRQWIYDLKELETSQTHDDLWNAAQNQLTVDGK

IHGFMRMYWAKKILEWTKLPEDALAWSIYLNDKYSMDGRDPNGYVGCMWSICGIHDQGWKEREIFGKIRYMNYKGCERKF

DVKAYVQKHNGKVINIKALSQKKTTKKTK

>Aphis_cra_CRY-1_KAF0762916.1 KAF0762916.1

MIGRKTVIMTIAVHWFRNGLRLHDNPALVEAQNNADNLITLFIFDETTFNTKWYGYNRLRFLLESLKDLNNNLTLVGGHL

YILQGSPVKIFQMIKEKIGLDFITFEQDCDHIGKSRDDKVKMFCNENEIKYIEKVSHTLWNPKLVIEKNGGIAPFTYKQF

QNTVNKIGQPPKPVGNIDWLSTIFEELPTSILDEFKVLNNPTPESFGLYPEFPENLISSHRWYGGETRALEQLNERLEYE

KEAFVNGFYLPNQVNPDLLSPSSSLSAALRYGCLSIRKFYWELSKLFIKQFEGDLLPQYSVTSQLIWRDYFYTMSIENKN

FGQMEDNPACISIPWNDIKIPENKKMLECWKTGKTGYPFIDAGMRQLMQEGWVHHVVRNSLACFLTRGDLWISWVEGLNH

FMKYLLDADFSVCSGNWIWVSSSTFEQLLDCPLCVCPVNYGLRLDPSGEYIKRYIPELKNMPIEFLYEPWKCPESVQKQV

GCIIGKDYPHCIVNHTKVSRGNRKKMLALRASMTNEHMVPHCCPSDREEAQKFMFLPDECMQQLLPLDNDDSEMYDFYKL

KLI

>Aphis_cra_CRY-1-_KAF0757513.1 KAF0757513.1

MDYKVQNKHTVHWFRKGLRLHDNPSLREGLINAKTFRCIFILDPWFAGASNVGINKWRFLLQCLVDLDNSLKKLNSRLFV

IKGQPAEALPKLFRLWGTTNFTFEEDPEPFGRVRDQNIKVMCSEMGISVITRCSHTLYQLDKIINVNGGKAPLTYHLFQT

LLECIDPPERAVPSIDKEFLGNAFTPTKYDHDEIFGVPTLEELGFKEINNPARQVWIGGETEALIRLQCHLERKAFIASY

GKPKMTSQSLIASPTGLAPYLKFGCLSTRLFFSELNELYKKIRKSQPPLSLHGQLLWRDFFYCASTNNPNFDRMVGNPIC

VQIPWDKNPRALSKWANGQTGYPWIDAIMIQLRQEGWIHCIARHAVACFLTRGDLWLSWEEGMKVFDELLLDADWSVNAG

YWMWYSCSSFYQEFIHCYCPVRFGRKVDPNGDYIRRYIPALNNMPNQYIHEPWLAPESIQFSANCIIGIDYPLPIVNHVN

ASKINLERMKLAYQQLSNCQPQLENGKLILISSLRR

>Aphis_cra_Unchar_KAF0772362.1 KAF0772362.1

MDKNNADVGHETTVHWFRKGMRLHDNPAFRLSCEARNSKGECYKLRPIYILDPYFRKYIRAGANRWRFLQQSLVDLDTTL

RKLGTRLYVIRGLPHEVFPDLFAKWNVKLLTFELDTEPYARERDNQVEHLARKHGVKVEQKVSHTIYNTELVLRANGGSV

PMTYQKFISVVESMPNPRRPIPAPDTLPPGCLINDDLNNQEFDVPTLDELLTLKGFNPAELKPCLYPGGEKEALRRLEEY

MKNKTWVCKFEKPNTSPNSLKPSTTVLSPYMKFGCLSASHFYYRLKEVIGNSPHSKPPVSLIGQLYWREFYYTVGASTPN

FDKMEGNSICCQVPWDNNPDALEAWTNGKTGYPFIDAIMRQLRDEGWIHHLARHAVACFLTRGDLWISWEKGLAVFEELL

LDADWSMNAGNWMWLSASAFFHQFFRVYSPVAFGKKTDKSGDYIRKYIPELAKYPDQYIYEPWSAPKSIQERAGCVIGVH

YPKRVVVHEDVYKNNITKMSLAYKSTKAGKSSSNTKKSREMSSPDKKNIKKAKLK

>Aphis_gly_hypoth_KAE9526623.1 KAE9526623.1

MDFKVQNKHTVHWFRKGLRLHDNPSLREGLINAKTFRCIFILDPWFAGASNVGINKWRFLLQCLVDLDNSLKKLNSRLFV

IKGQPAEALPKLFRLWGTTNFTFEEDPEPFGRVRDQNIKVMCSEMGISVITRCSHTLYQLDKIINVNGGKAPLTYHLFQT

LLECIDPPERAVPSIDKEFLGNAFTPTKYDHDEIFGVPTLEELGFKEINNPARQVWIGGETEALIRLQCHLERKAFIASY

GKPKMTSQSLIASPTGLAPYLKFGCLSTRLFFSELNELYKKIRKSQPPLSLHGQLLWRDFFYCVSTNNPNFDRMVGNPIC

VQIPWDKNPRALSKWANGQTGYPWIDAIMIQLRQEGWIHCIARYAVACFLTRGDLWLSWEEGMKVFDELLLDADWSVNAG

YWMWYSCSSFYQEFIHCYCPVRFGRKVDPNGDYIRRYIPALNNMPNQYIHEPWLAPESIQFSANCIIGIDYPLPIVNHVN

ASKINLERMKLAYQQLSNCQPQLENGKLILISSLRR

>Aphis_gly_hypoth_KAE9537491.1 KAE9537491.1

MVLKKCGQPFLSRVTTIKSGNIKQFQTLNGRKTVIMTIAVHWFRNGLRLHDNPALIEAQNNADNLITLFIFDETTFNTKW

YGYNRLRFLLESLKDLNNNLTLVGGHLYILQGSPVKIFKMIKEKIGLDFITFEQDCDHIGKNRDDKVKMFCNENEIKYIE

KVSHTLWNPKLVIEKNGGIAPFTYKQFQNTVNKIGQPPKPVGNIDWLSTIFEELPTSILDEFKVLNNPTPESFGLYPEFP

ENLISSHRWYGGETRALEQLNERLEYEKEAFVNGFYLPNQVNPDLLSPSSSLSAALRYGCLSIRKFYWELSKLFIKQFEG

DLLPQYSVTSQLIWRDYFYTMSIENKNFGQMEDNPACISIPWNDIKIPENKKMLECWKTGKTGYPFIDAGMRQLMQEGWV

HHVVRNSLACFLTRGDLWISWVEGLNHFMKYLLDADFSVCSGNWIWVSSSTFEQLLDCPLCVCPVNYGLRLDPSGEYIKR

YIPELKNMPIEFLYEPWKCPESVQKQVGCIIGKDYPNCIVNHTKVSRGNRKKMLALRASMTNEHMVPHCCPSDREEAQKF

MFLPDECMQQLLPLDNDDSEIFKLLFWTNYHKNQN

>Aphis_gly_hypoth_KAE9544189.1 KAE9544189.1

MSGNPPPTKKIKKTSSEVSTSKTSNFLNDIEAERKKTASSIMEFKFNKKRVRVLSEQKEVPEWAEGVIYWTFRDERIHDN

WALLYAQKLAIKNKVSLHITFCRLTQFLNCSLRHYKHIFQGLEELETECKDLNIQFHFLIGCAADILPEFVKKHKLGAIV

VDFMPVREHMSWAKQLADRIGSEVPVIQVDAHNIVPCWVASDKQEYGARTIRNKINNKLPEFLTEFPPVIKHPFNSKFKA

QPTNWDEADKTLEVDRSVVSVPGLKAGFKAGMTELENFLKKRLPKYSTDRNNPVKDGLSKLSPWLHFGQISAQRCILEVS

KLSKQYPESVAAYREEAIVRRELSDNFCFYNPKYDKIDGAPNWAQTTLNDHRKDKRMYVYTREELEGSRTHDDLWNSAQI

QLVKEGKMHGFLRMYWAKKILEWTDTPERALADAIYLNDKYSMDGRDPSGFVGCMWSICGIHDQGWRERDIFGKIRYMNY

AGCKRKFDINAFIVRYGGMVHKYTKK

>Aphis_gly_hypoth_KAE9545551.1 KAE9545551.1

MDKENADVEHETTVHWFRKGMRLHDNPAFRLSCEARNSKGECYKLRPIYILDPYFRKYIRAGANRWRFLQQSLVDLDTTL

RKLGTRLYVIKGLPHEVFPDLFAKWNVKLLTFELDTEPYARERDNQVEQLARKHGVKVEQKVSHTIYNTELVLRANGGSV

PMTYQKFISVVESMPNPRRPIPAPDTLPSECLLNDDLNNQEFDVPTLNELLTLKGFNPAELKPCLYPGGEKEALRRLEEY

MKNKTWVCKFEKPNTSPNSLKPSTTVLSPYMKFGCLSANHFYYRLKEVIGNSPHSKPPVSLIGQLYWREFYYTVGASTPN

FDKMEGNPICCQVPWDNNPDALEAWTNGKTGYPFIDAIMRQLRDEGWIHHLARHAVACFLTRGDLWISWEKGLAVFEELL

LDADWSMNAGNWMWLSASAFFHQFFRVYSPVAFGKKTDKSGDYIRKYIPELAKYPDQYIYEPWSAPKSIQERAGCVIGVH

YPKRVVIHEDVYKNNITKMSLAYKSTKAGKSSSNTKKSREMSSPDKKNIKKAKLK

>Aphis_gos_CRY-1_XP_027850736.2 XP_027850736.2

MDKKNADVGHETTVHWFRKGMRLHDNPAFRLSCEARNSKGECFKLRPIYILDPYFRKYIRAGANRWRFLQQSLVDLDTTL

RKLGTRLYVIRGLPHEVFPDLFAKWNVKLLTFELDTEPYARERDNQVEQLARKHGVKVEQKVSHTIYNTELVLRANGGSV

PMTYQKFISVVESMPNPRRPIPAPDTLPPECLLNDDLNNQEFDVPTLNELLTLKGFNPAELKPCLYPGGEKEALRRLEEY

MKNKTWVCKFEKPNTSPNSLKPSTTVLSPYMKFGCLSASHFYYRLKEVIGNSPHSKPPVSLIGQLYWREFYYTVGASTPN

FDKMEGNPICCQVPWDNNPDALEAWTNGKTGYPFIDAIMRQLRDEGWIHHLARHAVACFLTRGDLWISWEKGLAVFEELL

LDADWSMNAGNWMWLSASAFFHQFFRVYSPVAFGKKTDKSGDYIRKYIPELAKYPDQYIYEPWSAPKSIQERAGCVIGVH

YPKRVVVHEDVYKNNITKMSLAYKSTKAGKSSSNTKKSREMSSPDKKNIKKAKLK

>Aphis_gos_CRY-1__XP_027844197.2 XP_027844197.2

MTIAVHWFRNGLRLHDNPALIEAQNNADNLITLFIFDETTFNTKWYGYNRLRFLLESLKDLNNNLTLVGGHLYILQGSPV

KIFKMIKEKIGLDFITFEQDCDHIGKNRDDKVKMFCNENEIKYIEKVSHTLWNPKLVIEKNGGIAPFTYKQFQNTVNKIG

QPPKPVGNIDWLSTIFEELPTSILDEFKVLNNPTPESFGLYPEFPENLISSHRWYGGETRALEQLNERLEYEKEAFVNGF

YLPNQVNPDLLSPSSSLSAALRYGCLSIRKFYWELSKLFIKQFEGDLLPQYSVTSQLIWRDYFYTMSIENKNFGQMEDNP

ACISIPWNDIKIPENKKMLECWKTGKTGYPFIDAGMRQLMQEGWVHHVVRNSLACFLTRGDLWISWVEGLNHFMKYLLDA

DFSVCSGNWIWVSSSTFEQLLDCPLCVCPVNYGLRLDPSGEYIKRYIPELKNMPIEFLYEPWKCPESVQKQVGCIIGKDY

PNCIVNHTKVSRGNRKKMLALRASMTNEHMVPHCCPSDREEAQKFMFLPDECMQQLLPLDNDDSEILKLLFWTNYHKNQN

>Aphis_gos_CRY-1-_XP_027838715.1 XP_027838715.1

MDFKVQNKHTVHWFRKGLRLHDNPSLREGLINAKTFRCIFILDPWFAGASNVGINKWRFLLQCLVDLDNSLKKLNSRLFV

IKGQPAEALPKLFRLWGTTNFTFEEDPEPFGRVRDQNIKVMCSEMGISVITRCSHTLYQLDKIINVNGGKAPLTYHLFQT

LLECIDPPERAVPSIDKEFLGNAFTPTKYDHDEIFGVPTLEELGFKEINNPARQVWIGGETEALIRLQCHLERKAFIASY

GKPKMTSQSLIASPTGLAPYLKFGCLSTRLFFSELNELYKKIRKSQPPLSLHGQLLWRDFFYCASTNNPNFDRMVGNPIC

VQIPWDKNPRALSKWANGQTGYPWIDAIMIQLRQEGWIHCIARHAVACFLTRGDLWLSWEEGMKVFDELLLDADWSVNAG

YWMWYSCSSFYQEFIHCYCPVRFGRKVDPNGDYIRRYIPALNNMPNQYIHEPWLAPESIQFSANCIIGIDYPLPIVNHVN

ASKINLERMKLAYQQLSNCQPQLENGKLILISSLRR

>Aphis_gos_PL_XP_050056746.1 XP_050056746.1

MSSNSPPTKKFKKTSSEVGTSKTSNFLNDIEAERKKTASSIMEFKFNKKRVRVLSEQKEVPEWAEGVIYWTFRDERIHDN

WALLYAQKLAIKNKVSLHITFCRLTQFLNCSLRHYKHIFQGLEELETECKDLNIQFHFLIGCAADILPEFVKKHKLGAIV

VDFMPVREHMSWAKQLADRIGSEVPVIQVDAHNIVPCWVASDKQEYGARTIRNKINNKLPEFLTEFPPVIKHPFNSKFKA

QPTNWDEADKTLEVDRSVVSVPGLKAGFKAGMAELENFLKKRLPKYSTDRNNPVKDGLSKLSPWLHFGQISAQRCILEVS

KLSKQYPESVAAYREEAIVRRELSDNFCFYNPKYDKIDGAPNWAQTTLNDHRKDKRMYVYTREELESSRTHDDLWNSAQI

QLVKEGKMHGFLRMYWAKKILEWTDTPERALADAIYLNDKYSMDGRDPSGFVGCMWSICGIHDQGWRERDIFGKIRYMNY

AGCKRKFDINAFIARYGGMVHKYTKK

>Apis_cer_CRY-1__XP_016920577.1 XP_016920577.1

MTGSRSSEINPKEGLYDEGGKHTVHWFRKGLRLHDNPSLREGLAGASTFRCVFVLDPWFAGSTNIGINKWRFLLQCLEDL

DCSLRKLNSRLFVIRGQPADALPKLFKEWGTTNLTFEEDPEPFGRVRDHNISALCKELGISVVQKVSHTLYKLDEIIERN

GDKPPLTYHQFQTVVASMDPPEPPVPTVTSACVGSAYTPLKEDHDDHYGVPTLEELGFDTEGLLPPVWVGGESEALARLE

RHLERKAWVASFGRPKMTPQSLLPSQTGLSPYLRFGCLSTRLFYYQLTDLYKKIKKAVPPLSLHGQLLWREFFYCAATKN

PNFDRMQGNPICVQIPWDKNVEALAKWANGQTGFPWIDAIMTQLREEGWIHHLARHAVACFLTRGDLWISWEEGMKVFDE

LLLDADWSVNAGMWMWLSCSSFFQQFFHCYCPVRFGRKADPNGDYIRRYLPVLKNFPTRYIHEPWNAPLNVQRAAKCIIG

KDYSLPMVNHSKSSRINIERMKQVYQQLNKYRGNGVSLKGETVAFRQGLLNALPPPSMKETEEEKKKTKQSLSPSENQSK

MEILPKTTQRQHHH

>Apis_cer_PL_X1_XP_016904789.1 XP_016904789.1

MEELNPFKRRKVFDLLKKFEDNRKNTSESIMTFKFNKKRIKRLSNLNDIKENCDGILYWMFRDIRIQDNWALLFAQKIAL

KNNVPLHICFCIMSNFLNASIRYYKFLLKGLEEIEKECKKLNINFHLLHGEPNINILKFIKMYNIGAIITDFYPLKLPML

WIDNVKKNLPKDVPICQVDAHNIVPCWYASSKQEFSAKTIRNKINTKLDEFLTEFPPVIKHPYITKEMFEKNNWKIALEN

VEVDKSVKEITWAKPGYKNGIKELENFLQNRLKRYGDEHNNPLSNAISNLSPWFHFGMISVQRCILEIKEYKKLYKKSVE

SFMEEAIIRKELSDNFCFYNKKYDLIEGAYPWAIETLNKHRKDKRKYIYFLNHLENSETHDDLWNACQNQMVTIGKMHGF

LRMYWAKKILEWTETPEIALKWANYLNNKYSIDGCDPNGYVGCMWSICGVHDHGWPERDIFGKIRYMNYEGCKRKFNIAE

FVMKWGGKKANNDYIKI

>Apis_dor_CRY-1__XP_006620497.1 XP_006620497.1

MTGSRSSEINPKEGLYDEGGKHTVHWFRKGLRLHDNPSLREGLAGASTFRCVFVLDPWFAGSTNIGINKWRFLLQCLEDL

DCSLRKLNSRLFVIRGQPADALPKLFKEWGTTNLTFEEDPEPFGRVRDHNISALCKELGISVVQKVSHTLYKLDEIIERN

GDKPPLTYHQFQTVVASMDPPEPPVPTVTSACVGSAYTPLKEDHDDHYGVPTLEELGFDTEGLLPPVWVGGESEALARLE

RHLERKAWVASFGRPKMTPQSLLPSQTGLSPYLRFGCLSTRLFYYQLTDLYKKIKKAVPPLSLHGQLLWREFFYCAATKN

PNFDRMQGNPICVQIPWDKNVEALAKWANGQTGFPWIDAIMTQLREEGWIHHLARHAVACFLTRGDLWISWEEGMKVFDE

LLLDADWSVNAGMWMWLSCSSFFQQFFHCYCPVRFGRKADPNGDYIRRYLPVLKNFPTRYIHEPWNAPLNVQRAAKCIIG

KDYSLPMVNHSKSSRINIERMKQVYQQLNKYRGNGVSLKGETVAFRQGLLNALPPPSMKETEEEKKKKQSLPPSENQSKM

EILPKTTQRQHHH

>Apis_dor_PL-lik_XP_006619211.1 XP_006619211.1

MEELNPLKRRKVLDLLKKFEDNRKNTSESIMTFKFNKKRIKRLSNLNDIKENCNGILYWMFRDIRIQDNWALLFAQKTAL

KNNVPLHICFCIMSNFLNASIRYYKFLLKGLEEIEKEYFYPLKLPMLWIDNVKKNLPKDVPICQVDAHNIVPCWYASSKQ

EFAAKTIRNKINTKLEEFLTEFPPVIKHPYITKEKFEKNNWKIALQNIEVDKSVKEITWAKPGYRNGIKELENFLQNRLE

KYGDERNNPLSNAISNLSPWFHFGMISVQRCILEIKEYKKLYKKSVESFMEEAIIRKELSDNFCFYNEKYDLIEGAYPWA

IETLNKHRKDKRKYIYFLNHLENSETHDDLWNACQNQMVTIGKMHGFLRMYWAKKILEWTETPEIALKWANYLNNKYSID

GCDPNGYVGCMWSICGVHDHGWPERDIFGKIRYMNYEGCKRKFNIAEFVMKWGKRKEN

>Apis_flo_CRY-1__XP_012340625.1 XP_012340625.1

MTGSRNNEINPKEGLYDEGGKHTVHWFRKGLRLHDNPSLREGLAGASTFRCVFVLDPWFAGSTNIGINKWRFLLQCLEDL

DCSLRKLNSRLFVIRGQPADALPKLFKEWGTTNLTFEEDPEPFGRVRDHNISALCKELGISVVQKVSHTLYKLDEIIERN

GDKSPLTYHQFQTVVASMDPPEPPVPTVTSACVGSAYTPLKEDHDDHYGVPTLEELGFDTEGLLPPVWVGGESEALARLE

RHLERKAWVASFGRPKMTPQSLLPSQTGLSPYLRFGCLSTRLFYYQLTDLYKKIKKAVPPLSLHGQLLWREFFYCAATKN

PNFDRMQGNPICVQIPWDKNVEALAKWANGQTGFPWIDAIMTQLREEGWIHHLARHAVACFLTRGDLWISWEEGMKVFDE

LLLDADWSVNAGMWMWLSCSSFFQQFFHCYCPVRFGRKADPNGDYIRRYLPVLKNFPTRYIHEPWNAPLNVQRAAKCIIG

KDYSLPMVNHSKSSRINIERMKQVYQQLNKYRGNGVSLKGETVAFRQGLLNALPPPSMKEIEEEKKKTKQSLSSSENQSK

MEILPKTTQQQHHHQ

>Apis_flo_PL-lik_XP_012348866.1 XP_012348866.1

MEESNPFKRRKVLDLLKKFEDNRKNTSESIMTFKFNKKRIKRLSNLNDIKENCNGILYWMFRDIRIQDNWALLFAQKAAL

KNNVPLHICFCIMSNFLNASIRYYKFLLKGLEEIETECKKLNINFHLLHGEPNISILKFIKMYNMGAIITDFYPLKLPML

WIDNVKKNLPEDVPICQVDAHNIVPCWYASSKQEFAAKTIRNKINTKLEEFLTEFPPVIKHPYTTKEKFEKNNWKIALQN

VEVDKSVKEITWAKPGYRNGIKELENFLQNRLKKYGDERNNPLSNAISNLSPWFHFGMISVQRCILEIKEYKKLYKKSVE

SFMEEAIIRKELSDNFCFYNEKYDLIEGAYPWAIETLNKHRKDKRKYIYFLNHLENSETHDDLWNACQNQMVTIGKMHGF

LRMYWAKKILEWTETPEIALEWANYLNNKYSIDGCDPNGYVGCMWSICGVHDHGWPERDIFGKIRYMNYEGCKRKFNIAE

FVIKWGKKKTNELT

>Apis_lab_cry-1_XP_043787135.1 XP_043787135.1

MTGSRSSEINPKEGLYDEGGKHTVHWFRKGLRLHDNPSLREGLAGASTFRCVFVLDPWFAGSTNIGINKWRFLLQCLEDL

DCSLRKLNSRLFVIRGQPADALPKLFKEWGTTNLTFEEDPEPFGRVRDHNISALCKELGISVVQKVSHTLYKLDEIIERN

GDKPPLTYHQFQTVVASMDPPEPPVPTVTSACVGSAYTPLKEDHDDHYGVPTLEELGFDTEGLLPPVWVGGESEALARLE

RHLERKAWVASFGRPKMTPQSLLPSQTGLSPYLRFGCLSTRLFYYQLTDLYKKIKKAVPPLSLHGQLLWREFFYCAATKN

PNFDRMQGNPICVQIPWDKNVEALAKWANGQTGFPWIDAIMTQLREEGWIHHLARHAVACFLTRGDLWISWEEGMKVFDE

LLLDADWSVNAGMWMWLSCSSFFQQFFHCYCPVRFGRKADPNGDYIRRYLPVLKNFPTRYIHEPWNAPLNVQRAAKCIIG

KDYSLPMVNHSKSSRINIERMKQVYQQLNKYRGNGVSLKGETVAFRQGLLNALPPPSMKETEEEKKKKQSLPPSENQSKM

EILPKTTQRQHHH

>Apis_lab_PL-lik_XP_043787122.1 XP_043787122.1

MEELNPFKRRKVLDLLKKFEDNRKNTSESIMTFKFNKKRIKRLSNLNDIKENCNGILYWMFRDIRIQDNWALLFAQKTAL

KNNVPLHICFCIMSNFLNASIRYYKFLLKGLEEIEKECKKLNINFHLLYGEPNISILKFIKIYNMGAVITDFYPLKLPML

WIDNVKKNLPKDVPICQVDAHNIVPCWYASSKQEFAAKTIRNKINTKLEEFLTEFPPVIKHPHITKEKFEKNNWKIALQN

VEVDKSVKEITWAKPGYRNGIKELENFLQNRLEKYGDERNNPLSNTISNLSPWFHFGMISVQRCILEIKEYKKLYKKSVE

SFMEEAIIRKELSDNFCFYNEKYDLIEGAYPWAIETLNKHRKDKRKYIYFLNHLENSETHDDLWNACQNQMVTIGKMHGF

LRMYWAKKILEWTETPEIALKWANYLNNKYSIDGCDPNGYVGCMWSICGVHDHGWPERDIFGKIRYMNYEGCKRKFNIAE

FVMKWGKRKEN

>Apis_mel_NP_001077099.1_cry_2 NP_001077099.1

MTGSRSSEINPKEGLYDEGGKHTVHWFRKGLRLHDNPSLREGLAGASTFRCVFVLDPWFAGSTNIGINKWRFLLQCLEDL

DCSLRKLNSRLFVIRGQPADALPKLFKEWGTTNLTFEEDPEPFGRVRDHNISALCKELGISVVQKVSHTLYKLDEIIERN

GDKPPLTYHQFQTVVASMDPPEPPVPTVTSACVGSAYTPLKEDHDDHYGVPTLEELGFDTEGLLPPVWVGGESEALARLE

RHLERKAWVASFGRPKMTPQSLLPSQTGLSPYLRFGCLSTRLFYYQLTDLYKKIKKAVPPLSLHGQLLWREFFYCAATKN

PNFDRMQGNPICVQIPWDKNVEALAKWANGQTGFPWIDAIMTQLREEGWIHHLARHAVACFLTRGDLWISWEEGMKVFDE

LLLDADWSVNAGMWMWLSCSSFFQQFFHCYCPVRFGRKADPNGDYIRRYLPVLKNFPTRYIHEPWNAPLNVQRAAKCIIG

KDYSLPMVNHSKSSRINIERMKQVYQQLNKYRGNGVSLKGETVGLLNALPPSSMKETEEEKKKTKQSLSPSENQSKMEIL

PKTTQRQHHH

>Apis_mel_XP_006564509.1_deoxyribod XP_006564509.1

MEELNPFKRRKVFDLLKKFEDNRKNTSESIMTFKFNKKRIKRLNNLNDIKENCNGILYWMFRDIRIQDNWALLFAQKAAL

KNNVPLHICFCLISNFLNASIRYYKFLLKGLEEIEKECKKLNINFHLLHGEPNINILKFIKIYNMGAIITDFYPLKLPML

WIDNVKKNLPKDIPICQVDAHNIVPCWYASSKQEFAAKTIRNKINTKLEEFLTEFPPVIKHPYTTKEKFEKNNWKIALQN

VEVDKSVKEITWAKPGYENGIKELENFLQNRLKKYGDERNNPLSNAISNLSPWFHFGMISVQRCILEIKEYKKLYKKSVE

SFMEEAIIRKELSDNFCFYNEKYDLIEGAYPWAIETLNKHRKDKRKYIYFLNHLENSETHDDLWNACQNQMVTIGKMHGF

LRMYWAKKILEWTETPEIALKWANYLNNKYSIDGCDPNGYVGCMWSICGVHDHGWPERDIFGKIRYMNYEGCKRKFNIAE

FVMKWGKKKTNELI

>Apolygus_luc_hypoth_KAF6198968.1 KAF6198968.1

MAGWGAPVSAGFHSRRNHCLSRNEAGEKLHGGPSVLEGAGRREGACARSGDAAGGAPGGGGGNSVTAGAAIGGESMDRER

FMEEEEVERTVIRSGGILRRYVDEVRKLPVEYVYEPWTAPIEVQEQAGCVIGRDYPERIVDHNQVSSENCMRMETIRRNL

ATGVPHCCPSSTEELMQFMWLPPELHNHISKMSVDGFE

>Apolygus_luc_hypoth_KAF6198973.1 KAF6198973.1

MFRDQGNRCPTVHWFRHGLRLHDNPAMLEAVRDSTEFFAVFIFDGQSAGTSHVGYNRMKFLLESLKDLDAQLKKHGGRLY

TFKGNPTNVFRRLWEELGIKKICFEQDCEPIWSERDKSVVEMCSELGIECVEKVSHTLWDPKLVIRTNGGIPPLTYQMFM

HTTSVIGPPPRPCSEIDFDRVHFGVVPPYLWQELGVLNDIPTPEDFGLEKEKGNKLVIWVGGETRALKHLESRVQVCI

>Apolygus_luc_KAF6206655.1_GE061_0178 KAF6206655.1

MAPCCSRQEIRDIVTPPRDWIFPRAVTLRSHVFHPTTLPFVSVANFVSAVFPEKSLQLTAFLGWHCFMCYSDSPKRVTSS

LPTIEINDILDCLGNNNLIQLRGLRLHDNPSLKQGLSNATTFRCIFILDPWFAGSSNVGINKWRFLLQCLDDLDRSLRKL

NSRLFVIRGQPADILPKLLKEWGTTCLSFEEDPEPFGRVRDQNIIALCRSMNITVHTSVAHTLYKLEAIIEKNHGKAPLT

YHQFQGIISSMDPPPQPEPPVTLATIGRAKSIIREDHEDKFGVPTLEELGFDIEGLRPGVWLGGESEALSRLERHLERKA

WVASFGRPKMTPQSLHASQTGLSPYLRFGCLSTRLFYYQLNDLYRKIKRAIPPLSLHGQILWREFFYCAATRNPNFDRMV

GNPICVQVPWDKNPEALAKWANGQTGFPWIDAIMTQLREEGWIHHLARHAVACFLTRGDLWLSWEEGMKVFDELLLDADW

SVNAGMWMWLSCSSFFQQFFHCYCPVRFGRKADPNGDYIRKYLPILKNMPTKYIHEPWNAPENVQKAVKCVIGVDYPVPM

LNHSVVAKNNIERMKQVYHQLIKYKGPGIMSSPSGNYQEKKKEETIYDHNNIFATPVAPTLVKPASNIR

>Apolygus_luc_KAF6213161.1_GE061_0108 KAF6213161.1

MATAVLRTSRKAISVHLHLLEVRADKIFRRIYIIRMASAAKKLKISGESSSSTNKELKDFLHSVEKNRKEAATSIADFKF

NKNRVRVLSKEEAVADDNWALLYAQKLALKFKLSLRVCFCTLPKYMDSTIRQSMFMLRGLEEVNAELKQLDIPFHLLYSN

NKEEVAQNVLNLVDDHDVGCVVVDFSPLKIARAWVDDLKEILPKDIPLCEVDAHNIVPCWVASDKLEYGARTIRNKINSK

LGEFLTKFPPLIQHPYPVDKHSWSINWEDEAKKFEVDRTVEEITWAQPGYQAGMKTLHEFCEKRLRNFATKRNNPLVNAL

SNLSPWYHFGQISIQRCILHVNTFKSKYNESVNAFCEESIVRRELADNFCFYNAKYDKIDGAYDWAKKTLNDHKKDKRTY

VYSCDELASSKTHDDLWNSAQIQLVKEGKMHGFLRMYWAKKILEWTPSPEEALRIALYLNDRFSIDGRDPNGFVGCMWSI

CGIHDQGWREREIFGKIRFMNYDGCKRKFDVPAFVARYGGKVYKPSKK

>Arctia_pla_unname_CAB3237253.1 CAB3237253.1

MSSAAETLPASNTRRHPPDTSLPPPENPFGRCNGGKHTVHWFRKGLRLHDNPALREGLTNAVTFRCVFIIDPWFASSSNV

GINKWRFLLQCLEDLDRSLRKLNSRLFVVRGQPADALPKLFREWGTTALTFEEDPEPYGRVRDHNIMSKCREVGITVTSR

VSHTLYKLDQIIERNGGKAPLTYHQFQALIASMPPPPPAEKPITAHMLNGATTPLTDDHDDRFSVPTLEELGFEIEALKP

PVWIGGESEALARLERHLERKAWVASFGRPKMTPQSLLASQTGLSPYLRFGCLSTRLFYYQLTELYKRVKRVRPPLSLHG

QILWREFFYCAATRNPNFDRMEGNPICVQIPWEKNQEALAKWASGKTGFPWIDAIMIQLREEGWIHHLARHAVACFLTRG

DLWISWEEGMKVFDELLLDADWSVNAGMWMWLSCSSFFQQFFHCYCPVRFGRKTDPNGDFIRRYIPALKNMPTRYIHEPW

VAPDAVQQSARCLIGRDYPLPMLDHSKASQVNIERIKQVYAQLAKYKPQGTLNPNTVPRPNVMQSSPSPTSIIASINQSN

YLCSQTPEPPTTTPQIIPYKDNEVFQRPMQNSIRPDFVKPFKRVIIVQQENTKVTQPDNNKKQTKENYIENEQLEVSYKT

TDSRNEKQENYDLKNLVINNYVEGFPTTQEIFTNQQPNRNEYTQQTLKMNNFSYEKQKFYLSALKDSEMHVHVVHNDTPQ

TFSPPNLNREASH

>Arctia_pla_unname_CAB3243389.1 CAB3243389.1

MTKVPSVIHWFRLDLRIHDNLALRNAINEAENRKHHLKPIYFLDPDIKDKIGINRLRFLIQSLQDLNSNLKKLNSRLYCI

RGNAINYLPKLFEDWQVKFLTCQVDIDPIYVEQDEVIEKIAEEKDIFIVKRVQHTVYDYNNVLKKNNGSVPMTYQKFLSL

VQDVPVKECIEITKPISDDCKSKDGDLKKYDVPTLEEFQIDESNLEPLKYPGGETEGVKRLHMYMGRREWVCKFEKPNSS

PNSIEPSTTVLSPYISHGCLSAKLFYHKLKEAENGMPHSDPPVSLLGQLMWREFYYTAGAGTKNFDKMVGNPVCTQIPWG

KNDEHLKAWAEGKTGYPFVDAIMRQLKQEGWIHHLARHMVACFLTRGDLWISWEEGAKVFEGYLLDYDWSLNAGNWTWLS

ASAFFYKFFRVYSPIAFGKKTDKEGLYIRKYVPELKKYPTAFIYEPWKAPKSIQTTAGCVVGVDYPKRIVDHDKIHKENC

TKMSTAYKLNKERKAMKRPFPID

>Arctia_pla_unname_CAB3253117.1 CAB3253117.1

MLGGSVLWFRHGLRLHDNPSLHSAIEDRSVPFFPIFIFDGETAGTKLVGYNRMRYLLEALDDLDSQFKQYGGRLIMLKGQ

PNIVFRRLWEEFGIRKLCFEQDCEPVWRARDDSVKVACREIGVTCREHVSHTLWEPDTVIKANGGIPPLTYEMFLHTVAT

IGDPPRPSPDVDLAGVMFGTLPECFYQEFTVFDKAPKPEDLGVFLENEDIRMIRWVGGERPALKQMSQRLAVEYETFRRG

SYLPTHGNPDLLGPPISLSPALRFGCLSVRSFYWSVQDLFREVHRGQQSANNFITGQLIWREYFYTMSINNPNYGQMANN

PICLDIPWKNPESDELQRWMEGRTGFPFVDAAMRQLRTEGWLHHALRNTVASFLTRGTLWLSWEHGLHHFLKDLLDADWS

VCAGNWMWVSSSAFEALLDSSECACPVQLGQRLDPSGEYVKRYVPELARMPVEYIYEPWKAPIDVQERANCIIGKEYPAP

VVNHLAAAQRNRNAMKELRHILQKAPPHCCPSSEEEIRQFMWLNE

>Arctia_pla_unname_CAB3256320.1 CAB3256320.1

MSSTISPCQPPRTNAEKMWPYIEYYVGERDEEHRKDGDGTHHWSGAESLATYSGRLLRDTMHGVGEYRCRYRGEAHQADT

YEGHFYNNCMHGCGLTSYANGRVFSGLYYNNMRWGPGVETYARLRENVGLWRGLQLVRLSWRPETHSVVPDLMSTPEGHD

IAAKHRNLLVSTIRTIGETNPALELLKQCGAQPLLAAQNWTKLYPKNCTDEKSQLFHSDVFERAYYGNEIVTLEPFIDDT

KSKTQNESQQASNSVLGNDQDIFYSWNNNDVIVHMMQHSYKHSKQFTKSKINLRSILNGPRDLFKPAAEHELDCRTFLMA

CFLGYIKNVAQLINEYDVNPNVTDVQGNSALMYAACGNRTDVIHFLVEAGAEINNYNDACCTPLGAALINYACAELDIHP

SDIIKAILPIGIGTTGVATEKNVSEWEIDRNLGIPFTLNKKDLEAGAKTAKNVQVQAAINKKSKSFQSIGSKVFKKIDMI

AARESKTDTQSKSSDLFGGENRLYLTISHEFETIVNDMFLKTNGVTPVNYIFQVHDMVLEINAELEEQKKIPEKKDSKKE

KENAKVAKDVLKKPSGDLPCLSMDQSSSMKLRIKIKTEEMENLKATITQLLLHGADPRRVRCPQPALFMAIVAHAPDLIE

TLVKYGADVNEVYPQSLEYTALDIAVSQPLNYKNLEVIRTLLEMGANSNHRLPINNSDVDSDPNEPGPTLLHAVLAKQVE

DESYEDVRHQLLELLLKYGCDPIAQFKGRASMDIAISKSLDIFDIFIESPNTDLNAIINELNQNILVKMFYLPFCRTIGV

SERLQMLTNLLLFGADPLLKCQNAENVYDNLFVYAKSTLAELEKLKPASPTGKQDNKKSKNEVKPKEKTKSVVASSTKSV

LGAKALGTDEVGDYKQALSLVTNCARLLIIRWIQAELLKELIVIVDRFKHRHWNMILKEYKNSKRLGLWLTPTRCLEIWD

ILKTTKKRIYNDNKVLKHVLCIVIFYFKKMKKSLRTAAISSEDKTTIESEVTYFVRENKLVTKVGQVLKRPYVTPEITPA

DEINKFNVCFECAIPLKDEKIPCKMSPKKLKLSVTSPDSKTTNTDIGEFMKKLQSKREDTAKSILDFRFNKKRVRIVSQE

QMVPDNCQGVVYWMSRDSRVQDNWAFLFAQKLALKNEVPLHVCFCLIAKYLDASVRQFDFLIKGLEKVAVDCKKLNISFH

LLEGSGAEVLPQWVEEHNIGAVVCDFNPLRVPLGWLEGCRKKLKKDVPLIQVDAHNIVPCWVASEKQEYSARTIRNKITS

KLDEYLTEFPPVIKHPYTSKFEPEPIDWNDAIESREADKNVGPVAWARPGYDEALKMLKSFIDMRLKVFATKRNDPTIDA

LSNLSPWFHFGQISVQRVALCIQEYKTKYTESVNAFLEEAIVRRELADNFCFYCEHYDSIKGASAWAQKTLDDHRKDKRT

HIYTCEQLAKAETHDDLWNSAQIQLVKEGKMHGFLRMYWAKKILEWTPSPEDALKYAIYLNDHFSIDGRDPNGYVGCMWS

VCGIHDQGWAERSVFGKIRYMNYDGCKRKFDIKTFIARYGGKVHKYVPKK

>Aricia_age_CRY-1_XP_041976072.1 XP_041976072.1

MPIVKQKLQIIHNIVCKNICTMSQEATVVHWFRLDLRLHDNLALRNAINEAENRKQKLRPVYVIDPEIKSRAGENRIRFL

IQSLQNLDQNLRKINTRLYVVKSNNIDCLTTLFEKWNVRFVTMQVDIDQDVRKQEEIVEDYCNEKDIFIVKRVQHTVYDF

NSVLKKNNGSIPMTYQKFLSLVNDIQVKEVIHITKQVPNECKAPDFDSEDFSIPTLCELGIEESTLTECKYPGGETEGLK

RLDTYMAKKQWVCSFEKPNSSPNSIEPSTTVLSPYISHGCLSAKLFYHKLKEVESGMKHTMPPVSLMGQLMWREFYYTAG

AGTKNFDKMVGNAVCTQIPWGKNEAHLKAWAGGKTGYPFVDAIMRQLKQEGWIHHLARHMVACFLTRGDLWVSWEEGAKV

FEYLLLDYDWSLNAGNWMWLSASAFFYKYFRVYSPVAFGKKTDKTGLYIRKYVPELKKYPTEFIYEPWKAPKSVQRTAGC

IIGEGYPNRIVDHDTIHKENLQKMSLAYKSNKEKKALKRPRTSVV

>Aricia_age_CRY-1_XP_041978712.1 XP_041978712.1

MGSILWFRNGLRLHDNPSLLRAVEDKSGPLFPIFVFDGETDGSTVAGYNRMRYMLEALDDLDGQFRKHGGRLFMLRGKPE

VIFKRLWEHFGIRRLCFEQDCEPTSKARDERVRGACRVLGVHVYEHVAHTLWDPANVVVANGGIPPLTYEMFLHTVGIIG

DPPRPVADADLTDVKFVELPASFYEEFQVFEKAPKPDALGVYLEGEDIRMIRWVGGETAAMRQMQHRLATEYETFCRGSY

LPTHGNPDLLGPPVSLSPALRFGCLSVRRFYWALQDLYHQVHQGRLSATHFITGQLIWREYFYTMAINNPQYGQMAGNPI

CLDIPWKQPQGDELQRWKEGRTGFPFIDAAMRQLRLEGWLHHVLRNTVASFLTRGTLWLSWEHGLQHFLKYLLDADLSVC

AGNWMWVSSSAFEALLDSGECACPVRLGQRLEPSGRYVRRYVPELTGIPDEYIYEPWKAPLDVQTRAGCIIGKDYPAPVV

DHVACAHQNRNAMEELRRILEKAPHCCPSSEEEIRQFMWLDQSDISSVVS

>Aricia_age_CRY-1-_XP_041974732.1 XP_041974732.1

MSGAAKTLRASVTQSSEPLAPPPPPPPKPIFLKPHEKHTVHWFRKGLRLHDNPALREGLHHAATFRCVFIIDPWFASSSN

VGINKWRFLLQCLEDLDNSLKKLNSRLFVVRGQPADALPKLFREWGTTFLTFEEDPEPYGRVRDQNITTKCQEVGISVVS

RVSHTLYKLDSIIERNGGKAPLTYHQFQALIASMPPPPAAESTITKQTLNGAMTPILQDHDDRFGVPTLEELGFDTENLK

PPIWIGGENEALARLERHLERKAWVATFGRPKMTPQSLLASSTGLSPYLRFGCLSTRLFYFQLTELYKRVKRVRPPLSLH

GQILWREFFYCAATRNPNFDRMEGNPICVQIPWEKNQEALAKWANGQTGFPWIDAIMIQLRDEGWIHHLARHAVACFLTR

GDLWISWEEGMKVFDELLLDADWSVNAGMWMWLSCSSFFQQFFHCYCPVRFGRKTDPNGDFIRKYIPALKNMPAKYIHEP

WVAPQPVQEAAQCIIGRDYPLPMVDHAKASKINIERIKQVYEQLAKYKPQGALNPQVIQRPNVMQSSPSPTSIIRSINQS

NYLCSQAPESQPPSSNTPDIYKQEDVFICPNKNHIRSNIDSKRPQFKQVVIVQGDPKRNQQNTSITENNYIVNTFKMKQN

MEFPHPGKPNNYHMKNFVINNHLQNYANDLLITNRDKKKNETFTNSSKVDEYIMTRPKYYYTENEIISNKNGNPNFTPQP

LTGKFIPKDETVTRETESGSHNHTIASNNETQYTKTNKDLTSEK

>Aricia_age_PL_XP_041975665.1 XP_041975665.1

MHINFFSNHCAKAVRKFFTMASAAKKPKLASSEGSSSKQSLEEFRDSIQKKREVTADSIMNFKFNKKRVRIISQEQLVAD

KCEGIVYWMSRDSRVQDNWAFLYAQKLALKNEVPLHVCFCLIAKYLDASVRQFHFLVKGLEKVAEECKKLNISFHLLEGS

GAEALPQWIIDHNIGAVVCDFNPLRVPLGWVEGAKKKFKKDVPLIQVDAHNVVPCWVASDKQEYSARTIRNKINSKLDEY

LTQFPPVIKHPYTSKFKPEAIDWDEAIQSREADKSVGPVEWASPGYDSALKTLRDFLQKRLKQYATKRNDPTLDALSNLS

PWFHFGQISVQRVALCVKEHQKQCTESVNAFLEEAIVRSELADNFCFYNENYDSVKGAHAWAQKTLDDHRKDKRTHIYTL

DQLKDSKTHDDLWNSAQIQLVKEGKMHGFLRMYWCKKILEWTTSPEEALKFAIYLNDHYSIDGRDPNGFVGCMWSICGVH

DQGWAERAVFGKIRYMNYDGCKRKFNVAAFVARYGGKVHKYVPHH

>Asbolus_ver_CRY_2_RZC35584.1 RZC35584.1

MSGAIADTGRASKGQDKHMVHWFRKGLRLHDNPSLREGLKGAQTFRCVFVLDPWFAGSSNVGINKWRFLLQCLEDLDRSL

RKLNSRLFVIRGQPADALPKLFKEWGTTSLTFEEDPEPFGAVRDHNLTTLCQELGITVIQKVSHTLYHLQHITERNGGRA

PLTYHQFLAVIACMGPPPQPESPVTFSSLNGAHTPLSDDHDEKYGVPTLEELGFDTEGLLPPVWQGGESEALARLERHLE

RKAWVASFGRPKMTPQSLLPSQTGLSPYLRFGCLSTRLFYYQLTDLYKKIKKAFPPLSLHGQLLWREFFYCAATKNPNFD

KMIGNPICVQIPWDKNAEALAKWANGQTGFPWIDAIMTQLREEGWIHHLARHAVACFLTRGDLWLSWEEGMKVFEELLLD

ADWSVNAGMWMWLSCSSFFQQFFHCYCPIKFGRKADPNGDYIRKYLPVLKNMPVQYIHEPWTAPEHVQRAAKCIIGKDYS

LPMVNHAAASRINIQRMKQVYQQLSNYRSLENSSKFKDGYQDQPSIVTVGNPNRNNQ

>Athalia_ros_CRY-1_XP_012266148.2 XP_012266148.2

MTGSDKIVGTHCGGRQPIRGDGTKHTVHWFRKGLRLHDNPSLREGLLGATTFRCIFILDPWFAGSTNVGINKWRFLLQCL

EDLDQSLRKLNSRLFVIRGQPADALPKLFKEWGTTNLTFEEDPEPFGRVRDHNISALCQELGISVVQRVSHTLYKLDKII

EKNGGKAPLTYHQFQNVVAAMDSPLLPEPTVGPDCTAGAYTPLKDDHDDVYGVPTLEELGFDTKNLKAPVWIGGEGEALV

RLERHLERKAWVASFGRPKMTPQSLLASQTGLSPYLRFGCLSTRLFYYQLTNLYKKIKKAVPPLSLHGQLLWREFFYCAA

TRNPSFDRMQGNPICVQIPWDKNVEALAKWANGQTGFPWIDAIMTQLREEGWIHHLARHAVACFLTRGDLWISWEEGMKV

FDELLLDADWSVNAGMWMWLSCSSFFQQFFHCYCPVRFGRKADPNGDYIRRYLPVLKNFPTRYIHEPWNAPLSVQRASKC

IIGKEYSLPMVNHGKSSRINIERMKQVYQKLNKYRGNGPPTCPTENTIGA

>Athalia_ros_PL_X1_XP_048508926.1 XP_048508926.1

MQFAYLFRVENIRVRACIKRATILYRCHQVKTIRAAIGKFSKNFPPLRSIPHTAESITTFKFNKRRVKILTDLDKVRDKS

NGIVYWMFRDSRVQDNWAFLFAQKIAIKNAVPLHICFSVLPKFLDATIRHFKFLLKGLEQVEAECNNLNINFHLLLGEPN

VEILKFVTKYNMGAVITDFFPLRVHLSWVEDLKKKLPKDVPICQVDAHNIVPCWEASDKLEYSARTIRNKINSKLSEYLT

PFPPVIKHPHITKLKFVDNDWKNVWKHIKVDETVKEITWAQPGYEGGILELESFIEKRLKIYESKRNDPLGNALSNLSPW

FHFGMISVQRCILEVSKYKKTHTKSVESFMEEAIVRRELSDNFCFYNEHYDSLKGAKQWALDTLDKHREDKREYLYTLKE

FEKSMTHDDLWNSAQIQLVQEGKMHGFLRMYWAKKILEWTSTPEQALEWSIYLNDKYSMDGRDPSGYVGCMWSICGIHDQ

GWAERSIFGKIRYMNYKGCQRKFDVKAFVARWGGKVHNKKK

>Atta_cep_PREDIC_XP_012059193.1 XP_012059193.1

MTGSSKNEMGQAVTSGVRGDGGKHTVHWFRKGLRLHDNPSLKEGLAGASTFRCVFVLDPWFAGSTNVGINKWRFLLQCLE

DLDCSLRKLNSRLFVIRGQPADALPKLFKEWGTTDLTFEEDPEPFGRVRDHNISALCKELGISVVQRVSHTLYRLDEIIE

RNSGKPPLTYHQFQNVVAGMDPPEPPVPTVTAACIGSAYTPLKDDHDDHYGVPTLEELGFDTESLLPPVWVGGESEALAR

LERHLERKAWVASFGRPKMTPQSLLPSQTGLSPYLRFGCLSTRLFYYQLTDLYKKIKKAVPPLSLHGQLLWREFFYCAAT

KNPNFDKMQGNPICVQIPWDKNVEALAKWANGQTGFPWIDAIMTQLREEGWIHHLARHAVACFLTRGDLWISWEEGMKVF

DELLLDADWSVNAGMWMWLSCSSFFQQFFHCYCPVRFGRKADPNGDYIRRYLPVLKNFPTRYIHEPWNAPLSIQHAAKCI

IGKEYSLPMVNHNKSSRINIERMKQVYQQLNKYRDNGTSFKGENIGLLNALLASPTKDGDEEKRKQDSPNRENEQKMEAI

NSPTQQQ

>Atta_col_PREDIC_XP_018059414.1 XP_018059414.1

MTGSSKNEMGQAVTSGVRGDGGKHTVHWFRKGLRLHDNPSLKEGLAGASTFRCVFVLDPWFAGSTNVGINKWRFLLQCLE

DLDCSLRKLNSRLFVIRGQPADALPKLFKEWGTTDLTFEEDPEPFGRVRDHNISALCKELGISVVQRVSHTVYRLDEIIE

RNSGKPPLTYHQFQNVVAGMDPPEPPVPTVTAACIGSAYTPLKDDHDDHYGVPTLEELGFDTESLLPPVWVGGESEALAR

LERHLERKAWVASFGRPKMTPQSLLPSQTGLSPYLRFGCLSTRLFYYQLTDLYKKIKKAVPPLSLHGQLLWREFFYCAAT

KNPNFDKMQGNPICVQIPWDKNVEALAKWANGQTGFPWIDAIMTQLREEGWIHHLARHAVACFLTRGDLWISWEEGMKVF

DELLLDADWSVNAGMWMWLSCSSFFQQFFHCYCPVRFGRKADPNGDYIRRYLPVLKNFPTRYIHEPWNAPLSIQHAAKCI

IGKEYSLPMVNHNKSSRINIERMKQVYQQLNKYRDNGTSFKGENIGLLNALLASPTKDGDEEKRKQDSPNRENEQKMETI

NSPTQQQ

>Bactrocera_dor_CRY-1_XP_011206676.1 XP_011206676.1

MAKRANVMWFRHGLRLHDNPALLEAISDKTEGIALIPLFIFDGESAGTKTVGYNRMSFLLNSLAEIDKQLKAIRGASDIA

GKLYLFQGNPTTVFRRLNEYYRLNKICFEQDCEPIWNRRDDSVRALCNDLDIEAVEKVSHTLWDPRTVISTNGGIPPLTY

QMFLHTVEIIGAPPRPVEDPEWDGVEFLKLTDNMLMELNAFWQFPTPEDFNIFPDNISYVAKVKWRGGEQQALLHLAERL

KVEERAFKNGYYLPNQANPNILESPKSMSAHLRFGCLSVRRFYWSVHDLFKHVQIEAFYHRIHMAGGEHITGQLIWREYF

YTMSVNNPYYDRMEGNAICLNIPWAPPNQEQLQSWRSGQTGFPLIDAAMRQLLAEGWLHHTLRNTVATFLTRGALWQSWE

HGLRHFLKYLLDADWSVCAGNWMWVSSSAFERLLDSSLVSCPIAFSKRLDPKGEYIRQYVPELAKIPQEYIHEPWRMPQE

MQENYECVIGVQYPERIVDLAKVSKRNTLAMQTLRQSLIAGGAPDEGPPHCRPSNEEEVHQFFWLVD

>Bactrocera_dor_CRY-2__XP_011210045.2 XP_011210045.2

MESKITLVHWFRKGLRVHDNPALALVFSKAVSQPTKYCVRPIFILDTALLEWLRVGANRWRFLQQTLADLDTNLRKLNTQ

LYVVRGTPATVFQRIFKEWRVSLLTFESDIEPYALKRDAEIQRLAKAARVKVDAFCSHTIYNPDLVIQRNGGNAPLTYQK

FLSVIEKINVPLPVANPERLSDEQKPPKDTMEQENTNCYAYPTLDELVKRPEELGENKFVGGETEGLRRLDASLSNESWV

AAFEKPNTAPNSLEPSTTVLSPYLKFGCLSSRLFHQRLMSILKRQTKHSKPPVSLLGQLLWREFYYTAAASEPNFDRMLG

NKFCMQIPWETNEVHLSAWTNGRTGYPFIDAIMRQLRQEGWIHHLARHAVACFLTRGDLWISWEEGQKVFEELLLDQDWA

LNAGNWMWLSASAFFYQYFRVYSPVAFGKKTDPTGAYIRKYVPELAKYPAGCIYEPWKATLSAQREYGCVLGEDYPHRIV

IHETVYKENIKRMTAAYKINREVKEGKSPCDESGKDKRRKVGSSTQLTKKLRT

>Bactrocera_dor_PL_XP_011207519.3 XP_011207519.3

MKRSNNATNAVTKPKKTKSAASSGVDTNNESISAVGINEKASSTTISVQSLQQRRLATAESIAEFSFNKKRVRVLNSVEE

VSESRSGGVLYWMSREARVQDNWAFLYSQRLALKLKLPLLVTFCLVPKYLNATLRQYKFLIGGLKEVERECRELSIPLHL

CLGSPKDRLPELINKYKISALVCDITPLRVPQQWVEEVKEKLPIGVPFIQVDAHNVVPLWVASDKQEYGARTIRGKINLK

LPEFLTEFPPVIKHPHNKNIISSYEPVNWQEAEAALECDRSVDEVDWAQPGYTAACAQLQSFCTQRLRIFNEKRNDPTIN

ALSGLSPWFHFGHISVQRCVLEVLRLKSKYKASVEAFCEEAIVRRELADNFCYYNEHYDSLKGLHAWAAKTLEDHRKDKR

SPAYTLAEFEQARTHDDLWNAAQLQLLREGKIHGFLRMYWAKKILEWSSTPDAALETAIFLNDKYSLDGLDPNGYVGCMW

SIGGVHDQGWAERPIFGKIRYMNYKGCQRKFNVAAFVARYGSKVHDSK

>Bactrocera_dor_PL_XP_011207520.2 XP_011207520.2

MKRSKEKYNLRSKAESPSSSEEEDISGPSTDISPQLAISKHSLMQRRFSTANSISEFPFNKKRVRVLSSEDKVSEVHFGG

ILYWMSRDARVQDNWALLFAQRLALKFELPLLIIFCITPQDANITKRQYEFLLSGLEELEHELHELSIPLHLCFGSTACC

LTEFVRLHEISAVVCDFSPLREHQQCVEEIKLVLPVDIPFTQVDAHNIVPLWITSHKQEFVPFYMRRRIYPILDEYLTEF

PPVIKHPHNKNIFNIKRADWKKARNWFDGDCAVGVVKWAKSGYTAACEQLQSFCMKRLPIYGEKRDDPTVNALSGLSPWI

RFGQISAQRCVLEVRRYSSKCQKSVEAFCEEAIILRELADNFCYYNKNYDNLKGIYSWAFNTLDEHRYDERNPSYKLSKF

EQANTHDDLWNAAQIQLVREGKMHGFLRKYWAKKILEWSNSPEAALETAIYLNDKYSLDGRDSNGYVGCMFSIGGLHDGA

YRNRPIFGKIRYMNYKGCQRKFDVTAFVARYNTKDVVN

>Bactrocera_lat_PREDIC_XP_018787748.1 XP_018787748.1

MAKRANVMWFRHGLRLHDNPALLEAISDKAEGIALIPLFIFDGESAGTKTVGYNRMSFLLNSLAEIDKQLKAIRGASDIA

GKLYLFQGNPTTIFRRLNEYYRLNKICFEQDCEPIWNRRDDSVRALCNDLDIEAVEKVSHTLWDPRTVISTNGGIPPLTY

QMFLHTVEIIGAPPRPVEDPEWDGVEFLKLTDNMLMELNAFWQFPTPEDFNIFPDNISYVAKVKWRGGEQQALLHLAERL

KVEERAFKNGYYLPNQANPNILESPKSMSAHLRFGCLSVRRFYWSVHDLFKHVQIEAYYHRIHMAGGEHITGQLIWREYF

YTMSVNNPYYDRMEGNAICLNIPWAPPNQEQLQSWRSGQTGFPLIDAAMRQLLAEGWLHHTLRNTVATFLTRGALWQSWE

HGLRHFLKYLLDADWSVCAGNWMWVSSSAFERLLDSSLVSCPIAFSKRLDPKGEYIRQYVPELAKIPQEYIHEPWRMPQE

MQENYECVIGVQYPERIVDLAKVSKRNTLAMQTLRQSLIAGGAPDEGPPHCRPSNEEEVHQFFWLVD

>Bactrocera_lat_PREDIC_XP_018789201.1 XP_018789201.1

MESKTTLVHWFRKGLRVHDNPALALVFSKAVNQPTKYCVRPIFILDTALLEWLRVGANRWRFLQQTLADLDTNLRKLNTQ

LYVVRGTPATVFQRIFKEWRVSLLTFESDIEPYALKRDAEIQRLAKAARVKVDAFSSHTIYNPDLVIQRNGGNAPLTYQK

FLSVIEKLIVPLPVANPEILPDEQKPPKDTMEKENTNCYAYPTLDELVKRPEELGENKFVGGETEGLRRLDASLSNESWV

AAFEKPNTAPNSLEPSTTVLSPYLKFGCLSARLFHQRVMSILKRQTKHSKPPVSLIGQLLWREFYYTAAASEPNFDRMLG

NKFCIQIPWETNEVHLSAWTNGRTGYPFIDAIMRQLRQEGWIHHLARHAVACFLTRGDLWISWEEGQKVFEELLLDQDWA

LNAGNWMWLSASAFFYQYFRVYSPVAFGKKTDPTGAYIRKYVPELAKYPAGCIYEPWKAHLSAQREYGCVLGEDYPHRIV

IHETVHKENIKRMTAAYKINREVKEGKSPTDESGKDKRKKVGSSALLTKKLRT

>Bactrocera_lat_PREDIC_XP_018795683.1 XP_018795683.1

MKRVKSASNAVEKPKKTKFAASSGVDTNNDSISQVGINEKAASTTITVQSLQQRRLATAESIAEFSFNKKRVRVLNSVEE

VSESRSGGVLYWMSREVRVQDNWAFLYSQRLALKLKLPLLVTFCLVPKYLNATLRQYKFLIGGLEEVERECRELSIPLHL

RIGSPRDHLPELVNKYKISALVCDFTPLRVPQQWVEDVKEKLSIEVPFIQVDAHNVVPLWVASDKQEYGARTIRGKINSK

LSEFLTEFPPVIKHPHNKSLISNYDPVNWQEAEAALQCDRSVDEVDWAQPGYTAACAQLQSFCTKRLRIFNEKRNDPTIN

ALSGLSPWFHFGHISVQRCVLEVMRLKSKYKASVEAFCEEAIVRRELADNFCYYNEHYDSLKGLHAWAAKTLEDHRKDKR

SPAYTLVEFEQARTHDDLWNAAQLQLLHEGKMHGFLRMYWAKKILEWSSTPDAALETAIFLNDKYSLDGLDPNGYVGCMW

SIGGVHDQGWAERPIFGKIRYMNYKGCQRKFDVAGFVARYGSKVHNSK

>Bactrocera_lat_PREDIC_XP_018795691.1 XP_018795691.1

MIGMKRSKEKYNLRSKDESPSSSEEENISDPSTDISPQLVISKHSLMQRRLSTAKSISEFPFSKKRVRVLSSEDKVSEVQ

CGGILYWMLRDARVQDNWALLFAQRLALKFELPLIIIFCITPQDANIAKRQYEFLLSGLEELEYELHELSIPLHLCFGAA

ACCLIEFVRLHEISAVVCDFSPLREHQQHVDEVKLLLPVDIPFTQVDAHNIVPLWVTSHKQEFVPFYMRRRIYPILDEYL

TEFPPVIKHPHNKNIFNIKRADWKKARNWFDGDCSVGVVKWAKSGYTAACEQLQSFCTKRLPIYGEKRDDPTVNALSGLS

PWIRFGQISAQRCALEVRRYSSKCQKSVEAFCEVAIIHRELADNFCYYNKNYDNLKGIYSWAFNTLDEHRYDERNPSYQL

SEFEQANTHDDLWNAAQIQLVREGKMHGFLRKYWAKKILEWSNSPEEALEIAIYLNDKYSLDGRDSNGYVGCMFSIGGLH

DGAYRNRAIFGKIRYMNYKGCQRKFDVRAFVARYNTNEVIN

>Bactrocera_neo_CRY-1_XP_050318184.1 XP_050318184.1

MAKRANVMWFRHGLRLHDNPALLEAISDKTEGIALIPLFIFDGESAGTKTVGYNRMSFLLNSLAEIDKQLKAIRGASDIA

GKLYLFQGNPTTVFRRLNEYYRLNKICFEQDCEPIWNRRDDSVRALCNDLDIEAVEKVSHTLWDPRTVISTNGGIPPLTY

QMFLHTVEIIGAPPRPVEDPEWDGVEFLKLTDNMLMELNAFWQFPTPEDFNIFPDNISYVAKVKWRGGEQQALLHLAERL

KVEERAFKNGYYLPNQANPNILESPKSMSAHLRFGCLSVRRFYWSVHDLFKHVQIEAFYHRIHMAGGEHITGQLIWREYF

YTMSVNNPYYDRMEGNEICLNIPWAPPNQEQLQSWRSGQTGFPLIDAAMRQLLAEGWLHHTLRNTVATFLTRGALWQSWE

HGLRHFLKYLLDADWSVCAGNWMWVSSSAFERLLDSSLVSCPIAFSKRLDPKGEYIRQYVPELAKIPQEYIHEPWRMPQE

MQENYECVIGVQYPERIVDLAKVSKRNTLAMQTLRQSLIAGGAPDEGPPHCRPSNEEEVHQFFWLVD

>Bactrocera_neo_CRY-2_XP_050321951.1 XP_050321951.1

MESKTTLVHWFRKGLRVHDNPALTLVFSKAVSQPTKYCVRPIFILDTALLEWLRVGANRWRFLQQTLADLDANLRKLNTQ

LYVVRGTPATVFQRIFKEWRVSLLTFESDIEPYALKRDAEIQRMAKAARVKVDVFCSHTIYNPELVIQRNGGNAPLTYQK

FLSVIEKLNVPLPVANPERLSDEQKPTKDTMEQQNSSCYAYPTLDELVKRPEELGENKFVGGETEGLRRLDASLSNESWV

AAFEKPNTAPNSLEPSTTVLSPYLKFGCLSARLFHQRLMSILKRQTKHSKPPVSLLGQLLWREFYYTAAASEPNFDRMLG

NKFCMQIPWETNEVHLSAWTNGRTGYPFIDAIMRQLRQEGWIHHLARHAVACFLTRGDLWISWEEGQKVFEELLLDQDWA

LNAGNWMWLSASAFFYQYFRVYSPVAFGKKTDPTGAYIRKYVPELAKYPAGCIYEPWKATLSAQREYGCVLGKDYPHRIV

IHETVHKENIKRMTAAYKINREVKEGKSPTDGGGKDKRKKVGSSAQLTKKLRT

>Bactrocera_neo_PL-lik_XP_050326165.1 XP_050326165.1

MKRVKSATNAVAKPKKIKSAGSSGVDTNNDSFSRVGINEKAASTTISVQSLQQRRLVTAESIAEFSFNKKRVRVLNSVEE

VSESRSGGVLYWMSREARVQDNWAFLYSQRLALKLKLPLLVTFCLVPKYLNATLRQYKFLIGGLKEVERECRELSVPLHL

CFGSPKNRLPELVNKYKISALVCDFTPLRVPQQWVEEVKEKLSIEVPFIQVDAHNVVPLWIASDKQEYGARTIRGKINSK

LPEFLTEFPPVIKHPHNKNIISNYEPVNWQEAEAALECDRSVDEVDWAQPGYTAACAQLQSFCAKRLRIFNEKRNDPTIN

ALSGLSPWFHFGHISVQRCVLEVMRLKSKYKASVEAFCEEAIVRRELADNFCYYNEHYDSLKGLHAWAAKTLEDHRKDKR

SPSYTLAEFEQARTHDDLWNAAQLQLLREGKMHGFLRMYWAKKILEWSSTPDSALETAIFLNDKYSLDGLDPNGYVGCMW

SIGGVHDQGWAERPIFGKIRYMNYKGCQRKFDVAAFVARYGSKAHNSK

>Bactrocera_neo_PL-lik_XP_050326166.1 XP_050326166.1

MKRSKEKYNLRSKAESPSSSEEEDISEPSTDISPQLVISKHSLMQRRLSVASSISEFPFNKKRVRVLSSEDKVSEVQCGG

ILYWMSRDARVQDNWALLFAQRLALKFELPLLITFCITPQDANVTKRQYEFLLSGLEELEHELYELSIPLHLCFGSAACC

LPEFVRLHEISAVVCDFSPLREHQQRVEEVKSVLPVNIPFTQVDAHNIVPLWIASHKQEFVPFYMRRRIYPILDEFLTEF

PPVIKHPHNKNVFNFKLVDWKNARNWFDGDCSVGVVKWAKSGYTAACEQLQSFCTKRLPIYGEKRDDPTVNALSGLSPWI

RFGQISAQRCVLEVRRYSSKCQKSVEAFCEEAIIHRELADNFCYYNKNYDNLKGIYSWAFNTLDEHRYDERNPSYKLSDF

EQANTHDDLWNAAQIQLVREGKMHGFLRKYWAKKILEWSNSPEEALEIAIYLNDKYSLDGRDSNGYVGCMFSIGGLHDGA

YRNRAIFGKIRYMNYKGCQRKFNVTAFVARYNTKDVVD

>Bactrocera_ole_CRY-1_XP_014096643.1 XP_014096643.1

MAKRANVIWFRHGLRFHDNRALLEAISDKTEGIALIPLFIFDGESAGTKTVGYNRMSFLLNSLADIDKQLKAIRGANDIA

GKLYLFQGNPTTVFRRLNEYFRLNKICFEQDCEPIWNRRDDSVRALCNDLGIEAVEKVSHTLWDPRTVISTNGGIPPLTY

QMFLHTVEIIGAPPRPVQDPEWDGVKFLKLTDNMLMELNAFWQFPTPEDFNIFPDNINYVAKVKWLGGEQQALLHLAERL

KVEERAFKNGYYLPNQANPNILESPKSMSAHLRFGCLSVRRFYWSVHDLFKHVQIEALYQRIHMAGGEHITGQLIWREYF

YTMSVNNPYYDRMEGNAICLNIPWAPPNQEQLQSWRSGQTGFPLIDAAMRQLLAEGWLHHTLRNTVATFLTRGALWQSWE

HGLRHFLKYLLDADWSVCAGNWMWVSSSAFERLLDSSLVSCPIAFSKRLDPKGEYIRQYVPELAKVPQEYIYEPWRMPQD

MQENYECVIGVQYPERIVDLAKVSKRNTLAMQALRQSLIAGGAPDEGPPHCRPSNEEEVHQFFWLVD

>Bactrocera_ole_CRY-2_XP_014103186.2 XP_014103186.2

MESKTTLVHWFRKGLRVHDNPALALVFSKAVSQPTKYYVRPIFILDTALLEWLRVGANRWRFLQQTLADLDANLRKLNTQ

LYVIRGTPATVFPRIFKEWRVSLLTFETDIEPYALKRDAEIQRLAKAAEVNVDTFCSHTIYNPDLVIQKNCGNAPLTYQK

FLSIIEKFSVPLPVSNPERLVDEQKPPKDTMEQQNTNCYAYPTLDELVKRPDELGENKFIGGESEGLRRLDASLSNESWV

AAFEKPNTSPNSLEPSTTVLSPYLKFGCLSARLFHQRLIQILKRQNKYSKPPVSLLGQLLWREFYYTAAASEPNFDRMLG

NKFCMQIPWEINEVHLSAWTNGRTGYPFIDAIMRQLRQEGWIHHLARHAVACFLTRGDLWISWEEGQKVFEELLLDQDWA

LNAGNWMWLSASAFFYQYFRVYSPVAFGKKTDPTGAYIRKYVPELAKYPAGCIYEPWKATLSAQREYGCVLGKDYPHRIV

NHETVHKENIKRMTAAYKINREVKEGKSQTDESGKDKRKKVGSSAQLSKKRRT

>Bactrocera_ole_PL-lik_XP_014095123.2 XP_014095123.2

MKRVKSTPNAVAKLKKTKSAASSASDANNDSISPAAINEKAASSIISIESLQQRRLATAENIADFSFNKKRVRVLNTVEE

VSESRCGGVLYWMSREVRVQDNWAFLYSQRLALKLKLPLLVTFCLVPKYLNATLRQYKFLFGGLKEVEHECRELSIPLHL

CLGTPRECLPELVNKYKISALVCDFTPLRVPQQWVEEVKENLTTEVPFIQVDAHNVVPLWVASDKQEYGARTIRGKINSK

LAEFLTEFPPVIKHPHNKNIIANYEPIDWKGAEASLQCDRSVDEVDWAQPGYIAACAQLQSFCTKRLRIFNEKRNDPTIN

ALSGLSPWFHFGHISVQRCVLEVMRLKSKYKASVEAFCEEAIVRRELADNFCYYNEHYDSLKGLHAWAAKTLEDHRKDKR

SPSYTLAEFEQARTHDDLWNAAQIQLLREGKMHGFLRMYWAKKILEWSSTPEAALETAIFLNDKYSLDGLDPNGYVGCMW

SIGGVHDQGWAERPIFGKIRFMNYKGCQRKFDVAAFVARYGGKVYKSK

>Bactrocera_ole_PL-lik_XP_014095126.2 XP_014095126.2

MKRLKGKYNLRREAKSASSSEEEDISRPCTNISPQLLISKQSLNQRRLSTAKSISAFPFNKKRVRVLSAEDKVSDVHCGG

ILYWMSRDARVQDNWALLFAQRLALKFELPLLIAFCITPQDANITKRQYEFLLTGLEELEHELHELLIPLHLCFGSTACC

LSEFVRLHEISAIVCDFSPLREQQQRVEEVKLVLPSDIPFTQVDAHNIVPLWVASHKQEFVPFYMRRRIYPILDEFLTEF

PPVIKHPHNKNILNFERADWKKARNWFDGDCSVGVVKWAKSGYTAACEQLQSFCTKRLPIYGDKRDDPTANALSGLSPWL

HFGQISAQRCVLEVKRYSFKCQKSVEAFCEEAIIHRELADNFCYYNKNYDNLNGLYSWAFKTLDEHRDDERNPSYKLSEF

EKANTYDDLWNAAQIQLLREGKMHAFLRKYWAKKILEWSNSPEEALETAIYLNDKYSLDGRDPKGYVGCMFSIGGLHDGA

YRNRAIFGKIRYMNYKGCQRKFDVTAFVARYSTENVMN

>Bactrocera_try_CRY-1_XP_039961054.1 XP_039961054.1

MAKRANVMWFRHGLRLHDNPALLEAISDKTEGIALIPLFIFDGESAGTKTVGYNRMSFLLNSLAEIDKQLKGIRGASDIA

GKLYLFQGNPTTVFRRLNEYYRLNKICFEQDCEPIWNRRDDSVRALCNDLDIEAVEKVSHTLWDPRTVISTNGGIPPLTY

QMFLHTVEIIGAPPRPVEDPEWDGVEFLKLTDNMLMELNAFWQFPTPEDFNIFPDNISYVAKVKWRGGEQQALLHLAERL

KVEERAFKNGYYLPNQANPNILESPKSMSAHLRFGCLSVRRFYWSVHDLFKHVQIEAFYHRIHMAGGEHITGQLIWREYF

YTMSVNNPYYDRMEGNEICLNIPWAPPNQEQLQSWRSGQTGFPLIDAAMRQLLAEGWLHHTLRNTVATFLTRGALWQSWE

HGLRHFLKYLLDADWSVCAGNWMWVSSSAFERLLDSSLVSCPIAFSKRLDPKGEYIRQYVPELAKIPQEYIHEPWRMPQE

MQENYECVIGVQYPERIVDLAKVSKRNTLAMQTLRQSLIAGGAPDEGPPHCRPSNEEEVHQFFWLVD

>Bactrocera_try_CRY-2_XP_039952841.1 XP_039952841.1

MESKTTLVHWFRKGLRVHDNPALTLVFSKAVSQPTKYCVRPIFILDTALLEWLRVGANRWRFLQQTLADLDANLRKLNTQ

LYVVRGTPATVFQRIFKEWRVSLLTFESDIEPYALKRDAEIQRLAKAARVKVDVFCSHTIYNPELVIQRNGGNAPLTYQK

FLSVIEKLNVPLPVANPERLSDEQRPTKDSMEQQNSSCYAYPTLDELVKRPDELGENKFVGGETEGLRRLDASLSNESWV

AAFEKPNTAPNSLEPSTTVLSPYLKFGCLSARLFHQRLMSILKRQTKHSKPPVSLLGQLLWREFYYTAAASEPNFDRMLG

NKFCMQIPWETNEVHLSAWTNGRTGYPFIDAIMRQLRQEGWIHHLARHAVACFLTRGDLWISWEEGQKVFEELLLDQDWA

LNAGNWMWLSASAFFYQYFRVYSPVAFGKKTDPTGAYIRKYVPELAKYPAGCIYEPWKATLSAQREYSCVLGKDYPHRIV

IHETVHKENIKRMTAAYKINREVKEGKSPTDGGGKDKRKKVGSSAQLTKKLRT

>Bactrocera_try_PL-lik_XP_039955199.1 XP_039955199.1

MKRSKEKYNLRSKAESPSSSEEEDISEPSTDISPQLVISKHSLMQRRLSVASSISEFPFNKKRVRVLSSEDKVSEVQCGG

ILYWMSRDARVQDNWALLFAQRLALKFELPLLITFCITPQDANVTKRQYEFLLSGLEELEHELYELSIPLHLCFGSAACC

LPEFVRLHEISAVVCDFSPLREHQQRVEEVKSILPVNIPFTQVDAHNIVPLWIASHKQEFVPFYMRRRIYPILDEFLTEF

PPVIKHPHNKNVFNFKLVDWKNARNWFDGDCSVGVVKWAKSGYTAACEQLQSFCTKRLPIYGEKRDDPTVNALSGLSPWI

RFGQISAQRCVLEVRRYSSKCQKSVEAFCEEAIIHRELADNFCYYNKNYDNLKGIYSWAFNTLDEHRYDERNPSYKLSDF

EQANTHDDLWNAAQIQLVREGKMHGFLRKYWAKKILEWSNSPEEALEIAIYLNDKYSLDGRDSNGYVGCMFSIGGLHDGA

YRNRAIFGKIRYMNYKGCQRKFNVTAFVARYNTKDVVD

>Bactrocera_try_PL-lik_XP_039956686.1 XP_039956686.1

MKRVKSATNAVAKPKKIKSAGSSGVDTNNDSFSRVGINEKAASTTISVQSLQQRRLATAESIAEFSFNKKRVRVLNSVEE

VSESRSGGILYWMSREARVQDNWAFLYSQRLALKLKLPLLVTFCLVPKYLNATLRQYKFLIGGLKEVERDCRELSVPLHL

CFGSPKDRLPELVNKYKISALVCDFTPLRVPQQWVEEVKEKLSIEVPFIQADAHNVVPLWIASDKQEYGARTIRGKINSK

LPEFLTEFPPVIKHPHNKNIISNYEPVNWQEAEGALECDRSVDEVDWAQPGYTAACAQLQSFCAKRLRIFNEKRNDPTIN

ALSGLSPWFHYGHISVQRCVLEVMRLKSKYKASVEAFCEEAIVRRELADNFCYYNEHYDSLKGLHAWAAKTLEDHRKDKR

SPSYTLAEFEQARTHNDLWNAAQLQLLREGKMHGFMRMYWAKKILEWSSTPDAALETAIFLNDKYSLDGLDPNGYVGCMW

SIGGVHDQGWAERPIFGKIRYMNYKGCQRKFDVAAFVARYGSKAHNSK

>Belonocnema_kin_CRY-1_XP_033207632.1 XP_033207632.1

MTGGRDGDIGTGGIKVRGDGRKHTVHWFRKGLRLHDNPSLREGLIGASTFRCVFVLDPWFAGSTNVSINKWRFLLQCLDD

LDRSLRKLNSRLFVIRGQPADALPKLFKEWGTTNLTFEEDPEPFGRVRDLNISTLCEELGISVLHRTSHTLYNLKEIIEK

NGGRSPLTYHQFQNIVASMDSPELPAPTVTLDCLGNAITPLKEDHDDQYGVPTLEELGFDTEGLLPPVWVGGESEAIARL

ERHLERKAWVASFGRPKMTPQSLLASQTGLSPYLRFGCLSTRLFYYQLIDLYKKIKKTVPPLSLHGQLLWREFFYCAATN

NPNFNKMQGNPICVQIPWEKNVDALAKWANGQTGYPWIDAIMTQLREEGWIHHLARHAVACFLTRGDLWISWEEGMKVFD

ELLLDADWSINAGMWMWLSCSSFFQQFFHCHCPVRFGRKADPNGDYIRRYLPVLKNFPTRYIHEPWNAPMSVQRAAKCII

GRDYCLPMVNHSRSSRINIERMKHVYHNLNKYRENGEAV

>Belonocnema_kin_PL_XP_033208195.1 XP_033208195.1

MSDSSQAKKARTSSFIEAIEEDRKNAGESVLDFKFNKKRIRILTELDEVAKNCRGVVYWMFRDSRVQDNWALLYTQKIAL

KNKVPLHVCFCILPKFLDATIRHYKFLLKGLEEVERELKSLNINFTLLHGEPNSALLEFVKNYKMGAVVTDFFPLKLPMF

WVNDLKKNLPKNVPLCQVDAHNIVPCWVTSDKQEYSARTIRNKINSKLDEFLTPYPPVINHPHTSEQNLEKTDWKTTLKV

LKVDRTVDEVTWAKPGYKGGILELESFLKDRLDLYNTKRNDPTVDALSNLSPWFHFGMISVQRCILEAKKFKKKHKESIE

AFMEEAIIRSELADNFCFYNEHYDSIKGAYQWAIDTLDAHRKDKREYIYSLDQFEKSLTHDDLWNAAQNQLVEYGKMHGF

LRMYWAKKILEWTESPEDALKWAIYLNDRYSMDGCDPNGYVGCMWSICGIHDQGWKERDIFGKIRYMNYKGCQRKFAVSE

FVKKWGGKVHTHGKTEKK

>Bemisia_tab_XP_018904762.1_cry-2 XP_018904762.1

MAARGTAIHWIRKGMRLHDNPALIAAIESQKNVGEICILRPVYILDPYFRKYMRVSPNRWRFLQQALEDLDKSFRAIGTR

LYVLHGKPEEIFPELLKEWNVKLLTFESDIEPYACKRDSSVMSIAKKFGVKVLQSVSHTLYNPEIIIKKNLGKAPLTYQK

FTTVVDSIGLPPKPVDAPNALPKEAQPEDLAAAKYNVPTLAEFGLDVKSLHPTKFPGGETEGLARLAEKLSDKAWVCKFE

KPNTSPNSLEPSTTVLSPYLKFGCVSARLFYYKVKDVISKAPHSKPPTSLIGQLFWREFYYTVASVTPNFHKMEGNPVCC

QVPWDSNSEHLDAWAFGRTGYPFIDAIMRQLRQEGWIHHLARHAVACFLTRGDLWISWEEGQGVFEELLLDADWALNAGN

WMWLSASAFFHQFFRVYSPVAFGKKTDKFGDYIKKYVPELGKYPSEYIYEPWNAPKSLQERAGCVIGVDYPKRIVIHENV

YKNNIQRMSLAYKNNKQNTETTNSNPAGSSKSKTGKGGSTKRKPEAEVAASGIKKFLKKN

>Bemisia_tab_XP_018905816.1_photolyase XP_018905816.1

MSSAPPAKKAKAETSESSNGSSSSEGGFEEFLKQIEEDRKKAAKSVLEFKFNKKRVRILSKSKEVPEWGKGVIYWTFRDQ

RIHDNWAFLFAQKLALKNDVPLHFAFCRLPKFLDATLRHFKFIFEGLKETEKECKKLNIQFHFLIGCGKDVLPDFVKKHK

LGAVVIDFMPLRGPMAWADELSKSLPDGVPLVQVDAHNIVPVWETSEKLEYAARTIRNKVNGKLPEFLTQYPPVIKHPHS

GDLKASPIDWEEAEKTLEVDKSVGPVKWAKPGYRGGMQQLYTFVTKRIKNYGTARNDPNKNALSMLSPWFHFGHISVQRA

ILEVKKVKGYSESVAAFCEEAIVRRELSDNFCFYNKNYDSIKGAYDWAKKTLKDHSGDKRPYLYTKAELEQGHTHDDLWN

AAQNTLINDGKIHGFMRMYWAKKILEWTESPEQALQYAIYLNDKYSLDGRDPNGFVGCMWSICGIHDQGWSERAVFGKIR

YMNYEGCKRKFDINGYIIKHGGKVYTKGKENTMDKFVKKKK

>Bemisia_tab_XP_018906320.1_putative_c XP_018906320.1

MASPKILIYLLRRDLRVHDNPIFHKLTSMSSQANAPFTHLLPLYVFPAHQIEVSGFLSSSDEKSPYPEARSKVGKFWRCG

QLRAKFLAESVWDLKQNLETIGSGLEIRVGMLHDVVKQLVEGFKSKGVQVKGLWMTSEEGYEEKAEERQVRKIIVNAGGD

FQLWKDEKYFIDDDDIPFDDPQKYPDVFTKYRNTVEPLREAPRKVLPTPKKLPPLPQNIPPQAHPFKIPGNLKDLIAALQ

KPLDAGLGLKNPPQMPSAGASSAVPFAGGATSGQKRLKHLIESGAMTRYKDTRNGMVGTDYSSKLSLWLALGSLTAREVH

SALIDFEEGKTDVGKGAEGYGKGENKGTTHMRFELLWRDYMRLCTRKYGSRLFLVGGFRNARNIQWKHDNSIMQRWLEGT

TGIGLVDAAQRELFLTGFTSNRARQNVASFLTKHLEQDWRLGAEWYECNLVDYDVSSNWGNWQYTAGVGNDPREDRKFNP

VKQASDYDPKAEFVKAWIPEVRELQPEEAWQCWKASYAAKQKPGLRGNIMAERPLAKISFTPRSGGNDGHRGGGRSRGRA

QWF

>Bemisia_tab_XP_018915448.1_cry-1 XP_018915448.1

MSEDRSPRVSVLWFRHGLRIHDNPALLRALEDTDIFYPIFIFDGESAGTKLISYNRMKFLLESLEDLDNQFRAKGGKLYV

FFGSPIEIFAKFKAHLNFYKLTFEQDCEPIWQARDNSVKKLCDDLDIIWDESISHTLWDPREIIDTNGGSPPLTYSMFLH

TVSVIGPPPRPVEDPCWDGIKFGVLPDSLKRELDVRDKIPVPADFNLKPELKQSEYTIEWFGGENQALSNLRKRIAVEEK

AFRQGFCQPNQTQPDLLGPPTSQSAALRYGCLSVRKFFWALHDTFSDVHNMPSNSNITSQLIWREYFYTMSVDNKYYAEM

ARNPVCLNIPWSSVKDETAKANLKAWEDGKTGYPFIDALMRQLKQEGWIHHIGRNAVSSFLTRGDLWISWEEGLRHFLRY

LLDADWSVCAGNWMWVSSSAFEKLLDCNFCICPVNYGRRLDPYGEFVKRYVPELQKMPVEYVYEPWRAPLEVQEHANCVI

GRDYPKRIVDHNVVSHENKEKMKEICKSLKVRTPHCAPSDELEIRQYMWLPDSCTSHLSTLK

>Bemisia_tab_XP_018916708.1_cry-1-like XP_018916708.1

MLDDNMGQTSKHTMHWFRKGLRLHDNPSLMEGLIGASTFRCVFIIDPWFASTSNVGINKWRFLLQCLEDLDCSLRKLNSR

LFVIRGQPADALPKLFKEWNITCLSFEEDPEPFGKVRDQNIMALCKELSIEVISRVSHTLYKLDKIIEKNGGRAPLTYHQ

FQTVIAQLDPPQQAESPVSVRLIANAFTPISEDHDETYGVPTLEELGFSTEGLLPPVWLGGEAEAMTRLKRHLERKAWVA

SFDRPKMTPESLQASQTGLSPYLRFGCLSTRLFYYQLNDLYKKVIDIKKAVPPLSLHGQLLWREFFYCAATNNPNFDKMV

GNPICVQIPWDKNAEALAKWANAQTGFPWIDAIMTQLREEGWIHHLARHAVACFLTRGDLWMSWEEGMKVFEELLLDADW

SVNAGMWMWLSCSSFFQQFFHCYDPVRFGRKADPNGDYIRKYLPVLKNMPTKYIHEPWIAPGPVQKAAKCLIGEDYPLPM

VNHAVASRINIERMKQVYLQLSKYKNLGQHCMPISTKESTKSIMNSNIPIPLMDYS

>Bicyclus_any_CRY-1_XP_023936887.1 XP_023936887.1

MTKVASVIHWFRLDLRLHDNLALRNAINEAENRKQILRPIYVVDPDVQNKIGRNRLRFLFQSLRDLDENLRKINTRLFII

KGDPVVCLPQLFEEWQVKFITLQVDIDAELVKRDEVIEKICEEKDIFVVKRVQHTVYDFNTILRNNNGNIPLTYQKFLSL

VSNTQVKDTIEITKQISNDCKAPEFDSQDNDISVLEDFNFNEADLNECKYPGGETEALKRLATYMSKKQWVCNFEKPNTS

PNSIEPSTTVLSPYISHGCLSSKLFYHKLKEVENGMKHSSPPVSLLGQLMWREFYYTAGAGTKNFDKMVGNCVCTQIPWK

KNEEYLKAWAEGRTGYPFVDAIMRQLKQEGWIHHLARHMVACFLTRGDLWVSWEDGAKVFEDYLLDYDWSLNAGNWMWLS

ASAFFYKYYRVYSPVAFGKKTDKEGLYIRKYVPELKKYPAEFIYEPWKAPKSVQTTAGCIIGEDYPKRIVDHDKVHKENI

QKMNAAYKLSKEKKALKRKR

>Bicyclus_any_CRY-1_XP_023944559.1 XP_023944559.1

MAGGSVLWFRHGLRLHDNPSLNDALEDKAAPFFPVFIFDGETAGTKLVGYNRMRYLLEALNDLDNQFRKYGGRLHMIKGK

PDVVFKRLWKEFGIRKICFEQDCEPIWRMRDESVRDLCRELGVACRESVSHTLWDPDTVIRANGGIPPLTYQMFLHTVDI

IGDPPRPVADVDLSGVNFAALPDNFYTAFTVFEKVPKPEDLGIFLENEDIRMIRWVGGETAALNQMQKRLAVEYETFRRG

AYLPTHGSPDLLGPPISLSPALRFGCLSVRRFYWSLQDLFKQVHQGRLGSNQFINGQLIWREYFYTMSVNNPLYAQMSGN

PICLDIPWKEPEGDELQRWKEGRTGFPFVDAAMRQLLTEGWLHHAVRNTVASFLTRGTLWLSWEHGLQHFLKYLLDADWS

VCAGNWMWVSSSAFEALLDSSECVCPVRLGRRLEPTGHYVRRYVPELANMPVQYIYEPWSAPLHVQQKAKCVIGRDYPAP

VVDHRACEHRNRSAMQELRRVLDKAPHCCPSSDDEIRQFMWLGDESQHELDTA

>Bicyclus_any_CRY-1-_XP_023940173.1 XP_023940173.1

MSAAETLPAFVELPPEPTTPSPRGAAGKHTVHWFRKGLRLHDNPALREALVDATTFRCVFIIDPWFASSSNVGINKWRFL

LQCLEDLDDSLKKLNSRLYVVRGQPADALPKLFREWGTTALTFEEDPEPYGRVRDHNIMTKCRDVGISVTSKVSHTLYQL

DEIIERNGGKAPLTYHQFQALIASMPPPPAAEETITMATLNGAITPVADDHDDRFGVPTLEELGFETDGLQDPVWIGGEK

EGLLRLERHLERKAWVASFGRPKMTPQSLMASQTGLSPYLRFGCLSTRLFYYQLSDLYQRVKRVRPPLSLHGQILWREFF

YCAATRNPNFDRMEGNPICVQIPWEKNDDALAKWASGKSGFPWIDAIMIQLREEGWIHHLARHAVACFLTRGDLWISWEE

GMKVFDELLLDADWSVNAGMWMWLSCSSFFQQFFHCYCPVRFGRKTDPNGDFIKKYIPALKSMPVEYVHEPWMAPEAVQA

AAQCVVGRDYPLPIIDHNKASAVNIERMKQVYAQLAKFKPQGSINPQVVQRPNAMQSSPSPTSIIVNINQSNYLCSQSSD

PTQTKQYNEDYVFLKPSKNFRMRSDPNKQTSFKQVVIVHQVHKQRVHQPPAEVQYINLINSMQGSVAPENYDFTKLAINN

LLTNDPGAYLTRNDGYMQNDKSDEYTTGKPKMFYVENDEIPTDDNAQNYIPHPYNNYHCMERKSELNVDNTRNIHNLDIN

EDPSNANDEKQHDDEN

>Bicyclus_any_PL_XP_023952034.1 XP_023952034.1

MKISVVRFLNYNTKMASSAKKLKLSAPTTTAEGKNNNISDFMNQIQKKREGTAESILTFNFNKKRVRIISQEQEVADGCE

GIVYWMSRDSRVQDNWAFLFAQKLALKNEVPLHVCFCLIAKYLDASVRQFHFLIKGLEKVAAECKKLNISFHLLEGSGAD

ALPQWIIDHKIGAVVCDFNPLRVPMGWVEGAKKKFKKNIPLIQVDAHNIVPCWVASDKQEYSARTIRNKINSKLDEYLTE

FPPVIKHPHTSQFEPEPIDWDEAIESREADKSVGPVTWAAPGYDCAARVVKSFLDVRLKIFATKRNDPTLDALSNLSPWF

HFGQISVQRVALCVQQYKKQYTESVNAFLEEAIVRRELADNYCFYCEHYDSIQGASAWAQKTLDIHRKDKRSHIYTLEQL

SKAQTHDDLWNSAQIQMVKEGKMHGFLRMYWCKKILEWTPSPEDALKYAIYLNDHYSVDGRDPNGYVGCMWSICGVHDQG

WAERAVFGKVRYMNYDGCKRKFSVPAFVARYGGKAHKYAAQ

>Blattella_ger_PSN30513.1_cry-1 PSN30513.1

MSEETTNRSKVSVLWFRHGLRLHDNPALLEAIRDTDEFYPIFIFDGESAGTKAVGYNRMCYLLEALNDLNEQFKKYGGKL

YVFKGSPTQIFKRMWEELELGKICFEQDCEPLWYNRDSSVKTMCKELNINCVEKVSHTLWEPKAVIETNGGSPPLTYQMF

LHTVSVIGPPPRPVSDVNWNTVTFGKLPDYLAEEFKMFPEIPHPEDFNIFREAKTEKLIRWDGGETKALKQLLDRLDVEE

SAFRQGYYLPNQAQPDLLGPPTSLSPALRFGCLSVRRKFPSISVPNKSTIHHLVCKVRKTGTFADKKRNRKRTVLTEEKL

DDIGACFEQSPHKSLSKLTQQITHKDYNPHTLDELKENIRQQIALISMDELQRVFRNFRKRCEACIRHGGRHVHDLFNEV

HKGEPPACQNITGQLIWREYFYTMSVNNEFYAEMERNPICLNIPWHDGGDIETGHLAKWKQGKTGFPFIDAVMRQLLQIG

QFVLAIGCGFQVLLLNNFWIVHIVSVLSIMDVVLTHGEIIRYVPELNNYPVEYLYEPWKAPIEVQEKAGCVVGRDYPERI

VDHLKASEQNRADMNKIILGNIYSYRENRVILL

>Blattella_ger_PSN38921.1_C0J52_1186 PSN38921.1

MASKTKKMKLDMPASSSSQLSKTDFFLKLKEDRTTASESVMEFKFNKKRVRVLSEAAEVPERAEDNWAMLYAQKLALKNE

VPLHVSFCLVPKFMDATIRHYQFMLKGLQEVAEECKDLNIQFHLLLGQAVNILPKFVMQHKMGAVVTDFAPLRVPTKWVK

DVQAALPKDVPFCQQAESILEVDRSVKAVTWATPGTKAGLEVLHSFCTSRLKLFASKRNDPNANALSNLSPWFHFGGYNY

LRKGLNTVVQVLEKGMYPLLSRIAALVAEAVRNSFQSASAIRTNSQFPGSTRTALRRLKDEKLFARIAANKELLKDEHKL

FRLAFSEENTNRNWNRLIFSDDVTFSSTNGGKVIVYRPRGAGYDERYVAKQIRRESVDAFCEEAIVRRELADNFCFYNEK

YDSLDGAYDWAKITLEAHRKDKRQYLYTREELEKAVTHDDLWNSAQLQLVQEGKMHGFLRMYWAKKILEWTESPKKALAD

AIYLNDRFSIDGRDPNGFVGCMWSICGIHDQGWAERPIFGKIRYMNYEGCKRKFDIAAYIIKWGGKKHKK

>Blattella_ger_PSN44595.1_cry-1 PSN44595.1

MENGIKTSVLPQARLEGLWLLDGFPFEICARFLVGILPIVPTTMFRRFGSEFAQTELASVCHKRTTCRCTPKTIQAPLTY

HQFQTVVASMGPPPPAEPSITQKSINGTYTPIGEDHDDKFGVPTLEELERKAWVASFGRPKMTPQSLLASQTGLSPYLRF

GCLSTRLFYYQLTDLYKKIKKACPPLSLHGQLLWREFFYCAATKNPKFDRMIGNPICVQIPWDKNAEALAKWANGQTGFP

WIDAIMTQLREEGWIHHLARHAVACFLTRGDLWISWEEGMKVFEELLLDADWSVNAGMWIRYLPVLKNFPTRYIHEPWNA

PESVQKAAKCIVGKEYSLPMVNHAVASRINIERMKQVYQQLSKYRGPGTEEESSAKGCTTVGLLATVPTSQSQSTNILNP

MLMHREFKKSPPLEPKWERSPPDNAMNGHSPTYGEDPTKGIQLNCYQLQQQQQQMQIQNPHHQKQENEECE

>Bombus_aff_CRY-1-_XP_050593075.1 XP_050593075.1

MTGSRSSEINPDVTVRGEGGKHTVHWFRKGLRLHDNPSLREGLTGATTFRCVFVLDPWFAGSTNVGINKWRFLLQCLEDL

DCSLRKLNSRLFVIRGQPADALPKLFKEWGTTNLTFEEDPEPFGRVRDHNISALCKELGISVVQKVSHTLYKLDEIIERN

GGKPPLTYHQFQNVVASMDPPEPSVSTVTSACIGSAYTPLKEDHDDHYGVPTLEELGFDTEGLLPPVWVGGESEALARLE

RHLERKAWVASFGRPKMTPQSLLPSQTGLSPYLRFGCLSTRLFYYQLTDLYKKIKKAVPPLSLHGQLLWREFFYCAATKN

PNFDRMQGNPICVQIPWDKNVEALAKWANGQTGFPWIDAIMTQLREEGWIHHLARHAVACFLTRGDLWISWEEGMKVFDE

LLLDADWSVNAGMWMWLSCSSFFQQFFHCYCPVRFGRKADPNGDYIRRYLPILKNFPTRYIHEPWNAPLSVQRAAKCIIG

KDYSLPMVNHSKSSRINIERMKQVYQQLNKYRGNGASLKGETVGLLNALPPPSVKENEEEKKRTKQSPPPPENQSKMEAL

AKTTQHQQQHHQHQ

>Bombus_aff_PL-lik_XP_050573135.1 XP_050573135.1

MDELNPSKRKKVVNLLKKFEENRKNVSESIMTFNFNKQRIQYLSKLNDVKEKCKGILYWMFRDIRTQDNWALLFAQKIAV

ESNVPLHICFCIMPSFLNASMRYYKFLLKGLMEIEKECKQLNLNFHLLHGEPNESILKFVKTYNMGAVIVDFYPLKLPMS

WIDNVQKNLPEDVPIYQIDAHNIVPCWYASSKQEFAAKTIRNKINTKLQEFLTEFPPVIKHPYLTKEKFENNNWDITLQD

VEASKPTAEITWAKPGYRNGIKELENFIQNHLQKYGDEHNNPLSNAISNLSPWFHFGMISVQRCILEIREYKGLYKKSVE

SFMEEAIIRRELSDNFCFYNEKYDLVEGAYPWAIKTLNKHRKDTRKYIYSLSQLENSKTHDDLWNACQNQMIITGKMHGF

LRMYWAKKLLEWTETPEIALEWANYLNNKYSIDGCDPNGYVGCMWSICGVHDHGWPERDIFGKIRYMNYNGCKRKFNVAE

FVTKWGKKKPNELI

>Bombus_hun_crypto_XP_050476749.1 XP_050476749.1

MTGSRSSEINPDVTVRGEGGKHTVHWFRKGLRLHDNPSLREGLTGATTFRCVFVLDPWFAGSTNVGINKWRFLLQCLEDL

DCSLRKLNSRLFVIRGQPADALPKLFKEWGTTNLTFEEDPEPFGRVRDHNISALCKELGISVVQKVSHTLYKLDEIIERN

GGKPPLTYHQFQNVVASMDPPEPSVPTVTSACIGSAYTPLKEDHDDHYGVPTLEELGFDTEGLLPPVWVGGESEALARLE

RHLERKAWVASFGRPKMTPQSLLPSQTGLSPYLRFGCLSTRLFYYQLTDLYKKIKKAVPPLSLHGQLLWREFFYCAATKN

PNFDRMQGNPICVQIPWDKNVEALAKWANGQTGFPWIDAIMTQLREEGWIHHLARHAVACFLTRGDLWISWEEGMKVFDE

LLLDADWSVNAGMWMWLSCSSFFQQFFHCYCPVRFGRKADPNGDYIRRYLPILKNFPTRYIHEPWNAPLSVQRAAKCIIG

KDYSLPMVNHSKSSRINIERMKQVYQQLNKYRGNGASLKGETVGLLNALPPPSVKENEEEKKKTKQSPPPPENQSKMEAL

AKTTQHQQQHHQHQ

>Bombus_hun_PL-lik_XP_050477119.1 XP_050477119.1

MTFNFNKQRIKHLSELNDVKENCKGILYWMFRDIRTQDNWALLFAQKIAVKRNVPLHICFCIMPSFLNASMRYYKFLLKG

LMEIEEECKQLNLNFHLLHGEPNESILKFVKTYNMGAVIVDFYPLKLPMLWIDNVQKNLPEDVPIYQIDAHNIVPCWYAS

SKQEFAAKTIRNKINTKLQEFLTEFPPVIKHPYLTKEKFENNNWDITLQDVEASKPTTEITWAKPGYRNGIKELENFIQN

HLQKYGDERNNPLSNAISNLSPWFHFGMISVQRCILEIQEYKGLYKKSVESFMEEAIIRRELSDNFCFYNEKYDLVEGAY

PWAIKTLNKHRKDTRKYVYSLSQLENSKTHDDLWNACQNQMIITGKMHGFLRMYWAKKLLEWTETPEIALEWANYLNNKY

SIDGCDPNGYVGCMWSICGVHDHGWPERDIFGKIRYMNYNGCKRKFNVAEFVMKWGKKKANELI

>Bombus_imp_CRY_2_NP_001267051.1 NP_001267051.1

MTGSRSSEINPDVTVRGEGGKHTVHWFRKGLRLHDNPSLREGLTGATTFRCVFVLDPWFAGSTNVGINKWRFLLQCLEDL

DCSLRKLNSRLFVIRGQPADALPKLFKEWGTTNLTFEEDPEPFGRVRDHNISALCKELGISVVQKVSHTLYKLDEIIERN

GGKPPLTYHQFQNVVASMDPPEPSVPTVTSACIGSAYTPLKEDHDDHYGVPTLEELGFDTEGLLPPVWVGGESEALARLE

RHLERKAWVASFGRPKMTPQSLLPSQTGLSPYLRFGCLSTRLFYYQLTDLYKKIKKAVPPLSLHGQLLWREFFYCAATKN

PNFDRMQGNPICVQIPWDKNVEALAKWANGQTGFPWIDAIMTQLREEGWIHHLARHAVACFLTRGDLWISWEEGMKVFDE

LLLDADWSVNAGMWMWLSCSSFFQQFFHCYCPVRFGRKADPNGDYIRRYLPILKNFPTRYIHEPWNAPLSVQRAAKCIIG

KDYSLPMVNHSKSSRINIERMKQVYQQLNKYRGNGASLKGETVGLLNALPPPSVKENEEEKKKTKQSPPPPENQSKMEVL

AKTTQHQQQHHQHQ

>Bombus_imp_PL_X1_XP_024223040.1 XP_024223040.1

MDELNPSKRKKVINLLKKFEENRKNVSESIMTFNFNKQRIKHLSELNDVKENCKGILYWMFRDIRTQDNWALLFAQKIAV

KRNVPLHICFCIMPSFLNASMRYYKFLLKGLMEIEEECKQLNLNFHLLHGEPNESILKFVKTYNMGAVIVDFYPLKLPML

WIDNVQKNLPEDIPIYQIDAHNIVPCWYASSKQEFAAKTIRNKINTKLQEFLTEFPPVIKHPYLTKEKFENNNWDITLQD

VEASKPTTEITWAKPGYRNGIKELENFIQNHLQKYGDERNNPLSNVISNLSPWFHFGMISVQRCILEIQEYKGLYKKSVE

SFMEEAIIRRELSDNFCFYNEKYDLVEGAYPWAIKTLNKHRKDTRKYVYSLSQLENSKTHDDLWNACQNQMIITGKMHGF

LRMYWAKKLLEWTEIPEIALEWANYLNNKYSIDGCDPNGYVGCMWSICGVHDHGWPERDIFGKIRYMNYNGCKRKFNVAE

FVMKWGKKKASELI

>Bombus_pyr_CRY-1-_XP_043592444.1 XP_043592444.1

MTGSRSSEINPDVTVRGEGGKHTVHWFRKGLRLHDNPSLREGLTGATTFRCVFVLDPWFAGSTNVGINKWRFLLQCLEDL

DCSLRKLNSRLFVIRGQPADALPKLFKEWGTTNLTFEEDPEPFGRVRDHNISALCKELGISVVQKVSHTLYKLDEIIERN

GGKPPLTYHQFQNVVASMDPPEPSVPTVTSACIGSAYTPLKEDHDDHYGVPTLEELGFDTEGLLPPVWVGGESEALARLE

RHLERKAWVASFGRPKMTPQSLLPSQTGLSPYLRFGCLSTRLFYYQLTDLYKKIKKAVPPLSLHGQLLWREFFYCAATKN

PNFDRMQGNPICVQIPWDKNVEALAKWANGQTGFPWIDAIMTQLREEGWIHHLARHAVACFLTRGDLWISWEEGMKVFDE

LLLDADWSVNAGMWMWLSCSSFFQQFFHCYCPVRFGRKADPNGDYIRRYLPILKNFPTRYIHEPWNAPLSVQRAAKCIIG

KDYSLPMVNHSKSSRINIERMKQVYQQLNKYRGNGASLKGETVGLLNALPPPSVKENEEEKKKTKQSPPPPENQSKMEAL

TKTTQHQQQHHQHQ

>Bombus_pyr_PL-lik_XP_043592676.1 XP_043592676.1

MDELNPSKRKKVVNLLKKFEENRKNVSESIMTFNFNKQRIQYLSKLNDVKENCKGILYWMFRDIRTQDNWALLFAQKIAV

KSNVPLHICFCIMPSFLNASMRYYKFLLKGLMEIEEECKQLNLNFHLLHGEPNESILKFVKTYNMGTVIVDFYPLKLPMS

WIDNVQKNLPEDVPICQVDAHNIVPCWYASSKQEFAAKTIRNKINTKLQEFLTEFPPVIKHPYLTKEKFENNNWDITLQD

VEASEPTAEITWAKPGYRNGIKELENFIQNHLQKYGDEHNNPLSNAISNLSPWFHFGMISVQRCILEIQEYKGLYKKSVE

SFMEEAIIRRELSDNFCFYNEKYDLVEGAYPWAIRTLNKHRKDTRKYVYSLSQLENSKTHDDLWNACQNQMIITGKMHGF

LRMYWAKKLLEWTETPEIALEWANYLNNKYSIDGCDPNGYVGCMWSICGVHDHGWPERDIFGKIRYMNYNGCKRKFNVAE

FVTKWGKKKSNELI

>Bombus_ter_CRY_1_QDE55754.1 QDE55754.1

MTGSRSSEINPDVTVRGEGGKHTVHWFRKGLRLHDNPSLREGLSGATTFRCVFVLDPWFAGSTNVGINKWRFLLQCLEDL

DCSLRKLNSRLFVIRGQPADALPKLFKEWGTTNLTFEEDPEPFGRVRDHNISALCKELGISVVQKVSHTLYKLDEIIERN

GGKPPLTYHQFQNVVASMDPPEPSVSTVTSACIGSAYTPLKEDHDDHYGVPTLEELGFDTEGLLPPVWVGGESEALARLE

RHLERKAWVASFGRPKMTPQSLLPSQTGLSPYLRFGCLSTRLFYYQLTDLYKKIKKAVPPLSLHGQLLWREFFYCAATKN

PNFDRMQGNPICVQIPWDKNVEALAKWANGQTGFPWIDAIMTQLREEGWIHHLARHAVACFLTRGDLWISWEEGMKVFDE

LLLDADWSVNAGMWMWLSCSSFFQQFFHCYCPVRFGRKADPNGDYIRRYLPILKNFPTRYIHEPWNAPLSVQRAAKCIIG

KDYSLPMVNHSKSSRINIERMKQVYQQLNKYRGNGASLKGETVGLLNALPPPSVKEKEEEKKRTKQSPPPPENQSKMEAL

AKTTQHQQQHHQHQ

>Bombus_ter_PL_X1_XP_048265560.1 XP_048265560.1

MDELNPSKRKKVVNLLKKFEENRKNVSESIMTFNFNKQRIQYLSKLNDVKEKCKGILYWMFRDIRTQDNWALLFAQKIAV

ESNVPLHICFCIMPSFLNASMRYYKFLLKGLMEIEKECKQLNLNFHLLHGEPNESILKFVKTYNMGAVIVDFYPLKLPMS

WIDNVQKNLPEDVPIYQIDAHNIVPCWYASSKQEFAAKTIRNKINTKLQEFLTEFPPVIKHPYLTKEKFENNNWDITLQD

VEASKPTAEITWAKPGYRNGIKELENFIQNHLQKYGDEHNNPLSNAISNLSPWFHFGMISVQRCILEIREYKGLYKKSVE

SFMEEAIIRRELSDNFCFYNEKYDLVEGAYPWAIKTLNKHRKDTRIYIYSLSQLENSKTHDDLWNACQNQMIITGKMHGF

LRMYWAKKLLEWTETPEIALEWANYLNNKYSIDGCDPNGYVGCMWSICGVHDHGWPERDIFGKIRYMNYNGCKRKFNVAE

FVTKWGKKKPNELI

>Bombus_van_nea_CRY-1-_XP_033193421.1 XP_033193421.1

MTGSRSSEINPDVTVRGEGGKHTVHWFRKGLRLHDNPSLREGLTGATTFRCVFVLDPWFAGSTNVGINKWRFLLQCLEDL

DCSLRKLNSRLFVIRGQPADALPKLFKEWGTTNLTFEEDPEPFGRVRDHNISALCKELGISVVQKVSHTLYKLDEIIERN

GGKPPLTYHQFQNVVASMDPPEPSVPTVTSACIGSAYTPLKEDHDDHYGVPTLEELGFDTEGLLPPVWVGGESEALARLE

RHLERKAWVASFGRPKMTPQSLLPSQTGLSPYLRFGCLSTRLFYYQLTDLYKKIKKAVPPLSLHGQLLWREFFYCAATKN

PNFDRMQGNPICVQIPWDKNVEALAKWANGQTGFPWIDAIMTQLREEGWIHHLARHAVACFLTRGDLWISWEEGMKVFDE

LLLDADWSVNAGMWMWLSCSSFFQQFFHCYCPVRFGRKADPNGDYIRRYLPILKNFPTRYIHEPWNAPLSVQRAAKCIIG

KDYSLPMVNHSKSSRINIERMKQVYQQLNKYRGNGASLKGETVGLLNALPPPSVKENEEEKKKTKQSPPPPENQSKMEAL

AKTTQHQQQHHQHQ

>Bombus_van_nea_PL-lik_XP_033198945.1 XP_033198945.1

MDELNPSKRKKVVNLLKKFEENRKNVSESIMTFNFNKQRIKHLSELNDVKENCKGILYWMFRDIRTQDNWALLFAQKIAV

KRNVPLHICFCIMPSFLNASMRYYKFLLKGLMEIEEECKQLNLNFHLLHGEPNESILKFVKTYNMGAVIVDFYPLKLPML

WIDNVQKNLPEDVPIYQIDAHNIVPCWYASSKQEFAAKTIRNKINTKLQEFLTEFPPVIKHPYLTKEKFENNNWDITLQD

VEASKPTTEITWAKPGYRNGIKELENFIQNHLQKYGDERNNPLSNAISNLSPWFHFGMISVQRCILEIQEYKGLYKKSVE

SFMEEAIIRRELSDNFCFYNEKYDLVEGAYPWAIKTLNKHRKDTRKYVYSLSQLENSKTHDDLWNACQNQMIITGKMHGF

LRMYWAKKLLEWTETPEIALEWANYLNNKYSIDGCDPNGYVGCMWSICGVHDHGWPERDIFGKIRYMNYNGCKRKFNVAE

FVMKWGKKKANELI

>Bombus_vos_crypto_XP_033351814.1 XP_033351814.1

MTGSRSSEINPDVTVRGEGGKHTVHWFRKGLRLHDNPSLREGLTGATTFRCVFVLDPWFAGSTNVGINKWRFLLQCLEDL

DCSLRKLNSRLFVIRGQPADALPKLFKEWGTTNLTFEEDPEPFGRVRDHNISALCKELGISVVQKVSHTLYKLDEIIERN

GGKPPLTYHQFQNVVASMDPPEPSVPTVTSACIGSAYTPLKEDHDDHYGVPTLEELGFDTEGLLPPVWVGGESEALARLE

RHLERKAWVASFGRPKMTPQSLLPSQTGLSPYLRFGCLSTRLFYYQLTDLYKKIKKAVPPLSLHGQLLWREFFYCAATKN

PNFDRMQGNPICVQIPWDKNVEALAKWANGQTGFPWIDAIMTQLREEGWIHHLARHAVACFLTRGDLWISWEEGMKVFDE

LLLDADWSVNAGMWMWLSCSSFFQQFFHCYCPVRFGRKADPNGDYIRRYLPILKNFPTRYIHEPWNAPLSVQRAAKCIIG

KDYSLPMVNHSKSSRINIERMKQVYQQLNKYRGNGASLKGETVGLLNALPPPSVKENEEEKKKTKQSPPPPENQSKMEAL

AKTTQHQQQHHQHQ

>Bombus_vos_PL-lik_XP_033343430.1 XP_033343430.1

MDELNPSKRKKVVNLLKKFEENRKNVSESIMTFNFNKQRIKHLSELNDVKENCKGILYWMFRDIRTQDNWALLFAQKIAV

KRNVPLHICFCIMPSFLNASMRYYKFLLKGLMEIEEECKQLNLNFHLLHGEPNESILKFVKTYNMGAVIVDFYPLKLPML

WIDNVQKNLPEDVPIYQIDAHNIVPCWYASSKQEFAAKTIRNKINTKLQEFLTEFPPVIKHPYLTKEKFENNNWDITLQD

VEASKPTTEITWAKPGYRNGIKELENFIQNHLQKYGDERNNPLSNAISNLSPWFHFGMISVQRCILEIQEYKGLYKKSVE

SFMEEAIIRRELSDNFCFYNEKYDLVEGAYPWAIKTLNKHRKDTRKYVYSLSQLENSKTHDDLWNACQNQMIITGKMHGF

LRMYWAKKLLEWSETPEIALEWANYLNNKYSIDGCDPNGYVGCMWSICGVHDHGWPERDIFGKIRYMNYNGCKRKFNVAE

FVMKWGKKKANELI

>Bombyx_man_CRY-1_XP_028033363.1 XP_028033363.1

MLGGSVLWFRHGLRLHDNPSLHSALEEKSGPFFPIFIFDGETAGTKVVGYNRMRYLLEALDDLDKQFKKYGGRLLLVKGK

PSAVFRRLWEEFGIRKLCFEQDCEPVWRPRDESVKTACREIGVTCREHVSHTLWEPDTVIKANGGIPPLTYQMFLHTVAT

IGDPPRPVDNAKLRGIKFGTLPLCFYEEFTVYDKVPNPEDLGVFLENEDIRMIRWVGGETAALKQMQHRLAVEYETFCRG

SYLPTHGSPDLLGPPISLSPALRFGCLSVRKFYWSLQDLFQQVHQGSLCSTQYITGQLIWREYFYTMSVNNPHYGQMTDN

PICLDIPWKSPEGDELERWASGRTGFPFVDAAMRQLRLEGWLHHAVRNTVASFLTRGTLWLSWEHGLAHFLKYLLDADWS

VCAGNWMWVSSSAFEALLDSGECACPVRLGQRLDPSGEYVRRYVPELARVPTEYIYEPWKAPLDVQERANCIIGKDYPAP

VVNHIVAAQRNRNAMEELRMLLEKAPPHCCPSSEDEIRQFMWLNE

>Bombyx_man_CRY-1_XP_028042985.1 XP_028042985.1

MSKTPTVIHWFRLDLRIHDNLALRNAINEAENRKHLLRPIYFLDPNIKDKVGINRLRFLLQSLEDLDNNLKKLNTCLYVL

RGKAVDLLPKLFDDWQVKYLTCQVDIDPEFVQQDEYIEDIAEKKGVFINKRVQHTVYDVHKVLRENNGAVPLTYQKFLSL

VKSINVKEPIEISNVLSSHCKPIDIQSENYSIPNLKELQIDEETLAPVKYHGGETEALKRLNLYMSKKEWVCKFEKPNSS

PNSIEPSTTVLSPYISHGCLSAKLFYHKLKEVENGRQHTLPPVSLMGQLMWREFYYTAGTGVANFDKMVGNAICIQIPWT

KNDAFLKAWAEGKTGYPFVDAIMRQLKQEGWIHHLARHMVACFLTRGDLWISWEEGAKIFEDYLLDYDWSLNAGNWMWLS

ASAFFYKYFRVYSPVAFGQKTDKEGVYIKKYVPELKKYPREYIYEPWKAPQSIQRNAGCIIGEHYPKRIVNHDTIHKENI

QKMSTAYKLNREKKAVKRPRS

>Bombyx_man_CRY-1-_XP_028037074.1 XP_028037074.1

MSAAPETLPPPSAQGHTPARPTHMSAPRRTPGKHTVHWFRKGLRIHDNPALREGIIDAVTFRCVFIIDPWFASSSNVGIN

KWRFLLQCLEDLDKSLKKLNSRLFVVRGQPADALPKLFREWGTTALTFEEDPEPYGRVRDHNIISKCREVGITVTSRVSH

TLYKLDKIIERNGGKAPLTYHQFQALIASMPPPPPAEVTITPQMLNGATTPITDNHDDRFGVPTLEELGFETEGLKPPIW

IGGESEALARLERHLERKAWVASFGRPKMTPQSLLASQTGLSPYLRFGCLSTRLFYYQLTELYKRVKRVRPPLSLHGQIL

WREFFYCAATRNPNFDRMEGNPICVQIPWEKNQDALAKWANGQTGYPWIDAIMIQLREEGWIHHLSRHAVACFLTRGDLW

ISWEEGMKVFDELLLDADWSVNAGMWMWLSCSSFFQQFFHCYCPVRFGRKTDPNGDFIRKYIPALKNMPTRYIHEPWMAP

ESVQAAAQCSIGRDYPMPMVDHTKASQINIERIKQVYAQLARYKPQATLNANAVQRPNVLQSSPSPTSIIASINQSNFLC

SQTPDPQNATSHSFKEPSDTFPINKKNKDTESNQTRYKRVIIVQKTRNSNVEINVSAPQDRRASDNYITINEGDPAHSTD

AKKQENNYDFKNLAIDNHIRGFNNIQNINSQYYLTNYSTKKKETEIKNETDEGPFVRPESYGTDRAYTINRCPSMSNEKE

FETPKENLKQ

>Bombyx_man_PL_XP_028029913.1 XP_028029913.1

MLQIPLVNQFNTVLKRYSVFKMASASKKRKTSIPMALGKSNESTSTIEEFLKKLQKKREETAESILNFDFNKKRIRIISQ

EQIIPDDCDGVVYWMSRDSRVQDNWAFLFAQKLALKNEVPLHVCFCLISKYLDASLRQFHFLVRGLEKVAAECKKLNIQF

HLLEGSGAEVLPQWVVDHNIGVVVCDFNPLRVPMGWLDGVKGKLRKDVPLIQVDTHNVVPCWVASDKQEYSARTIRNKIN

SKLDEFLTEFPPVIKHPYSSKFEAEPIDWHEAIETREADKSVGPVDWAKPGYDEAVKMMKSFLDKRLNIYASKRNDPTQD

ALSNLSPWFHFGQISVQRVALCVQEYKSKFTESVNAFLEEAIVRRELADNFCFYCEHYDSVKGASAWAQKTLDDHRKDKR

THIYTLEKLSKGETHDDLWNSAQLQLVKEGKMHGFLRMYWCKKILEWTPTPEDALKYGIYLNDHYSIDGRDPSGFVGCMW

SICGIHDQGWAERAVFGKIRYMNYDGCKRKFDIKAFIARYGGKVHKYIPKK

>Bombyx_mor_NP_001182627.1_cry_2 NP_001182627.1

MSAAPETLPPPSAQAHTPARPTHMSAPRRTPGKHTVHWFRKGLRIHDNPALREGIIDAVTFRCVFIIDPWFASSSNVGIN

KWRFLLQCLEDLDKSLKKLNSRLFVVRGQPADALPKLFREWGTTALTFEEDPEPYGRVRDHNIISKCREVGITVTSRVSH

TLYKLDKIIERNGGKAPLTYHQFQALIASMPPPPPAEVTITPQMLNGATTPITDNHDDRFGVPTLEELGFETEGLKPPIW

IGGESEALARLERHLERKAWVASFGRPKMTPQSLLASQTGLSPYLRFGCLSTRLFYYQLTELYKRVKRVRPPLSLHGQIL

WREFFYCAATRNPNFDRMEGNPICVQIPWEKNQDALAKWANGQTGYPWIDAIMIQLREEGWIHHLSRHAVACFLTRGDLW

ISWEEGMKVFDELLLDADWSVNAGMWMWFSCSSFFQQFFHCYCPVRFGRKTDPNGDFIRKYIPALKNMPTRYIHEPWMAP

ESVQAAAQCSIGRDYPMPMVDHTKASQINIERIKQVYAQLARYKPQATLNANAVQRPNVLQSSPSPTSIIASINQSNFLC

SQTPDPQNATSHSFKEPSDTFPINKKNKDTESNQTRYKRVIIVQKTRNSNVEINVSAPQDRRASDNYITINEGDPAHSTD

AKKQENNYDFKNLAIDNHIRGFNNIQNINSQYYLTNYSTKKKETEIKNETDEGPFVRPESYGTDRAYTINRCPSMSNEKE

FETPKENLKQ

>Bombyx_mor_NP_001182628.1_cry_1 NP_001182628.1

MLGGSVLWFRHGLRLHDNPSLHSALEETSGPFFPIFIFDGETAGTKVVGYNRMRYLLEALDDLDKQFKKYGGRLLLVKGK

PSAVFRRLWEEFGIRKLCFEQDCEPVWRPRDESVKTACREIGVTCREHVSHTLWEPDTVIKANGGIPPLTYQMFLHTVAT

IGDPPRPVDNAKLRGIKFGTLPLCFYEEFTVYDKVPNPEDLGVFLENEDIRMIRWVGGETAALKQMQHRLAVEYETFCRG

SYLPTHGSPDLLGPPISLSPALRFGCLSVRKFYWSLQDLFQQVHQGSLCSTQYITGQLIWREYFYTMSVNNPHYGQMTDN

PICLDIPWKSPEGDELERWASGRTGFPFVDAAMRQLRLEGWLHHAVRNTVASFLTRGTLWLSWEHGLAHFLKYLLDADWS

VCAGNWMWVSSSAFEALLDSGECACPVRLGQRLDPSGEYVRRYVPELARVPTEYIYEPWKAPLDVQERANCIIGKDYPAP

VVNHIVAAQRNRNAMEDTKMEKNHAKQRGRPLASSNSGRRWNSPLDYQQTGREKIY

>Bombyx_mor_XP_004930027.2_photolyase XP_004930027.2

MLHIPLVNQFNTVLKRYSVFIMASASKKRKTSIPMALGKSNESTSTIEEFLKKLQKKREETAESILNFDFNKKRIRIISQ

EQIIPDDCDGVVYWMSRDSRVQDNWAFLFAQKLALKNEVPLHVCFCLISKYLDASLRQFHFLVKGLEKVAAECKKLNIQF

HLLEGSGAEVLPQWVVDHNIGAVVCDFNPLRVPMGWLDGVKGKLRKDVPLIQVDTHNVVPCWVASDKQEYSARTIRNKIN

SKLDEFLTEFPPVIKHPYSSKFEAEPIDWHEAIETREADKSVGPVDWAKPGYDEAVKMMKSFLDKRLNIYASKRNDPTQD

ALSNLSPWFHFGQISVQRVALCVQEYKSKFTESVNAFLEEAIVRRELADNFCFYCEHYDSVKGASAWAQKTLDDHRKDKR

THIYTLEKLSKGETHDDLWNSAQLQLVKEGKMHGFLRMYWCKKILEWTPTPEDALKYGIYLNDHYSIDGRDPSGFVGCMW

SICGIHDQGWAERAVFGKIRYMNYDGCKRKFDIKAFIARYGGKVHKYVPKK

>Bombyx_mor_XP_037874830.1_cry-1 XP_037874830.1

MSKTPTVIHWFRLDLRIHDNLALRNAINEAENRKHLLRPIYFLDPNIKDKVGINRLRFLLQSLEDLDNNLKKLNTCLYVL

RGKAVDLLPKLFDDWQVKYLTCQVDIDPEFVQQDEYIEDIAEKKGVFINKRVQHTVYDVHKVLRENNGAVPLTYQKFLSL

VKSINVKEPIEISNVLSSHCKPIDIQSENYSIPNLKELQIDEETLAPVKYHGGETEALKRLNLYMSKKEWVCKFEKPNSS

PNSIEPSTTVLSPYISHGCLSAKLFYYKLKEVENGRQHTLPPVSLMGQLMWREFYYTAGTGVANFDKMVGNAICIQIPWT

KNDAFLKAWAEGKTGYPFVDAIMRQLKQEGWIHHLARHMVACFLTRGDLWISWEEGAKIFEDYLLDYDWSLNAGNWMWLS

ASAFFYKYFRVYSPVAFGQKTDKEGVYIKKYVPELKKYPRE

>Bradysia_cop_XP_037034936.1_cry_DASH-l XP_037034936.1

MGKFKNCLVWLRNDLRFHDNEALLWAHENAETITPLFCFDPRMFATTHRYQFPKTGNFRSKFLIECVDDLNRTIKAKGSG

LIILSKRPEEALAFIRGKCKFEAIAFQKEITKEEINVERECENFCLQNNITFQAIWGSTLYHKDDLPYDVKSTPDTYTQF

RKDVETRSKVRTEKRMPEKLKPLPAELVGISEKIPLLQDLKLEEPNLHANSAFPFNGGETAALHRLKSYLWDTQAVEKYK

ITRNGLLGSEYSTKLSPWLAFGCISPRRIFYEIRKYETERVKNDSTYWVIFELLWRDYFRFVAVKYGNSLFYSGGIKKMK

YKWTEDMEQFKKWADGKTGVPFVDANMRELLQTGWMSNRGRQNVASFLTKDLKLDWRLGAEWFEYLLIDYDVCSNYGNWN

YSAGIGNDPRADRKFNMIKQALDYDAEGEFIKLWIPELVNIHKTGIHAPWTLRQGELGGVKLGIDYPNPMVTAQEWSRHL

HKSKSSDWAAASKSKQKGHDFYFKPNTSRK

>Bradysia_cop_XP_037040970.1_cry1-like XP_037040970.1

MTRSKFNSQLSGEKHTVHWFRKGLRLHDNPAIIEGLTGSKTLRCIFIIDPWFAGISNAGINKWRFLLQCLEDLDQNLRKL

NSRLFVIRGQPADALPKLFKQWGTTCLTFEEDPEPFGKVRDDNIAALCKESNISVIKTVSHTLYDLETIIELNGGEAPLT

YNQFQAIVASMEQPAAAVPTVTLEHLSTVFTPISEDHDEKYGVPTLYELGFETDNLQPPVWIGGETEALIRLERHLERKA

WVASFGRPKMTPQSLLASQTGLSPYLRFGCLSTRLFYHQLSDLYKKIKKACPPLSLHGQLLWREFFYCVATKNSNFDRMQ

NNPICVQIPWQKNPDALVKWAMGFTGFPWIDAIMTQLRNEGWIHHLARHAVACFLTRGDLWISWEEGMKVFDELLLDADW

SVNAGQWMWLSGSSFFQQIFHCYCPVRFGRKADPNGDYIRKYLPCLKTFPLRYIHEPWSAPEDVQRVAKCIIGQDYPLPI

VNHIRASRINMDRMRQAYQQLSKFRNANNTNNKRGKQLSVNLNTSNDNVPAVQQLDKGFLHPQRISIPSDSTYRQSYAHQ

LHQFKPELFAENVNYNSKRENSHTTVLAYSYNRQTHNDDKFENFDFSASSLNANASNYVDDDQKDGGHSKHRKCELNENF

DSFMEAENENDPTRMT

>Bradysia_cop_XP_037041975.1_cry1-like XP_037041975.1

MDKQQKTIIHWFRKGLRIHDNPALAEAIDLIRKSPNKYVLRPIFVLDPEIPNWLKVGPNRWRFLQESLLQLDSNLRKIKS

RLYVVRGDPKVIFPKLFNEWQVELLTFESDIEPYARQRDMLVEKQAKKFNVNVITTVSHTIYNPELIIQKSQGIAPKQYE

GFLKIVSTMSIPEPLEAPAALPAKSKPSKDSNEHTDSKCYDCPALHELGVNEADLGDVLYPGGETEGLKRFQAILRKPDW

ICGFEKPKTSPNSLKPSTTVLSPYLKFGCISSRLMYSDLQKVLKNKKKHTTPPVSLIGQLLWREFFYTAAASDPNFNRMV

GNSFCRQIPWKKNVQHLEAWTYGRTGYPFIDAIMRQLRQEGWIHHLARHAVACFLTRGDLWISWEDGQRVFEELLLDADW

ALNAGNWMWLSASAFYHLYYRVYSPVSFGKKTDKDGEYIRKYVPELANYPKSMIYEPWTATAAQQREFGCIIGKDYPQRI

VIHDVVLKKNMAKMADAYQKHRDGGGGGKFDVDNTQAKPEPQSPKTEPVSPSTSSGCSSSKILKMEQNSTIVKYFKKDPK

SP

>Bradysia_cop_XP_037043249.1_deoxyribod XP_037043249.1

MRHLNEIRLIVVYAKHLKINLSMSSSSISPPPKKRNKSNNPSTSSEVSTEVDSKGRPKSVNFKEQFKNARIQTAESVLDF

KFIKSRVRILSANGNVPESARGIVYWMSRDARVQDNWAFLFAQKLALKNEVPLHICFCLVPKFLDATIRHYKFLLNGLKE

VEAECSALNISFHLLIGTAATTIPKFVKDNGIGAVVCDFSPLRVPVQWVDDVLHKLPKTVPLVQVDAHNIVPVWETSDKQ

EYAARTIRGKINKKLDNFLTDFPPVIRHPYESDAKKIKIDWQTAFDSLEVDMTVDEVDWAKPGYSHGVEEMEQFCNHRLK

IFNDKRNDPLANALSNLSPWFHFGQISVQRTILTVKKYKKLYPSSVDAYVEEAVVRRELSDNFCFFNKNYDSINGLSGWA

KQTLIDHKKDKREWIYTRKQLDSSQTHDDLWNSAQIQLRLEGKMHGFLRMYWAKKILEWTNSPEEALEIAIYLNDRYSLD

GRDPSGYVGCMWSIGGVHDMGWKERAIFGKIRYMNYKGCEKKFDVKAFVSRYGGKVHSKGKKK

>Bradysia_odo_hypoth_KAG4065062.1 KAG4065062.1

MTTHLVWLRNDLRITDNKALSAACSDPQAKVLAVFIATPAQWRQHHMAPRQAAFIHANLLQVQAALAARGIELICHQGDD

FAGSRLAESDVSSLPAPRPRGEAVGFTAAAAFDYPLAEVGEGYPAGEEAALHRLRAFCREQVQDYAAQRDLPAIAGTSNL

SPYLAIGVISPRQCVNRLRVECPDVLEDPQSGAFCWLNELIWREFYRHLMVAYPALCKHRPFIAWTDKVCWQRNDAALLA

WQQGNTGYPIVDAAMRQLNQTGWMHNRLRMIVASFLVKDLLIDWRAGERYFMSQLLDGDLAANNGGWQWAASTGTDAAPY

FRIFNPTTQGERFDPQGTFIRKWLPELANVPDNELHQPHRWAEKQQQVLSYPPPIVQHQQARLETLAAFEAAKRGGL

>Bradysia_odo_hypoth_KAG4065954.1 KAG4065954.1

MTRSKFNSQFSGEKHTVHWFRKGLRLHDNPAIIEGLTGSKTLRCIFIIDPWFAGISNAGINKWRFLLQCLEDLDQNLRKL

NSRLFVIRGQPADALPKLFRQWGTTCLTFEEDPEPFGKVRDDNIAALCKESNITVIKTVSHTLYDLETIIELNGGEAPLT

YNQFQAIVASMEQPAAAVPTVTLEHLQTVFTPISEDHDEKYGVPTLYELGFETDNLQPPVWIGGETEALIRLERHLERKA

WVASFGRPKMTPQSLLASQTGLSPMQNNPICVQIPWQKNPDALVKWAMGFTGFPWIDAIMTQLRNEGWIHHLARHAVACF

LTRGDLWISWEEGMKVFDELLLDADWSVNAGQWMWLSGSSFFQQIFHCYCPVRFGRKADPNGDYIRKYLPCLRTFPLRYI

HEPWSAPEDVQRVAKCIIGQDYPLPIVNHIRASRINMDRMRQAYQQLSKYRNANDKSGKQLSTNVNASHDNVPAGQQFDK

GFLQPQRISIPSDSTYNQSYAHQLHQYKPELFAENINYNSKRDNTHTTVLAYSYNRQTHYDDKFENFDFSTSSLNANASN

YVDDDQKDVGHSKHRKCEINENFDSYMEAENENDPNRMT

>Bradysia_odo_hypoth_KAG4066065.1 KAG4066065.1

MLVEKQAKKFNVNVITTVSHTIYNPDLIIQKSQGVAPKQYQGFLKIVSSMSIPEPVEAPAALPAKSKPPKDSNELKDPTC

YDCPALHELRVNEAELGDVLYPGGETEGLKRFQTILRKTDWVCSFEKPKTSPNSLEPSTTVLSPYLKFGCISSRLMYTDL

QKVLKNKKKHTAPPVSLIGQLLWREFFYTAAASEPNFNRMVGNSICRQIPWQKNEQHLEAWTYGRTGYPFIDAIMRQLRQ

EGWIHHLARHAVACFLTRGDLWISWEDGQRVFEELLLDADWALNAGNWMWLSASAFYHLYSRVYSPVAFGKKTDKDGEYI

RKYVPELANYPKSMIYEPWTATAAQQREYGCIIGKDYPQRIVIHEVVLKKNMAKMADAYQKHRDGVGGGKFDVDNTQVKP

EPVSPKTEPVSPSTSSGCSSSKIMKVEQNNTILKYFKNEPKSP

>Bradysia_odo_hypoth_KAG4067580.1 KAG4067580.1

MSSSSTSPPPKKRNKSNNPSTSSEVSAEVDSKEGPKSVNFKEQFKNARIQTAKSVLDFKFNKSRVRILSANGNVPESAKG

IVYWMSRDARVQDNWAFLFAQKLALKNEVPLHICFSLVPKFLEATIRHYKFLLNGLKEVEGECSALNISFHLLIGTAATT

IPKFVKDNGIGAVVCDFSPLRVPAQWVDDVLHKLPKLVPLVQVDAHNIVPVWEASDKQEYAARTIRGKINKKLDHFLTGF

PPVIRHPYDSDAKKVKIDWQTAFDSLEVDMTVDEVDWAKPGYSHGVEELEQFCNHRLKLFNEKRNDPLGNALSNLSPWFH

FGQISVQRTILTVKKYKNLHPKSVDAFVEEAVVRRELSDNFCFFNKNYDSISGLSDWARQTLMDHKKDKREWIYTSEQLD

SSQTHDDLWNSAQIQLRLEGKMHGFLRMYWAKKILEWTNTPEEALEIAIYLNDRYSLDGRDPNGYVGCMWSIGGIHDMGW

KERAIFGKIRYMNYKGCERKFDVKAFVARYGGKVHSKGKKK

>Bradysia_odo_hypoth_KAG4073242.1 KAG4073242.1

MMGVEALLWAHENAETITPLFCFDPRMFTTTHRYQFPKTGNFRSKFLIECVDDLNRTIKAKGSGLIILYKQPAEALAIIR

DKCKFEAIAFQKEITKEEIDVERECETFCLQNKITFQAIWGSTLYHKDDLPYDVKSTPDTYTQFRKDVETRGKIRTEKRM

PEKLKPLPAELVGISERIPLLQNLKLEGKLPCFTIGDGSAFPFTGGETAALHRLKSYLWDTQAVEKYKITRNGLLGSEYS

TKLSPWLAFGCISPRRIFYEIRKYETERVKNDSTYWVIFELLWRDYFRFVAAKYKNSLFYSGGVKKLKYKWNEDMEQFRK

WADGKTGVPFVDANMRELLQTGWMSNRGRQNVASFLTKDLKLDWRMGAEWFEYLLIDYDVCSNYGNWNYSAGIGNDPRED

RKFNMIKQALDYDPEGEFTRLWVPELVNIHKTGIHAPWTLRQGELGGLKLGIDYPNPMVIAQEWSRHLHKSKSSDWAAAS

KSKQKGHDFYFKSNTSRK

>Brassicogethes_aen_unname_CAH0557099.1 CAH0557099.1

MSGVAGSGCRTAKGGQQQQDKHMVHWFRKGLRLHDNPSLREGLRGAKTFRCVFVLDPWFAGSSNVGINKWRFLLQCLEDL

DRSLRKLNSRLFVIRGQPADALPKLFKEWGTTSLTFEEDPEPFGRVRDHNITTLCQELGITVVQRVSHTLYHLQHISDKN

GGKAPLTYHQFLAVIACVDAPPKPELPVTASSLSGAHTPLSEDHDERYGVPTLEELGFDTDGLNPPVWQGGESEALARLE

RHLERKAWVASFGRPKMTPQSLLPSQTGLSPYLRFGCLSTRLFYYQLTDLYKKIKKAYPPLSLHGQLLWREFFYCAATKN

PNFDKMLGNPICVQIPWDKNAEALAKWANGQTGFPWIDAIMAQLRQEGWIHHLARHAVACFLTRGDLWLSWEEGMKVFEE

LLLDADWSVNAGMWMWLSCSSFFQQFFHCYCPIKFGRKADPNGDYIRKYLPILKNVPIQYIHEPWTAPENVQRAAKCVIG

RDYPMPMVNHSSASRVNIQRMKQVYQQLANYRNVNCSSGQGHFKEGYQDQLAMANLYDIEDAKYLKSTQQADHFEDNFNM

I

>Brassicogethes_aen_unname_CAH0562362.1 CAH0562362.1

MASLKPKNASGAVLLSKLTKDVFIKNISEARAAEGDIEHFCFNKSRLRVVSKTDDLKKNSKGIIYLMSRDCRVEDNWAVL

YSQKLALQNKLPLHICVLTKEFNCLYPTKRHLHFALEGFKEIQKECEVKKIGFHFLKANVKDFVKIVEDNQIGAVVLDFD

PLKKPVEWRNYLKDNLDKNVALIEVDAHNIVPAWLASEKQEVMAKTIRPKITKQLPEYLTGFPEVCKHNYQGKLDTDKKL

KNVDDVYDFYKPLYDVEIVKWAKPGAEGAYNTLLSFMQERLQYYGISSNDPSKNHTSNLSPWIRFGHIAAQRVALEVKSV

EKNCKEQVDKYLEELIVRRELAENYCFYNSNYDNINGAAEWARKTLLDHKSDKRVYTYTRDQFEKALTHDEMWNAATLQL

VQEGKIHGYMRMYWCKKILEWTKTPEEAIEIGLWLNDTFSLDGTCPNGFVGVMWSICCVHDQGWGEREIFGKIRFMVDYS

LKRKFNMEAYCARFGRSIGKTSAKKGVKRKAK

>Brenthis_ino_unname_CAH0722552.1 CAH0722552.1

MKVIKGLRLHDNPALREGLKDAKTFRCVFIIDPWFASSSNVGINKWRFLLQCLEDLDNSLRKLNSRLFVVRGQPADALPK

LFQEWGTTALTFEEDPEPYGRVRDHNIMTKCQEVGITVTSRISHTLYKLDNIIERNGGKAPLTYHQFQALIASMPPPPPA

EAAICAQSLNGAVTPVADDHDDRFGVPTLEELGFETEGLKPPIWIGGENEALLRLDRHLERKAWVASFGRPKMTPQSLLA

SQTGLSPYLRFGCLSTRLFYYQLTDLYKRVKRVRPPLSLHGQILWREFFYCAATRNPNFDKMEGNPICVQIPWEKNQDAL

SKWANAQTGYPWIDAIMTQLREEGWIHHLARHAVACFLTRGDLWISWEEGMKVFDELLLDADWSVNAGMWMWLSCSSFFQ

QFFHCYCPVRFGRKTDPNGDFIRKYIPALKNLPTRYVHEPWVAPEAVQQAAGCIVGRDYPMPIVDHSKASQINIERIKLV

YAQLAKFKPQGLPGAFIPHIMQRPNVMQSSPNPTSIITNINQSNYLCSQAPEPPAPSTPTSQFKHDSLFVRPTKINMRSN

FDSNKFKKIVIIQQEKISLQSTVENNFIVHGDTNSAYKMNDNRVKPGNYDFKNLAIDNNYISKFSNDQVNFLNQKTTNNA

VYNTDVKIDEYSTHKPKFYFTDNGVISHNDSDQTFASNHYATDYHRERNLQLKSDDNPKVYNSNPSKSDNKQSSGDRK

>Brenthis_ino_unname_CAH0723139.1 CAH0723139.1

MHLMLHINITLKTTSYPATGASPLFFSLQEKANMLGGSVLWFRHGLRLHDNPSLQSALEERSLPFFPVFIFDGETAGTKV

VGYNRMRYLLEALNDLDAQFKKYGGKLFMIKGKPDVVFKRLWEEFGIRKLCFEQDCEPIWRQRDEGVRTMCREIGVSCLE

HVSHTLWDPDTVIRTNGGIPPLTYQMFLHTVAIIGDPPRPVDDVDLKGVNFGSLSESFYKEFTIFDQAPKPEDLGVFLEN

EDIRMIRWVGGETAALHQMQQRLAVEYETFCRGSYLPTHGNPDLLGPPISLSPALRFGCLSVRRFYWCVQDLFHQVNQGQ

LASTQFITGQLIWREYFYTMSVNNPSYGQMNGNPICLSIPWKEPEGDELQSTRVSRGRWREGRTGFPFIDAAMRQLRTEG

WLHHAARNTVASFLTRGTLWLSWEHGLDHFLKYLLDADWSVCAGNWMWVSSSAFEALLDSGECACPVRLGRRLEPSGRYV

RRYVPELAHMPDQYIYEPWNAPIEVQERAGCVVGRDYPAPVVDHRAAAHRNRAAMQELRRVLEKAPPHCCPSSEDEIRQF

MWLGDENQPEITTA

>Brenthis_ino_unname_CAH0727559.1 CAH0727559.1

MVPDKCEGIVYWMSRDSRVQDNWAFLFAQKLALKNEVPLHVCFCIIAKYLDASVRQFHFLLKGLEKVSAECKKLNISFHL

LEGSGADALPQWVIDHKIGAVVCDFNPLRVPLGWLEGVKKKLKKDVPLIQVDAHNVVPCWVASDKQEYSARTIRNKINSK

LDEFLTEFPPVIKHPYISKFEPEPIDWDEAIDSREADKSVGPIEWATPGYDSAMKVLKGFLDKRLKIFASKRNDPTQDAL

SNLSPWYHFGQISVQRVALCVKEFKKTHTESVNAYLEEAIVRRELADNFCFYCEHYDSIKGASAWAQKTLDDHRKDKRSH

IYTLQQLSEAKTHDDLWNSAQIQLVKEGKMHGFLRMYWCKKILEWTQTPEQALKFAIYLNDHYSVDGRDPNGYVGCMWSI

CGIHDQGWAERAVFGKIRYMNYDGCKRKFPVPAFVARYGGKVHKYVPN

>Brenthis_ino_unname_CAH0728972.1 CAH0728972.1

MSKIPSVVHWFRLDLRLHDNLALRNAINEAENRKHILRPIYVIDPEINNKVGANRLRFLIQSLQDLDLNLRKINTRLFII

KGKATECLSRLFEKWQVNFLTLQVDIDVELVKQDEVIEQICEERNIFIVKRAQHTVYDFNSVLKKNNGNIPLTYQKFLSL

VADTQVKETIEITKNVIEECKSNDFSSDEYNVPSLDEVGLNHSDLSECKYPGGETEGLKRLNVYMAKKQWVCNFEKPNSS

PNSIEPSTTVLSPYISHGCLSAKLFYHKLKQVETGMKHTLPPVSLMGQLLWREFYYTAGAGTKNFDKMVGNSVCTQIPWG

KNEEHLKAWAEGRTGYPFVDAIMRQLKQEGWIHHLARHMVACFLTRGDLWISWEEGAKIFEDYLLDYDWSLNAGNWMWLS

ASAFFYKYYRVYSPIAFGKKTDKEGLYIRKYVPELKKYPSEFIYEPWKAPKSVQRTAGCVVGEGYPNRIVDHDKVHKENI

QKMNAAYKINKEKKALKRKR

>Calliphora_vic_CRY__AGX29490.1 AGX29490.1

SDKDEGIELLPIFIFDGESAGTKSVGFNRLKFLLDSLKDIHDQLQNLSLSLGRLYLLQGNPVQIFRRLHEQCGIKKLCFE

QDCEPIWNRRDNAVKELCHDLGITCLERISHTLWDPKKVIDTNGGIPP

>Callosobruchus_mac_unname_VEN42213.1 VEN42213.1

MSGMGTIEGQQFSSDDQGCKWMGNVKEKHTVHWFRKGLRLHDNPSLKEGLKGAKTFRCVFVLDPWFAGSSNVGINKWRFL

LQCLEDLDLSLRKLNSRLFVIRGQPADTLPKLFKEWGTTSLTFEEDPEPYGRVRDHNIIAMCKELGISVVQRVSHTLYHL

QNIIDHNGGQAPLTYHQFLAVVARIGPPPQPELPVNAGTIEGAHTPITEDHDDVYGVPTLEELGFDTEGLQAPVWQGGES

ESLARLERHLERKAWVASFGRPKMTPQSLLPSQTGLSPYLRFGCLSTRLFYYQLTDLYKKIKKAFPPLSLHGQLLWREFF

YCAATKNPNFDKMVGNPICVQIPWDKNAEALAKWANGQTGFPWIDAIMTQLRQEGWIHHLARHAVACFLTRGDLFISWEE

GMKVFEELLLDADWSVNAGMWMWLSCSSFFQQFFHCYCPVKFGRKADPNGDYIRRYLPVLKNMPVEYIHEPWLAPENVQR

ATKCVIGKDYPLPMVNHATASRINLQRMKQVYQQLANYKLVEDSKGNENYKDCYQQRRRSTNILQNSTAGNHPAS

>Callosobruchus_mac_unname_VEN43005.1 VEN43005.1

MAALKPRNSDSKIILSQLTKDLFLKNIDDARKAQGEHLESYDFNKSRCKVLSKAETVKNKSSGILYWMLRDCRVQDNWAM

IFAQRLALKQNLPLYVCYLFKDADKICPTVRHLTFLIEGLKLVEKECKDLNIGFYMLNSSVEELADVIEKNNIGGVVCDF

YPLKHPVELQKRLLNKLAEDVPVVQIDAHNIVPAWIASDKQEGMAKFLRPKINKNLPEYLCGFPKVSKHKYSGILKNLSG

HFKDLNEAYKCFKPKWDVEVVKWGEGPGEEGGLSMLRTFMVERLKYYGVTSNDPSKDNTSKLSPWLRFGQISAQRCALEV

KSLESLYKEQCERYLEELIVRRELTDNYCYYNQNYDNINGAANWAKETLKLHAKDKRTWTYTREQLENAQTHDEMWNAAQ

LQANTEGKINGYMRMYWAKKILEWTESPEQAIEYALWLNDTFCLDGTDPNGYVGVMWSICGVHDQGWRERDIFGKIRYMV

DYSLRQKFDMEAYCARYGTKMNVEKKKTKAVPKKGIKRKAV

>Camponotus_flo_EFN74495.1_cry-1 EFN74495.1

MTGSSNNEMVQGVASGVRGDGRKHTVHWFRKGLRLHDNPSLKEGLAGASTFRCVFVLDPWFAGSTNVGINKWRFLLQCLE

DLDCSLRKLNSRLFVIRGQPADALPKLFKEWGTTNLTFEEDSEPFGRVRDHNISALCKELGISVVQKVSHTLYKLDEIIE

RNNGKPPLTYHQFQNIVASMNPPEPPVSTVTAACIGNAYTPLKDDHDDHYGVPTLEELGFDTEGLLPPVWVGGESEALAR

LERHLERKAWVASFGRPKMTPQSLLPSQTGLSPYLRFGCLSTRLFYYQLTDLYKKVQDDCNPIVIKKAVPPLSLHGQLLW

REFFYCAATKNSNFDRMQGNPICVQIPWDKNVEALAKWANGQTGFPWIDAIMTQLREEGWIHHLARHAVACFLTRGDLWI

SWEEGMKVFDELLLDADWSVNAGMWMWLSCSSFFQQFFHCYCPVRFGRKADPNGDYIRRYLPVLKNFPTRYIHEPWNAPL

SIQHAAKCVIGKEYSLPMVNHNKTSRINIERMKQVYQQLNKYRGE

>Cataglyphis_his_CRY-1-_XP_050456296.1 XP_050456296.1

MTGSTNNEMGQGVTSGVLGDGRKHTVHWFRRGLRLHDNPSLREGLAGASTFRCVFVLDPWFAGSTNVGINKWRFLLQCLE

DLDCSLRKLNSRLFVIRGQPADALPKLFKEWGTTDLTFEEDPEPFGRVRDHNISALCKELGISVVQKVSHTLYKLDEIIE

RNNGKPPLTYHQFQNIVAGMDPPEPPVPTVTAACIGSAYTPLKDDHDDHYGVPTLEELGFDTEGLLPPVWVGGESEALAR

LERHLERKAWVASFGRPKMTPQSLLPSQTGLSPYLRFGCLSTRLFYYQLTDLYKKIKKAVPPLSLHGQLLWREFFYCAAT

KNPNFDRMQGNPICVQIPWDKNVEALAKWANGQTGFPWIDAIMTQLREEGWIHHLARHAVACFLTRGDLWISWEEGMKVF

DELLLDADWSVNAGMWMWLSCSSFFQQFFHCYCPVRFGRKADPNGDYIRRYLPVLKNFPTRYIHEPWNAPLSIQHAAKCI

IGKEYSLPMVNHNKTSRINIERMKQVYQQLNKYRDNGASFKGEKIGLLNALLAPPTKDADEEKRKQDSPNRGNEEKMETI

SNSAQQQQQQQQQQ

>Cephus_cin_CRY-1__XP_024937119.1 XP_024937119.1

MTGSRKIYDEGSQMQDDGRKHTVHWFRKGLRLHDNPSLQEGLVGASTFRCIFVLDPWFAGSTNVGINKWRFLLQCLEDLD

SSLRKLNSRLFVIRGQPADALPKLFKEWGTTNLTFEEDPEPFGRVRDHNISALCKELGISVVQKVSHTLYKLDQIIEKNG

GKPPLTYHQFQNVVASMDPPQSPVVTVTSGCVGNAYTPLKDDHDEIYGVPTLEELGFDTEGLLPPVWVGGESEALARLER

HLERKAWVASFGRPKMTPQSLLPSQTGLSPYLRFGCLSTRLFYYQLTDLYKKIKKTVPPLSLHGQLLWREFFYCAATKNP

NFDKMQGNPICVQIPWDRNVEALAKWANGQTGFPWIDAIMTQLREEGWIHHLARHAVACFLTRGDLWISWEEGMKVFDEL

LLDADWSVNAGMWMWLSCSSFFQQFFHCYCPVRFGRKADPNGDYIRRYLPILKNFPTRYIHDPWNAPLSVQRAAKCIIGR

EYSLPMVNHSKSSRINIERMKQVYQQLNKYRGNGTSLKGEGIGSLSIIPTDKVEVALSPEANRKMDVKDVRMQQ

>Cephus_cin_PL_X1_XP_015585787.1 XP_015585787.1

MEKESSSKKIKSSNLINQFIMERTNAADSIMHFRFDKKRVRILTSIEEVAEKCKGIVYWMFRDVRVQDNWAFLFAQKVAN

KNSVPLHVCFCILPTFLNSTMRHYKFLLKGLMEVDAECKQLNINFHLLHGEPNQAILDFVQKYKMGAVITDFFPLRLPLF

WLNDLKKKLPDNIPLCQVDAHNIVPCWITSEKLEYSARTIRNKINSRLEDYLTQFPPVTKHSFLTNQKFKKNDWENALRK

LDIEESVKEVPWTIPGYNGGILELQSFLDNRLAHFHTMRNNPLSNATSNLSPWFHFGMISVQRCILEVLKYKQMYKESVE

TFMEEAIVRRELSDNFCFNNEKYDAVEGANQWAIDTLNQHRSDKREYIYNLNELEHSLTHDDLWNSSQIQLVQEGKMHGF

LRMYWAKKILEWTHDPEEALKWAIYLNDKYSIDGCDPNGYVGCMWSICGIHDQGWKERNVFGKIRYMNYKGCERKFDVKE

FVKMWGGKVYNK

>Ceratina_cal_CRY-1__XP_017882053.1 XP_017882053.1

MKGGQNSEVNPEVAVRGDGGKHAVHWFRKGLRLHDNPSLREGLAGASTFRCVFVLDPWFAGSTNVGINKWRFLLQCLEDL

DCSLRKLNSRLFVIRGQPADALPKLFKEWGTTNLTFEEDPEPFGHVRDLNISALCKELGISVVQRVSHTLYKLDEIIERN

GGKPPLTYHQFQNIVASMDTPEPPVPTVTSACVGSAYTPLKEDHDDHYGVPTLEELGFDTEGLLPPVWVGGESEALARLE

RHLERKAWVASFGRPKMTPQSLLPSQTGLSPYLRFGCLSTRLFYYQLTDLYKKIKKAVPPLSLHGQLLWREFFYCAATKN

PNFDRMQGNPICVQIPWDKNVEALAKWANGQTGFPWIDAIMTQLREEGWIHHLARHAVACFLTRGDLWISWEEGMKVFDE

LLLDADWSVNAGMWMWLSCSSFFQQFFHCYCPVRFGRKADPNGDYIRRYLPVLKNFPTRYIHEPWNAPLSVQRTAKCIVG

KDYSLPMVNHSKSSRINIERMKQVYEQLNKYRGNGTSLKGETVGE

>Ceratina_cal_PL-lik_XP_017891920.1 XP_017891920.1

MDKSTPSWMSPPKRRKVRDLLKKFENNRKNTSESVMTYTFNKKRIRLLSNFNDVKENSNGILYWMFRDVRLQDNWAMLFA

QRTALKNSLPLHVCFCLMPNFLDASLRYYKFLLKGLMEVEEDCKELNINFHLLHGEPNESVLKFVKKYNMGAVIADFCPL

KLPMSWIDDVQKNLPKDVPICQVDAHNIVPCWHASSKQEIAARTIRNKINTKLDEYLTEFPPVVKHPHSTEGKFQSNNWE

TTLDNVEAGKPEDEIKWAEPGYKSSIRELEDFIETRLKRYAEQRNNPLSNVISNLSPWFHFGMLSVQRCILEIKEHRELY

KQSVDSFMEEAIVRKELSDNFCFYNDKYDLVEGAYPWAIETLNKHRKDKRKYIYSLSQLENCQTHDDLWNASQNQMVTMG

KMHGFLRMYWAKKILEWTESPEIALEWANYLNNKYSIDGCDPNGYVGCVWSICGIHDHGWGERDIFGKIRYMNYEGCKRK

FNVPEFVAKWKQKELDE

>Ceratitis_cap_CRY-1_XP_004531295.1 XP_004531295.1

MDTKTTLIHWFRKGLRVHDNPALNLIITKANSEPTKYYVRPIFILDAALIQWLRVGANRWRFLQQTLVDLDANLRKVNTQ

LYVVRGTPAAVFSRIIKDWRVSLLTYETDIEPYALKRDADVQRLAKEAGVKVETFCTHTIYNPELVIQRNGGSAPLTYQK

FLSVIEKIKVPPALGKLDSLIDVQPPPKDALELENANCYACPTIDELVKRPEELGANKFPGGETEALRRMNAALSDESWV

AAFEKPNTAPNSLDPSTTVLSPYLKFGCLSARLFHQRLMDILKRHPKHSKPPVSLHGQLLWREFYYTAAASETNFDRMLG

NKFCMQIPWEINEEHLTAWTHGRTGYPFIDAIMRQLRQEGWIHHLARHAVACFLTRGDLWISWEEGQKVFEELLLDQDWA

LNAGNWMWLSASAFFYQYFRVYSPVAFGKKTDPTGAYIRKYVPELAKYPAGCIYEPWKAALSAQREYGCVLGKDYPHRIV

KHEIVHKENIKRMTAAYKVNREVKEAKSPKKESSKGKRKQAGTSAQSTKKKQRT

>Ceratitis_cap_CRY-1__XP_004529289.1 XP_004529289.1

MAKRANVLWFRHGLRLHDNPALLEAIADKEEGIALIPIFIFDGESAGTKTVGYNRMRFLLNSLADIDSQLKAIPNNGHGL

GKLYLFQGNPAKIFRRLNEQYQLNKICFEQDCEPIWNRRDDSVRALCHELGIEAVEKVSHTLWDPRTVISTNGGIPPLTY

QMFLHTVEIIGPPPRPVEDPDWEGVEFLQLEESMLSKLNAFRNIPSPEDFDIVPENICYVAKVNWQGGERQALLHLAERL

KVEERAFKGGYYLPNQANPNIVESPKSMSAHLRFGCLSVRRFYWSVHDLFKNVQIEAFYHHIQMAGGEHITGQLIWREYF

YTMSVNNPFYDRMEENPICLNIPWAEPNSEHLERWRSGHTGFPIIDSAMRQLLAEGWLHHTLRNTVAMFLTRGALWQSWE

HGLQHFLKYLLDADWSVCAGNWMWVSSSAFERLLDTSLVSCPIAFSKRLDPKGEYIRQYVPELANVPQEYIHEPWRMSQE

LQEQCECVIGVQYPERIVDLAKVSKRNTLAMKALKQALIADGAPAEGPPHCRPSNAEEVHQFFWLVD

>Ceratitis_cap_PL_X1_XP_004535970.1 XP_004535970.1

MRRLRSFTDTKKYSLHKETESSSPEETNAKHVPTLKVIKKTTDGDISKESLCQKRLATAVSISEFPFNKKRVRVLSNESR

VSESLCGGILYWMYRDVRVQDNWAFLFAQRLALKFELPLLIAYIISPLDQVTTLRQYDFLLGGLEEVERECKDLLIPFHL

CVGDTAVCLPEFVRNHKIAAVVCDFTPLREQLQCVEQVKAALPVNVPFTQVDSHNIVPLWISSDKQEFVAYFMRRRINAR

LNEFLTEFPPVVEHPYNNKNTMNIKSIEWKQVRASLQCDHSVEPVKWLEPGYTMACAQLQSFCVERLAIYSAKRNDPTVN

ALSGLSPWLHFGQISAQRCILEVMRYQSQYKDSVEAFCDEAIVHRELADNFCYYNKNYDNLKGLYPWAIKTLEEHRTDER

NPTYAFTEFEGASTHDDLWNAAQMQLVHEGKMHGFLRMYWAKKILEWSSSPEVALKTAIHLNDKYSLDARDPNGYVGCMW

SIGGLHDHGYPNRPIFGKVRYMNYNGCQRKFDVKAFVAQYNPNCKTE

>Ceratitis_cap_PL_XP_004535731.1 XP_004535731.1

MKRIKNKSGNAIVTKKSKAESPVENDKDATSSVTTIEKVSITLQALQKRRLATAKSISEFPFNKKRVRVLSIVKEVPESR

PGGVLYWMSRDARVQDNWALLYAQRLSLKLKLPLFVCYCLVPKFLNATLRHYKFLLDGLKEVDQECRELSIPFKLFLGSP

LECIPEFVSSRKISAVVCDFSPLRVSQQWVEGIKNAIPRDVPFMQVDAHNLVPLWEASNKQEYGARTIRGKLNSKLAEFL

TEFPPVLRHAYNKEVMLNCTAINWKGAETSIQCDRSVDAVEWAQPGYKAACSQLESFCLNRLRIFSEKRNDPTVNALSGL

SPWFHFGQISVQRCVLEVMRFKPKYKASVESFCEEAIVRRELADNFCYYNEHYDNLKGLHAWAAKTLDDHRKDKRSPAYT

LAEFEQAHTHDDLWNASQLQLVREGKMHGFLRMYWAKKILEWSSTPEEALEVAILLNDKYSLDGRDPNGYVGCMWSIGGV

HDQGWAERPIFGKVRYMNYKGCQRKFDVATFVTRYGGTVYKAK

>Ceratosolen_sol_mar_PREDIC_XP_011496980.1 XP_011496980.1

MSTMIGSEINRDNNCNSDVDQQKIIERHTDKEFRMQDTNKKHTVHWFRKGLRLHDNPSLKEGLAGASTFRCVFVLDPWFA

GSTNVSINKWRFLLQCLEDLDQSLRKLNSRLFVIRGQPADALPKLFKEWGTTYLTFEEDPEPYGRVRDENITTLCKELGI

TVIQRVSHTLYKLGEIIQMNGGKSPLTYHQFQNVIAQMDAPEYPAMTVTANCIGSAYTPFKDDHDDFFGVPTLEELGFDT

EGLMAPVWVGGESEALARLERHLERKAWVASFGRPKMTPQSLLPSQTGLSPYLRFGCLSTRLFYYQLTDLYKKIKKTVPP

LSLHGQLLWREFFYCAATKNPNFDRMHGNPMCVQIPWDKNVEALAKWANGQTGFPWIDAIMTQLREEGWIHHLARHAVAC

FLTRGDLWISWEEGMKVFDELLLDADWSINAGMWMWLSCSSFFQQFFHCYCPVRFGRKADPNGDYIRRYLPVLKHFPTRY

IHEPWNAPMSTQRTAKCIIGQDYSLPMINHNKSSRINVERMKQVYQQLQVSIKGETIGLLSIVPIRSPLIYDQQDDEKNA

RTNFVEY

>Ceratosolen_sol_mar_PREDIC_XP_011505805.1 XP_011505805.1

TYKKLTIDLVKQIENQRKENGESILTFKFNKKRVRILTKLDEVTLHSKGIVYWMFRDARVHDNWALLFAQKIALKNRVPL

HVCFCVPPKFLDATLRHYKFLLGGLVEVEQDCRALNINFHLLYGEPNNMIFDFVNKYKMGAIIIDFFPLKLPLSWVDDLK

DKLSENIPICQVDAHNIVPCWIVSEKLEYAARTIRNKINSKLEEFLTEFPPVIKHPYVSNQKFDKTKWDKLLNNKLIDKS

VNEITWAKPGYEKGIEEFNNFIKNRLNFYNEKRNNPIYDAESKLSPWFHFGMISVQRCILEIVKYKSQYKKSVEDFMEEA

IIRRELSDNFCFYNSNYDNINGAFKWATITLNQHRNDMRDYLYTLDEFENGLTHDNLWNAAQMQLIKEGKMHGFLRMYWA

KKFLEWTEKPEDALKWSIYLNNKYSIDGQDPNGYVGCMWSICGVHDHGWKERPIFGKIRYMNYKGCERKFDVKAFIEKYT

TKAFTHKEKKK

>Ceutorhynchus_ass_unname_CAG9764033.1 CAG9764033.1

MASLKPRDFKSKLALSSLTKEQFLDEIIKSRKSQAESIENFAFNKSRCRVLSKTKDIASNSKGVLYWMIRDCRVQDNWAM

LFAQRLALRNKIPLMVCFSLFETHLLHPTRRHQQFLIDGLRQVEQELTGLNIPFYLIKESPKDLAKIVADNKLGGVVCDF

SPLRKPKEWIDILLKNLPGDVPLVQVDAHNIVPAWIASDKQEHMARTLRPKINKKLSEYLTGYPQVFKHPHTEKIQVKCL

NKLDNDEISNLIETLWEVEPVSNKAGSNEGLEALYKFLQNGLKHYGVTSNDPSKNHTSNLSYWITFGQISAQRIALETKS

LESIYKEQVEKYLEELIVRKELAENYCLYNKNYDSIEGAADWAKSSLILHKSDKRTYTYTREELEKSQTHDEMWNAAQLQ

ALKEGKIHGYMRMYWCKKILEWTKSPEEALEFALWLNDTFALDGNCPNGFVGVMWSICGVHDQGWREREIFGKIRFMVDY

SLRKKYDMDAYCARYGIGMKEGDKKWKKGTKRKLAK

>Ceutorhynchus_ass_unname_CAH1133232.1 CAH1133232.1

MSGGATIDTAALQEGKHAVHWFRKGLRLHDNPSLREGLKGATTFRCVFVLDPWFAGSSNVGINKWRFLLQCLEDLDRSLR

KLNSRLFVIRGQPADALPKLFREWGTTALTFEEDPEPFGRVRDHNITALCKELGITVVQKVSHTLYQLQHIIDRNGGKAP

LTYHQFLAVIACMGAPPYPEAPVTSNTVELAHTPLSEDHDELYGVPTLEELGFDTEGLNPPVWQGGETEALARLERHLER

KAWVASFGRPKMTPQSLLPSQTGLSPYLRFGCLSTRLFYYQLTDLYRKIKKAFPPLSLHGQLLWREFFYCAATKNPNFDK

MLGNPICVQIPWDKNAEALAKWANGQTGFPWIDAIMTQLRQEGWIHHLARHAVACFLTRGDLWISWEEGMKVFEELLLDA

DWSVNAGMWMWLSCSSFFQQFFHCYCPVKFGRKADPNGDYIRKYLPILKNIPTHYIHEPWMAPENIQKAAKCIVGNDYPL

PMVNHVSVSQVNIQRMKQVYQQLSNYRPHDSSATCSTNMKKGPYKENFHKELTLSYRN

>Chelonus_ins_CRY-1-_XP_034935205.1 XP_034935205.1

MTGSRHNDRGQGVPSCGSVNDGATISATNAPREKHTVHWFRKGLRLHDNPSLRAGLNGATTFRCVFVIDPWSAGSKSVGI

NKWRFLLHCLEDLDASLRKLNSRLFVIRGQPADVLPKIFREWGTTNLTFEEDPEPFGRARDHNIITLCKELGMSVVQMAS

HTLYKLDEILDRNGGKPPLTYHQFQNIIAGMDPPDLPVSTVTAECIGNAYTPLRDDHDDLYGVPTLEELGFDTEGLQPSV

WVGGESEALARLERHLGRKAWVASFGRPKMTPQSLLPSQTSLSPYLRFGCLSTRLFYYQLRDLYKKIKKATPPLSLHGQL

LWREFFYCAATKNPNFDRMQGNPICLQIPWDKNGEALAKWANGQTGFPWIDAIMTQLREEGWIHHLARHAVACFLTRGDL

WISWEEGMKVFDELLLDADWSVNAGMWMWLSCSSFFQQFFHCYCPVRFGRKADPNGDYIRRYLPVLKNFPTRYIHEPWVA

PLGIQRSAKCIIGRDYSLPMVNHSKSSRINIERMKQVYQQLSKYRGNGVIANGETIGLMRIIGHTSKICDDNENEEKKKK

DGSSPLPSEQKMIADSGFDTSASTPK

>Chelonus_ins_PL_XP_034943471.1 XP_034943471.1

MDSDEAPKKKLKMPSLFDKLIDKRKETADSILQFPFKKQRVRVLSKVDEVPVNAKGILYWMFRDGRVQDNWSFLFAQKLA

LKNKLPLHVCYCILPKFLDATLRHYKFLVESLEEVASDCKELNINFHLLHGVPNVVVYDLIKKHNMGALVVDFFPLRVPM

GWVDDLKNKLASSKIPICQVDAHNLVPCWVTSDKIEYGARTIRGKIKMNLEEYLTEFPPLIKHPYDSDFKIPTIDWSNAI

RDVEIDRTVDKVDWCKPGYRGALAELENFLNKRLPKYHTKRNDPTEDALSNLSPWFHFGQISVQRVILEVQEHKKKFKES

VENFMEEAIIRRELSDNFCFYNKNYDKLEGTNAWAIESLNKHRNDKRDYIYTRDEFEKSQTHDDLWNAAQNQLVKEGKIH

GFMRMYWAKKILEWTKTPEEALEWAIYLNDKYSMDGRDPNGYVGCMWSICGVHDQGWQERNVFGKIRYMNYKGCERKFDV

KAFVRKYDGKVINKKQNTLEGMFKKNKK

>Chilo_sup_AHL69753.1_cry_2 AHL69753.1

MSAAAQTLPSSTAARTHGPAALAPTPSKCNPTGKHTVHWFRKGLRLHDNPALREGLNNAVTFRCVFIIDPWFASSSNVGI

NKWRFLLQCLEDLDCSLRRLNSRLFVVRGQPADALPKLFREWGTTELTFEEDPEPYGRVRDHNIMSKCREIGINVVSRVS

HTLYKLDKIIERNGGKAPLTYHQFQALIASMPPPPPAEAAISAQGFNGATTPIADDHDDRFGVPTLEELGFDTEGLKPPV

WIGGESEALTRLERHLERKAWVASFGRPKMTPQSLFASQTGLSPYLRFGCLSTRLFYYQLTELYKRIKRVQPPLSLHGQI

LWREFFYCAATRNPNFDRMEGNPICVQIPWQKNQEALAKWANGQTGYPWIDAIMIQLREEGWIHHLARHAVACFLTRGDL

WISWEEGMKVFDELLLDADWSVNAGMWMWLSCSSFFQQFFHCYCPVRFGRKTDPNGDFIRRYIPALKNMPTRYIHEPWLA

PEAVQQSARCIVGRHYPLPMVDHTKASQINIERIKQVYAQLAQYKPLAILNPHTLQRPNVMQPSPSPTSILTNINQSNFL

CSQNSDAQIISTVQNSTTKSSNVFQHPGANNNSRNDTKQSQSKPVVIIQRENSANVQKIDYGRANPCPSPRPQQNYIING

QDVPHKSKEDTERAQKSKQEENYNLKIFDINKFIQDYSNNQKTYPNQERKTEDVYQQDEKSISYGFTKPKFYMSYPNNGI

VRNEHSHIETQEDEPTTRNHEYAGESTKETKKEKQIHNNCIQ

>Chilo_sup_CDK02014.2_cry_1 CDK02014.2

MLGGSVLWFRHGLRLHDNPALLSALEDRRMPLFPIFIFDGETAGTKLVGYNRMRYLLEALDDLDNQFKKFGGRLIMLKGK

PNIVFKRLWEEFGIRKLCFEQDCEPVWRARDDSVKDACREIGVQCREHVSHTLWEPDTVIRANGGIPPLTYQMFLHTVDT

IGDPPRPVVDADLCGVKFGALPECFYQEFTVFDKTPKPEELGVFLENEDIRMIRWVGGETAALKQMQQRLNVEYETFCRG

SYLPTHGNPDLLGPPISLSPALRFGCLSVRRFYWSVQDLFQKVHQGRLSSTHYITGQLIWREYFYTMSVNNPNYGQMAGN

PICLDIPWKSPEGDELQRWKEGRTGFPFVDAAMRQLLTEGWLHHVVRNTVASFLTRGSLWLSWEHGLQHFLKYLLDADWS

VCAGNWMWVSSSAFEALLDSGDCACPVRLGQRLDPSGEYVRRYLPALARVPDRYIYEPWKAPIEVQEQANCIIGKDYPAP

MVNHLVAAERNRFAMKDLREMLQKAPPHCCPSSEEEIRQFMWLNDEPAIALSTT

>Chilo_sup_QCH40574.1_CRY1_X1 QCH40574.1

MANKASVVHWFRLDLRIHDNLALRNAINEAENRQYYLRPVFIIDPDIKERVGVNRLRFLFQSLQDLDSNLRKLNSRLFIL

KGTADELFSKLFDQWQVKYLTSQIDIDPEIVKQDEIVDRLAEEKGIFIVRRVQHTVYDFHSVLKKNNGSVPLTYQKFLSL

VKDIKVKETIEITKTLSDYCKPPDSTCEEYNVPSLNDLGIDESSLKSCKYHGGETEGLKRLNLYMEKKNWVCKFEKPNTS

PNSIEPSTTVLSPYLSHGCLSSKLFYHRLKGVENGVAHSQPPVSLLGQLMWREFYYTAGAGTENFDKMVGNAVCTQIPWG

KNDKYLKAWSEGMTGYPFVDAIMRQLKQEGWIHHLARHMVACFLTRGDLWISWEEGARVFEDYLLDYDWSLNAGNWMWLS

ASAFFYKYFRVYSPVAFGKKTDKEGLFIRKYVPELKKYPTEYIYEPWKAPKSVQRTAGCVVGEGYPQRIVDHDKIHKENM

QKMAAAYKVNKEKKSLKRKLT

>Chilo_sup_RVE51139.1_evm_004282 RVE51139.1

MVAGFVKAESTNLPRIDALMVGEFVALNPDFCSAEQRNDKASIMASAAKKPKLSISSSSAKEETTDLTSFMQKIQKKREE

TAESILQYKFNKKRLRIVSQEQMVADKCEGIVYWMSRDSRVQDNWALLFAQKLALKNEVPLHVCFCLIAKYLDASVRQFH

FLIKGLEKVAADCKKLNITFHLLEGSGAEALPQWVVDHKIGAVVCDFNPLRVPLGWLEDCKKKLKKDVPLIQVDAHNIVP

CWEASNKQEYSARTIRNKINSKLGEYLTEFPPVIKHPYTSKFEPEPIDWDQAIESREADKSVGPVDWAKPGYDEAVKMLK

SFIDKRLKIFSSKRNDPTQDACSNLSPWFHFGQISVQRVALCVQEYKSKHTESVNAFLEEAIVRRELADNFCFYCEHYDS

LKGASAWAQKTLDDHRKDKRTHVYSLEQLSKAETHDDLWNSAQLQLVKEGKMHGFLRMYWCKKILEWSPTPEDALKYSIY

LNDHYSVDGRDPNGYVGCMWSICGIHDQGWAERAVFGKIRFMNYDGCKRKFNVNAFVARYGGKIHKNTSKK

>Chironomus_rip_unname_CAG9798887.1 CAG9798887.1

MSAKVTVIHWFRKGLRVHDNPALVKAIDGAIKRKAYLRPIFMLDPGIVKWMTVGANRWRFLQETLADLDKNLRKLNTRLY

VVRGNPKDHFPKLFEKWNVQLITFEHDIEPYSVKRDQILLKDAERFNVEVMIEYSLTVFNPELVIKKNKGSVPMTFQKFL

SVASELKVPQPAENPNKVPKSCEPELDDLEEKSIDCYNVPTLKQMGVSVEELSKASKFPGGETEALRRMTDTMKQVDYVC

KFEKPNTAPNSLEPSTTVLSPYLKFGALSVRLFYHEIKLAYKGRKHSQPPVSLEAQVIWREFYYCVGSATPNFDRMVGNR

ICAQIPWVHNQTYLDAWKNGMTGYPFIDAIMRQLKQEGWIHHLARHAVACFLTRGDLWINWEEGQKVFEEYLLDADWALN

AGNWMWLSASAFFHQYFRVYSPIAFGKKTDSQGLYIKKYVPELKNYPSGVIYEPWKVSLENQKKYGCIIGKDYPRQIVDH

DIVMKENLVKMKAAYAKKNLDTSKSEKRKAESPIKSSPKKKNSLDKYFKKK

>Chironomus_rip_unname_CAG9801410.1 CAG9801410.1

MSTVLFRKVYLFKFTSTIMKRFLTSDEPSTSKKAKTEDFLEEIQNNRLKTAESIVEFKFNKKRIKILNKYDEVPESNSEG

GICYWMARDQRVQDNWAMLFTQKLALKNKLPMHVVFCLTDKFMDATLRHYKFMLDGLEEVSLDLKKLSINFHLLIGEHKK

EIPKFVKDFKIGALVCDFSPLRIHREWVEKIKKELPSKVPFIQVDAHNIVPIWIASDKQEYAARTIRNKINSKLGDYLTE

FPPVIKHPYKPEDSLRPEPIDWEELLESLKIDRSVEPVDWIKPGYKNSIEMLESFIMKRLKHFGDKRNDPTLSVLSNLSP

YFHYGQIAVQRSIIEVKKYKSSASASVDAFCEEAIVRRELSDNFCFYNPNYDNLKGITDWAMKTLNDHRKDKRDYVYTRK

EFEEAKTHDDLWNSAQIQMTKEGKMHGFLRMYWAKKILEWTASPEEGLETAIYLNDRFNLDGRDPNGFVGCMWSIGGIHD

QGWGERKIFGKIRFMNYDGCKRKFDIKKFIARYGGVVHHKKK

>Chironomus_rip_unname_CAG9803342.1 CAG9803342.1

MAEKSILWFRHGLRLHDNPALIEALRSSGAEKNTFYPVFIFDGCSAGTQHVGYNRMKFLIESLEDLDEQFKELGAPGLFI

FRGDPVEIFRKMREELGITKICYEQDCEPIWNGRDERVENLCREFGIKSVEKISHTLWDPNRIIEVNGGFPPLTYQMFLH

TINVIGYPSKPCEFPDFSHVDFGEIPMNLHMQLGLMNGIPKPEDFSIFREKTGSEVYLTWKGGEKRALEQLELRLAIEQD

AFRNGTYLPNQANPDLLGVSTSMSAALRFGCLSVRKFYYAIHDLFASVQEILPNKHPYGHHITGQLIWREYFYTMSIKNP

NYGQMKNNPICLNIPWSIPNKDDVLKWKQGKTGFPIIDAAMRQLLTEGWLHHTLRNITATFLTRSGLWISWEVGLDHYLK

YLLDADWSVCSGNWMWVSSSAFEKLLDSSNFSIIALAYRLDPNGDYVKRYIPELRHFHQKYIHEPWKAPNNVQEACECII

GDDYPEPMIDLKRAMQINSNRMKEIRDSLIDSKPHVRPSNEDEIRTFFWINDDISVKA

>Chironomus_rip_unname_CAG9805920.1 CAG9805920.1

MTMTTGQSAAPSVGNVQPQQQKKEKHLVHWFRKGLRLHDNPAFKEGLKNATTFRCIFLVDPWFAGSSNVGINKWRFLLQC

LEDLDQNLRHLNSRLFVIRGQPAEKLPMLFKKWNTTCLTFEEDPEPFSRVRDHNITEMCKELKIEVITAVSHTLYNLEKI

IEKNNGKPPLTYHQFQAIIASIDPPPHPEPTITEELIGNARTPVNEDHDDKYGVPTLEELGFDTDGLKPPVWFGGETEAL

VRLERHLERKAWVASFGRPKMSPQSLLASQTGLSPCLRFGCLSTRLFYYQLTDLYKKIKKANPPLSLHGQLLWREFFYCC

ATKNPNFDKMAGNPICVQIPWDKNAEALAKWANGQTGYPWIDAIMQQLREEGWIHYLARHAVACFLTRGNLWISWEEGMK

VFEELLLDADWSVNAGMWLWLSCSSFFQQFFHCYCPVKFGRKADPNGDYIRRYVPALKNFPTKYIHEPWVASEAIQKAAK

CIIGKDYPLAMINHVTASKTNMERMKQVYQQLAKYRNLCNPDGGGSAISCMLAKAQASALANAEQINSSPSPTTILKSVN

SSGSYMCSTTRSNMEMQSPSPVVHYHEPNMINPSQLTQDQNNMMQHRNSSLTCLKDRHHKNQFDFDENDENSNKRNQYQY

HNSYNNYTQNQHQVISETQQQADQHTIELRMNNLGNERQDAE

>Choristoneura_fum_hypoth_KAI8429196.1 KAI8429196.1

MIKALHKMAAAVPSVIHWFRLDLRIHDNLALRNAINEAENRKYLLRPIYVLDPDIKNKVGVNRLRFLIQSLQDLDINLRK

LNTRLYVIRGKADEKIPELFDEWQVKYLTQQLDIDPEYVEKDCVIEKVAEKKDIFIVKRVQHTVYDCNSVLKKNNGSVPL

TYQKFLSLASELPVKECIEIKKQIPDSSNPSDFESHDYDVPSLSDLGIDDSTLVACKYAGGETEGLKRLDMYMARKDWVC

KFEKPNSFPNSLEPSTTVLSPYISHGCVSSKLFYHKLKEVENGRKHTEPPVSLMGQLMWREFYYTAGTGTENFDKMKGNS

VCTQIPWGKNDEHLKAWAEGRTGYPFVDAIMRQLKQEGWIHHLARHMVACFLTRGDLWISWEQGAKVFEDYLLDYDWSLN

AGNWMWLSASAFFYKYFRVYSPVAFGKKTDKEGLYIKKYVPELNKYPVAFIYEPWKAPKEVQQRAGCVIGVGYPKRIVDH

DKIHKENMQKMAAAYKANKEKKAQRPLKRKRSD

>Choristoneura_fum_hypoth_KAI8429887.1 KAI8429887.1

MSTATVSTRSNTRRPGSQVPRPSAGKHTVHWFRKGLRLHDNPALREGLTDAVTLRCIFIIDPWFASSSNVGINKWRFLLQ

CLDDLDNSLRKLNSRLFVVRGQPADALPKLFREWGTTALTFEEDPEPYGRVRDQNITSKCREVGISVTTRVSHTLFKLDT

IIERNGGKAPLTYHQFQALIAGMQAPLPPEATITSSMLKGTVTPVASDHDDRFGVPTLEELGFDVEGLKAPVWIGGETQA

LARLERHLERKAWVATFGRPKMTPQSLLASQTGLSPYLRFGCLSTRLFYYQLSELYTRVKHVRPPLSLHGQILWREFFYC

ASTRNPNFDRMEGNPICVQIPWEKNQDALAKWANGQTGFPWIDAIMIQLREEGWIHHLARHAVACFLTRGDLWISWEEGM

KSPNINMCTYTNLHSVYTKATTGQKVFDELLLDADWSVNAGTWMWLSCSSFFQQFFHCYCPVRFGRRIDPNGDYIRKYIP

ALVNMPTKFVHEPWMAPESVQRAAQCIIGRDYPVPMVDHNRAAHINIQRLKQVYSLLAKYKPQVALNSSTIQRPNIMKAP

ASPTSIIASMNHSNYLCSNQHAQRPRLPAKMAYKEMTTRSAPTQYAPNNNDIQVDNTNPPQFEQARTGPGTEATGVLHKD

IRHARSPRQHSQKDYIT

>Choristoneura_fum_hypoth_KAI8436281.1 KAI8436281.1

MLGGSVLWFRHGLRLHDNPALHSAVNDRTRPFFPVFIFDGETAGTKLVGYNRMRYLLEALDDLDNQFRKYGGRLIMVKAQ

PTAFFRRLWEEFGINRLCFEQDCEPVWRARDESVKTACREIGVTCHEHVSHTLWEPDTVIKANGGIPPLTYQMFLHTVAT

IGDPPRPVPNVDLTGVEFGRLPDCFHKEFTIFDKTPKPEDFGVFRDNEEDIRMIRWVGGETSALEHMKQRLSVERETFCR

GSFLPSHGSPDLLGPPISLSPALRFGCLSVRGFYWSIQDLFRDVHQGRLPPSPFITGQLIWREYFYTMSVNNPEYGQMSG

NPICLDIPWKEPEGDELNSWIEGRTGFPFIDAAMRQLRIEGWVHHSVRNTVASFLTRGTLWLSWEHGLQHFLKYLLDADW

SVCAGNWMWVSSSAFEALLDSSECACPVRLGQRLDPSGEYVRRYVPELTNMPERYIYEPWKAPIDVQERAQCVIGTHYPA

PIVDHLAAARRNRDAMQELRQMLEKAPPHCCPSSEDEIRQFMWLHDDTAIQANTA

>Chrysodeixis_cha_CPD_PL_ADE60793.1 ADE60793.1

HLSRVPCWVASEKQEYSARTIRNKINSKLDEYLTEFPPVIKHPYTAKFDPEPIDWDEAIVSREADKNVGPVAWARPGYDE

AVKMLKSFLENRLKVFATKRNDPTKDALSNLSPWFHFGQISVQRVALCVQEHKSKYTESVNAFLEEAIVRRELADNFCFY

CEHYDSIKGASQWAQKTLDDHRKDKRTHIYTLEQLAKSETHDDLWNSAQIQLVKEGKMHGFLRMYWAKKIGHSFFPK

>Chrysodeixis_inc_unname_CAD0195136.1 CAD0195136.1

MRLYYLPLLINEIKMASAPKKLKLSAPFSSETKGSSTNAEELMKKFQTKRVETAKSILEYKFNKKRLRIVSQEQTVPDKC

EGIVYWMSRDSRVQDNWAFLFAQKLALKNKVPLHVCFCLIAKYLDASVRQFDFLIKGLEKVAADCKKLNISFHLLEGSGA

EVLPQWVVKHNIGAVVCDFNPLRVPLGWLDGVKKKLKKDVPLIQVDAHNVVPCWVASEKQEYSARTIRNKINSKLDEYLT

EFPPIIKHPHTAKFDPEPIDWDEAIVSREADKNVGPVAWARPGYDEAVKMLKSFLDTRLKVFATKRNDPTKDALSNLSPW

FHFGQISVQRVALCVQEHKSKYTESVNAFLEEAIVRRELADNFCFYCEHYDSIKGASQWAQKTLDDHRKDKRTHIYTLEQ

LAKSETHDDLWNSAQIQLVKEGKMHGFLRMYWAKKILEWTATPEDALKYALYLNDHYSIDGRDPNGYVGCMWSVCGIHDQ

GWAERSVFGKIRFMNYDGCKRKFDIKAFIARYGGKVHKYVPKK

>Chrysodeixis_inc_unname_CAH0579486.1 CAH0579486.1

MLGGSVLWFRHGLRLHDNPSLHAALEDRNVPFFPMFIFDGETAGTKLVGYNRMRYLLEALDDLDQQFKKHGGRLIMLKGK

PNVVFRRLWEEFGIRKLCFEQDCEPVWRARDDSVKTACKEIGVTCKEHVSHTLWEPDTVIKANGGIPPLTYQMFLHTVAT

IGDPPRPVCDVDFTGVKFGTLPESFYQEFTVFDKAPKPEDLGVFLENEDIRMIRWVGGETTALKQMQERLAVEYETFCRG

SYLPTHGNPDLLGRPISLSPALRFGCLSVRSFYWSVQDLFRQVHQGRLDSTQFITGQLIWREYFYTMSVNNPNYGQMAGN

PICLDIPWKNPEGDELQKWVQGRTGFPFVDAAMRQLRSEGWLHHAARNTVASFLTRGTLWLSWEHGLQHFLKYLLDADWS

VCAGNWMWVSSSAFEALLDSGECACPVRLGQRLDPSGEYVRRYVPELARMPGEYIYEPWKAPIDVQERAKCIIGKDYPAP

VVNHVVAAQRNRNAMKELRHILQKAPPHCCPSSDAEIRQFMWLND

>Chrysodeixis_inc_unname_CAH0600495.1 CAH0600495.1

MEIDRDIKSRLFCTRCDVGNYVTMSAAAETLPAPSAAPQTPAAPAPPTPAPASERRPPGKHIVHWFRKGLRLHDNPALRE

GLLDATTFRCVFIIDPWFASSSNVGINKWRFLLQCLEDLDASLRKLNSRLFVVRGQPADALPKLFREWGTTALTFEEDPE

PYGRVRDHNIMSKCREVGITVTSRVSHTLYKLDQIIERNGGKAPLTYHQFQALIASMPPPPPAEPPISAQNLNGATTPVS

DDHDDRFGVPTLEELGFEIEGLKPPVWIGGESEALARLERHLERKAWVASFGRPKMTPQSLLASQTGLSPYLRFGCLSTR

LFYYQLTELYKRVKRVRPPLSLHGQILWREFFYCAATRNPNFDRMEGNPICVQIPWEKNQEALAKWASGQTGFPWIDAIM

IQLREEGWIHHLARHAVACFLTRGDLWISWEEGMKVFDELLLDADWSVNAGMWMWLSCSSFFQQFFHCYCPVRFGRKTDP

NGDFIRRYIPALKNMPTRYIHEPWVAPEAVQQSARCTIGRDYPMPMVDHSKASQVNIERIKQVYAQLAKYKPQAGALNPN

AVQRPNVMQSSPSPTSIITSINQSNYLCSQTPDPQTTSPQVISYKDNDVFQKPMQHRSIKPFKQVVIVQKTQNTNVIQSV

ANSTTKENYVVNRQVSYKAPINDLQSEKQENYDFKNLVINNFVQGYSKGQEIFDNQPSNRNEVFAQQTLKINSFNYEKQK

FYLSSFNDGLRGENIHNETPPSYETGIIHDSDLSFSRKTKTESPEDEAHESTCSQPMAINDDATITNENGQNDPSN

>Chrysoperla_carn__XP_044730763.1 XP_044730763.1

MNSLFMTKINIKNSAAFCSVEYYEGTWRAMSSGVGSAGDIRKTGGPPPPDKHMVHWFRKGLRLHDNPSLREGLVGATTFR

CVFILDPWFAGSSNVGINKWRFLLQCLEDLDRSLRKLNSRLFVIRGQPADALPKLFKEWGTTSLTFEEDPEPYGRVRDEN

ITALCNELGISVVQRVSHTLYKLHQIIENNGGQAPLTYHQFQAVIANMDAPSAAEGGITQKNIGNATTPLTDDHDDKYGV

PTLEELGFETEGLLPPVWQGGESEALARLERHLERKAWVASFGRPKMTPQSLLPSQTGLSPYLRFGCLSTRLFYYQLTDL

YKKIKKACPPLSLHGQLLWREFFYCAATKNANFDKMIGNPICVQIPWDKNAEALAKWANGQTGFPWIDAIMTQLREEGWI

HHLARHAVACFLTRGDLWLSWEEGMKVFEELLLDADWSVNAGMWMWLSCSSFFQQFFHCYCPVRFGRKADPNGDYIRRYL

PVLKNFPTRYIHEPWVAPESVQRAAKCIIGKNYPLPMVNHAVASRINIQRMKQVYQQLARYRNDDAEKKNLINVANQRQN

MVAMSPSPNTVMSSINNSYNLAVSQKQSSIPNNKTESAYNIASEQYNTDYNQQQNQQTSQHQQFYTIVQTNHSINTFTES

LNNQQEQFKQQHHHQQEQQQQSNQEQYENTRQTYCEPMKQSPINTQHNYDYMRENRNNFCNEPIKKSNLHNFCEPMKHSP

MVQIEQESYKQNYQVTVQDNDGNTSVVTIVQQQQQEQLIGNCSRTNTINDDDSSMTINIKREEQRQ

>Chrysoperla_carn__XP_044734495.1 XP_044734495.1

MESSALWFRHGLRLHDNPSLHEAIKDRDRLFYPIFIFDGESAGTKLCGYNRMKFLTECLEDLDKQFRKYGGQLFVFENKP

EVVFKALNKHYNLKLLCFEQDCEPVWRVRDENVKRLCDKRGVKWMECVSHTLWDPFRVIEENGGTPPLTYQAFLYVVSNI

GNPPRPVDDVNWQGIKFGIISPEIAAELKMFPKVPTAEDLGYTREAPNEIRMFRWIGGETQALKLLQNRLAIEKSAFDGG

YFLPNQANPDLLGAPTSQSAALRFGCLSVRRFYWNLQDVFKTVHGGNLPPSPHITGQLIWREYFYTMSVNNEYYAEMERN

PICLNIPWSEPKKEDLNRWKYGKTGFPFIDAVMRQLLLEGWVHHVARNAVACFLTRGDLWISWEYGLHHFFKYLLDADWS

VCAGNWMWVSSSAFEQVLDCSECVCPVAFGRRLDPGGEYVKRYLPELRSLPSEYLYEPWKAPIDVQERSNCIIGKDYPPP

IVDHTEASKRNKYFMQTLREQIMGGGNAPPHCRPSDENEAKSFLGLPSQCHEHCLIQI

>Chrysoperla_carn__XP_044735319.1 XP_044735319.1

MIANEYIASQKRTNSEAFKTKTKTKDDSTVKDETKTNDLITSLKEKRNQTAKSILDFDFKKKRVQILSQAKEIKESDSSG

GILYWMFRDCRVQDNWALLFAQKLALKNELPLNILFCLPQKYLEYNARHYKFLIKGLEEVQEECDELNINFNVCVGEPAI

QVVDFIESNHIQGVVCDFLPLKDCMQWQESVLNQIDSNQIPFIRVDAHNIVPCWIASDKLEYAARTIRNKINSQLSQYLQ

QYPPVIQHKYNNKNIINKKKIDWNDVLNQLKYDKSDELINNNEITWAEGGTSNGLKMLQSFQQKRIQLFGTKRNDPTISA

LSNLSPWLHFGHISAQRCVLVIQELKSKYTESVNSYCEEVIIRRELSDNFCYYNINKYNKLDGAPSWAQLTLKQHSKDKR

TYLYTLDELQHGRTHDDLWNSAQIELYKTGKLHGFMRMYWAKKVLEWTESPEQALQYALYLNDQYALDGNDPNGYVGCMW

SICGVHDQGWGEREIFGKIRFMNYQGCKRKFDIKAYIARWGGKEYNKKQETIPFKKLKK

>Chrysoperla_carn__XP_044742491.1 XP_044742491.1

MEKKTVIHWFRKGLRIHDNPALLAAIEEAVKSSSALRPIFILDPLILTWMRVGPNRWRFLQQSLDQLNENLTKIGSKLYV

IRGTPENVFKRIFKDWNISYITYEYDTEPYAKRRDALIEQLAHEANIRIESEISHTIYNTQLVIKKNGGKAPLTMQKFLS

VVDSMSKPPIPVPAPTKIPEICKPTLDSFEKENPHCYDCPTLLELKVNEKNLNACKFPGGETEALNRLNKILLKKSWICN

FEKPNTSSNSLEPSTTVLSPYLKFGCLSSRLFYYEINKIISGQKHSKPPVSLIGQLMWREFYYTVSSDTPNFDKMVGNPI

CCQVPWDTNEKHLEAWTYGKTGYPFIDAIMRQLRQEGWIHHLARHAVACFLTRGDLWISWEEGLKVFDELLLDSDWALNA

GNWMWLSASAFFHQFFRVYSPVAFGKKTDKFGDYIKKYVPELNKYPVDYIYEPWKAPLSVQTKAGCLIGKDYPKRIVIHE

DVYKVNISKMSLAYKNNKTNDNNKQKGESSKESEPAAKKMKKVSKSTNTTKSDSKKSKQTSLTKFLKK

>Cimex_lec_CRY-1-_XP_014255347.1 XP_014255347.1

MMTEKNTVHWFRKGLRLHDNPSLRQALKGAKTFRCIFILDPWFAGASNVGINKWRFLLQCLEDIDRSLRNLNSRLFVIRG

QPADILPKLLKEWGTTCLSFEEDPEPFGRVRDQNIIVMCKSMNIGVVTSVAHTLYKLEKIIEKNGGKAPLTYRQFQSIIS

SLEAPPHPEPTVTLSWIGSATSLLSDDHDEKYGIPTLEELGFDTEGLLPSVWHGGESEALSRLERHLERKAWVASFGRPK

MTPQSLLPSQTGLSPYLRFGCLSTRLFYYELNELYKRIKKAVPPLSLHGQILWREFFYCAATRNPNFDRMIGNPICVQIP

WDKNPEALAKWANGQTGFPWIDAIMTQLREEGWIHHLARHAVACFLTRGDLWISWEEGMKVFDELLLDADWSVNAGMWMW

LSCSSFFQQFFHCYCPVKFGRKADPNGDYIRKYLPVLKNIPTKYIHEPWNCPESVQKAAKCIIGIDYPRPMLNHSVVARH

NTERMKQVYQRLIKFKESGLSTLLNRIPVCYSEKMKRDDTYEDSILITPALPFNKGIL

>Cimex_lec_PL_X1_XP_014256100.1 XP_014256100.1

MCTPAKRIKTQGDESFEEFLKSVEAERHEVASSVLDFPYNKKRVRVLSEAKELPESSNGVLYWMTRENRVQDNWSLLFAQ

KLALKNEVPLHVCFCLLPKYMDATIRHYRFMLKGLEELSLELKNLQIPFHLIFCSDNVETVRKFVAKNDIGAVVLDFSPL

KISTSWVDQLKESLPDNVPLCQVDGHNIVPCWIASDKLEYGARTIRNKINTKLDEFLTKFPPVIKHPFKSNIKSEEIEWS

KLEEKLDVDMKVSPVNWAKPGYKAGMQTLFEFCDKRLKNFATKRNNPLENALSNLSTWFHFGQISIQRCILYVQTFKSKY

KDSVSSFCEEAIVRRELADNFCFYNKNYDNLDGAYDWAKKTLNDHKKDKRTWLYTDQELEEAKTHDDLWNSAQIQLNTEG

KMHGFLRMYWAKKILEWTQSPEDALRVALYLNDKYSLDGRDPNGFVGCMWSICGIHDQGWREREIFGKIRYMNYQGCERK

FDVKAFVARYGGKVYGKKKSKS

>Cinara_ced_Hypoth_VVC30291.1 VVC30291.1

MNILQTATSVMEFKFNKKRVRVLSKTSEVPEWADGVIYWTFRDERIHDNWALLYAQKLAIKNKVSLHITFCRLTKFLDCS

LRHYKHIFQGLEELENECKTLNIQFHFLIGSAAEILPDFVKKHKLGAVVVDFMPVREHMLWTQQLADSIGSEIPVVQVDA

HNIVPCWVASDKLEYAARTIRNKINKNLPEFLTEFPPVIKHPFSGKLKTQPTNWNDADKTLQVDRSVLPVPGLKAGFKAG

MNELDNFLKKRLSKYSTDRNNPVKDGLSNLSPWLHFGQISAQRCILEVSKFSKMYPESVLAYREETIVRRELSDNFCFYN

PKYDSIEGAPNWAQVTLNDHRKDKRKFVYTREELEKSRTYDDLWNSAQIQLVKDGKMHGFLRMYWAKKILEWTDTPERAL

ADAIYLNDKYSMDGRDPSGFVGCMWSICGVHDQGWREREIFGKIRFMNYDGCKRKFDINAFIARYGGMVHKYTKK

>Cinara_ced_Hypoth_VVC34308.1 VVC34308.1

MDQPKNKHTVHWFRKGLRLHDNPSLREGLVDATTFRCIFILDPWFAGASNSGINKWRFLLECLVDLDNSLKKLNSRLFVI

KGQPAEALPKLFKLWGTTNFTFEEDPEPYGRVRDQNITVMCHEMGISVIKRCSHTLYQLDKIININNGKAPLTYHLFQTL

LECIDPPDCAVPNIDLEFLGGAYTPIRFDHDEIFGVPSLSDLGFKEMNDVSNHIWKGGEIEALIRLQRHLERKAFIATYG

KPKMTPQSLLASPTGLAPYLRFGCLSTRLFYSELNSLYQKMRKSRPPLSLYGQLLWRDFFYCASTNNPNFDRMVGNPICM

QIPWDKNPQALSKWAKGQTGYPWIDAIMIQLKKEGWIHCIARHAVACFLTRGDLWLSWEEGMKVFEELLLDADWSVNAGS

WMWYSCSSFFQEFIHCYCPIRFGRKADPNGDYIRRYIPVLQNMPTKYIHEPWLAPKTIQFAANCIIGIDYPLPIIDHVFA

SKINLERMKLAYEHLSNCQPRLENNKLILISSPKR

>Cinara_ced_Rossma_VVC31760.1 VVC31760.1

MTYNMTVAVHWFRNGLRLHDNPALIEAVNKADKLITLFIFEENMYNTDVIGYHPMRFLLESLDDLNTSLTKLGGCLYILQ

GNPVKIFNRIKEEIGLNLITYEQDCAHNGRIRDELVVQYCNANNVQYIEKVSHTLWNPKTIIDTNGGEPPLTFKKFQTLA

LSINKPLKPINNVDWSTVSFGKLPVTLLEELKALDNPTTQDFGIVPEAPEINNSYNCWYGGETKALEKLTDRLNFEKEAF

VNGFYLPNQINPNLLDPSYSLSAALRYGCLSIRKLYWELSKLFIQNFEGDLLPQYSATSQLIWRDHFYSMSFNNEYFDQM

EDNMACLKIPWNDIETGENKQMLEYWKTGKTGYPFIDAGMRQLLQEGWVHHVVRNSLACFLTRGDLWISWTEGLKHFLKY

LIDADYSVCSGNWIWVSSSTFEQILDCPLCICPVSYGVRLDPSGEYIRRYVPELKNMPDQYLNQPWKCPDSIQKEVGCII

GKDYPHRIVEHTEVARENRKKMQSLRISLMNSSMVPHCRPSDQTEVKQFMHLPEECMDQLFLNDNLTTYEHLHIH

>Cinara_ced_Rossma_VVC43723.1 VVC43723.1

MNPNKKDRCHETSIHWFRKGMRLHDNPAFKLSYELKTCCGEYYKLRPIYILDPYFRKYIRAGMNRWRFLQQSLVDLDLTL

RKLGTRLYVIRGLPEDVFPDLFKKWNVKLLTFELDTEPYARKRDEQIETLARQHGVKVDQKVSHTMYNTELVVRANRGSV

PMTYQKFLSVINSMPDPRQPIPAPDKLPSECLFDDDLNNCEYDVPTLNELLTLKGFDPVDLKPCLYPGGEKEALKRLEEY

MEKKSWVCKFEKPDTSPNSLKPSTTVLSPYMKFGCLSASHFYYRLKEVIGNSPHTKPPVSLIGQLYWREFYYTVGAATPN

FDKMVGNPICCQVPWDDNPVALEAWTNGKTGYPFIDAIMRQLRDEGWIHHLARHSVACFLTRGDLWISWEKGLAVFEELL

LDADWSMNAGNWMWLSASAFFHQFFRVYSPVVFGKKTDKSGDYIRKYIPELAKYPDQYIYEPWLAPKSIQERAGCVVGIH

YPKRVVIHEDVYKININKMSLAYKTTKADKCGSSTKSKNAPSSSNEKNVKKAKHK

>Cloeon_dip_CAB3360699.1_protein CAB3360699.1

MFTNKLTQQTNLLTQQLTTIFLTRIFSLSVANSFIRQSRTMASGPKKLKLGESSKPINNDAFFTEINEARAKAGKSVMDF

KFNKKRIKVLSKASDVPEECKGIVYWMSRDQRVQDNWAFLFAQKLAIKNKVPLHICFCLLSKFLGAPFRHFKFMLDGLQE

VQQECKKLEIQFHLLEGEAASVLPAFIKAHDIGGVVIDFSPLRTHRSWVDELKKKLPSTVPLCQVDAHNIVPCWVASDKL

EYGARTIRKKVMDKLPEFLTEFPLVAKHEYPAKFKAEPIDWTAVEEKLEVDRSVGPVSWAKPGSEAGLAMLNEFCCKRIK

LFGSKRNDPNINALSNLSPWFHFGHLSVQRAIKYVQKFKSSHSESVAAFVEEAVVRRELSDNFCMHNPKYDSIEGTNNWA

KETLEVHKKDKREYIYSLEQFDQAKTHDDLWNSAQLQMVREGKMHGFLRMYWAKKILEWTESPEQALEFAIHLNDKYNLD

GRGPQWICWLHVVDLRHSRPGLGREASVWQDSLHELPGLQTQV

>Cloeon_dip_CAB3360724.1_protein CAB3360724.1

MSGTETGTRSPPHAEKHTVHWFRKGLRLHDNPSLLEGLRAATTFRCVFILDPWFAGSSNVGINKWRFLLQCLEDLDRSLR

KLNSRLFVIRGQPADTLPKLFKEWGTTCLTFEEDPEPFGRVRDQNIMTLCKELGLSVVSRISHTLYDLEKILEKNNGKPP

LTYHQFQNVVASMDSPPQPEPMVTLDTLRGCWTPLTEDHDDKYGVPSLEELGFDTEGLLPPVWRGGESEALARLERHLER

KAWVASFGRPKMTPQSLLASQTGLSPYLRFGCLSTRLFYYQLTDLYKKIKKAAPPLSLHGQLLWREFFYCAATKNPNFDK

MAGNPICVQIPWDKNPEALAKWANGQTGFPWIDAIMTQLREEGWIHHLARHAVACFLTRGDLWVSWEEGMKVFEELLLDA

DWSVNGGMWMWLSCSSFFQQFFHCYCPVRFGRKADPNGDYIRRYLPVLKNFPTKYIHEPWNAPEAVQRAAKCIIGKDYAV

PMVNHAIASRINMERMKQVYQQLSKYRGAGLLATVPSSQNGAIPPSEPLHSVTTLSGLPNNMNLKQSSLSPPKDKHRSKS

PGAPMLPPARPQQHHKSNQQQQQQIGDCHFQKQSGI

>Cloeon_dip_CAB3366165.1_protein CAB3366165.1

MSRGTVIHWFRKGLRLHDNPALLEAIRECEKQPEVIKELRPIFILDPLILDHMRVGPNRWRFLQQSLSNLDDRLKALGSA

LLVIRGKPEEVFPKLFKTWNVKCITFENDIEPYAKKRDEQIEAMAKDAGIKVIQKVSHTLYSPAKILNANMGKVPLTYQK

FVSVAEGLGPPPAAVDEPKKVPPGYSQILESRELKIPKEEDIFGNTNVPTLEELGLDESKLEPCLYPGGEAEALQRMQIC

LAKKAWICDFEKPNTSPNSLIPSTTVLSPYLKFGCLSPRLFFHELNKIIQGRKHSKPPVSLVGQMYWREFYYVVGFATPN

YDRMVGNSICCQIPWGNNQEHLKAWTEGRTGYPFIDAIMTQLRKEGWIHHLARHAVACFLTRGDLWISWEEGQKVFEELL

LDADWALNAGNWMWLSASAFFHQYFRVYSPVVFGKKTDKFGDYIKKYLPILKKYPVEYIYEPWNAPLSVQKAAGCIIGTD

YPRPIVKHEVVSKENIAKMNAAYKANKANKGEAGKSKSSAAGGGSSAKQAKKR

>Cloeon_dip_CAB3385983.1_protein CAB3385983.1

MRIQCIGRSPPHARVIVPREAPVTWTATYAPALGAPVIQQRQWPTMNRPVQMSVLWFRHGLRLHDNPALLDAISGPPSMF

FPIFIFDGESAGTKVIGFNRMRFLLESLADLDRQLRSRGGRLWLLRGDPSKIFQRLWEELALTKVCFEQDCEPIWNERDQ

SVRATCAERGVRCEEFVSHTLWDPKAVIYANGGLPPLTYQMFLHTVSTLGEPPRPVEDPDWTGVTFGTLPPRLVKEFQVF

DEVPKPEYFGLSQPGYGLVRWIGGETRALQHLRERLTQEEQAFRKGIILPNQSRPDLLGPPTSQSAALRYGCLSVRRFYW

NLHDLFNAVSQGDPPAGLNISGQLIWREYFYTMSVYNPHYAEMSANPICLDVPWDKNPTFLEKWKKGQTGFPFIDAAMRQ

LVSEGWIHHVARNSVACFLTRGDLWISWEDGLAFFLEHLIDADWSVCAGNWMWVSSSAFEQLLDCSKCLCPVNYGRRLDP

WGEYIKRYVPELRNLPVEYLYEPWKAPLDVQRDAGCIIGDHYPERIVDHQRASNQNRQVFQLIEGFNNNDFNIK

>Clunio_mar_AFS34617.1_cry_2 AFS34617.1

MPDPNQLPKKEKHIVHWFRKGLRLHDNPALREGLKNATTFRAVFFVDPWFAGSSNVGINKWRFLLQCLEDLDQNLRRLNS

RLFVIRGQPAEKLPMLFKKWNTTCLTFEEDPEPFSKVRDNNITEMCKELNIEVISAVSHTLYKLEKIIEKNNGKAPLTYH

QFQAIIASMEPPPPAEATIAEDIIGNTRTPIDDDHDDRYGVPTLEELGFDTEGLKPPIWIGGETEALARLERHLERKAWV

ASFGRPKMSPQSLLASQTGLSPYLRFGCLSTRLFYYQLTDLYKKIKKTCPPLSLHGQLLWREFFYCCATKNPNFDKMSGN

PICVQIPWDKNPEALAKWANGQTGYPWIDAIMSQLREEGWIHHLARHAVACFLTRGNLWISWEEGMKVFEELLLDADWSV

NAGMWLWLSCSSFFQQFFHCYCPVKFGRKADPNGDYIRKYIPALKNFPTKYIHEPWVASEAIQRTAKCIIGKDYPLPMIN

HVIASRNNMGRMKQVYQQLAKYRQTPNNPEFGGSAISNILAKAQASAKAFTEQQSSSPSPTTILKTVNNSGNYMCSQRSD

HIEMPPQTVLYHEPSLNASSSVLQQQNMMKHRNNTNDMCHQQENINEFQYDEDGTSQHHNSYNYSQKQMTALNETQQQED

QHTIEFRMNNLGNEDTG

>Clunio_mar_CRK90905.1_CLUMA_CG00 CRK90905.1

MTKSVLWFRQGLRLHDNPALIEAINTDDGRRQVTFYPVFIFDGESAGTKDVGYNRMKFLLESLLDLDEQFKQLGAPGLFI

FQGKPTEIFQNLHDNIGINKICFEQDCEYNERDNEIKYLSRELGIEVVEKVSHTLWNPEDIIRINGGFAPLTYQMLLHTV

NVLGLPPRPVNNEVDFSQVNFGTIPEHHKINLRLMKSIPSPEDFLIFPENTGSDVYVNWNGGERHALEQMIDRVKHEEEA

FASGTYLPNQANVDLMGSPKSMSAALRFGCLSVRRFYYTIHDKFNEVQDKMVYKLPGGHHITGQLMWREYFYTMSINNPY

YGQIKNNPICLNIPWKSSEEDEILKWKQGRTGVPIIDASMRQLLAEGWLHHTLRNLTATFLTRTGLWISWEVGLQHFLKY

LLDADLPICGGNWMWVSSSAFETLLDSSRCSIISLAHRLDPKGEYIKRYVPELRNFPMKYIHEPWRAPIDIQEDIECIIG

QDYPSPMIDIVQALQINCNRMKKIRESLIESQPHVRPSNEDEIRTFFWIADEIAIQCN

>Clunio_mar_CRL02690.1_CLUMA_CG01 CRL02690.1

MSKRQTAIHWFRKGLRVHDNPALATAVNEAIKRDLILRPMFMLDPTIIQWLKVGPNRWRFLQQTLADLDKNLRKLNTKLF

VVRGNPKEDFIKLFKEWNVILLTFESDIEPYSVDRDATVIKAAKQFHIEVIQECSHTIFNPELVVKRNGGSAPLTYQKFL

SVASSIKVPSPVEFPPKLPASSLPVNDHYEEKDENCYDVPTLKELGLNEKDLGSCQFPGGESEALSRMEMNLKRKKWICD

FEKPKTSPNSLEPSTTVLSPYLKFGALSSRLFYKRLKEVYKGSKHSNPPVSLEGQIMWREFYYTVAATTPNFNKMVGNRV

CAQIPWVKNQKYLEAWSFGRTGYPFIDAIMRQLRQEGWIHHLARHAVACFLTRGDLWCHWEEGQKVFEELLLDADWALNA

GNWMWLSASAFFHQYFRVYSPVAFGKKTDSEGKFIKKYVPELKNFPSGIIYEPWKASLENQNKYGCIIGKDYPNRIVDHD

IVMKENLVKMKAAYAKKKKFKR

>Clunio_mar_CRL08549.1_CLUMA_CG02 CRL08549.1

MKRFLETNDQQSSSKKTKTENSSSDFVISIQNNRLKTAESIRDFKFNKKRVKILSKVEDVAEKSNGIAYWMARDQRVQDN

WALLFSQNLAIKNKIPLHVVFCLTDGFLGATIRHFDFMLKGLEEVANDCTTLNINFHLLRGEHTKEIPKFIKNNKIGAVV

CDFSPLRIHRSWVDGIKKELPPYVPLVQVDAHNIVPIWITSDKQEYAARTIRNKINSKLGEFLTEFPPLVKHPHKSTEMP

DKIDWKKTLQSLKVDETVGVVDWIKPGYKNAVEVLETFINKRLKVFATKRNDPTINALSNLSPFFHFGQISVQRAIIEVQ

KHKSKAKDSVDAFCEEAIVRRELSDNFCFHNKNYDNLDGITDWAKITLNAHRKDKRPYLYTRDELEKSKTHDDLWNAAQN

QLRVEAKLHGFLRMYWAKKILEWTASPEEALETAIYLNDRYNLDGRDPNGYVGCMWSIGGIHDQGWGERAVFGKIRYMNY

EGCKRKFDIKAFIARYGGVVHKKK

>Coccinella_sept__XP_044750690.1 XP_044750690.1

MTDNETSDITSHIHHHRMASIKDKHTVHWFRKGLRFHDNPAMLEGLKNSKTFRCVFVLDPWFAGSSNVGINKWRFLLQCL

DDLDNNLKNLNSRLFVVRGQPADALPKLFKEWGTTCFSFEEDPEPFGQVRDKNIIALCNELGITVIQRPSHTLYDLDKII

EKNGGTAPITYHQFLEVISSMGSPSEPLPTPFDSVSSNQLNTPFTNDHDHEFGVPTLEQLGFDTKTLSAAVWNGGESEAL

RRLERHLERKAWVASFGMPKMTPQSLLSSQTGLSPYLRFGCLSTRLFYYRLTALYKKIKNAFPPLSLHGQLLWREFFYCA

ATRNPNFDKMLGNPICVQIPWDQNAEALAKWANGQTGFPWIDAIMTQLRQEGWIHHLARHAVACFLTRGDLWISWEEGMK

VFEELLLDADWSVNAGTWMWLSCSSFFQQFSHCYCPVKFGRKADPNGDYIRKYICVLRNMPIKYIHDPWTAPDNVQKAAK

CVIGKDYPLPMVNHSLVSKINIQRMKQVYRQLKSYSENNDEFGTQITENCKNSCQYVQTDK

>Coccinella_sept__XP_044764764.1 XP_044764764.1

MAPNVLKDLAKEIFAKNIATQRSEAGKSIKEFKFKKDRCRLLSKYEDLREDSKGIIYWMSRESRVNDNWALLFAQKLCFK

YEVPLHVCFFLDDYKELYPTTRQAGFLRKGLEHVRKDLEKLNIPFYLLKKSPLELVDIIQANDIGCLVCDFFPLRIVTNW

QEKLKELLPSNVAIVQVDAHNIVPCWIASDKLERAARSIRPKILKQLPTYLTEFPAVSKQKEKKELVIKPGLEWSKNDKL

DIEFVEEITWAEPGEEGGIEMLRTFLLERLQYYGISSNEPSKKHQSNLSPWLHFGQISAQRIALEVSKLKKTWTTQCERF

LEEAIIRKEICDNFCLYNPNYDSFEGADKWAKETLNLHRDDKRDFLYTPEQFENCLTHDPIWNSAQFQLVTEGKLQGYMR

MYWCKKILEWTESPEKAIKTALWLNDKYSIDGSDPNGFVGVMWSICGIHDQGFKERPVFGKIRFMVDYSLSRKYDIKTYC

AKYRPNAKVSGNITKYLQTSNKKNEDKECDSKSPNKTEGKNGKNKETETEDKVKPAKQSSDKSCGKKRTVKSETHEESKK

KRKV

>Colaphellus_bow_CRY2_APP94027.1 APP94027.1

MNGRTDQASNGQEKHTVHWFRKGLRLHDNPSLKEGLKGAKTFRCVFVLDPWFAGSSSVGVNKWRFLLQCLEDLDRNLRKL

NSRLFVIRGQPADALPKLFKEWGTTVLTFEEDPEPFGKVRDHNITALCEELGTKVITRSSHTLYNLDQIIERNGGEAPLT

YHQFLAVIASMGPPPQPELPVNASSLNGAYTPLSDDHDEKYGVPTLEELGFDTEGLNPPVWQGGESESLARLERHLERKA

WVASFGRPKMTPQSLLPSLTGLSPYLRFGCLSTRLFYYQLTDLYKKIKKAFPPLSLHGQLLWREFFYCAATKNPNFDKMN

GNPICVQIPWDKNAEALAKWANGQTGFPWIDAIMAQLRQEGWIHHLARHAVACFLTRGDLWISWEEGMKVFEELLLDADW

SVNAGMWMWLSCSSFFQQFFHCYCPVKFGRKADPNGDYIRKYLPVLKNMPVQYIHEPWLAPENVQQAAKCIIGKSYPLPM

VNHTTASRINIQRMKQVYQQLANYKMIENARYPVADGCSDKFQRQPNVVTVGNPDKTV

>Colias_cro_CRY-1_XP_045493555.1 XP_045493555.1

MIKYLQTMSAIPSVVHWFRLDLRLHDNLALRNAINEAENRKHILRPIFIIDSEIKDQIRGNRLRFLIQSLQDLDTNLRKL

NTRLYIIKGKRTQCLLEVLKKWDVKYITSQVDIDPVYVSQDEIVEKYCEENNIFIVKRVQHTVYDCNSALKKNNGNVPMT

YQKFLSLVKDVQVKETIEITKQVSDNCKPTDFDSKDYDVPSLSEMGLDESTLSECKYPGGETEALKRLDVYMAKKQWVCA

FEKPNTSPNSIEPSTTVLSPYLSHGCLSAKLFYHKLKQVESGSRHSEPPVSLLGQLMWREFYYVAGTGTENFDKMVGNAV

CTQIPWKKNDEYLQAWAEGRTGYPFVDAIMRQLRQEGWIHHLARHMVACFLTRGDLWVSWEEGAKVFEDLLLDYDWSLNA

GNWMWLSASAFFYKFFRVYSPVAFGKKTDKEGLYIRKYVPELKKYPTEYIYEPWKAPKSIQTAAGCIIGVDYPKRIVDHD

KIHKENLQKMSAAYKINKEKKAKKRPRSD

>Colias_cro_CRY-1_XP_045497381.1 XP_045497381.1

MLGGSVLWFRHGLRLHDNPALRDALEDTSIPFFPIFVFDGETAGTKSVGYNRMRYLLEALDDLDRQFRMHGGRLLMIKGK

PSHVIRRLWEEFGISKLCFEQDCEPIWRARDESVRSVCREIGVTCRERVSHTLWEPDTIIRVNGGIPPLTYQMFLHTVAI

IGDPPRPVDDVDLRKVNFGVLPDSFYSEFVVFDKTPKPEDLGVFLEKEDIRMIRWVGGETAALQQADQRLAVEHETFRKG

SYLPTHGSPDLLGPPISLSPALRFGCLSVRRFYWAVQDLFQKVHQGRISSTHFITGQLIWREYFYTMSVNNPRYGQMEGN

PICLDIPWKQPQGDHLQRWKEGRTGFPFIDAAMRQLLAEGWLHHALRNTVASFLTRGTLWLSWEHGLKHFLKYLLDADWS

VCAGNWMWVSSSAFEALLDSGECACPVRLGLRLEPSGRYVRRYVPELTHMPDAYIYEPWKAPLDVQQRANCVIGRDYPPP

ILDHLEAAERNRAAMQELRRILDKAQPHCCPSSEDEIRQFMWLHEDPQLEQIANTN

>Colias_cro_CRY-1-_XP_045503844.1 XP_045503844.1

MSAAAETLPASSSRNPGPAPTSSTPPTRQPALKHTVHWFRKGLRLHDNPALREGLVDAVTFRCVFIIDPWFASSSNVGIN

KWRFLLQCLEDLDRSLRKLNSRLFVVRGQPADALPKLFREWGTTALTFEEDPEPYGRVRDHNIMTKCREVGITVISRVSH

TLYKLDQIIERNGGKAPLTYHQFQALIASMPAPPAAESAISLQNLNGATTPLTVDHDDRFGVPTLEELGFETEGLKPPVW

IGGENEALARLERHLERKAWVASFGRPKMTPQSLLASQTGLSPYLRFGCLSTRLFYYQLTELYKRIKRVRPPLSLHGQIL

WREFFYCAATRNPNFDRMVGNPICVQIPWEKNQDALAKWANGQTGFPWIDAIMIQLREEGWIHHLARHAVACFLTRGDLW

ISWEEGMKVFDELLLDADWSVNAGMWMWLSCSSFFQQFFHCYCPVRFGRKTDPNGDFIRKYIPALKNMPTRYIHEPWVSP

DSVQQAARCIIGRDYPLPMVDHTKASQINIERIKQVYAQLAKYKPQGVINQVIQRPNVMQSSPSPTSIIASINQSNYLCS

QITEVQNTTTPSAQELKEDDIFIRPNNVDTKPPLFKQVLFVHQKAKLVQQSSSCPPPKEHYLMNGELNIYKMNNTEDLQN

SNKNENCNHKNLTLSRCNANFNKNQFEKNSDTSGTYAQSIKIVEYEDEKPKLFMMSNGIVPHNNLKDNYLNSSLIKEYDA

DNKKSITKGGKIHQIVINNDRQFLGVPDKNTEQHEN

>Colias_cro_PL_XP_045496154.1 XP_045496154.1

MRINIKQGLHSYLTMASAAKKIKLSTPSSSGENKTNIVEFMNSIQKKREDTAESILKYKFNKKRLRIISHEQMVPDSCEG

IVYWMSRDSRVQDNWAFLFAQKLALKNEVPLHVCFCLIAKYLDASVRQFHFLIKGLEKVAEECKKLNISFHLLEGSGADA

LPQWVVKHKIGAVVCDFNPLRVPMGWLEGVKKKLKKDVPLIQVDAHNVVPCWIASDKQEYSARTIRNKINSKLDEFLTEF

PSVIKHPYTSKFEPEPIDWDEAIESREADKSVGPIDWAGPGYEEAMKTLKSFIDNRLKIFATKRNDPTQNALSNLSPWFH

FGQISVQRVALCVQAFKSKQTESVNAFLEEAIVRSELADNFCFYCEHYDSLKGASQWAQKTLDDHRKDKRTHIYTLDQLC

KAETHDDLWNSAQIQLVKEGKMHGFLRMYWCKKILEWTPSPEDALKYSIYMNDHYSVDGRDSNGYTGCMWSICGIHDQGW

AERAVFGKIRYMNYDGCKRKFDIKAFIARYGGKAHKYVPKK

>Colletes_gig_CRY-1-_XP_043255405.1 XP_043255405.1

MVTEERQERGKMTGSRNRDINPEAAVRGDGGKHTVHWFRKGLRLHDNPSLRESLAGASTFRCVFVLDPWFAGSTNVGINK

WRFLLQCLEDLDCSLRKLNSRLFVIRGQPAEALPKLFKEWGTTNLTFEEDPEPFGRVRDHNISTLCKELGISVVQRISHT

LYKLDEIIEKNGGKPPLTYHQFQNVVASMDSPEPPVSTVTSSCVGSAYTPLKEDHDDHYGVPTLEELGFDTEGLRPPVWV

GGESEALARLGRHLERKAWVASFGRPKMTPQSLLPSQTGLSPYLRFGCLSTRLFYYQLTDLYKKIKKAVPPLSLHGQLLW

REFFYCAATKNPNFDRMQGNPICVQIPWDKNVEALAKWANGQTGFPWIDAIMTQLREEGWIHHLARHAVACFLTRGDLWI

SWEEGMKVFDELLLDADWSVNAGMWMWLSCSSFFQQFFHCYCPVRFGRKADPNGDYIRRYLPVLKNFPTRFIHEPWNAPL

SVQRAAKCIIGQDYSLPIVNHSRSSRINIERMKQVYQQLNKYRGNGASLKGETVGLLNALPPPPVKETEEETKQKQPSPP

PETQPKMEVLDQTTQHQRQ

>Colletes_gig_PL-lik_XP_043263264.1 XP_043263264.1

MTESSPSKRRKTLDLLTKFKSNRENTAECVMTFNFKKERVRVLNSLKDVKKECKGILYWMFRDVRIQDNWALLFAQKTAL

KNNVPLHVCFCIMPSFLDASIRYYKFLLKGLEKVEEECKTLNINFHLLHGEPNIGILKFVKTFNMGAVITDFCPLKLPMS

WINDLQNNLPKDVPICQVDAHNIVPCWCASPKEEFAARTIRNKIHTKLEEFLTEFPPIVKHLYATTNKFEKNDWETALQN

VKVDTSVDEITWATPGYEHGIKELESFIQNRLKQYADKRNDPLSDATSNLSPWFHFGMISVQRCILEIQEYKKLYPKSVQ

SFIEEAVTRRELSDNFCFYNENYDLLEGAHAWAIETLTKHREDKRKYVYSLNRLENSETHDDLWNACQNQMVTTGKMSGF

LRMYWAKKILEWTATPESALETAIFLNNKYSIDGCDPNGYVGCMWSICGVHDHGWPEREIFGKIRYMNYEGCKRKFNISE

FVTKWGKEEKR

>Contarinia_nas_CRY-1-_XP_031635667.1 XP_031635667.1

MNTSACITMTDQYKVGTSKAYHDNITITPSKVVHKERHIIHWFRRGLRLHDNPALYEGLRNCTTFRCIFILDPWFADSSN

VGINKWRFLLQCLEDLDQNLKKINSRLFVVRGQATNVLPELFKEWGTTCLTFEEDPEPFGKERDQKIIKICKDLDIEVIQ

VVSHTLYKLEKIIEANGNRTPLTYNQFQCIIETMDPPPVAEMEISPELLKAATTPLTDDHEDRFGVPSLDELRFDTENLK

PPVWQGGETEALQRMERHLERKAWVASFGRPRMTPQSLLASQTGLSPYLRFGCLSVRVFYHQLSDLYKKIKKAQPPLSLH

GQLLWREFFYCAATKNGKFDRMRGNPICVQIPWEKNPEALAKWANGQTGYPWIDAIMTQLREEGWIHHLARHAVACFLTR

GDLWISWEEGMKVFDELLLDADWSVNAGMWMWLSSSSFFQQFFHCYCPVRFGRRADPNGDFIRKYLPILKNFPTRYIHEP

WNAPESIQKAAKCIIGRDYSMPMVDHSTASRTNIERLRQVYNQLYKYRDIKISKKESQIVLEMVKSTDKYVANSPVSLMD

MHMYSRKSNYKGLLDTDECQGNQLTELGNEQHHQRIRYVENDHVESVKELKYNKRVKTLIENETNRNYISHNYLTENQDE

LLEETQISDQMKQQSRQNESQHQSCYEQNIGYAPNEQQNLIFCEQVECDTRNHSNRKGLENHTDSSPTYNYIIRQENAHN

PNSKDNLIDAVQSNDVNHLAQHFNDRLQTHSHHNNTKTLLATYQPNDTFNG

>Contarinia_nas_CRY-2_XP_031630019.1 XP_031630019.1

MEKRKSVIHWFRKGLRIHDNPALIAAIDAVQKNKDYVLRPIYILDPEWTQFWRMSANKWRFLHQSLVQLDENLKTLNTRL

YVIRGTPKEEFPRLFKEWNVTLMTFEAEIEPFNQRRDALVKVNADKCNVKLEEFHSHTIYNPYYVLQSNNNEVIMRYQSF

VSLVERINVQPALEITDKHKMKLEHRPPKDTKEKANASCYDLPELNELPMDESTLGPNKFPGGENEALDRMEKMLSRKEW

ICDFEKPKTSPNSIEPSTTVLSPYFSFGCLSSRLFYHKLKAILSKKEKHTQPPVSLMGQLMWREFYYAAAAAGPNFDKMV

GNKICRQIPWKFDQELLDAWAHGKTGYPFIDAIMRQLRTEGWIHHLARHAVACFLTRGDLWISWEQGQHVFEELLLDADW

ALNAGNWMWLSASAFFYQYFRVYSPVAFGKKTDPFGNYIRKYCPELKEYPSQFIYEPWKATVGEQRRFHCEIGKDYPKRI

VIHEVAQDLNKGRMKRAYDVHNGRRDEGEPSEKVFVKEETHKPLKIEIKQEDSD

>Contarinia_nas_PL_XP_031632441.1 XP_031632441.1

MLFQSAASIYQKIKQNKSSVVHKIAPKLVDQCIRKIMAPPTKKSKTEEETNESEFLSIFEDDRKKAAKNILDFKFNKSRV

RILNKQNDVKQNSNGIIYWMFRDQRVQDNWAFLFAQKLAMKNQVPLHVCFCLLPKFLDANTRHYKFLVKGLQEIEEECNT

LDINFHLFYGDGGTEVPKFVQKSSMGAVVCDFCPLRVPMQWIENLKKSIPADIPIIQVDAHNIVPVWVTSDKQEYAARTI

RNKIVSKLSDYLTEFPPLVKHPHKSELKYSKPDWINCWKHVDIKELPEITWATPGYKGGIKQLEIFCLKRLKDYATKRND

PTLNFLSDLSPWFHFGQIAPQRAILQVSKYKKFKESVEAFREEAIVRRELADNFCFFNEHYDSLKGCTSWAQKTLNDHRK

DKREWTYTVEEFEQAKTHDDLWNSAQIQLNKEGKMHGFLRMYWAKKILEWSESPESALTTAIYLNDYYSMDGRDPNGYVG

CMWSIGGIHDQGWTERAIFGKIRYMNYAGCKRKFDVNAFVARYGGKVHQKSKK

>Copidosoma_flo_CRY-1-_XP_023245061.1 XP_023245061.1

MNTSPSCKTSDSHYNHYHHQHHQEQQYHDFFTISKTKDTWTDGIAVQGDGKKHTVHWFRKGLRIHDNPSLRDGLAGATTF

RCIFVLDPWFAGSTNVSINKWRFLLQCLEDLDRSLQKFNSRMFVIRGQPADVLPRLLKEWGTTCLTFEEDPEPYGKVRDE

NIKSMCLELGITVIQKVSHTLYKLDEIIEKNGGKSPLTYHQFQSMIIRMNPPDNPTATITAECIGCAYTPLTHNHDDFFG

VPSLEELSFDTEGLTAPVWVGGESEALTRLERHLERKAWAASFGRPKMTPQSLLASQTGLSPYLRFGCLGTRLFYHQLTD

LYKKIKKAVPPLSLHGQLLWREFFYCAATKNPNFDRMHGNPICLQIPWDKNIEAVAKWANGQTGFPWIDAIMTQLREEGW

IHHLARHAVACFLTRGDLWISWEEGMKVFDELLLDADWSINAGMWMWLSCSSFFQQFFHCYCPVRFGRKADPNGDYIRRY

LPVLKNFPTCYIHEPWNAPLSVQRAAKCIIGKEYSLPMVNHSKSSRLNIQRLKQAHQQLQKYKSYGSLITPKTPSTMYDH

QDSERNEKQ

>Copidosoma_flo_PL_XP_014214610.1 XP_014214610.1

MSEPAKKKLKTTDLVKQFEEERNQTADSVMSFKFNKKRIRILSETTDEVAKNSEGILYWMFRDARIQDNWALLFAQKIAI

KNKLPLHICYCILPKFLDATLRHYKWLLENLEEVAKGCKALNINFHLLIGEPNDVVVDFVKKYKMGAVVADFFPLRVPLF

WLDEIKKKIPSNVPVCQVDAHNIVPCWVASEKLEYAARTIRNKINSKLSEFLTEFPPVIKHPYKSDQKFKENNFATALDK

VLIDKTVDKIDWAKPGYLGAVDQLDSFIKTRLKLYSEKRNNPTINALSNLSPWFHFGMISVQRCILEVAKYKTAYKASVE

AFMEESIVRRELSDNFCFYNPNYDSIKGAYDWAAKTLDEHRKDKREYVYTLEEFEQGLTHDDLWNAAEIQLVKEGKIHGF

LRMYWAKKILEWTKSPEDALKYSIYLNDKYSMDGRDPNGYVGCMWSICGIHDQGWRERSVFGKIRYMNYEGCKKKFDVPA

FVKKYGAKVHNKKNKLNLKPSK

>Coptotermes_for_hypoth_GFG34256.1 GFG34256.1

MNAENSEKHTVHWFRKGLRLHDNPSLREGLKSAATFRCVFILDPWFAGSSNVGINKWRFLLQCLEDLDQNLRKLNSRLFV

IRGQPADALPKLFKEWGTTNLTFEEDPEPFGRVRDQNIMAMCKELGITVISRVSHTLYKLETIIEKNGGKAPLTYHQFQT

IVASMETPPPAEPTINQHFLNGAYTPIGEDHDEKYCVPTLEELGFETDGLLPPVWKGGESEALARLERHLERKAWVASFG

RPKMTPQSLLASQTGLSPYLRFGCLSTRLFYYQLTDLYKKIKKACPPLSLHGQLLWREFFYCAATKNPNFDKMSGNPICV

QIPWDRNAEALAKWANGQTGFPWIDAIMTQLRQEGWIHHIARHAVACFLTRGDLWLSWEEGMKVFEELLLDADWSVNAGM

WMWLSCSSFFQQFFHCYCPVRFGRKADPNGDYIRKYLPVLKNFPTRYIHEPWNAPEAVQKAAKCIIGKEYSLPMVNHAVA

SRINIERMKQVYQQLSKYRGLGLLATVPTSQSQTSNTLNPTLVQKDFQKSPSSENKWETLPGDDINGYSLVICDEDFKTG

IQPYCYKSRSKEEYE

>Cotesia_con_GSCOCG_CAD6229467.1 CAD6229467.1

MTGSRHNNRGRGVPSCEGIKDASITPNTTASGTGTATTTTGSATTGDRGKHTVHWFRKGLRLHDNPSLREGLNGANTFRC

VFVIDPWSAGSKSIGINKWRFLLQCLEDLDASLRKLNSRLFVIRGQPADVLPKIFREWGTTNLTFEEDPEPFGRARDHNI

TTLCRELGISVLQMVSHTLYKLDEILDKNGGKPPLTYHQFQNIVAEMDPPVPPVPTITAECIGDAFTPLREDHDDLYGVP

TLEELGFDTEGLQSSVWVGGESEALARLERHLGRKAWVASFGRPKMTPQSLLPNQTSLSPYLRFGCLSTRLFYYQLRDLY

RKIKKASPPLSLHGQLLWREFFYCAATKNPNFDRMQGNPICLQIPWDKNNEALAKWANGQTGFPWIDAIMTQLREEGWIH

HLARHAVACFLTRGDLWISWEEGMKIFDQLLLDADWSVNAGMWMWLSCSSFFQQFFHCYCPVRFGRKADPNGDYIRRYLP

VLKNYPTRYIHEPWVAPLCVQKSAKCIIGRDYSLPMVNHSKSSRINIERMKQVYQQLSKYRDNGIIANGETIGLMRIIGH

TSKPAEEQEAIKKQALSPHTSAATDDKKMITNSFDPTISPNE

>Cotesia_con_GSCOCG_CAD6234110.1 CAD6234110.1

MSEPQKKKLKSSSLFDKFNDNRQNAASSILDFKFNKNRVEVLSSVDAVAKNSKGILYWMFRDGRVQDNWSFLFAQKLALK

NRLPLHVCYCILPKFLDATLRHYKFLIESLEEVSNDCKDLNINFHLLHGVPNVVVLDLIKKHKMGALVVDFFPLRVPLGW

VENLKNSIPKDVPLCQVDAHNIVPCKVASDKLEYGARTIRSKINTKLPEYLTEYPPLIKHPHDSLFEIPTIDWKNALKDV

EIDLTVDKVDWCKPGYRGALAELESFIKNRLPHYNTKRNDPTQDALSKLSPWFHFGQISVQRVILEVKEYKKKYKESVEN

FMEESIIRRELSDNFCFHNKNYDKVEGTNAWAIESLNQHRKDKREYLYTRDELEKSQTHDDLWNAAQNQMVREGKMHGFL

RMYWAKKILEWTPSPEDALAWSIYLNDKYSMDGRDPNGYVGCMWSICGIHDQGWKERSVFGKIRYMNYKGCERKFDVKAF

VRKYDGKIVNKKNDISKIFKKK

>Cotesia_glo_CRY-1__XP_044579684.1 XP_044579684.1

MTGSRHNNRGRGVPSCEGIKDASITPNTTASGTGTATTTTGSATTGDRGKHTVHWFRKGLRLHDNPSLREGLNGANTFRC

VFVIDPWSAGSKSIGINKWRFLLQCLEDLDASLRKLNSRLFVIRGQPADVLPKIFREWGTTNLTFEEDPEPFGRARDHNI

TTLCRELGISVLQMVSHTLYKLDEILDRNGGKPPLTYHQFQNIVAEMDPPVPPVPTITAECIGDAFTPLREDHDDLYGVP

TLEELGFDTEGLQSSVWVGGESEALARLERHLGRKAWVASFGRPKMTPQSLLPNQTSLSPYLRFGCLSTRLFYYQLRDLY

RKIKKASPPLSLHGQLLWREFFYCAATKNPNFDRMQGNPICLQIPWDKNNEALAKWANGQTGFPWIDAIMTQLREEGWIH

HLARHAVACFLTRGDLWISWEEGMKIFDQLLLDADWSVNAGMWMWLSCSSFFQQFFHCYCPVRFGRKADPNGDYIRRYLP

VLKNYPTRYIHEPWVAPLCVQKSAKCIIGRDYSLPMVNHSKSSRINIERMKQVYQQLSKYRDNGIIANGETLGLMRIIGH

TSKPAEEQEAIKKQALSPHTSAATDDKKMITNSFDPTILPNE

>Cotesia_glo_PL_XP_044578578.1 XP_044578578.1

MSEPQKKKLKSSSLFDKFNDNRQNTASSILDFKFNKNRVKVLSSVDAVAKNSKGILYWMFRDGRVQDNWSFLFAQKLALK

NRLPLHVCYCILPKFLDATLRHYKFLIESLEEVSNDCKDLNINFHLLHGVPNVVVLDLIKKHKMGALVVDFFPLRVPLGW

VEDLKNSIPKDVPLCQVDAHNIVPCRVASDKLEYGARTIRSKINTKLPEYLTEFPPLIKHPHDSLFEIPTIDWKNALKDV

EIDLTVDKVDWCKPGYRGALAELESFIKNRLPHYNTKRNDPTQDALSKLSPWFHFGQISVQRVILEVKEYKKKYKESVEN

FMEESIIRRELSDNFCFHNKNYDKVEGTNAWAIESLNQHRKDKREYLYTRDELEKSQTHDDLWNAAQNQMVREGKMHGFL

RMYWAKKILEWTPSPEDALAWSIYLNDKYSMDGRDPNGYVGCMWSICGIHDQGWKERSVFGKIRYMNYKGCERKFDVKAF

VRKYDGKIVNKKNDISKIFKKK

>Cryptotermes_sec_CRY-1__XP_023706832.1 XP_023706832.1

MSEANSAKHTVHWFRKGLRLHDNPSLREGLKCATTFRCVFILDPWFAGSSNVGINKWRFLLQCLEDLDRNLRKLNSRLFV

IRGQPADALPKLFKEWGTTYLTFEEDPEPFGRVRDQNIMAMCEELGITVISRVSHTLYKLESIIEKNGGKAPLTYHQFQT

VVASMESPPPAEPALNLKFISGAYTPIGEDHDEKYGVPTLEELGFDTEGLLPPVWQGGESEALARLERHLERKAWVASFG

RPKMTPQSLLASQTGLSPYLRFGCLSTRLFYYQLTDLYKKIKKTCPPLSLHGQLLWREFFYCAATKNPNFDRMNGNPICV

QIPWNKNAEALAKWANGQTGFPWIDAIMTQLREEGWIHHLARHAVACFLTRGDLWISWEDGMKVFEELLLDADWSVNAGM

WMWLSCSSFFQQFFHCYCPVRFGRKADPNGDYIRKYLPVLKNFPTRYIHEPWNAPEAVQKAAKCIIGKEYSLPMVNHAVA

SRINIERMKQVYQQLSKYRGPGLLATVPTSQSRSPNSLNSLSVKADLKKSPSSETKLETPSDEIAVLQ

>Ctenocephalides_fel_XP_026474410.1_LOW_QUALIT XP_026474410.1

MEVDSKEEVVPGKHAVHWFRRGLRLHDNPALLEALKGADTLRCVYILDPWAARGAGANALNKWRFLLESLTDLDASLKSR

LDCRLHVVRGQPADALPALLERWGVTLLSYETDPEPYGRARDRKLAARCRDNGVEIVEYESHTLYPLDSIIEHNGGSAPV

TFEQFLGVLTTMGPPDPPAGPVRRDRLFTTRTRVPTDPDYDQSYAVPTLSELGFEANSSEPPVWRGGEAEALARLERHAE

KRTLALNSSPNFDTLPPGFLLADGAGLSPYLRFGCLSARLCYHQLADLAPATKPLLQPPPAVMPLLWREFLYCCGADNPR

FDRVQGNPLCLQTPWDRDPTALAKWANGQTGFPWIDAAMSQLRSEGWIHHLARFALIDFLTKGALFISWEEGMKVFEDLL

LDADWSTNAGSWLWLSCSSFFRKPAPSVCPVALGKLVDPDAEYIRKYVPALRGFRSARLVHAPWLASREEQRRAACRLGA

QYPLPLLVDHARTRKENARRTRLACETLPGYRNRERRLSESFQDESDIDCNCEPVIPKQQIAQDEPIMDTNIHLPDIRQE

IKSLPEFESTINSLFI

>Culex_pip_mol_CRY_2_AXG24361.1 AXG24361.1

MTKQQQQLQHSLSHRQSQKSHNSGNGGGGGGGGSGNNNSSSQQHLPKKHTVHWFRKGLRLHDNPALREGLKDAASLRCVF

VIDPWFAGSSNVGINKWRFLLQCLEDLDRNLRQLNSRLFVIRGQPADALPKLFKEWGTTCLTFEEDPEPFGKVRDHNISE

MCKELNIDVISAVSHTLYKLERIIEKNNGRAPLTYNQFQAIIASMDAPPQPEPAITLAAIGRAVTPQCDDHDDKYGVPTL

EELGFETEGLKPPIWVGGETEALARLERHLERKAWVASFGRPKMTPQSLLASQTGLSPYLRFGCLSTRLFYYQLTDLYKK

IKKAYPPLSLHGQLFWREFFYCAATKNPNFDKMAGNPICVQIPWDRNAEALAKWASGQTGFPWIDAIMTQLREEGWIHHL

ARHAVACFLTRGDLWISWEEGMKVFEELLLDADWSVNAGMWMWLSCSSFFQQFFHCYCPVKFGRKADPNGDYIRRYLPVL

KNFPTRYIHEPWNAPESVQRAARCIIGKNYPLPMVNHAIASRANMERIKQVYQQLAKYRSPSHNSSSLPASTECGTEKGG

SAIAGVMTAAKVQHMNASSLNDSPSPTTIMTSVNSSGNYMCRSNPSAQSEYQHQDQQRHHHHLHQQQQSQQLRSPGRQHS

GEHQYNNLLSHQQQHQQQQQQQPSSAYIKPENPSMAVNHNITGNSTNTANTNTTNDDLMNAHFNTLQDQLNNSMSTLGGS

SEYERNQENLYNSQFKVEYSDNFNSGYGMRNTEFYGRRRDDDPVLHFRQEKQQHHQQQQNQNLDEGSYKRPLKPLSPDGQ

QQNAIAECHRRQQQQQLNAADLQQQQNQQQPMQQSPCGEDKLSTVD

>Culex_pip_pal_CRY-1-_XP_039447540.1 XP_039447540.1

MGMTKQQQQLQHSLSHRQSQKSHNSGNGGGGGGSSGSNNSSSQQHLPKKHTVHWFRKGLRLHDNPALREGLKDAASLRCV

FVIDPWFAGSSNVGINKWRFLLQCLEDLDRNLRQLNSRLFVIRGQPADALPKLFKEWGTTCLTFEEDPEPFGKVRDHNIS

EMCKELNIDVISAVSHTLYKLERIIEKNNGRAPLTYNQFQAIIASMDAPPQPEPAITLAAIGRAVTPQCDDHDDKYGVPT

LEELGFETEGLKPPIWVGGETEALARLERHLERKAWVASFGRPKMTPQSLLASQTGLSPYLRFGCLSTRLFYYQLTDLYK

KIKKAYPPLSLHGQLFWREFFYCAATKNPNFDKMAGNPICVQIPWDRNAEALAKWASGQTGFPWIDAIMTQLRDEGWIHH

LARHAVACFLTRGDLWISWEEGMKVFEELLLDADWSVNAGMWMWLSCSSFFQQFFHCYCPVKFGRKADPNGDYIRRYLPV

LKNFPTRYIHEPWNAPESVQRAARCIIGKNYPLPMVNHAIASRANMERIKQVYQQLAKYRSPSHNSSSVPASTECGTEKG

GSAIAGVMTAAKVQHMNASSLNDSPSPTTIMTSVNSSGNYMCRSNPSAQSEYQHQDQQRHHHHLHQQQQQQQQQSQQRRS

PGRQHSGEHQYNNLLSQQQQHQQQQQQQPMQQSPCGEDKLSTVD

>Culex_pip_pal_CRY-2_XP_039432020.1 XP_039432020.1

MSSKQTAVHWFRKGLRVHDNPALAAAVDRVRGQPSKLVLRPVFILDPGIIRWLRVGPNRWRFLQQTLADLDANLRKLNSR

LYVVRGNPEEMFPELFREWNVTLLTFEHDIEPYSVKRDATVRELARQAKIEVQVEKSLTIYDPDEILKKNGGKIPLTYQK

YGSLASMCKTPGPIGVPDKVPAESVPEKDIRERKDGKCYDPPTLDELKVRQEDLGECKFPGGETEALRRLQDYMRRKSWV

CAFEKPNTSPNSLEPSTTVLSPYVKFGCLSARLFMAELKKVLAGQKHSQPPVSLVGQLMWREFYYCAAAAEPNFDKMVGN

SVCLQVPWETNPEHLAAWTHGRTGYPFIDAIMRQLCQEGWIHHLARHAVACFLTRGDLWISWEEGQRVFEELLLDADWAL

NAGNWMWLSASAFFHQFFRVYSPVAFGKKTDPEGKYIKKYVPELAKFPAGIIYEPWKANAETQKKLGCIIGKDYPHRIVI

HEEISKKNISRMSEAYRKNKALKEGGAATTKEVDSKSDAGMEPTPSGKKRKASSSSSTPKKPSPKKSKLQSKMEKFLKKK

>Culex_pip_pal_crypto_XP_039431501.1 XP_039431501.1

MTSNNILWFRHGLRLHDNPSLLEALRNDGGGQSESVRLYPIFIFDGESAGTKLVGYNRMKFLMESLDDLDRQLKAIGGQL

YIFRGNAVNVMRRLFEELNIKKLCYEQDCEPIWKERDDQIVNLCRMMDVKCVEKVSHTLWDPEQVIATNGGIPPLTYQMF

LHTVNIIGEPPRPVGAPSFEFVEFGRLPSILSTELKLFQRAPVPEDFGIYYEGNADIARQRWTGGEAKALELLGRRLKQE

EEAFREGYYLPTQARPDFLAPPSSMSAALRFGCLSVRMFYWCVHDLFARVQANNQLKHPGGHHITGQLIWREYFYTMSVH

NPHYAVMELNPICLNIPWYEAKDDSLDRWKEGRTGFPLIDAAMRQLMAEGWLHHILRNITATFLTRGGLWISWEAGVQHF

LKYLLDADWSVCAGNWMWVSSSAFEKLLDSSSCTSPVALARRLDPKGEYVKRYLPELEKFPALYVHEPWKAPPELQEQYG

CVIGKDYPAPMVNLAEVNKCNANKMNAIRQKLLDQGGSTPAHCRPSDMDEVRQFFWLPEDVAAES

>Culex_pip_pal_PL_XP_039438176.1 XP_039438176.1

MRFLPRVDCSILQKGRYFATMKKASSSKSDEPAAKKPKVEGGPIKVDDFVDKFKAIRQETAKSVLDFDFKKKRIRILSDA

KEVEEGKAGVVYWMSRDARVQDNWAFLFAQKLAMKNELPLHVCFSLVPKFLEATIRHFKFMLKGLEEVAKECESLNIQFH

MLTGMAKDTVPKFVKAHKMGAVVCDFSPLRVPAQWVEDVRKALSAEVPLCQVDAHNVVPVWVTSEKLEYAARTIRTKVNN

NLNTYLTQFPPVVKHPHKSKLKAEPIDWPKLLDTLQVDRTVDEVEWAVPGYTGGVATLQGFVEKRLRKFNAKRNDPTDDA

LSNLSPWFHFGQISVQRAILAVKKYGKGFSEGVASFCEEAIVRRELSDNFCYYNKNYDNLQGAYDWARKTLDDHRKDKRT

HVYTRDQLEQAKTHDDLWNSAQLQMVKEGKMHGFLRMYWAKKILEWTKSPEEALETAIYLNDRYQLDGRDPNGYVGCMWS

IAGIHDQGWREREVFGKIRYMNYEGCKRKFDVAAFVARYGGKVYKSK

>Culex_pip_CRY_1__AIW65406.1 AIW65406.1

EQDCEPIWKERDDRIVNLCRMMDVKCVEKVSHTLWDPEQVIATNGGIPPLTYQMFLHTVNIIGEPPRPVGAPSFEFVEFG

RLPSILSTELKLFQRAPVPEDFGIYYEGNADLARQRWTGGEANALELLGRRLKQEEEAFREGYYLPTQARPDLLASPSSM

SAALRFGCLSVRMFYWCVHDLF

>Culex_pip_CRY_2__AIW65407.1 AIW65407.1

KGLRLHDNPALREGLKDAASLRCVFVIDPWFAGSSNVGINKWRFLLLCLEDLDRNLRQLNSRLFVIRGQPADALPKLFKE

WGTTCLTFEEDPEPFGKVRDHNISEMCKELNIDVISAVSHTLYKLERIIEKNNGRAPLTYNQFQAIIASMDAPPQPEPAI

TLAAIGRAVTPQCDDHDDKYGVPTLEELGFETEGLKPPIWVGGETEALARLERHLERKAWVASFGRPKMTPQSLLASQTG

LSPYLRFGCLSTRLFYYQLTDLYKKIKKAYPPLSLHGQLFWREFFYCAATKNPNFDKMAGNPICVQIPWDRNAEALAKWA

SGQTGFPWIDAIMTQLREEGWIHHLARHAVACFLTRGDLWISWEEGMKVFEELLLDADWSVNAGMWMWLSCSSFFQQFFH

CYCPVKFGRKADPNGDYIRRYLPVLKNFPTRYIHEPWNAPESVQRAARCIIGKNYPLPMGEPRDRQPGQHGAHQTGVPTA

GPSIAAHPHNSSSLPASTECGTEKGGSAIAGVMTAAKVQHMNASSLNDSPSPTTIMTSVNSSGNYMCRSNPSAQSEYQHQ

DQQRHTTTSTNNNSRSSLGHRVVNTRA

>Culex_qui_CRY_2_AXG24360.1 AXG24360.1

MTKQQQQLQHSLSHRQSQKSHNSGNGGGGGGGGSGNNNSSSQQHLPKKHTVHWFRKGLRLHDNPALREGLKDAASLRCVF

VIDPWFAGSSNVGINKWRFLLQCLEDLDRNLRQLNSRLFVIRGQPADALPKLFKEWGTTCLTFEEDPEPFGKVRDHNISE

MCKELNIDVISAVSHTLYKLERIIEKNNGRAPLTYNQFQAIIASMDAPPQPEPAITLAAIGRAVTPQCDDHDDKYGVPTL

EELGFETEGLKPPIWVGGETEALARLERHLERKAWVASFGRPKMTPQSLLASQTGLSPYLRFGCLSTRLFYYQLTDLYKK

IKKAYPPLSLHGQLFWREFFYCAATKNPNFDKMAGNPICVQIPWDRNAEALAKWASGQTGFPWIDAIMTQLREEGWIHHL

ARHAVACFLTRGDLWISWEEGMKVFEELLLDADWSVNAGMWMWLSCSSFFQQFFHCYCPVKFGRKADPNGDYIRRYLPVL

KNFPTRYIHEPWNAPESVQRAARCIIGKNYPLPMVNHAIASRANMERIKQVYQQLAKYLSPSHNSSSLPASAECGTEKGG

SAIAGVMTAAKVQHMNASSLNDSPSPTTIMTSVNSSGNYMCRSNPSAQSEYQHQDQQRHHHHLHQQQQQQQQQSQQRRSP

GRQHSGDHQYNNLLSHQQQHQQQQQQLASSAYIKPENPSMAVNHNSTGNSTNTNTTNDDLMNAHFNTLQDQLNNSMSTLG

GSSEYERNQENLYNSQFKVEYSDNFNSGYGMRNTEFYGCRRDDDPVLHFHQEKQQHHQQQQNQNQNLDEGSYKQPLKPLS

PDGQQQNAIAECHRRQQQLNAADLQQQQNQQQQQPMQQSPCGEDKLSTVD

>Culex_qui_CRY-1_XP_038121812.1 XP_038121812.1

MTSNNILWFRHGLRLHDNPSLLEALRNDGGGQSESVRLYPIFIFDGESAGTKLVGYNRMKFLMESLDDLDRQLKAIGGQL

YIFRGNAVNVMRRLFEELNIKKLCYEQDCEPIWKERDDQIVNLCRMMDVKCVEKVSHTLWDPEQVIATNGGIPPLTYQMF

LHTVNIIGEPPRPVGAPSFEFVEFGRLPSILSTELKLFQRAPVPEDFGIYYEGNADIARQRWTGGEAKALELLGRRLKQE

EEAFREGYYLPTQARPDFLAPPSSMSAALRFGCLSVRMFYWCVHDLFARVQANNQLKHPGGHHITGQLIWREYFYTMSVH

NPHYAVMELNPICLNIPWYEAKDDSLDRWKEGRTGFPLIDAAMRQLMAEGWLHHILRNITATFLTRGGLWISWEAGVQHF

LKYLLDADWSVCAGNWMWVSSSAFEKLLDSSSCTSPVALARRLDPKGEYVKRYLPELEKFPALYVHEPWKAPPELQEQYG

CVIGKDYPAPMVNLAEVNKCNANKMNAIRQKLLDQGGSTPAHCRPSDMDEVRQFFWLPEDVAAES

>Culex_qui_CRY-2_XP_038113109.1 XP_038113109.1

MSTKQTAVHWFRKGLRVHDNPALAAAVDRVRGQPSKLVLRPVFILDPGIIRWLRVGPNRWRFLQQTLADLDASLRKLNSR

LYVVRGNPVEMFPELFREWNVTLLTFEHDIEPYSVKRDATVRELARQAKVEVQVEKSLTIYDPDEILKKNGGKIPLTYQK

YGSLASMCKTPGPIGVPDKVPAESVPEKDKRERKDGKCYDPPTLDELKVRQEDLSECKFPGGETEALRRLQDYMRRKSWV

CAFEKPNTSPNSLEPSTTVLSPYVKFGCLSARLFMAELKKVLAGQKHSQPPVSLVGQLMWREFYYCAAAAEPNFDKMVGN

SVCLQVPWETNPEHLAAWTHGRTGYPFIDAIMRQLRQEGWIHHLARHAVACFLTRGDLWISWEEGQRVFEELLLDADWAL

NAGNWMWLSASAFFHQFFRVYSPVAFGKKTDPEGKYIKKYVPELAKFPSGIIYEPWKANLETQKKLGCIIGKDYPHRIVI

HEEISKKNISRMSEAYRKNKALKEGGAATTKEVDSKSDAGMEPTPSGKKRKASSSSSTPKKPSPKKSKLQSKMEKFLKKK

>Culex_qui_PL_EDS41013.1 EDS41013.1

MFTMKKASSSKSDEPAAKKPKVEGGPIKVDDFVDKFKAIRQETAKSVLDFDFKKKRIRILSDAKEVEEGKAGVVYWMSRD

ARVQDNWAFLFAQKLAMKNELPLHVCFSLVPKFLEATIRHFKFMLKGLEEVAKECESLNIQFHMLTGMAKDTVPKFVKAH

KMGAVVCDFSPLRVPAQWVEDVRKALPAEVPLCQVDAHNVVPVWVTSEKLEYAARTIRTKVNNNLNTYLTQFPPVVKHPH

KSKLKAEPIDWPKLLDTLQVDRTVDEVEWAVPGYTGGVATLQGFVEKRLRKFNAKRNDPTDDALSNLSPWFHFGQISVQR

AILAVKKYGKGFSEGVASFCEEAIVRRELSDNFCYYNKNYDNLKGAYDWARKTLDDHRKDKRTHVYTRDQLEQAKTHDDL

WNSAQLQMVKEGKMHGFLRMYWAKKILEWTKSPEEALETAIYLNDRYQLDGRDPNGYVGCMWSIAGIHDQGWREREVFGK

IRYMNYEGCKRKFDVAAFVARYGGKVYKSK

>Cyphomyrmex_cos_PREDIC_XP_018405279.1 XP_018405279.1

MTGSSNNEMGQTVTSGVRGDGGKHTVHWFRKGLRLHDNPSLKEGLAGASTFRCVFVLDPWFAGSTNVGINKWRFLLQCLE

DLDCSLRKLNSRLFVIRGQPADALPKLFKEWGTTNLTFEEDPEPFGRVRDHNISALCKELSISVVQRVSHTLYRLDEIIE

RNGGKPPLTYHQFQDVVAGMDSPEPPVPTVTAACIGSAYTPLKDDHDDHYGVPTLEELGFDTESLLPPVWVGGESEALAR

LERHLERKAWVASFGRPKMTPQSLLPSQTGLSPYLRFGCLSTRLFYYQLTDLYKKIKKAVPPLSLHGQLLWREFFYCAAT

KNPNFDKMQGNPICVQIPWDKNVEALAKWANGQTGFPWIDAIMTQLREEGWIHHLARHAVACFLTRGDLWISWEEGMKVF

DELLLDADWSVNAGMWMWLSCSSFFQQFFHCYCPVRFGRKADPNGDYIRRYLPMLKNFPTRYIHEPWNAPLSIQHAAKCI

IGKEYSLPMVNHSKSSRINIERMKQVYQQLNKYRGNGTSFKGENIGLLNALLAPPAKDSDEEKRKQDSPNRENEQKMETI

SSPTQQQQQQQQQ

>Daktulosphaira_vit_CRY-1_XP_050537923.1 XP_050537923.1

MSIGIHWYRHGLRLHDNPALVEAAKKTNKLITLYIFDGKIEGDQQIGYNRMRFLLESLKDLDDNLKLRGGCLYILQGNPV

DIFEKIKNEVGLNLISFEQDCEPIWKKRDDAVKRFSRENNIECIEKVSHTLWDPKLIIQNNGGVAPLTFENFLSITSKIG

NPPRPVPHVDWLSVNFAELPTSVLKKFKALNNPSPEYFNMYPEVPEIHSSHNRLYGGETKALEQLMVRLEFEKEAFINGF

YLPNQVNPDLLGPPSSMSAALRLGCLSIRKFYWELSKLFMQTFEEDLLPQHSATSQLIWRDYFYVMSVDNDQFDQIENNP

ACIKIPWGDLELEKNRMHLNCWKYGKTGYPFIDAGMRQLLQEGWIHHVVRNSVACFLTRGDLWISWTEGFKHFIKFLLDG

DWAVCSGNWIWVSSSTFEQILDCPLCVCPVSYGKRLDPTGEYVRRYVPELRKLPDKYLFEPWKCPIEIQKKVGCIIGKDY

PHRIVDHQNASCENRKKMQELRSSLMNENSIPHCRPSNTTELKKFMYLSNDCMEELCMNLHSEYSNIY

>Daktulosphaira_vit_CRY-1_XP_050541619.1 XP_050541619.1

MNFEQPSKHTVHWFRKGLRLHDNPSLRDGLVGSKTFRCIFILDPWFAGASNVGINKWRFLLQCLEDLDNSLKELNSRLFV

IRGQPAEALPKLFKMWGTTNFTFEEDPEPYGRVRDENISAMCREMGISVITRVSHTLYKLDKIINMNGGKAPLTYHLFQK

LLEYIDPPDDAVPRVDKDFVGDCITPIEFDHDDKFGVPTLEELGFEDVDNTKVQYIWKGGETEALIRLQRHLERKAYIAS

YGKPKMTPQSLLASRTGLTPYLRFGCLSTRLFFSELNNLYRKIRKAQPPLSLHGQLLWRDFFYCASTNNPNFDKMIGNPI

CVQIPWNTNIQALSKWANGQTGYPWIDAIMIQLRNEGWIHCIARHAVACFLTRGDMWLSWEEGMKVFEELLLDADWSVNA

GSWMWYSCSSFFQEFIHCYCPVRFGRKADPNGDYIRKYIPVLKNIPNKYIHEPWLAPESVQKSAKCVIGIDYPQPIVNHV

VASKINLERMKLAYQQLANCQPRLENGKLILISDPTIYCEKG

>Daktulosphaira_vit_LOW_QU_XP_050523060.1 XP_050523060.1

MLIEIKYIIGQISVQRCILEVSKFSSKYSESVAAYRKEAIVRRELSDNFCFYNPKYDSIKSAANWAQTTLNEHREDKRKY

VYTREEHESLQTHDDLWNSAQIQLVKEGKMHGFLRMYWSKKILEWTDTPEXALAESIYLNDKYSMDGRDPSGFVGSMWSI

CGIHDQGWREREIFGKITYMNYDGCKRKFDVNAFVTRYGGKVHKYVKK

>Danaus_chr_unname_CAG9560692.1 CAG9560692.1

MTKVASVIHWFRLDLRLHDNLALRNAINEAENRKQILRPIYVIDPDIKNRVGCNRLRFLFQSLKNLDTSLRKINTRLYVI

KGKAVECLPKLFDEWHVKFLTLQVDIDADLVKQDEVIEEFCEANNIFVVKRMQHTVYDFNSVVKKNNGSIPLTYQKFLSL

VSDVQVKDIIQISKSVSDECKGNDYDSQDYDVPSLEEFGINESELSECKYPGGESEGLKRLDVYMAKKQWVCKFEKPKSS

PNSIEPSTTVLSPYISHGCLSAKLFYHKLKQVESGSKHTLPPVSLMGQLMWREFYYTAGSGTKNFDKMEGNSVCTQIPWK

KNDAHLKAWAEGKTGYPFVDAIMRQLKQEGWIHHLARHMVACFLTRGDLWISWEEGAKVFEDYLLDYDWSLNAGNWMWLS

ASAFFYKYYRVYSPIAFGKKTDKDGLYIRKYVPELKKYPSEFIYEPWKAPKGVQKTAGCVIGEGYPNRIVDHDKVHKENI

QKMNAAYKINKEKKAMKRPRQ

>Danaus_chr_unname_CAG9575816.1 CAG9575816.1

MKHWKSFIESIIVGCDDGNVIMSVAETVPLRARSPTTQKTQPASVPKEKHTVHWFRKGLRLHDNPALREGLVDATTFRCV

FIIDPWFASSSNVGINKWRFLLQCLEDLDKNLRKLNSRLFVVRGQPADALPKLFREWGTTALTFEEDPEPYGRVRDHNIM

TKCREVGIQVISRVSHTLYKLDDIIEKNGGKAPLTYHQFQALIASMPPPPSAEPTITLETLNRAVTPISDNHDERFGVPT

LEELGFDTEGLKPPIWIGGENEALLRLERHLERKAWVASFGRPKMTPESLLSSQTGLSPYLRFGCLSTRLFYYQLSELYK

RIKQERPPLSLHGQILWREFFYCAATRNPNFDRMEGNPICVQIPWEKNQEALKKWANGQTGFPWIDAIMIQLRNDGWIHH

LARHAVACFLTRGDLWISWEEGMKVFDELLLDADWSVNAGMWMWLSCSSFFQQFFHCYCPVRFGRKTDPNGDFIRHYIPV

LKNMPTRYIHEPWVCPEEIQKSIRCIIGKDYPMPIVDHTKASEINLERIKQVYAQLAKFKPQGALIPQMLQRPNVLQSSP

SPTSIIANINQSNYLCSQNNDVPTPTNQTTNQFKEDAVFLKPTVNNIKSNVDKQQQFKQVVIVQEDKHAENQRHSVDNKY

IVNEINKNINDIPVKQNNYDFKALTLNLNKFSNEPLTFLNQTPNKNESFGQDVNNVIDVYSTSKPKFYFTENGVITHNDN

AQTFKRDSYSDNYNKESTESNRVSEVHSNPQTDKISSEKKN

>Danaus_chr_unname_CAG9581750.1 CAG9581750.1

MKIRFLTHFTTKMASSAKKPKLSLPIKNESSTINVDDFMKQIHKKREETAKSILDFNFNKSRIRIISQEQMVSDDCEGIV

YWMSRDSRVQDNWAFLFAQKLALKNKVPLHVCFCLIAKYLDASVRQFHFLIKGLEKVAADCEKLNISFHLLEGSGAEVLP

QWVIDHKIGAVVCDFNPLRVPLGWVEGAKKKLKKDVPLIQVDAHNVVPCWVASNKQEYSARTIRNKINSKLDEYLTEFPP

VIKHPHSSSFKPEPIDWDKAIETREADKSVGPIEWAGPGYDNAVKTLKSFLDKRLKVFATKRNDPTQDALSNLSPWFHFG

QISAQRVALCVKEYKGKYTESVNSYLEEAIVRRELADNFCFYCEHYDSIKGASQWAQKTLDDHRNDKRTHIYTLEQFCKA

ETHDDLWNSAQIQLVKEGKMHGFLRMYWCKKILEWTSSPEDALKYAIYLNDHYSVDGRDPSGYVGCMWSICGVHDQGWAE

RTVFGKIRFMNYEGCKRKFNVPAFVCRYGGKVHKYNNLMEKQKKK

>Danaus_chr_unname_CAG9584912.1 CAG9584912.1

MSYNILVISSTSMLGGNVIWFRHGLRLHDNPSLHSALEDPSAPFFPIFIFDGETAGTKMVGYNRMRYLLEALNDLDQQFR

KYGGKLLMIKGRPDLIFRRLWEEFGIRTLCFEQDCEPIWRPRDASVRALCRDIGVSCREHVAHTLWNPDTVIKANGGIPP

LTYQMFLHTVEIIGNPPRPVDDVDLNGVNFGSLPESFYREFTVFDKAPKPEDLGVFLENEDIRMIRWVGGETAALKQMQE

RLAVEYETFCRGSYLPTHGNPDLLGPPISLSPALRFGCLSVRRFYWSLQDLFQQVHQGRLASTQFITGQLIWREYFYTMS

VNNPNYAQMSGNPICLDIPWKEPENDELQRWKEGRTGFPFVDAAMRQLRTEGWLHHVVRNTVASFLTRGTLWLSWEHGLQ

HFLKYLLDADWSVCAGNWMWVSSSAFEALLDSGECACPVRLGRRLEPSGHYVRRYVPELARMPGEYIYEPWRAPIEVQEA

AGCIIGRDYPAPVVDHTAAAARNRANMQELRRLLEKAPPHCCPSSEDEVRQFMWLGDDSQPELTTT

>Danaus_ple_ple_OWR51555.1_KGM_210113 OWR51555.1

MQHTVYDFNSVVKKNNGSIPLTYQKFLSLVSDVQVKDIIQISKGVSDECKASDYDSQGYDIPSLEEFGVNESELSECKYP

GGESEGLKRLDVYMAKKQWVCNFEKPKSSPNSIEPSTTVLSPYISHGCLSAKLFYHKLKQVENGSKHTLPPVSLMGQLMW

REFYYTAGSGTENFDKMVGNSVCTQIPWKKNDAHLKAWAEGKTGYPFVDAIMRQLKQEGWIHHLARHMVACFLTRGDLWI

SWEEGAKVFEDFLLDYDWSLNAGNWMWLSASAFFYKYYRVYSPVAFGKKTDKDGLYIRKYVPELKKYPSEFIYEPWKAPK

GVQKTAGCVIGEGYPNRIVDHDKVHKDNIQKMNSAYKVNKEKKAMKRPRQ

>Danaus_ple_ple_XP_032522602.1_cry-1 XP_032522602.1

MLGGNVIWFRHGLRLHDNPSLHSALEDASSPFFPIFIFDGETAGTKMVGYNRMRYLLEALNDLDQQFRKYGGKLLMIKGR

PDLIFRRLWEEFGIRTLCFEQDCEPIWRPRDASVRALCRDIGVSCREHVAHTLWNPDTVIKANGGIPPLTYQMFLHTVEI

IGNPPRPVDDVDLNGVNFGSLPESFYREFVVFDKAPKPEDLGVFLENEDIRMIRWVGGETAALKQMQERLAVEYETFCRG

SYLPTHGNPDLLGPPISLSPALRFGCLSVRRFYWSLQDLFQQVHQGRLASTQFITGQLIWREYFYTMSVNNPNYAQMSGN

PICLDIPWKEPENDELQRWKEGRTGFPFVDAAMRQLRTEGWLHHVVRNTVASFLTRGTLWLSWEHGLQHFLKYLLDADWS

VCAGNWMWVSSSAFEALLDSGECACPVRLGRRLEPTGHYVRRYVPELARMPGEYIYEPWRAPLEVQEAAGCVIGRDYPAP

VVDHTAAAARNRANMQELRRLLEKAPPHCCPSSEDEVRQFMWLGDDSQPELTTT

>Danaus_ple_ple_XP_032529216.1_photolyase XP_032529216.1

MKIKFLTHFTTKMASSAKKPKLLLPIKNESSTINVDDFMKQIHKKREETAKSILDFNFNKSRLRIISQEQMVSDDCEGIV

YWMSRDSRVQDNWAFLYAQELALKNKVPLHVCFCLIAKYLDASVRQFHFLIKGLEKVAADCDKLNISFHLLEGNGAEVLP

QWVIDHRIGAVVCDFNPLRVPLGWVEGAKKKLKKDVPLIQVDAHNVVPCWVASNKQEYSARTIRNKINSKLDEYLTEFPP

VIKHPHSSSFKPEPIDWDKAIETREADKSVGPIGWAGPGYDNAVKTLKSFLDKRLKVFATKRNDPTQDALSNLSPWFHFG

QISAQRVALCVKEYKTKYTESVNSYLEEAIVRRELADNFCFYCEHYDSIKGASQWAQKTLDDHRNDKRTHIYTLEQFCKA

ETHDDLWNSAQIQMVKEGKMHGFLRMYWCKKILEWTSSPEEALKYAIYLNDHYSVDGRDPSGYVGCMWSICGVHDQGWAE

RAVFGKIRFMNYDGCKRKFNVPAFVCRYGGKVHKYNNLTDKQKKK

>Danaus_ple_XP_032511499.1_cry-1-like XP_032511499.1

MSVAETLPLRARSPTAQKSSQPAGVPKEKHTVHWFRKGLRLHDNPALREGLVDATTFRCVFIIDPWFASSSNVGINKWRF

LLQCLEDLDKNLRKLNSRLFVVRGQPADALPKLFREWGTTALTFEEDPEPYGRVRDHNIMTKCREVGIQVTSRVSHTLYK

LDDIIEKNGGKAPLTYHQFQALIASMPPPPSAEPTISLETLNRAVTPISDNHDERFGVPTLEELGFDTEGLKPPIWIGGE

NEALLRLERHLERKAWVASFGRPKMTPESLLSSQTGLSPYLRFGCLSTRLFYYQLSELYKRIKQERPPLSLHGQILWREF

FYCAATRNPNFDRMEGNPICVQIPWEKNQEALKKWANGQTGFPWIDAIMIQLRNDGWIHHLARHAVACFLTRGDLWISWE

EGMKVFDELLLDADWSVNAGMWMWLSCSSFFQQFFHCYCPVRFGRKTDPNGDFIRKYIPVLKNMPTRYIHEPWVCPEEIQ

KSIRCIIGKDYPMPIVDHTKASEINLERIKQVYAQLAKFKPQGALIPQMLQRPNVLQSSPSPTSIIANINQSNYLCSQSS

DVPTPTNQTTNQFKEDAVFLKPTVNNIKSNVDKQQQFKQVVIVQEDKHSENQRHSVGNKYIVNEINKNINDIPVKQNNYD

FKALTLNLNKFSNEPLTFLNQTPNKNESFGQDVNNVIDVYSTSKPKFYFTDNGVITHNENAQTFKRDSYSDNYNKESTGS

NRVGEVHSNNPQTDKISSEKKN

>Dendroctonus_pon_CRY-1_XP_048521261.1 XP_048521261.1

MNVDVLESAAHQEGKHAVHWFRKGLRLHDNPSLREGLKGATTFRCVFVLDPWFASSSNVGINKWRFLLQCLDDLDRSLKK

LDSRLFVIRGQPADALPKLFREWGTTALTFEEDPEPFGRVRDHNITTLCKEMGITVDQKDSHTLYQLQNIIDRNGGKAPL

TYHQFLAIIARMGPPPQPEPSVTTHSVHNAHTPLSDDHDEKYGVPTLQELGFDIEGLNPPVWQGGETEALTRLERHLERK

AWVASFGRPKMTPQSLLPSQTGLSPYLRFGCLSTRLFYYQLTDLYKKIKKAFPPLSLHGQLLWREFFYCAATKNPNFDKM

LGNPICVQIPWDKNAEALAKWANGQTGFPWIDAIMTQLRQEGWIHHLARHAVACFLTRGDLWISWEEGMKVFEELLLDAD

WSVNAGTWMWLSCSSFFQQFFHCYCPVKFGRKADPNGDYIRKYLPMLKNMPTQYIHEPWMAPDNVQKGAKCTVGLHYPLP

MVNHITVSRLNIQRMKQVYQQLSNYRPSELSSNCSYLKKPAKPDHFKYHHQAKF

>Diabrotica_bal_unname_CAG9829534.1 CAG9829534.1

MSGSVCHTSTGKEKHTVHWFRKGLRLHDNPSLKEGLKGAKTFRCVFVLDPWFAGSSSVGINKWRFLLQCLEDLDRNLRKL

NSRLFVIRGQPADALPKLFKEWGTTALTFEEDPEPFGRVRDHNITALCNELGITVVQHVSHTLYHLQHIIDRNGGKAPLT

YNQFLAIIASMGPPPKPELPVNASVLNGAYTPLSDDHDEKYGVPTLEELGFDTEGLNPPVWQGGESESLSRLERHLERKA

WVASFGRPKMTPQSLLPSQTGLSPYLRFGCLSTRLFYYQLTDLYKKIKKAFPPLSLHGQLLWREFFYCAATKNSNFDKMF

GNPICVQIPWDKNAAALAKWANGQTGFPWIDAIMTQLRQEGWIHHIARHAVACFLTRGDLWISWEEGMKVFEELLLDADW

SVNAGSWMWLSCSSFFQQFFHCYCPVKFGRKADPNGDYIRKYLPVLKNMPTQYIHEPWLASEKIQQASKCIIGKDYPLPM

VNHGVASRINIQRMRQVYQQLANYKMVENARFEVKEQQIRENSIIDSAV

>Diabrotica_bal_unname_CAG9834436.1 CAG9834436.1

MASLKPRNSSGKILLDQLTKEYFIKNIVSAREAQGESVENFNFNKDRCRVLSPNENIKDGSKGILYWMYRDCRVQDNWAL

IFAQRLAIKKKLPLYVCYSLKDAHQQYPTQRHFNFFIEGLKFVQKEFRQLNIGFHLLNASPKELAKLIVSNDIGGVVCDF

SPLKHPRKLQTALLDSLPDEVPLVQVDAHNIVPVWIASCKQEGMAKFLRPKITQKLDEYLTGFPNISKHKYSGKLNIQNE

IELDEALNCYTPKYDVPEIKWGDGPGPEAGLTMLRKFIVENLREYGSTSNDPSKDNTSKLSPWINFGQISAQRCALEVKS

VSSLFKEQCDKYLEELIVRRELTDNFCYYNSNYDNLNGAANWAKESLKLHSNDKRIWTYTKKELENAETHDEMWNAAQLQ

AHQEGKIHGYMRMYWCKKILEWTESPEQAIEYGLWLNDTFCLDGTDPNGYVGVMWSICGIHDQGWKERDIFGKIRYMVDY

SLRRKFNMDAYCARYGRKILTDSKKKRAASISSDDKKGKNKKRVR

>Diabrotica_vir_vir_CRY-1-_XP_028129721.2 XP_028129721.2

MSGSVCHTSTGKEKHTVHWFRKGLRLHDNPSLKEGLKGAKTFRCVFVLDPWFAGSSSVGINKWRFLLQCLEDLDRNLRKL

NSRLFVIRGQPADALPKLFKEWGTTALTFEEDPEPFGRVRDHNITALCNELGITVVQHVSHTLYHLQHIIDRNGGKAPLT

YNQFLAIIASMGPPPKPELPVNASVLNGAYTPLNDDHDEKYGVPTLEELGFDTEGLNPPVWQGGESESLSRLERHLERKA

WVASFGRPKMTPQSLLPSQTGLSPYLRFGCLSTRLFYYQLTDLYKKIKKAFPPLSLHGQLLWREFFYCAATKNSNFDKMF

GNPICVQIPWDKNAAALAKWANGQTGFPWIDAIMTQLRQEGWIHHIARHAAACFLTRGDLWISWEEGMKVFEELLLDADW

SVNAGSWMWLSCSSFFQQFFHCYCPVKFGRKADPNGDYIRKYLPVLKNMPTQYIHEPWLASEKIQQASKCIIGKDYPLPM

VNHGVASRINIQRMRQVYQQLANYKMVENARFEVKEQQIREISIIDSAV

>Diabrotica_vir_vir_PL_X1_XP_028131050.2 XP_028131050.2

MASLKPRNSNGKILLDQLTKEYFIKNILWAREAQGESVENFEFNKDRCRVLSPNENIKDKSKGILYWMYRDCRVQDNWAL

IFAQRLAIKKKLPLHVCYSLKDAHEQYPTQRHFNFFIEGLKFVQKEFQQLNIGFHLLNVSPKELAKLIASNDIGGVVCDF

SPLRHPKKLQTELLKSLHNEIPVVQVDAHNIVPVWIASDKQEGMAKFLRPKISKNLDEYLTGFPNISKHKYSGKLNLQNE

IELDQAVNYYTPKYDVPEIKWGDGPGPEAGLTMLRKFIVENLREYGNTSNDPSKDNTSKLSPWINFGQISAQRCALEVKS

VSSLFKEQCDKYLEELIVRRELTDNFCYYNSNYDNLNGAAKWAQETLKVHSKDKRTWTYTRKQLENAETHDEMWNAAQLQ

AHQEGKIHGYMRMYWCKKILEWTESPEQAIEYGLWLNDTFCLDGTDPNGYVGVMWSICGIHDQGWKEREIFGKIRYMVDY

SLRRKFNMDAYCARYGRKILTDSKRKRDASSGSDDKKGKNKKKAR

>Diachasma_all_CRY-1_XP_015118990.1 XP_015118990.1

MAVHRRIDKSQFISKLSAAPPDAKHVVHWFRKGLRLHDNPSLRCGLNGACTFRCIFVIDPWSAGSKSIGINKWRFLLQCL

EDLDTSLRKLNSRLFVIRGQPTDALPKLFREWGTTHLTFEEDPEPFGRARDYNITTLCKELRISVASIASHTLYKLDKIL

EKNKGKAPLTYHQFQTIVQGMDPPDPPVAIVTAETIGTAYTPLQEDHDDIYGVPTLEEIGFDTEGLQPPVWVGGESEGLI

RLERHLERKAWVASFGRPKMTPQSLLPSQTSLSPYLRFGCLSTRLFYYQLRDLYKKIKKTMPPLSLHGQLLWREFFYCAA

TKNPNFDRMQGNPICLQIPWDKNAEALAKWANGQTGFPWIDAIMTQLREEGWIHHLARHAVACFLTRGDLWISWEEGMKK

IR

>Diachasma_all_PL_XP_015114233.1 XP_015114233.1

MEPPKKKAKTLGLFEKFIENRRKTADSIMDFPMNKKRIRVLSNAKEVVKNSKGILYWMFRDPRVQDNWALLYAQKLALKN

RLPLHVCYCILPKFLDATLRHYKFLVESLEEVSNDCKELNINFHLLLGEPNSVVLDFVKKYNIGALVIDFFPLRLPRFWV

EDIGKKLPEDVPLCQVDAHNIVPCWQASDKLEYAARTIRSKINSKLPEYLTEFPPVIKHPHDSDFEFPKIDWKNSLNEVL

IDKTVDKVEWCKPGYRGAVGQLETFITERLKNYNEKRNDPVQDATSGLSPWFHFGQISVARVILEVQEYQKNYQASVNSF

IEEAVVRRELSENFCFYNENYDKIEGANAWAIQSLNAHRKDKREWVYTLEEFEKSQTHEDLWNAAQNQLVRDGKIHGFMR

MYWAKKILEWSPTPEDALAWSIYLNDKYSMDGRDPNGYVGCMWSICGIHDQGWREREVFGKIRYMNYKGCERKFDVKAYV

KKFDGKIVNKKKKETAEKLPKKSKK

>Dianemobius_nig_BAF45421.1_cry_precur BAF45421.1

MDNRSNVHKKVAVHRFRHGLRLHDNPALLDAVKDCDAFLPIFIFDGESAGTKLVGYNRMKFLLESLQDIDSQLKKYGGNL

YLFHGTPLCVFQYISQTIGLHKLCFEQDCEPIWQHRDDLVKKFCKENGIKCIERVSHTLWNPHDVIKTNGGIPPLTFEMF

VHTVSVIGPPPRPVEDVEWSVVNFGVLPMSSIPSDIKVFKNFPTPEDFGISSEVGNMNRIIQWIGGESQALRHLQERLKV

EENAFREGYCLPNQARPDLLGPPTSQSAALRFGCLSVRKFYWSIQDMYSSICGPSPNQNITSQLIWREYFYTMSVGNEYY

AEMDRNPICLNIPWKNDYGSDFNKWKEGKTGYPFIDAIMRQLIQEGWIHHVARNAVACFLTRGDLWISWEEGLNFFLQYL

LDADWSVCAGNWMWVSSSAFEQLLDCSHCMCPVNYGRRLDPWGQYIKRYIPELKNYPVEYLYEPWKAPLHVQETAGCIVG

KDYPERIIDHQIASEKNRSYMDEIRNRLMNPPPHCRPSSEKETRQFMWFPDDCSEHSSQ

>Dianemobius_nig_BCX29417.1_cry_2,_par BCX29417.1

FGRVRDQNIMAMCREMGISVISRVSHTLYRLESIIEKNGGKAPLTYHQFQTVVASMDSPPPAEPRISARTTDGVHTPVTD

DHDDRFGVPTLEELGFDTEGLLPPVWTGGESEALARLERHLERKAWVASFGRPKMTPQSLLASQTGLSPYLRFGCLSTRL

FYYQLTDLYKKIKKACPPLSLHGQLLWREFFYCAATNNSNFDRMNGNPICVQIPWDKNPEALAKWATGQTGFPWIDAIMT

QLREEGWIHHLARHAVACFLTRGDLWISWEEGMKVFEELLLDADWSVNAG

>Diaphorina_cit_hypoth_KAI5692562.1 KAI5692562.1

MSPLSAPTNPNSTEKHMVHWFRKGLRMHDNPSLREGLKGCTTFRFLLQCLEDLDINLRKLNSRLFVIRGQPADILPKLFK

EWKTTCLTFEEDPEPFGKVRDQNIMTLCRELNIEVIARVSHTLYDLDQIIEKNGGKTPLTYHQFQSIVAKMDSPSPAEAP

VTPLLVRQATTPLRDDHDEKYGVPTLEELGFDIEGLLPPTWKGGETEAMRRLERHLERKAWVASFGRPKMTPQSLLASQT

GLSPFLRFGCLSTRLFYHDLNKLYKRIKKAPPPLSLHGQLLWREFFYCAATRNPNFDRMLGNPICVQIPWDVNMEALAKW

ANAQTGFPWIDAIMTQLREEGWIHHLARHAVACFLTRGDLWVSWEEGMKIFDELLLDADWSVNAGMWMWLSCSSFFQQFF

HCYCPVKFGRKADPNGDFIR

>Diaphorina_cit_hypoth_KAI5697792.1 KAI5697792.1

MSTINSSEPPTKKSKVVKPSDYVKPSSSSNISSDFIKTLEKHRKSTAGSISEFKFNKKRAKVLSEAQEFPEWGEGVIYWM

FRDERVHDNWALLFAQKLALKNKVPLHVCFCRLKKFLDCSLRHYKFIFDGLKEVHEDCKKLNIEFHFLIGGAHEILPQFV

EKHKLGAVVIDFMPLREHMGWADTLKKDLPKDVPLIQIDAHNIVPCWIASDKQEYGARTIRNKINSKLPEFLTEFPPVIK

HPYSGKLKAETIDWEAAEESLQVDKTVGPVAQIKSGYRAALATLESFVNKRISLYETARNDPTKNALSNLSPYFHFGQLS

VQRAVLEVRRVLPKHSKAVDSFCEEAIVRRELADNFCYYNKNYDKVEGAFDWAKKTLNDHRKDKREYLYSRSELEEALTH

DDLWNSAQIQLTKHAKMHGFLRMYWAKKILEWTKTPEEALSTAIYLNDKYSMDGRDPSGFVGCMWSICGIHDQGWAERAV

FGKIRYMNYAGCKRKFDVAAFIARYGGKVHKQWNLFEPRYFCKFSKLKAQALNNNGDSPRHQAALSEAKKKDRILYVLHA

VGAERCESGKSGCNDGCRYGGSYDGTPPPAPLTDQVRGIIDFYPDISELRKSSNKGGRVLCLDGGGIKGVVLIAQLMALE

KLTQGRSILECFDWIAGTSTGGILASGLAAGKPLRELMNTYFLIKDKVFGLTRPFDDEELRNILKEILGPDTVMSEVQTT

KLLITGTLCDQSPVKLHLFRNYPAPSTLVAPNAPADRDPPLADHTQQIIWQAARSSGAAPTYFE

>Diaphorina_cit_hypoth_KAI5727476.1 KAI5727476.1

MDTSNSPNNKEVAVHWFRHGLRLHDNPALFEASLNSVLYPVFIFDGETAGTMHIGYNRFRFLLECLADLDRQLKSHGGQL

FIVQGSPISIFQKLKRELNFTKLCFEQDCEALWHKRDKKVKKWCAENNITVKEFVSHTLWDPEVVIQTNGNVPPLTYKMY

LHTVSCIGLPPRPKEDIDFRHVTFGTMSESLQRDVSLFQTVPKPEQFHKYPEMDFGDPLIRWLGGETEALIKLNERLSQE

IESFKSGVYLSNQVSPDLTGPPTSQSAALKFGCLSVRRFYWALHDHFNTIHEGRPPSHFNITGQLIWREYFYTMSAHNPY

YDQMEKNPICLNIPWLPESHPNKEKYLNAWKNGQTGYPFIDAVMRQLRREGWVHHVARNAVACFLTRGDLWINWEDGVRH

FFYYLLDADWSVNAGNWMWVSSSAFEQLLDCTYCVCPVNFGRRLDPDGIYIKRYVPELRQFPIQYIYEPWKAPLGVQEKA

NCIISKDYPERIVNHVQASLENKQKMLEIRNSLLEETPHCRPADTEEVYQFMMFPENCNDHLCANLF

>Diaphorina_cit_hypoth_KAI5736374.1 KAI5736374.1

MGGTPECAVHWIRKGMRLHDNPALLSAINYKNEKGQNILLKPLYILDPHFRKFMRVGPNRWRFLQQSLADLDQKFRALGS

RLYVVQGKPEEVFPDIFKTWNIKLLTWEYDIEPYAKKRDGLVEDMAKEYKVKVEQHVSHTLYNTNLVIKANGGKPPMTYQ

KLVSVLESLPKPKPADDAPTSLPRECQGILHSDEHLVPTMKEMGLDESSIPLCKFPGGETEALKRLEKSLANKEWVRKFE

KPNTAPNSLEPSTTVLSPYLKFGCLSVRLFYHELKKILATGPHAKPPVSLLGQIYWREFYYVVGSDTPNFDKMKGNKICC

QVDWDTNEKYLEAWSHGKTGYPFIDAIMRQLRLEGWIHHLARHAVACFLTRGDLYLSWEEGQSVFEELLLDADWAMNAGN

WMWLSASAFFHQFFRVYSPVAFGKKTDKFGEYIKKYVPELKKYPVDYIYSPWEAPLSIQKTAGCVIGVDYPKRIVIHEDI

HKKNIARLSAAYKKNKANASAGEKGSDSSTSSSPARKSDSKSKRKSSTQTTLNFTKKKKS

>Diatraea_sac_unname_CAG9784866.1 CAG9784866.1

MASVVHWFRLDLRIHDNLALRNAINEAENRKYLLRPVFIIDTDIKQKIGANRLRFLIQSLQDLDINLRKLNSRLFILRGN

AKELFSKLFDEWQVKYLTTQVDIDPEIVQQDEIIEKLAESKDIYIVRRVQHTVYDVHSVLKKNNGNVPLTYQKFLSLVND

INVKDTIEITKTLSDHCKPSDLESNNYNVPTLQELGIDESSLNPCKYPGGEIEGLKRLHLYMAKKQWVCKFEKPNTSPNS

IEPSTTVLSPYLSHGCLSSKLFYHKLKQVENGVPHSQPPVSLLGQLMWREFYYTAGAGTENFDKMVGNPVCTQIPWVKNE

NYLKAWAEGRTGYPFVDAIMRQLKQEGWIHHLARHMVACFLTRGDLWISWEEGAKVFEDYLLDYDWSLNAGNWMWLSASA

FFYKYFRVYSPVAFGKKTDKEGLYIRKYVPELKKYPTEFIYEPWKAPKSVQKTAGCIIGEDYPQRVVDHDKIHKENMQKM

SAAYKNNKEKKSLKRKLSTN

>Diatraea_sac_unname_CAG9785938.1 CAG9785938.1

MLGGSVLWFRHGLRLHDNPSLLSALEDRRMPLFPIFIFDGETAGTKLVGYNRMRYLLEALDDLDCQFKKFGGRLIMLKGK

PNIVFRRLWEEFGIRKLCFEQDCEPVWRARDDSVKNECREIGVKCVEHVSHTLWEPDMVIKANGGIPPLTYQMFLHTVAT

IGDPPRPVMEADLCGVKFGSLPQCFYEEFTVFDKTPKPEDFGVFLENEDIRMIRWVGGETAALKQMQQRLNVEHETFCRG

SYLPTHGSPDLLGPPISLSPALRFGCLSVRRFYWSMQDLFRKVHQGRLSSTHFITGQLIWREYFYTMSVNNPNYGQMAGN

PICLDIPWKNPEGDELQRWREGRTGFPFVDAAMRQLRVEGWLHHAVRNTVASFLTRGTLWLSWEHGLDHFLKYLLDADWS

VCAGNWMWVSSSAFEALLDAGECACPVTLGHRLDPSGEYVRRYVPELGRLPALYIYEPWKAPIDVQERANCIIGKDYPAP

VVNHLLAAQRNRLAMKELRELLQKAPPHCCPSSDDEIRQFMWLNEENQPTNAIATN

>Diatraea_sac_unname_CAG9796370.1 CAG9796370.1

MRFFSIKTFCKMASAAKKPKLSTLASVNTEETTTDLNSFMQKIQAKREETAESILHYKFNRKRLRIISQEQTVSDNCEGI

VYWMSRDSRVQDNWAFLFAQKLALKNEVPLHVCFCLISKYLDASVRQFHFLVKGLEKVAAECKKLNISFHLLEGSGAEAL

PQWVADHKIGAVVCDFNPLRVPLGWLEGCKKKLKKDVPLIQVDAHNIVPCWEASDKQEYSARTIRNKINSKLSEYLTDFP

PLIKHPFTSKFEPEPIDWDEAIESREADKSVGPVNWARPGYDEAMKMLKSFIEKRLKIFSSKRNDPTQDACSNLSPWFHF

GQISVQRVALCVQEYKTKHTECVNAFLEEAIVRRELADNFCFYCEHYDSIKGASNWAQKTLDDHRKDKRTHIYSLEQLSK

AETHDELWNSAQLQLVKEGKMHGFLRMYWCKKILEWTSTPEDALRYSIYLNDHYSIDGRDPNGYVGCMWSTCGIHDQGWA

ERTVFGKIRYMNYDGCKRKFNVAAFVARYGGKVHKYTSKK

>Diatraea_sac_unname_CAH0747382.1 CAH0747382.1

MSAAADALPASSAGASGPAVPAPTKCSPAAKHIVHWFRKGLRLHDNPALRAGLNGAATFRCVFIIDPWFASSSNVGINKW

RFLLQCLEDLDCSLRKLNSRLFVVRGQPADALPKLFREWGITELTFEEDPEPYGRVRDHNIMSKCREFGINVVSRVSHTL

YKLDKIIERNGGKAPLTYHQFQALIASMPPPPPAEATLSAQTLNGATTPITDDHDDRFGVPTLEELGFETEGLKPPVWVG

GESEALVRLERHLERKAWVASFGRPKMTPQSLLASQTGLSPYLRFGCLSTRLFYYQLTELYKRIKRVQPPLSLHGQILWR

EFFYCAATRNPNFDRMEGNPICVQIPWERNQEALAKWANGQTGFPWIDAIMIQLRQEGWIHHLARHAVACFLTRGDLWIS

WEEGMKIFDELLLDADWSVNAGMWMWLSCSSFFQQFFHCYCPVRFGRKTDPNGDFIRRYIPALKNMPTRYIHEPWVAPES

VQQSARCIIGRHYPLPMVDHNKASQINIERIKQVYAQLAKYKPQAILNPQMLQRPNVIQSSPSPTTIIANINQSNYLCSQ

TSEAQSTATVQSSTFKNSNVFLHPGNNNSRHDLKQPQFKQVVIVQKQNNTNLQKVCPPPQSDYVINGQVNSPYKNNEDIE

RVQKSSKSENYNFKNLDINQYIQDYSSNKETYQSQQQRTEEIYQQDKANNFEFVKPKFYLPYPNNGIVHNNHAHETPKDG

HFTHDHNYDKETSNEAKKDKQMLNNCIQ

>Dinoponera_qua_PREDIC_XP_014472379.1 XP_014472379.1

MTGSSNNKMGQGVTSIRGDGRKHTVHWFRKGLRLHDNPSLREGLAGASTFRCVFVLDPWFAGSTNVGINKWRFLLQCLED

LDCSLRKLNSRLFVIRGQPADALPKLFKEWGTTNLTFEEDPEPFGRVRDHNISALCKELGISVVQRVSHTLYKLDEIIEK

NGGKPPLTYHQFQNVVASMKPPEPPVPTVTSACIGSAYTPLKDDHDDHYGVPTLEELGFDTEGLLPPVWVGSESEALARL

ERHLERKAWVASFGRPKMTPQSLLPSQTGLSPYLRFGCLSTRLFYYQLTDLYKKIKKAMPPLSLHGQLLWREFFYCAATK

NPNFDRMQGNPICVQIPWDKNVEALAKWANGQTGFPWIDAIMTQLREEGWIHHLARHAVACFLTRGDLWISWEEGMKVFD

ELLLDADWSVNAGMWMWLSCSSFFQQFFHCYCPVRFGRKADPNGDYIRRYLPVLKNFPTRYIHEPWNAPLSIQHAAKCII

GKEYSLPIVNHSKSSRINIERMKQVYQQLNKYRGNGASFKGETVGLLNALLAPPTNDTDEKKTKQDSPDQENEQKMETIS

SPTQQQQQQQQQQQQK

>Diprion_sim_CRY-1-_XP_046747043.1 XP_046747043.1

MTGSEKAQHGSAKPSRGDGKKHIVHWFRKGLRLHDNPALREGLTGATTFRCVFVLDPWFAGSTNVGINKWRFLLQCLEDL

DRSLWKLNSRLFVIRGQPADALPKLFKEWGTTDLTFEEDPEPFGRVRDQNITTLCEEMGISVVQRVSHTLYKLDSIIEKN

GGKAPLTYHQFQNVVAAMDPPSPPEASVTPEFTTGAYTPVRVDHNDVYGVPTLEELGFETENLRPPVWIGGESEALVRLE

RHLARKYWVASFGRPKMTPQSLLASQTGLSPYLRFGCLSTRLFFYQLTDLYKKIKKAVPPLSLHGQLLWREFFYCAATRN

PNFDRMQGNPICVQIPWDKNAEALAKWANGQTGFPWIDAIMTQLREEGWIHHLTRHAVACFLTRGDLWLSWEEGMKVFDE

LLLDADWSVNAGMWMWLSCSSFFQQFFHCYCPVRFGRKADPNGDYIRRYVPALKNFPAKYIHEPWNAPQSIQQAAKCIIG

KDYSLPMVDHGKSSRINIERMTQVYQQLNKYRGNGSSGTRGDKIEGLLNGAPAAATLLQCEDDEKNKHQTTSNPSLSPAR

HRKITGSTLSMVTLRQPDQTAEFPSAGND

>Diprion_sim_PL_XP_046742385.1 XP_046742385.1

MDKFVAKRAKLSNFVNKLEEDRKNHSESVMTFKFNKKRIRVLTATDKVKDNCKGIVYWMFRDSRVQDNWAFLFAQKIAMK

NRLPLHICFCVLPKFLGATIRHYKFHLAALEQVELECKNLNVNFHLLHGEPNDVILKFVGKYDMGAVITDFFPLRLPLSW

VDDLKKKLPENIPLCQVDAHNIVPCWEASDKLEYSARTIRNKINSKLDAYLTEFPPVIKHPYTTKQKFTQNDWENALKDV

EIDKSVKEITWAKPGYEEGILELERFIEKRLKLYDSKRNDPTVNALSNLSPWFHFGMISVQRCIIEVSKYKNSHKKSVEG

FMEEAIVRRELSDNFCFYNEHYDSLKGAKQWAMDTLDNHRKDKREYIYTLKEFENSLTHDDLWNSAQNQLIQDGKIHGFL

RMYWAKKILEWTRTPEEALEWSIYLNDKYSMDGRDPSGYVGCMWSICGIHDQGWAERSIFGKIRFMNYKGCQRKFDVKAF

VARWGGKVHTKKK

>Diuraphis_nox_PREDIC_XP_015363961.1 XP_015363961.1

MDKNNSDAGYETTVHWFRKGMRLHDNPAFRLSYEAKNGSGEHYKLRPIYILDPYFRKYIRAGANRWRFLQQSLVDLDTTL

RQLGTRLYVIRGLPHEVFPDLFVKWNVKLLTFELDTEPYARERDNQVEQLARKHSVKVEQKVSHTIYNTELVLRANGGSV

PMTYQKFVSVVGSMPTPRRPIPAPDMLPSECLLDDDLNNPEFDVPTLDELLTLKGFNPTELKPCLYPGGEKEALRRLEEY

MKNKTWVCKFEKPNTSPNSLKPSTTVLSPYMKFGCLSASHFYYRLKEVIGNSPHSKPPVSLIGQLYWREFYYTVGASTPN

FDKMVGNSICCQVPWDNNPDALEAWTNGKTGYPFIDAIMRQLRDEGWIHHLARHAVACFLTRGDLWISWEKGLAVFEELL

LDADWSLNAGNWMWLSASAFFHQFFRVYSPVAFGKKTDKSGDYIRKYIPELAKYPDQYIYEPWSAPKSIQERAGCVVGVH

YPKRVVVHEDVYKNNITKMSLAYKSSKASKSSNTKKSRDMSSSPDKKNIKKPKLK

>Diuraphis_nox_PREDIC_XP_015367949.1 XP_015367949.1

MSKMSDNSPPTKKIKKTNSEVSTSKTSNFLNDVAAERNKTAPSIMEFKFNKKRLRVLSEKKEVPEWAEGVIYWTFRDERI

HDNWALLYAQKLAIKNKVSLHITFCRLKQFLNCSLRHYKHIFQGLEELETECKSLDIQFHFLIGCAADILPXFVRKHKLG

AIVVDFMPVREHMTWTKQLAEXIGSEVPVIQVDSHNIVPCWVASDKQEYSARTIRNKINNKLPEFLTEFPPIIKHPFPST

FKAQPTNWDEADKTLEVDRSVVSVPGLKAGFKAGMSELEQFLKKRLPKYSTDRNNPVKDGLSKLSPWLHFGQISAQRCIL

EVSKLSKQYPDSVAAYREEAIIRRELSDNFCYYNPKYDKIEGAPTWAQTTLDEHRKDKRMYVYTREELEGSRTHDDLWNS

AQIQLVKEGKMHGFLRMYWAKKILEWTDTPDRALADAIYLNDKYSMDGRDPSGFVGCMWSICGIHDQGWREREIFGKIRY

MNYEGCKRKFDINAFIARYGGMVHKYTKK

>Diuraphis_nox_PREDIC_XP_015369276.1 XP_015369276.1

MTVAVHWFRNGLRLHDNPALIEAHNNAEKLLTLYIFDETTFNPTWYGYNPMRFLLESLIDLNNNLTLVGGRLYILRGNPV

NIFKIIKEKIGLNFITFEQDCDHIGRSRDDKVKAFCDENGVKYIEKVSHTLWNPKTIIEKNGGVPPFTFKQFQNIANQIG

HPPIPVGNLDWLSVIFEELPASMLDEFKILNNPTPETFGIYPEIPENLTTPYRWYGGETRALEQLKERLEYEREAFVNGF

YLPNQVNPDLLSPPSSLSAALRYGCLSIRKFHWELSKLFIKQFEGDLLPQYSVTSQLIWRDYFYTMSIDNKNFGQIEDNP

ACISIPWNDVKIPENKKMLECWKSGKTGYPFIDAGMRQLMQEGWVHHVVRNSLASFLTRGDLWISWVEGLNHFMKYLLDA

DFSVCAGNWIWVSSSTFEQLLDCPLCVCPVSYGLRLDPSGEYIRRYVPELKNMPAQYLYEPWKCPESVQKQVGCIIGKDY

PNRIVDHTIASRGNRKKMLALRVSMSSENVVPHCCPSDREEVQKFMYLPDECIQQLLPLENSDGEAYEFYKCH

>Diuraphis_nox_PREDIC_XP_015373503.1 XP_015373503.1

MEFNVQHKHTVHWFRKGLRIHDNPSLREGLINASTFRCIFILDPWFAGVSNVGINKWRFLLQCLSDLDNSLKKLNSRLFV

IKGQPAEALPKLFRQWGTTNFTFEEDPEPFGRVRDQNIKVMCSEMGISVITRCSHTLYQLDKIINVNGGKAPLTYHLFQK

LLECIDPPERAVPSIDKEFLGNAFTPIKYDHDEIFGVPTLEELGFKEINNSARHIWVGGETEALIRLQCHLERKAFIASY

GKPKMTPQSLVASPTGLAPYLKFGCLSTRLFFSELNELYKKIRKSQPPLSLHGQLLWRDFFYCASTNNPNFDRMVGNPIC

VQIPWDKNPQALSKWANGQTGYPWIDAIMIQLRQEGWIHCIARHAVACFLTRGDLWLSWEEGMKVFDELLLDADWSVNAG

YWMWYSCSSFYQEFIHCYCPVRFGRKVDPNGDYVRRYIPVLNNMPSQYIHEPWLAPESIQFTANCIIGIDYPLPIVNHVN

ASKINLERMKLAYQQLSNCQPQLENGKLILISSARR

>Drosophila_alb_CRY-2_XP_034098531.1 XP_034098531.1

MSIEIESKSTQRRTLVHWFRKGLRVHDNPALLQVFDVARTAPHKYSVRPIFLLDPGILDWLQVGANRWRFLQQSLSDLDN

QLRELNSRLYVVRGKPVELFPKLFERWNVELLTYESDIEPYAVQRDAAVQKLAAVHQVHVDTHCSHTIYNPEVVMARNLG

KAPITYQKFLGIVDQLKLPKVREKPEKLPEEVQPSEDALELADASVYDCPTLEQLVKRPDELGDNKFPGGETEALRRLEA

SLRDEQWVASFEKPKTSPNSLEPSTTVLSPYLKFGCLSARLLYERLKGILARHPKHSKPPVSLVGQLLWREFYYTAAAAE

PNFDRMLGNAYCIQIAWNEQPDHLAAWTHGRTGYPFIDAIMRQLRQEGWIHHLARHAVACFLTRGDLWISWEEGQRVFEQ

LLLDQDWALNAGNWMWLSASAFFYQYFRVYSPVAFGKKTDPTGAYIRKYVPELAKYPANCIFEPWKATLSAQREYGCVLG

KDYPHRIVNHDIVHKENIKRMTAAYKVNREVRTGKQEPDEELDEPTGKRKASSIKSGRAAKRRR

>Drosophila_alb_LOW_QU_XP_034115265.1 XP_034115265.1

MALRSANVMWFRHGLRXHDNPALLSALADKDHGIALIPIFIFDGESAGTKSVGFNRMRFLLDSLQDIDQQLQSETEGRGR

LFIFEXKSSGDLRRLNEQVRLHKICVEQDCEPIWNERDEATKTLCNELGIEYVEKVSHTLWDPRTVIDTNGGIAPLTYQM

FLHTVQIIGLPPRPVHDPHFGGVSFVQLSPELRRDLGCFEQPPTPEHFNIYSDNMGYLSKINWIGGESQALELLXERLKI

EQHAFERGFYLPNQAMPNILDTPRSMSAHLRFGCLSVXRFYWSVHDLFKNVQLRACVRGVQMTGGAHITGQLIWARIFLY

TMSVNNPNYDRMEGNEICLNIPWAKPNDEQLQRWRLGQTGFPLIDGAMRQLLAEGWLHHTLRNTVATFLTRGGLWQNWEY

GVQHFLKYLXDADWSVCAGNWMWVSSSAFERLLDSSLVTCPVALAKRLDPEGIYIKQYVPELQNVPQEFIHEPWRMSAEQ

QERYECLIGVHYPERILDLAVAGKRNTLAMNSLRNSLITPQPHCRPSNEEEVRQFFWLVD

>Drosophila_alb_PL_XP_034108756.1 XP_034108756.1

MFFGGSRLFRTLHMKRTKATTSRAVKKKPKKEESSSSGEETELERTSSPERESTSPGYKNVDQFIGYMQDQRLASAADVH

DFAFRKNRVRLLSAENDVKESCKGGVVFWMSRDARVQDNWAFLYAQRTALKLELPLSVVFCLVPKFLNATLRHYKFMLGG

LQEVEQECRKLNIAFHLLLGPAVERLPEFVAAQDMGAVVCDFAPLRLPRQWVTDVTKALPKNVPLLQVDAHNVVPVWVTS

EKQEYAARTIRNKINSKLPEFLTEFPPLIKHPHGKASKVKDVNWAAAQQQLTCDMSVDEVEWAKPGYKAACRQLYEFCTH

RLRDFNEKRNNPLADAVSGLSPWLHFGQISAQRCILEVKRYAGKYKASVEGYCEETIVRRELSDNFCYYNENYDSLKGLS

DWAYQSLEAHRKDKRSPCYTLDELEQSRTYDDLWNSAQLQLVNEGKMHGFLRMYWAKKILEWTETPEQALEYSILLNDKY

SLDGRDPNGYVGCMWSVGGIHDNAWKERAIFGKIRYMNYQGCKRKFDVNAFVMRYGGKAYKKDDN

>Drosophila_ana_CRY-1_XP_001954843.1 XP_001954843.1

MATRGANVIWFRHGLRLHDNPALLAALAEKDQGIALLPVFIFDGESAGTKNVGYNRMRFLLDSLQDIDDQIQTLTDGRGR

LLLFEGKPEHIFRRLHEQLRLHKICLEQDCEPIWNHRDETIRSLCHELGIEFVEKVSHTLWNPQSVIETNGGIPPLTYQM

FLHTVQILGLPPRPVNDARLEDASFVQMDPELLRNLGYLEQIPTPEHFNVYGDNMGFLSKIRWRGGERQALLLLDERLKV

EQHAFEKGYYMPNQALPNIQETPKSMSPHLRFGCLSVRRFYWSVHDLFKNVQLRACVRGVQMTGGAHITGQLIWREYFYT

MSVNNPNYDRMDGNEICLSIPWAKRDETQLQKWRLGQTGFPLIDAAMRQLLAEGWLHHVLRNTVATFLTRGGLWQSWEHG

VQHFLRYLLDADWSVCAGNWMWVSSSAFERLLDSSLVTCPVALAKRLDPDGAYIRQYVPELKNVPREFIHEPWRMNLQQQ

EQYECLIGVHYPDRLIDLSKAVKGNMLAMKTLRDSLITPPPHCRPSNEEEVRTFFWLADLTI

>Drosophila_ana_CRY-2_XP_001962202.1 XP_001962202.1

MDAKHATLVHWFRKGLRVHDNPALSQIFKVANTAPGKYFVRPIFILDPGILDWMQVGANRWRFLQQTLHDLDQQLQKLGS

RLFVVRGKPAEVFPRIFKSWRVELLTFETDIEPYSLARDSAVQKLAKSDGVKVETHCSHTIYNPELVIAKNLGKAPITYQ

KFLGIVEKLKLPTVLDLPEKLKEKVQPPKDDIEEKDSEAYDCPTMEQLVKRPEDLGPLKFPGGETEALRRMEESLKDELW

VARFEKPNTAPNSLEPSTTVLSPYLKFGCLSSRLFHQRLKEILKRQTKHSQPPVSLIGQMMWREFYYTVAAAEPNFDRML

GNVYCLQIPWEEHPDHLKAWTYGQTGYPFIDAIMRQLRQEGWIHHLARHAVACFLTRGDLWISWEEGQRVFEQLLLDQDW

ALNAGNWMWLSASAFFHQYFRVYSPVAFGKKTDPQGNYIRKYVPELAKYPAGCIYEPWKASLSDQRAYGCVLGTDYPHRI

VKHEVVHKENIKRMGAAYKVNREVRTGKQEDSFEEKPETSTSGKRKLGKTSGNSAKRKR

>Drosophila_ana_PL_X1_XP_044571636.1 XP_044571636.1

MNGMFKTSKRVAIPSLRWLHQQIIMKRTKTAKAGPAKKTAKKEKPKSEESDQESSGSSQLEASSSKSVLSKPEYKNLEQF

LTHCQDQRTSTAANIKEFNFLKKRVRVLSKNGDVGDSCEGGVIYWMSRDGRVQDNWALLFAQRLALKLELPLAVVFCLVP

KFLNATIRHYKFMMGGLQEVEQQCRELDIPFHLLLGPAVDRIPEFVKSRKVGAVICDFAPLRVPRKWVDDVVKALPKTVP

LVQVDAHNVVPVWVASDKQEYAARTIRNKINSKLSEYLTEFPPVVKHPHGTGCRKVDPVDWTAAYEMLECDKSVDEVDWA

KPGYKAACKQLYEFCSRRLRQFNDKRNDPLADAISGLSPWLHFGQISAQRCALEVQRFRGQHKASAEAFCEEAIVRRELA

DNFCYYNEHYDSLKGLSSWAYQTLDAHRKDKRDPCYSLEEMEKALTYDDLWNSAQLQLVREGKMHGFLRMYWAKKILEWT

ETPEQALEYAILLNDKYSLDGRDPNGYVGCMWSIGGVHDMGWKERSIFGKIRYMNYQGCRRKFDVNAFVIPLTIQHIDFS

FHTTKMDAKISCDRPPEAPERILLSFLRSFLRS

>Drosophila_ari_PREDIC_XP_017856393.1 XP_017856393.1

MARCGANVIWFRHGLRLHDNPAMLAALSNKDQDVALIPIFIFDGESAGTKNVGYNRMRFLLDSLQDIDRQLQEATEGRGG

LLICEGQPVRIFRRLHEHVGLHKICVEQDCEPIWNERDEAVKSLCRELSIEYVEKVSHTLWDPRTVIDTNGGIAPLTYQM

FLHTVQIIGLPPRPVRDPNFEGVKFVQLSPELRHDIGCFEMTPTPEHFNVYSDNMGYLAKINWVGGETQALLLLGERLKV

EQHAFERGYYLPNQAMPNILDTPKSMSAHLRFGCLSVRRFYWCMHDLFKNVQLRACVRGVQMSGGAHITGQLIWREYFYT

MSVNNPQYDRMEGNEICLSIPWAKPDAEQLQRWRLGQTGFPLIDSAMRQLLAEGWLHHTLRNTVATFLTRGGLWQNWEFG

LQHFLKYLLDADWSVCAGNWMWVSSSAFERLLDSSLVTCPVALAKRLDPNGQYIKQYVPELQHVPKEYIHEPWLMSAEEQ

KRYECLIGVHYPDRIIDLSLASKRNMLAMKALRNSLIEPPPHCRPSNEEEVRQFFWLAD

>Drosophila_ari_PREDIC_XP_017859131.1 XP_017859131.1

MAAERRTLIHWFRKGLRVHDNPALTQIFNKARASPDKFSVRPIFVLDPGILDWMQVGANRWRFLQQSLLDLDKNLKELNS

RLYIVRGKPVDIFPNLFDRWNVELLTFETDIEPYAMKRDKAVQDIAAAHGVKVDTHCSHTIYNPEIVVAKNFGRAPITYQ

KFLSVVEKLKLPKVLDKPQRLPNGVQPIADELEVGQSDVYECPTLDQLVKRPQELGINKFPGGEREALRRLDTSLSDEHW

VASFEKPNTAPNSLEPSTTVLSPYLKFGCLSARLVHQRLQEILKRHPKHSKPPVSLVGQLLWREFYYTAASVEPNFDRML

GNVYCLQIPWQERPDHLDAWAHGRTGYPFIDAIMRQLRQEGWIHHLARHAVACFLTRGDLWISWEEGQRVFEQLLLDQDW

ALNAGNWMWLSASAFFHQYFRVYSPVAFGKKTDRTGAYIRKYVPELAKYPAGCIYEPWKATLADQREFGCVLGIDYPHRI

VNHDIVHKENIKRMSAAYKINREVRTGKQEDEIDYDSSMSLTTNKRNASFETKRSTAKRKR

>Drosophila_ari_PREDIC_XP_017865817.1 XP_017865817.1

MHIGGSVSTKFALLGGCRVLFQLLHMKRNKSNAVRAAKKKPKKAESNGSSNESESGSGSGEETVTITPEKKTKPSYNSFE

QFLSHVQDQRLKAAANVHEFAFRKKRVRVLSSASDVRENCNGGVVYWMSRDARVQDNWALLFAQRLALKLELPLTVVFCL

VPKFLNATLRHYKFMLGGLKEVEQQCRELNVSFHLLIGPAVERLPQFVKDEQIGAVICDFAPLRLPRQWVEDVVKALPAH

VPLTQVDAHNVVPLWVTSDKQEYAARTIRNKINSKLSEYLTEFPPLIKHAYGNVKGSRSVDWTAAYQTLSCDKSVDAVEW

AKPGYTAACRQLYDFCTRRLRHFNDKRNDPMADALSGLSPWLHFGQISAQRCVLEVGRYKTIYKESVEAYFEEAIVRREL

ADNFCYYNEHYDSLKGLYSWAYESLQAHRKDKRSPCYNLEELEHSRTYDDLWNSAQLQLVKEGKMHGFLRMYWAKKILEW

TETPEQALEYSILLNDKYSLDGRDPNGYVGCMWSIGGIHDQGWKERAIFGKIRYMNYQGCKRKFDVNAYVMRYGGKVHKK

KES

>Drosophila_bia_CRY-1_XP_043946773.1 XP_043946773.1

MATRGANVIWFRHGLRLHDNPALLAALADKDQGIALIPVFIFDGESAGTKSVGYNRMRFLLDSLQDIDDQLQVATEGRGR

LHVFEGEPAHIFRRLHEQVRLHRICIEQDCEPIWNERDETIRSLCRELSIDLVEKVSHTLWDPRTVIDTNGGIPPLTYQM

FLHTVQIIGLPPRPAADPRLDDVSFVELAPELRRSLGYFEELPTPEHFNVYGDNMGFLAKINWRGGETQALLLLEERLKV

EQHAFERGFYLPNQALPNIHDTPKSMSAHLRFGCLSVRRFYWSVHDLFKNVQLRACVRGVQMTGGAHITGQLIWREYFYT

MSVNNPNYDRMEGNEICLTIPWAKPNEDLLQRWRLGQTGFPLIDGAMRQLLAEGWLHHTLRNTVATFLTRGGMWQSWEHG

LQHFLKYLLDADWSVCAGNWMWVSSSAFERLLDSSLVTCPVALAKRLDPEGAYIKQYVPELMNVPKEFVHEPWRMSPEQQ

EQYECLIGVHYPERIIDLSVYVKRNMMAMKALRNSLITPPPHCRPSNEEEVRQFFWLADVAV

>Drosophila_bia_CRY-2_XP_016964619.1 XP_016964619.1

MDAQRSTLVHWFRKGLRVHDNPALSQIFTTANAKPGKYHIRPIFILDPGILDWMQVGANRWRFLQQTLEDLDKQLRKLDS

RLYVVRGKPADVFPKIFKSWRVELLTFETDIEPYSLSRDAAVQKLAKSEGVRVETHCSHTIYNPELVIAKNFGKAPITYQ

KFLGIVDKLKVPNVLGDPEKLSKKVEPPKDEVEQEDSAAYDCPTMEQLVKRPEELGPNKFPGGETEALRRMEKSLKDELW

VARFEKPNTAPNSLEPSTTVLSPYLKFGCLSARLFHQKLKEILKRQTKHSHPPVSLVGQLLWREFYYTVATAEPNFDRML

GNVYCMQIPWQEQPDHLKAWAHGRTGYPFIDAIMRQLRQEGWIHHLARHAVACFLTRGDLWISWEEGQRVFEQLLLDQDW

ALNAGNWMWLSASAFFHQYFRVYSPVAFGKKTDPQGHYIRKYVPELAKYPAGCIYEPWKASLADQRAYGCVLGTDYPHRI

VKHEVVHKENIKRMGAAYKVNREVRTGKEEESSFEEKPETSTSGKRKRKAAGNAPKRKR

>Drosophila_bia_PL-lik_XP_016968387.1 XP_016968387.1

MFTLATFWRESFKIAHPLHIMKRTKVTKAGPSKKSAKKEEDSSKKMNKSTQESSDEASPSKASLASKPDYQNFEQFLTHL

DHQRVCTAASIQEFTFRKKRARVLSKTEDVEESSQGGVVYWMSRDCRVQDNWALLFAQRLALKLELPLSVVFCLVPKFLN

ATIRHYKFMIEGLQEVEQQCRSLEIPFHLLMGSAAEKLPEFVKSQDVGAVVCDFCPLRVPRQWVEDVGKALPKCVPLVQV

DAHNVVPLWVASDKQEYAARTIRNKINSKLGEFLTEFPPVIRHPHGTGCKKAKPVDWSAAYDMLQCDMDVDEVQWAKPGY

KAACQQLYDFCTRRLRKFNDKRNDPMADALSGLSPWLHFGQISAQRCALEVQRFRGQHKASADAFCEEAIVRRELADNFC

FYNEHYDSLKGLSSWAYQTLDAHRKDKRDPCYGLEELEKSLTYDDLWNSAQLQLVREGKMHGFLRMYWAKKILEWTATPE

EALEYAILLNDKYSLDGRDPNGYVGCMWSIGGVHDMGWKERAIFGKVRYMNYQGCKRKFDVNAFVMRYGGKVHKKK

>Drosophila_bip_CRY-1_XP_017103840.2 XP_017103840.2

MATRGANVIWFRHGLRLHDNPALLAALAGKDQGIALLPIFIFDGESAGTKNVGFNRMRFLLDSLQDIDDQIQTLTEGRGR

LLVFEGEPKHIFRRLHERVHLHKICFEQDCEPIWNHRDETIRSLCEELGIQCVEKVSHTLWDPRIVIETNGGIPPLTYQM

FLHTVQIIGLPPRPTSNARLDNATFVQLEPEILRSLGFLEQIPTPEHFNVYGDNMGFLSKIRWRGGEREALLLLEERLKV

EQHAFEKGFYLPNQAMPNIQETPKSMSPHLRFGCLSVRRFYWSVHDLFQNVQLRACVRGVQMTGGAHITGQLIWREYFYT

MSVNNPNYDRMEGNEICLSIPWAKPNETQLQKWRLGQTGFPLIDAAMRQLLAEGWLHHVLRNTVATFLTRGGLWQSWEHG

VQHFLRYLLDADWSVCAGNWMWVSSSAFERLLDSSLVTCPVALAKRLDPEGAYIRQYVPELKNVPRELIHEPWRMSLQQQ

EQYECLIGVHYPDRLIDLTKAVKGNMLAMKSLRDSLITPPPHCRPSNEEEVRTFFWLADLTI

>Drosophila_bip_CRY-2_XP_017097267.2 XP_017097267.2

MDAKHATLVHWFRKGLRVHDNPALLQIFKVANGTPGKYFVRPIFILDPGILDWMQVGANRWRFLQQTLHDLDQQLQKLGS

RLFVVRGKPAEVFPRIFKSWRVELLTFESDIEPYSLARDSAVQKLAKSEGVKVETHCSHTIFNPELVIARNLGKAPITYQ

KFLGIVEKLKLPTVLGLPEKLKGEGQPPKDDIEEKDSEAYDCPTMEQLVKRPEELGPNKFPGGETEALRRMEESLKDELW

VARFEKPNTAPNSLEPSTTVLSPYLKFGCLSSRLFHQRLKEILKRQTKHSQPPVSLIGQMMWREFYYTVAAAEPNFDRML

GNVYCLQIPWEEHPDHLKAWTYGKTGYPFIDAIMRQLRQEGWIHHLARHAVACFLTRGDLWISWEEGQRVFEQLLLDQDW

ALNAGNWMWLSASAFFHQYFRVYSPVAFGKKTDPQGNYIRKYVPELAKYPAGSIYEPWKVSLADQRAYGCVLGTDYPHRI

VKHEVVHKENIKRMGAAYKVNREVRTGKQEDSFEEPEPSSSGKRKLGKTTGNSAKRKR

>Drosophila_bip_hypoth_KAH8233008.1 KAH8233008.1

RDSETDFYFVPYVQLLKQSIMKRTKTSKAGPPKKAAKKETPKSEESDQESSGSSQVEASSSKTLLLKPEYKNLEQFLTHC

EHQRTSTAASIKEFNFLKKRVRVLSKNGDVGDSCEGGVVYWMSRDGRVQDNWAMLFAQRLALKLELPLAVVFCLVPKFLN

ATIRHYKFMMGGLQEVEQQCRELDIPFHLLLGPAVDRLPEFVKSRKVGAVICDFAPLRVPRKWVEDVGKALPKTVPLVQV

DAHNVVPVWVASDKQEYAARTIRNKINSKLSEYLTEFPPVIKHPHGTGCRKVDPVDWTAAYEMLECDKTVDEVDWAKPGY

KAACKQLYEFCSRRLRIFNDKRNDPIADAISGLSPWLHFGQISAQRCALEVQRFRGQHKASAEAFCEEAIVRRELADNFC

YYNEHYDSLKGLSSWAYETLNAHRKDKRDPCYSLDEMEKALTYDDLWNSAQLQLVREGKMHGFLRMYWAKKILEWSETPE

QALEYAILLNDKYSLDGRDPNGYVGCMWSIGGIHDMGWKERSIFGKIRYMNYQGCRRKFDVNAFVIRYGGKVHKKK

>Drosophila_bir_hypoth_KAH8249538.1 KAH8249538.1

MDGKKTTLVHWFRKGLRVHDNPALSHIFSAANYSPEKFCVRPIFILDPGILDWMQVGANRWRFLQQTLQDLDEQLRKLNS

RLFVVRGKPAEVFPRIFKSWRVELLTFESDIEPYSLTRDAAIQKLAKAEGVKVVSHCSHTIYNPELVIAKNLGKAPITYQ

KFLSIVDQLKVPKVLEVPEKLKAVVAQPPKDDVELGDAAAYDCPTMEQLVKRPEDLGPNKFPGGETEALRRMDESLKDEL

WVARFEKPNTAPNSLEPSTTVLSPYLKFGCLSARLFNQRLKDILKRQTKHSQPPVSLIGQLMWREFYYTVAAAEPNFDRM

LGNVYCLQIPWQEQPDHLEAWTHGRTGYPFIDAIMRQLRQEGWIHHLARHAVACFLTRGDLWISWEEGQRVFEQLLLDQD

WALNAGNWMWLSASAFFHQYFRVYSPVAFGKKTDPQGDYIRKYVPELAKYPKNCIYEPWKATLADQRAYGCVLGTDYPHR

IVKHEVVHKENIKRMSAAYKVNREVRTGKEEEEDSFEEKPEASTSGKRKVRKTAGSAAKRRR

>Drosophila_bir_hypoth_KAH8253055.1 KAH8253055.1

ITPLLTMKRAKAAKAGPSKKVAKKQEQSPKKEASGQESSDDEPSTSKAVPAKPNYQNLQQFIAHLEHQRSVTAANIQEFQ

FRKKRVRVLSKSADVKESCKGGVVYWMSRDARVQDNWALLFAQRLALKLELPLTVVFCLVPKFLNATIRHYKFLMGGLEE

VEQQCRSLDIPFHLLLGPAVQRLPEFVRSQDLGAVVCDFAPLRLPRQWVEDVGKALPKSVPLVQVDAHNVVPLWVASDKQ

EYAARTIRNKINSKLGEFLTEFPPVVRHPHGRGCQGVKPVDWPAAYATLECDTDVGEVDWAKPGYKAACQQLYEFCSRRL

RHFNDKRNDPMADALSGLSPWLHFGQISAQRCALEVQRFRGQHKASADAFCEEAIVRRELADNFCYYNENYDSLKGLTAW

AYQTLDAHRKDKRDPCYSLEELEESLTYDDLWNSAQLQLVREGKMHGFLRMYWAKKILEWSPTPEQALEYAILLNDKYSL

DGRDPNGYVGCMWSIGGVHDMGWKERAIFGKIRYMNYQGCRRKFDVNAFVMRYGGKVYKRK

>Drosophila_bir_hypoth_KAH8256334.1 KAH8256334.1

MATRGANVIWFRHGLRLHDNPALVAALSDKDQGIALIPVFIFDGESAGTKCVGYNRMRFLLDSLQDIDDQIQAATEGRGR

LHVFEGEPVNIFRRLNEQVRLHRICLEQDCEPIWIDRDDSVRSMCRELNIDFVEKVSHTLWDPRTVIDTNGGIPPLTYQM

FLHTVQIIGLPPRPAADVRLEDATFVELAPELRRSLGYFEKMPSPDHFNVYSDNMGFLAKINWRGGETQALLLLEERLKV

EQHAFERGLYLPNQALPNIHDTPKSMSAHLRFGCLSVRRFYWSVHDLFKNVQLRACVRGVQMTGGAHITGQLIWREYFYT

MSVNNPNYDRMDGNEICLSIPWAKPDEDLLQRWRLGQTGFPLIDAAMRQLLAEGWLHHTLRNTVATFLTRGGLWQSWEHG

LQHFLKYLLDADWSVCAGNWMWVSSSAFERLLDSSLVTCPVALAKRLDPDGAYIKQYVPELMGVPKEFVHEPWRMSAEQQ

EQYECLIGVHYPERIIDLSKAVKRNMLAMTALRNSLITPPPHCRPSNEEEVRQFFWLADQTV

>Drosophila_bun_hypoth_KAH8248212.1 KAH8248212.1

MATRGANVMWFRHGLRLHDNPALVAALSDKDQGIALIPVFIFDGESAGTKCVGYNRMRFLLDSLQDIDDQIQAATEGRGR

LHVFEGEPVNIFRRLNEQVRLHRICIEQDCEPIWNDRDDSVRSLCHELGIDFVEKVSHTLWDPRMVIDTNGGIPPLTYQM

FLHTVQIIGLPPRPAADVHLEDASFIELAPELRRSLGYFEKMPNPDHFNVYGDNMGFLAKINWRGGETQALLLLEERLKV

EQHAFERGFYLPNQALPNIHDTPKSMSAHLRFGCLSVRRFYWSVHDLFKNVQLRACVRGVQMSGGAHITGQLIWREYFYT

MSVNNPNYDRMDGNEICLSIPWAKPDEDLLQRWRLGQTGFPLIDAAMRQLLAEGWLHHTLRNTVATFLTRGGLWQSWEHG

LQHFLKYLLDADWSVCAGNWMWVSSSAFERLLDSSLVTCPVALAKRLDPEGAYIKQYVPELMGVPKEFVHEPWRMSAEQQ

EQYECLIGVHYPERIIDLSKAVKRNMLAMTALRNSLITPPPHCRPSNEEEVRQFFWLADQTV

>Drosophila_bun_hypoth_KAH8255322.1 KAH8255322.1

MDGRKSTLVHWFRKGLRVHDNPALSQIFYAANASPEKFSVRPIFILDPGILDWMQVGANRWRFLQQTLQDLDEQLRKLNS

RLFVVRGKPAEVFSRIFKSWSVELLTFESDIEPYSLARDASIQKLAKTEGVKVVTHCSHTIYNPELVLAKNLGKAPITYQ

KFLSIVDQLKISKVLEVPEKLKVVVKPPKDDLELGDLAAYDCPTMEQLVKRPEELGPNKFPGGETEALRRMDESLKDEVW

VARFEKPNTAPNSLEPSTTVLSPYLKFGCLSARLFYQRLKDILKRHTKHSHPPVSLIGQLMWREFYYTVAAAEPNFDRML

GNVYCLQIPWQEHPDHLEAWTHGRTGYPFIDAIMRQLRQEGWIHHLARHAVACFLTRGDLWISWEEGQRVFEQLLLDQDW

ALNAGNWMWLSASAFFHQYFRVYSPVAFGKKTDPQGNYIRKYVPELAKYPKGCIYEPWKATLADQRAYGCVLGTDYPHRI

VKHEVVHKENIKRMSAAYKVNREVRTGKEEEDSFEEKPVASTSGKRKVRKATGSAAKRKR

>Drosophila_bun_hypoth_KAH8258160.1 KAH8258160.1

LRTMKRAQAAKAGPSKKVTKKQEKSPRKKSKSDQDSSGGSGDEEASASKASLTKPDYQNLEQFIVHLEHQRSETAANIQE

FPFRKKRVRVLSQSADVKESCQGGVVYWMSRDGRVQDNWALLFAQRLALKLELPLTVVFCLVPKFLNATIRHYKFLMGGL

EEVEQQCRSLDIPFHLLLGPAVQRLPEFVKSQDIGAVVCDFAPLRLPRQWVEDVGKALPKSVPLVQVDAHNVVPLWVASD

KQEYAARTIRNKINSKLGEFLTEFPAVVKHPHGKGCQGVKAVDWPAAYATLQCDQDVDEVEWAKPGYKAACQQLYEFCSR

RLRHFNDKRNDPMADALSGLSPWLHFGQISAQRCALEVQRFRGQHKASADAFCEEAIVRRELADNFCYYNEHYDSLKGLT

AWAYQTLDTHRKDKRDPCYSLEELEKSLTYDDLWNSAQLQLVREGKMHGFLRMYWAKKILEWSPSPEQALEYAILLNDKY

SLDGRDPNGYVGCMWSIGGVHDMGWKERAIFGKIRYMNYQGCRRKFDVNAFVMRYGGKVHKRK

>Drosophila_bus_CRY-1_XP_017845052.1 XP_017845052.1

MAALAANVIWFRHGLRLHDNPALQAALLDQDQGVALIPIFIFDGESAGTKSVGYNRMRFLLDSLEDINEQLQKATENRGR

LWICQGQPVDIFRRINEQVRLIKICVEQDCEPIWNERDNAVRNLCRQLDVEYVEKVSHTLWDPRLVMDTNGGIAPLTYQM

FLHTVHIIGLPPRPVDAPNLQRVSFVQLSPELADKLGCLQQSLTPEQFNVYSDNMGYLAKTNWRGGETQALLLLGERLKV

EQHAFERGFYLPNQAMPNILEPPKSMSAHLRFGCLSVRRFYWSVHDLFKNVQLRACVRGVEMTGGAHITSQLIWREYFYT

MSVNNPQYDRMEGNEICLSIPWAKPHEEQLQRWRLGQTGFPLIDAAMRQLLAEGWLHHTLRNTVATFLTRGGLWQNWEHG

LQHFLKYLLDADWSVCAGNWMWVSSSAFERLLDSSLVTCPVALAKRLDPQGVYIRQYVPELKRVPREFIHEPWRMNAEQQ

ERFECLVGIHYPERIIDLEKACKRNMLAMRALRHSLIEPPPHCRPSNEEEVRQFFWLAD

>Drosophila_bus_CRY-2_XP_017855124.2 XP_017855124.2

MGTQRKSLVHWFRKGLRVHDNPALSETFKVARDSPDEFCVRPIFILDPGILDWMQVGANRWRFLQQSLADLDLQLRKLNS

RLYVVRGKPIEVFPQLFERWHVHLLTYETDIEPYAVTRDATVQKLAAAAGVKVDTHCSHTIYNPELIIARNMGKAPVTYQ

KFLSIVEKLKLPKALDVLPRLPDGMQPVKDANEVNDTKVYDCPTLEQLVKRPEELGDNKFPGGETEALRRMEASLDDEVW

VAKFEKPNTAPNSLEPSTTVLSPYLKFGCLSARLLHRRLQDILARHPKHSKPPVSLVGQLMWREFYYTAAAADPNFDRML

GNAYSLQIPWQENSDHLEAWAHGRTGYPFIDAIMRQLRQEGWIHHLARHAVACFLTRGDLWISWEEGQRVFEQLLLDQDW

ALNAGNWMWLSASAFFHQYFRVYSPVAFGKKTDSTGAYIRKYVPELAKYPAGCIYEPWKATLSAQREYGCVLGVDYPHRI

VDHDVVHKENIKRMSAAYKVNREVRTGKEEVDEYELKSQGNAKRKARAVATGRTAKRKR

>Drosophila_bus_phr__ALC42266.1 ALC42266.1

SMKRARSTKAGAKSRPVKKSESESSVDEPAPKATKNIKVKEEKAAINYSNFEEFLNHLQERRVDAAADVHEFAFKKQRVR

LLSAISDVKENCPGGVVYWMSRDARVQDNWALLFAQRLAIKLELPLTVVFCLVPKFLNATLRHYKFLLGGLQEVEQECKE

LNIGFELLLGSAVDRLPELVKKNDIGAVICDFAPLRLPRQWVEQVAEALPSQVPLTQVDAHNIVPLWVTSEKQEYAARTI

RNKINSKLCDYLTEFPPVISHPHKLAKPSKAVDWIAAEAMLTCDRSVDAVDWAKPGYTAACRQLYEFCTRRLRHFNDKRN

DPSANALSGLSPWLHFGHISAQRCILEVQRYSAQHKASADAFCEEAIVRRELADNFCYYNEHYDSLKGLYDWAYQTLQAH

RQDKRSPCYTLAQLEQSHTADDLWNSAQLQLVQEGKMHGFLRMYWAKKILEWTETPELALEYAILLNDKYSLDGRDPNGY

VGCMWSIGGIHDQGWKERAIFGKIRYMNYQGCKRKFDVNAFVMRYGGKVYKN

>Drosophila_ele_CRY-1_XP_017112179.1 XP_017112179.1

MEMATRGANVMWFRHGLRLHDNPALLAALADKDQGIALIPVFIFDGESAGTKCVGYNRMRFLLDSLQDIDEQLQAATEGR

GRLHVFEGEPAHIFRRLHEQVRLHRICMEQDCEPIWNDRDESVRSLCRELKIDFVEKVSHTLWDPRTVIDTNGGIPPLTY

QMFLHTVQIIGLPPRPAADAQLENASFVELAPEMRQSLGYFERLPTPEHFNVYGDNMGFLAKINWRGGETQALLLLEERL

KVEQHAFERGFYLPNQASPNILDKPKSMSAHLRFGCLSVRRFYWCVHDLFKNVQLRACVRGVQMTGGAHITGQLIWREYF

YTMSVNNPNYDRMDGNEICLSIPWAKPNEDLLQRWRLGQTGFPLIDGAMRQLLAEGWLHHTLRNTVATFLTRGGMWQSWE

HGLQHFLKYLLDADWSVCAGNWMWVSSSAFERLLDSSLVTCPVALAKRLDPEGAYIKHYVPELMNVPKEFVHEPWRMSAE

QQEQYECLIGVHYPERIIDLSMAVKRNMLAMKALRNSLMTPPPHCRPSNEEEVRQFFWLADVEV

>Drosophila_ele_CRY-2_XP_017120898.1 XP_017120898.1

MGAERSTLVHWFRKGLRVHDNPALSQIFAAANAAPGKFFVRPIFILDPGILDWIQVGANRWRFLQQTLSDLDQQLRKLDS

RLFVVRGKPAEVFPRIFKSWRVELLTFETDIEPYSLARDEAVQKLAKSAGVKVETHCSHTIYNPELVIAKNLGKAPITYQ

KFLGIVEQLKIPKVLGVPQKLKIINEPTNDEVEQEDSTAYDCPTMEQLVKRPEELGPNKFPGGETEALRRMDKSLSDELW

VARFEKPNTAPNSLEPSTTVLSPYLKFGCLSARLFHQRLKEILKRQTKHSQPPVSLVGQLMWREFYYTVAAAEPNFDRML

GNVYCLQIPWQKQPIHLKAWTHGRTGYPFIDAIMRQLRQEGWIHHLARHAVACFLTRGDLWISWEEGQRVFEQLLLDQDW

ALNAGNWMWLSASAFFHQYFRVYSPVAFGKKTDPQGHYIRKYVPELSKYPAGCIYEPWKASLADQRAYGCVLGTDYPHRI

VKHEIVHKENIKRMGAAYKVNREVRTGKEEASFEEKPETSTSGKRKARKAVENAPKRKR

>Drosophila_ele_PL_XP_017132851.1 XP_017132851.1

MPPHGAVMFTLAINWRECFKIILPLHTMKRAKTTKAGPSKKATKKQEGNRKEESKDSQESTEEEASPSKASSASKPDYQN

FDQFLTHLEVQRFSTADSIKEFSFRKKRVRVLSKNEDVEESSKGGVVYWMSRDGRVQDNWALLFAQRLALKLELPLSVVF

CLVPKFLNATIRHYKFMMGGLQEVEQQCRTLDIPFHLLIGPPVERLPDFVKTNDIGAVVCDFSPLRLPRQWVDDVGKALP

KSVPLVQVDAHNVVPLWVASDKQEYAARTIRNKINSKLDEFLSEFPPVVPHPHGSGCKGAKAVDWPAAYAKLQCDMDVDE

VQGAQPGYKAACQQLYEFCSRRLRHFNDKRNDPTADALSGLSPWLHFGQISAQRCALEVQRFRGQHKASADAFCEEAIVR

RELADNFCYYNEHYDSLKGLSSWAYQTLDAHRKDKRDPCYNLEELEKSLTYDDLWNSAQLQLVREGKMHGFLRMYWAKKI

LEWTATPELALEYAILLNDKYSLDGRDPNGYVGCMWSIGGVHDMGWKERAIFGKVRYMNYQGCRRKFDVNAFVMRYGGKV

HKNK

>Drosophila_ere_CRY-1_XP_001979555.1 XP_001979555.1

MAKRGANVIWFRHGLRLHDNPALLAALADKDQGVALIPVFIFDGESAGTKNVGYNRMRFLLDSLQDIDDQLQEATDGRGR

LLVFEGEPAFIFRRLHEQVRLHRLCIEQDCEPIWNHRDETIRALCRELGIDFVEKVSHTLWDPQLVIETNGGIPPLTYQM

FLHTVQIIGLPPRPTADARLEDATFVELDPEFRRSINMFEKLPTPHHFNVYEDNMGFLAKIKWRGGEKQALLLLGERLKV

EQHAFERGFYLPNQALPNIHDSPKSMSAHLRFGCLSVRRFYWSVHDLFKNVQLRACVRGVQMTGGAHITGQLIWREYFYT

MSVNNPNYDRMEGNEICLSIPWAKPKEDLLQRWRLGQTGFPLIDGAMRQLLAEGWLHHTLRNTVATFLTRGGLWQSWEHG

LQHFLKYLLDADWSVCAGNWMWVSSSAFERLLDSSLVTCPVALAKRLDPDGTYIKQYVPELVKVPKEFVHEPWRMSAEQQ

EQYECLIGVHYPERIIDLTMAVQRNMLAMKALRNSLITPPPHCRPSNEEEVRQFFWLADVVV

>Drosophila_ere_CRY-2_XP_001974136.1 XP_001974136.1

MDVKRSTLVHWFRKGLRVHDNPALSHIFSAANAAPGKYFVRPIFILDPGILDWMQVGANRWRFLQQALEDLDNQLRKLGS

RLFVVRGKPAEVFPRIFKSWRVEILTFETDIEPYSLSRDASVQKLAKAAGVKVETHCSHTIYNPELVIAKNLGKAPITYQ

KFLGIVEQLKLPKVLGAPEKLQNITTPPNDEVEQEDSAAYDCPTMEQLVKRPEELGPNKFPGGETEALRRMEDSLKDELW

VARFEKPNTAPNSLEPSTTVLSPYLKFGCLSARLFNQKLKEIIKRQPKHSQPPVSLIGQLMWREFYYTVAAAEPNFDRMM

GNVYCMQIPWQEHPNHLEAWTHGRTGYPFIDAIMRQLRQEGWIHHLARHAVACFLTRGDLWISWEEGQRVFEQLLLDQDW

ALNAGNWMWLSASAFFHQYFRVYSPVAFGKKTDPQGHYIRKYVPELSKYPSGCIYEPWKASLADQRAYGCVLGTDYPHRI

VKHEVVHKENIKRMGAAYKVNREVRTGKEEESSFEEKSESSTSGKRKVRKAAGNAPKRKR

>Drosophila_ere_PL_X1_XP_026835478.1 XP_026835478.1

MFTKASYWRESFKIFLQLQAMKRTKAQKAGPSKKAPKSEESRSKPKSDAESNDEEASTSKTSLVSKPDYQNFEQFLTHLE

HQRICTAANIQEFPFKKKRVRVLSKSDDVKESSLGGVVYWMSRDGRVQDNWALLFAQRLAFKLELPLSVVFCLVPKFLNA

TIRHYKFMMGGLQEVEQQCRALDIPFHLLMGPAVEKLPEFVKSKDIGAVVCDFAPLRLPRQWVEDVGKALPKSVPLVQVD

AHNVVPLWVASDKQEYAARTIRNKINSKLGEFLSEFPPVVRHPYGTGCKNVNVVDWSAAYASLQCDMEVGEVQWATPGYK

GACQQLYEFCSRRLRHFNDKRNDPTADALSGLSPWLHFGHISAQRCALEVQRFRGQHKASADAFCEEAIVRRELADNFCF

YNEHYDSLKGLSTWAYQTLDAHRKDKRDPCYSLEELEKSLTYDDLWNSAQLQLVREGKMHGFLRMYWAKKILEWTATPER

ALEYAILLNDKYSLDGRDPNGYVGCMWSIGGVHDMGWKERAIFGKVRYMNYQGCRRKFDVNAFVMRYGGKVHKK

>Drosophila_eug_CRY-2__XP_017082456.1 XP_017082456.1

MEAERRTLVHWFRKGLRVHDNPALSQIFTAANAEPGKFIIRPIFILDPGILDWMQVGANRWRFLQQTLEDLDKQLRKLNS

RLFVVRGKPADVFPRIFEQWRVEILTFETDIEPYSLSRDAAVQKLAKSANVKVETHCSHTIYNPEIVIAKNFGKAPITYQ

KFLGIVEQLKVPKVLGIPEKLKSRNEPKKDDVEQEDSEAYDCPTMEQLVKRPEELGPNKFPGGETEALLRMDESLKDELW

VARFEKPNTAPNSLEPSTTVLSPYLKFGCLSARLFHQKLKEILKRQTKHSQPPVSLIGQLLWREFYYTVAAAEPNFDRML

GNVYCLQIPWQEQPDHLKAWTHGRTGYPFIDAIMRQLRLEGWIHHLARHAVACFLTRGDLWISWEEGQRVFEQLLLDQDW

ALNAGNWMWLSASAFFHQYFRVYSPVAFGKKTDPQGHYIRKYVPELSKYPAGCIYEPWKASLADQRAYGCVLGTDYPHRI

VKHEVVHKENIKRMGAAYKVNREVRTGKEEVSFEEPETSTSGKRKVRKATGNAPKRKR

>Drosophila_eug_LOW_QU_XP_017085005.2 XP_017085005.2

MATRGANVIWFRHGLRLHDNPALLAALADKDQGIALIPIFIFDGESAGTKCVGYNRMRFLLDSLQDIDDQIQAATEGRGR

LHVFQGEPAHIFRRLHEQVRLHRICIEQDCEPIWNDRDETIRSLCRELNIDLVEKVSHTLWDPRTVIDTNGGIPPLTYQM

FLHTVQIIGLPPRPAADARLDDASFIELAPELRRSLGYFEKLPTPEHFNVFGDNMGFLAKINWRGGETQALVLLEERLKV

EQHAFERGFYLPNQALPNILETPKSMSAHLRFGCLSVRRFYWSVHDLFKNVQLRACVRGVQMTGGAHITGQLIWREYFYT

MSVNNPNYDRMEGNEICLSIPWAKPNEDLLQRWRLGQTGFPLIDGAMRQLLAEGWLHHTLRNTVATFLTRGGMWQSWEHG

LQHFLKYLLDADWSVCAGNWMWVSSSAFERLLDSSLVTCPVALAKRLDPEGAYIKQYVPELMNVPKEFVHEPWRMSPEQQ

EKYECLIGVHYPERMIDLSLAVKRNMLAMKALRNSLITPPPHCRPSNEEEVRQFFWLADVAI

>Drosophila_eug_PL-lik_XP_017079323.1 XP_017079323.1

MFRSLKQLIGLPSCHSIMFTFASHWRESFKIDFSLHIMKRAKVTKAGPSKKVAKKQDGSPKKDATSEQESSDAEASTSKS

SLASKPDYQNFEQFLTHLDHQRSCAASNIKDFSFRKKRVRVLSKTDDVKESCQGGIVYWMSRDGRVQDNWALLFAQRLAL

KLDLPLSVVFCLVPKFLNATIRHYKFMMGGLQEVEQQCRSLDIPFHLLMGPAVEKLPEFVKSKDIGAVICDFAPLRLPRQ

WVEDVGIALPKSVPLVQVDAHNVVPLWVASDKQEYAARTIRNKINSKLGEFLTEFPPVVRHPHGTGCKDVKPVDWSAAYA

MLQCDMDVDEVEWAKPGYKAACKQLYEFCSRRLRHFNDKRNDPMADVLSGLSPWLHFGQISAQRCALEVQRFRTQHKASA

DAFCEEAIVRRELADNFCFYNEHYDSLKGLSSWAYQTLDAHRKDKRDPCYSLEELEKSLTYDDLWNSAQLQLVREGKMHG

FLRMYWAKKILEWTATPEEALEYAILLNDKYSLDGRDPNGYVGCMWSIGGVHDMGWKERPIFGKVRYMNYQGCRRKFDVN

AFVMRYGGKVHKKK

>Drosophila_fic_CRY-1_XP_017060772.1 XP_017060772.1

MATRGANVIWFRHGLRLHDNPALLSALADKDQGVSIVPIFIFDGESAGTKCVGFNRMRFLLDSLQDIDEQLQAATEGRGR

LHIFEGEPAYIFRRLHEQFRIYKICIEQDCEPIWSDRDESIRSLCRELGIDLVEKVSHTLWDPRTVIDTNGGIPPLTYQM

FLHTVQIIGLPPRPVADARLEDASFVELDPELRRSLGYFEKLPSPEHFNIYSDNMGFLAKINWRGGETQALLLLEERLKV

EQHAFERGFYLPNQALPNILDTPKSMSAHLRFGCLSVRRFYWNVHDLFKNVQLRACVRGVQMTGGAHITGQLIWREYFYT

MSVNNPNYDRMENNEICLSISWAKPNEDLLQRWRLGQTGFPLIDGAMRQLLAEGWLHHTLRNTVATFLTRGGMWQSWEHG

LQHFLKYLLDADWSVCAGNWMWVSSSAFERLLDSSLVTCPVALAKRLDPDGTYIKQYVPELANVPKEFVHEPWRMSAEQQ

EQYECLIGVHYPERIIDLSLAVKRNMLAMKALRNSLITPPPHCRPSNEEEVRQFFWLADVTF

>Drosophila_fic_CRY-2_XP_017043962.1 XP_017043962.1

MDAKQRTLVHWFRKGLRVHDNPALSQIFTAANAAPGKYFVRPIFILDPGILDWMQVGANRWRFLQQTLEDLDKQLRKLDS

RLFVVRGKPAEVFPRIFKSWRVELLTFETDIEPYSLSRDSAVQKLAKVDGVKVETHCSHTIYNPDLVIAKNLGKAPITYQ

KFLGIVDKLKVPNVLGVPEKLKTRLDPPKDEVEQDDSTAYDVPTLEQLVKRPEELGPNKFPGGETEALRRMKDSLSDELW

VARFEKPNTAPNSLEPSTTVLSPYLKFGCLSARLFNEKLKEILKRQPKHSQPPVSLIGQLMWREFYYTVAAAEPNFDRML

GNVYCLQVPWQEQPEHLKAWTYGRTGYPFIDAIMRQLRQEGWVHHLARHAVACFLTRGDLWISWEEGQRVFEQLLLDQDW

ALNAGNWMWLSASAFFHQYFRVYSPVAFGKKTDPQGHYIRKYVPELAKYPAGCIYEPWKASLADQRAYGCVLGTDYPHRI

VKHEIVHKENIKRMGAAYKVNREVRTGKEEDSFEEPETSTSGKRKVRKATGNAAKRGKR

>Drosophila_fic_PL-lik_XP_017061488.1 XP_017061488.1

MFTLSSYWRESFKIVLQLNTMKRTKTAKAGSSKKAAKSQGGSRKEEPKSDQDGREKEASPSKVSLASKPDYQNFDQFLIH

LEDQRCIAAASIQEFSFRKKRARVLSETEDVEDSCQGGIVYWMSRDCRVQDNWALLFAQRLALKLELPLSVVFCLVPKFL

NATIRHYKFMIGGLQEVEQQCRSVNVPFYLLMGSAVDQLPDFVKSKGIGAVVCDFSPLRVPRKWVEDVGNSLPKSVPLVQ

VDAHNVVPLWVASDKQEYAARTIRNKINSKLGEFLSEFPPVVPHPHGTGCKDLKPVDWPAAYATLQCDMDVDEVAWAKPG

YKAGCKQLYEFCTRRLRHFNDKRNDPMADALSGLSPWLHFGQISAQRCALEVQRFRGQFKASADAFCEEAIVRRELADNF

CFYNEHYDSLKGLSSWAFETLEAHRKDKRDPCYGLEELEKSLTYDDLWNSAQLQLVREGKMHGFLRMYWAKKILEWTATP

EQALEYAILLNDKYSLDGRDPNGYVGCMWSIGGIHDMGWKERAIFGKVRFMNYQGCKRKFDVNAFVMRYGGKVHKKK

>Drosophila_gri_CRY-1_XP_001994667.1 XP_001994667.1

MTLRGANVIWFRHGLRLHDNPALLSALADKDYGIALIPIFIFDGESAGTKSVGYNRMRFLLDSLDDIDKQLKEVSDGQGG

LLICEGQPTDIFRRLHDQVRLHKICVELDCEPIWNERDEAINNLCRELGIEYVEMVSHTLWDPRTVIDTNGGIAPLTYQM

FLHTVHIIGLPPRPVHDPQFDGVGFVQLSAEMRREIGCLEKTPSPEHFNVYSDNMGTLAKINWVGGESQALLLLGERLKV

EQHAFERGYYLPNQALPNIVDKPKSMSAHLRFGCLSVRRFYWNVHDLFKNVQLRACIRGVQMTSGAHITGQLIWREYFYT

MSVNNPQYDRMEGNEICLNIPWAKPNEDHLQRWRLGQTGFPLIDAAMRQLLAEGWLHHTLRNTVATFLTRGGLWQNWEFG

LQHFLKYLLDADWSVCAGNWMWVSSSAFERLLDTSQVSCPVALAKRFDPNGVYIKQYVPELRNVTKEFIHEPWRMTAEQQ

EIAECLIGVHYPERIIDLSLASKRNMAAMKALRNSLIEPTPHCRPSNEEEVRQFFWLDE

>Drosophila_gri_CRY-2_XP_001987903.1 XP_001987903.1

MTAQRRTLVHWFRKGLRVHDNPALFQIFEVARAAPEKFYVRPIFILDPGILDWMQVGANRWRFLQQSLSDLDQQLRALNT

RLFVVRGKPVDIFPGLFERWQVQLLTYETDIEPYAVLRDAAVQQLAASQGVKVDTHCSHTIYNPELVIVRNLGKPPITYQ

KFLGIVEKLKLPKVLNKPKKLPDGMQPLADSDIYDYPTLEQLVKRPEDLGINKFPGGESEALRRMEASLADEQWVAKFEK

PKTAPNSLEPSTTVLSPYLKFGCLSARLFHERLQEILARQPKHSKPPVSLVGQLLWREFYYTVAAAEPNFDRMLGNAYCL

QIPWQEQPEHLEAWAHGRTGYPFIDAIMRQLRQEGWIHHLARHAVACFLTRGDLWISWEEGQRVFEQLLLDQDWALNAGN

WMWLSASAFFHQYFRVYSPVAFGKKTDPKGDYIKKYVPELAKYPAKCIYEPWKATLGEQRDYGCVLGSDYPHRIVNHDVV

HKENIKRMSDAYKVNREVRTGKQEEGHEEEEEYEEHPTGKRKASSKVTGSKLKHKLLK

>Drosophila_gri_GH2129_EDW01185.1 EDW01185.1

MKRTKTRSAAGAGKKKLIKDESSSDDEETVQVQSPTEKSKLSYKNFEQFISHMQEQRIASASNVHEFAFRKRRVHVLSNA

ADVKEQCKGGVVYWMSRDARVQDNWALLFAQRLALKLELPLSVVFCLVPKFLNATLRHYMFMLGGLQEVEQQCRDLNISF

QLLLGPAVERLPAFVVAEEIGAVVCDFAPLRLPRQWVTDVVKALPRNVPLTQVDAHNIVPLWVTSEKQEYAARTIRNKIN

SKLSEYLTEFPPVIKHPHGNGQKDKPVDWAAAEALLTCDRSVDVVDWAKPGYTAGSRQLYEFCTRRLRYFDEKRNDPTAD

ALSGLSPWLHFGQISAQRCALEVQRYTSMHKASADAFCEEAIVRRELADNFCYYNEHYDSLKGLHDWAYQTLQEHRTDER

NPCYSLEELEQSRTYDDLWNSAQLQLVKEGKMHGFLRMYWAKKILEWTATPELALEYCILLNDKYSLDGRDPNGYVGCMW

SIGGLHDQGWRERAIFGKIRFMNYQGCKRKFDVNAYVMRWGGKVHKKQEK

>Drosophila_gua_CRY-1_XP_034131710.1 XP_034131710.1

MAARGANVLWFRHGLRLHDNPALLAALEEKDQGIALIPVFIFDGESAGTKSVGYNRMRFLLDSLQDIDEQLQAATEGRGR

LFVFEGEPTLIFRRLHEQVRLHKICAELDCEPIWNERDEAARLMCRELGIEYVEKVSHTLWDPRLVIETNGGIPPLTYQM

FLHTVQIIGVPPRPAVDAHIDDATFIQLAPELRQHLGCFDKIPNPEHFNIYSDNMGFLAKINWRGGETQALALLEERLKV

ERNAFERGYYLPNQAIPNIQEAPKSMSAHLRFGCLSVRRFYWSVHDLFENVQLAACVRGVQMAGGAHITGQLIWREYFYT

MSVNNPNYDRMEGNEICLSIPWAKPDKNLLQRWRLGQTGFPLIDGAMRQLLAEGWLHHTLRNTVATFLTRGGLWQSWEPG

LQHFLKYLLDADWSVCAGNWMWVSSSAFERLLDSSLVTCPVALAKRLDPEGVYIRRYVPELKNLPREFIHEPWRLSAEQQ

VQYECLVGVHYPERVIDLSKAVKRNMMAMTSLRNSLITPPPHCRPSNEEEVRQFFWLANY

>Drosophila_gua_CRY-2_XP_034126312.1 XP_034126312.1

MMDAKHATLVHWFRKGLRVHDNPALSQIFATANVAPEKFHVRPIFILDPGILDWIQVGANRWRFLQQTLDDLDQQLRQLH

SRLFVVRGQPVDVFPRIFKSWRVELLTFETDIEPYALKRDAAVQKLAKDAGIKVDTHCSHTIYNPELVIAKNMGKAPITY

QKFLSVVEQLKTPKVLELPEKLSKKALPPKDEVEQHDEAAYDCPTLQQLVKRPEDLGPNKFPGGETEGLRRMKESLRDEL

WVARFEKPNTAPNSLEPSTTVLSPYLKFGCLSARLFHQQLKEILKRQPKHSQPPVSLIGQIMWREFYYTVAAAEPNFDRM

VGNVYCLQIPWQEQADHLEAWTHGRTGYPFIDAIMRQLRQEGWIHHLARHAVACFLTRGDLWISWEEGQRVFEQLLLDQD

WALNAGNWMWLSASAFFHQYFRVYSPVAFGKKTDPQGHYIRKYVPELAKYPNGCIYEPWKATLADQRAYGCVLGVDYPHR

IVKHELVHKENIKRMSAAYKVNREVRTGKAEDSPEADRSVAGKRKVAKSTGNAAKRKR

>Drosophila_gua_PL-lik_XP_034122802.1 XP_034122802.1

MFISSYWRKIFNKPNRYTVGLNLVRRLASIYTMKRTKTGTAGASKKAPKKEKKSAEPEEAASGSSGDEAVASSSKLAQSK

PEYKNFDQFLAYLEQQRCETAASVQEFPFKKKRVRVLSKANDVGENCPGGVVYWMLRDARVQDNWALLFAQRLALKLELP

LAVVFCLVPKFLNANIRHYKFMMGGLQEVEQQCRELGIPFHVLLGSAVDRLPEFVRSKHVGAVVCDFSPLRGPRKWVEDV

AKALPKNVPLTQVDAHNVVPLWVTSDKQEYAARTIRNKINSKLGEFLSDFPPVIKHPHGEGCKKVKPIDWPAAYAMLECD

MDVDEVQWAKPGYKAACLQLYEFCSRRLGKFNDKRNDPTVDALSGLSPWLHFGQISAQRCALEVQRYRSQHKASADAFCE

EAIVRRELADNFCYYNENYDNLKGLSSWALQSLEAHRKDKRDPCYTLEELEQSLTYDDLWNSAQLQLVREGKMHGFLRMY

WAKKILEWTATPEQALEYSILLNDKYSLDGRDPNGYVGCMWSIGGVHDMGWKERAIFGKIRYMNYQGCKRKFDVNAFVMR

YGGKVHK

>Drosophila_gun_hypoth_KAI8037274.1 KAI8037274.1

MFLVYSASPISRGCVNGLFQPLTADRAATARRYVMFTLAINWRECFKIILPLHTMKRAKTTQAGPSKKATKKQEGNRKDE

PKDSQESSEEEASTSKASFASKPDYQNFDQFLTHLEVQRFSTADSIKEFSFRKKRVRVLSKNEDVKESSKGGVVYWMSRD

GRVQDNWALLFAQRLALKLELPLSVVFCLVPKFLNATIRHYKFMMGGLQEVEQQCRTLDISFHLLIGPPVERLPEFVKTN

DIGAVVCDFSPLRLPRQWVDDVGKALPKSVPLVQVDAHNVVPLWVASDKQEYAARTIRNKINSKLNEFLSEFPPVVPHPH

GSGCKRSKPVDWPAAYAKLQCDMDVDEVQWAQPGYKAACEQLYEFCSLRLRHFNDKRNDPTADALSGLSPWLHFGQISAQ

RCALEVQRFRGQHKASADAFCEEAIVRRELADNFCYYNEHYDSLKGLSSWAYQTLDAHRKDKRDPCYNLEELEKSLTYDD

LWNSAQLQLVREGKMHGFLRMYWAKKILEWTATPELALEYAILLNDKYSLDGRDPNGYVGCMWSIGGIHDMGWKERAIFG

KVRYMNYQGCRRKFDVNAFVMRYGGKVHKKK

>Drosophila_gun_hypoth_KAI8043096.1 KAI8043096.1

MGAERSTLVHWFRKGLRVHDNPALSQIFAAANAAPGTFFVRPIFILDPGILDWMQVGANRWRFLQQTLSDLDQQLRKLDS

RLFVVRGKPAEVFPRIFKSWRVELLTFETDIEPYSLARDEAVQKLAKSAGVKVETHCSHTIYNPELVIARNLGKAPITYQ

KFLGIVEQLKIPKVLGVPEKLKIINEPTNDEVEQEDSTAYECPTMEQLVKRPEELGPNKFPGGETEALRRMDESLSDELW

VARFEKPNTAPNSLEPSTTVLSPYLKFGCLSARLFHQRLKEILKRQTKHSQPPVSLVGQLMWREFYYTVAAAEPNFDRML

GNVYCLQIPWQEQPNHLKAWTHGRTGYPFIDAIMRQLRQEGWIHHLARHAVACFLTRGDLWISWEEGQRVFEQLLLDQDW

ALNAGNWMWLSASAFFHQYFRVYSPVAFGKKTDPQGHYIRKYVPELSKYPAGCIYEPWKASLADQRAYGCVLGTDYPHRI

VKHEIVHKENIKRMGAAYKVNREVRTGKEEASFEEKPETSTSGKRKARKAVENAPKRKR

>Drosophila_gun_hypoth_KAI8045421.1 KAI8045421.1

MEMATRGANVMWFRHGLRLHDNPALLAALADKDQGIALIPVFIFDGESAGTKCVGYNRMRFLLDSLQDIDEQLQAATEGR

GRLHVFEGEPAHIFRRLHEQVRLHRICMEQDCEPIWNDRDESVRSLCRELKIDFVEKVSHTLWDPRTVIDTNGGIPPLTY

QMFLHTVQIIGLPPRPAADAQLDNASFVELAPEMRQSLGYFERLPTPEHFNVYGDNMGFLAKINWRGGETQALLLLEERL

KVEQHAFERGFYLPNQASPNILDTPKSMSAHLRFGCLSVRRFYWCVHDLFKNVQLRACVRGVQMTGGAHITGQLIWREYF

YTMSVNNPNYDRMDGNEICLSIPWAKPNEDLLQRWRLGQTGFPLIDGAMRQLLAEGWLHHTLRNTVATFLTRGGMWQSWE

HGLQHFLKYLLDADWSVCAGNWMWVSSSAFERLLDSSLVTCPVALAKRLDPEGAYIKHYVPELMNVPKEFVHEPWRMSAE

QQEQYECLIGVHYPERIIDLSLAVKRNMQAMKALRNSLMTPPPHCRPSNEEEVRQFFWLADGAV

>Drosophila_hyd_CRY-1__XP_023163819.1 XP_023163819.1

MAAERRTLVHWFRKGLRVHDNPALMQIFNKARASPDKFYVRPIFVLDPGILDWMQVGANRWRFLQQILVDLDQNLKELNS

RLFVIRGKPVDIFPNLFDRWHVELLTYETDIEPYAVKRDVAVQNIAAAHGVTVDTHCSHTIYNPEVVIAKNLGRAPVTYQ

KFLSVVEKLKLPKVLDKPERLPKGTQPIADEFEAAEPDVYDCPTLDQLVKRPQELGINKFPGGETEALRRLEKSLSDEHW

VASFEKPNTAPNSLEPSTTVLSPYLKFGCLSARLVHQRLQEILKRHPKHSKPPVSLVGQILWREFYYTVAAAESNFDRML

GNIYCLQIPWQERPDHLDAWAHGRTGYPFIDAIMRQLRQEGWIHHLARHAVACFLTRGDLWISWEDGQRVFEQLLLDQDW

ALNAGNWMWLSASAFFHQYFRVYSPVAFGKKTDRTGAYIRKYVPELAKYPAACIYEPWKATLADQRDFGCVLGVDYPHRI

VNHDIVHKENIKRMSAAYKVNREMRTGKTEDEIDSDSSLPMTTGKRKASFETKRSNIKRKR

>Drosophila_hyd_LOW_QU_XP_023171160.2 XP_023171160.2

MALRGANVIWFRHGLRLHDNPAMLAALSNKDLGVALIPIFIFDGESAGTKNVGYNRMRFLLDSLQDIDRQLQEATEGRGG

LLICQGQPVQIFRRLHXHVRLHKICVEQDCEPIWNERDEATKNLCRDLGIEYVEKVSHTLWDPRTVIDTNGGIAPLTYQM

FLHTVQIIGLPPRPVRDPHFEGVSFVQLSPELRNDIGCFEMIPTPEHFNVYSDNMGYLAKINWVGGETQALLLLGERLKV

EQHAFERGYYLPNQAMPNILDTPKSMSAHLRFGCLSVRRFYWCMHDLFKNVQLRACVRGVQMSGGAHITGQLIWREYFYT

MSVNNPQYDRMEGNEICLSIPWAKPDADQLQRWRLGQTGFPLIDSAMRQLLAEGWLHHTLRNTVATFLTRGGLWQNWEFG

LQHFLKYLLDADWSVCAGNWMWVSSSAFERLLDSSLVTCPVALAKRLDPNGQYIKQYVPELQHVPKEFIHEPWLMSADQQ

KRYECLIGVHYPDRIIDLSLASKRNMLAMKALRNSLIEPPPHCRPSNEEEVRQFFWLAD

>Drosophila_hyd_PL-lik_XP_023171500.1 XP_023171500.1

MQIGGSLRSPFSFLGGFRITFQLLPMKRNKSNAASSAKKRPKNNERSSSSTSKSSGSGSGSDEETVTITPEKKTKPNYNS

FDQFLSHIQEQRLKAAPNIHEFAFRKKRVRVLSSASDVNEQYKGGVVYWMSRDARVQDNWALLFAQRLALKLELPLTVVY

CLVPKFLNATLRHYKFMLGGLQEVERQCRELNISFKLLLGPAVERLPQFVNDEQIGAVICDFAPLRLPRQWVEDVVKALP

ANVPLTQVDAHNVVPLWVTSEKQEYAARTIRNKINSKLSEYLTEFPPLIKHAHGNVKGSHGVDWTAAHKMLSCDMSVDAV

EWAQPGYTSACKQLYDFCTRRLRLFYDKRNDPMADALSGLSPWLHFGQISAQRCVLEVSRYKTLFKASAEAFCEEAIVRR

ELADNFCYYNEHYDNLKGLHSWAYETLQTHRKDKRSPCYTLEELEQSRTYDDLWNSAQLQLVKEGKMHGFLRMYWAKKIL

EWTETPEQALEYSILLNDKYSLDGRDPNGYVGCMWSIGGIHDQGWKERAIFGKIRYMNYQGCKRKFDVNAFVMRYGGKVH

KKEN

>Drosophila_imm_hypoth_KAH8263612.1 KAH8263612.1

MNAKCNQSSQRRTLVHWFRKGLRVHDNPALSQVFDVARSAPHAYSVRPIFLLDPGILDWMQVGANRWRFLQQTLCDLDKQ

LRELNSRLYVVRGKPVELFPELFKRWHVELLTYESDIEPYAVKRDAAVQRLAAADQVKVDTHCSHTIYNPELVMARNLGK

APITYQKFLGIVEQLKLPKVRDKPEKLPQEVQPLLDVLELADAHVYDCPTLAHLVKRPDELGVNKFPGGETEALRRLEAS

LSDEKWVASFEKPKTAPNSLEPSTTVLSPYLKFGCLSARLLYERLREILARHPKHSKPPVSLVGQLLWREFYYTVAAAEP

NFDRMMGNAYCMQIDWQEQPDHLAAWTHGRTGYPFIDAIMRQLRQEGWIHHLARHAVACFLTRGDLWISWEEGQRVFEQL

LLDQDWALNAGNWMWLSASAFFHQYFRVYSPVAFGKKTDPTGAYIRKYVPELAKYPANCIFEPWKATLSAQREYGCVLGV

DYPQRIVNHDIVHKENIKRMSAAYKVNREVRTGKQEDEEADKPLTGKRKASSTKSGVAAKRRR

>Drosophila_imm_hypoth_KAH8310473.1 KAH8310473.1

MALRSANVIWFRHGLRLHDNPALLSALADKDHGVALIPIFIFDGESAGTKSVGFNRMRFLLDSLQDIDQQLQSETDGRGR

LFIFQGNPVQIFRRLNEQVRLHKICVEQDCEPIWNERDDATKTLCSELGIEYVEKVSHTLWDPRTVIDTNGGIAPLTYQM

FLHTVQIIGLPPRPVHDPHFGGVSFVQLSPELRRDLGCFEQTPTPEHFNIYSDNMGYLAKINWIGGESRALELLGERLKI

EQHAFERGFYLPNQAMPNILDTPKSMSAHLRFGCLSVRRFYWSVHDLFKNVQLRACVRGVQMTGGAHITGQLIWREYFYT

MSVNNPNYDRMEGNEICLSIPWAKPDDEQLQRWRLGQTGFPLIDGAMRQLLAEGWLHHTLRNTVATFLTRGGLWQNWEFG

VQHFLKYLLDADWSVCAGNWMWVSSSAFERLLDSSLVTCPVALAKRLDPEGIYIKQYVPELQNVPKEFIHEPWRMSAEQQ

ERYECLIGVHYPERILDLSVAGKRNTLAMNSLRSSLITPPPHCRPSNEEEVRQFFWLAD

>Drosophila_inn_CRY-1_XP_034486152.1 XP_034486152.1

MALRTANVIWFRHGLRLHDNPALLSALSDRDHGVALIPIFIFDGESAGTKSVGFNRMRFLLDSLEDIDKQLQSETDGRGR

LLICQGQPVQIFRRLSEQVRLHKICVEQDCEPIWNERDESMKKLCKELGFEYVEKVSHTLWNPRTVIETNGGIAPLTYQM

FLHTVQIVGLPPRPVHDPHFGGVSFVQLSPELRRDLGCFEQTPSPEHFNIYSDNMGYLAKTKWVGGETQALTLLGERLKV

EQHAFERGYYLPNQARPNILDTPKSMSAHLRFGCLSVRRFYWSVHDLFKNVQLRACVRGVQMSGGAHITGQLIWREYFYT

MSVNNPQYGCMEGNEICLNIPWAKPNEDQLERWRLGQTGFPLIDGAMRQLLAEGWLHHTLRNTVATFLTRGGLWQNWEVG

VQHFLKYLLDADWSVCAGNWMWVSSSAFERLLDSSLVTCPVALAKRLDPNGLYIKQYVPELKNVPKEFIHEPWRMSAEQQ

ENYECLIGVHYPKRILDLSLATKRNMLAMKNLRDSLITPPPHCQPSNEKEVRQFFWLAD

>Drosophila_inn_CRY-2_XP_034474723.1 XP_034474723.1

MTAKRHTLVHWFRKGLRVHDNPALAQVFDVARASAEQYQVRPIFILDPGILDWMQVGANRWRFLQQTLWDLDQQLRALNS

RLYVVRGKPVDVFPQLFERWHVQLLTYETDIEPYAVQRDTAVQKLAAERGVKVDTHCSHTIYNPEVVIARNLGKAPITYQ

KFLGIVEKLKLPKVLTKPEKLPQGMQPSEDALEAADAHVYDCPTLEQLVKRPDELGINKFPGGETEALRRMEASLSNEHW

VANFEKPNTAPNSLEPSTTVLSPYLKFGCLSARLLYDRLREILARQPKHSKPPVSLVGQLLWREFYYTVGAAEPNFDRML

GNSYCMQIAWQEQPEHLKAWTHGHTGYPFIDAIMRQLRQEGWIHHLARHAVACFLTRGDLWISWEEGQRVFEQLLLDQDW

ALNAGNWMWLSASAFFHQYFRVYSPVAFGKKTDPTGEYIRKYVPELAKYPAGCIFEPWKATLSAQRDYGCVLGVDYPHRI

VNHDIVHKENIKRMSAAYKVNREVRTGKQEDDYDDEDDKPPQKSTGKRKAASRTTGGTAKRRR

>Drosophila_inn_PL-lik_XP_034477525.1 XP_034477525.1

MKTTVFGGFRLFFRTLHMKRSKPSSAGAAKKKPKKEVKESSSSGEETVATVPAKTFTPSYKNVEQFMSHIQDQRLASASN

VHEFAFKKKRVREVSGASDVREGCKGGVVYWMSRDGRVQDNWALLFAQRLALKLELPLTVVFCLVPKFLNATLRHYKFML

GGLQEVEQECLKLNISFKLLLGPAVERLPEFVQAEDIGAVVCDFAPLRLPRQWVSDVAKKLPGHVPITQVDAHNIVPLWV

TSEKQEYAARTIRNKINSKLQEFLTEFPPVIKHPHGKETRLQPVDWDAAYKMLTCDMSVDEVDWAKPGYTAACRQLYEFC

TRRLRHFDGKRNDPTANALSGLSPWLHFGQISAQRCILEVKRFAGMHKASADAFCEETIVRRELADNFCYYNEHYDSLKG

LHGWAYQTLDAHRQDKRSPCYTLEELEHSRTYDDLWNSAQLQLVKEGKMHGFLRMYWAKKILEWTATPEQALEYSILLND

KYSLDGRDPNGYVGCMWSIGGVHDQGWKERAIFGKIRYMNYQGCKRKFDVNAFVMRYGGKVHKKADN

>Drosophila_iro_hypoth_KAH8279595.1 KAH8279595.1

MDAQQRTLVHWFRKGLRVHDNPALAQVFALANSAPGKYSVRPIFILDPGILDWMQVGANRWRFLQQTLQDLDEQLRKLNS

RLFVVRGKPAEVFARILKSWRVELLTFETDIEPYSLTRDAAVQKLAKDEGVKVETHCSHTIYNPELVIAKNLGRAPITYQ

KFLGIVEQLKLPKVLQLPEKLLEAKEPPKDEVEESDAEAYDCPTMEQLVKRPEELGPNKFPGGETEALRRMEESLRDELW

VARFEKPNTAPNSLEPSTTVLSPYLKFGCLSARLFHQRLKEILKRQTKHSQPPVSLVGQLLWREFYYTVAAAEPNFDRML

GNVYCLQIPWQEQPDHLKAWAHGRTGYPFIDAIMRQLRQEGWIHHLARHAVACFLTRGDLWISWEEGQRVFEQLLLDQDW

ALNAGNWMWLSASAFFHQYFRVYSPVAFGKKTDPKGNYIRKYVPELAKYPAGSIYEPWKVSLGDQRAYGCVLGTDYPHRI

VKHELVHKENIKRMSAAYKVNREVRTGKQEDSFEEKPESSGKRKVAKATGNAAKRRR

>Drosophila_iro_hypoth_KAH8280088.1 KAH8280088.1

MATRGANVIWFRHGLRLHDNPALLAALADKDQGIALLPVFIFDGESAGTKCVGYNRMRFLLDSLQDIDTQLQARTEGRGR

LHVFEGDPAHIFRRLHDQVRLHKICFEQDCEPIWNQRDDNIRALCQELGIECVEKVSHTLWDPRTVINTNGGIPPLTYQM

FLHTVHIIGLPPRPTADAHLEDASFVDLSHELRRSLGHLEQIPTPEHFNIFSDNMGFLAKMIWRGGETQALLLLEQRLKV

EQHAFERGFYLPNQALPNIQDTPKSMSPHLRFGCLSVRRFYWSVHDLFQNVQLRASVRGVQMTGGAHITGQLIWREYFYT

MSVKNPHYDRMEGNEICLNIPWAKPDETKLEKWRLGQTGFPLIDAAMRQLLAEGWLHHVLRNTVATFLTRGGMWQSWEHG

VQHFLKYLLDADWSVCAGNWMWVSSSAFERLLDSSLVTCPVALAKRLDPDGAYIRQYVPELQNVPREFIHEPWRMSADQQ

QQYECLVGVHYPERILDLSRAVKSNMLAMKALRHSLIEPPPHCRPSNEEEVRNFFWLADLAV

>Drosophila_iro_hypoth_KAH8284650.1 KAH8284650.1

MLTLSSVWRQSFKKVSFSQIYTTQFNDLVVQQLHPPIMKRTKAAKAGPSKKPAKKKPESDTESSSGNEEKPSPSKTSIKP

KPEYQNFEQFLLHLEEQRSSTAANIREFSFLKKRVRVLSKSADVSESCQGGIVYWMSRDGRVQDNWAMLFAQRLALKLEL

PLSVVFCLVPRFLNATLRHYKFMMVGLQEVEQQCRALDIPFHLLLGPAVERLPEFVKSRQVGAVICDFAPLRVPRKWVED

VGQALPKSVPLVQVDAHNVVPVWVASDKQEYAARTIRNKINSKLGEFLTEFPPVIKHPHGTGCLKVDPVDWPAAYAMLQC

DMDVGEVQWAKPGYKAACQQLYDFCSRRLRHFNDKRNDPMADALSGLSPWLHFGQISAQRCALEVQRFRGQHKASAEAFC

EEAIVRRELADNFCFYNEHYDSLKGLSAWAYQTLDAHRKDKRDPCYSLEELEKALTYDDLWNSAQLQLVREGKMHGFLRM

YWAKKILEWSETPEQALEYAILLNDKYSLDGRDPNGYVGCMWSIGGVHDMGWKERSIFGKIRYMNYQGCKRKFDVNAFVI

RYGGKVHKQNKK

>Drosophila_jam_hypoth_KAH8281418.1 KAH8281418.1

MDGKKSTLVHWFRKGLRVHDNPALSQIFSAANASPEKFCVRPIFILDPGILDWMQVGANRWRFLQQTLQDLDEQLRKLNS

RLFVVRGKPAEVFPRIFKSWRVELLTFESDIEPYSLSRDAAVQKLAKAEGVKVVTHCSHTIYNPELVIAKNLGKAPITYQ

KFLGIVDQLKVPKVLEVPEKLKSVAEPPKDEVEQEDSAAYDCPTMEQLVKRPEELGPNKFPGGETEALRRMDDSLRDELW

VARFEKPNTAPNSLEPSTTVLSPYLKFGCLSARLFNQRLKDILKRQTKHSQPPVSLIGQLMWREFYYTVAAAEPNFDRML

GNVYCLQIPWQEHPGHLEAWTHGRTGYPFIDAIMRQLRQEGWIHHLARHAVACFLTRGDLWISWEEGQRVFEQLLLDQDW

ALNAGNWMWLSASAFFHQYFRVYSPVAFGKKTDPQGHYIRKYVPELAKYPKDCIYEPWKATLADQRAYGCVLGTDYPHRI

VKHEVVHKENIKRMSAAYKVNREVRTGKEEEDSFEEKSEASTSGKRKVRKAAGSAAKRKR

>Drosophila_jam_hypoth_KAH8291068.1 KAH8291068.1

MFRIASNWRECFKIITPLRTMKRTKAAKAGPSKKVAKKQEKSPKKESSDQDPSSGSGDEAPSTSKASLSKPDYQSLEQFM

AHLEHQRSATAANIQEFPFRKKRARVLSSSADVKESCQGGVVYWMSRDARVQDNWALLFAQRLALKLELPLTVVFCLVPK

FLNATMRHYKFLMGGLEEVEQQCRSLDIPFHLLLGPAVQRLPEFVRTQDLGAVVCDFAPLRLPRQWVEDVGKALPKSVPL

VQVDAHNVVPLWVASDKQEYAARTIRTKINSKLGEFLSEFPPVVKHPHGRGCQDVKPVDWAAAYATLQCDTDVGEVEWAK

PGYKAACQQLYEFCSRRLRHFNDKRNDPMADALSGLSPWLHFGQISAQRCALEVQRFRGQHKASADAFCEEAIVRRELAD

NFCYYNEHYDSLKGLTPWAYQTLDAHRKDKRDPCYSLEELEKSLTYDDLWNSAQLQLVREGKMHGFLRMYWAKKILEWSP

TPEQALEYAILLNDKYSLDGRDPNGYVGCMWSIGGVHDMGWKERAIFGKIRYMNYQGCRRKFDVNAFVMRYGGKVHKRKG

KE

>Drosophila_jam_hypoth_KAH8292437.1 KAH8292437.1

MAARGANVIWFRHGLRLHDNPALVAALADKDQGIALIPVFIFDGESAGTKCVGYNRMRFLLDSLQDIDDQIQAATEGRGR

LHVFEGEPVNIFRRLNEQVRLHRICMEQDCEPIWNSRDDSVRSLCRELGIDFVEKVSHTLWDPRMVIDTNGGIPPLTYQM

FLHTVQIIGLPPRPAADVHLEDVSFIELAPELRRNLGYFEEIPNPEHFNVYGDNMGFLAKINWRGGETQALLLLEERLKV

EQHAFERGFYLPNQALPNIHDTPKSMSAHLRFGCLSVRRFYWSVHDLFKNVQLRACVRGVQMTGGAHITGQLIWREYFYT

MSVNNPNYDRMDGNEICLSIPWAKPDENLLQRWRLGQTGFPLIDAAMRQLLAEGWLHHTLRNTVATFLTRGGLWQSWEHG

LQHFLKYLLDADWSVCAGNWMWVSSSAFERLLDSSLVTCPVALAKRLDPEGAYIKQYVPELMGVPKEFVHEPWRMSAEQQ

EQYECLIGVHYPERIIDLSKAVKRNMLAMTALRNSLITPPPHCRPSNEEEVRQFFWLADQTV

>Drosophila_kik_CRY-1_XP_017036029.1 XP_017036029.1

MATRGANVIWFRHGLRLHDNPALVAALGDKDQGIALIPVFIFDGESAGTKCVGYNRMRFLLDSLQDIDDQIQAATEGRGR

LHVFEGEPVNIFRRLNEQVRLHRICIEQDCEPIWSDRDDSVRSLCRELGIDFVEKVSHTLWDPRTVIDTNGGIPPLTYQM

FLHTVQIIGLPPRPAADVHLEDVSFVELAPEMRRNLGYFEQMPNPEHFNVYGDNMGFLAKINWRGGETQALLLLEERLKV

EQHAFERGFYLPNQALPNIHDTPKSMSAHLRFGCLSVRRFYWSVHDLFKNVQLRACVRGVQMTGGAHITGQLIWREYFYT

MSVNNPNYDRMEGNEICLSIPWAKPDEDLLQRWRLGQTGFPLIDAAMRQLLAEGWLHHTLRNTVATFLTRGGLWQSWEHG

LQHFLKYLLDADWSVCAGNWMWVSSSAFERLLDSSLVTCPVALAKRLDPEGAYIKQYVPELLGVPKEFVHEPWRMTAEQQ

EQYECLIGVHYPERIIDLSKAVKRNMLAMTALRNSLITPPPHCRPSNEEEVRQFFWLADQTV

>Drosophila_kik_CRY-2_XP_017022206.1 XP_017022206.1

MDGKRSTLVHWFRKGLRVHDNPALSQIFSAANAAPEKFCVRPIFILDPGILDWMQVGANRWRFLQQTLQDLDEQLRKLNS

RLFVVRGKPVEVFPRIFKTWRVELLTFESDIEPYSLSRDAAVQKLAQTEGVKVVTHCSHTIYNPELVISKNLGKAPITYQ

KFLGIVDQLKIPKVLEVPDKLKGVAQPPKDDVEQGTPAAYDCPTMEQLVKRPEELGPNKFPGGETEALRRMDESLKDEVW

VARFEKPNTAPNSLEPSTTVLSPYLKFGCLSARLFNQKLKEILKRQPKHSQPPVSLIGQLMWREFYYTVAAAEPNFDRML

GNVYCLQIPWQEQPDHLEAWTHGRTGYPFIDAIMRQLRQEGWIHHLARHAVACFLTRGDLWISWEEGQRVFEQLLLDQDW

ALNAGNWMWLSASAFFHQYFRVYSPVAFGKKTDPQGDYIRKYVQELAKYPKGCIYEPWKATLADQRAYGCVLGTDYPHRI

VKHEVVHKENIKRMSAAYKVNREVRTGKEDEDSFEEKPEASTSGKRKVRKTAGSAAKRKR

>Drosophila_kik_PL-lik_XP_017025192.1 XP_017025192.1

MYNIVSYWRECLKISLQITPLHTMKRAKAAKAGPSKKVAKKQDKSPKKESSDQDSSGGSGVEEPSISKVPLSKPDYHNLE

QFLAHLEHQRSATAANIHEFPFRKKRVRVLSKSGDVKESCQGGVVYWMSRDARVQDNWALLFAQRLALKLELPLTVVFCL

VPKFLNATIRHYKFLMGGLEEVEQQCRSLDIPFHLLLGPAVERLPEFMKSQDMGAVVCDFAPLRLPRQWVEDVGKALPQS

VPLVQADAHNVVPLWVASDKQEYAARTIRNKINSKLDEFLTEFPPVIKHPHGKGCQEVKPVDWPAAYASLQCDTDVGEVE

WAKPGYKAACQQLYEFCSRRLRHFNDKRNDPMADALSGLSPWLHFGQISAQRCALEVQRFRGQHKASADAFCEEAIVRRE

LADNFCYYNEHYDSLKGLTAWAYQTLDAHRKDKRDPCYSLEELEKSLTYDDLWNAAQLQLVREGKMHGFLRMYWAKKILE

WSPTPEQALEYAILLNDKYSLDGRDPNGYVGCMWSIGGVHDMGWKERAIFGKIRYMNYQGCRRKFDVNAFVMRYGAKVHK

RK

>Drosophila_mau_CRY-1_XP_033163524.1 XP_033163524.1

MATRGANVIWFRHGLRLHDNPALLAALADKDQGIAIIPVFIFDGESAGTKNVGYNRMRFLLDSLQDIDDQLQAATDGRGR

LLVFEGEPAYIFRRLHEQVRLHRICIEQDCEPIWNERDESIRSLCRELSIDFVEKVSHTLWDPQLVIETNGGIPPLTYQM

FLHTVQIIGLPPRPTADARLDDATFVELDPEFSRSLKLFERLPTPEHFNVYGDNMGFLAKINWRGGETQALLLLDERLKV

EQHAFERGFYLPNQALPNIHDSPKSMSAHLRFGCLSVRRFYWSVHDLFKNVQLRACVRGVQMTGGAHITGQLIWREYFYT

MSVNNPNYDRMEGNEICLSIPWAKPNEDLLQRWRLGQTGFPLIDGAMRQLLAEGWLHHTLRNTVATFLTRGGLWQSWEHG

LQHFLKYLLDADWSVCAGNWMWVSSSAFERLLDSSLVTCPVALAKRLDPDGTYIKQYVPELMNVPKEFVHEPWRMSAEQQ

EQYECLIGVHYPERIIDLSIAVKRNMLAMKSLRNSLITPPPHCRPSNEEEVRQFFWLADVVV

>Drosophila_mau_LOW_QU_XP_033163501.1 XP_033163501.1

MDAQRSTLVHWFRKGLRVHDNPALSHIFTAANAAPGKYFVRPIFILDPGILDWMQVGANRWRFLQQTLEDLDNQLRKLDS

RLFVVRGKPAEVFPRIFKSWRVEMLTFETDIEPYSLTRDAAVQKLAKAEGVKVETHCSHTIYNPELVIAKNLGKAPITYQ

KFLGIVDQLKVPKVLGLPEKLKKMPTPPKDEVEQKDSAAYDCPTMEQLVKRPEELGPNKFPGGETEALRRMEESLKDEIW

VARFEKPNTAPNSLEPSTTVLSPYLKFGCLSARLFNQKLKEIIKRQPKHSQPPVSLIGQLMWREFYYTVAAAEPNFDRML

GNVYCMQIPWQEHPDHLEAWTHGRTGYPFIDAIMRQLRQEGWIHHLARHACVACFLTRGDLWISWEEGQRVFEQLLLDQD

WALNAGNWMWLSASAFFHQYFRVYSPVAFGKKTDPQGHYIRKYVPELSKYPAGCIYEPWKASLADQRAYGCVLGTDYPHR

IVKHEVVHKENIKRMGAAYKVNREVRTGKEEESSFEEKSESSTSGKRKVRRAAGSAPKRKR

>Drosophila_mau_PL_X1_XP_033154085.1 XP_033154085.1

MFTLASYWRESFKIVLPLQTMKRTKAQKAGPSKRAAKSEKASSKPKSDQESSDEEASTSKASLVSKPDYQNFEQFLTHLE

HQRVCTAASIQEFSFRKKRVRVLSKTEDVKESSLGGVVYWMSRDGRVQDNWALLFAQRLALKLEIPLTVVFCLVPKFLNA

TIRHYKFMMGGLQEVEQQCRALDIPFHLLMGPAAEKLPQFVKSNDIGAVVCDFAPLRLPRQWVDDVGKALPKSVPLVQVD

AHNVVPLWVASDKQEYAARTIRNKINSKLGEYLSEFPPVVRHPHGTGCKNVQAVDWSAAYASLQCDMEVDEVQWAKPGYK

AACQQLYEFCSRRLRHFNDKRNDPTADALSGLSPWLHFGHISAQRCALEVQRFRGQHKASADAFCEEAIVRRELADNFCF

YNEHYDSLKGLSSWAYQTLDAHRKDKRDPCYSLEELEKSLTYDDLWNSAQLQLVREGKMHGFLRMYWAKKILEWTATPEQ

ALEYSILLNDKYSLDGRDPNGYVGCMWSIGGVHDMGWKERAIFGKVRYMNYQGCRRKFDVNAFVMRYGGKVHKKK

>Drosophila_mel_BAA12067.1_photolyase BAA12067.1

MDSQRSTLVHWFRKGLRLHDNPALSHIFTAANAAPGKYFVRPIFILDPGILDWMQVGANRWRFLQQTLEDLDNQLRKLNS

RLFVVRGKPAEVFPRIFKSWRVEMLTFETDIEPYSVTRDAAVQKLAKAEGVRVETHCSHTIYNPELVKAKNLGKAPITYQ

KFLGIVEQLKVPKVLGVPEKLKKMPTPPKDEVEQKDSAAYDCPTIKQLVKRPEELGPNKFPGGETEALRRMEESLKDEIW

VARFEKPNTAPNSLEPSTTVLSPYLKFGCLSARLFNQKLKEIIKRQPKHSQPPVSLIGQLMWREFYYTVAAAEPNFDRML

GNVYCMQIPWQEHPDHLEAWTHGRTGYPFIDAIMRQLRQEGWIHHLARHAVACFLTRGDLWISWEEGQRVFEQLLLDQDW

ALNAGNWMWLSASAFFHQYFRVYSPVAFGKKTDPQGHYIRKYVPELSKYPATCIYEPWKASLVDQRAYGCVLGTDYPHRI

VKHEVVHKENIKRMGAAYKVNREVRTGKEEESSFEEKSETSTSGKRKVRRATGSAPKRKR

>Drosophila_mel_NP_523653.2_photorepai NP_523653.2

MFTLASYWRESFKIVLPLQAMKRTKAQKAGPSKKAAKNEKASSEPKSDQESSDEEASTSKALLVSKPDYQNFEQFLTHLE

HQRVCTAANIQEFSFRKKRVRVLSKTEDVKESSLGGVVYWMSRDGRVQDNWALLFAQRLALKLELPLTVVFCLVPKFLNA

TIRHYKFMMGGLQEVEQQCRALDIPFHLLMGSAVEKLPQFVKSKDIGAVVCDFAPLRLPRQWVEDVGKALPKSVPLVQVD

AHNVVPLWVASDKQEYAARTIRNKINSKLGEYLSVFPPVVRHPHGTGCKNVNTVDWSAAYASLQCDMEVDEVQWAKPGYK

AACQQLYEFCSRRLRHFNDKRNDPTADALSGLSPWLHFGHISAQRCALEVQRFRGQHKASADAFCEEAIVRRELADNFCF

YNEHYDSLKGLSSWAYQTLDAHRKDKRDPCYSLEELEKSLTYDDLWNSAQLQLVREGKMHGFLRMYWAKKILEWTATPEH

ALEYAILLNDKYSLDGRDPNGYVGCMWSIGGVHDMGWKERAIFGKVRYMNYQGCRRKFDVNAFVMRYGGKVHKKK

>Drosophila_mel_NP_732407.1_cry NP_732407.1

MATRGANVIWFRHGLRLHDNPALLAALADKDQGIALIPVFIFDGESAGTKNVGYNRMRFLLDSLQDIDDQLQAATDGRGR

LLVFEGEPAYIFRRLHEQVRLHRICIEQDCEPIWNERDESIRSLCRELNIDFVEKVSHTLWDPQLVIETNGGIPPLTYQM

FLHTVQIIGLPPRPTADARLEDATFVELDPEFCRSLKLFEQLPTPEHFNVYGDNMGFLAKINWRGGETQALLLLDERLKV

EQHAFERGFYLPNQALPNIHDSPKSMSAHLRFGCLSVRRFYWSVHDLFKNVQLRACVRGVQMTGGAHITGQLIWREYFYT

MSVNNPNYDRMEGNDICLSIPWAKPNENLLQSWRLGQTGFPLIDGAMRQLLAEGWLHHTLRNTVATFLTRGGLWQSWEHG

LQHFLKYLLDADWSVCAGNWMWVSSSAFERLLDSSLVTCPVALAKRLDPDGTYIKQYVPELMNVPKEFVHEPWRMSAEQQ

EQYECLIGVHYPERIIDLSMAVKRNMLAMKSLRNSLITPPPHCRPSNEEEVRQFFWLADVVV

>Drosophila_mir_CRY-1_XP_017145329.1 XP_017145329.1

MVPRGANVLWFRHGLRLHDNPALLAALEEKDQGIPLIPVFIFDGESAGTKSVGYNRMRFLLDSLQDLDEQLQSATEGRGR

LFVFEGEPTLIFRRLHEQVRLHKICAELDCEPIWNERDESARLLCRELGIEYVEKVSHTLWDPRLVIETNGGIPPLTYQM

FLHTVQIIGVPPRPAIDAHINDATFIQLAPELRQHLGCFDQVPNPEHFNIYSDNMGFLAKINWRGGETQALALLEERLKV

ERNAFERGYYLPNQANPNIQEAPKSMSAHLRFGCLSVRRFYWSVHDLFENVQLAACVRGVQIEGGAHITGQLIWREYFYT

MSVNNPNYDRMEGNEICLTIPWAKPDENLLQRWRLGQTGFPLIDGAMRQLLAEGWLHHTLRNTVATFLTRGGLWQSWEPG

LKHFLKYLLDADWSVCAGNWMWVSSSAFERLLDSSLVSCPVALAKRLDPEGVYIRRYVPELKNLPKEYIHEPWRLSAEQQ

VKYECLIGVHYPERIIDLSKAVKRNMMAMTALRNSLITPPPHCRPSNEEEVRQFFWLANY

>Drosophila_mir_CRY-2_XP_017154316.1 XP_017154316.1

MDAKHATLVHWFRKGLRVHDNPALTQIFSAANAAPEKFHVRPIFILDPGILDWMQVGANRWRFLQQTLHDLDQQLRKLNS

RLFVVRGKPVDVFPRVFKSWRVELLTFETDIEPYALQRDAAVQKLAKAEGIKVDTHCSHTIYNPELVIAKNMGKAPITYQ

KFLSVVDQLKVPKVLELPEQLAKKALPPKDEVEQQDDNAYDCPTLEQLVKRPEDLGPNKFPGGETEGLRRMKESLRDELW

VARFEKPNTAPNSLEPSTTVLSPYLKFGCLSARLFHQQLKAILKRQSKHSQPPVSLIGQLLWREFYYTVAAAEPNFDRML

GNVYCLQIPWQEQADHLEAWTHGRTGYPFIDAIMRQLRQEGWIHHLARHAVACFLTRGDLWISWEEGQRVFEQLLLDQDW

ALNAGNWMWLSASAFFHQYFRVYSPVAFGKKTDPQGHYIRKYVPELAKYPNGCIYEPWKATLADQREYGCVLGVDYPHRI

VKHELVHKENIKRMSAAYKVNREVRTGKEEDSPEATGKRKVAKTIGNAAKRKR

>Drosophila_mir_PL_XP_017147310.1 XP_017147310.1

MFIPTYWRPNFNNINCSSIVGLLVNNFVRQRAPIYTMKRAKSGTAGASKKAPKKEKKSAEPAASSSQESSSGGEEPASSS

KLVQSKPEYKNFEQFLAHLVQQRSESAVNIQEFPFKKKRVRVLSKVNDVRDKCTGGVVYWMSRDGRVQDNWALLFAQRLA

LKLEMPLAVVFCLVPKFLNATIRHYKFMMGGLQEVEEQCRELGIPFHLLLGPAVDRLPEFVRSKDVGAVVCDFAPLRVPR

KWVEDVAKALPKNVPLTQVDAHNVVPLWVASDKQEYAARTIRNKINSKLGEFLSDFPPVIKHPHGEGCKKVKPIDWPAAY

AMLECDMDVDEVKWAKPGYKAACLQLYEFCSRRLGKFNDKRNDPTVDALSGLSPWLHFGQISAQRCVLEVQRYRSQHKAS

ADAYCEEAIVRRELADNFCYYNENYDSLKGLSPWAYQSLEAHRKDKRDPCYTLEELEQSLTYDDLWNSAQLQLVREGKMH

GFLRMYWAKKILEWTATPEQALEYAILLNDKYSLDGRDPNGYVGCMWSIGGIHDMGWKERAIFGKIRYMNYQGCKRKFDV

NAFVMRYGGKVHKKKG

>Drosophila_moj_CRY-1_XP_001998330.1 XP_001998330.1

MAQCGANVIWFRHGLRLHDNPAMLAALSNKDQGVALIPIFIFDGESAGTKNVGYNRMRFLLDSLQDIDRQLQEATEGRGG

LLICEGQPVHIFRRLHEHVGLHKICVEQDCEPIWNERDEAVKSLCRELGIEYVEKVSHTLWDPRTVIDTNGGIAPLTYQM

FLHTVQIIGLPPRPVRDPNFEGVKFVQLSPELRHDIGCFEMTPTPEHFNVYSDNMGYLAKINWVGGETQALLLLGERLKV

EQHAFERGYYLPNQAMPNILDTPKSMSAHLRFGCLSVRRFYWCMHDLFKNVQLRACVRGVQMSGGAHITGQLIWREYFYT

MSVNNPQYDRMEGNEICLSIPWAKPDAEQLQRWRLGQTGFPLIDSAMRQLLAEGWLHHTLRNTVATFLTRGGLWQNWEFG

LQHFLKYLLDADWSVCAGNWMWVSSSAFERLLDSSLVTCPVALAKRLDPNGQYIKQYVPELQHVPKEYIHEPWLMSAEEQ

KRYECLIGVHYPDRIIDLSLASKRNMLAMKALRNSLIEPPPHCRPSNEEEVRQFFWLAD

>Drosophila_moj_CRY-2_XP_002004062.1 XP_002004062.1

MAVERRTLIHWFRKGLRVHDNPALIQIFNKAGASPDKFSVRPIFVLDPGILDWMKVGANRWRFLQQSLLDLDKNLKELNS

RLYIVRGKPVDIFPNLFDRWNVELLTFETDIEPYAVKRDKAVQDIAAAHGVKVDTHCSHTIYNPEIVIAKNFGRAPITYQ

KFLSVVEKLKLPKVLDKPQRLPNGVQPIADELEMGQSDVYECPTLDQLVKRPQELGINKFPGGEREALRRLDTSLSDEHW

VASFEKPNTAPNSLEPSTTVLSPYLKFGCLSARLVHQRLHEILKRHPKHSKPPVSLVGQLLWREFYYTAAAVEPNFDRML

GNVYCLQIPWQERPDHLDAWAHGRTGYPFIDAIMRQLRQEGWIHHLARHAVACFLTRGDLWISWEEGQRVFEELLLDHDW

ALNAGNWMWLSASAFFHQYFRVYSPVAFGKKTDRTGAYIRKYVPELAKYPAGCIYEPWKATLADQREFGCVLGIDYPHRI

VNHDIVHKENIKRMSAAYKINREVRTGKQEDEIDYDSSMSLTTRQRNASFET

>Drosophila_moj_PL_XP_002004196.2 XP_002004196.2

MQIGGSVSTKFALLGGCRVLFQLLHMKRNKSNAARAAKKKPKKAESSGSSNESESGSGSGEETVTITPEKKTKPSYNSFE

QFLSHVQDQRLKAAANVHEFAFRKKRVRVLSSASDVKENCNGGVVYWMSRDARVQDNWALLFAQRLALKLELPLTVVFCL

VPKFLNATLRHYKFMLGGLQEVEQQCRELNVSFHLLLGPAVERLPQFVKDEKIGAVICDFAPLRLPRQWVEDVVKALPAH

VPLTQVDAHNVVPLWVTSDKQEYAARTIRNKINSKLSEYLTEFPPLIKHAYGNVKGSRSVDWTAAYEMLSCDKSVDAVEW

AKPGYTAACRQLYDFCTRRLRHFNDKRNDPMADALSGLSPWLHFGQISAQRCVLEVGRYKTIYKESVEAYFEEAIVRREL

ADNFCYYNEHYDSLKGLYSWAYESLQAHRKDKRSPCYSLEELEHSRTYDDLWNSAQLQLVKEGKMHGFLRMYWAKKILEW

TETPEQALEYSILLNDKYSLDGRDPNGYVGCMWSIGGIHDQGWKERAIFGKIRYMNYQGCKRKFDVNAYVMRYGGKVHKK

KES

>Drosophila_nav_CRY-1_XP_030242854.1 XP_030242854.1

MARRGANVIWFRHGLRLHDNPAMLAALSNKDQGVALIPIFIFDGESAGTKNVGYNRMRFLLDSLQDIDRQLQEATEGRGG

LLICEGQPVHIFRRLHEHVGLHKICVEQDCEPIWNERDEATKSLCRELGIEYVEKVSHTLWDPRTVIDTNGGIAPLTYQM

FLHTVQIIGLPPRPVRDPHFGGVKFVQLSPELRHDIGCFEMTPTPEHFNVYSDNMGYLAKINWVGGETQALLLLGERLKV

EQHAFERGYYLPNQAMPNILDTPKSMSAHLRFGCLSVRRFYWCMHDLFKNVQLRACVRGVQMSGGAHITGQLIWREYFYT

MSVNNPQYDRMEGNEICLSIPWAKPDAEQLQRWRLGQTGFPLIDSAMRQLLAEGWLHHTLRNTVATFLTRGGLWQNWEFG

LQHFLKYLLDADWSVCAGNWMWVSSSAFERLLDSSLVTCPVALAKRLDPNGQYIKQYVPELQHVPKEYIHEPWLMSAEEQ

KRYECLIGVHYPDRIIDLSLASKRNMLAMKALRNSLIEPPPHCRPSNEEEVRQFFWLAD

>Drosophila_nav_CRY-2_XP_017954545.1 XP_017954545.1

MAVERRTLIHWFRKGLRVHDNPALTQIFNKAEASPDKFTVRPIFVLDPGILDWMQVGANRWRFLQQTLLDLDKSLKELNS

RLYIVRGKPVDIFPSLFDRWNVELLTFETDIEPYAVKRDKAVQDIAVAHGVKVDTHCSHTIYNPEIVIAKNLGKAPITYQ

KFLSVVETLKLPKVLDKPQRLPNGVQPIADALELEQSDVYECPTLDQLVKRPQELGINKFPGGETEALRRLDTSLSDEHW

VASFEKPNTSPNSLEPSTTVLSPYLKFGCLSARLVHQRLQEILKRHPKHSKPPVSLMGQLLWREFYYTAAAVEPNFDRML

GNAYCLQIPWQERPDHLDAWAHGRTGYPFIDAIMRQLRQEGWIHHLARHAVACFLTRGDLWISWEEGQRVFEQLLLDQDW

ALNAGNWMWLSASAFFHQYFRVYSPVAFGKKTDRTGAYVRKYVPELAKYPAGCIYEPWKATLADQREFGCVLGIDYPHRI

VNHDIVHKENIKRMSAAYKINRQVRTGKQEDEIDYDTMSLTNGKRNARFETERSTAKRKC

>Drosophila_nav_PL_XP_030238967.1 XP_030238967.1

MQIGGSVRTKFALLGGCRILFQLLHMKRNKSNAARAAKKKPKKTESSGSSNESQSGSGSGEETVTITPEKKTKPSYNSFE

QFLSHVQDQRLKAAVNVHEFAFRKKRVRVLSSASDVKENCNGGVVYWMSRDARVQDNWALLFAQRLALKLELPLTVVFCL

VPKFLNATLRHYKFLLGGLQEVEQQCRELNISFHLLLGPAAERLPQFVKDQKIGAVICDFSPLRLPRQWVDDVVKALPVH

VPLTQVDAHNVVPLWVTSDKQEYAARTIRNKINSKLNEYLTEFPPLIKHAYGNANGSRSVDWTAAYEMLSCDKSVDAVEW

AKPGYTSACRQLYDFCTLRLRHFNNKRNDPMADALSGLSPWLHFGQISAQRCVLEVGRYKTIYKESVEAYCEEAIVRREL

ADNFCYYNEHYDSLKGLYSWAYESLQAHRKDKRSPCYSLEELEHSRTYDDLWNSAQLQLVKEGKMHGFLRMYWAKKILEW

TETPEQALEYSILLNDKYSLDGRDPNGYVGCMWSIGGIHDQGWKERAIFGKIRYMNYQGCKRKFDVNAYVMRYGGKVHKK

KEG

>Drosophila_nov_CRY-1_XP_030565865.1 XP_030565865.1

MALRGANVIWFRHGLRLHDNPALLAALSDKDQGIALIPIFIFDGESAGTKSVGYNRMRFLLDSLQDIDMQLKKATKGRGG

LLMCQGQPTQIFRRLHERVRLHKICVEQDCEPIWNERDEATKSLCHELSIEYVEKVSHTLWDPRTVIDTNGGIAPLTYQM

FLHTVQIIGLPPRPIPDPNFEVVSIAQLPPELRKDIGCFEQTPTPEDFNIYSDNMGYLAKMNWIGGETQALSLLGERLKV

EQHAFERGYYLPNQALPNILETPKSMSAHLRFGCLSVRRFYWSMHDLFKNVQLRACVRGVQMTGGAHITGQLIWREYFYT

MSVNNPQYDRMEGNEICLTIPWSKPDADQLQRWRLGQTGFPLIDSAMRQLLAEGWLHHTLRNTVATFLTRGGLWQNWELG

LEHFLKYLLDADWSVCAGNWMWVSSSAFERLLDSSLVTCPVALAKRLDPNGAYIKQYVPELKNVPKEFIHEPWRMSSEQQ

ERYECLLGFHYPQRIIDLPLASKRNTLAMKALRNSLIEPPPHCRPSNEEEVRQFFWLTD

>Drosophila_nov_CRY-2_XP_030561576.1 XP_030561576.1

MSAKRRTLVHWFRKGLRVHDNPALSQIFNEARASPEKFYIRPIFILDPGILDWMQVGANRWRFLQQSLVDLDKKLKELNS

RLYIVRGKPVDVFPELFERWNVQLLTFESDIEPYALQRDTTVQKIASEHGVKVDTYCSHTIYNPELVIAKNLGKAPVTYQ

KFLGIVDKLKLPKALAVPESLPDGMKPIADEFEEIDSCVYDCPTLDQLVKRPQELGVNKFLGGETEALRRMEASLIDENW

VAAFEKPNTAPNSLEPSTTVLSPYLKFGCLSARLLHHRLKEILVRKPKHSKPPVSLVGQLLWREFYYTVAAADPNFDRML

GNAYCLQIPWQKQPDHLEAWTHGRTGYPFIDAIMRQLRQEGWIHHLARHAVACFLTRGDLWISWEDGQRVFEQLLLDQDW

ALNAGNWMWLSASAFFHQYFRVYSPVAFGKKTDPTGAYIRKYVPELAKYPAGCIYEPWKATLSAQREYGCVLGVDYPHRI

VNHDIVHKENIKRMSAAYKVNREVRTGKQEDQNDYEPSTSQSTGKRKASAEAKKTVAKRRR

>Drosophila_nov_PL-lik_XP_030560010.1 XP_030560010.1

MQIVKYKFVLLGGIRALSQILHMKRNKNSSAAGGAKKKPKKNESSGSDEETSVTIATEKKTKPSYNSFEQFLSHMQAQRV

ATARDVHDFAFKKKRVRVLSTVSDVKEQSKGGVVYWMSRDARVQDNWALLFAQRLALKLELPLTVVFCLVPKFLNATLRH

YKFMLGGLQEVEQQCRELNISFQLLLGPAAERLPEFVTAEDIGAVICDFAPLRLPRQWVSDVVKALPGHVPLTQVDAHNI

VPLWVTSEKQEYAARTIRNKINSKLGEFLSEFPPVIRHPHGSGQGKESTDWTAAHALLTCDKTVDAVDWAKPGYTAGCRQ

LYEFCTRRLRHFNEKRNDPTADALSGLSPWLHFGQISAQRCILEVQRYSAMHKASADAFCEEAIVRRELADNFCYYNEHY

DSLKGLHDWAYQTLQAHRKDKRSPCYTLEELEQARTYDDLWNSAQLQLVKEGKMHGFLRMYWAKKILEWTETPELALEYS

ILLNDKYSLDGRDPNGYVGCMWSIGGVHDQGWKERDIFGKIRYMNYQGCKRKFDINAFVMRYGGKVHK

>Drosophila_obs_CRY-1_XP_022216182.1 XP_022216182.1

MATRGANVLWFRHGLRLHDNPALLAALQEKDQGIALIPVFIFDGESAGTKSVGYNRMRFLLDSLQDIDEQLQAATEGRGR

LFVFEGEPTLIFRRLHEQVRLHKICAEVDCEPIWNERDESARLLCRELGIEYVEKVSHTLWDPRLVIETNGGIPPLTYQM

FLHTVQIIGVPPRPATDAHIDDATFIQLAPELRRHLGCFDKIPSPEHFNIYSDNMGFLAKINWRGGETQALALLEERLKV

ERNAFERGYYLPNQAMPNIQEAPKSMSAHLRFGCLSVRHFYWSVHDLFENVQLAACVRGVQLTGGAHITGQLIWREYFYT

MSVNNPNYDRMDGNEICLTIPWAKRDEDLLQRWRLGQTGFPLIDGAMRQLLAEGWLHHTLRNTVATFLTRGGLWQSWEHG

LQHFLKYLLDADWSVCAGNWMWVSSSAFERLLDSSLVTCPVALAKRLDPEGVYIRRYVPELKNLPREYIHEPWRLSAEQQ

VQYECLIGVHYPERVIDLSKAVKRNMTAMTALRNSLITPPPHCRPSNEEEVRQFFWLANY

>Drosophila_obs_CRY-2_XP_022214732.2 XP_022214732.2

MMDAKHATLVHWFRKGLRVHDNPALTQIFAAANAAPENIHVRPIFILDPGILDWMQVGANRWRFLQQTLHDLDQQLRQLN

SRLFVVRGKPVDVFPRIFKSWRVELLTFETDIEPYALQRDAAVQKLAKDAGIKVDTHCSHTIYNPELVIAKNMGKAPITY

QKFLSVVDQLKAPKVLQLPGKLSKKALPPKDEVEQQDGAAYDCPTLQQLVKKPEDLGPNKFPGGETEGLRRMKESLQDEL

WVARFEKPNTAPNSLEPSTTVLSPYLKFGCLSARLFHQQLKEILKRQSKHSQPPVSLIGQLLWREFYYTVAAAEPNFDRM

LGNVYCLQIPWQEQADHLEAWTHARTGYPFIDAIMRQLRQEGWIHHLARHAVACFLTRGDLWISWEEGQRVFEQLLLDQD

WALNAGNWMWLSASAFFHQYFRVYSPVAFGKKTDPQGHYIRKYVPELAKYPNGCIYEPWKATLADQRAYGCVLGVDYPHR

IVKHELVHKENIKRMSAAYKVNREVRTGKEEDSPEADRSATGKRKVAKTTANAAKRKR

>Drosophila_obs_PL_XP_022228882.2 XP_022228882.2

MFRPIGAQISIVNCCYIVGLHFVRQLAPIYTMKRAKTGTAGASKKAPKKENKSVEAEEASSSGDEALASSSKLAQTKPEY

KNFEQFLAHLEQQRSEAAANVQEFSFKKKRVRVLSKVNDVGEKCTGGVVYWMSRDGRVQDNWALLFAQRLALKLELPLAV

VFCLVPKFLNATIRHYKFMMGGLQEVEQQCRELDIPFHLLLGPAVDRLPEFVRSKNVGAVVCDFAPLRVPRKWVEDVAKA

LPKNVPLTQVDAHNVVPLWVASDKQEYAARTIRNKINSKLGEFLSDFPPVIKHPHGEGCKKVKPIDWPAAYAMLECDMDV

DEVQWAKPGYKAACLQLYEFCSRRLGKFNDKRNDPTVDALSGLSPWLHFGQISAQRCALEVQRYRGQHKASADAFCEEAI

VRRELADNFCYYNENYDSLKGLTPWAYQSLEAHRKDKRDPCYTLEELEQSLTYDDLWNSAQLQLVREGKMHGFLRMYWAK

KILEWTATPEQALEYSILLNDKYSLDGRDPNGYVGCMWSIGGIHDMGWKERAIFGKIRYMNYQGCKRKFDVNAFVMRYGG

KVHKKKG

>Drosophila_pan_hypoth_KAH8325690.1 KAH8325690.1

MDAKHATLVHWFRKGLRVHDNPALSQIFKVANTAPEKYFVRPIFILDPGILDWMQVGANRWRFLQQTLHDLDQQLQKLGS

RLFVVRGKPAEVFPRIFKRWRVELLTFETDIEPYSLARDAAVQKLAKSNGVKVETHCSHTIFNPELVIAKNLGKAPITYQ

KFLGIVEKLKLPTVLDLPEKLKEEVQPPKDDIEEKDSEAYDCPTMEQLVKRPEDLGPLKFPGGETEALRRMEESLKDELW

VARFEKPNTAPNSLEPSTTVLSPYLKFGCLSSRLFHQRLKEILKRQTKHSQPPVSLIGQMMWREFYYTVAAAEPNFDRML

GNVYCLQIPWEEHPDHLKAWTYGQTGYPFIDAIMRQLRQEGWIHHLARHAVACFLTRGDLWISWEEGQRVFEQLLLDQDW

ALNAGNWMWLSASAFFHQYFRVYSPVAFGKKTDPQGNYIRKYVPELAKYPAGCIYEPWKASLSDQRAYGCVLGTDYPHRI

VKHEVVHKENIKRMGAAYKVNREVRTGKQEDSFEEKPETSSTGKRKLGKTSGNSAKRKR

>Drosophila_pan_hypoth_KAH8326574.1 KAH8326574.1

MATRGANVIWFRHGLRLHDNPALLAALAEKEQGIALLPVFIFDGESAGTKNVGYNRMRFLLDSLQDIDDQIQTLTEGRGR

LLVFEGKPEHIFRRLHEQLRLHKICLEQDCEPIWNHRDETIRSLCHELGIEFVEKVSHTLWNPQSVIETNGGIPPLTYQM

FLHTVQILGLPPRPVNDARLEDASFVQMDPELLRNLGYLEQIPTPEHFNVYGDNMGFLSKIRWRGGERQALLLLDERLKV

EQHAFEKGYYMPNQALPNIQETPKSMSPHLRFGCLSVRRFYWSVHDLFKNVQLRACVRGVQMTGGAHITGQLIWREYFYT

MSVNNPNYDRMDGNEICLSIPWAKRDETQLQKWRLGQTGFPLIDAAMRQLLAEGWLHHVLRNTVATFLTRGGLWQSWEHG

VQHFLRYLLDADWSVCAGNWMWVSSSAFERLLDSSLVTCPVALAKRLDPDGAYIRQYVPELKNVPREFIHEPWRMSLQQQ

ELYECLIGVHYPDRLIDLSKAVKGNMLAMKTLRDSLITPPPHCRPSNEEEVRTFFWLADLTI

>Drosophila_pan_hypoth_KAH8340081.1 KAH8340081.1

MKRTKTAKAGPAKKTAKKETPKSEESDQESSGSSQVEASSSKSVLSKPEYKNLEQFLTHCQDQRTSTAANIKEFNFLKKR

VRVLSKNGDVGDSCEGGVIYWMSRDGRVQDNWALLFAQRLALKLELPLAVVFCLVPKFLNATIRHYKFMMGGLQEVEQQC

RELDIPFHLLLGPAVDRIPEFVKSRKVGAVICDFAPLRVPRKWVDDVGKALPKTVPLVQVDAHNVVPVWVASDKQEYAAR

TIRNKINSKLSEYLTEFPPVVKHPHGTGCQKVDPVDWTAAYEMLECDKSVDEVDWAKPGYKAACKQLYEFCSRRLRQFND

KRNDPLADAISGLSPWLHFGQISAQRCALEVQRFRGQHKASAEAFCEEAIVRRELADNFCYYNEHYDSLKGLSSWAYQTL

DAHRKDKRDPCYSLEEMEKALTYDDLWNSAQLQLVREGKMHGFLRMYWAKKILEWTETPEQALEYAILLNDKYSLDGRDP

NGYVGCMWSIGGVHDMGWKERSIFGKIRYMNYQGCRRKFDVNAFVIRYGGKVHKKKKE

>Drosophila_per_CRY1_XP_002019874.1 XP_002019874.1

MVPRGANVLWFRHGLRLHDNPALLAALEEKDQGIPLIPVFIFDGESAGTKSVGYNRMRFLLDSLQDLDEQLQSATEGRGR

LFVFEGEPTLIFRRLHEQVRLHKICAELDCEPIWNERDESARLLCRELGIEYVEKVSHTLWDPRLVIETNGGIPPLTYQM

FLHTVQIIGVPPRPAIDAHINDATFIQLAPELRQHLGCFDQVPNPEHFNIYSDNMGFLAKINWRGGETQALALLEERLKV

ERNAFERGYYLPNQANPNIQEAPKSMSAHLRFGCLSVRRFYWSVHDLFENVQLAACVRGVQIEGGAHITGQLIWREYFYT

MSVNNPNYDRMEGNEICLTIPWAKPDENLLQRWRLGQTGFPLIDGAMRQLLAEGWLHHTLRNTVATFLTRGGLWQSWEPG

LKHFLKYLLDADWSVCAGNWMWVSSSAFERLLDSSLVTCPVALAKRLDPEGVYIRRYVPELKNLPKEYIHEPWRLSAEQQ

VKFECLIGVHYPERIIDLSKAVKRNMMAMTALRNSLITPPPHCRPSNEEEVRQFFWLANY

>Drosophila_per_CRY-2_XP_002015258.2 XP_002015258.2

MDATHATLVHWFRKGLRVHDNPALTQIFSAANAAPEKFHVRPIFILDPGILDWMQVGANRWRFLQQTLHDLDQQLRKLNS

RLFVVRGKPVDVFPRVFKSWRVELLTFETDIEPYALQRDAAVQKLAKAEGIKVDTHCSHTIYNPELVIAKNMGKAPITYQ

KFLSVVDQLKVPKVLELPEQLVKKALPPKDEVEQQDDNAYDCPTLEQLVKRPEELGPNKFPGGETEGLRRMKESLRDELW

VARFEKPNTAPNSLEPSTTVLSPYLKFGCLSARIFHQQLKAILKRQSKHSQPPVSLIGQLLWREFYYTVAAAEPNFDRML

GNVYCLQIPWQEQADHLEAWTHGRTGYPFIDAIMRQLRQEGWIHHLARHAVACFLTRGDLWISWEAGQRVFEQLLLDQDW

ALNAGNWMWLSASAFFHQYFRVYSPVAFGKKTDPQGHYIRKYVPELAKYPNGCIYEPWKATLADQREYGCVLGVDYPHRI

VKHELVHKENIKRMSAAYKVNREARTGKEEDSPEAPGKRKVAKSIGNAAKRKR

>Drosophila_per_PL_XP_002017941.1 XP_002017941.1

MKRAKSGTAGASKKAPKKEKKSAEPAASSSQESSSGGEEGASSSKLVQSKPEYKNFEQFLAHLEQQRSESAANIQEFPFK

KKRVRVLSKVNDVQDKCSGGVVYWMSRDGRVQDNWALLFAQRLALKLEMPLAVVFCLVPKFLNATIRHYKFMMGGLQEVE

EQCRDLGIPFHLLLGPAVDRLPEFVRSKDVGAVVCDFAPLRVPRKWVEDVAKALPKNVPLTQVDAHNVVPLWVASDKQEY

AARTIRNKINSKLGEFLSDFPPVIKHPYGEGCKKVKPIDWPAAYAMLECDMDVDEVKWAKPGYKAACLQLYEFCSRRLGK

FNDKRNDPTVDALSGLSPWLHFGQISAQRCVLEVQRYRSQHKASADAYCEEAIVRRELADNFCYYNENYDSLKGLSPWAY

QSLEAHRKDKRDPCYTLEELEQSLTYDDLWNSAQLQLVREGKMHGFLRMYWAKKILEWTATPEQALEYAILLNDKYSLDG

RDPNGYVGCMWSIGGIHDMGWKERAIFGKIRYMNYQGCKRKFDVNAFVMRYGGKVHKKKG

>Drosophila_pse.ana_hypoth_KAH8317692.1 KAH8317692.1

MDAKHATLVHWFRKGLRVHDNPALSQIFKVANGTPGKYFVRPIFILDPGILDWMQVGANRWRFLQQTLYDLDQQLQKLGS

RLFVVRGKPAEVFPRIFKSWRVELLTFESDIEPYSLARDSAVQKLAKSEGVKVETHCSHTIFNPELVIARNLGKAPITYQ

KFLGIVEKLKLPTVLDLPEKLKEEGQPPKDDIEEKDSEAYDCPTMEQLVKRPEELGPNKFPGGETEALRRMEESLKDELW

VARFEKPNTAPNSLEPSTTVLSPYLKFGCLSSRLFHQRLKEILKRQTKHSQPPVSLIGQMMWREFYYTVAAAEPNFDRML

GNVYCLQIPWEEHPDHLKAWTYGQTGYPFIDAIMRQLRQEGWIHHLARHAVACFLTRGDLWISWEEGQRVFEQLLLDQDW

ALNAGNWMWLSASAFFHQYFRVYSPVAFGKKTDPQGNYIRKYVPELAKYPSGCIYEPWKASLADQRAYGCVLGTDYPHRI

VKHEVVHKENIKRMGAAYKVNREVRTGKQEDSFEEKPETSSTGKRKLGKTTGNSAKRKR

>Drosophila_pse.ana_hypoth_KAH8319913.1 KAH8319913.1

RVSETDFYFVPYVQLLQQTIMKRTKTTKAGPAKKAAKKETLKSEDSDQESGGSSQVEESSSSKTVLLKPDYKNLEEFLAH

CEHQRSTTAASIKEFNFLKKRVRVLSKNGDVGDSCEGGVVYWMSRDGRVQDNWALLFAQRLALKLELPLAVVFCLVPKFL

NATIRHYKFMMGGLQEVEQQCRELDIPFHLLLGPAVDRLPQFVKSRKVGAVICDFAPLRVPRKWVEDVGKALPKTVPLVQ

VDAHNVVPVWVASDKQEYAARTIRNKINSKLSEYLTEFPPVIKHPHGPGCRKVDPVDWTAAYEKLECDKSVDEVDWAKSG

YKAACKQLYEFCSRRLRIFNDKRNDPIADALSGLSPWLHFGQISAQRCALEVQRFRGQHKASAEAFCEEAIVRRELADNF

CYYNEHYDSLKGLSSWAYETLNAHRKDKRDPCYSLDEMEKALTYDDLWNSAQLQLVREGKMHGFLRMYWAKKILEWSETP

EQALEYAILLNDKYSLDGRDPNGYVGCMWSIGGVHDMGWKERSIFGKIRYMNYQGCRRKFDVNAFVIRYGGKVHKKK

>Drosophila_pse.ana_hypoth_KAH8325003.1 KAH8325003.1

MATRGANVIWFRHGLRLHDNPALLAALAGKDQGIALLPIFIFDGESAGTKNVGFNRMRFLLDSLQDIDDQIQTLTEGRGR

LLVFQGEPKDIFRRLHEQVHLHKICFEQDCEPIWNQRDETIRSLCEELGIQCVEKVSHTLWDPRTVIETNGGIPPLTYQM

FLHTVQIIGLPPRPTSNARLDNATFVQLEPEILRSIGYLEQIPTPEHFNVYGDNMGFLSKIRWRGGEREALLLLEERLKV

EQQAFEKGFYLPNQAMPNIQETPKSMSPHLRFGCLSVRRFYWSVHDLFQNVQLRACVRGIQMTGGAHITGQLIWREYFYT

MSVNNPNYDRMEGNEICLSIPWAKPNEAQLQKWRLGQTGFPLIDAAMRQLLAEGWLHHVLRNTVATFLTRGGLWQSWEHG

VQHFLRYLLDADWSVCAGNWMWVSSSAFERLLDSSLVTCPVALAKRLDPEGAYIRQYVPELKNVPRELIHEPWRMSLQQQ

EQYECLIGVHYPDRLIDLTKAVKGNMLAMKSLRDSLITPPPHCRPSNEEEVRTFFWLADLTI

>Drosophila_pse.obs_CRY-1_XP_001360014.2 XP_001360014.2

MVPRGANVLWFRHGLRLHDNPALLAALEEKDQGIPLIPVFIFDGESAGTKSVGYNRMRFLLDSLQDLDEQLQSATEGRGR

LFVFEGEPTLIFRRLHEQVRLHKICAELDCEPIWNERDESARLLCRELGIEYVEKVSHTLWDPRLVIETNGGIPPLTYQM

FLHTVQIIGVPPRPAIDAHINDATFIQLAPELRQHLGCFDQVPNPEHFNIYSDNMGFLAKINWRGGETQALALLEERLKV

ERNAFERGYYLPNQANPNIQEAPKSMSAHLRFGCLSVRRFYWSVHDLFENVQLAACVRGVQIEGGAHITGQLIWREYFYT

MSVNNPNYDRMEGNEICLTIPWAKPDENLLQRWRLGQTGFPLIDGAMRQLLAEGWLHHTLRNTVATFLTRGGLWQSWEPG

LKHFLKYLLDADWSVCAGNWMWVSSSAFERLLDSSLVTCPVALAKRLDPEGVYIRRYVPELKNLPKEYIHEPWRLSAEQQ

VKFECLIGVHYPERIIDLSKAVKRNMMAMTALRNSLITPPPHCRPSNEEEVRQFFWLANY

>Drosophila_pse.obs_CRY-2_XP_015036266.2 XP_015036266.2

MDATHATLVHWFRKGLRVHDNPALTQIFSAANAAPEKFHVRPIFILDPGILDWMQVGANRWRFLQQTLHDLDQQLRKLNS

RLFVVRGKPVDVFPRVFKSWRVELLTFETDIEPYALQRDAAVQKLAKAEGIKVDTHCSHTIYNPELVIAKNIGKAPITYQ

KFLSVVDQLKVPKVLELPEKLAKKALPPKDEVEQQDDNAYDCPTLEQLVKRPEDLGPNKFPGGETEGLRRMKESLRDELW

VARFEKPNTAPNSLEPSTTVLSPYLKFGCLSARLFHQQLKAILKRQSKHSQPPVSLIGQLLWREFYYTVAAAEPNFDRML

GNVYCLQIPWQEQADHLEAWTHGRTGYPFIDAIMRQLRQEGWIHHLARHAVACFLTRGDLWISWEEGQRVFEQLLLDQDW

ALNAGNWMWLSASAFFHQYFRVYSPVAFGKKTDPQGHYIRKYVPELAKYPNGCIYEPWKATLADQREYGCVLGVDYPHRI

VKHELVHKENIKRMSAAYKVNREARTGKEEDSPEAPGKRKVAKTIGNAAKRKR

>Drosophila_pse.obs_PL_XP_001361001.4 XP_001361001.4

MFIPTYWRPNFNNINCSSIVGLLVNNFVRQLAPIYTMKRAKSGTAGASKKAPKKEKKSAEPAASSSQESSSSGEEGASSS

KLVQSKPEYKNFEQFLAHLEQQRSESAANIQEFPFKKKRVRVLSKVNDVQDKCSGGVVYWMSRDGRVQDNWALLFAQRLA

LKLEMPLAVVFCLVPKFLNATIRHYKFMMGGLQEVEEQCRELGIPFHLLLGPAVDRLPEFVRSKDVGAVVCDFAPLRVPR

KWVEDVAKALPKNVPLTQVDAHNVVPLWVASDKQEYAARTIRNKINSKLGEFLSDFPPVIKHPYGEGCKKVKPIDWPAAY

AMLECDMDVDEVKWAKPGYKAACLQLYEFCSRRLGKFNDKRNDPTVDALSGLSPWLHFGQISAQRCVLEVQRYRSQHKAS

ADAYCEEAIVRRELADNFCYYNENYDSLKGLSPWAYQSLEAHRKDKRDPCYTLEELEQSLTYDDLWNSTQLQLVREGKMH

GFLRMYWAKKILEWTATPEQALEYAILLNDKYSLDGRDPNGYVGCMWSIGGIHDMGWKERAIFGKIRYMNYQGCKRKFDV

NAFVMRYGGKVHKKKG

>Drosophila_pse.tak_hypoth_KAH8344410.1 KAH8344410.1

MTTRGANVIWFRHGLRLHDNPALLAALADKDQGIALIPIFIFDGESAGTKCVGYNRMRFLLDSLQDIDDQLQAATKGRGR

LHVFEGEPAYIFRRLHEQVRLHRICMEQDCEPIWNERDETIRSLCRDLGIDLVEKVSHTLWDPRTVIDTNGGIPPLTYQM

FLHTVQIIGLPPRPAADARLDDVSFVDLAPELRRSLGYFEQLPTPEHFNVYGDNMGFLAKINWRGGETQALLLLAERLKV

EQHAFERGFYLPNQALPNIHDTPKSMSAHLRFGCLSVRRFYWSVHDLFKNVQLRACVRGVQMTGGAHITGQLIWREYFYT

MSVNNPNYDRMEGNEICLNIPWAKPNEDLLQRWRLGQTGFPLIDGAMRQLLAEGWLHHTLRNTVATFLTRGGMWQSWEHG

LQHFLKYLLDADWSVCAGNWMWVSSSAFERLLDSSLVTCPVALAKRLDPEGAYIKQYVPELMNVPKEFVHEPWRMTAEQQ

EQYECLIGVHYPERIIDLSTDVKRNMLAMKALRNSLMTPPPHCRPSNEEEVRQFFWLADVAV

>Drosophila_pse.tak_hypoth_KAH8351983.1 KAH8351983.1

MDKDRSTLVHWFRKGLRVHDNPALSQIFTAANAKPGKFCIRPIFILDPGILDWMQVGANRWRFLQQTLDDLDKQLRKLDS

RLFVVRGKPAEVFPRIFKSWRVELLTFETDIEPYSLSRDAAVQKLAKSDGVKVETHCSHTIYNPELVIAKNLGKAPITYQ

KFLGIVDQLKVPKVLGVPEKLKIKIDPPKDEVELKDSAAYDCPTMEQLVKRPEELGPNKFPGGETEALRRMEESLKDELW

VARFEKPNTAPNSLEPSTTVLSPYLKFGCLSARLFHQKLKEILKRQTKHSQPPVSLIGQLMWREFYYTVAAAEPNFDRML

GNIYCLQIPWQDQPDHLEAWTHGRTGYPFIDAIMRQLRQEGWIHHLARHAVACFLTRGDLWISWEEGQRVFEQLLLDQDW

ALNAGNWMWLSASAFFHQYFRVYSPVAFGKKTDPQGHYIRKYVPELSKYPAGCIYEPWKASLADQRAYGCVLGTDYPHRI

VKHEVVHKENIKRMGAAYKVNREVRTGKEEQSSFEENTETSTSGKRKVRKAVGNAPKRKRKI

>Drosophila_pse.tak_hypoth_KAH8367245.1 KAH8367245.1

MFTLAPYWRQSFKIALSLHTMKRTKATKAGPSKKSAKKQEDEKSEEDSSDVEPSTSKASLASKPDYQNFEQFLTHLEHQR

SCTAPSIQEFSFRKKRVRVLSKTEDVKESSHGGVVYWMSRDGRVQDNWALLFAQRLALKLELPLSVVFCLVPKFLNATIR

HYKFMMGGLQEVEQQCRSLAIPFHLLLGPAVEKLPEFVKSRNIGAVVCDFAPLRLPRQWLEDVGKALPKSVPLVQVDAHN

VVPLWVASDKQEYAARTIRNKINSKLGEFLSEFPPVVQHPHGMGCKEAKPVDWPAAYAMLQCDMDVDEVQWAKPGYRAAC

QQLYEFCTRRLRKFNDKRNDPTADALSGLSPWLHFGQISAQRCALEVQRFRGQHKASADAFCEEAIVRRELADNFCFYNE

HYDSLKGLSSWAYQTLDAHRKDKRDPCYSLEELEMSLTYDDLWNSAQLQLVREGKMHGFLRMYWAKKILEWTATPEQALE

YAILLNDKYSLDGRDPNGYVGCMWSIGGVHDMGWKERAIFGKVRYMNYQGCRRKFDVNAFVMRYGGKVQNKK

>Drosophila_rho_CRY-1_XP_016974807.1 XP_016974807.1

MATRGANVMWFRHGLRLHDNPALLAALADKDQGIALIPVFIFDGESAGTKNVGYNRFRFLLDSLQNIDEQLQAATEGRGR

LHVFEGEPAHIFRRLNEQVRLHRICMEQDCEPIWNDRDETVRSLCRELNIDFVEKVSHTLWDPRSVIDTNGGIPPLTYQM

FLHTVQIIGLPPRPAADARLDDASFVELAPELRRSLGYFEKLPTPEHFNVYGDNMGFLAKINWRGGESQALLLLEERLKV

EQHAFERGFYLPNQALPNILDTPKSMSAHLRFGCLSVRRFYWCVHDLFKNVQLRACVRGVQMTGGAHITGQLIWREYFYT

MSVNNPNYDRMEGNEICLSIPWAKPNEDLLQRWRLGQTGFPLIDGAMRQLLAEGWLHHTLRNTVATFLTRGGMWQSWEHG

LQHFLKYLLDADWSVCAGNWMWVSSSAFERLLDSSLVTCPVALAKRLDPEGAYIKQYVPELMNVPKEFVHEPWRMSAEQQ

EQYECLIGVHYPERIIDLSMAVKRNMLAMKALRNSLMTPPPHCRPSNEEEVRQFFWLADVAV

>Drosophila_rho_CRY-2_XP_016976032.1 XP_016976032.1

MCAERSTLVHWFRKGLRVHDNPALSQVFTAANAAPGQFFIRPIFILDPGILDWMQVGANRWRFLQQTLNDLDQELRKLDS

RLFVVRGKPAEVFPRIFKSWRVELLTFETDIEPYSLARDEAVQKLAKSEGVKVETHCSHTIYNPELVIAKNLGKAPITYQ

KFLGIVEQLKIPKVLGVPEKLKRINDPPKDEVEQEDSTAYDCPTMEQLVKRPEELGLNKFPGGETEALRRMKESLSDELW

VARFEKPNTAPNSLEPSTTVLSPYLKFGCLSARLFHQKLKEILKRQTKHSQPPVSLIGQLLWREFYYTVAAAEPNFDRML

GNVYCLQIPWQEQPKHLEAWTHGRTGYPFIDAIMRQLRQEGWIHHLARHAVACFLTRGDLWISWEEGQRVFEQLLLDQDW

ALNAGNWMWLSASAFFHQYFRVYSPVAFGKKTDPQGHYIRKYVPELSKYPAGCIYEPWKASLADQRAYGCVLGTDYPHRI

VKHEVVHKENIKRMGAAYKVNREVRTGKAEDSLEEKPETSTTGKRKALKASGNAPKRKR

>Drosophila_rho_PL-lik_XP_016990538.2 XP_016990538.2

MLTLGPYWRECFKIILLHHTMKRAKTTKAGPPKKNSKKQTDSPKEDPKSGHESTDEEASPSKASFVSKPDYQNFEQFLTH

LEVQRADTAASIQEFSFRKKRVRVLSKIEDVEESSQGGVVYWMSRDGRVQDNWALLFAQRLALKLELPLAVVFCLVPKFL

NATIRHYKFMMGGLQEVEQQCRTLDIPFHLLMGPAVERLPEFVKSNDIGAVVCDFAPLRLPRQWVDDVGKALPKNVPLVQ

VDAHNVVPLWVTSDKQEYAARTIRNKINSKLGEFLSEFPPVVRHPHRSRCKEAKIVDWPAAFAKLECDMDVDEVQWAQPG

YKAACQQLYDFCSRRLRNFNDKRNDPTADVLSGLSPWLHFGQISAQRCALEVQRFRGQHKASADAFCEEAIVRRELADNF

CYYNEHYDNLKGLSSWAYQTLDAHRKDIRDPCYGLEELEKSLTYDDLWNSAQLQLVREGKMHGFLRMYWAKKILEWTATP

EQALEYAILLNDKYSLDGRDPNGYVGCMWSIGGVHDMGWKERAIFGKVRYMNYQGCRRKFDVNAFVMRYGGKVHKKK

>Drosophila_rub_hypoth_KAH8359072.1 KAH8359072.1

MALRSANVMWFRHGLRLHDNPALLSALADKDHGVALIPMFIFDGESAGTKSVGFNRLRFLLDSLQDIDQQLQSETGGRGR

LLIFQGNPVQIFRRLHEQVRLHKICVEQDCEPIWNERDEATKNLCSELGIEYVEKVSHTLWDPRTVIETNGGIAPLTYQM

FLHTVHIIGLPPRPVHDPHFGGVSFVQLSPELRRELGCFEQPPTPEHFNIFSDNMGYLAKINWVGGETQALELLGERLKI

EQHAFERGFYLPNQAMPNILDTPKSMSAHLRFGCLSVRRFYWSVHDLFKNVQLRACVRGLQMTGGAHITGQLIWREYFYT

MSVNNPHYDRMEGNEICLNIPWAKPDDELLQRWRLGQTGFPLIDGAMRQLLAEGWLHHTLRNTVATFLTRGGLWQNWEFG

VQHFLKYLLDADWSVCAGNWMWVSSSAFERLLDSSLVTCPVALAKRLDPEGIYIKQYVPELQNVPREFIHEPWRMSPEQQ

ERYECLIGVHYPERILDLSVAAKRNTLAMNTLRNSLISPPPHCRPSNEEEVRQFFWLAD

>Drosophila_rub_hypoth_KAH8370648.1 KAH8370648.1

KFKFALLFTSSPSLTLYMKRTKKTNTAAPGRKKPRKDESSSEEEKASSPEPEPAAIPIPSFKNVDGFISNMQEKRSAAAA

NVHAFDFRKKRIRMLSAENDVKEPCKGGVVYWMSRDGRVQDNWAFLFAQRLALKVELPLSVVFCLVPKFLNATLRHYKFM

MGGLQEVEQECRKLNVAFHLLLGPAVERLPEFVIAQDIGAVVCDFAPLRLPRQWVTDVGKALPKNVPFAQVDAHNIVPLW

VASEKQEYAARTIRNKINSKLDEFLTEFPPLIKHPHGKASLVKTVDWAAAQQLLTCDMSVDEVEWAKPGYKAACRQLYEF

CTHRLRYFDEKRNDPMSDALSGLSPWLHFGQISAQRCILEVKRYAGKHKASAAAFCEETIVRRELADNFCYYNEHYDSLK

GLNDWAYQSLDAHRKDKRSPCYTLEELEQSRTYDDLWNSAQLQLVNEGKMHGFLRMYWAKKILEWTATPEQALEYSILLN

DKYSLDGRDPNGYVGCMWSIGGVHDQGWKERAIFGKIRYMNYQGCKRKFDVKAFVMRYGGKVYKKD

>Drosophila_rub_hypoth_KAH8378405.1 KAH8378405.1

MDVKGGNVSQRRTLVHWFRKGLRVHDNPALLQVFEVARAAPQNYSVRAIFLLDPGILDWLQVGANRWRFLQQSLCDLDKQ

LRELNSRLYVVRGKPVELFPQLFERWHVELLTYESDIEPYAVQRDAAVQKLAAAHQVKVDTYCSHTIYNPEVVMARNLGK

APITYQKFLGIVEKLKLPKVRGRPEKLPEEVQPSEDSLESTDPHVYDCPTLAQLIKRPEELGVNKFPGGETEALRRLEAS

LRDEQWVASFEKPKTAPNSLEPSTTVLSPYLKFGCLSARLLYQRLQEILARHTKHSKPPVSLVGQLLWREFYYTAAAAEP

NFDRMLGNAYCMQIAWQEQPDHLAAWTHGRTGYPFIDAIMRQLRLEGWIHHLARHAVACFLTRGDLWISWEEGQRVFEQL

LLDQDWALNAGNWLWLSASAFFHQYFRVYSPVAFGKKTDPTGAYIRKYVPELAKYPANCIFEPWKATLSAQREYGCVLGV

DYPQRIVNHDVVHKENIKRMSAAYKVNREVRTGKQEDEEEDDKTSPELTGKRKAKSIKSGVAAKRRR

>Drosophila_san_CRY-1_XP_039493794.1 XP_039493794.1

MATRGANVIWFRHGLRLHDNPALLAALADKDQGIALIPVFIFDGESAGTKNVGYNRMRFLLDSLQDIDDQLQAATDGRGR

LLVFEGEPANIFRRLHEQVRLHRICIEQDCEPIWNDRDESIRSLCRELNIDFVEKVSHTLWDPQLVIETNGGIPPLTYQM

FLHTVQIIGLPPRPTADARLEDATFVELDSEFCRSLNLFEKLPAPDHFNVYTDNMGFLAKINWRGGETQALLLLDERLKV

EQHAFERGFYLPNQALPNIHDSPKSMSAHLRFGCLSVRRFYWSVHDLFKNVQLRACVRGVQMTGGAHITGQLIWREYFYT

MSVNNPNYDRMEGNEICLSIPWAKPKEDLLQRWRLGQTGFPLIDGAMRQLLAEGWLHHTLRNTVATFLTRGGLWQSWEHG

LQHFLKYLLDADWSVCAGNWMWVSSSAFERLLDSSLVTCPVALAKRLDPEGTYIKQYVPELINVPREFVHEPWRMSVEQQ

EQYECLIGVHYPERIIDLSMAVKRNMLAMKTLRNSLITPPPHCRPSNEEEVRQFFWLADVVV

>Drosophila_san_CRY-2_XP_039484386.1 XP_039484386.1

MDAQRSTLVHWFRKGLRVHDNPALSQIFSAANAAPGKYFVRPIFILDPGILDWMQVGANRWRFLQQTLEDLDNQLRKLNS

RLFVVRGKPADVFPRIFKSWRVEILSFETDIEPYSMTRDAAVQKLAKTEGVKVETHCSHTIYNPELVIAKNLGKAPITYQ

KFLGIVEQLKLPKVLGSPEKLTNITSPPKDEVEQEDLAAYDCPTMEQLVKRPEELGPNKFPGGETEALRRMEDSLKDEIW

VARFEKPNTAPNSLEPSTTVLSPYLKFGCLSARLFYQKLTEIIKRQPKHSQPPVSLIGQLMWREFYYTLAAAEPNFDRML

GNVYCMQIPWQEHPDHLEAWTHGRTGYPFIDAIMRQLRQEGWIHHLARHAVACFLTRGDLWISWEEGQRVFEQLLLDQDW

ALNAGNWMWLSASAFFYQYFRVYSPVAFGKKTDPQGHYIRKYVPELSKYPSGCIYEPWKASLADQRAYGCVLGTDYPHRI

VKHEVVHKDNIKRMGAAYKVNREVRTGKQEESSFEEKSETSTSGKRKVRRVAGNAPKRKR

>Drosophila_san_PL-lik_XP_039478912.1 XP_039478912.1

MFTNALYWRESFKIAMKRTKAQKVGPSKKAAKSEEGSSKPKSDQESNDEEASTSKTSLVSKPEYQNFEQFLTHLEHQRVC

TAANIQEFPFRKKRVRVLSKTDDVKKSSLGGVVYWMSRDGRVQDNWALLFAQRLAFKLELPLSVVFCLVPKFLNATIRHY

KFMMGGLQEVEQQCRALDIPFHLLMGPAVEKLPEFVKSKDIGAVICDFAPLRLPRQWVENVSKALPKSVPMVQVDAHNVV

PLWVASDKQEYAARTIRNKINSKLGEFLSEFPPVVRHPFGTGCKNVNTVDWLAAYASLQCDMEVEEVQWAKPGYTAACQQ

LYEFCSRRLRHFNDKRNDPTADALSGLSPWLHFGHISAQRCALEVQRFRGQYKASADSFCEEAIVRRELADNFCFYNEHY

DSLKGLSSWAYQTLDAHRKDKRDPCYRLEELEKSLTYDDLWNSAQLQLVREGKMHGFLRMYWAKKILEWTATPEEALEYA

ILLNDKYSLDGRDPNGYVGCMWSIGGVHDMGWKERAIFGKVRYMNYQGCKRKFDVNAFVMQYGGKVHKKK

>Drosophila_sec_CRY-1__XP_002043337.1 XP_002043337.1

MATRGANVIWFRHGLRLHDNPALLAALADKDQGIAIIPVFIFDGESAGTKNVGYNRMRFLLDSLQDIDDQLQAATDGRGR

LLVFEGEPAYIFRRLHEQLRLHRICIEQDCEPIWNERDESIRSLCRELSIDFVEKVSHTLWDPQLVIETNGGIPPLTYQM

FLHTVQIIGLPPRPTADARLDDATFVELDPEFCRSLKLFDQLPTPEHFNVYGDNMGFLAKINWRGGETQALLLLDERLKV

EQHAFERGFYLPNQALPNIHDSPKSMSAHLRFGCLSVRRFYWSVHDLFKNVQLRACVRGVQMTGGAHITGQLIWREYFYT

MSVNNPNYDRMEGNEICLSIPWAKPNEDLLQRWRLGQTGFPLIDGAMRQLLAEGWLHHTLRNTVATFLTRGGLWQSWEHG

LQHFLKYLLDADWSVCAGNWMWVSSSAFERLLDSSLVTCPVALAKRLDPDGTYIKQYVPELMNVPKEFVHEPWRMSAEQQ

EQYECLIGVHYPERIIDLSMAVKRNMLAMKSLRNSLITPPPHCRPSNEEEVRQFFWLADVVV

>Drosophila_sec_CRY-2_XP_002042448.1 XP_002042448.1

MDAQRSTLVHWFRKGLRVHDNPALSHIFTAANAAPGKYFVRPIFILDPGILDWMQVGANRWRFLQQTLEDLDNQLRKLDS

RLFVVRGKPAEVFPRIFKSWRVEMLTFETDIEPYSLTRDAAVQKLAKAEGVKVETHCSHTIYNPELVIAKNLGKAPITYQ

KFLGIVDQLKVPKVLGVPEKLKKMPTPPKDEVEQKDSAAYDCPTMEQLVKRPEELGPNKFPGGETEALRRMEESLKDEIW

VARFEKPNTAPNSLEPSTTVLSPYLKFGCLSARLFNQKLKEIIKRQPKHSQPPVSLIGQLMWREFYYTVAAAEPNFDRML

GNVYCMQIPWQKHPDHLEAWTHGRTGYPFIDAIMRQLRQEGWIHHLARHAVACFLTRGDLWISWEEGQRVFEQLLLDQDW

ALNAGNWMWLSASAFFHQYFRVYSPVAFGKKTDPQGHYIRKYVPELSKYPAGCIYEPWKASLADQRAYGCVLGTDYPHRI

VKHEVVHKENIKRMGAAYKVNREVRTGKEEESSFEEKSESSTSGKRKVRRAAGSAPKRKR

>Drosophila_sec_PL_X1_XP_002032814.1 XP_002032814.1

MFTLASYWRESFKIVLPLQAMKRTKAQKAGPSKKAAKSEKASSKPKSDQESSDEEASTSKASLVSKPDYQNFEQFLTHLE

HQRVCTAASIQEFSFRKKRVRVLSKTEDVKESSLGGVVYWMSRDGRVQDNWALLFAQRLALKLELPLTVVFCLVPKFLNA

TIRHYKFMMGGLQEVEQQCRALDIPFHLLMGPAVEKLPQFVKSKDIGAVVCDFAPLRLPRQWVEDVGKALPQTVPLVQVD

AHNVVPLWVASDKQEYAARTIRNKINSKLGEYLSEFPPVVQHPHGTGCKNVKAVDWSAAYASLQCDMEVDEVQWAKPGYK

AACQQLYEFCSRRLRHFNDKRNDPTADALSGLSPWLHFGHISAQRCALEVQRFRGQHKASADAFCEEAIVRRELADNFCF

YNEHYDSLKGLSSWAYQTLDAHRKDKRDPCYSLEELEKSLTYDDLWNSAQLQLVREGKMHGFLRMYWAKKILEWTATPEQ

ALEYAILLNDKYSLDGRDPNGYVGCMWSIGGVHDMGWKERAVFGKVRYMNYQGCRRKFDVNAFVMRYGGKVHKKK

>Drosophila_ser_CRY-1_XP_020813525.1 XP_020813525.1

MAVRGANVMWFRHGLRLHDNPALVAALSDKDQGIALIPVFIFDGESAGTKCVGYNRMRFLLDSLQDIDDQIQAATEGRGR

LHVFEGEPVNIFRRLNEHVRLHRICMEQDCEPIWNDRDDSVRSLCHELGIDFVEKVSHTLWDPRMVIDTNGGIPPLTYQM

FLHTVQIIGLPPRPAADVHLEDATFVELAPELRRSVGYFEKMPNPDHFNVYGDNMGFLAKINWRGGETQALLLLEERLEV

EQHAFERGFYLPNQALPNIHDTPKSMSAHLRFGCLSVRRFYWSVHDLFKNVQLRACVRGVQMSGGAHITGQLIWREYFYT

MSVNNPNYDRMDGNEICLSIPWAKPDEDLLQRWRLGQTGFPLIDAAMRQLLAEGWLHHTLRNTVATFLTRGGLWQSWEHG

LQHFLKYLLDADWSVCAGNWMWVSSSAFERLLDSSLVTCPVALAKRLDPEGVYIKQYVPELMGVPKEFVHEPWRMSAEQQ

EQYECLIGVHYPERIIDLSKAVKRNMLAMKALRNSLITPPPHCRPSNEEEVRQFFWLADQTV

>Drosophila_ser_LOW_QU_XP_020812365.1 XP_020812365.1

MDVKKSTLVHWFRKGLRVHDNPALSQIFTAANASPEKFCVRPIFILDPGILDWMQVGANRWRFLQQTLQDLDDQLRKLDS

RLXVVRGKPAEVFPRIFKSWRVELLTFESDIEPYSLARDALIQKLAKTEGVKVVTHCSHTIYNPELVIAKNLGKAPITYQ

KFLAIVDLLKIPKVLEAPEKLKGVAKPPKDDVELQDSAAYDYPTMXQLVKRPEELGPNKFPGGETEALRRMDESLKDELW

VARFEKPNTAPNSLEPSTTVLSPYLKFGCLSARLFYQRLKEILKRQTKHSQPPVSLIGQLMWREFYYTVAAAEPNFDRML

GNVYCLQIPWQXHPDHLEAWTHGRTGYPFIDAIMRQLRQEGWIHHLARHAVACFLTRGDLWISWEEGQRVFEQLLLDQDW

ALNAGNWMWLSASAFFHQYFRVYSPVAFGKKTDPQGDYIRKYVPELAKYPXSSIYEPWKATLADQRAYGCVLGIDYPHRI

VKHEVVHKENIKRMSAAYKVNREVRTGKEEEDSFEEKPEASTSGKRKVRKAAGSAAKRKR

>Drosophila_ser_PL_XP_020818123.1 XP_020818123.1

MHRILSNWRDYFKIISALRTMKRTQAAKAGPSKKVAKKQEKSPKKESNSDQNSSGFSGDEVASTSKASLSKPDYQNLEQF

IDHLEHQRSATAANIQEFPFRKKRVRVLSKSADVEESCQGGVVYWMSRDARVQDNWALLFAQRLALKLELPLTVVFCLVP

KFLNATIRHYKFLMAGLEEVEQQCRSLDIPFHLLLGPAVQRLPEFVKSQDMGAVVCDFAPLRLPRQWVEDVGKALPKSVP

LVQVDAHNVVPLWVTSDKQEYAARTIRNKINSKLGEFLTEFPAVVKHPHGTGCQGVKPVDWPAAYATLQCDTDVGEVDWA

KPGYKAACQQLYEFCSRRLRHFNDKRNDPMADALSGLSPWLHFGHISAQRCALEVQRFRGQHKASADAFCEEAIVRRELA

DNFCYYNEHYDSLKGLTAWAYQTLDAHRKDKRDPCYSLEELEKSLTYDDLWNSAQLQLVREGKMHGFLRMYWAKKILEWS

PTPEQALEYAILLNDKYSLDGRDPNGYVGCMWSIGGVHDMGWKERAIFGKIRYMNYQGCRRKFDVNAFVMRYGGKVHKRK

E

>Drosophila_set_hypoth_KAH8369439.1 KAH8369439.1

MASRGANVIWFRHGLRLHDNPALLSALADKDQGIALMPVFIFDGESAGTKCVGYNRMRFLLDSLQDIDEQLLELTDGRGR

LHVFEGDPEHIFRRLHEQVTLHKICIEQDCEPIWNQRDKNIRNLCRELGIEFVEQVSHTLWDPRTIIDTNGGIPPLTYQM

FLHTVQIIGLPPRPAASARLEGTSFIQLPPDFRRSIGHFEQMPTPEHFNVYSDNMGFLAKINWRGGERQALVLLEDRLKV

EQHAFERGFYLPNQALPNIQDSPKSMSAHLRFGCLSVRRFYWCVHDLFKNVQLRACVRGMQMTGGAHITGQLIWREYFYT

MSVNNPNYDRMEGNEICLSIPWAKPDERVLQKWRLGQTGFPLIDAAMRQLLAEGWLHHVLRNTVATFLTRGGLWQSWEFG

LQHFLKYLLDADWSVCAGNWMWVSSSAFERLLDSSLVTCPVALAKRLDPEGAYIRQYVPELQNVPREFIHEPWRMSTEQQ

EQYECLIGVHYPDRIIDLTRAVKSNMLAMKNLRQSLMTPPPHCRPSNEEEVRNFFWLADLAV

>Drosophila_set_hypoth_KAH8372949.1 KAH8372949.1

LLPLNTMKRTKTTKAGPSKKAAKKEENAKPEKKSDPESNNSSDEGASSSKTVVSKPEYQNFEQFLAHLESQRSTTAQNIL

KFSFRKQRVRVLSKSNDVKEGCQGGVVYWMSRDGRVQDNWAMLFAQRLALKLELPLAVVFCLVPKFLNATIRHYKFMMGG

LQEVEQHCRALDIPFHLLLGPAVERLPEFVKSHDVGAVICDFAPLRLPRKWVEDVGKALPKSVPLVQVDAHNVVPLWVAS

DKQEYAARTIRNKINSKLGEFLTEFPQVVKHPHGTGCRDVHTVDWTAAYAMLQCDMDVGEVKWAKPGYKAACQQLYEFCS

RRLRHFNDKRNDPMADSLSGLSPWLHFGQISAQRCALEVQRFRGQHKASAEAFCEEAIVRRELADNFCFYNENYDSLKGL

SAWAYQSLDAHRKDKRDPCYTLEELEKSHTYDDLWNSAQLQLVREGKMHGFLRMYWAKKILEWTETPEQALEYAILLNDK

YSLDGRDPNGYVGCMWSIGGVHDMGWKERSIFGKIRYMNYQGCRRKFDVNGFVMRYGGKVHNKT

>Drosophila_set_hypoth_KAH8412809.1 KAH8412809.1

MDAHHVTLVHWFRKGLRVHDNPGLSQVFAAANAAPGKSVVRPIFILDPGILDWMQVGANRWRFLQQTLQDLDQQLRKLNS

RLFVVRGKPADVFPRIFKSWRVELLTFETDIEPYSIARDAAVQKLAKCEGVKVETHCSHTVYNPELVIAKNLGKAPITYQ

KFLGIVEQLKVPKVLDLPEMLKGNVQPPQDEVEKEDPSAYDYPTMDQLVKRPEDLGPNKFPGGETEALRRMEESLSDELW

VARFEKPNTAPNSLEPSTTVLSPYLKFGCLSSRLFHLRLKEILKRQTKHSQPPVSLIGQLMWREFYYTVAAAEPNFDRML

GNVYCLQIPWQEQADHLEAWTHGRTGYPFIDAIMRQLRQEGWIHHLARHAVACFLTRGDLWISWEEGQRVFEQLLLDQDW

ALNAGNWMWLSASAFFHQYFRVYSPVAFGKKTDPKGSYIRKYVPELAKYPAGSIYEPWKVSLADQRAYGCVLGTDYPHRI

VKHEIVHKENIKRMSAAYKVNREVRTGKEEESFEEKPETSVSGKRKAVRKTAGNAAKRKR

>Drosophila_sim_CRY-1_XP_002102858.1 XP_002102858.1

MATRGANVIWFRHGLRLHDNPALLAALADKDQGIALIPVFIFDGESAGTKNVGYNRMRFLLDSLQDIDDQLQAATDGRGR

LLVFEGEPAYIFRRLHEQVRLHRICIEQDCEPIWNERDENIRSLCRELSIDFVEKVSHTLWDPQLVIETNGGIPPLTYQM

FLHTVQIIGLPPRPTADARLDDATFVELEPEFCRSLKLFEQLPMPEHFNVYGDNMGFLAKINWRGGETQALLLLDERLKV

EQHAFERGFYLPNQALPNIHDSPKSMSAHLRFGCLSVRRFYWSVHDLFKNVQLRACVRGVQMTGGAHITGQLIWREYFYT

MSVNNPNYDRMEGNEICLSIPWAKPNEDLLQRWRLGQTGFPLIDGAMRQLLAEGWLHHTLRNTVATFLTRGGLWQSWEHG

LQHFLKYLLDADWSVCAGNWMWVSSSAFERLLDSSLVTCPVALAKRLDPDGTYIKQYVPELMNVPKEYVHEPWRMSAEQQ

EQYECLIGVHYPERIIDLSMAVKRNMLAMKSLRNSLITPPPHCRPSNEEEVRQFFWLADVVV

>Drosophila_sim_CRY-2__XP_002080051.1 XP_002080051.1

MDAQRSTLVHWFRKGLRVHDNPALSHIFTAANAAPGKYFVRPIFILDPGILDWMQVGANRWRFLQQTLEDLDNQLRKLDS

RLFVVRGKPAEVFPRIFKSWRVEMLTFETDIEPYSLTRDAAVQKLAKAEGVKVETHCSHTIYNPELVIAKNLGKAPITYQ

KFLGIVDQLKVPKVLGVPEKLKKMHTPPKDEVEQKDSAAYDCPTMEQLVKRPEELGPNKFPGGETEALRRMEESLKDEIW

VARFEKPNTAPNSLEPSTTVLSPYLKFGCLSARLFNQKLKEIIKRQPKHSQPPVSLIGQLMWREFYYTVAAAEPNFDRML

GNVYCMQIPWQEHPDHLEAWTHGRTGYPFIDAIMRQLRQEGWIHHLARHAVACFLTRGDLWISWEEGQRVFEQLLLDQDW

ALNAGNWMWLSASAFFHQYFRVYSPVAFGKKTDPQGHYIRKYVPELSKYPAGCIYEPWKASLADQRAYGCVLGTDYPHRI

VKHEVVHKENIKRMGAAYKVNREVRTGKEEESSFEEKSESSTSGKRKVRRAAGSAPKRKR

>Drosophila_sim_PL_X1_XP_002080494.3 XP_002080494.3

MFTLASYWRESFKIVLPLQAMKRTKAQKAGPSKKAAKSEKASSKPKSDQESSDEEASTSKALLVSKPDYQNFEQFLTHLE

HQRVCTAASIQEFSFRKKRVRVLSKTEDVKESSLGGVVYWMSRDGRVQDNWALLFAQRLALKLELPLTVVFCLVPKFLNA

TIRHYKFMMGGLQEVEQQCRALDIPFHLLMGPAVEKLPQFVKSKDIGAVVCDFAPLRLPRQWVEDVGKALPQTVPLVQVD

AHNVVPLWVASDKQEYAARTIRNKINSKLGEYLSEFPPVVQHPHGTGCKNVKAVDWSAAYASLQCDMEVDEVQWAKPGYK

AACQQLYEFCSRRLRHFNDKRNDPTADALSGLSPWLHFGHISAQRCALEVQRFRGQHKASADAFCEEAIVRRELADNFCF

YNEHYDSLKGLSSWAYQTLDAHRKDKRDPCYNLEELEKSLTYDDLWNSAQLQLVREGKMHGFLRMYWAKKILEWTATPEQ

ALEYAILLNDKYSLDGRDPNGYVGCMWSIGGVHDMGWKERAIFGKVRYMNYQGCRRKFDVNAFVMRYGGKVHKKK

>Drosophila_subobsc_CRY-1_XP_034664285.1 XP_034664285.1

MAARGANVLWFRHGLRLHDNPALLAALEEKDQGIALIPVFIFDGESAGTKSVGYNRMRFLLDSLQDIDEQLQAATEGRGR

LFVFEGEPTLIFRRLHEQVRLHKICAELDCEPIWNERDEAARLMCRELGIEYVEKVSHTLWDPRLVIETNGGIPPLTYQM

FLHTVQIIGVPPRPAVDAHIDDATFIQLAPELRQHLGCFDKIPNPEHFNIYSDNMGFLAKINWRGGETQALALLEERLKV

ERNAFERGYYLPNQAMPNIQEAPKSMSAHLRFGCLSVRRFYWSVHDLFENVQLAACVRGVQMAGGAHITGQLIWREYFYT

MSVNNPNYDRMEGNEICLSIPWAKPDEDLLQRWRLGQTGFPLIDGAMRQLLAEGWLHHTLRNTVATFLTRGGLWQSWEPG

LKHFLKYLLDADWSVCAGNWMWVSSSAFERLLDSSLVTCPVALAKRLDPEGVYIRRYVPELKNLPREFIHEPWRLSAEQQ

VQYECLIGVHYPERVIDLSKAVKRNMMAMTSLRNSLITPPPHCRPSNEEEVRQFFWLANY

>Drosophila_subobsc_CRY-2_XP_034668615.1 XP_034668615.1

MMDAKHTTLVHWFRKGLRVHDNPALSQIFAAANVAPEKFHVRPIFILDPGILDWMQVGANRWRFLQQTLDDLDQQLRQLH

SRLFVVRGKPVDVFPRIFKSWRVELLTFETDIEPYALKRDAAVQKLAKDAGIKVDTHCSHTIYNPELVIAKNLGKAPITY

QKFLSVIEQLKTPKVLELPEKLSKKALPPKDEVEQQDEAAYDCPTLQQLVKRPEDLGPNKFPGGETEGLRRMKESLRDEL

WVARFEKPNTAPNSLEPSTTVLSPYLKFGCLSARLFHQQLKEILKRQPKHSQPPVSLVGQVMWREFYYTVAAAEPNFDRM

LGNVYCLQIPWQEQADHLEAWTHGRTGYPFIDAIMRQLRKEGWIHHLARHAVACFLTRGDLWISWEEGQRVFEQLLLDQD

WALNAGNWMWLSASAFFHQYFRVYSPVAFGKKTDPQGHYIRKYVPELAKYPNGCIYEPWKATLSDQRAYGCVLGVDYPHR

IVKHELVHKENIKRMSAAYKVNREVRTGKEEDSPEADRSAAGKRKVAKSTGNAAKRKR

>Drosophila_subobsc_PL-lik_XP_034650866.1 XP_034650866.1

MLISSYWRKNFNKPNGFTVGLNLVRRLASIYTMKRTKTDTAGASKKAPKKEKKSAEPEEGASGSSGDEAVSSSSKLAQTK

PEYKNFDQFLAHLEQQRSEAAASVQEFLFKKKRVRVLSKANDVGEKCTGGVVYWMSRDGRVQDNWALLFAQRLALKLELP

LAVVFCLVPKFLNATIRHYKFMMGGLQEVEQQCRELGIPFHLLLGPAVDRLPEFVRSKHVGAVVCDFAPLRVPRKWVEDV

AKALPKNVPLTQVDAHNVVPLWVASDKQEYAARTIRNKINSKLGEFLSEFPPVIKHPHGDGCKKVKPIDWAAAYAMLECD

MDVDEVKWAKPGYKAACLQLYEFCSRRLGKFNDKRNDPTVDALSGLSPWLHFGQISAQRCALEVQRFRSQHKASADAFCE

EAIVRRELADNFCYYNENYDSLKGLSPWALQSLEAHRKDKRDPCYTLEELEQSLTYDDLWNSAQLQLVREGKMHGFLRMY

WAKKILEWTAAPEQALEYAILLNDKYSLDGRDPNGYVGCMWSIGGVHDMGWKERAIFGKIRYMNYQGCKRKFDVNAFVMR

YGGKVHKKKA

>Drosophila_subpulc_CRY-1_XP_037728777.1 XP_037728777.1

MATRGANVIWFRHGLRLHDNPALLAALADKDQGIALVPVFIFDGESAGTKSVGYNRMRFLLDSLQDLDDQLQLATEGRGR

LHVFEGEPAHIFRRLNEQVRLHRICIEQDCEPIWNERDETIRSLCRELSIDLVEKVSHTLWDPRTVIDTNGGIPPLTYQM

FLHTVQIIGLPPRPAADPRLDDASFVELAPELRRSLGYFEDMPTPEHFNVYGDNMGFLAKINWRGGETQALLLLEERLKV

EQHAFERGFYLPNQALPNIHDTPKSMSAHLRFGCLSVRRFYWSVHDLFKNVQLRACVRGVQMTGGAHITGQLIWREYFYT

MSVNNPNYDRMEGNEICLTIPWAKPNEDLLQRWRLGQTGFPLIDGAMRQLLAEGWLHHTLRNTVATFLTRGGMWQSWEHG

LQHFLKYLLDADWSVCAGNWMWVSSSAFERLLDSSLVTCPVALAKRLDPEGAYIKQYVPELMNVPKEFVHEPWRMSADQQ

EQYECLIGVHYPERIIDLSMDVKRNMLAMKALRNSLITPPPHCRPSNEEEVRQFFWLADVAV

>Drosophila_subpulc_CRY-2_XP_037711095.1 XP_037711095.1

MEAEKSTLVHWFRKGLRVHDNPALSQIFKAANVKPGKYYIRPIFILDPGILDWMQVGANRWRFLQQTLEDLDKQLRKLDS

RLFVVRGKPAEVFPRLFKSWRVELLTFETDIEPYSISRDEAVQKLAKCEGVRVETHCSHTIYNPDLVIAKNLGKAPITYQ

KFLGIVDKLKVPTVLGDPEKLLKKLEPPKDEVEQKDSEAYDYPTMEQLVKRPEELGPNKFPGGETEALRRMKESLKDELW

VARFEKPNTAPNSLEPSTTVLSPYLKFGCLSARLFNEKLKEILKRQPKHSQPPVSLVGQLMWREFYYTVATAEPNFDRML

GNIYCLQIPWQEHPEHLEAWTHGRTGYPFIDAIMRQLRQEGWIHHLARHAVACFLTRGDLWISWEEGQRVFEQLLLDQDW

ALNAGNWMWLSASAFFHQYFRVYSPVAFGKKTDPQGHYIRKYVPELAKYPAGCIYEPWKASLADQRTNGCVLGTDYPHRI

VKHEVVHKENIKRMGAAYKVNREVRTGKEEESSFEEKPETSTSGKRRVRKAAGNAPKRKR

>Drosophila_subpulc_PL-lik_XP_037716556.1 XP_037716556.1

MFTLATYWRESFKIVLPLHIMKRTKVTKAGPSKKSAKKGEDSPKKVDKSKQESSDEEASTSKASLASKPDYQNFEQFLTH

LDHQRVCAAANIQEFAFRKKRARVLSKTEDVEESSQGGIVYWMSRDARVQDNWALLFAQRLALKLELPLSVVFCLVPKFL

NATIRHYKFMMGGLQEVEQQCRSLDIPFHLLMGNAAENLPEFVKSKDIGAVICDFAPLRLPRKWVEDVGKALPKSVPLVQ

VDAHNVVPLWVASDKQEYAARTIRNKINSKLGEFLTEFPPVVKHPHGAGCKEAKPVDWSAAYDMLQCDTDVDEVQWAKPG

YKAACQQLYEFCTRRLRKFNDKRNDPMADALSGLSPWLHFGQISAQRCALEVQRFRGQHKASAEAFCEEAIVRRELADNF

CFYNEHYDSLKGLSSWAYQTLDTHRKDKRDPCYGLEELEKSLTYDDLWNSAQLQLVREGKMHGFLRMYWAKKILEWTATP

EEALEYAILLNDKYSLDGRDPNGYVGCMWSIGGIHDMGWKERAIFGKVRYMNYQGCKRKFDVNAFVMRYGGKVHNKK

>Drosophila_sul_hypoth_KAH8391103.1 KAH8391103.1

MKRTKATTSGAAKKKPKKEESSSSGEETELERTSSPKRESTSPGYKNVDQFIGHMQDQRLASAADVHDFVFRKNRVRLLS

AENDVKESCKGGVVYWMSRDARVQDNWALLYAQRTALKLKLPLSVVFCLVPKFLNATLRHYKFMLGGLQEVEQECRKLNI

AFHLLLGPAVERLPEFVAAQDIGAVVCDFAPLRLPRQWVTDVTKALPKNVPLLQVDAHNVVPVWVTSEKQEYAARTIRNK

INSKLPEFLTEFPPLIKHPHGKASKVKAVNWAAAQQQLTCDMSVDEVEWAKPGYKAACRQLYEFCTHRLRDFNEKRNDPM

ADAVSGLSPWLHFGQISAQRCILEVKRYAGKYKASVEGFCEETIVRRELADNFCYYNENYDSLKGVSEWAYQSLEAHRKD

KRSPCYTLDELEQSCTYDDLWNSAQLQLVNEGKMHGFLRMYWAKKILEWTETPEQALEYSILLNDKYSLDGRDPNGYVGC

MWSVGGIHDTAWKERAIFGKIRYMNYQGCKRKFDVTAFVMRYGGKAYKNNDN

>Drosophila_sul_hypoth_KAH8392372.1 KAH8392372.1

MSIELQSKSSQRRTLVHWFRKGLRVHDNPALLQVFDVARAAPHKYSVRPIFLLDPGILDWLQVGANRWRFLQQSLCDLDN

QLRELNSRLYVVRGKPVELFPKLFERWNVELLTYESDIEPYAVQRDAAVQKLAAVHQVHVDTYCSHTIYNPEVVMARNLG

KAPITYQKFLGIVEQLKLPKVREKPEKLPKEVQPSEDALELADASVYNCPTLEQLVKRPDELGDNKFPGGETEALRRLDA

SLRDEQWVASFEKPKTSPNSLEPSTTVLSPYLKFGCLSARLLYERLKGILARHPKHSKPPVSLVGQLLWREFYYTAAAAE

PNFDRMLGNTYCIQIAWNEQPDHLEAWTHGRTGYPFIDAIMRQLRQEGWIHHLARHAVACFLTRGDLWISWEEGQRVFEQ

LLLDQDWALNAGNWMWLSASAFFYQYFRVYSPVAFGKKTDPTGAYIKKYVPELAKYPANCIFEPWKATLSAQREYGCVLG

KDYPHRIVNHDIVHKENIKRMTAAYKVNREVRTGKQEPDEELDEPTGKRKASSIKSGRPAKRRR

>Drosophila_sul_hypoth_KAH8399309.1 KAH8399309.1

MALRSANVMWFRHGLRLHDNPALLSALADKDHGVALIPIFIFDGESAGTKSVGFNRMRFLLDSLQDIDQQLQSETEGRGR

LFIFEGNPVEIFRRLNEQVRLHKICVEQDCEPIWNERDDATKNLCNELGIEYVEKVSHTLWDPRTVIDTNGGIAPLTYQM

FLHTVQIIGLPPRPVHDPHFGGVSFVQLSPELRRDLGYFEQPPTPEHFNIYSDNMGYLSKINWIGGESQALELLGERLKI

EQHAFERGFYLPNQAMPNILDTPRSMSAHLRFGCLSVRRFYWSVHDLFKNVQLRACVRGVQMTGGAHITGQLIWREYFYT

MSVNNPNYDRMEGNEICLNIPWAKPNDEQLQRWRLGQTGFPLIDGAMRQLLAEGWLHHTLRNTVATFLTRGGLWQNWEYG

VQHFLKYLLDADWSVCAGNWMWVSSSAFERLLDSSLVTCPVALAKRLDPEGIYIKQYVPELQNLPKEFIHEPWRMSAEQQ

ERYECLIGVHYPERILDLAVAGKRNTLAMNSLRNSLITPQPHCRPSNEEEVRQFFWLVD

>Drosophila_suz_CRY-2_XP_016925170.1 XP_016925170.1

MDAERSTLVHWFRKGLRVHDNPALSQIFKAANAKSGKYYIRPIFILDPGILDWMQVGANRWRFLQQTLEDLDKQLQRLDS

RLFVVRGKPAEVFPRIFKSWRVELLTFETDIEPYSISRDEAVQKLAKSEGVRVETHCSHTIYNPDLVIAKNLGKAPITYQ

KFLGIVDKLKVPTVLGDPEKLLKKIEPPKDEVEQKDSEAYDCPTMEQLVKRPEELGPNKFPGGETEALRRMEESLKDELW

VARFEKPNTAPNSLEPSTTVLSPYLKFGCLSARLFYQKLKEILKRQTKHSQPPVSLVGQLMWREFYYTVAAAEPNFDRML

GNIYCLQIPWQEQPEHLEAWTHGRTGYPLIDAIMRQLRQEGWIHHLARHAVACFLTRGDLWISWEEGQRVFEQLLLDQDW

ALNAGNWMWLSASAFFHQYFRVYSPVAFGKKTDPQGHYIRKYVPELAKYPAGCIYEPWKASLADQRTYGCVLGTDYPHRI

VKHEVVHKENIKRMGAAYKVNREVRTGKEEQSSFEEKPETSTSGKRKVRKAAGNAPKRKR

>Drosophila_suz_LOW_QU_XP_036672555.1 XP_036672555.1

MTTRGANVIWFRHGLRLHDNPALLAALADKDQGIALIPVFIFDGESAGTKSVGYNRMRFLLDSLQDLDDQLQLATEGRGR

LHVFEGEPAHIFRRLNEQVRLHRICIEQDCEPIWNERDETIRSLCRELSIDLVEKVSHTLWDPRTVIDTNGGIPPLTYQM

FLHTVQIIGLPPRPAADPRLDDASFVELAPELRRSLGYFEEMPTPEHFNVYGDNMGFLAKINWRGGETQALLLLEERLKV

EQHAFERGFYLPNQALPNIHDTPKSMSAHLRFGCLSVRRFYWSVHDLFKNVQLRACVRGVQMTGGAHITGQLIWREYFYT

MSVNNPNYDRMEGNEICLTIPWAKPNEDLLQRWRLGQTGFPLIDGAMRQLLAEGWLHHTLRNTVATFLTRGGMWQSWEHG

LQHFLKYLLDADWSVCAGNWMWVSSSAFERLLDSSLVTCPVALAKRLDPEGAYIKQYVPEXMSVPKEFVHEPWRMSAEQQ

EQYECLIGVHYPERIIDLSMDVKRNMLAMKALRNSLITPPPHCRPSNEEEVRQFFWLADVAV

>Drosophila_suz_PL-lik_XP_016930561.1 XP_016930561.1

MFTLATYWRESFKIVLPLHIMKRTKVTKAGPSKKSSKKEDSPKKVDKSKQESSDEEASTSKASLASKPDYQNFEQFLTHL

DHQRVCTAASIQEFSFRKKRARVLSRTEDVEESSKGGIVYWMSRDARVQDNWALLFAQRLALKLELPLSVVFCLVPKFLN

ATIRHYKFMMGGLQEVEQQCRSMDIPFHLLMGNAAEKLPEFVKSNDIGAVICDFAPLRLPRKWVEDVGKALPKSVPLVQV

DAHNVVPLWVASDKQEYAARTIRNKINSKLGEFLTEFPPVIRHPHRTGCKEAKPVDWTAAYDMLQCDTDVNEVQWAKPGY

KAACQQLYEFCTRRLRKFNDKRNDPMADALSGLSPWLHFGQISAQRCALEVQRFRGQHKASAEAFCEEAIVRRELADNFC

FYNEHYDSLKGLSSWAYQTLDAHRKDKRDPCYGLEELEKSLTYDDLWNSAQLQLVREGKMHGFLRMYWAKKILEWTATPE

EALEYAILLNDKYSLDGRDPNGYVGCMWSIGGIHDMGWKERAIFGKVRYMNYQGCKRKFDVNAFVMRYGGKVHKKK

>Drosophila_tak_CRY-1_XP_016996585.2 XP_016996585.2

MATRGANVIWFRHGLRLHDNPALLAALADKDQGIALIPVFIFDGESAGTKCVGYNRMRFLLDSLQDIDDQLQAVTEGRGR

LHVFEGEPAHIFRRLHEQVRLHRICMEQDCEPIWNERDETIRSLCRDLGIDLVEKVSHTLWDPRTVIDTNGGIPPLTYQM

FLHTVQIIGLPPRPAADARLDDASFVELAPELRRCLGYFEQLPTPEHFNVYGDNMGFLAKINWRGGETQALLLLAERLKV

EQHAFERGFYLPNQALPNIHETPKSMSAHLRFGCLSVRRFYWSVHDLFKNVQLRACVRGVQMTGGAHITGQLIWREYFYT

MSVNNPNYDRMEGNEICLNIPWAKPNEDLLQRWRLGQTGFPLIDGAMRQLLAEGWLHHTLRNTVATFLTRGGMWQSWEHG

LQHFLKYLLDADWSVCAGNWMWVSSSAFERLLDSSLVTCPVALAKRLDPEGAYIKQYVPELMNVPKEFVHEPWRMTAEQQ

EQYECLIGVHYPERIIDLSMYVKRNMLAMKALRNSLMTPPPHCRPSNEEEVRQFFWLADVAV

>Drosophila_tak_CRY-2_XP_016994124.2 XP_016994124.2

MDKDRSTLVHWFRKGLRVHDNPALSQIFTAANAKPGKFFIRPIFILDPGILDWMQVGANRWRFLQQTLEDLDKQLRKLDS

RLFVVRGKPAEVFPRIFKSWRVELLTFETDIEPYSLSRDAAVQKLAKLEGVRVETHCSHTIYNPELVIAKNLGKAPITYQ

KFLGIVDQLKVPKVLGFPEKLKIKVVPPKDEVEQKDSAAYDCPTMEQLVKRPEELGPNKFPGGETEALRRMEESLKDELW

VARFEKPNTAPNSLEPSTTVLSPYLKFGCLSARLFHQRLKEILKRQTKHSQPPVSLIGQLLWREFYYTVAAAEPNFDRML

GNIYCLQIPWQEQPDHLEAWTHGRTGYPFIDAIMRQLRQEGWIHHLARHAVACFLTRGDLWISWEEGQRVFEQLLLDQDW

ALNAGNWMWLSASAFFHQYFRVYSPVAFGKKTDPQGHYIRKYVPELSKYPAGCIYEPWKASLADQRAYGCVLGTDYPHRI

VKHEVVHKENIKRMGAAYKVNREVRTGKEEQSSFEESTETSTSGKRKVRKAGGNAPKRKR

>Drosophila_tak_PL_XP_017012635.2 XP_017012635.2

MKRTKATKAGPSKKPAKKQEDSSKEEEQSEQDSSDAEPSTSKALLASKPDYQNFEQFLTHLEHQRSCTAPSIQEFSFRKK

RVRVLSKTEDVKESSDGGVVYWMSRDGRVQDNWALLFAQRLALKLELPLSVVFCLVPKFLNATIRHYKFMMGGLQEVEQQ

CRSLAIPFHLLMGPAAEKLPEFVKSRNIGAVVCDFAPLRLPRQWVEDVGKALPKSVPLVQVDAHNVVPLWVASDKQEYAA

RTIRNKINSKLGEFLSEFPPVVQHPHGTGCQEAKPVDWEAAYAMLQCDMDVDEVQWAKPGYRAACQQLYEFCTRRLRKFN

DKRNDPTADALSGLSPWLHFGQISAQRCALEVQRFRGQHKASADAFCEEAIVRRELADNFCFYNEHYDSLKGLSSWAYQT

LDAHRKDKRDPCYSLEELEMSLTYDDLWNSAQLQLVREGKMHGFLRMYWAKKILEWTATPEQALEFAILLNDKYSLDGRD

PNGYVGCMWSIGGVHDMGWKERAIFGKVRYMNYQGCRRKFDVNAFVMRYGGKVHKKK

>Drosophila_tei_CRY-1_XP_043641923.1 XP_043641923.1

MDAQRFTLVHWFRKGLRVHDNPALSQIFSAANAAPGKYFVRPIFILDPGILDWMQVGANRWRFLQQTLEDLDNQLRKLNS

RLFVVRGKPAEVFPRIFKSWRVEILSFETDIEPYSMSRDAAVQKLAKTESVKVETHCSHTIFNPELVIAKNLGKAPITYQ

KFLGIVEQLKLPKVLGSPEKLKNITSPPKDEVEQEDLAAYDCPTMEQLVKRPEELGPNKFPGGETEALRRMEDSLKDEIW

VARFEKPNTAPNSLEPSTTVLSPYLKFGCLSARLFNQKLTEIIKRQPKHSQPPVSLIGQLMWREFYYTVAAAEPNFDRML

GNVYCMQIPWQEHPDHLEAWTHGRTGYPFIDAIMRQLRQEGWIHHLARHAVACFLTRGDLWISWEEGQRVFEQLLLDQDW

ALNAGNWMWLSASAFFHQYFRVYSPVAFGKKTDPQGHYIRKYVPELSKYPSGCIYEPWKASLADQRAYGCVLGTDYPHRI

VKHEVVHKDNIKRMGAAYKVNREVRTGKQEESSFEEKSESSTSGKRKVRRVAGNAPKRKR

>Drosophila_tei_CRY-1_XP_043656850.1 XP_043656850.1

MATRGANVIWFRHGLRLHDNPALLAALADKDQGIALIPIFIFDGESAGTKNVGYNRMRFLLDSLQDIDDQLQAATDGRGR

LLVFEGEPANIFRRLHEQVRLHRICIEQDCEPIWNDRDESIRSLCRELSIDFVEKVSHTLWDPQLVIETNGGIPPLTYQM

FLHTVQIIGLPPRPTADARLEEATFMELDSELCRSLNLFEKLPAPDHFNVYADNMGFLAKTNWRGGETQALLLLDERLKV

EQHAFERGFYLPNQALPNIHDSPKSMSAHLRFGCLSVRRFYWSVHDLFKNVQLRACVRGVQMSGGAHITGQLIWREYFYT

MSVNNPNYDRMEGNEICLSIPWAKPKEDLLQRWRLGQTGFPLIDGAMRQLLAEGWLHHTLRNTVATFLTRGGLWQSWEHG

LQHFLKYLLDADWSVCAGNWMWVSSSAFERLLDSSLVTCPVALAKRLDPDGTYIKQYVPELINVPREFVHEPWRMSVEQQ

EQYECLIGVHYPKRIIDLSMAVKRNMLAMKTLRNSLITPPPHCRPSNEEEVRQFFWLADVVV

>Drosophila_tei_PL-lik_XP_043640532.1 XP_043640532.1

MLTNASYWRELFKIAMKRTKAQKAGPSKKAAKSEEGSFKPKSDQESNDEEASTSKTSLVSKPEYQNFEQFLTHLEHQRIC

TAANIQEFPFRKKRVRVLSKTDDVKESSLGGVVYWMSRDGRVQDNWALLFAQRLAFKLELPLSVVFCLVPKFLDATIRHY

KFMMGGLQEVEQQCRALDIPFHLLMGPAVEKLPEFVKSKDIGAVVCDFAPLRLPLQWVENVSKALPKSVPMVQVDAHNVV

PLWVTSDKQEYAARTIRNKINSKLGEFLSEFPPVVRHPFGTGCKNVNAVDWLAAYASLQCDMEVDEVQWAKPGYKAACQQ

LYEFCSRRLRHFNDKRNDPTADALSGLSPWLHFGHISAQRCALEVQRFRGQYKASADSFCEEAIVRRELADNFCFYNEHY

DSLKGISSWAYQTLDAHRKDKRDPCYRLEELEKSLTYDDLWNSAQLQLVREGKMHGFLRMYWAKKILEWTATPEQALEYA

ILLNDKYSLDGRDPNGYVGCMWSIGGVHDMGWKERAIFGKVRYMNYQGCRRKFDVNAFVMRYGGKVHKKK

>Drosophila_vir_CRY-1_XP_002053627.1 XP_002053627.1

MALRGANVIWFRHGLRLHDNPALLAALSDKDQGIALIPIFIFDGESAGTKSVGYNRMRFLLDSLQDIDMQLQKATEGRGG

LLMCQGQPTQIFRRLHERVRLHKICVEQDCEPIWNERDEATKSLCRELNIEYVEKVSHTLWDPRTVIDTNGGIAPLTYQM

FLHTVQIIGLPPRPIPDPNFEGVSIAQLPQELRKDIGCLEKTPTPEDFNIYSDNMGYLAKMNWIGGETQALSLLGERLKV

EQHAFERGYYLPNQALPNILETPKSMSAHLRFGCLSVRRFYWSMHDLFKNVQMRACVRGVQMTGGAHITGQLIWREYFYT

MSVNNPQYDRMEGNEICLSIPWSKPDADQLQRWRLGQTGFPLIDSAMRQLLAEGWLHHTLRNTVATFLTRGGLWQNWELG

LEHFLKYLLDADWSVCAGNWMWVSSSAFERLLDSSLVTCPVALAKRLDPNGAYIKQYVPELKNVPKEFIHEPWRMSSEQQ

ERYECLLGFHYPQRIIDLSLASKRNTMAMKALRNSLIEPPPHCRPSNEEEVRQFFWLTN

>Drosophila_vir_CRY-2_XP_002052207.1 XP_002052207.1

MSAKRRTLVHWFRKGLRVHDNPALSQIFNDARASPEKFYVRPIFILDPGILDWMQVGANRWRFLQQSLVDLDKKLKELNS

RLYIVRGKPVDVFPELFERWNVQLLTFESDIEPYALQRDTTVQKIASEHGVKVDTHCSHTIYNPELVIAKNLGKAPVTYQ

KFLGIVDKLKLPKVLAVPEGLPDGIKPIADEFEAVDSCVYDCPTLDQLVKRPQELGVNKFPGGETEALRRMEASLIDENW

VAAFEKPNTAPNSLEPSTTVLSPYLKFGCLSARLLHERLKEILVRKPKHSKPPVSLVGQLLWREFYYTVAAADPNFDRML

GNAYCLQIPWQKQPDHLEAWTHGRTGYPFIDAIMRQLRQEGWIHHLARHAVACFLTRGDLWISWEDGQRVFEQLLLDQDW

ALNAGNWMWLSASAFFHQYFRVYSPVAFGKKTDPTGAYIRKYVPELAKYPAGCIYEPWKATLSAQREYGCVLGVDYPHRI

VNHDIVHKENIKRMSAAYKVNREVRTGKQEDQNDYEPSPSQSTGKRKASSEAKKTVAKRRR

>Drosophila_vir_PL_XP_032292197.1 XP_032292197.1

MQIVKYKFVSLGGFRALAQTLHMKRNKNSSAAGGAKKKPKKNESSGSDEETTVTIATEKKTKPSYNNFEQFLSHMQAQRI

AAARDVHDFAFKKKRVRVLSTVSDVKEQSKGGVLYWMSRDARVQDNWALLFAQRLALKLELPLTVVFCLVPKFLNATLRH

YKFMLGGLQEVEQQCHELNISFQLLLGPAAERLPEFVTAEDIGAVICDFAPLRLPRQWVADVVKALPGHVPLTQVDAHNI

VPLWVTSEKQEYAARTIRNKINSKLGEFLSEFPPVIRHPHGKGQAKESTDWTAAYALLSCDKTVDAVDWAKPGYTAGCRQ

LYEFCTRRLRHFNEKRNDPTADALSGLSPWLHFGQISAQRCILEVQRYSAMHKASADAFCEEAIVRRELADNFCYYNEHY

DSLKGLHDWAYQTLQAHRKDKRSPCYTLEELEQARTYDDLWNSAQLQLVKEGKMHGFLRMYWAKKILEWTETPELALEYS

ILLNDKYSLDGRDPNGYVGCMWSIGGVHDQGWKERDIFGKIRYMNYQGCKRKFDINAFVMRYGGKVHQKKEN

>Drosophila_wil_CRY-1_XP_002072813.1 XP_002072813.1

MANVTCGANVIWFRHGLRLHDNPALLSALADKDQGIALIPIFIFDGESAGTKDVGYNRMRFLLDSLQDLDGKVQQATEGR

GRLHIFQGQPKEIFRRLHQQLQLKKICFEQDCEPIWSQRDASVKSLCQELGIEWVEMVSHTLWDPHTVIETNGGIPPLTY

QMFLHTVEIIGLPPRPTNDPNWNNVGFVEISQELQRELKYLQHFPTPEYFNIYCDNMGYLAKINWRGGETEALLLLQERL

KVEQSAFERGYYLPNQALPNILDTPKSMSPHLRFGCLSVRRFYWNVHDLFKNVQLQACVRGVQMTGGSHITGQLIWREYF

YTMSVNNSNYDRMDGNEICLNIPWSKPHEEKLERWRLGQTGFPLIDGAMRQLLAEGWLHHTLRNTVATFLTRGGLWQSWE

HGVQHFLKYLLDADWSVCAGNWMWVSSSAFERLLDSSLVTCSQAMAKRLDPDGLYIKQYVPELRNVPKEYIHEPWRMSAE

DQERYECLIGVHYPEPIIDLSLALKRNTLAMTNLRNSLITPPPHCRPSNEEEVRQFFWLAESM

>Drosophila_wil_CRY-2_XP_002064736.1 XP_002064736.1

MEAKTKATLIHWFRKGLRVHDNPALMQIFTTAQAAPKKFYVRPIFILDPGILDWMQVGANRWRFLQQTLDDLDKQLRQLN

SRLYVVRGKPIEVFPRLFKSWNVELLTFESDIEPYAQTRDAAVQKIAKAAGIKVETHCSHTIYNPELVIAKNLGKAPITY

QKFLALVEKLKLAKVLDKPEKLDALTQPSRDELEQENDKVYDCPSQEQLVKKPNELGPNIFPGGETEGLRRMEDSLNDEL

WVARFEKPNTAPNSLEPSTTVLSPYLKFGCLSSRLFYQRLKEILAKHPKHSQPPVSLVGQVLWREFYYTVAAAEPNFDRM

LGNVYCLQIPWQEHPEHLEAWTYGRTGYPFIDAIMRQLRQEGWIHHLARHAVACFLTRGDLWISWEEGQRVFEQLLLDQD

WALNAGNWMWLSASAFFHQYFRVYSPVAFGKKTDPQGNYIRKYCPELANYPNKQIYEPWLATLQAQREYGCVLGEDYPHR

IVKHELVHKENIKKMSEAYKINREVRTGKQEDSMLNDDNDELEDDRSATGKRKGNKAAGAAKRRRK

>Drosophila_wil_PL_XP_002063542.4 XP_002063542.4

MYFVIRAKFSRLLIQESMKRTRTKETQPQPQKKLKKEKETESESESSSNNEEEVPSTSSKKSVVHTSFQEFLTHLQHQRL

ATASNIQEFSFKKKRVRILSKVIDVREGCRGGVVYWMSRDGRIQDNWALLFAQRLALKLDLPLAVVFCLVPKFLNATLRH

YKFMLGGLKEVEKECRDLNIGFHLLLGPAREELPKFVRDNDIGAVICDFAPLRVPRQWVEDVTKGLPKDVPFTQVDAHNI

VPLWVASDKQEYAARTIRNKINSKLGEYLTEFPVVAKHPHGKSSAKINWTSAYEMLECDKSVDEVEWAQPGYNAACRQLY

EFCTRRLGKFHNQRNNPTADALSGLSPWLHFGQISAQRCVLEVKRYAKQHKASTEAFCEETIVRRELADNFCYYNENYDS

LKGVTSWAYQSLDAHRKDPRDPCYKLEDLETARTYDDLWNSAQLQLVKEGKMHGFLRMYWGKKILEWTESPEQALEWTIL

LNDKYSLDGRDPNGYVGCMWSIGGIHDTAWKERPIFGKVRYMNYQGCKRKFDVNAFVARYGGKVYPKKDKK

>Drosophila_yak_CRY-1_XP_002096288.1 XP_002096288.1

MATRGANVIWFRHGLRLHDNPALLAALADKDQGIALIPVFIFDGESAGTKNVGYNRMRFLLDSLQDIDDQLQAATDGRGR

LLVFEGEPANIFRRLHEQVRLHRICIEQDCEPIWNDRDESIRFLCRELNIDFVEKVSHTLWDPQLVIETNGGIPPLTYQM

FLHTVQIIGLPPRPTADARLEDATFVELDSEFCRSLNLFEKLPAPDHFNVYTDNMGFLAKINWRGGETQALLLLDERLKV

EQHAFERGFYLPNQALPNIHDSPKSMSAHLRFGCLSVRRFYWSVHDLFKNVQLRACVRGVQMTGGAHLTGQLIWREYFYT

MSVNNPNYDRMEGNEICLSIPWAKPKEDLLQRWRLGQTGFPLIDGAMRQLLAEGWLHHTLRNTVATFLTRGGLWQSWEHG

LQHFLKYLLDADWSVCAGNWMWVSSSAFERLLDSSLVTCPVALAKRLDPEGTYIKQYVPELINVPREFVHEPWRMSVEQQ

EQYECLIGVHYPERIIDLSMAVKRNMLAMKTLRNSLITPPPHCRPSNEEEVRQFFWLADVVV

>Drosophila_yak_CRY-2_XP_002090823.1 XP_002090823.1

MDAQRSTLVHWFRKGLRVHDNPALSQIFSAANAAPGKYFVRPIFILDPGILDWMQVGANRWRFLQQTLEDLDNQLRKLNS

RLFVVRGKPADVFPRIFKSWRVEILSFETDIEPYSMTRDAAVQKLAKTEGVKVETYCSHTIYNPELVIAKNLGKAPITYQ

KFLGIVEQLKLPKVLGSPEKLTNITSPPKDEVEQEDLAAYDCPTMEQLVKRPEELGPNKFPGGETEALRRMEDSLKDEIW

VARFEKPNTAPNSLEPSTTVLSPYLKFGCLSARLFYQKLTEIIKRQSKHSQPPVSLIGQLMWREFYYTVAAAEPNFDRML

GNVYCMQIPWQEHPDHLEAWTHGRTGYPFIDAIMRQLRQEGWIHHLARHAVACFLTRGDLWISWEEGQRVFEQLLLDQDW

ALNAGNWMWLSASAFFYQYFRVYSPVAFGKKTDPQGHYIRKYVPELSKYPSGCIYEPWKASLADQRAYGCVLGTDYPHRI

VKHEVVHKDNIKRMGAAYKVNREVRTGKQEESSFEEKSETSTSGKRKVRRVAGNAPKRKR

>Drosophila_yak_PL_XP_002089533.2 XP_002089533.2

MFTNALYWRESFKIAMKRTKAQKVGPSKKAAKSDEGSFKPKSDQESNDEEASTSKSSLVSKPEYQNFEQFLTHLEHQRIC

TAANIQEFPFRKKRVRVLSKTDDVKESSLGGVVYWMSRDGRVQDNWALLFAQRLAFKLELPLSVVFCLVPKFLNATIRHY

KFMMGGLQEVEQQCRALDIPFHLLMGPAVEKLPEFVKSKDIGAVICDFAPLRLPRQWVENVSKALPKSVPMVQVDAHNVV

PLWVASDKQEYAARTIRNKINSKLGEFLSEFPPVVRHPFGTGCKNVNAVDWLAAYASLQCDMEVEEVQWAKPGYKAACQQ

LYEFCSRRLRHFNDKRNDPTADALSGLSPWLHFGHISAQRCALEVQRFRGQYKASADSFCEEAIVRRELADNFCFYNDQY

DSLKGLSSWAYQTLDAHRKDKRDPCYRLEELEKSLTYDDLWNSAQLQLVREGKMHGFLRMYWAKKILEWTATPEEALEYA

ILLNDKYSLDGRDPNGYVGCMWSIGGVHDMGWKERAIFGKVRYMNYQGCKRKFDVNAFVMRYGGKVHKKK

>Dufourea_nov_PREDIC_XP_015428952.1 XP_015428952.1

MDKASPAKRIKSSDLLEKFKNNRENTAESILTFNFNKKRVRLLSNLNDVKDGCKGILYWMFRDVRVQDNWALLFAQRTAL

KNKVPFHICFCIMPKFLEASMRHYKFLLQGLQEIEEECKSLNINFQLLYGEPNVAIVKFVKMYRIGAIIADFHPLKLPMS

WISDVQSNLPDVPICQVDAHNIVPCWEASQKQEFSARTIRNKINTKLEEFLTEFPPVVKHPYLTEEKFGKNNWKMALNNV

EVDASVCEIIWAKPGYKNGIKEFENFLQNRLKKYANERNDPLSNATSNLSPWFHFGMISVQRCILEIQNYKKLYSKSVES

FMEEAIIRRELSDNFCFYNENYDLVEGAHAWAIETLNKHRKDKREYIYSLNELEKSETHDDLWNACQNQMVTTGKMHGFL

RMYWAKKILEWSETPENALQSANYLNNKYSIDGCDPNGYIGCAWSICGIHDHGWSERQIFGKIRYMNYEGCKRKFEVKKF

VSQWEKKETDNNVSK

>Dufourea_nov_PREDIC_XP_015438404.1 XP_015438404.1

MTGSRNREINSEVGVRGDGEKHTVHWFRKGLRLHDNPSLREGLAGASTFRCVFVLDPWFAGSTNVGINKWRFLLQCLEDL

DCSLRKLNSRLFVIRGQPADALPKLFKEWGTTNLTFEEDPEPFGRVRDHNISALCKELGISVVQKVSHTLYKLDEIIEKN

GGKPPLTYHQFQNVVASMDSPEPSVPTVTSVCVGSAYTPLKEDHDDHYGVPTLEELGFDTEGLRPPVWVGGESEALARLG

RHLERKAWVASFGRPKMTPQSLLPSQTGLSPYLRFGCLSTRLFYYQLTDLYKKIKKAVPPLSLHGQLLWREFFYCAATKN

PNFDRMQGNPICVQIPWDKNVEALAKWANGQTGFPWIDAIMTQLREEGWIHHLARHAVACFLTRGDLWISWEEGMKVFDE

LLLDADWSVNAGMWMWLSCSSFFQQFFHCYCPVRFGRKADPNGDYIRRYLPVLKSFPTRYIHEPWNAPLSVQRTAKCIIG

QDYSLPMVNHSKSSRINIERMKQVYQQLNKYRGNGASLKGETVGE

>Eciton_bur_hypoth_KAH0945976.1 KAH0945976.1

MNFQGQILDKKNGYLILEKYEIEALQQTISDRMIEKSVCPIARSGSELIFSRSSALLLKGISRKKMTGNNNEMSQEMTSG

VQNDSKKHTVHWFRKGLRLHDNPSLSEGLAGASTFRCVFVLDPWFAGSTNVGINKWRFLLQCLEDLDSSLRKLNSRLFVI

RGQPADALPKLFKEWGTTNLTFEEDPEPFGRIRDHNISALCKELGISVVQKVSHTLYKLDKIIEKNGGKAPLTYHQFQNV

VASMDPPESPVSAVTATCIGSAYTPLKNDHDDHYGVPTLEELGFDTEGLLPPVWVGGESEALARLERHLERKAWVASFGR

PKMTPQSLLPNQTGLSPYLRFGCLSTRLFYYQLTELYKKIKKAMPPLSLHGQLLWREFFYCAATRNPNFDRMQGNSICVQ

IPWDKNAEALAKWANGQTGFPWIDAIMTQLREEGWIHHLARHAVACFLTRGDLWISWEEGMKVFDELLLDADWSVNAGMW

MWLSCSSFFQQFFHCYCPVRFGRKADPNGDYIRRYLPVLKNFPSKYIHEPWNAPLSIQHAAKCIIGKEYSLPMVNHSKSS

RINIERMKQVYQQLNKYRGNGASFKGENIGLLNALLASPKNSDEGKQKQDSPCQENNQKMDSISNSAQQQQQQKQQ

>Ephemera_dan_KAF4518708.1_(6-4)-phot KAF4518708.1

MSSSGTVIHWFRKGLRLHDNPALLSAIEKIDDKCFELRPIFILDPWIVTKLRVGTNRWRFLQQSLQDLDSKLRAIGSRLY

VLRGKPENVFPKIFKDWKVKRLTFELDTEPYARERDARIEKLAKEAGVPVAQKVSHTLYDTERVIMTNMGKPPLTYQKMV

SLAEQMGRPLLPKSAPESLPDECKVGAAGLPVNETDWPNTDVPSLEELGVDPAKLQPCLYPGGETEALRRMEEHLAKKAW

ICKFEKPETSPNSLKPSTTVLSPYIKFGCLSARTFYHGSAHSKPPVSLVGQMLWREFFYTVSAATPNFDRMQGNPICCQI

PWGSNPTHLKAWTEGQTGFPFIDAVMRQLRQEGWIHHLARHSVACFLTRGDLWISWEEGMKVFEEFLLDADWALNAGNWM

WLSASAFFHQYFRVYSPIAFGKKTDKDGSFIRKYVPELAKYPAEYIYEPWKSSLSVQRAAGCIIGQDYPRPIVNHDEASK

RNKSRMAEAYKKNKQNKTDEGSSNAKKRGAGNDRGFAKKFKQ

>Ephemera_dan_KAF4519522.1_B566_EDAN0 KAF4519522.1

MASPEKRVKVGDNTKSPGSTSTSNVGDFIANIENEREKAASSILEFKFNKKRVRILSKASDVAVNASGIVYWMSRDQRVQ

DNWALLFAQKLAMKNKIALHVCFCLLPKFLDATIRHFKFMLDGLVEVQTECSKLGLQFHLLQGDAASVLPSFVKTHKIGA

LVADFSPLRTHLDWTESVKKTLPSDVDAHNLVPCWVASDKLEYGARTIRNKITSKLPEFLTQFPPVVKHPYPASFKAEPI

DWSELESSLEVDTSVGPVTWAAPGTQAGLAVLHEFCQKRLKNFGTKRNDPTINVLSNLSPWFHFGQISVQRAILTVNKFK

SKYAESVAAFVEESVVRRELSDNFCFYNPKYDSIDGTNAWAKTTLIQHKKDKRPYLYTQEELDSAHTHDDLWNSAQIQLV

KEGKMHGFLRMYWAKKILEWTVSPEEALRIAIYLNDRYSLDGRDPNGYVGCMWSICGIHDQGWAEREVFGKIRYMNYAGC

KRKFDVSAFVARYGGKVHFNKSNGQQTLKVTKGKSK

>Ephemera_dan_KAF4522540.1_Chryptochr KAF4522540.1

MAGMNKLPRISVLWFRHGLRLHDNPALLDAIKNSDEFYPIFIFDGESAGTRLVGYNRMRFLLEALADLDRQLRAVGGQLY

LLQGSPSRLFQRLWEERSLTKLCFEQDCEPIWHARDAKVRATCIDRCIHCNEHISHTLWDPKEVIRTNGGSPPLTYQLFL

HTVSVIGDPPRPVADPDWSNVKFGKLPEIITQEFKMFACVPSPEHFGLTPPATSPVVRWVGGETQALKHLQERLQVEEAA

FMRGVILPNRASPDLLAPPTSQSAALRFGCLSVRREGWMHHVARNSVACFLTRGALWQSWEHGLQHFLKHLIDADWSVCA

GNWMWVSSSSFEQLLDCSQYVCPVNYGRRRYVPELRRYPVEYLYEPWKAPLETQQNAGCIIGEDYPERIVDHTKALKQNR

KYMQKLRDEMMEATPDHCCPSNEDEVLSFMQLPEACFEELNMSQA

>Ephemera_dan_KAF4523712.1_cry_2 KAF4523712.1

MNCVLNKDNTTSSNTVSEKHTVHWFRKGLRLHDNPSLHEGLKDATSLRCVFILDPWFAGSSNVGINKWRFLLQCLEDLDR

SLRKLNSRLFVIRGQPADALPKLFKEWGTTCLTFEEDPEPFGRVRDQNIMTLCRELGLSVISRISHTLYDLEKILEKNGG

KAPLTYYQFQNVVASMDPPPEPEHTVDSFGDTNTPVSDDHDEKYGVPSLEELGFVVDGLLPPVWRGGESEALARLERHLE

RKAWVASFGRPKMTPQSLLASQTGLSPYMRFGCLSTRLFYYQLTDLYKKIKKTVPPLSLHGQLLWREFFYCAATKNPNFD

KMVGNPICVQIPWDKNPEALAKWANGQTGFPWIDAIMTQLREEGWIHHLARHAVACFLTRGDLWISWEEGMKVFEELLLD

ADWSVNGGMWMWLSCSSFFQQFFHCYCPVRFGRKADPNGDFIRQVRRYLPVLKKFPTRYIHEPWNAPESVQKAAKCVIGK

DYGMPMVNHAVASRINIERMKQVYQQLSKYHGAGLLETVPSSQMNGINLYENITLTTNSSDTTVLYKSNVNRQKSKQKRH

H

>Eufriesea_mex_PREDIC_XP_017765362.1 XP_017765362.1

MTGSRNNEINPEVTVHGEGGKHTVHWFRKGLRLHDNPSLREGLAGASTFRCVFVLDPWFAGSTNVGINKWRFLLQCLEDL

DCSLRKLNSRLFVIRGQPADALPKLFKEWGTTNLTFEEDPEPFGRVRDHNISALCKELGISVVQKVSHTLYKLDEIIERN

GGKPPLTYHQFQTVVASMDPPEPPVPTVTSACVGSAYTPLKEDHDDHYGVPTLEELGFDTEGLLPPVWVGGESEALARLE

RHLERKAWVASFGRPKMTPQSLLPSQTGLSPYLRFGCLSTRLFYYQLTDLYKKIKKAVPPLSLHGQLLWREFFYCAATKN

PNFDRMQGNPICVQIPWDKNVEALAKWANGQTGFPWIDAIMTQLREEGWIHHLARHAVACFLTRGDLWISWEEGMKVFDE

LLLDADWSVNAGMWMWLSCSSFFQQFFHCYCPVRFGRKADPNGDYIRRYLPVLKNFPTRYIHEPWNAPLSIQRAAKCIIG

KEYSLPMVNHSKSSRINIERMKQVYQQLNKYRGNGAALKGETVGLLNTLPPPPVKVNEEEKKTKQSPPPSEDQPKMESLT

KTTHQQQHHH

>Eufriesea_mex_PREDIC_XP_017767330.1 XP_017767330.1

MITLVSWILYHINKAKRNRKKLDVMNAFKRRKTLDLLKKFEHNRKNTCESIMTFNFNKQRIRLLTDLNDVKINCKGILYW

MFRDIRIQDNWALLFAQKTALKNNVPLHICFCIMPSFLNASIRYYKFLLKGLIEIEQECKTLNISFHLLHGEPNVSILKF

VKTYNMGAVITDFYPLKLSISWIDKVQKNLPKDVPICQVDAHNIVPCWYASSKQEFSARTIRNKLNTKLEEFLTEFPPVI

RHPYITKQKFKNNDWEIALQNLEVDKSTTEITWAKPGYRNGIKELENFLTYRLKKYGDERNNPLLNTISNLSPWFHFGMI

SIQRCILEIKEYKNLYKKSVESFMEETIIRRELSDNFCFYNDKYDLVEGAYPWAIETLNKHRTDKRKYIYSLNHLENSET

HDDLWNACQNQMVIIGKMHGYLRMYWAKKLLEWTETPEIALEWANYLNNKYSIDGCDPNGYVGCMWSICGVHDHGWTERN

IFGKIRYMNYEGCKRKFNVAEFVMKWKKKETDEFT

>Eumeta_jap_CRY-1_GBP64109.1 GBP64109.1

MIVWPSSDSKNGSALAQPRGDDSTLDVAENHKKQLRPIYIIDDDIKNRCNVGINRVRFLLESLIDLNSNLRKLNLQLYVV

NGKPLEVFSKLFTEWKVDFLTFEKAVEPEIIEEDEHIEELSNKNNVFVVQRISHTIYDCQSIINKNNGNIPLTYPKFLSL

VQDVQVKITNEIIKPVSNYCQPSIDAFEKQDPNCYDVNQHFKLSSEDLSTEICYPGGETEALKRLNDILQKKDWICNFEK

PNTSPNSLEPSTTVLSPYISLGCLSAKLFYKELKTIERNHPHTKPPVSLLGQLMWREFYYVAATGTVNFDKMIGNPICMQ

IPWDKNESFLKAWAEGKTGYPFIDAIMKQLISEGWIHHLARHMVACFLTRGDLWISWEEGAKIFEKYLLDYDWALNSGNW

LWLSASAFFYKYFRVYSPIAFGKKTDKDGLYIRKYVPELKKYPSEYIYEPWKAPLSVQKQANCIIGTRVDLALGSRMGVG

HTNEVVRTDSNSITIVRPIPGGSYDPHDPFWIRLYSYHYLRSTGRALATVAAQGVGDVGDRRRPRA

>Eumeta_jap_CRY-1_GBP68250.1 GBP68250.1

MPEKENLRMILWMGGETAALKQMNKRLLVEYETFCRGSYLPTLGEPDLLGPPISLSPALRFGCLSVRKFYWALQDLFKQI

HGGELQSTYFVSGQLIWREYFYTMSVNNPNYGQMADNPICLDIPWKEPQGDELQRWADGLTGFPFVDAAMRQLRSEGWLH

HAVRNTVASFLTRGTLWLSWEHGLNHFLKYLLDADWSVCAGNWMWVSSSAFEALLDTSECACPVRLGQRLDPGGEYVRRY

VPELRNLPQDYIFEPWKAPLEVQQYAKCVIGRDYPSPIVSHKVAAQRNRNAMMKLREMLQKAPLHCCPSSDDEIRQFMWL

REDGVEVTAE

>Eumeta_jap_CRY-1_GBP87169.1 GBP87169.1

MLVKIAQNWFKCFQSGNLDIRDEPRSSRLVMDKVDAILKRLKQDRHIDSYDIAEELGIENKTACVDVKRIFHLCRLVIGN

IDVQERFKFNIGLESKRRGGPVSGSSAPARALATSSVQYSNARGREKERYTKIESRREKEFLLQSLEDLDSSLRKLNSRL

FVVRGQPTDALPKLFREWGTTALTFEEDPEPYGRVRDHNIMTKCREVGITVSSRVSHTLYQLDKIIELNGGKAPLTYHQF

QALVASMPPPPPAEPAICRQTLHGAVTPLGDDHDDRFGVPTLEELGFETEGLKPPMWIGGENEALIRLERHLERKAWVAS

FGRPKMTPQSLLASQTGLSPYLRFGCLSTRLFYYQLTELYKRVKRVRPPLSLHGQLLWREFFYCAATRNPNFDRMEGNPI

CVQIPWEKNQDALAKWANGQTGYPWIDAIMIQLREEGWIHQLARHAVACFLTRGDLWISWEEGMKVFDELMLDADWSVNA

GTWMWLSCSSFFQQFFHCYCPVRFGRKADPQGDFISMDFEVAVQLVILLIDPPFDTKQGVLLPPLGWDISISINFFIRIQ

LCGLKERITLDVAFLESSATLVSCQLDLLAL

>Eumeta_jap_PL_GBO99360.1 GBO99360.1

MHVARKDKRAYIYSLEELKDAQTHDDLWNSAQIQMVKEGKMHGFLRMYWCKKILEWTTSPEEALRFAIYLNDHYSIDGRD

ANGYVGCMWSICGVHDQGWAERAVFGKIRYMNYNGCKRKFDVKAFVARYGGQAHVHAPGPPIPASRTKPIKEPKRRI

>Euphydryas_edi_unname_CAH2099909.1 CAH2099909.1

MSTAAETLPASSTRTPVATLGATLPVRPPGKHTVHWFRKGLRLHDNPALREALTDATTFRCVFIIDPWFASSSNVGINKW

RFLLQCLEDLDSNLKKLNSRLFVVRGQPADALPKLFREWGTTALTFEEDPEPYGRVRDHNIMTKCREVGITVTSRVSHTL

YKLDNIIERNGGKAPLTYHQFQALIASMPPPPPAEAAVSNQTLNGAVTPLTDDHDDRFGVPTLEELGFETEGLKPPVWIG

GENEALLRLERHLERKAWVASFGRPKMTPQSLLASQTGLSPYLRFGCLSTRLFYYQLTELYKRIKRVRPPLSLHGQILWR

EFFYCAATRNPNFDRMEGNPICVQIPWEKNQDALAKWANGQTGFPWIDAIMIQLREEGWIHHLARHAVACFLTRGDLWIS

WEEGMKVFDELLLDADWSVNAGMWMWLSCSSFFQQFFHCYCPVRFGRKTDPNGDFIRKYIPVLKNLPTRYIHEPWVAPEA

VQQAANCVVGRDYPLPIVDHTKASQINIERIKQVYAQLAKYKPQQGPSISQVLQRPTVMQSSPSPTSIITNINQSNYLCS

QTPDPTPQSSPSTQYKEDDIFLRPTKNNIRSTGIVNKNISFKQVVIVQQEKNSRVQQANAENNYDMNGVNGDTTKPFKIN

AMQNSEKTGNYDFKNLVINNGIQKLGTDQLVYLNQQTNKNELYNQDVKIDEYSTSKPKYFFTDNGVIPHNESDQNFTSNY

GNNYNSNERNTHVKSEDSQNENKMNINSPSPSDSRRTDENK

>Euphydryas_edi_unname_CAH2102172.1 CAH2102172.1

MLAGNKRTATSDTKLSSLAKKSKKLDANTTTEDFAKEIQKKREDTSKSISDFKFNKKRLRIISQEQMVPEDCEGIVYWMS

RDSRVQDNWAFLYAQYLALKNEVPLHVCFCLIAKYLDASVRQFHFLLKGLEKVSKECKKLNISFHLLEGHGGDVLPQWVK

DHKIGAVVCDFNPLRVPLGWLEVAKKKLKKDVPLIQVDAHNVVPCWVASDKQEYSARTIRNKINSKLDEFLTEFPPVIKH

PYTSKFEPEPIDFDEAIKTREADESVKPIEWASPGYDSALEILKSFIEKRLKIFATKRNDPTVDALSNLSPWFHFGQISV

QRVALCVQEYKSKYTESVNSYLEEAIVRRELADNFCFYCENYDSIKGASAWAQKTLDDHRKDKRTHIYTLEQFSNATTHD

DLWNAAQLQLVQEGKMHGFLRMYWCKKILEWTPSPEEALQYAIYLNDHYSVDGRDPSGYVGCMWSICGIHDQGWAERAVF

GKVRYMNYEGCKRKFDVPAFVARYGRREYLTRESVAELVCT

>Euphydryas_edi_unname_CAH2106081.1 CAH2106081.1

MLGGSVLWFRHGLRLHDNPSLQTALQDKSIPFFPIFIFDGETAGTKIVGYNRMRYLLEALNDLDTQFKKYGGKLHMIKGK

PDVVFRRLWEEFGIRKICFEQDCEPIWRARDDSVRNVCREIGVSCCENVSHTLWDPETVINANGGIPPLTYQMFLHTVEI

IGNPPRPVDDVDLKGVNFGTLTKSFYDEFTVFDKAPKPEDLGVFLENEDIRMIRWVGGETAALYQMQNRLAVEYETFLRG

SYLPTHGNPDLLGPPISLSPALRFGCLSVRRFYWCVQDLFQQVNQGRLASTQFITGQLIWREYFYTMSVNNPNYAQMAGN

PICLDIPWKEPENDELQRWKEGRTGFPFVDAAMRQLRTEGWLHHAVRNTVASFLTRGALWLSWEHGLAHFLKYLLDADWS

VCAGNWMWVSSSAFEALLDSGECVCPVRLGRRLEPSGHYVRRYVPELARMPVDYIYDPWNAPLEVQERVGCVIGKDYPAP

LVDHLAAAQRNRSAMQELRRILEKAPPHCCPSSEDEVRQFMWLGEESQVEIATA

>Euphydryas_edi_unname_CAH2107041.1 CAH2107041.1

MSTLQTVVHWFRLDLRLHDNLALRNAINEAENRKHYLKPIYVIEPDIKNKIGANRLRFLLQSLQDLDVNLRKINSRLFIV

KGRAVDLLPELFEKWNVKFLTLQVDIDAELVKQDEIIEKFCEEKDIFVVKRMQHTVYDFNSVIKKNNGGIPLTYQKFLSL

VADTQVKDTIEIAKQISDDCKSTDFELEEYKVPSLEEVGINESELLECKYPGGETEGLKRLNIYMNKKQWVCNFEKPNSS

PNSIEPSTTVLSPYISHGCLSAKLFYHKLKQVENGMKHTLPPVSLMGQLMWREFYYVAGAGTKNFDKMVGNTVCTQIPWG

KNEVHLKAWAEGKTGYPFVDAIMRQLKQEGWIHHLARHMVACFLTRGDLWISWEEGAKVFEDYLLDYDWSLNAGNWMWLS

ASAFFYKYYRVYSPVAFGKKTDKEGLYIRKYVPELKKYPSEFIYEPWKAPKSVQRTAGCVVGEDYPNRIVDHDKVHKVNI

QKMNAAYKVNKEKKALKRKR

>Fopius_ari_PREDIC_XP_011304667.1 XP_011304667.1

MLQPRNSYSPYPPIPSFPASSNTLCLGRKLSTEKTWKSIYMVYNISVISWLHNNCLKMAERQQRNENQLASNLKSFPSDA

KHVVHWFRKGLRLHDNPSLRHGLNEASTFRCVFVIDPWSAGSKSIGVNKWRFLLQCLEDLDSSLRKLNSRLFVIRGQPTD

VLPKLFREWGTTHLTFEEDPEPFGRARDHNISTLCKELAICVMAMASHTLYKLDEILDRNKGKAPLTYHQFQTIVAAMNP

PSPPVEIVTAETIGNAYTPLQENHDEIYGVPTLEELGFDIEGLKPPVWIGGESEALIRLERHLERKAWVASFGRPKMTPQ

SLLPSQTSLSPYLRFGCLSTRLFYYQLRALYKKVKKAVPPLSLHGQLLWREFFYCAATKNPNFDRMQGNPICLQIPWDKN

PEALAKWANGQTGFPWIDAIMTQLREEGWIHHLARHAVACFLTRGDLWISWEEGMKIFDELLLDADWSVNAGMWMWLSCS

SFFQQFFHCYCPVRFGRKADPNGDYIRRYLPVLRHFPTRYIHEPWIAPLSIQRAAKCIIGHDYSLPIVNHGQCSKTNIER

MKQVYQQLKKYRDNVQAGLLWV

>Fopius_ari_PREDIC_XP_011311459.1 XP_011311459.1

MQQWVNGNNPMVGEHIILSPVNGRSIHRKLCCVEWAAASIMEFSLNKKRIRVLSNAKEVAKDSKGILYWMFRDPRVQDNW

AFLYAQKLALKNRLPLHVCYCIVPKFLDATLRHYKFLVESLEEVSTDCRDLNVNFHLLLGEPNSVVLDFVKKYTMGALVI

DFFPLRLPRFWVKDIQEKLPEDIPLCQVDAHNIVPCWQTSDKLEYAARTIRPKINSKLPEYLTEFPPVIRHPYDSAFKFP

GIDWKNCLNGVLIDKTVDRVEWCKPGYRGAVEQLEMFVTERLKNYNEKRNDPVEDATSGLSPWFHFGQISVARVILEVEE

HKKSCQASVNSFMEEAIVRRELSDNFCYYNENYDKIEGSNAWAIQTLDAHRKDKREWIYTLEEFETSQTHEDLWNAAQNQ

LVREGKIHGFMRMYWAKKILEWSRTPEDALAWSIYLNDRYSMDGRDPNGYVGCMWSICGIHDQGWKEREIFGKIRYMNYK

GCERKFDVKAYVKKFDGKIVNKEKSKKIVKSTKKSRK

>Formica_exs_CRY-1-_XP_029670486.1 XP_029670486.1

MTGSNNNEMDQGVTSGVRGDGRKHTVHWFRKGLRLHDNPSLREGLAGASTFRCVFVLDPWFAGSTNVGINKWRFLLQCLE

DLDCSLRKLNSRLFVIRGQPADALPKLFKEWGTTNLTFEEDPEPFGGVRDHNISALCKEIGISVVQKVSHTLYKLDEIIE

RNNGKPPLTYHQFQNVVAGMDPPEPPVPTVTAACIGSAYTPLKDDHDDHYGVPTLEELGFDTEGLLPPVWVGGESEALAR

LERHLERKAWVASFGRPKMTPQSLLPSQTGLSPYLRFGCLSTRLFYYQLTDLYKKIKKAVPPLSLHGQLLWREFFYCAAT

KNPNFDRMQGNPICVQIPWDKNIEALAKWANGQTGFPWIDAIMTQLREEGWIHHLARHAVACFLTRGDLWISWEEGMKVF

DELLLDADWSVNAGMWMWLSCSSFFQQFFHCYCPVRFGRKADPNGDYIRRYLPVLKNFPTRYIHEPWNAPLSIQHAAKCV

IGKEYSLPMVNHNKTSRINIERMKQVYQQLNKYRDNGVSFKGEKIGLLNALLAPTKETDEEKRKQDSPNQENEQKMETIS

NSPQQQQ

>Frankliniella_occ_KAE8746400.1_cry_2,_par KAE8746400.1

MCSSESESEFITSPSAGSAATSIRPSPALSAATASAAAPRTKHSVHWFRKGLRLHDNPSLRHGLKGATTLRCIYFLDPWF

AGSSNVGINKWRFLLQCLEDLDRSLRKFNSRLFVIRGQPADALPKLFKEWGTTNLTFEEDPEPFGRVRDQNIMAMCKELS

ISVVSKVSHTLYELEKIIEKNGGKPPLTYHQFQNVVASMDPPPQAMPTITSRNMQDCYTPIEDDHDEKYGVPTLEELGFD

TEGLKPAVWLGGETEALARLEHHLERKAWVASFGRPKMTPQSLLASQTGLSPYLRFGCLSTRLFYYQLTDLYKKIKKAVP

PLSLHGQLLWREFFYCAATRNPNFDKMQGNPVCVQIPWDHNLEAIAKWANGQTGFPWIDAIMTQLREEGWIHHLARHAVA

CFLTRGDLWISWEEGMKVFEELLLDADWSVNAGMWMWLSCSSFFQQFFHCYCPVRFGRKADPNGDYIRRYLPILKNFPTR

YIHEPWSAPENVQRAAKCVVGKDYPKPMVNHGTASRINIERMKQVYTQLSKYRGS

>Frankliniella_occ_KAE8747724.1_6-4_Photol KAE8747724.1

MSKATAVHWFRKGLRLHDNPALVAALKSEIELRPVYIFDPWYEKNVLCGPNRWRFLHQSLTDLDKSLRDIGSRLFVFRGT

PEEVFTSLFSEWKVQRLTFEIDIEPYALERDEQIIKIARQEDVEVIQKISHTLYNTELVLKANMDKPPLTYQKLVSLLQS

LGEPPEAVAAPKELLSEQRVSSKLLDSHDYDIPTLEDLGVDQSQLGPCLFPGGETEGLARLGRCLERKEWISTFEKPKTI

PNSLSPSTTVLSPYIRFGCVSSRLTYHKINEILKVYKKHSKPPVSLIGQMYWREFYYVVASATPNFDKMIGNKVCCAVPW

DENPEFVEKWAQAQTGYPFIDACMRQLRQEGWIHHLARHAVACFLTRGDLYQSWEVGQKVFEELLLDADWALNAGNWMWM

SASAFYHQFYRVYSPVAFGKKTDVLGQYIR

>Frankliniella_occ_XP_026276018.1_photolyase XP_026276018.1

MSSNKEPPKKKIKVNDSPSSPSVSAQSNLKQRLMLDRKETAESILNFKFNKKRVRILSKATEVPSDCKGIAYWMFRDERV

QDNWAFLFAQKLALKNKVPLHVCFCLLTKFLDGTIRQFKFLLKGLREVQKECEELNIEFHMLYGSGGDVMPGFVEKNKIG

ALVIDFMPLRNVMAYADQLKKALPKDVPLCQVDAHNIVPCWEASNKLEYGARTIRPKIHKQLPEYLTQFPPVIKHPYSGP

LKAEKVDWDRCEAHLEVDRSVDEVEWAVPGYSGGIATLESFINKRLKFFGTKRNDPTQDALSNISPWLHFGQVSAQRCIL

VVKALRSKYPESVDGYIEEAIIRRELSDNFCFYNPKYDSIEGTNDWAKKTLNDHKKDKRPYLYTREELRDSKTHDDLWNS

AQIQLVKEGKMHGFLRMYWAKKILEWTASPEQGLADAIYLNDRYSLDGRDPSGYVGCMWSVCGIHDQGWGEREVFGKIRY

MNYQGCKRKFDISAFVARYGGKVYTKSATMDKFLTKGKKK

>Frankliniella_occ_XP_026282294.1_cry-1 XP_026282294.1

MATSDNNMCDKATVLWFRRGLRLHDNPALIKALSTKNVFYPIFIFDGETAGITKESSYNRVRFFLESLKDLDRQLRAKKG

KLHFIKGDPVKVFEAIHNIVPLELVSFEQDPEPMWESRDSKAKAYFDQHTIKWTEEISHTLWNPLDIIKANGGVPPLTYE

MFLHILSVVGDPPRPVEDPEWEGVKFGFLPLNIGNEVTVYHSIPTPEQLGYLQTRSSDQQINRWIGGEQTALIKLQDRLK

VEERAFLDGIYLPNQARPNLLGPSSSQSAALSFGCLSIRKFYWAVHDMFNKIYLGHTQLNQSITGQFIWREFFYTMSIKN

PYYGEMERNPLCLNLPWTKNREDLAKKWENGLTGYPFIDAVMRQLHQEGWVHHVARNAVACFLTRGDLWISWEVGLRLFL

RLLLDADWSLCAGNWMWVSSSAFEQLLDCSSCVCPVNYGRRLDPQGEYIRRYIPELRNYPIEYLYEPWKAPLKVQEMAGC

YIGQEYPERIVDHAVVSEKNRKMMENIRLTLVEESTPHCCPSNEEEVRQFMWLPESCSDYLCANRK

>Frieseomelitta_var_CRY-1-_XP_043510193.1 XP_043510193.1

MKMTGSRSSEINPEVTLHGEGGKHTVHWFRKGLRLHDNPSLREGLVGASTFRCVFVLDPWFAGSTNVGINKWRFLLQCLE

DLDCSLRKLNSRLFVIRGQPADALPKLFKEWGTTNLTFEEDSEPFGRVRDHNISALCKELGISVVQRVSHTLYKLDEIIE

RNGGKPPLTYHQFQTVVASMDPPEPPVPTVTSVCVGSAYTPLKEDHDDHYGVPTLEELGFDTEGLLPPVWVGGESEALAR

LERHLERKAWVASFGRPKMTPQSLLPSQTGLSPYLRFGCLSTRLFYYQLTDLYKKIKKAVPPLSLHGQLLWREFFYCAAT

KNPNFDRMQGNPICVQIPWDKNVEALAKWANGQTGFPWIDAIMTQLREEGWIHHLARHAVACFLTRGDLWISWEEGMKVF

DELLLDADWSVNAGMWMWLSCSSFFQQFFHCYCPVRFGRKADPNGDYIRRYLPVLKNFPTRYIHEPWNAPLSVQRAAKCI

VGKDYSLPMVNHSKSSRINIERMKQVYQQLNKYRGNGASLKGETVGLLNALPLPSVKENEEEKKKTKQSPPPSENQSKME

TLVKGTQHQQQHHQHQ

>Frieseomelitta_var_PL_X1_XP_043518404.1 XP_043518404.1

MNQLNPSKRKKVVDLLKKFEENRRNTSESIMTFSFNKKRIRRLSKLNDVKENCKGILYWMLRDFRIQDNWALLFAQKIAL

KNDVPLHICFCIMPNFLNASIRYYKFLLKGLLEIEKECKQLKINFHLLHGEPNMRILKFVKMYNMGAVIVDFYPLKLPMY

WVDNVQKNLPKNIPICQVDAHNIVPCWHASSEQEFAARTIRNKINVKLEEFLTEFPPVIEHPYLTKENFESNNWDIALQD

VDTDKLVNEITWAEPGYMGGIKELENFIQNRLQKYGDERNNPLSNATSNLSPWFHFGMISVQRCILEIKEYKRLYKKSVE

SFMEETIIRRELSDNFCFYNEKYDLVEGAYPWAIETLNKHRKDKRKYVYSLSQLENFKTHDDLWNACQNQMVIVGKMHGF

LRMYWAKKILEWTETPEIALEWANYLNNKYSIDGCDPNGYVGCMWSICGVHDHGWSERDIFGKIRYMNYEGCKRKFNVAE

FVKKWGKKEDN

>Galleria_mel_CRY-1_XP_026751200.1 XP_026751200.1

MAKPTVIHWFRLDLRIHDNLALRNAINEAENRQHFLRPIYVIDPDIKNKIGINRLRFLIQSLQDLDFNLKKLNSRLYVIK

GKAIDIIPKLFDEWQVKYLTTQVDIDPEIVKQDEIIEKIANEKDIFIVRRVQHTIYDVHSVIRKNNGNVPLTYQKFLSLV

QDLQVKSTIDILKSISDHCKTPDNCCNDYNVPNLEDFSIDENTLDLCKYQGGETVGLKRLHMYIAKKQWICKFEKPNSSP

NSIEPSTTVLSPYISHGCLSSKLFYHKLKEVENGVPHTMPPVSLMGQLMWREFYYTAGTGTENFDKMVGNSLCTQIPWVK

NDVHLKAWAEGQTGYPFVDAIMRQLKQEGWIHHLARHMVACFLTRGDLWISWEDGAKIFQDYLLDYDWSLNAGNWMWLSA

SAFFYKYFRVYSPVAFGKKTDKEGLFIRKYVPELKKIPSEFIYEPWKAPKSVQITAGCIIGENYPKRIVDHDKIHKENMQ

KMSVAYKVNKEKKSLKRPR

>Galleria_mel_CRY-1__XP_031765239.1 XP_031765239.1

MLGGSVLWFRHGLRLHDNPSLHDAIEDRKAPFFPVFIFDGESAGTKLVGYNRMRYLLEALDDLDNQFKRHGGRLFMIKGK

PTNVIRRLWEEFGIRKLCFEQDCEPVWRARDDSVKAACREIGVSCREHVSHTLWEPDTVIRANGGIPPLTYQMFLHTVAT

IGDPPRPVPNADLCGVQFGILPECFHEEFTVFDKTPKPEDLGVFLENEDIRMIRWVGGETTALKQMEQRLAVEYATFCRG

SYLPTHGNPDLLGPPISLSPALRFGCLSVRRFYWSVQDLFHKVHQGRLAATHFITGQLIWREYFYTMSVNNPNYGQMAGN

PICLDIPWKNPEGDELQRWTEGRTGFPFVDAAMRQLRTEGWLHHAVRNTVASFLTRGTLWLSWEHGLNHFLKYLLDADWS

VCAGNWMWVSSSAFEALLDSGECACPVRLGQRLDPSGEYVRRYVPELSRMPVQYIYEPWKAPIDVQERADCVIGKNYPAP

VVNHIVAAQKNRNAMNELRNILQKAPPHCCPSSEEEIRQFMWLSEDNQQSNTA

>Galleria_mel_CRY-1-_XP_026764725.1 XP_026764725.1

MSAAAETLPASSARPHTPEVPLPAPSTQCHVGKHTVHWFRKGLRLHDNPALREGLNGAITFRCVFIIDPWFASSSNVGIN

KWRFLLQCLEDLDNSLRKLNSRLFVVRGQPADALPKLFKEWGTTALTFEEDPEPYGRVRDHNIMTKCREVGITVISRVSH

TLYKLDKIIERNGGKAPLTYHQFQALIASMPPPPPAEAAITAQMLNGATTPVADDHDDRFGVPTLEELGFETEGLKPAVW

LGGESEALVRLERHLERKAWVASFGRPKMTPQSLLASQTGLSPYLRFGCLSTRLFYYQLTELYKRVKQVCPPLSLHGQIL

WREFFYCAATRNPNFDRMEGNPICVQIPWEKNHEALAKWASGQTGFPWIDAIMIQLREEGWIHHLARHAVACFLTRGDLW

ISWEEGMKVFDELLLDADWSVNAGMWMWLSCSSFFQQFFHCYCPVRFGRKTDPNGDFIRRYIPALKNMPTRYIHEPWMAP

ESVQQAARCTIGRDYPLPMVNHEKVSQINIERIKLVYSQLAKYKPQATLNSQALQRPNVMQSSPSPTSIITSINQSNYLC

SQTPDPQNTGSQAVPYKETATHVFQQPSKHISRAESKQQFKQVVIVQQKSNVTPETIIKRSQLSSENYINDGQLNVTYKT

NQKIDTLQTTQENYNFKNLAINNYNQEYFNREIYQNQQMNGNEVCQDALKNSNYVYVKPKFYLTSTDNGVVRNDTSQSFV

STASTKNYTVDEKSVGEKPSDSSCLPEINSDKVFAVTEDETNTSANNITP

>Galleria_mel_PL_XP_026758871.1 XP_026758871.1

MRLKLLVKTTCHYLKMASAPKKPKLSTASTSSDETKQTIEDFIKKIHVKREETAESISKFKFIKKRLRIISQSQLVPESC

EGIVYWMSRDSRVQDNWALLFAQQLALKNEVPLHVCFCLVAKYLDASIRQFHFLLKGLEKVAADCKKLNISFHLLEGSGA

DVLPQWVIDHSIGAVVCDFNPLRVPLSWLEGLKKKLQNDVPLIQVDAHNIIPCWVTSDKQEYSARTIRNKINSKLEEYLT

EFPPVIKHPYTSKFEPEPIDWNEAIESREADKSVGPIEWAKPGYDEAMKMLKSFLDVRLKIFASKRNDPMQDALSNLSPW

LHFGQISAQRVALCVQEYKTKFTESVNAFLEEAIVRRELADNFCFYCEHYDSIKGASSWAQKTLDDHRKDKRTYIYSLEE

LSKSLTHDDLWNSAQLQLVKEGKMHGFLRMYWCKKILEWTPSPEDALKYAIYLNDHYSMDGRDPNGYVGCMWSICGIHDQ

GWAERAVFGKIRFMNYDGCKRKFDVNAFVARYGGKVHKYVPKK

>Glossina_fus_CRY-1_XP_037892833.1 XP_037892833.1

MSANVLWFRHGLRLHDNPALLEALADREKGIALVPIFIFDGESAGTKCTGYNRLRFLLDSLRDLDDQFRSQRGRLYTFQG

NPTTIFKELHECFGINKLCVEQDCEPIWNRRDNDVKELCNELGIKWVEKISHTLWNPRQVIQTNGGIPPLTYQMFLHTVQ

VIGLPPRPAENPNWTGVNILNINETILEKLPGFTEIPQPEQFNVFAEDLNRLALVKWKGGETEALILLEERLKVEGEAFK

RGYYLPNQANPNILETPKSMSPHLRFGCLSVRKFYWAVHDLFEDVQQHVKMFGYQIMSGAHITGQLIWREYFYTMSVNNP

YYDRMEENEICLNIPWAPVNDEQLESWKLGKTGFPLVDAAMRQLLAEGWIHHTLRNTVATFLTRGGLWFNWEHGLQHFLK

YLLDADWSVCAGNWMWVSSSAFERLLDSSLVTCPLAMAKRLDPFGQYIKQYVPELAHVPKEYIHEPWRMPLSEQKRSECI

IGEHYPERIIDFVKAAESNMTAMRNLRQDLIEGGAPPPPHCRPSDEAEVRQFFWLV

>Glossina_fus_CRY-2-_XP_037899254.1 XP_037899254.1

MSGKPSLVHWFRKGLRVHDNPALTDVFEQARAYPSSYSIRPIYILDSTVTEWTKIGANRWRFLQESLEDLHSQLEALGSR

LYVIRGSPSTVFARLFKEWHVEVLTFEEDIEPYALKYGAAVKEMAKASNVTVKTHYSHTIYDPHSIMRINEDKAPLTYQK

FMELVKTLEQSKPLEKPKSLRNLNKPEKDFHEKEDIKCYDIPLLDELVKTKEELGVNKFKGGETEALKRLNEVLADEKWI

IEFEKPNTKPNSLEPSTTALSPYLSFGCLSSRYFCQRLTDILKRCPNHTKPPASLMGQLMWREFFYTAATYEPNFDRMMG

NSFCLQIPWLSNAAHLDAWTFGRTGYPFIDACMRQLRHEGWIHHLARHAVACFLTRGDLWISWEEGEKVFEELLLDQDWA

LNAGNWMWLSASAFFYKYFHVYSPVAFGKKTDRSGSYIRKYVPELAQYPGDKIYEPWKVSLADQREYGCVLGSDYPHRIV

NHEVEHKENIKRMSAAYKVNREVRTGKSEEDNADEARKRKNKTSSKQSAKMKKV

>Glossina_fus_PL_XP_037895799.1 XP_037895799.1

MLKSVKTTAAFINFNAHKSLFKMKRSKQSAKISKKIKFSNRQEFDEDHNNSLAESFRQKRLCCSDSVIDFAYKKKRVRIL

TENQREVREDCEGPVVYWMYRDQRVQDNWAFLYAQRLALKLELPLCVCFCLLPKYIHTTLRHYKFMLTGLEEVAQECEDL

NVNFHLLNGPAHQSLVEFLKEVDAATVVCDFSPLRLPLQWLEEIKEVLPTTIPFVQIDAHNVVPVWIASQKQEYAARTIR

NKINSQLEEYLTEFPPLIKHKYLNKKPSTKVNWRSIYNTLKCLKTVDEVSGVMPGYKNACRKLLEFCQKRLKLFHEKRND

PNIDALSGLSPWFNFGQISIQRCILEIKTYEHKFKDSVVAFCEEAIVRRELADNFCFYNQNYDNFEGLQDWSRKTLNEHR

KDVRSPCYSLEEFEQARTHDDLWNSAQLQLMREGKMHGFLRMYWAKKILEWSKSPEDALEFSLLLNDRYSLDGTDPNGYV

GCMWSIGGIHDQGWAERKIFGKIRYMNYQGCKRKFDVAAFVARYGGVAHIKT

>Gonioctena_qui_hypoth_KAG5867875.1 KAG5867875.1

MASLKPRGANGKIALDKLTKDLFLKDISESRKSQGESDDFDFNKKRCRILSKNENIKEKSDGILYWMYRDCRVQDNWAMI

YAQRLAMKQKLPLFVCFSIKDAHQQYPTKRHFKFLLEGLKLVQKECEHLNISFYLLNSSPKDLAKLIENNNIGGVVCDFS

PLKHPKKLHESLLEHLPNDIPVVQVDAHNIVPVWKASDKQEGMAKFLRTKITKHLPEYLTGFPVISKHKYSGKLDIKNKI

EKIDDAFSHYKPKWDVAEVKWCGGPGEEAGYLQLHDFIIKNLRHYGETSNDPSKDNSSKLSPWINFGQISAQRCALEVKS

VDSVYKEQCDKYLEELIVRSELTDNYCFYNNNYDNINGAANWAKETLKLHAKDKRAWVYTREQLERAETHDEMWNSAQLQ

AHHEGKIHNYMRMYWCKKILEWTESPEQAIEYGLWLNDTFCLDGTDPNGYVGVMWSICGVHDQGWREREIFGKIRYMVDY

SLRRKYNMDAYCARFGRKILGDSKKNAAIIPKEPKESKKKQVDSKKGAKRKAT

>Gonioctena_qui_hypoth_KAG5876664.1 KAG5876664.1

MSGSTGQTSNGQEKHTVHWFRKGLRLHDNPSLKEGLKGAKTFRCVFVLDPWFAGASSVGINKWRFLLQCLEDLDRNLRKL

NSRLFVIRGQPADALPKLFKEWGTTALTFEEDPEPFGKVRDDNITALCEELGITVIQRASHTLYHLDHIIELNGGKAPLT

YHQFLAVIACMGPPPQPELPVTASTLNGAYTPLADDHDEKYGVPTLEELGFDTEGLNPPVWQGGESESLARLERHLERKA

WVASFGRPKMTPQSLLPSQTGLSPYLRFGCLSTRLFYYQLTDLYKKIKKAFPPLSLHGQLLWREFFYCAATKNPNFDKMN

GNAICVQIPWDKNAEALAKWANGQTGFPWIDAIMTQLRKEGWIHHLARHAVACFLTRGDLWISWEEGMKVFEELLLDADW

SVNAGMWMWLSCSSFFQQFFHCYCPVKFGRKADPNGDYIRKYLPALKNMPLRYIHEPWLAPENIQHAAKCIIGKSYPLPM

VNHTTASRINIQRMEQVYQQLANYKIVENTRYPVKDRYSETFQRQPNIVTVSNPDS

>Gryllus_bim_BAX56238.1_cry_1 BAX56238.1

MTELSEVRKNASVLWFRHGLRLHDNPALQETLKDNDVFLPIFIFDGKTAGTNIIGYNRMKFLLESLQDIDNKLQEVGGKL

SLIRGNPVEVFHFISEKFNLRKICFEQDCEPIWQERDNAVKKFCLENKIECFEKVSHTLWDPYLVQKTNGGVPPLTFQMF

LHTISVLGSPPRPIGDVDWTLVEFGTLTPLPFPHNLHIFQHFPTPEDFGIYPEEKNGDRIIQWIGGETQALIHLKERLKV

EEDAFRKGYYLPNQARPDLLGPPSSQSAALRFGCLSVRRFYWSIQDIFTKIHGNNQVPNQHITSQLIWREFFYTMSVGNK

YYDEMERNPICLNIPWKENTKGHLELWEQGKTGYPFIDAVMRQLVQEGWIHHVARNAVACFLTRGVLWISWEAGLKFFLK

YLLDADWSVCAGNWMWVSSSAFEQLLDCSHCMCPVNYGRRLDPWGEYIKRYIPELRNYPVEYLYEPWKAPLHVQEQAGCI

VGKDYPQRMIDHLQASEKNRQYMEDIRNQLMNPPPHCRPSNEKETREFMWCHDNCFDH

>Gryllus_bim_BAX56244.1_cry_2_tran BAX56244.1

MTDVYQQLPLAAARGAGGPPGYGAPPAKHTVHWFRKGLRLHDNPSLREGLKGASTFRCIFILDPWFAGSSNVGINKWRFL

LQCLEDLDQSLRKLNSRLFVVRGQPADALPKLFKEWGTTNLTFEEDPEPFGRVRDQNIMAMCREMGISVVSRVSHTLYRL

ESIIEKNGGKPPLTYHQFQTVVASMDSPPAAEPRITARAIEGIHTPLHDDHDDRFGVPTLEELGFDTEGLLPPVWTGGES

EALARLERHLERKAWVASFGRPKMTPQSLQASQTGLSPYLRFGCLSTRLFYYQLTDLYKKIKKACPPLSLHGQLLWREFF

YCAATNNPNFDRMNGNPICVQIPWDRNPEALAKWATGQTGFPWIDAIMTQLREEGWIHHLARHAVACFLTRGDLWISWEE

GMKVFEELLLDADWSVNAGMWMWLSCSSFFQQFFHCYCPVRFGRKADPNGDYIRKYLPVLKNFPAKYIHEPWNAPESVQR

AARCIVGKEYSLPMVNHAVASRINIERMKQVYQQLSKYRGPPGLLAAVPSSQQPNRQSNTNNPCSKYGSGGLGRIKQEEG

DIVSDQEGKENNHKHHHTLHSLNQQHN

>Habropoda_lab_PREDIC_XP_017789593.1 XP_017789593.1

MTGSSSSELNPEVGVRGEGGKHTVHWFRKGLRLHDNPSLREGLAGASTFRCVFVLDPWFAGSTNVGINKWRFLLQCLEDL

DCSLRKLNSRLFVIRGQPADALPKLFKEWGTTNLTFEEDPEPFGRVRDHNISALCKELGISVVQRVSHTLYQLDEIIERN

GGKPPLTYHQFQNVVASMDPPEPPVSTITSACVGSAYTPLKEDHDDHYGVPTLEELGFDTEGLLPPVWVGGESEALARLE

RHLERKAWVASFGRPKMTPQSLLPSQTGLSPYLRFGCLSTRLFYYQLADLYKKIKKAIPPLSLHGQLLWREFFYCAATKN

PNFDRMQGNPICVQIPWDKNVEALAKWANGQTGFPWIDAIMTQLREEGWIHHLARHAVACFLTRGDLWISWEEGMKVFDE

LLLDADWSVNAGMWMWLSCSSFFQQFFHCYCPVRFGRKADPNGDYIRRYLPILKNFPTRYIHEPWNAPLSVQRAAKCIIG

KDYSLPMVNHSKSSRINIERMKQVYQQLHKYRGNGASLKGETVGLLNTLPPPPPPPVKKNEEEKKKTKQSPAPPSENQPK

MEGVSKTVQHQQQHHHQHQHLHQHQHQHQHQRQQ

>Habropoda_lab_PREDIC_XP_017792011.1 XP_017792011.1

MDELSPSKRRKVFDLLXSIVTFNFNKKRIRLLNNLNDVKTECKGILYWMFRDIRVEDNWALLFAQKTALKHSLPLHICFC

IMPSFLNASTRYYKFLLKGLMEIEEECKKLSIHFHLLHGEPNLSVLKFIKTYKMGAVITDFYPLKLPMSWIDNVQKNLPK

DIPICQVDAHNIVPCWYASSKQEYAARTIRTKINTKLEEFLTEFPPVIKHPYLTKEKFENNNWEIALQNVQADDTSINEI

TWARPGYKNGIKELDCFLQNRLKMYADKRNNPLLNTISDLSPWFHFGMISIQRSVLEIKEYRKLYPKSVESFMEEAIIRR

ELSDNFCFYNEKYDLVEGAHPWAIETLNKHRKDKRKYIYSLNQLEDSETHDDLWNACQYQMVAIGKMHGFLRMYWAKKIL

EWTETPEVALKWANYLNNKYSIDGCDPNGYVGCMWSICGVHDQGWREREIFGKIRYMNYEGCKRKFNIAEFVKKWGK

>Halyomorpha_hal_CRY-1-_XP_014279454.1 XP_014279454.1

MTEKHTVHWFRKGLRLHDNPSLRQGLKGAKTFRCIFILDPWFANASNVGINKWRFLLQCLEDLDRSLRKLNSRLFVLRGQ

PADILPKLFKEWGTTCLTFEEDPEPFGRVRDQNISTMCKGMNITVISLVAHTLYKLEFIIERNGGRAPLTYHQFQTVVAG

MDSPPLPDPPVTAATIGDAVSPISDNHDEKYGVPTLEELGFCTEGLVPGVWLGGESEALSRLERHLERKAWVASFGKPKM

TPQSLLPSQTGLSPYLRFGCLSTRLFFYQLNDLYRKIKKAVPPLSLHGQVLWREFFYCAATKNPNFDKMIGNPICVQVPW

DKNPEALAKWANGQTGFPWIDAIMTQLREEGWIHHLARHAVACFLTRGDLWISWEEGMKVFEELLLDADWSVNAGMWMWL

SCSSFFQQFFHCYCPVRFGRKADPNGDYIRKYLPVLKNMPTKYIHEPWNCPESVQRAAKCTIGVDYPLPMLNHSVVSKHN

IKRMKQVYLQLRNFRQPGIPPAAPLQEPIMEKKKIEENSYEENIFATPSQTFKGNMKNTK

>Halyomorpha_hal_PL-lik_XP_014272968.1 XP_014272968.1

MTNPFKKLKTESSGDSGFEEFLKGIENERIQNGKSVEHFGYNKNRIRFFSKSKEIPSWSKGILYWMTREERIQDNWSLLY

AQKVALKHKLPLHICFYLRRTFMNAPIRHFKFLLKGLEETSKESKKLNIPFHMFISDEGENKVLDFILENKFGYVVIDFS

PLRIARTWAENLKKTLPDDVPLVQVDGHNIVPCWVASDKLEYGARTIRNKLKNKFTEFLTPFPTVTDHPYPGELKAKEID

WGAAEASLEVDRTIEEVSWAKPGYLNGMKMLHEFCQKRLSKFAQKRNDPLGNALSNLSPWFHFGQISVQRCILVVESFKS

KYKESVESFCEEAIIRRELSDNFCYYNPNYDNINGAYDWARKTLEEHRKDKREWLYTQEELEKSLTHDDLWNSTQIQLVK

EGKIHGFLRMYWAKKILEWTESPEKALEIAIYLNDKYSLDGRDPNGFVGCMWSICGIHDQGWKERPVFGKIRYMNYKGCE

RKFNVSAFVARYGGKVHKKKK

>Harmonia_axy_CRY-1-_XP_045471055.1 XP_045471055.1

MNENERADLHSGNYSIGMASKEKDKHTVHWFRKGLRFHDNPALQAGLRNAKTFRCVFVLDPWFAGSSNVGINKWRFLLQC

LDDLDRSLKKFNSRLFVVRGQPADALPKLFKAWGTTCLSFEEDPEPFGQVRDNNIIALCNELGITVVQRVSHTLYDLQTI

IDKNGGMAPITYHQFLDVLSSMGTPDEPIPEPFKSDTRDDLKTPFMNNHDYEFGVPSLEQLGFDTLALPPPVWIGGESEA

LKRLERHLDRKAWVASFGMPKMTPQSLLSSRTGLSPYLRFGCLSTRLFYYRLTDLYRKIKNARPPLSLHGQLLWREFFYC

AATKNPNFDKMLGNPICVQIPWDHNAEALAKWANGQTGFPWIDAIMTQLRQEGWIHHLARHAVACFLTRGDLWISWEEGM

KVFEELLLDADWSVNAGTWMWLSCSSFFQQFSHCYCPVKFGRKADPNGDYIRKYISVLRNMPIKYIHDPWTAPDTVQKAA

KCVIGKDYPLPMVNHALVSRINVQRMKQVYRQLKNYQNIENSDEYGTQLSGNCKSSNQFVHT

>Harmonia_axy_PL-lik_XP_045482644.1 XP_045482644.1

MAPTILKDLAKEIFAKNIVKSRSETGKSIQEFKFKKDRCRLLSKYEDLREDSKGIIYWMSRESRVNDNWALLFAQKLCFK

YEVPLHVCFFLDDFKELYPTTRQTGFLRKGLDHVKKDLEKLNIPFYLLKNSPLELVDIIKTNDIGCLVCDFFPLRIITNW

QKKLQESLPSNVAIVQVDAHNIVPCWVASDKLERAARSIRPKILKQLPTYLTEFPQVSKQKETKELIIKSELKWSKGDKL

EIEHVEEITWAEPGEEGGLEMLRTFLLERLQYYGISSNEPSKKHQSNLSPWLHFGQISAQRIALEVSKLKKTWTTQCERF

LEEAIIRKEICDNFCLYNSNYDSLQGADKWAQDTLNLHRDDKRDYLYTAEQFEQCLTHDPIWNSAQFQLVTEGKLQGYMR

MYWCKKILEWTESPEKAIETALWLNDKYSIDGSDPNGFVGVMWSVCGIHDQGFKERPVFGKIRFMVDYSLSRKYDIKTYC

AKYRPNAKVSSNITKYLQSSKSKTEDGKGQGKTEQKDTKKTDIKDSKKLKAENHTSEKKRNIKDESDGGSKKKRKI

>Harpegnathos_sal_CRY-1__XP_019696624.2 XP_019696624.2

MTGSSNNKMGQGVTSVRGDGRKHTVHWFRKGLRLHDNPSLREGLAGASTFRCVFVLDPWFAGSTNVGINKWRFLLQCLED

LDCSLRKLNSRLFVIRGQPADALPKLFKEWGTTNLTFEEDPEPFGRVRDHNISALCKELGISVVQRVSHTLYKLDEIIEK

NGGKPPLTYHQFQNVVASMEPPEPPVLTVTSACIGSAYTPLKDDHDDHYGVPTLEELGFDTEGLLPPVWVGGESEALARL

ERHLERKAWVASFGRPKMTPQSLLPSQTGLSPYLRFGCLSTRLFYYQLTDLYKKIKKAMPPLSLHGQLLWREFFYCAATK

NPNFDRMQGNPICVQIPWDKNVEALAKWANGQTGFPWIDAIMTQLREEGWIHHLARHAVACFLTRGDLWISWEEGMKVFD

ELLLDADWSVNAGMWMWLSCSSFFQQFFHCYCPVRFGRKADPNGDYIRRYLPVLKNFPTRYIHEPWNAPLSIQHAAKCIV

GKEYSLPMVNHSKSSRINIERMKQVYQQLNKYRGNGE

>Helicoverpa_arm_ADN94465.2_cry_2 ADN94465.2

MSAAAETLPAPNARKHTPPATAAAPQPPSARRTNGKHIVHWFRKGLRLHDNPALREGLLDAATFRCVFIIDPWFASSSNV

GINKWRFLLQCLEDLDSSLRKLNSRLFVVRGQPADALPKLFREWGTTALTFEEDPEPYGRVRDHNIMSKCREVGITVTSR

VSHTLYKLDQIIERNGGKAPLTYHQFQALIASMPPPPPAEAPISAQMLNGATTPLNDDHDDRFGVPTLEELGFETEGLKP

PVWIGGESEALARLERHLERKAWVASFGRPKMTPQSLLASQTGLSPYLRFGCLSTRLFYYQLTELYKRVKRVRPPLSLHG

QILWREFFYCAATRNPNFDRMEGNPICVQIPWEKNQEALAKWASGQTGFPWIDAIMIQLREEGWIHHLARHAVACFLTRG

DLWISWEEGMKVFDELLLDADWSVNAGMWMWLSCSSFFQQFFHCYCPVRFGRKTDPNGDFIRRYIPALKNMPTRYIHEPW

VAPEAVQQSARCIIGRDYPMPMVDHSKASQVNIERIKQVYAQLAKYKPQGTLNPNAVQRPNVMQSSPSPNSIITSINQSN

YLCSQTPDPQPSPQIIPYKDNDVVFQKPMNHRSMKPSFKQVVIVQKKQNTNVIQTVSNCTPQSKENYAVNGQMDNSYKTP

INESLQSAKQENMTLKT

>Helicoverpa_arm_XP_021184238.1_cry-1_X1 XP_021184238.1

MLGGSVLWFRHGLRLHDNPSLHSALEEKGFPFFPIFIFDGETAGTKLVGYNRMRYLLEALDDLDSQFKKFGGRLIMLKGK

PNVVFRRLWEEFGIRKLCFEQDCEPVWRARDDSVKSACKEIGVVCREHVSHTLWEPETVIKANGGIPPLTYQMFLHTVAT

IGDPPRPVPNIDFTGVKFGSLPECFYQEFTVYDKTPKPEDLGVFLENEDIRMIRWVGGETTALKQMQQRLSVEYETFLRG

SYLPTHGNPDLLGPPISLSPALRFGCLSVRSFYWAVQDLFRQVHQGRLTTNSASHFITGQLIWREYFYTMSVNNPNYGQM

AGNPICLDIPWKNPEGDELQRWVEGRTGFPFVDAAMRQLRTEGWLHHAARNTVASFLTRGTLWLSWEHGLNHFLKYLLDA

DWSVCAGNWMWVSSSAFEALLDSGECACPVRLGQRLDPSGEYVRRYVPELARMPVEYIYEPWKAPIDVQERATCVIGKDY

PAPVVNHLVAAQRNKNAMKWLSRTVADRLQKDSWLDIVGELRHMLQKAPPHCCPSSEDEIRQFMWLNE

>Helicoverpa_arm_XP_021185587.1_photolyase XP_021185587.1

MRFPYLPSLIRTVNMASAPKKIKLSQTKLTSKSSSENKESNSNVEEFMKKLQDKREDTAKSILEYKFNKKRVRIISLEQM

VPDKCEGVVYWMSRDSRVQDNWAFLFAQKLALKNEVPLHVCFCLIAKYLDASVRQFDFLVKGLEKVAADCKKLNISFHLL

EGSGAEVLPQWVVKHNIGAVVCDFNPLRVPLGWLDGVKKKLKKGVPLIQVDAHNIVPCWVASDKQEYSARTIRNKINSKL

DEYLTEFPPVIKHPYTAKFEPEPIDWDEAIVSREADKNVGPVEWARPGYDNAIKMLKSFLDQRLKVFATKRNDPTMNALS

NLSPWFHFGQISVQRVALCVQQQKSKCTESVNSFLEEAIVRRELADNFCFYCEHYDSIKGASNWAQKTLDDHRKDKRTHI

YTLEQLANSETHDDLWNSAQIQLVKEGKMHGFLRMYWAKKILEWTPSVFGKIRYMNYDGCKRKFDIKAFIARYGGKVHKY

VPKK

>Helicoverpa_arm_XP_021188646.1_cry-1 XP_021188646.1

MTKVPSVIHWFRLDLRIHDNLALRNAINEAENRKHHLRPVYFIDPDIKSKVGINRLRFLVQSLQDLNENLKKLNTRLYII

RGNAVEELPKLFKKWQVKYLTSQVDIDPIYVKQDEIIDKIAEKSDIFIVRRVQHTVYDVHSVLKKNNGSVPLTYQKFLSL

VQDVQVKECIEITKAVSDDCKPKDFDSKQYDVPNLDELDIDETALQPLKYPGGETEGVKRLHMYMAKRDWVCKFEKPNSS

PNSIEPSTTVLSPYISHGCLSAKLFYHKLQEALSGRKHSEPPVSLLGQLMWREFYYTAGAGTENFDKMVGNPVCTQIPWG

KNDEHLKAWAEGRTGYPFVDAIMRQLKQEGWIHHLARHMVACFLTRGDLWISWEEGAKVFEDYLLDYDWSLNAGNWMWLS

ASAFFYKFFRVYSPVAFGKKTDKEGLYIRKYVPELKKYPTAFIYEPWKAPKNVQTTAGCIIGKDYPKRIVDHDTIHKENC

QKMSVAYKLNKERKALKRPLT

>Helicoverpa_zea_CRY-1_XP_047020760.1 XP_047020760.1

MTKVPSVIHWFRLDLRIHDNLALRNAINEAENRKHHLRPVYFLDPDIKGKVGINRLRFLVQSLQDLNENLKKLNTRLYII

RGNAVEELPKLFKKWQVKHLTSQVDIDPIYVKQDEIIDKIAEKSDIFIVRRVQHTVYDVHSVLKKNNSSVPLTYQKFLSL

VQDVQVKECIEITKEVSDDCKPKDFDSKQYDVPNLNELDIDESALQPLKYPGGETEGVKRLHMYMAKREWVCKFEKPNSS

PNSIEPSTTVLSPYISHGCLSAKLFYHKLQEALSGRKHSEPPVSLLGQLMWREFYYTAGAGTENFDKMVGNPVCTQIPWA

KNDEHLKAWAEGRTGYPFVDAIMRQLKQEGWIHHLARHMVACFLTRGDLWISWEEGAKVFEDYLLDYDWSLNAGNWMWLS

ASAFFYKFFRVYSPVAFGKKTDKEGLYIRKYVPELKKYPTAFIYEPWKAPKNVQTTAGCIIGKDYPKRIVDHDTIHKENC

QKMSVAYKLNKERKALKRPLT

>Helicoverpa_zea_CRY-1__XP_047023933.1 XP_047023933.1

MLGGSVLWFRHGLRLHDNPSLHSALEEKGFPFFPIFIFDGETAGTKLVGYNRMRYLLEALDDLDSQFKKFGGRLIMLKGK

PNVVFRRLWEEFGIRKLCFEQDCEPVWRARDDSVKSACKEIGVVCREHVSHTLWEPETVIKANGGIPPLTYQMFLHTVAT

IGDPPRPVPNIDFTGVKFGSLPECFYQEFTVYDKTPKPEDLGVFLENEDIRMIRWVGGETTALKQMQQRLSVEYETFLRG

SYLPTHGNPDLLGPPISLSPALRFGCLSVRSFYWAVQDLFRQVHQGRLTTNSASHFITGQLIWREYFYTMSVNNPNYGQM

AGNPICLDIPWKNPEGDELQRWVEGRTGFPFVDAAMRQLRTEGWLHHAARNTVASFLTRGTLWLSWEHGLNHFLKYLLDA

DWSVCAGNWMWVSSSAFEALLDSGECACPVRLGQRLDPSGEYVRRYVPELARMPVEYIYEPWKAPIDVQERATCVIGKDY

PAPVVNHLLAAQRNKNAMKELRHMLQKAPPHCCPSSEDEIRQFMWLNE

>Helicoverpa_zea_CRY-1-_XP_047029359.1 XP_047029359.1

MSAAAETLPAPNARTHAPPATAAAPQPPSARRTNGKHIVHWFRKGLRLHDNPALREGLLDAATFRCVFIIDPWFASSSNV

GINKWRFLLQCLEDLDSSLRKLNSRLFVVRGQPADALPKLFREWGTTALTFEEDPEPYGRVRDHNIMSKCREVGITVTSR

VSHTLYKLDQIIERNGGKAPLTYHQFQALIASMPPPPPAEAPISAQMLNGATTPLNDDHDDRFGVPTLEELGFETEGLKP

PVWIGGESEALARLERHLERKAWVASFGRPKMTPQSLLASQTGLSPYLRFGCLSTRLFYYQLTELYKRVKRVRPPLSLHG

QILWREFFYCAATRNPNFDRMEGNPICVQIPWEKNQEALAKWASGQTGFPWIDAIMIQLREEGWIHHLARHAVACFLTRG

DLWISWEEGMKVFDELLLDADWSVNAGMWMWLSCSSFFQQFFHCYCPVRFGRKTDPNGDFIRRYIPALKNMPTRYIHEPW

VAPEAVQQSARCIIGRDYPMPMVDHSKASQVNIERIKQVYAQLAKYKPQGTLNPNAVQRPNVMQSSPSPNSIITSINQSN

YLCSQTPDPQPSPQIIPYKDNDVVFQKPMNHRSMKPSFKQVVIVQKKQNTNVIQTVSNCTPQSKENYAVNGQMDNSYKTP

INESLQTAKQEKYDFKNLVINNYVQDYSNNQEIFQNEQQNRDNIYEQETLKINNFSYEKQKFYLSNYTENGLRIAAVHND

TPAPFVAALNHDSTMTFNRENKSDTGKDKANESTCLHPMSINNDGTITNESRQNNPSNECGYNSSNENQK

>Helicoverpa_zea_PL_XP_047025090.1 XP_047025090.1

MRFPYLPSLIRTVNMASAPKKIKLSQTKLTSKSASENKESNSNVEEFMKKLQDKREDTAKSILEYKFNKKRVRIISLEQM

VPDKCEGVVYWMSRDSRVQDNWAFLFAQKLALKNEVPLHVCFCLIAKYLDASVRQFDFLVKGLEKVAADCKKLNISFHLL

EGSGAEVLPQWVVKHNIGAVVCDFNPLRVPLGWLDGVKKKLKKDVPLIQVDAHNIVPCWVASDKQEYSARTIRNKINSKL

DEYLTEFPPVIKHPYSAKFEPEPIDWDEAIVSREADKNVGPVEWARPGYDNAIKMLKSFLDQRLKVFATKRNDPTMDALS

NLSPWFHFGQISVQRVALCVQQQKSKCTESVNSFLEEAIVRRELADNFCFYCEHYDSIKGASNWAQKTLDDHRKDKRTHI

YTLEQLANSETHDDLWNSAQIQLVKEGKMHGFLRMYWAKKILEWTPSPEDALKYAIYLNDHYSIDGRDPSGYVGCMWSIC

GIHDQGWAERAVFGKIRYMNYDGCKRKFDIKAFIARYGGKVHKYVPKK

>Hermetia_ill_CAD7078538.1_unnamed_pr CAD7078538.1

MYVKIETERLNFIRLNQAKLRSEEYIHLRDAIDTEGNTANIGRLIILPATYIGSPRHMHEYAQDAMTYIRHCGRPDLFMT

FTCNPKWIEIVQLLLPGQTSSDRPITARVFRQKLRSLMNYIVKQRVLGDTRCWMYSIEWQKRGLPHAHILIWLVERIQTD

QIDDIITAEIPDHEADPDLHDVVITNMIHGPCGAINTESSCMVDGNFYLTDILQISRFHILHAMKRSATGVTKTPSNSSQ

KSAKPNNQDGFSPSTESGFLKTIEESRVKTASSVLEFHFQKKRVRVLTEATEVKESAGSILYWMSRDCRVQDNWAFLFAQ

KLALKNRLPLHVCFCLVPKFLDATIRHYKFMLKGLQEVCQECKDLDINFHLLLGSGGEKIPEFVKEMEVGAVVCDFSPLR

VPLKWLDDVKNNLQEDVPLCQVDAHNIVPVWVTSDKQEYAARTIRNKINGKLDVYLTEFPPVIKHPFVDREYNDVDWPGA

FNSLECNREVEEVDWAQPGYKAACRVLETFCSSRLKLFNSKRNDPTVNALSNLAPWFHFGHISVQRCVLAVRDYKSKYPE

SVAAFCEEAIVRRELSDNFCFYNEHYDSLKGLASWAEKTLSDHRKDKRTYIYTTDELENAKTHDDLWNSAQIQLVKEAKI

HGFLRMYWAKKILEWTESPEKALQVAIYLNDKYSLDGRDPNGYVGCMWSIGGIHDQGWREREIFGKIRYMNYDGCKRKFD

VPAFVARYGGKVYSKKK

>Hermetia_ill_XP_037903861.1_LOW_QUALIT XP_037903861.1

MSTTNAGKQTIVHWFRKGQRIHDNPPLKKACALVNANPEKYVLRPIFILDPGIPRWMTVGANRFRFLQESLVQFNDNLKA

INSRLYVVRGTPSDVFPRLFEEWRVKVLTFESDIEPYAKKRDAEVRKLADECDVEVIVENSHTIYDPYKVLMLNSGRPII

NYRSFQAVIAKLPAAEPVPTPDPIKCSPPLKDFIEEKNGVCYDVPSLEQIGVDVNSLGPSKFPGGETEALSRMEHHLKNT

EWICSFEEPNTFPNSLEPSTTVLSPYLKFGCLSSRLFYKXLTEILAKNKKHSKPPVSLVGQLLWREFYYSAAAGEPNFDK

MEGNRICYQIPWTSNPEYLEAWTYGRTGYPFIDAIMRQLRQEGWIHHLARHAVACFLTRGDLWISWEEGQKVFEELLLDA

DWALNAGNWMWLSASAFFYQYFRVYSPVVFGKKTDKDGKYIRKYVPELKNYPASLIYEPWKATVAQQKLYGCVVGKDYPK

RIVVHEEVYKKNLEKMSKAYKVNKELLAKGKEETGYGVSSKASGVKRKYEEDEDEC

>Hermetia_ill_XP_037923019.1_cry1-like_ XP_037923019.1

MSAPQVERTNPNIEYNNVRPINGERHTVHWFRKGLRLHDNPALREGLIGATTFRCVFVIDPWFAGSSNVGINKWRFLLQC

LEDLDRSLRQLNSRLFVIRGQPADALPKLFKEWGTTCLTFEEDPEPFGKVRDKNVIEMCRELKIEVIVAVSHTLYKLDTI

IERNAGRAPLTYHQFQAIIAEMKPPAKPEEAINSENIGNVYTPMSEDHDDKYNVPTLYELGFDTEGLKPPVWVGGETEAL

ARLERHLERKAWVASFGRPKMTPQSLLASQTGLSPYLRFGCLSTRLFYHQLSGKFQNLYKKIKKGNPPLSLHGQLLWREF

FYCAATRNPNFDKMEGNPICVQIPWDKNCEALAKWANGQTGFPWIDAIMTQLREEGWIHHLARHAVACFLTRGDLWISWE

EGMKVFEELLLDADWSVNAGMWMWLSCSSFFQQFFHCYCPVRFGRKADPNGDYIRRYLPVLKNFPTKYIHEPWNAPESVQ

RAAKCIIGKEYSLPMVNHAVASRTNMERLKQVYQQIAKYRPQDDESSMKGGSAIPGMLAAARAQCGTTMNIVSSPSPTTV

ITNINNSENNYICQQQQQAPNSGHVNLVTNNHQQTARSRYIITKREYDFSKQESLTVTSTSGYIQHPEQPPHQKSGYDKS

ILGENFQYNENIKNQQERNNQHYNRMTISDLNNEQNIKSEGLTADVLTYRKQYINHSVVVHYGYNSNECTNELSYDNYRN

EYPGSNHDITLQIYHQQQQHHQQQQLQQQHQMQHQPQHDSLNNLSNVHQQLQQQMKTEANEIKAPNFKCDLDENSK

>Hermetia_ill_XP_037923127.1_cry1 XP_037923127.1

MYNNSCDGNTVGPSVLWFRHGLRLHDNPALLAALENKHKGSQFLPIFIFDSESAGTKLIGYNRMRFLLTALADLDQQFKA

QGGQLYFLQGKPSEIFRRLWEECAISTICYEQDCEPIWRERDSSVENLCHDIGVNCIEKISHTLWNPQAVIQANGGIPPL

TYEMFLHTVETLGLPSRPVPNPNWNEVKFFKLPEKMLKEFQAFSKPPNPEDFDVYFENCDSDVTEAWIGGETKALENLRY

RLKVEENAFQRGFYLPNHTHPDILGPSRSLSPHLRYGCLSIRRFYWDLHDLFEEVKRMNGAHSPSGPHITGQLIWREYFY

TMSVNNINYAQMKNNDICLNIPWAVPKGDEFERWKAGKTGFPLIDAAMRQLLSEGWLHHILRNTVASFLTRGALWLSWEL

GLQHFLKYLIDADWSVCAGNWMWVSSSAFEKLLDSSLCTCPLSLARRLDPNGDYIKRYIPELRCLPKEYIHEPWNTPLEV

QEACGCVIGVNYPEPMLDLQTASMKNAQAMRFLRDTLIAGGAPKEGPPHCRPSNEHEIRQFFWLVE

>Heterotrigona_ita_unname_CAD1473210.1 CAD1473210.1

MTGSRSSEINPEVTLHGEGGKHTVHWFRKGLRLHDNPSLREGLVGASTFRCVFVLDPWFAGSTNVGINKWRFLLQCLEDL

DCSLRKLNSRLFVIRGQPADALPKLFKEWGTTNLTFEEDPEPFGRVRDHNISALCKELGISVVQRVSHTLYKLDEIIERN

GGKPPLTYHQFQTVVASMDPPEPPVPTVTSVCVGSAYTPLKEDHDDHYGVPTLEELGFDTEGLLPPVWVGGESEALARLE

RHLERKAWVASFGRPKMTPQSLLPSQTGLSPYLRFGCLSTRLFYYQLTDLYKKIKKAVPPLSLHGQLLWREFFYCAATKN

PNFDRMQGNPICVQIPWDKNVEALAKWANGQTGFPWIDAIMTQLREEGWIHHLARHAVACFLTRGDLWISWEEGMKVFDE

LLLDADWSVNAGMWMWLSCSSFFQQFFHCYCPVRFGRKADPNGDYIRRYLPVLKNFPTRYIHEPWNAPLSVQRAAKCIVG

KDYSLPMVNHSKSSRINIERMKQVYQQLNKYRGNGLLNALPPPSVKETEEEKKKTKQSPPPPENQSKMETLVKGTQHQQQ

HHQHQ

>Heterotrigona_ita_unname_CAD1474615.1 CAD1474615.1

TSESIMTFNFNKKRIRRLNKLNDVKENCKGILYWMLRDFRIQDNWALLFAQKTALKNNVALHVCFCIMPSFLNASIRYYK

FLLKGLLEIEKECKQLKINFHLLYGEPNMSILKFVKMYNMGAVIVDFYPLKLPMYWVDNVQKNLPKDIPICQADAHNIVP

CWHASSKQEFAAKTIRNKINVKLEEFLTEFPPVIEHPYLTKENFERNNWDIALQDVDADKSVNEITWAEPGYIGGIKELE

NFIQNRLQKYGDERNNPLSNVTSNLSPWFHFGMISVQRCILEIKEYKRLYKKSVESFMEEAIIRRELSDNFCFYNEKYDL

VEGAYPWAIETLNKHRKDKRKYVYSLSQLENFKTHDDLWNACQNQMVTVGKMHAFLRMYWAKKILEWTETPEIALEWANY

LNNKYSIDGCDPNGYVGCMWSICGIHDHGWSERDIFGKIRYMNYEGCKRKFNVAEFVRKWGKKEDN

>Holotrichia_obl_CRY_UTD45278.1 UTD45278.1

MSGNLGLGSAGQRAEGRGAGQEKHTVHWFRKGLRLHDNPSLREGLKGATTFRCVFVLDPWFAGSSNVGINKWRFLLQCLE

DLDRSLRKLNSRLFVIRGQPADALPKLFKEWGTTVLTFEEDPEPFGRVRDHNITALCKELGITVIQRVSHTLYQLQQIID

RNCGRAPLTYHQFLAIIACMGPPPMAELPVTQQTLNGATTPINDDHDDKYGVPTLEELGFDTEGLLPPVWQGGESEALAR

LERHLERKAWVASFGRPKMTPQSLLPSQTGLSPYLRFGCLSTRLFYYQLTDLYKKIKKTFPPLSLHGQLLWREFFYCAAT

KNPNFDKMLGNPICVQIPWDKNAEALAKWASGQTGFPWIDAIMTQLREEGWIHHLARHAVACFLTRGDLWISWEEGMKVF

EELLLDADWSVNAGMWMWLSCSSFFQQFFHCYCPVKFGRKADPNGDYIRKYVPILKNMPVQYIHEPWTAPVSVQRAAKCI

IGKDYPLPMVNHAFASKINIQRMKQVYQQLAKYRTLENESCKISENYKDGYQPQVVTVGNSDKINDH

>Homalodisca_vit_CRY-1_XP_046660097.1 XP_046660097.1

MAELPGQKVGVHWFRHGLRFHDNPALLELLQSCQVFVPIFIFDGFTGFSGAKTVSYNRIRFLLECLLDLNNQLAARGGRL

YVCAGDPPKVLHKLKEELGQFKLSFDEDYEPIWHQRDLAVRQWCQNEGIEFIERGSHTLWDPRTVIQTNGGVPPLTYQMF

VHTMKVLGPPPRPVSDIDWSCVSFGKLENFGSDNIKLFPGIPAAEELGISQESGLGERVVVWEGGENRALRQLQDRVIVE

AEAFKRGLYLPNQAQPDLLAPPTSLSAALSAGCLSVRRFYWEIHDLYKDIHQGLQPASENITGQLIWREYFYTMSVNNPS

YGQVTGNPICLGISWGQGGGNEEAVERWRQGQTGYPFIDAAMRQLRQEGWIHHIARNAVACFLTRGDLWVSWEAGLAHFL

HQLIDADWSVCAGNWLWVSSSAFEQLLDCSQCVCPVNYGRRLDPHGLYVKRYVPEVQHLPVQYIYEPWKASLEVQEKSNC

IIGKDYPERIVNHQQASQKNRQMMIRIRNSLVENRPQHCCPSSVEEVRQFMWLPEKFTDHLCESVHT

>Homalodisca_vit_CRY-1-_XP_046667864.1 XP_046667864.1

MMKDHPKHTVHWFRKGLRLHDNPSLRAGLKGATTFRCIFILDPYFAGSSNEGINKWRFLLQCLEDLDNSLRKLGSRLFVI

RGQPTEALPKLFEEWKTTNLTFDYDPEPFGRKRDRHVREMCSSSGISVTSYVSHTLYNLDDIIAKNYNQPPLTYHRFQEV

VELLGRPPSAVGDIDPSFIGSAYTPLCDNHNERYGVPSLEELGFETEQLESPVWQGGETEALARLERHLQRKAWVASFAK

PKMSPSSLLASPCGLSPYLRFGCLSTRTFYHQLTQLYKSIKKDDPPLSLHGQLLWREFFYCAATNNPKFDKMIGNPICVQ

IPWDKNEEALAKWAKGKTGFPWIDAIMVQLRQEGWIHHLSRHAVACFLTRGDLWISWEEGMKVFEDLLLDADWSVNAGSW

MWHSNSSFFQQFFSCYCPVRFGRKADPNGEFIRKYLPVLKNFPTEHIHDPWTASEDVQKASNCVIGRDYPVPMVNHSEIS

AINSQRMRQVFLQLKKYRGPGNGYSVPTGRGLFTFNNPLRIKAEYNMDLSSNKENVFCFNEDDNSNNALYQ

>Homalodisca_vit_CRY-2_XP_046676545.1 XP_046676545.1

MPPPSSVHWFRKGLRIHDNPALISALEKEGPVFYQLRPLFILDPDIKKFLRVGPNRWRFLQQSLEDLDNNLRKIGSRLYV

LRGSPENVLREIFTEWNVKRLTFEVDIEPYALKRDTVITKLAEDAGVEVVKKVSHTIYNTELIIAKNMGKAPLTIQKWIS

VAESLPPPPKALAAPTRVPQEARAPLEEGKYLVPTLEELGVDPDELGPVLYPGGETEALRRMEEHIARKEYICKFEKPNT

SPNSLEPSTTVLSPYLKFGCLSARLFYYKLKEVVSGRPHSKPPVSLIGQMYWREFYYTVASTTPNFDKMVGNPVCIQVPW

NKNPQYLEAWTHGKTGYPFIDAIMRQLRQEGWIHHLARHAVACFLTRGDMWQSWEDGQKVFEELLLDADWALNAGNWMWL

SASAFFSQFYRVYSPVAFGKKTDKLGDYIRKYVPEVRKLPPEYIYEPWEAPLSVQKQAKCVVGTDYPRRVVIHEEVYKNN

IGRMSAAYKQTKESKVAAEKSSKRPSSQNDSSPNKKAKTKTLKDFFKK

>Homalodisca_vit_PL-lik_XP_046671859.1 XP_046671859.1

MGSEEPSPKKLKLSGDVSKTDKSDYVRILADERKKAAESVLEFKFNKKRVKILSSAQDVPDWAEGVVYWMFRDERVQDNW

AFLFAQKLALKNEIPLHICFCVKPKFLDATIRHYKFLFKGLEEVASECKSLNIEFHLLIGNGEEVLPGFVEKNKIGAVVV

DFMPLRDCLSWAQTLKKSLPKEVPLIQVDAHNIVPCWTASDKLEYGARTIRGKITRQLPEFLTQFPPVVKHPFSGKLKAD

KIDWEAAEKSLQMDLSVAPVTGFVPGYKAGISVLQEFCNKRIKLFGSKRNDPTVNALSNLSPYFHFGQISVQRAILCVKK

LGSSHKESVDAFVEEAVIRRELSDNFCYYNKKYDSIEGAYDWAKKTLNDHKKDKRTYVYTRSELEESKTHDDLWNSAQIQ

LVREGKMHGFLRMYWAKKILEWTASPEEALASAIYLNDRYSMDGRDPSGYVGCMWSICGIHDQGWREREIFGKIRYMNYE

GCKRKFNVSAFVARYGGKVHKYIKK

>Hyposmocoma_kah_CRY-1_XP_026324719.1 XP_026324719.1

MQGGSAMWFRHGLRLHDNPALHDALRDLAGAPFFPVFVFDGETAGTKLVGYNRMRYLLEALEDLNRQFQRSGGKLIMVKG

QPEAVFRRLWEEFGIRRLCFEQDCEPVWRARDERVARACRDAGVECREHVSHTLWAPDAVIGANGGIPPLTYQMFLHTVA

TIGDPPRPVADADLRGVTFGALPRSFYEEFTVFDKTPKPEDLGVFLEDEDVRMIRWVGGEGAALKQMDDRLVVERETFCR

GSYLPTHGSPDLLGPPVSLSPALRFGCLSVRKFYWALQDLFQQVHQGRLSGAHFITGQLIWREYFYTMSVNNPNYGQIAG

NPICLDIPWKSPEGDELERWRAGRTGFPFVDAAMRQLRAEGWLHHVVRNAVASFLTRGTLWLSWEHGLRHFLKYLLDADW

SVCAGNWMWVSSSAFEALLDSGACACPVRLGRRLDPSGEYVRRYVPELARLPARFVYEPWRAPLAEQQRAQCVVGRDYPA

PLADPAAAGAANARAMRRLRDALQRAPPHCCPSDADEVRRFMWLHDEPQPLAAASD

>Hyposmocoma_kah_CRY-1_XP_026328818.1 XP_026328818.1

MSKVPSVIHWFRLDLRIHDNLALRNAINEAENRKHLLRPIYVLDPNIKSKIGVNRLRFLIQSLQDLDTNLRKLNSRLYIL

KGNPVELLPKFFDQWQVKFLTTQVDIDPEIVDQEEAVEKIAEEKDVFIIKRVQHTVYDVYSVLKKNNGAVPLTYQKFMSL

VNDIQVKEAKDISKVVSDYCKPTDEDLDAYDVPKLEEFVDESTLTPCKYPGGETEAIKRLNNYMAKKQWVCKFEKPNTSP

NSIEPSTTVLSPYLSHGCLSPKLFYHKLKEAESGMKHSDPPVSLMGQLMWREFYYTAGAGTKNFDKMVGNPICMQIPWVK

KEEYIKAWAEGKTGYPFVDAIMRQLKQEGWIHHLARHMVACFLTRGDLWVSWEEGAKIFEDYLLDYDWSLNAGNWMWLSA

SAFFYKYFRVYSPVAFGKKTDKEGLYIRKYVPELKKYPTAFIYEPWKAPKSIQNTAGCIIGEGYPHRIVDHEKIHKENMQ

KMSSAYRMNKEKKAMKRSIS

>Hyposmocoma_kah_CRY-1-_XP_026324181.1 XP_026324181.1

MSAAPETLLVAAVTTDRSIETATPAPPLRSPVGKHLVHWFRKGLRLHDNPAFREGLKTAVTFRCIFIIDPWFASSSNVGI

NKWRFLLQCLEDLDRSLRKLNSRLFVVRGQPADALPKLFREWGTTALTFEEDPEPYGRVRDYNITTKCREVGITVISKVS

HTLYKLDTIIDLNGGKAPLTYHQFQALIASMPPPPPAEDTISAQSLNGASTPISDDHDERFGVPTLEELGFEVEGLKPPV

WIGGETEALVRLERHLERKAWVASFGRPKMTPQSLLASQTGLSPYLRFGCLSTRLFYYQLTELYKRIKRVRPPLSLHGQI

LWREFFYCAATRNPNFDRMEGNPICVQIPWEKNSEALAKWANGQTGFPWIDAIMIQLRDEGWIHHLARHAVACFLTRGDL

WISWEEGMKVFDELLLDADWSVNAGMWMWLSCSSFFQQFFHCYCPVRFGRKTDPNGDFIRKYIPALRNMPTRYIHEPWVA

PESVQQSARCLIGSDYPVPMVDHAKASQVNIERIKQVYAQLAKYKPQGSLNPQPILRPNVVQSSPSPTSIIASINQSNYL

CSQASDSQSTSNSQIIPNKDDVFQHPGKTNTAGLDKQIPFKQVYIIQKTKDLNTVTENITENQPSQPSYTIMNGLSDVSY

KTRQNIENQPNDKQENYDLKNLAINSNVPKYSNIQIHQQLTKTEILSSQLGKDDNFQENKVNFFMPATPENIMRISNLSH

VFVNDQLCQDYSRKGDLECGEEKVDTKHLTMTLPDRPFTNDTDNKEP

>Hyposmocoma_kah_PL_XP_026319839.1 XP_026319839.1

MARIHYCVTVVGNLVYKRNLFHFYAKMTSPAKKPKLTDVAASNSAESNSSVEEFMKKLQQTREDTAESILKFKFNKNRVR

IVSQEQMVPDSCEGIVYWMSRDSRVQDNWAFLFAQKLALKNKVPLHVCFCIIAKYLDASVRQFHFLLKGLEKVAADCKKL

NISFHLLEGSGAEALPEWIVEHNIGAVVCDFNPLRTPMSWLEGLKEKLKKDVPLIQVDAHNIVPCWVASDKQEYSARTIR

NKINSKLAEYLTEFPPVIKHPYISTFEPEPIDWDKAIETREADKTVGPVDWAKPGYDEAVKMLKSFIEKRLKIFATKRND

PTQDALSNLSPWFHFGQISVQRVALCVQEYKSKHTESVNAFLEEAIVRRELADNFCFYCEFYDSLKGASDWAKKTLDTHR

KDKRTHIYTLDQLSKAETHDDLWNSAQIQLVKEGKMHGFLRMYWCKKILEWTESPEDALKYAIYLNDHYSIDGRDPNGYV

GCMWSICGIHDQGWAERAVFGKIRYMNYEGCKRKFDIKTFIARYGGKVHKYTPKK

>Ignelater_lum_hypoth_KAF2881718.1 KAF2881718.1

MSVGVGNVGQASKEPEKHVIHWFRKGLRFHDNPALREGLKGAKTFRCVFILDPWFAGSSNVGINKWRFLLQCLEDLDRSL

RKMNSRLFVIRGQPADVLPKLFKEWGTTLLTFEEDPEPFGRVRDHNILTICREMGITVVQKVSHTLYYLQHIIDRNGGKA

PLTYQQFQTIIASMDPPPKAESPVTAKTLNGTVTPLTEDHDDKYGVPTLEELGFDTEGLLPPVWQGGESEALARLERHLE

RKAWVASFGRPKMTPQSLLASQTGLSPYLRFGCLSTRLFYYQLTDLYKKIKKAFPPLSLHGQLLWREFFYCAATKNANFD

KMLGNPICVQIPWDKNAEALAKWANGQTGFPWIDAIMTQLREEGWIHHLARHAVACFLTRGDLWLSWEEGMKVFEELLLD

ADWSVNAGMWMWLSCSSFFQQFFHCYCPVKFGRKSDPNGDYIRKYLPVLKNMPTQYIHEPWTAPESVQRASKCIIGKDYP

LPIVNHALASRTNIQRMKQVYQQLAKYRNIDHCNISQKYKECFQPSIAVAIGSVINSQQNNKRRRGDYT

>Iphiclides_pod_unname_CAH2037079.1 CAH2037079.1

MLGGSVLWFRHGLRLHDNPALHRALEDKNQPFFPVFIFDGETAGGIREGESPDGTKVVGYNRMRYLLEALEDLDNQFKKH

GGRLLMIKGKPDIIFRRLWEEFGIRKICFEQDCEPVWRPRDDAVKAVCREVGMACCEFVSHTLWEPDTVIRVNGGIPPLT

YQMFLHTVSTIGDPPRPVADVDLRSVNFGVLPERFYSEFTVFNKVPKPEDLGVCPEEADMRMIRWVGGETTALRQMQQRL

SVEYATFCRGSYMPTHGSPDLLGPPISLSPALRFGCLSVRKFYWALQDLFNQVHQGRIPATQLVTGQLIWREYFYTMSVN

NPNYGQMAGNPICLDIPWKNPEGDELKRWKEGRTGFPFIDAAMRQLRSEGWLHHALRNTVASFLTRGTLWLSWEHGLEHF

LKYLLDADWSVCAGNWMWVSSSAFEALLDSSECACPVLLGRRLEPSGEYVRRYVPELARMPDNYIYEPWKAPIEVQERAK

CIIGRDYPAPVVDHVEASQRNRNALKELRRILEKAPPHCCPSSEDEIRQFMWLGDDAQSDISSY

>Iphiclides_pod_unname_CAH2050796.1 CAH2050796.1

MSRLSCTTVKGKSWPYEQYYTGERDAEKIKCVKGENHWTGAKGLFYSNIRWGPGIESHVDVRQNVGLWLGNKLIRLAWKP

PSHNLILDLMTTNAGRACVNAHRNLMETSSEAIDESNEVIELLIKYGSKAKSAKASWGKLYPKNLTDLTSPVFRHQLFDQ

NYYGSRNIDKLIEVEKMPERIQSFDNNKTYFAWNNNEIIKHMMKHAFIHGKQLENKINIGNILSESRKKLKQPAKHELDC

RTLLMASYLGDTESVVHLVTNEDIHPDVTDLQGNSVLMYATCGDQTELIHFLVEAGASVNNFNDSCCTALGVALIRLICQ

VKGISVNDMAQAFTPETALSTDSSIQNVCEWNFGTDNNTLQNKNNGPTRSASKLLKSQVSHKRIKSLPSLKEQSLKTNPD

TPGKVPDQGFVTGNDDLNESVQQYTIVKNKYTARVALDYSAAGNGSPISYVFEVGDVTNNIIDNEVEEQKKTPEKNPKKV

TSKALKEQVNGKVKLTNGLRTQSKENPQELRDTEKIFTDSYERILLTINQLLLDGADPSLVRCPQPALFMAVTSGCSKLV

KQLIDHGANVNEFYPHVLGYSVLDIAISYPLTNENLKVINVLLENGADTQHRLPYNNHNSNESMIPGPTLLHAVLAKTAD

SEIEEEIRRHLLELLLTHNCDSEEQFKGRSAIDVAMSKGADIFNVFIGHPKVNLNAIINQSNQNVLTKMFTLSYFKTLES

KHRLEILTNLLRHGADPLQTCQNDEEKFYNIIVYAKKFLHGCENVEIKHSPIAARKQSENKIKKNGKSNDDKGAKQKTVI

GDVDDYKQALELKLALKNEVPLHVCFCLIAKYLDASVRQFHFLIKGLEKVAAECKKLNISFHLLEGSGADALPQWIIDHK

IGAVVCDFNPLRTPMSWVEGAKKKFKKDIPLIQVDTHNVVPCWVASDKQEYSARTIRNKINSKLDEYLTQFPPVIKHPYT

SKFEPKPIDWDKAIETREADKTVGPVEWASPGYDEAVKTLKTFLEKRLKIFASKRNDPTQDALSNLSPWFHFGQISVQRV

ALCVQKYKKQYTESVNAYLEEAIVRSELADNFCFYCEHYDSIKGASNWAQKTLDDHRKDKRTHIYTLEELAKAETHDELW

NSAQLQMVKEGKMHGFLRMYWCKKILEWTPSPEDALKYAIYLNDHYSIDGRDPNGYVGCMWSICGIHDQGWAERAVFGKV

RYMNYEGCKRKFHVNAFVARYGGKKHKYIAKK

>Iphiclides_pod_unname_CAH2068605.1 CAH2068605.1

MSKLSLKNIFEKLYEGHVGALEMSAAAQTLYAASVRAQASAVPEPAPSAFSHARGKHTVHWFRKGLRLHDNPSLREGLTD

AVTFRCIFIIDPWFASSSNVGINKWRFLLECLEDLDSSLRKLNSRLFVVRGQPNDALPRLFREWGTTALSFEEDPEPYGR

VRDHNITTKCRDIGVTVISRVSHTLYKLDKIIERNGGKAPLTYHQFQALVAGMPPPPPAEPPISAQTLNGAFTPLGEDHE

ERFGVPTLEELGFDTEDLKSPVWIGGESEALMRLERHLERKAWVASFGRPKMTPQSLLASPTGLSPYLRFGCLSPRLFYY

QLTALYKRVKGVQPPLSLHGQILWREFFYCAATRNPNFDRMVGNPICVQIPWVKNQDALSKWANGQTGFPWIDSIMIQLR

EEGWIHHLARHAVACFLTRGDLWISWEEGMKVFDELLLDADWSVNAGTWMWLSCSSFFQQFFHCYCPVRFGRKTDPNGDF

IRRYIPVLRNMPTRYIHEPWVAPEAVQQSAGCVVGRDYPLPMIDHTKASQVNIERIKQVYAQLAKYKPQSSLNPQAVARP

NITQSSPSPTSIIKSINQSNYLCSRTPDSQLTLTPSQQPPFKTGTNISLRPGNSERDTKQQYTHIVIVEQGQNTNMSSET

PQGLPTHDKKGQPEISNDLNPIKQNYDLKNLIIKNSDPQNSQQAFHGQPCKIDKLAQPVQIDNYASDKSEFHMQLYKDNE

TQERNTNTMSDLYVPNQPNRETINESVTENNENLQNMVISTNNLNSDQSNKEDPPDN

>Ischnura_eleg__XP_046383944.1 XP_046383944.1

MNPAAIQFIKTENVSTSAGQGQISDILLKLDRDRNKVASSAAAYDFNLKRVRLMSETEKIPDRMQGVVYWMSRDARVQDN

WAMLFSQRIALKHRVGLHVCFCLVPKFLDATIRHFKFLLGGLRVVESDLKKLNIQFHLLRGKADDVLPEFVEKNSIGAVV

TDFSPLRTPLSWVKSFKEKLNPSIPFWQVDAHNIVPMWVTSNELEYAAYTIRGKIKHHLPEFLTHFPPVISHPYPGTVKA

EPVDWAAAEASLQVDQSVKEVSWAVPGTSAGLNYLQSFVANRLQLYATKRNEPTVKAQSNLSPWIHFGQISIQRCVLEVQ

EKRPKCPESVDGWVEQAVIRRELADNFCYFNPNYDNLNGADQWAKDTLNNHRKDKREYVYSLAQFEAARTHDDLWNASQI

QMMREGKMHGFLRMYWAKKILEWSESPDDALSISIYLNDRYNLDGRDPNGYVGIMWSICGIHDHGFPERPVLGRIRWMSY

EGCKRKFDVMSFVSRYGAKKYRYEGKK

>Ischnura_eleg__XP_046393096.1 XP_046393096.1

MGNDNPEKHTVHWFRKGLRLHDNPSLREGLKGATTFRCVFILDPWFAGSSNVGINKWRFLLQCLEDLDRSLKKLNSRLFV

IRGQPADALPKLFKEWGTTFLTFEEDPEPFGRVRDQNIIAMCKENGIEVCSKVSHTLYKLENIISKNAGKPPLTYHQFQK

VVASMDRPECAAPPITAPFVGSAYTPVLDDHDDKYGVPTLEELGFDTEGLLPPVWQGGESEALARLERHLERKAWVASFG

RPKMTPQSLLASQTGLSPYLRFGCLSTRLFYYQLTDLYQKIKKARPPLSLHGQILWREFFYCAATQNPNFDKMAGNPICV

QIPWDKNQEALAKWANGQTGFPWIDAIMAQLREEGWIHHIARHAVACFLTRGDLWISWEEGMKVFEELLLDADWSVNAGM

WMWLSCSSFFQQFFHCYCPVRFGRKADPNGDFIRRYIPALKNFPTKYIHEPWTAPESVQKAAKCIIGKDYSVPMVNHAGA

SRVNMERMKQVYQQLSKYRGAGSEHRPSAKECFVGLLATVPIAPSAWTSQSSKETKENRPATQNTKNDVNAQKSLSTSPK

LIISSDIASREA

>Ischnura_eleg__XP_046403167.1 XP_046403167.1

MVGSAIHWFRKGLRIHDNPALLAVTEKVDGENCIIRPVFILDPWFVKNMRVGPNRWRFLQQSLMDLHESLKKMGSRLYVL

KGKPEEVLPIAFRVWNVKLLTFELDIEPYARERDERIEALAKDAGVRVMQKISHTLFDTEAVIKANLGRPPLTYQKLISV

VSGLGAPPNPVTMPDKFPKECFVGNEKADVHGEIPGWPEFNVPSLKDLGVKEEELGPNLHHGGETEALKRLSKNIAKKEW

ICKFEKPNTSPNSLQPSTTVLSPYLKFGCLSSRLFYYKLKEVTKGQPRVSQPPVSLIGQLLWREFYYVVGSATPNFDRMK

GNPVCCQVPWNNNPEHLEAWTMGRTGYPFIDAIMTQLRLEGWIHHLARHAVACFLTRGDLWISWEEGQKVFEELLLDADW

ALNAGNWMWLSASAFFHQFFRVYSPVAFGKKTDKLGDYIRKYVPKLAKFPPQYIYEPWDAPLSVQKAAGCIIGVDYPKRI

VIHESVSKANIQRMSAAYKSNKERKAGEEDEGAPKKSKGNTKKRASDSDPLSSSANKKSKTKGNKSIANYFEK

>Ischnura_eleg__XP_046405998.1 XP_046405998.1

MSELDFPKKTERGSALIWFRHGLRLHDNPALHEALKDCSQFYAVFIFDGESAGTKLVGYNRMKFLLESLADLNDQLERRG

GKLYLLKGTPSTIFKRLHEEVGLAKICFEQDCEPIWKERDNSVKTTCEELGMKCVEKVSHTLWDPKEIIQTNGGKPPLTY

QLFIETVPLIGEPRRPVQDVEWTNIKFGILPDHLLDEFEAFSSIPSPEAFGLEPEVKDKSPVVRWIGGETHALHQLTKRL

QVEENAFKNGVYLPNQANPDLIGPPTSLSAALRFGCLSVRRFYWEIHDVWMKIRGKDCPPSDSITGQLIWREYFYTMSVD

NPFYAEMERNPICLTIPWVDDDENLQKWAKGETGYPFIDASMRQLLLEGWIPHAARNAVACFLTRGDLWISWEKGLSVFL

YHLLDADWSVCAGNWMWVSSSAFERLLDCTMCVSPVSYGRRLDPWGTYIKRYVPELSGFPPEYIHEPWKAPIVVQESAGC

IIGHDYPERIVDHAVASKRNQKYMEAIRNSLMSDFPAPHCCPSNEDETRHFLMMDDAEIDHVH

>Ischnura_ele_PL_XP_046383389.1 XP_046383389.1

MAKPPPAKKSKIEHFLSGGSSSSTYEPEENIWKKLEKDRAQVASSITDFKFNKKRVKVLTKVGDVPDNSQGIVYWMSRDM

RVQDNWAMLFAQKLALKNKVGLHVCFCLVPKFLEATIRHYKFLLGGLREVETELKALDIQFHLLLGEAASVLPNFVKKHS

IGAVVCDFSPLRVPKSWVEAVKKCLPSDVPLCQVDAHNIVPVWEASNKLEYAARTIRGKITKQLPDYLTQFPPVIKHPVP

VKVKAEPVDWDEAEKSLEVDRSVDEVKWAKPGTRAGLEMLQSFISKRLKVFGSKRNDPTVNALSNLSPWFHFGHISVQRC

ILEVKKNRSKYADSVDAFVEEAVIRRELSDNFCFYNPHYDSIKGTNDWAKKTLEDHKKDKRAYVYTRAQLDEAKTHDDLW

NSAQIQLVKEGKMHGFLRMYWAKKILEWTESPEVALADAIYLNDRYSLDGRDPNGYVGCMWSVCGIHDQGWAERPIFGKI

RCMTYDGCKRKFDVAAFVARHGGKKHKYEGKK

>Ladona_fulv_hypoth_KAG8236888.1 KAG8236888.1

MTTSKIQVVKAQGTSLHGTNVSLPDAPFDLRRVKLLSSVNQVPSYIKGVVYWMSRDARVQDNWAMLFAQRLALKNRVALH

VCYCLLPKFLDATIRHFRFLLSGLQEVESELKSLNIEFHLLRGAADEVLCDFVKKNYIGAVVTDFSPLRTPLSWVDSFKR

NLSQDIPFWQVDAHNIVPLWAASDQLESAAYTIRGKIKGQLKEFLTPFPPVLKHPFSSSEKAELVDWELAESSLQVDRSV

GEVAWAQPGTAAAHKMLQHFISNGLRLYGTMRNEPNVKAQSNLSPWIHFGHISVQRCALTIQELRSKYPESVDSWLEQAI

IRRELADNFCYYNPNYDNFNGADGWARKTLDDH

>Ladona_fulv__KAG8231453.1 KAG8231453.1

MRSRNPNENKVTLLWFRHGLRLHDNPALLEALRCSKEFYAVFIFDGESAGTRVIGHNRLKFLLESLSDINSQLQSHGGKL

YLLNGKPFEIFKTLKEEVGLQKICFEQDCEPIWKERDDSVKDLCQEIGIECVEEVSHTLWDPRVVIETNGGIPPLTYQMF

LHTVAAIGPPPRPVQDADWTGVNFGILPKKVVDKFEAFTSVPSPEEFGLVDNTKSKGPKVRWIGGESNALKNLSFRLEVE

NNAFCNGIYLPNQVNPDLIGPPTSQSAALSFGCLSVRKFYWAIHDLFNQVYGENLSNESITGQLIWREYFYTMSVDNPFY

AEMKRNPICLDIPWHEDQENLESWKQGKTGYPFIDAAMRQLVQEGWVHHAARNAVAFFLTRGDLWISWEHGLAFFLYYLI

DADWSVCAGNWMWVSSSAFEKLLDCSQCVSPVSYGRRLDPCGIYIKKYVPELSRFPIEYLHEPWKASLELQQSCGCIIGR

DYPERIVDHNVASLRNRKYMEDICLSLMISPPKHCCPSDEEETRRFLMITQEDVDHIH

>Ladona_fulv__KAG8231874.1 KAG8231874.1

MEAAETHDDLWNSAQIQLSKEGKMHGFLRMYWAKKILEWTESPEEALSIAIYLNDRFSLDGRDPNGFVGCMWSICGIHDQ

GWAERAVFGKIRYMNYEGCKRKFDVAAFVARYGGKKYPYKGKK

>Ladona_fulv__KAG8233196.1 KAG8233196.1

MAKGSVIHWYRKGLRVHDNPALLAATEKINGEYYEILPVFVLDPWIVKTMRVGPNRWRFLQQSLKNLDENLRKIGSRLYV

LRGKPEDVLPEAFKAWKVKAITFETDTEPYARERDERIETLAKKAGVSVIQKTSHTLYNPEAVLKANLGKPPLTYQKFLS

VMNGMGPPPKPVMMPKNIPVGCLGYDAMKDKGSKLPGGPEYGVPSLSDLGVSEESLGPSLFPGGETEALNRLSKFTAKKD

WICKFEKPNTSPNSLQPSTTVLSPYLKFGCLSPRTFYYKIQEVIKGQSRVSQPPVSLIGQLIWREFYYVVSSATPNFDKM

EGNPVCLQIPWDVNKEYLEAWSKGKTGYPFIDAIMTQLRSEGWIHHLARHAVACFLTRGDLWISWEEGQKVFEELLLDAD

WALNAGNWMWLSASAFFHQFFRVYSPVAFGKKTDKLGDYIRKYVPQLAKFPPQYIYEPWEAPLSVQKAAGCIIGDDYPKR

IVIHETISKINIKRMSEAYKKNKESKIKAEDDVEQ

>Ladona_fulv__KAG8235753.1 KAG8235753.1

MGSEKTEKHTVHWFRKGLRLHDNPSLREGLKGATTFRCVFILDPWFAGSSNVGINKWRFLLQCLEDLDRSLRKLNSRLFV

IRGQPADALPKLFKEWGTTFLTFEEDPEPFGRVRDQNITAMCKEIGIEVCSKVSHTLYKLEDIIKKNNGKPPLTYHQFQM

VVASMDPPEPAAPPIVKSFVGSAYTPISDDHDEKYGVPTLEELGFDTEGLLPPVWQGGETEALARLERHLERKAWVASFG

RPKMTPQSLLASQTGLSPYLRFGCLSTRLFYYQLTDLYRKIKKAPPPLSLHGQILWREFFYCAATHNSNFDKMAGNPICV

QIPWDKNQEALAKWANGQTGFPWIDAIMTQLREEGWIHHLARHAVACFLTRGDLWISWEEGMKVGSHDKVFEELLLDADW

SVNAGMWMWLSCSSFFQQFFHCYCPVRFGRKADPNGDYIRRYLPVLKNFPTKYIHEPWMAPESVQRASKCLIGKDYSVPM

IKKAPPPLSLHGQILWREFFYCAATHNSNFDKMAGNPICVQIPWDKNQEALAKWANGQTGFPWIDAIMTQLREEGWIHHL

ARHAVACFLTRGDLWISWEEGMKVGSHDKVFEELLLDADWSVNAGMWMWLSCSSFFQQFFHCYCPVRFGRKADPNGDYI

>Lamprigera_yun_hypoth_KAF5281231.1 KAF5281231.1

MSAINECMDSASKGPEKHMVHWFRKGLRFHDNPALREGLKGASTFRCVFILDPWFAGSSNVGINKWRFLLQCLEDLDRSL

RKMNSRLFVVRGQPADALPKLFKEWGTTLLTFEEDPEPFGRVRDHNIITICQEMGITVTQKVSHTLYYLDHIINRNGGKA

PFTYHQFQAVIASIDPPPFAEPPISTADVAGIKTPLSEDHDEKYGVPTLEELGFDTEGLLPPVWQGGEGEALARLERHLE

RKAWVASFGRPKMTPQSLLASQTGLSPYLRFGCLSTRLFYYQLTNLYKKIKKTFPPLSLHGQLLWREFFYCAATNNTNFD

KMLGNPICVQIPWDKNAEALTKWANVRYLHSLYFMNSFYFKGQTGFSWIDAIMTQLREEGWIHHLARHAVACFLTRGDLW

LSWEEGMRVFEELLLDADWSVNAGMWMWLSCSSFFQQFFHYYCPVKFGRKADPNGDYIKKYLPALKNFPTQYIHEPWTAP

ENVQRASKCIIGKDYPLPIINHAVASRTNIQRMKQVYQKLIKYRNIAHCNVPQTFMYEKNLVSINSNKN

>Laodelphax_str_AVP27638.1_cry_1 AVP27638.1

MSDEMSSSVLWFRHGLRFHDNPALHAAIKKEDNFYPIFIFDGESAGTKVIGSNRMRFLLESLKDLDTQLQNVGQRLFVFK

GSPVKIFDWMAKSIKMKTLCFEQDCEPIWAERDNAVKNFCDGAGVKCVEKVSHTLWNPKEVIEANGGVPPLTYQMFLYTV

STIGNPPRPEADVDWSKVKFGKLPEDIPDDIFLYAGVPTLEDFNMHAYDGGERMVRWVGGETNAIANLRNRILVEEEAFR

CGFYLPNQANPDLVAPPTSQSAALRFGCLSVRRFYWTLHDLFNEIHEGKLTSNQNITGQLIWREYFYTMSVDNIHYGEMT

RNPICLDIPWMPKTNKLHNEFLQRWKEGMTGYPFIDAAMRQLLQEGWIHHVARNAVASFLTRGDLWLSWEAGLRHFMEHL

LDADWSVCAGNWMWVSSSAFEQLLDCSHCVCPVNYGRRLDPWGVYVKRYVPELRNFPVQFIYEPWKLPLEDQEKYNCVIG

RDYPERIVEHKVASQINRKKMEHIRDSLMSKVPHCCPSSAEEVRQFMWLPENCSDHVCVPN

>Laodelphax_str_AVP27639.1_cry_2 AVP27639.1

MTGEAVAKQQCGWSQDMVGGGGGRGGVQRGPEGGGGNKKQVGGQKHTVHWFRKGLRLHDNPSLREGLKNAATFRCIFILD

PWFTGASNVSINKWRFLLQCLEDLDNSLKKLNSRLFVVRGQPADVLPKIFKEWGTTNLTFEEDPEPFGRVRDQNVMAICK

EMGISVVSKVSHTLYRLDDIIEKNGGTAPLTYRKFQTVVASLDEPPQAEPTVTAKVVGAAVTPIADDHDEKFGVPSLDEL

GFDTEGLLPPVWQGGESEALARLERHLERKAWVASFGRPKMTPQSLLASQTGLSPYLRFGCLSTRLFYYQLSDLYKKIKK

AAPPPSLHGQLLWREFFYCAATNNSNFDRMVGNPICVQIPWTNNPEALAKWANGQTGFPWIDAIMHQLREEGWIHHLARH

AVACFLTRGDLWVSWEKGMVVFDELLLDADWSVNAGMWMWLSCSSFFQQFFHVYCPVRFGRLADPSGDFIRRYVPALKNF

PSAYIHEPWNCPIELQKSAKCIIGEDFPLPMVNHVQASRVNMERLKQVYSRLAEFRNNNMHLMRPLPSPSGPTTSNGVMT

MLMDNCEISVNGLKHERNNAKEAIDKGLFNTPFLPKHKDKLNQKRYIMHK

>Laodelphax_str_RZF47186.1_LSTR_LSTR0 RZF47186.1

MASKSDKSPPAKKQKLLDDVPSKSDTKSGSSLIKELKEKRNKTAASILQFKFNKNRVRILSEAKDVPDRAEGIVYWMFRD

ERVQDNWALLFAQKLALKNKIPLHVCFCLKPRFMEATIRHYKFLLKGLEEVAEDCKKLNIQFNFLIGDGERVLPGFVEKN

SIGAVVVDFMPLKGPMSWAEQLKKTLPKDVPLCQVDAHNIVPCWIASDKQEYGARTIRNKINNKLKEYLTEFPPVIKHTY

GTVKDEVIDWEGGEKTLEVDRSVEPVKWLTPGYSGAIQMLDSFINKRLKKFATKRNDPVVDALSNLSPYFHFGQISVQRA

ILTVREHRSKCPESVDAFCEEAIIRRELADNFCFYNKNYDNINGSFDWAKKTLNDHRKDKREYIYTKEELENAKTHDDLW

NSAELQLLKEGKIHGFLRMYWAKKILEWTTSPEEALEISIYLNDKYSMDGRDPNGFVGCMWSICGIHDQGWAERNVFGKI

RYMNYAGCKRKFDVAAFVARYGGKSYPYKKSK

>Lasius_nig_CRY_2_KMQ95506.1 KMQ95506.1

MNGYPDTRTLMERFLLQCLEDLDCSLKKLNSRLFVIRGQPADALPKLFKEWGTTNLTFEEDPEPFGRVRDHNITALCKEL

GISVVQKVSHTLYKLDEIIERNNGKPPLTYHQFQNVVAGMNSPEPPVSTVTAACIGNAYTPLKDDHDDHYGVPTLEELGF

DTEGLLPPVWVGGESEALARLERHLERKAWVASFGRPKMTPQSLLPSQTGLSPYLRFGCLSTRLFYYQLTDLYKKIKKAV

PPLSLHGQLLWREFFYCAATKNPNFDRMQGNPICVQIPWDKNVQALAKWANGQTGFPWIDAIMTQLREEGWIHHLARHAV

ACFLTRGDLWISWEEGMKVFDELLLDADWSVNAGMWMWLSCSSFFQQFFHCYCPVRFGRKADPNGDYIRRYLPVLKNFPT

RYIHEPWNAPLSIQHTAKCIIGKEYSLPMVNHSKNSRINIERMKQVYQQLNKYRGNGASFKGEKIGLLNALLASPTEVDE

EKRKQDSSNRKNEQKIETINNSAQQQQQQQQQQQQ

>Leguminivora_gly_CRY-1_XP_047986229.1 XP_047986229.1

MLRSIQIMARATPAVIHWFRLDLRVHDNLALRNAINEAENRKYYLRPIYIIDPDIKDKIGVNRLRFLVQSLQDLDDNLKK

LNTRLYIIKGKAEEKLPELFDKWQVKNLTLQLDIDPELARVDDIIEKIAEKKEIFVVKRVQHTVYDCNRVLTKNNGSVPM

TYQKFLSLASEQPVKACIEINKQISDSCRPLDHDSQEYDVPNLSDLGIDESSLCPCKYVGGETEGLKRLDMYMARKEWVC

KFEKPNSFPNSLEPSTTVLSPYISHGCVSAKLFYNKLKEVENGRRHTEPPVSLMGQLMWREFYYTAGTGTENFDKMVGNP

LCIQIPWVKNEAHLKAWAEGRTGFPFVDAIMRQLKQEGWIHHLARHMVACFLTRGDLWISWEEGAKVFEDYLLDYDWSLN

AGNWMWLSASAFFYKYFRVYSPVAFGKKTDKEGEYIKKYVPELAKYPEAFIYEPWKAPKEVQRRAGCVVGEGYPKRIVEH

DKIHKDNMAKMNAAYKANKEKKEKKSLKRKRTDD

>Leguminivora_gly_CRY-1__XP_047995227.1 XP_047995227.1

MMGGSVLWFRHGLRLHDNPALHSALEDNTVPFYPIFIFDGETAGTKLVGYNRMRYLLEALDDLDQQFRRHGGRLIMLKGQ

PNVVFRRLWEEFGIRKLCFEQDCEPVWRARDQSVKQACREIGVTCREHVSHTLWEPDTVIKANGGIPPLTYQMFLHTVAT

IGDPPRPVGDLDLSGVKFGTLPECFYSEFTVFDKTPKPEDFGVHLENEDIRMIRWVGGETTALKQMKHRLSVEYETFCRG

SYLPTHGSPDLLGPPISLSPALRFGCLSVRSFYWAVQDLYRQVHQGRLPSNQFVTGQLIWREYFYTMSVNNPQYGQMAGN

PICLDIPWKEPSGDELKSWIEGRTGFPFIDAAMRQLRIEGWLHHAVRNTVASFLTRGTLWLSWEHGLNHFLKYLLDADWS

VCAGNWMWVSSSAFEALLDSGECACPVRLGQRLDPSGEYVRRYVPELTNMPPLYIYEPWKAPIEVQERAKCIIGQHYPAP

VVNHLAAARRNRNAMQELRQMLQKAPPHCCPSSEEEIRQFMWLPDDILVQVNSA

>Leguminivora_gly_CRY-1-_XP_048006614.1 XP_048006614.1

MSSSATVTGAAPGRMAEPRPGKHTVHWFRKGLRLHDNPALREGLNGAVTLRCVFIIDPWFASSFNVGINKWRFLLQCLDD

LDSSLKKLNSRLFVVRGQPADALPKLFREWGTTVLTFEEDPEPYGRVRDHNIMSKCREVGITVLSRVSHTLFKLDKIIER

NGGKAPLTYHQFQALIASMPPPPAAEATITPQLFNGTVTPISDDHDERFGVPTLEELGFETEGLKPPVWIGGESEALARL

ERHLERKAWVASFGRPKMTPQSLLASQTGLSPYLRFGCLSTRLFYYQLTELYKRIKQVRPPLSLHGQILWREFFYCAATR

NPNFDRMEGNPICVQIPWDKNQDALAKWANGQTGFPWIDAIMIQLRDEGWIHHLARHAVACFLTRGDLWISWEEGMKVFD

ELLLDADWSVNAGMWMWLSCSSFFQQFFHCYCPVRFGRKSDPNGDFIRKYIPVLKKIPTQYIHEPWLAPEAVQVAAHCVV

GRDYPLPMVDHSKASKVNIERIKQVYAQLAKYSPHGNVLNPNSVPRPNAVLSSPSPSSIIASINQSNYLCSKSPDPSQNS

QTQQGKTFKEIPNYNDTNRALRPQKVGSPKPPPFKEIVIVPIVQKTNVNNENVAEGRAQ

>Leguminivora_gly_LOW_QU_XP_047985638.1 XP_047985638.1

MLMPRKWRGFVAVNKFIETAQLFHFARDFQQHTLKMASAAKKPKLSQPSISSSESKTSIDDFMKSLQAKRDETAKSILEY

KFNKKRLRIISQEQMVPDDCEGIVYWMSRDSRVQDNWAFLFAQKLALKNEVPLHVCFCLIAKYLDASVRQFDFLIKGLKT

VAEQCKELDISFHLLEGDGGDVLPQWVIDHKIGAVVCDFNPLRTPLGWLEKAKKGLKKDVPLIQVFHLSRXPCWVASDKQ

EYSARTIRNKINSKLDEYLTQFPPVIKHPYKSKFKPEPIDWEQAIESREADKSVGPVDWATPGYHAAVKMLRSFLDKRLP

IFATKRNDPTLDALSNLSPWFHFGQISVQRVALCVQEYKKKYTESVNAFLEEAIVRRELADNFCFYNEHYDSVKGAHSWA

AKTLDDHRKDKRSHIYTLEELSQAKTHDDLWNSAQIQLNKEGKMHGFLRMYWAKKILEWTPSPEDALKYAIYLNDHYSID

GRDPSGYVGCMWSICGTHDQGWAERAVFGKIRYMNYDGCKRKFTVPAFVARYGGKVHKYVPKK

>Leptidea_sin_CRY-1_XP_050681692.1 XP_050681692.1

MRCSIKYNWPLLVLNSQSMSVLGSVVHWFRLDLRVHDNLALRNAINEAENRKNFLRPIYIIEPDILKQIRGNRLRFLIQS

LHDLDANLRKLNTRLYIVRGKTSECLPELLKKWNVKYLTLQVDIDPFYIKQDEVVENFCDKNDIFVVKRVQHTVYNPQTV

LKKNNGNVPLTYQKFLSLVNDIQVKECIEIKKEISEDCKSSDFNSKSYDVPSLTEIGLDESSDFKYPGGESEGLKRLLTY

MAKKQWVCSFEKPNTSPNSIEPSTTVLSPYISHGCLSARLFYHKLKQVESEMRHSEPPVSLLGQLMWREFYYTAGTGTEN

FDRMVGNSLCTQIPWGNNKDHLKAWSEGKTGYPFVDAIMRQLTQEGWIHHLARHMVACFLTRGDLWVSWEEGAKVFEDLL

LDYDWSLNAGNWMWLSASAFFYKYFRVYSPVAFGKKTDKDGLYIKKYVPELKKFPSEYIYEPWKAPQSVQRAAGCIIGEN

YPKRIVDHDKVHKENMKKMSEAYKINKEKKALKRPRSD

>Leptidea_sin_CRY-1__XP_050684341.1 XP_050684341.1

MQKELDNMLGGSVLWFRHGLRLHDNPALLDAVSDKNEPFFPIFIFDGETAGTSLVGYNRMRYLLEALDDLGSQLRQHGGR

LHLVKGNPTDIIRRLREELGLKKICFEQDCEPIWRARDDRVRNLCREVGVVCREHVSHTLWEPDTILRHNGNIPPLTYQM

FLHTVSIIGDPPRPVSDVDLREVQFGALPDAFCKEFCVFDKTPKPEDLGVFLENEDIRMIRWVGGETAALKQMEQRLAVE

RETFFRGSYLPTHSSPDLLGPPVSLSPALRFGCLSVRKFYWAVQDLFIEVHKGRMSSAPFITGQLIWREYFYTMSVNNPH

YGQMAGNPICMRIPWREPKGDELQRWKEGRTGYPLVDAAMRQLRAEGWLHHVLRNTVASFLTRGTLWLCWEHGLQHFLKY

LLDADWSVCAGNWMWVSSSAFEVLLDSGDCASPVLLGRRLDPTGHYVRRYVPELERVPDIYIYEPWKMPLEVQRRARCVI

GRDYPAPIVNHEEAATRNRNTMNELRRIMNKAQPHCCPSSEEEARQFMWLNEDSVSVDGN

>Leptidea_sin_PL_XP_050681948.1 XP_050681948.1

MKRINRNLFKMATAAKKIKLSAPTSSKEKKLESNLGDFITSIQKKREETADSILNFKFNKKRVRIISQEQLVADQCEGII

YWMSRDSRVQDNWAFLFAQKLALKNEVPLHVCFCLIAKYLDASVRQFHFLIKGLEKVAEECNQLNISFHLLEGSGADALP

QWVVDHKIGAVVCDFNPLRVPMSWLEGVKKKLKKDVPLIQVDAHNVVPCWEASDKQEYSARTIRGKINSKLDEFLTEFPP

VIKHPYKSKFEPEPIDWEDAIESREADKSVAPIEWAGPGYLEAMKTLKSFLDTRLKIFASKRNDPTQNALSNLSPWFHFG

QISVQRVALCVQEFKSKYTESVNAYLEEAIVRRELADNFCFYCKHYDSVKGASNWAQKTLDDHRKDKRTHIYTLEQLCKA

ETHDDLWNSAQIQLVKEGKMHGFLRMYWCKKVLEWTPSPEDALKYAIYMNDHYSVDGRDPSGYTGCMWSICGVHDQGWAE

RSVFGKVRFMNYDGCKRKFDIKAFIARYGGKAHKYVPAK

>Leptidea_sin_unname_VVC94173.1 VVC94173.1

MSAAAQTLRQSSSRPVHPVHPHAPPCRTQSGKHTVHWFRKGLRIHDNPALREGLTDATTFRCVFIIDPWFASSSNGGINK

WRFLLQCLEDLDRSLRKLNSRLFVVRGQPADALPKLFREWGITTLTFEEDPEPYGRVRDHNISTKCREVGITVASRVSHT

LYNLDHIIDRNGGKAPLTYHQFQALIASMPPPPPAEKTISLQMLNGAITPISIDHDERFGVPTLQELGFDIEGLRPPVWI

GGEKEALSRLERHLERKAWVASFGRPKMTPQSLLASQTGLSPYLRFGCLSTRLFYYQLTELYKRIKRVRPPLSLHGQILW

REFFYCAATRNPNFDRMLGNPICVQIPWEKNQDALSKWASGRTGFPWIDSIMIQLREEGWIHHLARHAVACFLTRGDLWI

SWEEGMKVFDELLLDADWSVNAGMWMWLSCSSFFQQFFHCYCPVRFGRKTDPNGDFIRKYIPALRNMPTRYIHEPWMAPE

AIQESAHCVIGRDYPLPMIDHSKASEINIERIKQVYAQLAKYQPQGLINLNVQRPNGMQSSPSPTSIITSMNQSNFLCSQ

SSDHQNSSMSSVQPLKEEIFIRPVKNTKDNIEIAKQSLFKQVILFQQQAQNSRYVEPHPPSREHYIMNRGLNTAYKMQNK

ELEIADCEKNVMNPSIDNYTNSKLYILENSARVDTEKSYTTNKAGEFVKEEKIIDTTNSTVKPLNLMMNNEQFNEDSTQD

QNIKSKACK

>Leptinotarsa_dec_XP_023017756.1_cry-2-like XP_023017756.1

MSGSTGQTSTGQEKHTVHWFRKGLRLHDNPSLREGLTGAKTFRCVFVLDPWFAGSSSVGVNKWRFLLQCLEDLDRSLRKL

NSRLFVIRGQPADALPKLFKEWGTTVLTFEEDPEPFGKVRDHNITALCRELGITEFFYFSITHVVSGLNPPVWQGGESEA

LTRLERHLERKAWVASFGRPKMTPQSLLPSQTGLSPYLRFGCISTRLFYYQLTDLYKKIKKAFPPLSLHGQLLWREFFYC

AATKNPNFDKMHGNPICVQIPWDKNVEALAKWANGQTGFPWIDAIMTQLRQEGWIHHLARHAVACFLTRGDLWISWEEGM

KVFEELLLDADWSVNAGMWMWLSCSSFFQQFFHCYCPVKFGRKADPNGDYIRKYLPILNNMPLQYIHEPWLAPDSVQQAA

KCIVGKSYPLPMVNHTNASRINIQRMKQVYQQLANYKVMETARYPVNTGYSETFQRQPSAVTVGNPDNGQAA

>Leptinotarsa_dec_XP_023024919.1_photolyase XP_023024919.1

MASLKPRSSSGKVVLEKLTKELFLKNIVESRESQGGTDEFDFNKTRCRVLTDNETVKDKSNGILYWMYRDCRIQDNWAMV

FAQRLAIKQKVPLFVCFTVKDAHQQYPTMRHFKFLIEGLKLLKKECESLNIGFYLLNSAPKDLAKSIVDNNIGGVICDFS

PLKHPKKLQEVLLEHLPDDVPVVQVDAHNIVPVWKASDKQEGMAKFLRTKITKQLPEYLTGFPIIRRHKYSGKPNFKNEI

ESFDSAYSHYTPKWDVPEIKWGDGPGEKAGYSMLLDFILKNLRHYGATSNDPSKDNSSKLSPWINFGQISAQRCALEVKS

VDSIYKEQCDKYLEELIVRRELTDNYCFFNSNYDNINGAANWAKETLKLHSKDKRTWIYTREQLEKAETHDEMWNSAQLQ

AHHEGKIHNYMRMYWCKKILEWTESPEQAIEYGLWLNDTFCLDGTDPNGYVGVMWSICGVHDQGWREREIFGKIRYMVDY

SLRRKYNMDAYCARFGRKILGDSKTKAAIIPKEPKESKGKNETKKGKGIKRKAT

>Leptopilina_het_CRY-1__XP_043467129.1 XP_043467129.1

MTGSRSSDLGTGAIKIQKDGHKHTVHWFRKGLRLHDNPSFREGLIGASTFRCVFVLDPWFAGSTNVSINKWRFLLQCLED

LDRSLRKLNSRLFVIRGQPADALPKLFKEWGTTNLTFEEDPEPFGRVRDHNISAICKELGISVIHKASHTLYKLEEIIEK

NIGKTPLTYHQFQNVIASMDPPELPAPTVTLTCIGNAFTPLKEDHDDQYGVPTLEELGFDITGLLPPVWIGGESEALARL

ERHLERKAWVASFGRPKMTPQSLLASQTGLSPYLRFGCLSTRLFYYQLTDLYKKIKKTVPPLSLHGQLLWREFFYCAATN

NSNFDKMQGNPICVQIPWDKNVDALAKWANGQTGFPWIDAIMTQLREEGWIHHLARHAVACFLTRGDLWISWEEGMKVFD

ELLLDADWSINAGMWMWLSCSSFFQQFFHCYCPVRFGRKADPNGDYIRRYLPVLKNFPARYIHEPWNAPMCVQRAAKCII

GREYSHPMVNHTKSSRINIERMKNVYQHLNKYRHPEILISPLASHATANPSEEMNQQKNTEDNFSNDVKESETIY

>Leptopilina_het_PL_X1_XP_043466984.1 XP_043466984.1

MKIVNRAFASKFHNLFYQVCKEYSKMEKSKSAKKARTLSLMDKIKEERENTAASVLEFKFNKKRIRILTKLDEVAENCNG

VVYWMFRDARVQDNWAFLFAQKIALKNQVPLHVCFCILPKFLDATIRHYKFLLKNLQEVEEECKSLNINFSLLHGEPNSA

ILDFVLKYKMGAVITDFFPLRLPMFWVDDLKKKLPEDVPFCQVDAHNIVPCWVTSDKQEYAARTIRNKINSKLEEFLTPF

PPVIKHPYSSKQQLKDNDWKTALKDVKIDKTVDEILWATPGYEGGILELESFFKNRLKTYSSKRNDPVADAVSNLSPWFH

FGMISVQRCILEVQKYKKEYKPSVEAFMEEAIIRRELSDNFCFYNENYDSMKGAYQWAIDTLEAHRKDKREYLYDLKELE

HSQTHDDLWNAAQNQLVQEGKMHGFLRMYWAKKILEWTGTPEDALKWSIYLNDKYSMDGRDPNGYVGCMWSICGIHDQGW

KERPIFGKIRYMNYQGCQRKFDVAQFVSKWGGKVVHSTKKKGKK

>Linepithema_hum_PREDIC_XP_012217865.1 XP_012217865.1

MTGSSNNEMDQEVTTGVRSDGGKHTVHWFRKGLRLHDNPSLREGLAGASTFRCVFVLDPWFAGSTNVGINKWRFLLQCLE

DLDCSLRKLNSRLFVIRGQPADALPKLFKEWGTTNLTFEEDPEPFGRVRDHNISALCKELGISVVQRVSHTLYKLDEIIE

RNSGKSPLTYHQFQNVVASMDPPKPPVSTVTAACIGSAYTPLKDDHDDHYGVPTLDELGFDTEGLLPPVWVGGESEALAR

LERHLERKAWVASFGRPKMTPQSLLPSQTGLSPYLRFGCLSTRLFYYQLTDLYKKIKKAMPPLSLHGQLLWREFFYCAAT

KNPNFDRMQGNPICVQIPWDKNVEALAKWANGQTGYPWIDAIMTQLREEGWIHHLARHAVACFLTRGDLWISWEEGMKVF

DELLLDADWSVNAGMWMWLSCSSFFQQFFHCYCPVRFGRKADPNGDYIKRYLPVLKNFPARYIHEPWNAPLNIQHAAKCI

IGKEYSLPMVNHNKNSRINIERMKQVYQQLNKYRGNGTSFKGESIGLLKALLALPKKNDEEKQDSPNQKNEQKINYPTQQ

Q

>Lucilia_cup_CRY-1_XP_023306440.2 XP_023306440.2

MSINIIWFRHGLRLHDNPALLEAISDKDLGIELLPIFIFDGESAGTKSVGYNRLKFLLDSLKDIYDQLQNLSLSLGRLYV

LQGNPVQIFRRLHEQCGIRKLCFEQDCEPIWNRRDNAVKKLCHDLGITCLERISHTLWDPKKVIDTNGGIPPLTYQMFLH

TVQIIGLPPRPVPNPDWTNVKFHKLSEKLIMELKVYLEFPTPEDFNVYPDNLSYLAKVKWIGGETQALLHLHQRLKVEEN

AFKCGYYLPNQAKPNILESPKSMSAHLRFGCLSVRKFYWDVHDLFKNVQMQAEGLGMHMSGGAHITGQLIWREYFYTMSV

NNPNYDRMEDNEICLNIPWAQPNHEQIQRWTMGQTGFPLIDAAMRQLLAEGWLHHTLRNTVATFLTRGGLWQNWEHGLRY

FLKYLLDADWSVCAGNWMWVSSSAFERLLDSSLVTCPVALAKRLDPMGQYIKQYVPELSKVPKEFIHEPWRMPLNVQEQS

DCLIGVHYPNRLIDLSVASQRNMLAMRALRNSLIAEGAPDNGPPHCRPSNEEEVRNFFWLAD

>Lucilia_cup_CRY-2__XP_046812613.1 XP_046812613.1

MSEKRTLIHWFRKGLRVHDNPALKTVFDKAQQQPDKYCVRPIFILDPGIIEWLRVGANRWRFLQQSLEDLHQQLITLNSR

LYVVRGNPKNVFPRLFEEWQTELLTFEEDIEPYSLKRDKEIQELAKQFKVQVSTYCSHTVYNPHVVIQKNQGKAPLTYQK

FLSLVEKIKVPLPEDKPIKLTCSLPAKDTWELKDENCYDVPTLKDLVKKLEDLGPLKFPGGETEALKRLTASMADESWVA

AFEKPKTYPNSLEPSTTVLSPYLKFGCLSSRLFYQQLNAILKRQPKHSKPPVSLMGQLMWREFYYTAAAYEPNFDRMMGN

SFCLQIPWQTNDKHLEAWTFARTGYPFIDAIMRQLRQEGWIHHLARHAVACFLTRGDLWISWEEGQKVFEELLLDQDWAL

NAGNWMWLSASAFFHQYFRVYSPVAFGKKTDPTGSYIRKYIPELAKYPAGAIYEPWKVSLADQRKYDCILGTDYPHRIVN

HDIVHKENIKRMTAAYKVNREVKTGKPEDDDENSEEQVGKRKSNKTTSTTSAPKKRKKN

>Lucilia_cup_PL_X1_XP_023303202.2 XP_023303202.2

MKIIFTKLFSIKYNNLNKRLYTMKRAAATSSKSSKTVDSSKKSKNLNEFVQKTSTSKGSEDVNNFLLSLKEKRLKCAASI

VDFSFKKKRVRILSKTQEVREDCQGPVVYWMSRDVRVQDNWAFLYAQRLALKLQLPLVVVFCLVPKFLNATIRHYKFMLG

GLQEVAEECNDLNITFQLLMGPAKERVPEFVATHDVAAVVCDFAPLRVPLQWVEDVKEKLPDSVPFIQVDAHNIVPVWVA

SDKQEYGARTIRNKINTKLPEFLTEFPALIEHNIKFKTHLEKVDWKAAFDSLQCDMSVDEVANIKPGYKAACQQLQEFCE

QRLKRFSDKRNDPTQNALSGLSPWFHFGQISVQRCVLEVKEYKTKFKDSVEAFCEEAIVRRELADNFCYYNKNYDNLQGL

HAWAAKTLNDHRKDKRTPCYTLEEFEKSRTHDDLWNSAQLQLVNEGKMHGFLRMYWAKKILEWTESPEQALEYAILLNDK

YSLDGRDPNGYVGCMWSIGGIHDQGWAERAIFGKIRYMNYQGCKRKFDVNAFVARYGGKVYCKK

>Lucilia_ser_CRY-1_XP_037810294.1 XP_037810294.1

MSAFASNTLSCKDRKRGTLNECNTDNDFSTMSVNIIWFRHGLRLHDNPALLEAISDKDLGIELLPIFIFDGESAGTKSVG

YNRLKFLLDSLKDIYDQLQNLSLSLGRLYVLQGNPVQIFRRLHEQCGIRKLCFEQDCEPIWNRRDNAVKKLCHDLGITCL

ERISHTLWDPKKVIDTNGGIPPLTYQMFLHTVQIIGLPPRPVPSPDWTNVKFHKLSEKLIMELKVYLEFPTPEDFNVYPD

NLSYLAKVKWIGGETQALLHLHQRLKVEENAFKCGYYLPNQAKPNILESPKSMSAHLRFGCLSVRKFYWDVHDLFKNVQM

QAEGLGMHMSGGAHITGQLIWREYFYTMSVNNPNYDRMEDNEICLNIPWAQPNHEQIQRWTMGQTGFPLIDAAMRQLLAE

GWLHHTLRNTVATFLTRGGLWQNWEHGLRYFLKYLLDADWSVCAGNWMWVSSSAFERLLDSSLVTCPVALAKRLDPMGQY

IKQYVPELSKVPKEYIHEPWRMPLNVQEQSDCLIGVHYPDRLIDLSVASQRNMLAMRALRNSLIAEGAPDNGPPHCRPSN

EEEIRNFFWLAD

>Lucilia_ser_CRY-2_XP_037821650.1 XP_037821650.1

MSEKRSLIHWFRKGLRVHDNPALKTVFDKAQQQPEKYCVRPIFILDPGIIEWLRVGANRWRFLQQSLEDLHQQLTALNSR

LYVVRGNPKNVFPRLFDEWQTELLTFEEDIEPYSLKRDKEIQELAIKLKVKVSTYCSHTVYNPHVVIQKNQGKAPLTYQK

FLSLVEKIKVPLPEDKPIKLKCSLPAKDSWELEDENCYEVPTLKDLVKKPEDLGPLKFPGGETEALKRLTASLADESWVA

AFEKPKTHPNSLEPSTTVLSPYLKFGCLSSRLFYQQLKEILKRQPKHSKPPVSLMGQLMWREFYYTAAAYEPNFDRMMGN

SFCLQIPWQTNDKHLEAWSYARTGYPFIDAIMRQLRQEGWIHHLARHAVACFLTRGDLWISWEEGQKVFEELLLDQDWAL

NAGNWMWLSASAFFHQYFRVYSPVAFGKKTDPTGSYIRKYVPELAKYPAGAIYEPWKVSLADQRKYGCVLGTDYPHRIVN

HDIVHKENIKRMTAAYKVNREVKTGKQEDDENSEELVGKRKSNKTSSSSSAAKKRKKN

>Lucilia_ser_PL_XP_037822376.1 XP_037822376.1

MKIIFTRLFFIRYNYFSKGLYTMKRAAATSSKSSKTADSSKKSKNLNEFVQKTSTSKCKEDVNNFILSLQEKRLRCAASI

EDFSFKKKRVRILSKTQEVREDCQGPVVYWMSRDVRVQDNWAFLYAQRLALKLQLPLVVVFCLVPKFLNATIRHYKFMLG

GLQEVAEECNDLNITFQLLIGPAKERAPEFVATHDVAAVVCDFAPLRVPLQWVDDVKEKLPDSVPFIQVDAHNVVPVWVA

SDKQEYGARTIRNKINSKLPEFLTEFPALIKHNIKFKTQLEKVDWKAAFDSLECDMSVDEVENIKPGYKAACQQLLEFCA

QRLKKFSEKRNDPTQNALSGLSPWFHFGQISVQRCILEVKEYKTKFKDSVEAFCEEAIVRRELADNFCYYNRNYDNLQGL

HAWAAKTLNDHRKDKRSPCYTMEEFEKSRTHDDLWNSAQLQLVNEGKMHGFLRMYWAKKILEWTESPEQALEYAILLNDK

YSLDGRDPNGYVGCMWSIGGIHDQGWAERAIFGKIRYMNYQGCKRKFDVNAFVARYGGKVYSKK

>Mamestra_bra_antenn_AAY23345.1 AAY23345.1

MLGGSVLWFRHGLRLHDNPSLHSALEEKGFPFFPVFIFDGETAGTKVVGYNRMRYLLEALEDLDNQLKKHGGRLIMIKGK

PNVVFRRLWEEFGIRRLCFEQDCEPVWRARDDSVKSACKEIGVVCKEHVSHTLWEPDTVIKANGGIPPLTYQMFLHTVAT

IGDPPRPVSDIDFTGVKFGSLPESFYHEFTVYDKTPKPEDLGVFLENEDIRMIRWVGGETTALKQMQQRLAVEYETFLRG

SYLPTHGNPDLLGPPISLSPALRFGCLSVRSFYWSVQDLFRQVHQGRLATQSASHFITGQLIWREYFYTMSVNNPNYGQM

AGNPICLDIPWKEPEGDELQRWVEGRTGFPFVDAAMRQLRTEGWLHHAARNTVASFLTRGTLWLSWEHGLNHFLKYLLDA

DWSVCAGNWMWVSSSAFEALLDSGECACPVRLGQRLDPSGEYVRRYVPELARMPVQYIYEPWKAPIDIQERATCIIGKDY

PAPVVNHLVAAQRNKNAMKWLGRSVAGRLDKEKWFELVGELRHFLQKAPPHCCPSSEDEIRQFMWLNE

>Manduca_sex_CRY-1_XP_037298981.1 XP_037298981.1

MTKVPTVIHWFRLDLRIHDNLALRNAINEAENRKHYLRPIYVIDPNIKNRVGLNRLRFLLQSLQDLDMNLRNKNSRLYII

RGNAMEELPKLFEKWQVKNITCQVDIDPEVVQQDEFIEEIAEKKGIFINKRVQHTVYDVHRVLKNNNGAVPLTYQKFLSL

VNDLPVKECIELTKSVPDECKPPDFESNKYNIPSLDELDIDDTNLEPLKYPGGETEAIKRLNLYMSKKQWVCKFEKPNSS

PNSIEPSTTVLSPYISHGCLSAKLFYHKLKEVERGSQHTLPPVSLLGQLMWREFYYTAGAGTNNFDKMVGNPLCMQIPWG

KNEEHLKAWAEGRTGYPFVDAIMRQLKQEGWIHHLARHMVACFLTRGDLWISWEEGAKVFEEYLLDYDWSLNAGNWMWLS

ASAFFYKYFRVYSPVAFGKKTDKEGLYIKKYVPELKKFPSSFIYEPWKAPKSIQTAAGCVIGVDYPKRIVDHDKIHKENM

QKMNIAYKANREKKAVKRPRS

>Manduca_sex_CRY-1__XP_030026594.1 XP_030026594.1

MLGGSVLWFRHGLRLHDNPAMHSALQDRSVPFFPLFIFDGETAGTKLVGYNRMRYLLEALEDLDSQFKKFGGRLIMIKGK

PSHVIRRLWEEFGIRKVCFEQDCEPVWRPRDDSVKSICREIGVTCHEHVSHTLWEPDTVIRANGGIPPLTYQMFLHTVAT

IGDPPRPVGDLDLLGVKFSTLPECFYQEFTVFDKTPKPEDLGVFLENEDIRMIRWVGGETTALKQMQQRLAVEYDTFCKG

SYLPTHGNPDLLGPPISLSPALRFGCLSVRKFYWAVQDLFRQVHQGRLTSTHFITGQLIWREYFYTMSVNNPNYGQMADN

PICLDIPWKNPEGDELERWAEGRTGFPFVDAAMRQLRAEGWLHHAVRNTVASFLTRGTLWLSWEHGLNHFLKYLLDADWS

VCAGNWMWVSSSAFEALLDSGECACPVRLGQRLDPSGEYVRRYVPELARVPVEYIYEPWKAPLDVQERASCIIGKDYPAP

LVNHVVAAQRNRNAMKELRHILQKAPPHCCPSSEDEIRQFMWLNE

>Manduca_sex_LOW_QU_XP_037301546.1 XP_037301546.1

MSAAAESLPASSARTHAAGSQARRPPGKHIVHWFRKGLRLHDNPALREGLVDAVTFRCVFLIDPWFASSSNVGINKWRFL

LQCLEDLDKSLKKLNSRLFVVRGQPADALPKLFREWGTTALTFEEDPEPYGRVRDHNIMSKCREVGITVISRVSHTLYKL

DNIIERNGGKAPLTYHQFQALIASMPPPPAAEPPISAQTLNGAATPVADDHDDRFGVPTLEELGFETENLKPPIWIGGES

EALARLERHLERKAWVASFGRPKMTPQSLLASQTGLSPYLRFGCLSTRLFYYQLTELYKRIKRVRPPLSLHGQILWREFF

YCAATRNPNFDRMEGNPICVQIPWEKNQEALAKWANGQTGFPWIDAIMVQLREEGWIHHLARHAVACFLTRGDLWISWEE

GMKVFDELLLDADWSVNAGMWMWLSCSSFFQQFFHCYCPVRFGRKTDPNGDFIRRYIPALKNMPTRYIHEPWVAPESVQQ

SARCVIGSDYPMPMVDHCKASQINIERIKQVYAQLAKYKPQGTLNPQAVQRPNVMQSSPSPTSIIRSINQSNYLCSQTPD

PTQQSTNQNTYKDQSTVFQRPTKTFKSPDSPQFXNKLLVVQQTQNTDNPSENSDCTTRPSENAIVSDRQDIVYKSHQSRH

LQSGGKQENYDYKNYNIDHISGYDNQDMYQSQQINQNELYSQQDIKIDTYDYNKSKYYLSNYAENNVLVSSATPTFITPS

MNHQSNMNYNREGKNKSKNNKSSDAVFLHPLSVVSDRMYSGDNRQTQSSNECEYSSASDDNQK

>Manduca_sex_PL_XP_030022133.1 XP_030022133.1

MLIIRTESLKVVLKMASAAKKPKLSTSSLSVTAETGKNVEEFMKALQTKRETTADSILKYNFNKKRIRIISREQMVPDDC

EGIVYWMSRDSRVQDNWAFLFAQKLALKNEVPLHVCFCLIAKYLDASVRQFHFLVKGLEKVAADCKKLNISFHLLEGSGA

EVLPQWVIDHKIGAVVCDFNPLRVPLGWLEGVKKDLKKDVPLIQVDAHNVVPCWVASDKQEYSARTIRNKINSKLDEYLS

EFPPVIKHPYTSKFEPEPIDWDNAIETREADKTVGPVEWAKPGYDEALKMLKSFIDKRLKIYATKRNDPTQDALSNMSPW

FHFGQISVQRVALCVQEYKSKYTESVNAFLEEAIVRRELADNFCFYCEHYDSVKGASNWAQKTLDDHRKDKRSHIYTLET

LSKSETHDDLWNSAQLQLVKEGKMHGFLRMYWCKKILEWTPSPEDALKYAIYLNDHYSIDGRDPSGFVGCMWSICGIHDQ

GWAERAVFGKIRYMNYDGCKRKFDIKAFIARYGGKVHKYVPAKAGK

>Maniola_hyp_CRY-1_XP_034824901.1 XP_034824901.1

MTKSASVLHWFRLDLRLHDNLALRNAINEAENRKHILRPTYVLDPDIKNKVGRNRLRFLIQSLQDLDANLRKINSRLFII

KGNAVDCLPQLFEKWQVKFITLQVDIDPELVKQDEVIEKICEKKDIFMVKRVQHTVYDFNTTLRNNNGNVPLTYQKFLSL

VANTQVKDTIEITKQVSDDCKGPEFDSKDNDISLLNDLGFDEADFNECKYPGGETEGLKRLNTYMAKKQWVCNFEKPNTS

PNSIEPSTTVLSPYISHGCLSSKLFYHKLKEVESGMKHSSPPVSLLGQLMWREFYYTAGAGTKNFDKMVGNPVCTQIPWK

KNEEYLKAWAEGRTGYPFVDAIMRQLKQEGWIHHLARHMVACFLTRGDLWVSWEEGAKVFEDYLLDYDWSLNAGNWMWLS

ASAFFYKYYRVYSPIAFGKKTDKEGLYIRKHVPELKKYPSEFIYEPWKAPKSVQTAAGCIIGQDYPKRIVDHDKVHKDNI

QKMNAAYKLSKEKKALKRKR

>Maniola_hyp_CRY-1_XP_034830720.1 XP_034830720.1

MLGGSVLWFRHGLRLHDNPSLNTALEDKTVPFFPVFIFDGETAGTKVVGYSRMRYLLEALQDLDNQFKKYGGRLHMIKGK

PDVVFRRLWEEFGIRKICFEQDCEPIWRARDESVKSLCREIGVACRENVSHTLWDPDVIIRANGGIPPLTYQMFLHTVAI

IGNPPRPVDDVDLRGVNFVSLPDSFYKEFTVFDKAPKPEDLGIFLENEDIRMIRWVGGETEALVQMQSRLAVEYETFCRG

SYLPTHGNPDLLSRPISLSPALRFGCLSVRRFYWCLQDLFQQVHQGRLASGQFITGQLIWREYFYTMSVNNPQYAQMAGN

PICLNIPWKEPEGDELQRWKEGRTGFPFIDAAMRQLRTEGWLHHAVRNTVASFLTRGTLWLSWEHGLQHFLKYLLDADWS

VCAGNWMWVSSSAFEALLDSGECACPVRLGRRLEPSGHYVRRYVPELAKMPVEYIYEPWRAPLDVQQKADCVIGRDYPAP

VVDHRAAAHRNRSAMQELRRVLEKAPPHCCPSSEDEIRQFMWLGDESQMEFNTAS

>Maniola_hyp_CRY-1-_XP_034840818.1 XP_034840818.1

MLAAVETLPAPCLAPRLPAPARNPTGKHTVHWFRKGLRLHDNPALREGLVDAATFRCVFIIDPWFASSSNVGINKWRFLL

QCLEDLDSSLKKLNSRLFVVRGQPADALPKLFREWGTTALTFEEDPEPYGRVRDHNIMTKCREVGISVTSRVSHTLYKLD

NIIERNGGKAPLTYHQFQALIASMPPPPPAEVTITVQTLNGAVTPISDDHDDRFGVPSLEELGFETEGLKPPVWVGGENE

GLLRLERHLERKAWVASFGRPKMTPQSLLASQTGLSPYLRFGCLSTRLFYYQLTELYKRVKHVRPPLSLHGQILWREFFY

CAATKNPNFDRMEGNPICVQIPWEKNQDALAKWASAKTGFPWIDAIMIQLREEGWIHHLARHAVACFLTRGDLFISWEEG

MKVFDELLLDADWSVNAGMWMWLSCSSFFQQFFHCYCPVRFGRKTDPNGDFIRKYIPALKNMPTRYIHEPWQAPAAVQEA

ARCHVGRDYPMPIVDHHKASQANIERIKLVYTQLAKFKPAGSLNPQIVQRPNVMQSSPSPTSIIVNINQSNYLCSQTPDP

PQPTPEFKEDYVFLKPTRNNRIKADPNKQTPFKQIVIVQQDQKPSVQRQPPAEVSYISNNIQCPVKSESYDFKQLVINNS

TPNERDIIFVNKTTDEQEVYRQNVDLDEYTTSKPKLYFIDNGVTTHNDNAQKFSPQSYINDYNTKEKSQQNVDDTVHVHN

MEVDKDHTKADDKTTSDANK

>Maniola_jur_crypto_XP_045760482.1 XP_045760482.1

MLAAVETLPASGLAPRLLPPARNPTGKHTVHWFRKGLRLHDNPALREGLVDATTFRCVFIIDPWFASSSNVGINKWRFLL

QCLEDLDSSLKKLNSRLFVVRGQPADALPKLFREWGTTALTFEEDPEPYGRVRDHNIMTKCREVGISVTSRVSHTLYKLD

NIIERNGGKAPLTYHQFQALIASMPPPPPAEASITVQTLNGAVTPIADDHDDRFGVPSLEELGFETEGLKPPIWVGGENE

GLLRLERHLERKAWVASFGRPKMTPQSLLASQTGLSPYLRFGCLSTRLFYYQLTELYKRVKQVRPPLSLHGQILWREFFY

CAATKNPNFDRMEGNPICVQIPWEKNQDALAKWASGKTGFPWIDAIMIQLREEGWIHHLARHAVACFLTRGDLWISWEEG

MKVFDELLLDADWSVNAGMWMWLSCSSFFQQFFHCYCPVRFGRKTDPNGDFIRKYIPALKNMPTRYIHEPWQAPAAVQEA

ARCLVGRDYPMPIVDHHKASQTNIERIKQVYAQLAKFKPPGSLNPQIVQRPNVMQSSPSPTSIIVNINQSNYLCSQTPEP

PQNTPEFKEDYVFLKPRNNRIKVDPNKQTPFKQIVIIQQDQSQRVQRQPPAEVSYIINNMQRPVKSENYDFKKLVINNSI

PNERDTFVNETTDKQEAYEQNVNLDEYTTSKPKLYFIDNGVISHDDNAPNFIPQSYVNDYNIKEKSQQNIDDTTRVHNME

VDKDQTKADDKKTVGEK

>Maniola_jur_crypto_XP_045764552.1 XP_045764552.1

MTKAASVLHWFRLDLRLHDNLALRNAINEAENRKHILRPTYVLDPDIKNKVGRNRLKFLIQSLRDLDANLRKINSRLFII

KGRAVDCLPQLFEKWQVKFITLQVDIDPELVKQDEVIEKICEEKDIFMVKRVQHTVYDFNTTLRNNNGNVPLTYQKFLSL

VASTQVKDTIEITKQVSEDCKGSEFDSKDDDISLLDDLGFTEEDLNECKYPGGETEGLKRLNTYMTKKQWVCNFEKPNTS

PNSIEPSTTVLSPYISHGCLSSKLFYHKLKEVESGMKHSSPPVSLLGQLMWREFYYTAGAGTENFDKMVGNPLCTQIPWK

KNEEYLKAWAEGRTGYPFVDAIMRQLKQEGWIHHLARHMVACFLTRGDLWVSWEEGAKVFEDYLLDYDWSLNAGNWMWLS

ASAFFYKYYRVYSPIAFGKKTDKEGLYIRKYVPELKKYPSEFIYEPWKAPKSTQMAAGCIIGQDYPKRIVDHDKVHKDNI

QKMNAAYKLKKENKTLKRKR

>Maniola_jur_crypto_XP_045771787.1 XP_045771787.1

MLGGSVLWFRHGLRLHDNPSLNTALEDKSVPFFPVFIFDGETAGTKVVGYNRMRYLLEALQDLDNQFKKYGGRLHMIKGK

PEAVFRRLWEEFGIRKICFEQDCEPIWRARDECVKSLCREIGVGCRENVSHTLWDPDVIIRANGGIPPLTYQMFLHTVAI

IGNPPRPVDDVDLRGVNFVALPDSFYQEFTIFDKAPKPEDLGIFLEHEDIRMIRWVGGETAALVQMQNRLAVEYETFCKG

SYLPTHGNPDLLSRPISLSPALRFGCLSVRRFYWCLQDLFQQVHQGRLGSSQFITGQLIWREYFYTMSVNNPQYAQMAGN

PICLDIPWKEPEGDELERWKEGRTGFPFVDAAMRQLRTEGWLHHAVRNTVASFLTRGTLWLSWEHGLQHFLKYLLDADWS

VCAGNWMWVSSSAFEALLDSGECACPVRLGRRLEPSGHYVRRYVPELAKMPVEYIYEPWRAPLDVQQKAECIIGRDYPAP

VVDHRAAAHRNRSAMQELRRVLEKAPPHCCPSSEDEVRQFMWLGDENQIVVNTDS

>Maniola_jur_PL_XP_045775666.1 XP_045775666.1

MRNSAFKLLLTQTRMASSAKKLKLSAPSTSSENKTKIDDFMNQIQKRREETAESILKFNFNKKRVRIISQEQMVADGCEG

IVYWMSRDSRVQDNWAFLFAQKLALKNEVPLHVCFCLIAKYLDASVRQFHFLIKGLEKVAAECKKLNISFHLLEGSGAEA

LPQWIIDHKIGAVVCDFNPLRVPMSWLDGAKKKFKKNVPLIQVDAHNVVPCWVASDKQEYSARTIRNKINSKLDEYLTEF

PPVIKHPYTSKFEPEPIDWDEAIESREADKSVGPIEWAAPGYDNAVKVLKSFLDTRLKVFATKRNDPTQDALSNLSPWFH

FGQISVQRVALCVQEYKKKYTESVNAYLEESIVRRELADNFCFYCENYDSIKGASAWAQKTLDDHRKDKRPYIYTLEQLS

KAQTHDDLWNSAQIQLVKEGKMHGFLRMYWCKKILEWTPSPEDALKYAIYLNDHYSVDGRDPSGYVGCMWSICGIHDQGW

AERAVFGKIRYMNYEGCKRKFSVPAFVARYGGKVHKYVAKK

>Megachile_rot_PREDIC_XP_012148996.1 XP_012148996.1

MDESSPPKRKKRSDLLTKFKNNRENASESIMTFPFNKKRVRLLSELNDVKDCKGILYWMFRDIRVQDNWALLFAQRTALK

NNVPLHICFCIMPSFLYASIRYYKFLLKGLMKVEEECKNLNINFHLLNGEPNMSILKFVKAHRMGAVIADFNPLRLPMSW

INDLQKDLPEYIPFCQVDAHNIVPCWIASSKQEYAARTIRSKINTKLEEFLTEFPPVIKHPHKIKSKIEGNNWEKALQNI

SVDMSVDEITWAKPGYEYGVKALENFLQNGIKKYATERNDPLANALSNLSPWFHFGMISVQRCILEITEYKKIHPKSVES

FMEEAIIRRELSDNFCFYNKKYDLIEGAYPWTIETLDKHRKDKRKYLYSLDQLENSETHDDLWNACQNQMVMIGKMHGFL

RMYWAKKILEWTESPESALQWAIYLNNKYSIDGCDPNGYVGCMWSICGIHDQGWSERQIFGKIRYMNYEGCKRKFDVKEF

ILKWGKKKNEL

>Megachile_rot_PREDIC_XP_012149385.1 XP_012149385.1

MTGSRNSEIDPEVRVHGDGGKHTVHWFRKGLRLHDNPSLREGLAGASTFRCVFVLDPWFAGSTNVGINKWRFLLQCLEDL

DCSLRKLNSRLFVIRGQPADALPKLFKEWGTTNLTFEEDPEPFGRVRDHNISALCKELGISVVQKVSHTLYKLDEIIEKN

GGKPPLTYHQFQNVVACMDVPEPPVPTVTFACVGSAYTPVKEDHDDHYGVPTLEELGFDTEGLLPPVWVGGESEALARLE

RHLERKAWVASFGRPKMTPQSLLPSQTGLSPYLRFGCLSTRLFYYQLTDLYKKIKKAVPPLSLHGQLLWREFFYCAATNN

PNFDRMQGNPICVQIPWDKNIEALAKWANGQTGFPWIDAIMTQLREEGWIHHLARHAVACFLTRGDLWISWEEGMKVFDE

LLLDADWSVNAGMWMWLSCSSFFQQFFHCYCPIRFGRKADPNGDYIRRYLPVLKNFPTRYIHEPWNAPLSVQRAAKCIIG

KDYPLPMVNHSKSSRINIERMKQVYQQLNKYRGNGAPIKGLLNTLSPPVTKGNEEEKKKQVQSPSPSENQLKMETLAKTT

QQQQQQQQQQQQQLQQPHHHQHQHQ

>Megalopta_gen_CRY-1-_XP_033325936.1 XP_033325936.1

MTGSRSREINPDVGNQGQKHTVHWFRRGLRLHDNPSLREGLAGAATFRCVFVLDPWFAGSTNVGINKWRFLLQCLEDLDC

SLRKLNSRLFVIRGQPADALPKLFKEWGTTNLTFEEDPEPFGRVRDHNISALCNELGISVVQRVSHTLYKLDEIIEKNGG

KPPLTYHQFQNVVASMDPPEPPVQTVTSVCVGSAYTPLKEDHDDHYGVPTLEELGFDTEGLRPPVWVGGESEALARLGRH

LERKAWVASFGRPKMTPQSLLPSQTGLSPYLRFGCLSTRLFYYQLTDLYKKIKKAVPPLSLHGQLLWREFFYCAATKNPN

FDRMQGNPICVQIPWDKNVEALAKWANGQTGFPWIDAIMTQLREEGWIHHLARHAVACFLTRGDLWISWEEGMKVFDELL

LDADWSVNAGMWMWLSCSSFFQQFFHCYCPVRFGRKADPNGDYIRRYLPVLKNIPTRYIHEPWNAPLSVQRTAKCIIGQD

YSLPMVNHGKSSRINIERMKQVYQQLNKYRGNGTSLKGETVGLLNALPPQLVKDGEEEAKKQASPSPESQTSIEPLSKVT

QQQQQQQHSLQ

>Megalopta_gen_PL-lik_XP_033331461.1 XP_033331461.1

MDESSAPKRKRSSDLANKFMKDRRNTAESISTFHFSKKRVRRLSKFNDIKDDCKGILYWMFRDIRVQDNWAVLFAQRTAL

KNNLPLHICFCLMTNFLEASMRYYKFLLQGLEQVEGECETLNINFHLLHGEPSDSIVKFVETYKMGAVIADFCPLKLPMS

WIDNVQNKLPEDIPFCQVDAHNIVPCWEASQKQEFAAKTIRNKINTKLNEFLTEFPPVVKHLYLTDEKFEKNNWKIALDG

LEIERTVDEIAWAKPGYENGIKELESFLENRLEKYAAERNDPLSNAISNLSPWFHFGMISVQRCILEIQKYKKLYPKSVE

CFMEEAIIRRELSDNFCLYNKNYDVIEGANAWAIETLNKHRKDKRKYIYSLHDLENSKTHDDLWNACQNQLVRTGKMHGF

LRMYWAKKILEWTKSPEDALEWANHLNNKYSIDGCDPNGYVGCAWSICGIHDHGWPEREIFGKIRYMNYEGCKRKFRVKE

FISLWGKKEN

>Melanaphis_sac_CRY-1_XP_025204580.1 XP_025204580.1

MTVAVHWFRNGLRLHDNPALIEAQKNAESLITLFIFDTTMYNIEWSGYNRMRFLLESLKVLNDNLTLVGGHLYILQGAPV

KIFQMIKEKVGLDLITFEQDCDHIGRNRDNKVKMFCNENEIKYIEKVSHTLWNPKEVIDKNGGVPPFTYKQFQNTVNKIG

QPPKPVGNIDWLSTIFEELPASILEEFKVLNDPTPETFGLYPEFPENPIFSHRWYGGENRALEQLKERLECEKEAFVNGF

YLPNQVNPDLLSPSSSLSVALRYGCLSIRKFYWDLSKLFIKQFEGDLLPQYSVTSQLLWRDYFYTMSIDNKNFGQMEDNP

ACISIPWNDIKIPENKKMLECWKAGKTGFPFIDAGMRQLMQEGWVHHVVRNSLACFLTRGDLWISWVEGLNHFMKYLLDA

DLSVCSGNWIWVSSSTFEQLLDCPLCVCPVNYGLRLDPSGEYIKRYIPELKNMPIEYLYEPWKCPESVQKEVGCIIGKDY

PNCIVNHTKVSRGNRKKMLALRNSMTNEHMVPHCCPSDREEVQKCMFLPDECMQQLLPLDNDDSEMYDFYKCN

>Melanaphis_sac_CRY-1-_XP_025199458.1 XP_025199458.1

MDFEVQNKHTVHWFRKGLRLHDNPSLREGLINAKTFRCIFILDPWFAGASNVGINKWRFLLQCLVDLDNSLKKLNSRLFV

IKGQPAEALPKLFKLWGTTNFTFEEDPEPFGRVRDQNIKVMCSEMGISVITRCSHTLYQLDKIINVNGGKAPLTYHLFQK

LLECIDPPERAVPTIDKEFLGNAFTPTKYDHDEIFGVPTLEELGFKEINNPARQVWIGGETEALIRLQCHLERKAFIASY

GKPKMTPQSLIASPTGLAPYLKFGCLSTRLFFSELNELYKKIRKSQPPLSLHGQLLWRDFFYCASTNNPNFDRMVGNPIC

VQIPWDKNPRALSKWANGQTGYPWIDAIMIQLRQEGWIHCIARHAVACFLTRGDLWLSWEEGMKLYHVLCTL

>Melanaphis_sac_CRY-2_XP_025205008.1 XP_025205008.1

MDKNNVDVGHETSVHWFRKGMRLHDNPAFRLSFEAKNNSGANRWRFLQQSLVDLDTTLRKLGTRLYVIRGLPHEVFPDLF

AKWNVKLLTFELDTEPYARERDNQVEQLARKHGVKVEQKVSHTIYNTELVLRANGGCVPMTYQKFISVVDSMPNPRQPIP

APHTLPSECLLNDDLNNQEFDVPTLEELLTLKGFNPAELKPCLYPGGEKEALRRLEEYMKNKTWVCKFEKPNTSPNSLKP

STTVLSPYMKFGCLSASHFYYRLKEVIGNSPHSKPPVSLIGQLYWREFYYTVGASTPNFDKMEGNSICCQVPWDNNPDAL

EAWTNGKTGYPFIDAIMRQLRDEGWIHHLARHAVACFLTRGDLWISWEKGLAVFEELLLDADWSMNAGNWMWLSASAFFH

QFFRVYSPVAFGKKTDKSGDYIRKYIPELIKYPDQYIYEPWLAPKSIQERAGCVVGVHYPKRIVVHEDVYKNNITKMSLA

YKSSKAGKSSSSTKTSRDKSFPDKKNIKKAKLK

>Melanaphis_sac_PL_X2_XP_025192987.1 XP_025192987.1

MIDLILTMSEKSPPTKKFKKTSPEVSTSKTDNFLKDIEAERKKTAPSIMEFKFNKKRVRVLSELKEVPEWAEGIIYWTFR

DERIHDNWALLYAQKLAIKNKVSLHITFCRLKQFLNCSLRHYIHIFQGLEELETECKELDIQFHFLIGCAADILPEFVKK

HKLGAIVVDFMPVREHLLWAQQLAERIGSEVPVIQVDAHNIVPCWEASDKQEYSARTIRNKINNKLPEFLTEFPPVIKHP

YQSKFKAQPTNWDEAGKTLEVDRSVVSVPGLKAGFKAGMSELENFIKKRLQKYSIDRNNPVKDGLSKLSPWLHFGQISAQ

RCILEVSKLSKKYPESVAAYREEAIIRRELSDNFCFYNPKYDKVDGAPNWAQITLNDHRKDKRMFVYTREELENSRTHDD

LWNSAQLQMVKEGKMHGFLRMYWAKKILEWTDTPERALADAIYLNDKYSMDGRDPSGFVGCMWSICGIHDQGWRERDIFG

KIRYMNYAGCKKKFDINAFIARYGGMVHKYTKK

>Melipona_qua_CRY-1_KOX67887.1 KOX67887.1

MTGSRSSEINPEVTLHSEGGKHTVHWFRKGLRLHDNPSLREGLVGASTFRCVFVLDPWFAGSTNVGINKWRFLLQCLEDL

DCSLRKLNSRLFVIRGQPADALPKLFKEWGTTNLTFEEDPEPFGRVRDHNISALCKELGISVVQKVSHTLYKLDEIIERN

GGKPPLTYHQFQTVVASMDPPEPPVPTVTSVCVGSAYTPLKEDHDDHYGVPTLEELGFDTEGLLPPVWVGGESEALARLE

RHLERKAWVASFGRPKMTPQSLLPSQTGLSPYLRFGCLSTRLFYYQLTDLYKKIKKAVPPLSLHGQLLWREFFYCAATKN

PNFDRMQGNPICVQIPWDKNVEALAKWANGQTGFPWIDAIMTQLREEGWIHHLARHAVACFLTRGDLWISWEEGMKVFDE

LLLDADWSVNAGMWMWLSCSSFFQQFFHCYCPVRFGRKADPNGDYIRRYLPVLKNFPTRYIHEPWNAPLSVQRAAKCIVG

KDYSLPMVNHSKSSRINIERMKQVYQQLNKYRGNGASLKGETVGLLNALPPPPAKENEEEKKKTKQSPPSSENQSKIETL

VKGTQHQ

>Melipona_qua_PL_KOX78228.1 KOX78228.1

MNELNLSKRKKVDLLKKFEENRRNTSESIMTFNFNKKRIRRLSKLNNVKENSKGILYWMLRDFRIQDNWALLFAQKTALK

NNVPLHVCFCIMPSFLNASIRYYKFLLKGLLEIEKECEQLKINFHLLHGEPNMSILKFVKMYNMGAVIVDFYPLKLPMCW

VDNVQKNLPKDIPICQVDAHNIVPCWHASSKQEFAAKTIRNKINVELEEFLTEFPPVIEHPYSTKENFENNNWDIALQDV

DTDKSVNEITWAKPGYMGGTKELENFIQNRLQKYGDERNNPLSNATSNLSPWFHFGMISVQRCILEIKEYKRLYKKSVES

FMEEAIIRRELSDNFCFYNEKYDLIEGAYPWAIETLNKHRKDKRKYVYSLSQLENFKTHDDLWNACQNQMVMIGKMHGFL

RMYWAKKILEWTEIPEIALEWANYLNNKYSIDGCDPNGYVGCMWSICGVHDHGWSERDIFGKIRYMNYEGCKRKFNVAEF

VRKWRKKEDN

>Melitaea_cin_CRY-1_XP_045446444.1 XP_045446444.1

MLKIKSLKLKNNSLCWLLSRSCKQYLRKMSTSQTVVHWFRLDLRLHDNLALRNAINEAENRKHNLKPIYVIESDIKNKIG

ANRLRFLLQSLQNLDENLRKINSRLFIVRGKAVDILPELFEKWNVKFLTLQVDIDAELVKQDEIIENFCEAKDIFLVKRM

QHTVYDFNSVIKKNNGGIPLTYQKFLSLVAETQVKDTVEITKNISDECKSADFESEDFKVPTLEEIGINESELLECKYPG

GETEGLKRLDIYMSKKQWVCNFEKPNSSPNSIEPSTTVLSPYISHGCLSAKLFYHKLKQVENGMKHTLPPVSLMGQLMWR

EFYYVAGAGTRNFDKMVGNTVCTQIPWGKNDIHLKAWAEGKTGYPFVDAIMRQLKQEGWIHHLARHMVACFLTRGDLWIS

WEEGAKVFEDYLLDYDWSLNAGNWMWLSASAFFYKYYRVYSPIAFGKKTDKEGLYIRKYVPELKKYPSEFIYEPWKAPKS

VQRTAGCVVGEDYPNRIVDHDKVHKDNIQKMNAAYKLNKEKKALKRKR

>Melitaea_cin_CRY-1_XP_045448899.1 XP_045448899.1

MMGGSVLWFRHGLRLHDNPSLQAALQDRSIPFFPVFIFDGETAGTKEVGYNRMRYLLEALNDLDIQFNKYGGKLHMIKGK

PDVVFRRLWEELGIRKICFEQDCEPIWRARDERVRNLCREIGVSCCENVSHTLWDPDKVINANGGIPPLTYQMFLHTVGI

IGNPPRPVDDVDLKGVNFGTLPKSFYEEFTVFDKSPKPEDLGVFLENEDIRMIRWVGGETAALNQMQNRLAVEYETFLRG

SYLPTHGNPDLLGSPISLSPALRFGCLSVRRFYWSVQDLFQQVHQGRLSATQFITGQLIWREYFYTMSVNNPNYAQMAGN

PICLDIPWKEPENDELQRWKEGRTGFPFVDAAMRQLRTEGWLHHVVRNTVASFLTRGTLWLSWEHGLAHFLKYLLDADWS

VCAGNWMWVSSSAFEALLDSGECACPVRLGRRLEPTGQYVRRYVPELARMPVEYIYDPWNAPLQVQQRAGCVVGRHYPAR

VVDQRAAAQRNRAAMQELRRMLEKVPPHCCPSSEDEVRQFMWLGEETQAEVSTS

>Melitaea_cin_CRY-1-_XP_045455168.1 XP_045455168.1

MTKCREVGITVTSRVSHTLYKLDNIIERNGGKAPLTYHQFQALIASMPPPPPAEAAISVRMLNGAITPLSDDHDDRFGVP

TLEELGFETEGLKPPVWIGGENEALLRLERHLERKAWVASFGRPKMTPQSLLASQTGLSPYLRFGCLSTRLFYYQLTELY

KRIKRVRPPLSLHGQILWREFFYCAATRNPNFDRMEGNPICVQIPWKKNQDALAKWANAQTGFPWIDAIMTQLREEGWIH

HLARHAVACFLTRGDLWISWEEGMKVFDELLLDADWSVNAGMWMWLSCSSFFQQFFHCYCPVRFGRKTDPNGDFIRKYIP

VLKNLPTRYIHEPWVAPEAVQQAANCVIGRDYPLPMVDHAKASQINIERIKQVYAQLAKYKPQQGPSMTQVLQRPNVMQS

SPSPTSIIASINQSNYLCSQAPDPAPQSSSSSQYKEDDMFLRPTKNSIRGAGMANRNTLFKQVVIVQQAQNSRVQQNTAE

NNYDMNGVNREADKSFKINNNAMQNSEKSENYDFKNLVINSGSKKFCNNPLVYINQQTNKTEYYDQDVKIDEYSTSKPKY

FFTNNGVIPHNDTNQNFTSNYGNNYNRNEIKAHVKSEDVENENKMNINSPSDSKRTTDTNK

>Melitaea_cin_PL_XP_045449275.1 XP_045449275.1

MFQRVFNFKTLNCAQLGKSPIDCLCVNKRNMLSGSKRTASSATKLSTPAKKSKNFDASTLTNDFAKEIQKKREDTADSIL

DYKFNKKRLRIISQEQMVPEDCEGIVYWMSRDSRVQDNWAFLYAQYLALKNEVPLHVCFCLIAKYLDASVRQFHFLIKGL

EKVAEECKKLNISFHLLEGHGGDVLPQWVIDHKIGAVVCDFNPLRVPLGWLEVAKKKLKKDVPLIQVDAHNIVPCWVASD

KQEYSARTIRNKINSKLDEYLTEFPPVIKHPYTSKFEPEPIDWDEAITTREADESVKPIEWASPGYDSALEILKSFIDKR

LKIFATKRNDPTVDALSNLSPWFHFGQISVQRVALCIQPYKSKYTESVNSFLEEAIVRRELADNYCFYCEHYDSIKGASA

WAQKTLDDHRKDKRTYIYSLEQLSNAKTHDDLWNAAQLQLVQEGKMHGFLRMYWCKKILEWTPSPEDALKYAIYLNDHYS

VDGRDPNGYVGCMWSICGIHDQGWAERAVFGKVRYMNYDGCKRKFDVPAFVARYGRSGKVNSLFNGAKKSKQPDRPQ

>Microplitis_dem_PREDIC_XP_008544828.1 XP_008544828.1

MTGSRHNNRGRSVPSCKGIKDASITPNTTASGTGTATTGSATTSDRGKHTVHWFRKGLRLHDNPSLREGLSGANTFRCVF

VIDPWSAGSKSIGINKWRFLLQCLEDLDASLRKLNSRLFVIRGQPADVLPKIFREWGTTNLTFEEDPEPFGRARDHNIAT

LCKELGISVLQMVSHTLYKLDEILDRNGGKPPLTYHQFQNIVAGMDPPISPVPSITAESLGEAYTPLRDDHDDLYGVPTL

EELGFDTEGLQSSVWVGGESEALARLERHLGRKAWVASFGRPKMTPQSLLPNQTSLSPYLRFGCLSTRLFYYQLRDLYRK

IKKASPPLSLHGQLLWREFFYCAATKNPNFDRMQGNPICLQIPWDKNNEALAKWANGQTGFPWIDAIMTQLREEGWIHHL

ARHAVACFLTRGDLWISWEEGMKIFDQLLLDADWSVNAGMWMWLSCSSFFQQFFHCYCPVRFGRKADPSGDYIRRYLPVL

KSYPTRYIHEPWVAPLCVQRSAKCIIGRDYPLPMVNHSKSSRINIERMKQVYQQLSKYRDNGE

>Microplitis_dem_PREDIC_XP_008555956.1 XP_008555956.1

MSEPQKKKFKSSNFIDELDQKRKNTASSILNFPFIKKRVKVLSAVDEVAKNSKGILYWMFREGRVQDNWSFLFAQKLALK

NKLPLHVCYCILPKFLDATLRHYKFLVESLEEVSNDCKDLNISFHLLHGTPNVVVLDLVKKHKMGALVVDFFPLRVPSGW

VEDLKNSIPKDVPLCQVDGHNLVPCWVASDKLEYGARTIRGKINTKLPEYLTEFPPLIKHPHDSAFKIPTIDWKNALKDV

QIDRTVDKVDWCKPGYRGALMELESFITKRLKNYNTKRNDPTEDALSKLSPWFHFGQISVQRVILEVREYKKQHKESVEN

FMEESIIRRELSDNFCFYNKNYDSVEGANAWAIESLNQHRKDKREYIYTRDELENSQTHDDLWNAAQNQMVREGKMHGFL

RMYWAKKILEWTPSPEDALAWAIYLNDKYSMDGRDPNGYVGCMWSICGIHDQGWKERSVFGKIRYMNYKGCERKFDVKSF

VRKYDGKVVNKKNDISKMFKKK

>Mischocyttarus_mex_hypoth_KAI4474106.1 KAI4474106.1

MTGSRKSEFGNGVAVQGDGRKHTVHWFRRGLRLHDNPSLRDGLAGASTFRCVFILDPWFAGSTNVGINKWRFLLQCLEDL

DCSLRKLNSRLFVIRGQPADTLPKLFKEWGTTNLTFEEDPEPFGRVRDHKISTLCKELGISVVQRVSHTLYKLDEIIERN

GGKPPLTYHQFQNVVASMDTPEKPVSTVTSVCIGDAYTPLKDDHDDHFGVPTLEELGTQTHLYTHTSKLTHMGTRRTRVQ

LATCMEP

>Mischocyttarus_mex_hypoth_KAI4502921.1 KAI4502921.1

MGEEPPNKKLRTIDLITKIENDRKNEANSVMDFRFNKKRVKLLKISNDIKDCKGILYWMFRDARVQDNWALLFAQRTAIK

NSVPLHICFCILPKFLNATMRHYKFLLNGLKEVEKECIDLNINFHLLNGEPDSVVPDFVKKYNMGAVITDFLPLKLPMYW

VEEVMNKLPDDVPMCQVDAHNIVPCWLASDKLEYAARTIRPKINSKLNEYLTGFPPVIKHKYSTTESFPKNKWSSALNNV

KADVSVKEVTWAKPGYSGGILELDSFIKERLKEYHSKRNDPVSDAVSNLSPWFHFGMISVQRCILVVMEHKKTMAKPTEI

FMEEAIVRRELSDNFCFYNENYDSMKGAYNWAIETLNKHRNDKREYVYNLNELENSMTHDDLWNACQNQLVQQGKMHGFL

RMYWAKKILEWSPTPENALEWALLLNDKYSLDGNDPNGYVGCMWSICGIHDQGFTERQVFGKIRFMNYKGCQRKFDVKSF

VAKWSKKNPNKTQKLFK

>Modicogryllus_sia_CRY_1_BBD05665.1 BBD05665.1

MCLYPFLYLMAKVQEQILLDTNRMKFLLESLQDIDNNLHRVGGKLYIFRGNPVDVFHFISEQFNLHKICFEQDCEPIWQE

RDNAVKQFCLENKIECCEKVSHTLWDPHLVQKTNGGVPPLTFQMFLHTVSVIGPPPRPVGDINWSSVEFGTLTQLSLPHN

LHMFQQLLMPEDFGIYPEVKNRDRIVHWIGGERQALKHLKERLKVEEDAFREGYYLPNQARPDLLGPPSSQSAALRFGCL

SVRRFYWSIQDLFSNIHGSSQVPNHNITTQLIWREFFYTMSVGNKYYAEMERNPICLSIPWKENTKGHLELWEQGKTGYP

FVDAVMRQLVQDGWIHHVARNAVACFLTRGVLWISWEAGLKFFLKYLLDADWSVCAGNWMWVSSSAFEQLLDCSHCMCPV

NYGRRLDPWGEYIKRYIPELRNYPVEYLYEPWKAPLHVQEQADCIVGQDYPQRIVDHLKASEKNRQYMEDIRNQLMNPPP

HCCPSNEKEARQFMRFPDNCSDH

>Modicogryllus_sia_CRY_2_BBD05666.1 BBD05666.1

MTDVYQQLPPAAARGAGIAPGGYCVPPAKHTVHWFRKGLRLHDKPSLREGLKGASTFRCIFILDPWFAGSSNVGINKWRF

LLQCLEDLDQSLRKLNSRLFVVRGQPADALPKLFKEWGTTNLTFEEDPEPFGRVRDQNIMAMCREMGISVVSRVSHTLYR

LESIIEKNGGKPPLTYHQFQTVVASMDCPPAAEPRITARAIEGIHTPLHDDHDDRFGVPTLEELGFDTEGLLPPVWTGGE

SEALARLERHLERKAWVASFGQPKMTPQSLQASQTGLSPYLRFGCLSTRLFYYQLTDLYKKIKKACPPLSLHGQLLWREF

FYCAATNNPNFDRMNGNPICVQIPWDRNPEALAKWATGQTGFPWIDAIMTQLREEGWIHHLARHAVACFLTRGDLWISWE

EGMKVFEELLLDADWSVNAGMWMWLSCSSFFQQFFHCYCPVRFGRKADPNGDYIRKYLPVLKNFPAKYTHEPWNAPESVQ

RAARCIVGKEYSLPMVNHAVASRINIERMKQVYQQLNKYRGPPGLLAAVPSSHTQQTEQ

>Monomorium_pha_CRY-1_XP_012538568.1 XP_012538568.1

MTGSSNNEIGQAVTSGVRGDGGKHTVHWFRKGLRLHDNPSLKEGLEGASTFRCVFVLDPWFAGSTNVGINKWRFLLQCLE

DLDCSLRKLNSRLFVIRGQPADALPKLFKEWGTTNLTFEEDPEPFGRVRDHNISALCKELGISVVQRASHTLYRLDEIIE

RNGGKPPLTYHQFQNVVAGMDPPEPPVPTVTAACIGSAYTPLKDDHDDHYGVPTLEELGFDTESLLPPVWVGGESEALTR

LERHLERKAWVASFGRPKMTPQSLLPSQTGLSPYLRFGCLSTRLFYYQLTDLYMKIKKAVPPLSLHGQLLWREFFYCAAT

KNPNFDRMQGNPICVQIPWDKNVEALAKWANGQTGFPWIDAIMTQLREEGWIHHLARHAVACFLTRGDLWLSWEEGMKVF

DELLLDADWSVNAGMWMWLSCSSFFQQFFHCYCPVRFGRKADPNGDYIRRYLPVLKNFPTRYIHEPWNAPLSIQHAAKCI

IGKEYSLPMVNHSKSSRINIERMKQVYQQLNKYRGNGTSFKGENIGLLNVLLTLPAKDDDEQKRKQDSPRTSRENEQKME

TSSPTEQQQQQQQQQQ

>Musca_dom_XP_005177055.1_photolyase XP_005177055.1

MKRARGAEVENSKSAKKQTNDAAAKPSESSPGKSTSNFIDNFKQQRLECASDVLQFSFKKKRVRILSKDQEVREDCNGPV

VYWMSRDMRVQDNWAFLYAQRLALKLELPLVVVFCLVPKFSNATIRHYKFLLGGLEEVASECEELQIPFQLLMGPAKERL

LEFMKEHDGAAVVCDFAPLRVPLQWVDDVKVGLEKACKCPLIQVDAHNVVPVWVASEKQEYGARTIRNKINSKLPEFLTE

FPPVVKHKYKLKVALPNVDRKKAYESLECDMTVDEVPGIRPGYKAACKQLEEFCTKRIKLFGEKRNDPTINALSGLSPWF

HFGQISVQRCVLAVRLNKNKYKESVEAFCEEAIVRRELADNFCYYNKNYDNLKGLHDWAAKTLNDHRKDKRSPCYSLEEF

ETAHTHDDLWNSAQLQLVKEGKMHGFLRMYWAKKILEWSESPEKALEIAILLNDKYSLDGRDPNGYVGCMWSIGGIHDQG

WAERAIFGKIRYMNYQGCKRKFDVNAFVVRYGGKAYHKK

>Musca_dom_XP_005178207.1_cry-1_X1 XP_005178207.1

MSVNIHWFRHGLRLHDNPALLEAISDKNRGIKLLPVFIFDGESAGTKCVGYNRFKFLLDSLKDLDDQFRAISGGNGGRLY

LFQGNPVQIFRSLHEKFRVHKLCFEQDCEPIWNERDNSVKALCHDLGIEAVEKISHTLWDPQTVIDTNGGIPPLTYQMFL

HTVQVIGLPPRPADDPDWTNVEFIQIEENLIKELRGFLEFPTPEQFKIFPEKLSYLAKTKWIGGETQALLHLRERLNVEE

NAFRCGRYLPNQASPNILESPKSMSAHLRFGCLSVRKFYWDVHDLFRTVQLQAEGFGMQMFGGAHITGQLIWREYFYTMS

VNNPYYDRMEGNAICLTIPWAPTNIEQLNRWTEGQTGFPLIDAAMRQLLAEGWLHHTLRNTVATFLTRGGLWQNWEHGLR

YFLKHLLDADWSVCAGNWMWVSSSAFERLLDSSLVTCPVALAKRLDPLGTYIKQYVPELAKVPQLFIHEPWRMPLEEQEK

AECLIGVHYPSPMIDLTLATERNMKAMRDLRMSLTSAGAPDKTPPHCRPSNEEEVRHFFWLAD

>Musca_dom_XP_005182773.1_cry-1 XP_005182773.1

MSEKSTLIHWFRKGLRTHDNPALFKIFEKSHADPQKYCIRPIFILDPSLLQWLRVGANRWRFLQRSLDDLNKQLMALNSR

LYVVRGNPNNVFPRLFKEWQTELLTFEEDIEPFALKRDAEIKTMAREHKVEVETFWSHTVFNPYTVMQKNMGKAPLTYQK

FLGIIEKMKAPEPLERPEKLKRNNVCKDGWEVEDEKCYDVPLLADLVKNMDELGEEKFPGGETEALKRLEASLSNVGWVT

AFEKPNTAPNSLEPSTTVLSPYLKFGCLSSRLFHKKLSAILKANAKHSKPPVSLMGQLYWREFYYTAATGEPNFDRMIGN

SVCMQIPWQTNDAHLEAWTYGRTGYPFIDAIMRQLRQEGWIHHLARHAVACFLTRGDLWISWEEGQKVFEELLLDQDWAL

NAGNWMWLSASAFFYQYFRVYSPVAFGKKTDPTGAYIRKYVPELAKYPAGAIYEPWKVALGDQRKYGCVIGTDYPHRIVN

HDIVHKENIKRMSAAYKVNREVKTGKEDQEDGGGGKKRKRDTSAGGGNGPSGSKAKKKKK

>Mythimna_sep_CRY_1_AFR54426.1 AFR54426.1

MLGGSVLWFRHGLRLHDNPSLHCALEEKGFPFFPIFIFDGETAGTKLVGYNRMRYLLEALEDLDSQLKKHGGRLIMLKGK

PNVVFRRLWEEFGIRRLCFEQDCEPVWRARDDSVKAACKEIGVVCKENVSHTLWEPDTVIKANGGIPPLTYQMFLHTVAT

IGDPPRPVSNVDFTGVKFGSLPECFYQEFTVFDKTPKPEDLGVFLENEDIRMIRWVGGETTALKQMQQRLAVEYETFLRG

SYLPTHGNPDLLGPPISLSPALRFGCLSVRSFYWALQDLFRQVHQGRLATQSASHVIAGQLIWREYFYTMSVNNPNYGQM

AGNPICLDIPWKEPQGDELQRWVEGRTGFPFVDAAMRQLRTEGWLHHAARNTVASFLTRGTLWLSWEHGLNHFLKYLLDA

DWSVCAGNWMWVSSSAFEALLDSGECACPVRLGQRLDPSGEYVRRYVPELARMPVQYIYEPWKAPIDVQERATCIIGKDY

PGPVVNHLVAAQKNKNAMKELRHILQKAPPHCCPSSEDEIRQFMWLNE

>Mythimna_sep_CRY_2_AFR54427.1 AFR54427.1

MWLKKKEMIGEEVSTARRPGDKHIVHWFRKGLRLHDNPALKDGLVDATTFRCVFIIDPWFASSSNVGINKWRFLLQCLED

LDSSLRKLNSRLFVVRGQPADALPKLFREWGTTALSFEEDPEPYGRVRDHNIMSKCREVGITVTSRVSHTLYKLDQIIER

NGGKAPLTYHQFQALIASMPPPPKAEAPITAQTLNGATTPVTDDHDDRFGVPTLEELGFETEGLKPPVWVGGESEALARL

ERHLERKAWVASFGRPKMTPQSLLASQTGLSPYLRFGCLSTRLFYYQLTELYKRVKRVRPPLSLHGQILWREFFYCAATR

NPNFDRMEGNPICVQIPWEKNQEALAKWASGQTGYPWIDAIIIQLREEGWIHHLARHAVACFLTRGDLWISWEEGMKVFD

ELLLDADWSVNAGMWMWLSCSSFFQQFFHCYCPVRFGRKTDPNGDFIRRYIPALKNMPTRYIHEPWVAPESVQQSARCII

GRDYPMPMVDHAKASQVNIERIKQVYAQLAKYKPQGTLNPNAVQRPNVMQSSPSPNSIITSINQSNYLCSQAPEPPTTTP

QIIPYKDNDVVFQKPMNHRSMKPSFKQVVIVQKKQNTNVIQTVTQSKEKYIVNGQPAKQENYDFKNLVINNYVQGYSNNQ

EIFQNQQTNKNELFAQPTLKINSFNYEKQKFFLSAFTDNGVRRSAVHDEVPPPFTVALNHDTNMPYIRENKNEKDKSNDG

TFLHPMSINDEGTITNENRQNDTSNDCENYTSNENQK

>Myzus_per_CRY-1__XP_022178571.1 XP_022178571.1

MTVAVHWFRNGLRLHDNPALIEAHNNAEKLITLYIFDETTFNAKWYGYNPMRFLLESLKDLNKNLTLVGGCLYILQGNPV

NIFKIIKEKIGLNFITFEQDCDHIGRKRDEKVKTFCNENDVKYIEKVSHTLWNPKTIIEKNGGVPPFTFKQFQNTANQIG

HPPIPVGNVDWLSVIFEELPASVLDEIKVLHNPTPETFGIYPEVPENLTSPYRWYGGETRALEQLKERLEYEREAFVNGF

YLPNQVNPDLLSPPSSLSAALRYGCLSIRKFHWELSKLFIKQFEGDLLPQYSVTSQLIWRDYFYTMSIDNKNFGQMEDNP

ACISIPWNDVKIPENKKMLECWKAGKTGYPFIDAGMRQLMQEGWVHHVVRNSLASFLTRGDLWISWVEGLNHFMKYLLDA

DFSVCAGNWIWVSSSTFEQLLDCPLCVCPVSYGLRLDPSGEYIKRYVPELKNMPAQYLYEPWKCPESVQKQVGCIIGKDY

PNRIVDHTIASRGNRKKMLALRVSMTNDNMQVPHCCPSDREEVQKFMYLPDECIQQLLPLENNDSEAYDFYKCH

>Myzus_per_CRY-1-_XP_022179100.1 XP_022179100.1

MEFKVQHKHTVHWFRKGLRIHDNPSLREGLINANTFRCIFILDPWFAGASNVGINKWRFLLQCLSDLDNSLKKLNSRLFV

IKGQPAEALPKLFRQWGTTNFTFEEDPEPFGRVRDQNIKVMCSEMGISVITRCSHTLYQLDKIINVNGGKAPLTYHLFQK

LLECIDPPERAVPSIDKEFLGNAFTPTKYDHDEIFGVPTFEELGFKEINNSARHIWVGGETEALIRLQCHLERKAFIASY

GKPKMTPQSLVASPTGLAPYLKFGCLSTRLFFSELNELYKKIRKSQPPLSLHGQLLWRDFFYCASTNNPNFDRMVGNPIC

VQIPWDKNPRALSKWANGQTGYPWIDAIMIQLRQEGWIHCIARHAVACFLTRGDLWLSWEEGMKVFDELLLDADWSVNAG

YWMWYSCSSFYQEFIHCYCPVRFGRKVDPNGDYIRRYIPVLNNMPNQYIHEPWLAPESIQFTANCIIGIDYPLPIVNHVN

ASKINLERMKLAYQQLSNCQPQLENGKLILISSLRR

>Myzus_per_CRY-2_XP_022169149.1 XP_022169149.1

MDKTSADARHETTVHWFRKGMRLHDNPAFKLSCEAKNGSGERYKLRPIYILDPYFRKYIRAGANRWRFLQQSMVDLDTTL

RQLGTRLYVIRGLPHEVFPDLFAKWNVKLLTFELDTEPYARERDSQVEQLARKQGVKVEQKVSHTIYNTELVLRANGGSV

PMTYQKFVSVVGSMPTPRRPIPAPDMLPPECLLDDDLNNPEFDVPTLDELLKLKGFNPAELKPCLYPGGEKEAVRRLEEY

MKNKSWVCKFEKPNTSPNSLKPSTTVLSPYMKFGCLSASHFYYRLKEVIGNSPHSKPPVSLIGQLYWREFYYTVGASTPN

FDKMIGNPICCQVPWDDNPDALEAWTNGRTGYPFIDAIMRQLRDEGWIHHLARHAVACFLTRGDLWISWEKGLAVFEELL

LDADWSMNAGNWMWLSASAFFHQFFRVYSPVAFGKKTDKSGDYIRKYIPELAKYPDQYIYEPWSAPKSLQERAGCVVGVH

YPKRVVVHEDVYKNNITKMSLAYKSTKAGKSSNSKKSRDMSSSPDKKNIKKSKLK

>Myzus_per_PL_XP_022172380.1 XP_022172380.1

MSDNSPPTKKLKKTSSEVGTSKTSNFLNDIASERNKTAPSIMEFKFNKKRVRVLSEQKEVPEWAEGVIYWTFRDERIQDN

WALLYAQKLAIKNKVSLHITFCRLKQFLNCSLRHYKHIFQGLEELETECKSLDIQFHFLIGCAADILPDFVKKHKLGAIV

VDFMPVREHMTWAKQLAERVGSEVPVIQVDAHNIVPCWVASDKQEYSARTIRNKINNKLPEFLTEFPPVIKHPFPSTFKA

KPTNWDEADKTLEVDRSVISVPGLKAGFKAGMSELELFLKKRLPKYSTDRNNPVKDGLSKLSPWLHFGQISAQRCILEVS

KLSKQYPESVAAYREEAIVRRELSDNFCFYNPKYDKIEGAPNWAQTTLNEHRKDKRMFVYTREELEKSRTHDDLWNSAQI

QLVKEGKMHGFLRMYWAKKILEWTDTPDRALADAIYLNDKYSMDGRDPSGFVGCMWSICGIHDQGWREREIFGKIRYMNY

EGCKRKFDINAFIVRYGGMVHKYTKK

>Nasonia_vit_XP_008204431.1_cry-1_X1 XP_008204431.1

MTGSQGNDIGCEISCRADIYQQPQKQLQQQHHQQQSQIQQPSPPLVNNDTKSDGLAIQGDGKKHTVHWFRKGLRLHDNPS

LREGLAGASTFRCVFVLDPWFAGSANVSINKWRFLLQCLEDLDRSLHQLNSRLFVIRGQPADALPKLFREWGTTSLTFEE

DPEPYGRVRDENITTLCKELGITVVQRVSHTLYKLDEIIEKNGGKPPLTYHQFQNVIARMDPPEYPAAAVTAACIGSAYT

PLKDDHDDFFGVPTLEELGFDTEGLMAPVWVGGETEALARLERHLERKAWVASFGRPKMTPQSLLPSQTGLSPYLRFGCL

STRLFYYQLADLYKKVGFYIFKDKYICILRLHFKQLYGFWTFIQIKKTIPPLSLHGQLLWREFFYCAATNNPNFDRMHGN

PICVQIPWDKNVVALSKWANGQTGFPWIDAIMTQLREEGWIHQLARHAVACFLTRGDLWISWEEGMKVFDELLLDADWSI

NAGMWMWLSCSSFFQQFFHCYCPVRFGRKADPNGDYIRRYLPVLKNFPTRYIHEPWNAPLSVQRAAKCIIGQEYALPMVN

HSKSSRINVERMKRVYQQLSKYRANGVSLKGETIGLLSIVPTTPSLPFDQQQQQQDDDKKQQQNSLNVDNSPNNDCPASH

LHQQQLQQHVHQHQYH

>Nasonia_vit_XP_031784876.1_photolyase XP_031784876.1

MAPCIFVTKLIAIKYLSKTNFTVTKATSSTNFKMSGPSTKKLRTSHTDLVKQIEEQRNQTADSVMTFKFNKKRVRILTDS

DEVSSESKGIVYWMFRDARVHDNWAMLFAQKIALKNKVPLHVCFCILPKFLDATIRHYKFLLEALEEVEKDCKELNINFH

LLHGEPNTAIINFVEKYKMGAVIADFFPLRLPLFWLEDIKKKLPKKIPLCQVDAHNIVPCWVASEKLEYAARTIRNKINS

KLDEFLTEFPPVIKHPHTSDQKFDKINWDTALDDVLVDKSVDKITWAKAGYKGGIAELDKFLKIRLRIYDEKRNNPIFNA

LSNLSPWFHFGMISVQRCILEAKKYKSQYNKSVEAFMEEAIVRRELSDNFCFYNEHYDSLKGAYDWARETLNQHRNDKRD

YIYTLNELENGLTHDDLWNAAEIQLVKEGKIHGFLRMYWAKKILEWTETPDDALKWSIYLNDKYSMDGRDPNGYVGCMWS

ICGVHDQGWRERPVFGKIRYMNYKGCERKFDVQAFVMKYGAKVQLTKDENKRKGKKK

>Neodiprion_fab_CRY-1-_XP_046429293.1 XP_046429293.1

MTDGEKAQHGGLKPFRRDGKKHIVHWFRKGLRLHDNPALREGLTGATTFRCVFVLDPWFAGSTNVGINKWRFLLQCLEDL

DRSLWKLNSRLFVIRGQPADVLPKLFKEWGTTDLTFEEDPEPFGRVLNQNITTLCEEMGISVVQRVSHTLYKLDSIIEKN

GGKAPLTYHQFLNVAAAMDPPPPPEALVTSEFTTGAYTPIMVNHNDVYGVPTLEELGFETENLLPPVWIGGETEALVRLE

YHLARRYWVASFGRPKMTPQSLLASQTGLSPYLRFGCLSTRLFFYQLTDLYKKVLRIKKAVPPLSLHGQLLWREFFYCAA

TRNPSFDRMQGNPICVQIPWDKNAEALAKWANGQTGFPWIDAIMTQLREEGWIHHLTRHAVACFLTRGDLWLSWEEGMKV

FDELLLDADWSVNAGMWMWLSCSSFFQQFFHCYCPVRFGRKADPNGDYIRRYVPALKTFPAKYIHEPWNAPQGIQQTAKC

IIGKDYPLPMVDHGKSSRINIERMKQVYQQLNKYRGNGLPGTRGETIEGLLNCTLATATLPQSEEDEKNKHQTTSNPSLS

PARHQKTICSILSMVTLRQLDQTAEFPSSEMD

>Neodiprion_fab_PL_X1_XP_046420615.1 XP_046420615.1

MDKFVAKRAKLSNFVNKLEEDRKNDSESVMKFKFNKKRVRVLTAIDKVKDNCKGIVYWMFRDSRVQDNWAFLFAQKTAIK

NRLPLHVCFCILPKFLGATIRHYKFHLAALKQVELECKNLNVNFHLLRGEPNDVILKFVEKHDMGAVITDFFPLRIPLSW

VDDLKKKLPEDIPLCQVDAHNIVPCWEASDKLEYSARTIRNKINSKLDEYLTQFPPVIKHPYTTKQKFTENDWENALKDV

EIDKSVKEITWAKPGYEEGILELERFIEKRLKLYDSKRNDPTLNALSNLSPWFHFGMISVQRCILEVSKYKNSHKKSVES

FMEEAIVRRELSDNFCFYNEHYDSLKGAKQWAVDTLDNHRKDKREYVYTLKEFENSLTHDDLWNSAQNQLLQDGKIHGFL

RMYWAKKILEWTRTPEEALEWSIYLNDKYSMDGRDPSGYVGCMWSICGIHDQGWAERSIFGKIRFMNYKGCQRKFDVKAF

VARWGGKVHTKKK

>Neodiprion_lec_XP_015515077.1_cry-1_X1 XP_015515077.1

MTDGEKAQHGGLKPFRRDGKKHTVHWFRKGLRLHDNPALREGLTGATTFRCVFVLDPWFAGSTNVGINKWRFLLQCLEDL

DRSLWKLNSRLFVIRGQPADVLPKLFKEWGTTDLTFEEDPEPFGRVLNQNITTLCEEMGISVVQRVSHTLYKLDSIIEKN

GGKAPLTYHQFQNVAAAMDPPPPPEALVTSEFTTGAYTPIMVNHNDVYGVPTLEELGFETENLLPPVWIGGETEALVRLE

YHLARRYWVASFGRPKMTPQSLLASQTGLSPYLRFGCLSTRLFFYQLTDLYKKIKKAVPPLSLHGQLLWREFFYCAATRN

PSFDRMQGNPICVQIPWDKNAEALAKWANGQTGFPWIDAIMTQLREEGWIHHLTRHAVACFLTRGDLWLSWEEGMKVFDE

LLLDADWSVNAGMWMWLSCSSFFQQFFHCYCPVRFGRKADPNGDYIRRYVPALKTFPAKYIHEPWNAPQSIQQTAKCIIG

KDYPLPMVDHGKSSRINIERMKQVYQQLNKYRGNGLPGTRGETIEGLLNCTLATATLPQSEEDEKHKHQTTSNPSLSPAR

HQKTICSILSMVTLRQLDQTAEFPSSEMD

>Neodiprion_lec_XP_015522462.1_photolyase XP_015522462.1

MDQFVAKRAKLSNFVNKLEEDRKNDSESVMKFKFNKKRVRVLTAIDKVKENCRGIVYWMFRDSRVQDNWAFLFAQKTAIK

NRLPLHVCFCVLPKFLGATIRHYKFHLAALKQVELECKNLNVNFHLLRGEPNDVILKFVEKYDMGAVITDFFPLRLPLSW

VDDLKKKLPEDIPLCQVDAHNIVPCWEASDKLEYSARTIRNKINSKLDEYLTQFPPVIKHPYTTKQKFTENDWENALKDV

EIDKSVKEITWAKPGYEEGILELERFIEKRLKLYDSKRNDPTLNALSNLSPWFHFGMISVQRCILEVSKYKNSHKKSVES

FMEEAIVRRELSDNFCFYNEHYDSLKGAKQWAVDTLDNHRKDKREYVYTLKEFENSLTHDDLWNSAQNQLLQDGKIHGFL

RMYWAKKILEWTRTPEEALEWSIYLNDKYSMDGRDPSGYVGCMWSICGIHDQGWAERSIFGKIRFMNYKGCQRKFDVKAF

VARWGGKVHTKKK

>Neodiprion_pin_CRY-1__XP_046483484.1 XP_046483484.1

MTDGEKAQHGGLKPFRRDGKKHTVHWFRKGLRLHDNPALREGLTGATTFRCVFVLDPWFAGSTNVGINKWRFLLQCLEDL

DRSLWKLNSRLFVIRGQPADVLPKLFKEWGTTDLTFEEDPEPFGRVLNQNITTLCEEMGISVVQRVSHTLYKLDSIIEKN

GGKAPLTYHQFQNVAAAMDPPPPPEALVTSEFTTGAYTPIMVNHNDVYGVPTLEELGFETENLLPPVWIGGETEALVRLE

YHLARRYWVASFGRPKMTPQSLLASQTGLSPYLRFGCLSTRLFFYQLTDLYKKVLRIKKAVPPLSLHGQLLWREFFYCAA

TRNPSFDRMQGNPICVQIPWDKNAEALAKWANGQTGFPWIDAIMTQLREEGWIHHLTRHAVACFLTRGDLWLSWEEGMKV

FDELLLDADWSVNAGMWMWLSCSSFFQQFFHCYCPVRFGRKADPNGDYIRRYVPALKTFPAKYIHEPWNAPQSIQQTAKC

IIGKDYPLPMVDHGKSSRINIERMKQVYQQLNKYRGNGLPGTRGETIVSNDWQTYDSEFYSFRSRRTAKLYASDSHTPPV

SRR

>Neodiprion_pin_PL_X1_XP_046477006.1 XP_046477006.1

MDQFVAKRAKLSNFVNKLEEDRKNDSESVMKFKFNKKRVRVLTAIDKVKENCKGIVYWMFRDSRVQDNWAFLFAQKTAIK

NRLPLHVCFCVLPKFLGATIRHYKFHLAALKQVELECKNLNVNFHLLRGEPNDVILKFVEKYDMGAVITDFFPLRLPLSW

VDDLKKKLPEDIPLCQVDAHNIVPCWEASDKLEYSARTIRNKINSKLDEYLTQFPPVIKHPYTTKQKFTENDWENALKDV

EIDKSVKEITWAKPGYEEGILELERFIEKRLKLYDSKRNDPTLNALSNLSPWFHFGMISVQRCILEVSKYKNSHKKSVES

FMEEAIVRRELSDNFCFYNEHYDSLKGAKQWAVDTLDNHRKDKREYVYTLKEFENSLTHDDLWNSAQNQLLQDGKIHGFL

RMYWAKKILEWTRTPEEALEWSIYLNDKYSMDGRDPSGYVGCMWSICGIHDQGWAERSIFGKIRFMNYKGCQRKFDVKAF

VARWGGKVHTKKK

>Neodiprion_vir_crypto_XP_046619635.1 XP_046619635.1

MTDGEKAQHGGLKPFRRDGKKHIVHWFRKGLRLHDNPALREGLTGATTFRCVFVLDPWFAGSTNVGINKWRFLLQCLEDL

DRSLWKLNSRLFVIRGQPADVLPKLFKEWGTTDLTFEEDPEPFGRVLNQNITTLCEEMGISVVQRVSHTLYKLDSIIEKN

GGKAPLTYHQFLNVAAAMDPPPPPEALVTSEFTTGAYTPIMVNHNDVYGVPTLEELGFETENLLPPVWIGGETEALVRLE

YHLARRYWVASFGRPKMTPQSLLASQTGLSPYLRFGCLSTRLFFYQLTDLYKKVLRIKKAVPPLSLHGQLLWREFFYCAA

TRNPSFDRMQGNPICVQIPWDKNAEALAKWANGQTGFPWIDAIMTQLREEGWIHHLTRHAVACFLTRGDLWLSWEEGMKV

FDELLLDADWSVNAGMWMWLSCSSFFQQFFHCYCPVRFGRKADPNGDYIRRYVPALKTFPAKYIHEPWNAPQGIQQTAKC

IIGKDYPLPMVDHGKSSRINIERMKQVYQQLNKYRGNGLPGTRGETIEGLLNCTLATATLPQSEEDEKNKHQTTSNPSLS

PARHQKTICSILSMVTLRQLDQTAEFPSSEMD

>Neodiprion_vir_PL_X1_XP_046614427.1 XP_046614427.1

MDKFVAKRAKLSNFVNKLEEDRKNDSESVMKFKFNKKRVRVLTAIDKVKDNCKGIVYWMFRDSRVQDNWAFLFAQKTAIK

NRLPLHVCFCVLPKFLGATIRHYKFHLAALKQVELECKNLNVNFHLLRGEPNDVILKFVEKYDMGAVITDFFPLRLPLSW

VNDLKKKLPEDIPLCQVDAHNIVPCWEASDKLEYSARTIRNKINSKLDEYLTQFPPVIKHPYTTKQKFSENDWENALKDV

EIDKSVKEITWAKPGYEEGILELERFIEKRLKLYDSKRNDPTLNALSNLSPWFHFGMISVQRCILEVSKYKNSHKKSVES

FMEEAIVRRELSDNFCFYNEHYDSLKGAKQWAVDTLDNHRKDKREYVYTLKEFENSLTHDDLWNSAQNQLLQDGKIHGFL

RMYWAKKILEWTRTPEEALEWSIYLNDKYSMDGRDPSGYVGCMWSICGIHDQGWAERSIFGKIRFMNYKGCQRKFDVKAF

VARWGGKVHTKKK

>Nesidiocoris_ten_unname_CAB0002124.1 CAB0002124.1

MKFLLESLEDLDKQLKRHGGRLHMFKGDPCSVFRRLWEDIEINKICFEQDCEPIWRERDQEVMSMCQELGIECIERVSHT

LWDPKLVIKTNGGIPPLTYQMFLHTTSVIGTPPKPCPGPDFSHIKFGSLSSSLAQELRVLDSVPTPENFGLVKEVGDKLV

VWVGGETRALQHLESRLQAEKEAFAQRILLSNQTQPNLVGSPTSQSAALRFGCLSIRRFYWTIVDTFNEVFEDLPMPSQS

VTGQLVWREYFYTMSVDNPYYAEMERNPICLKIDWLSRTDPTYQKKFESWKNGMTGYPFIDAVMRQLLAEGWVNHVARNA

VACFLTRGDLWISWEDGLNHFLKYLLDADWSVCAGNWMYVSSSAFEQLLDCSYCICPVNFGRRLDPYGEYVKRYVPEVRN

LPLEYIFEPWSAPIDVQEASKCVIGKDYPERIVDHNDVSAQNSAKMEAVRRRLVNKIPHCCPSNTEELYQFMWLPPEQHD

HLLGDDMN

>Nesidiocoris_ten_unname_CAB0005475.1 CAB0005475.1

MNRDQRVQDNWALLYAQKLALKFKLPLHVCFCTLPKYMDATIRHSMFMLKGESIVREVQAFCSLEVCPVFKRKFAHFPGL

KEVSTELDELDIPFHLLYCDSKDVVGQKVLDLVSQHEIGCVVVDFSPLRIARGWVDDLKKILPKDIPLCEVDAHNIVPCW

VASDKLEYGARTIRNKINNKLGEYLTQFPPVIKHPHTARCPQSVIDWEEEAKKFEVDRTVGEIDWAKPGYKEGMKTLYEF

CEKRLKNFGTKRNNPLMKALSNLSPWYHFGQISIQRCILYVRTFKKQNSESVDAFCEESIVRRELADNFCFYNPNYDKID

GAYDWAKKTLNDHKKDKRTYVYTCEQLAESKTHDDLWNSAQIQLVKEGKMHGFLRMYWAKKILEWTPSPEEALRIALYLN

DRYSIDGRDPNGFVGCMWSICGIHDQGWREREIFGKIRYMNYEGCKRKFDVAAFVARYGGKVYKASKK

>Nesidiocoris_ten_unname_CAB0011249.1 CAB0011249.1

MGEKHLVHWFRKGLRLHDNPSLKRGLKNATTFRCIFILDPWFAGSSNVGINKWRFLLQCLEDLDRNLRKLNSRLFVIRGQ

PADILPKLLKEWGTTCLTFEEDPEPFGRVRDQNIIALCRSMSITVYTEVAHTLYKLESIIEKNNGKAPLTYHQFQGIISS

MDPPPLPESPVSLATIENAWVASFGRPKMTPQSLLASQTGLSPYLRFGCLSTRLFYYQLNDLYRKIKRAVPPLSLHGQIL

WREFFYCAATRNPNFDRMIGNPICVQVPWDKNPEALAKWANVSRHSNVGILQFNNDKKIWFGDWYVDFIDNLSIVFLCAS

KFFMIQGQTGFPWIDAIMTQLREEGWIHHLARHAVACFLTRGDLWISWEEGMKVFDELLLDADWSVNAGMWMWLSCSSFF

QQFFHCYCPVRFGRKADPNGDYIRRYLPVLKNMPTKYIHEPWSCPESVQKAAKCIIGVDYAVPMLNHSVVAKHNIERMRQ

VYHQLIKYKGSGWLMNDAIFRISIEFDRNIKYDVNGNITYFSMHVLFLFYDFYKKIRHSK

>Nezara_vir_unname_CAH1398890.1 CAH1398890.1

MTEKHTVHWFRKGLRLHDNPSLRQGLKGAKTFRCIFILDPWFANASNVGINKWRFLLQCLEDLDRSLRKLNSRLFVLRGQ

PADILPKLFKEWGTTCLSFEEDPEPFGRVRDQNISTMCKAMNITVISLVAHTLYKLEFIIERNGGRAPLTYHQFQTVVAG

MDSPPLPDPPVTAATIADAISPISDNHDEKYGVPTLEELGFCTEGLVPGVWQGGESEALSRLERHLERKAWVASFGKPKM

TPQSLLPSQTGLSPYLRFGCLSTRLFYYQLNDLYRKIKKAVPPLSLHGQVLWREFFYCAATKNPNFDKMIGNPICVQVPW

DKNPEALAKWANGQTGFPWIDAIMTQLREEGWIHHLARHAVACFLTRGDLWISWEEGMKVFEELLLDADWSVNAGMWMWL

SCSSFFQQFFHCYCPVRFGRKADPNGDYIRKYLPVLKNMPTKYIHEPWNCPESVQRAAKCTIGVDYPLPMLNHSVVSKHN

IKRMKQVYLQLRNFKQPGIPPAPLQESLLEKKKAEENSYEENIFATPSQTFKGNNMKNAK

>Nezara_vir_unname_CAH1405666.1 CAH1405666.1

MTNPFKKLKTEGSGDTGFEEFLKGIENDRIQAGKTVQQFGYNKNRIRFFSKCKEIPSWSKGVLYWMTREERIQDNWSLLY

AQKVALKHKLPLHICFYLRRTFMNAPIRHFKFLLKGLEETAKESRKLNIPFHMFISDEGENQIIDFMVENKFGYVVIDFS

PLRIARTWAENLKKTLPDDVPLVQVDGHNIVPCWVASDKLEYGARTIRNKLKNKFAEFLTPFPPVTEHPYSGEQKAKEID

WAAAEASLEVDRTIDEVSWAKPGYLNGMKMLHEFCQKRLSKFAQKRNDPLGNALSNLSPWFHFGQISVQRCILVVESFKS

KYKESVESFCEEAIIRRELSDNFCYYNPNYDNIKGAYDWARKTLEEHKKDKREWLYTQEELENSLTHDDLWNSTQIQLVK

EGKIHGFLRMYWAKKILEWTESPEKALEISIYLNDKYSLDGRDPNGFVGCMWSICGIHDQGWKERPVFGKIRYMNYKGCE

RKFNVSAFVARYGGKVHKKKK

>Nicrophorus_ves_PREDIC_XP_017769677.1 XP_017769677.1

MNSHVGPSRGCPPDKHMVHWFRKGLRLHDNPSLKEGLKGATTFRCVFVLDPWFAGSSNVGINKWRFLLQCLEDLDRNLKK

LNSRLFVIRGQPADALPKLFKEWGTTCLTFEEDPEPFGRVRDHNITTLCKELDITVVQRVSHTLYHLQNIIDKNNGRAPL

TYHQFLAVIAGMDAPPTPECLVTSKALNGAYTPISEDHDEKYGVPTLEELGFDTDGLLPPVWQGGESEALGRLERHLERK

AWVASFGRPKMTPQSLLPSQTGLSPYLRFGCLSTRLFYYQLTDLYKKIKRAFPPLSLHGQLLWREFFYCAATKNANFDKM

IGNPICVQIPWDKNAEALAKWANGQTGFPWIDAIMTQLREEGWIHHLARHAVACFLTRGDLWLSWEEGMKVFEELLLDAD

WSVNAGMWMWLSCSSFFQQFFHCYCPVKFGRKADPNGDYIRKYLPVLKNMPVQYIHEPWTAPENVQRATKCIIGKDYPLP

IVNHGVASRINIQRMKQVYQQLTKYRILDRHHSCSTPMKSYKDGYQARAVTVGNSNDD

>Nilaparvata_lug_CRY-1_XP_039290665.1 XP_039290665.1

MSDEIRGSSVLWFRHGLRFHDNPALHAAIQANYNFYPIFIFDGESAGTKVVGPNRMRFLLESLKDLDSQLQIVGQRLFVF

KGSPVKIFEWMAKSLNMKTLCFEQDCEPIWAERDNAVKSFCDGAGVKWIEKVSHTLWNPKEVIEANGGVPPLTYQMFLYT

VSTIGNPPRPETDVDWSKVKFGKLPEDIPDDILLYPGVPTLEDFNMRAYEGGERMVRWVGGETTAIANLRSRILVEEEAF

RCGFYLPNQANPDLVAPPTSQSAALRFGCLSVRRFYWTLHDLFNEIHMGKLPSNQNITGQLIWREYFYTMSVDNAHYGEM

ARNPICLDIPWMPASNANHSHFLLRWKQGMTGYPFIDAAMRQLLQEGWIHHVARNAVASFLTRGDLWLSWEEGLRHFLEH

LLDADWSVCAGNWMWVSSSAFEQLLDCSHCVCPVNYGRRLDPWGVYVKRYVPELSQFPVQFIYEPWKLPLEEQEKYNCVI

GRDYPERIVEHKVASQINRKKMEQIRDSLMNGVPHCCPSNTEEVRQFMWLPDNCSDHVCVPT

>Nilaparvata_lug_CRY-1__XP_039295831.1 XP_039295831.1

MTGEVVPSQCGWSRGQDMMGGGRGVGDNRMQRIPQEGGGVGGTKKQAGGGGQKHTVHWFRKGLRLHDNPSLREGLRNATT

FRCIFILDPWFTGASNVSINKWRFLLQCLEDLDNSLKKLNSRLFVVRGQPADVLPKIFKEWGTTNLTFEEDPEPFGRVRD

QNVMAICKEMGITVVSRVSHTLYRLDDIIDKNGGTAPLTYRKFQTVVASLDEPPKAEPTVTAKVVATAVTPINEDHDDKF

GVPSLEELGFDTEGLLPPVWQGGESEALARLERHLERKAWVASFGRPKMTPQSLLASQTGLSPYLRFGCLSTRLFYYQLS

DLYKKIKKAAPPPSLHGQLLWREFFYCAATNNSNFDRMVGNPICVQIPWTNNPEALAKWANGQTGFPWIDAIMHQLREEG

WIHHLARHAVACFLTRGDLWVSWEKGMVVFDELLLDADWSVNAGMWMWLSCSSFFQQFFHVYCPVRFGRLADPSGDFIRR

YVPALKNFPSAYIHEPWNCPVELQRSAKCIIGEDFPLPMVNHVQASRVNMERLKQVYSRLAEFRNNNMHLMRPLPSPSGP

TTSNGVMTMLMDNCEISSQSNHVVNGIKNERNSKESIDKAMFNTPFLPKHKDKLNQKRFIMHK

>Nilaparvata_lug_PL__XP_039299515.1 XP_039299515.1

ILDNWALLFAQKLALKNEIPLHVCFCLKPRFMEATIRHYKFLLKGLEEVAEDCKKLNIQFNFLIGDGERVLPGFVEKNSI

GAVVVDFMPLKGPMSWAEQLKKTLPKDVPLCQVDAHNIVPCWIASDKQEYGARTIRNKINNKLKEYLTEFPPLIKHKYGQ

VKDEVIDWEGGEKTLEVDRSVEPVTWLTPGYRGAIQMLDSFINKRLKKFSTKRNDPTVDALSNLSPYFHFGQMSVQRAIL

TVREHRSKAPESVDAFCEEAIIRRELADNFCFYNKNYDNINGCFDWAKKTLNDHRKDKREYIYTKEELENAKTHDDLWNS

AEVQLLKEGKIHGFLRMYWAKKILEWTTSPEEALEISIYLNDKYSMDGRDPNGFVGCMWSICGIHDQGWAERNVFGKIRY

MNYAGCKRKFDVAAFVARYGGKSYPYKKSK

>Nomia_mel_CRY-1__XP_031848260.1 XP_031848260.1

MTGSRNRDINPEVGIRAEGQKHTVHWFRRGLRLHDNPSLREGLAGASTFRCVFVLDPWFAGSTNVGINKWRFLLQCLEDL

DCSLRKLNSRLFVIRGQPADALPKLFKEWGTTNLTFEEDPEPFGRVRDHNISALCNELGISVVQKVSHTLYKLDEIIEKN

GGKPPLTYHQFQNVVAGMDPPEPPVQTVTSVCVGSAYTPLKEDHDDHYGVPTLEELGFDTEGLRPPVWVGGESEALARLG

RHLERKAWVASFGRPKMTPQSLLPSQTGLSPYLRFGCLSTRLFYYQLTDLYKKIKKAVPPLSLHGQLLWREFFYCAATKN

PNFDRMQGNPICVQIPWDKNVEALAKWANGQTGFPWIDAIMTQLREEGWIHHLARHAVACFLTRGDLWISWEEGMKVFDE

LLLDADWSVNAGMWMWLSCSSFFQQFFHCYCPVRFGRKADPNGDYIRRYLPVLKNIPTRYIHEPWNAPLSVQRAAKCIIG

QDYSLPMVNHSKSSRINIERMKQVYQQLNKYRGNVSSLRLQEPPSKARPLVC

>Nomia_mel_PL_XP_031847830.1 XP_031847830.1

MDELSPPKRIKTFDLLEKFKNNRKNTAESIMTFNFNKKRVRLLSKLNDVRDGCKGILYWMFRDVRVQDNWALLFAQRTAL

KNKLPLHICFCIMPNFLGASMRHYKFLLQGLKEIEAECKTLNINFHLLHGEPNVSIIEFVKTYQMGAVIADFCPLKLPMS

WINDVQNNLPVDVPFCQVDAHNIVPCWEASEKQEFAARTIRNKINTRLKEFLVEFPPAIRHPYLTKGRFKENNWETALEN

IEVDSAVGEIKWAKPGYENGIKELESFIQNRLKRYATERNDPLSNATSNLSPWFHFGMISVQRCILEVQEYKKLYSKSVE

SFMEEAIVRRELSDNFCFYNENYDVVEGAYAWAIETLNKHRKDKRTYVYTLNELENSETHDDLWNACQNQMITVGKMHGF

LRMYWAKKILEWAETPENALEWANYLNNKYSIDGCDPNGYVGCAWSICGVHDHGWTERSIFGKIRYMNYEGCKRKFKVKE

FVDKWEKKEADNNISK

>Nylanderia_ful_CRY-1-_XP_029167776.1 XP_029167776.1

MTGSSNNEMVQGVTPGVRGDGRKHTVHWFRKGLRLHDNPSLREGLAGASTFRCVFVLDPWFAGSTNVGINKWRFLLQCLE

DLDCSLKKLNSRLFVIRGQPADALPKLFKEWGTTNLTFEEDPEPFGRVRDHNISALCKELGISVVQKVSHTLYKLDEIIE

RNNGKPPLTYHQFQNVVAGMDPPEPPVPTVTAACIGSAYTPLKDDHDDHYGVPTLEELGFDTEGLLSPVWVGGESEALAR

LERHLERKAWVASFGRPKMTPQSLLPSQTGLSPYLRFGCLSTRLFYYQLTDLYKKIKKAVPPLSLHGQLLWREFFYCAST

TNPNFDRMQGNPICVQIPWDKNVEALAKWANGQTGFPWIDAIMTQLREEGWIHHLARHAVACFLTRGDLWISWEEGMKVF

DELLLDADWSVNAGMWMWLSCSSFFQQFFHCYCPVRFGRKADPNGDYIRRYLPVLKNFPTRYIHEPWNAPLSIQHTAKCI

IGKEYSLPMVNHSKNSRINIERMKQVYQQLNKYRGNGASFKGENIGLLNALLAPPTDAEEEQRKQDSANRENEQKMETIN

NSAQQQQQQQQQQ

>Nymphalis_io_CRY-1_XP_050342844.1 XP_050342844.1

MSKLPTVVHWFRLDLRLHDNLALRNAINEAENRKHILKPIYVVDPDIKNKIGANRLRFLIQSLQDLDLNLRKINSRLFII

KGNAVQCLPQIFEKWNVNFLTQQVDIDAELVKQDEVIDQVCEENDIFVVKRMQHTVYDFNSVIKKNNGSIPLTYQKFLSL

VADTQVKDTIEISKKILEECKSTDFESNEYNVPSLEDVGIDDSELLECKYPGGETEGLKRLNVYMEKKQWVCSFEKPNSS

PNSIEPSTTVLSPYLSHGCLSAKLFYHKLKQVENGMKHTLPPVSLMGQLMWREFYYVAGAGTKNFDKMVGNTVCTQIPWG

KNEAHLKAWAEGRTGYPFVDAIMRQLKQEGWIHHLARHMVACFLTRGDLWISWEEGAKVFEDYLLDYDWSLNAGNWMWLS

ASAFFYKYFRVYSPIAFGKKTDKEGLYIRKYVPELKKYPSEFIYEPWKAPKSVQRTAGCIIGEDYPNRIVDHDKVHKDNI

QKMNAAYKVNKEKKALKRKR

>Nymphalis_io_CRY-1_XP_050345943.1 XP_050345943.1

MVGGSVLWFRHGLRLHDNPSLQNALEDINTPLFPIFIFDGETAGTKIVGYNRMRYLLEALNDLDLQFKKYGGKLHLIKGK

PEHVFRRFWEVLGIRKICFEQDCEPIWRARDDCVRNLCREIGISCHENVSHTLWDPDSVIQANGGIPPLTYQMFLHTVAI

IGDPPRPVGDVDLKGVNFASLPRSFYDEFTIFEEVPKPEDLGVFLENEDIRMIRWVGGETAALKQMQNRLAVEYETFCRG

SYLPTHGNPDLLGPPISLSPALRFGCLSVRRFYWCVQDLFQQVHQGRLASTQFITGQLIWREYFYTMSVNNPNYAQMANN

PICLDIPWKEPESDELQRWKEGRTGFPFVDAAMRQLRSEGWLHHAVRNTVASFLTRGTLWLSWEHGLAHFLKYLLDADWS

VCAGNWMWVSSSAFEALLDSGECACPVRLGRRLEPSGHYVRRYVPELAHMPVDYIYEPWNAPLEVQERAGCVVGRHYPAP

VVDHRAAAQRNRANMQDLRKMLEKAPPHCCPSSEDEVRQFMWLGDETQNDIPSA

>Nymphalis_io_CRY-1-_XP_050359972.1 XP_050359972.1

MFFPGDLVLRTPAERIARHVILDSDVGDVVMSAAAETPPASSTRTPVAAQVSTPSGRQPVKNTVHWFRKGLRLHDNPALR

EGLTGAATFRCIFIIDPWFASSSNVGINKWRFLLQCLEDLDNNLKKLNSRLFVVRGQPADALPKLFREWETTALTFEEDP

EPYGRVRDHNITTKCREVGIKVTSRVSHTLYKLDNIIERNGGKAPLTYHQFQALIASMPPPPPAEPAITADMLNGAITPL

GADHEDRFGVPTLEELGFDTDGLKPPLWIGGENEALLRLERHLERKAWVASFGRPKMTPQSLLASQTGLSPYLRFGCLST

RLFYYQLTELYTRVKRVLPPLSLHGQILWREFFYCAATKNPNFDRMEGNPICVQIPWQKNQDALLKWANGKTGYPWIDAI

MIQLREEGWIHHLSRHAAACFLTRGDLWISWEEGMKVFDELLLDADWSVNAGMWMWLSCSSFFQQFFHCYCPVRFGRKID

PNGDFIRKYIPALRNLPTRFIHEPWMAPETVQQDANCIIGRDYPQPIVDHAKASQVNIERIKQVYAQLAKYKPPGSSIPQ

VLQRPNVTQSSPSPTSIIANINQSSYLCSQTSDSASQSSPATQIKDDGFLRPANIRSIATNKATPFKQVVIIQQERNKRE

RTVVEDMNGSNNGDTNKSIKMNDNTNTTQNLEKSGNYDIRNLLLNNCIRKFSSDQLAFLNQRTNKTENFTQDIKIDEYST

SKPKLFFTENGVMPCHDSKQKITSNNFSNNNHSKDSDTKVQSEDSSNEHNMNIKDRPTPSDSQDNISDNEKK

>Nymphalis_io_PL_X1_XP_050348778.1 XP_050348778.1

MISGLKRSVITVSNISSKGKSSKKNKTYKHENVDIDNIKTEIQKKREETAESILNFSFNKKRLRIVSQEQMVADDCEGIV

YWMSRDSRVQDNWAFLFAQKLALKNKVPLHVCFCLIAEYLEASVRQFDFLLKGLEQVAEECNKLNISFHLLDGSGGEVLP

QWVFDHKIGAVVCDFNPLRIPLGWLEDVKKKLKKDVPLIQVDAHNVVPCWVASDKREYSARTIRNKITSKLEEFLTEFPP

VIKHPYSSKFEPEPINWEEALKTRKADESVLPVNWAKPGYDYALQVLKSFIDERLKIYATKRNDPTQNALSNLSPWFHFG

QISVQRVALCVQEYKSKHTESVNAFLEEAIVRRELADNYCFYCDHYDSIEGASAWAQKTLNDHKKDTRPYVYNLEQLCQA

ITHDNLWNAAQIQMVDEGKMHGFLRMYWCKKILEWTESPEKALEYAIYLNDHYSIDGRDPNGYVGCMWSICGIHDQGWAE

RAVFGKIRYMNFEGCKRKFDVATFMQRYARKSNISIIHVNRNSLCMSHCWAKGLLSSF

>Ochrogaster_lun_PL__AFV08847.1 AFV08847.1

PNSIEPSTTVLSPYLSHGCLSSKLFYHKLKEIESCMPHTSPPVSLLGQLMWREFYYTAGAGTDNFDKMVGNPLCIQIPWG

KNDEHLKAWAEGRTGYPFVDAIMRQLKQEGWIHHLARHMVACFLTRGDLWISWEEGAKIFEDYLLDYDWSLNVGNWMWLS

ASAFFYKYFRVYSPIAFGKKTDKEGLYIRKYVPELGKYPTEYIYEPWKAPKSVQTSA

>Odontomachus_bru_CRY-1__XP_032680930.1 XP_032680930.1

MAVRRGSVRNGPLKKMTGSSNNKMGQGVTSVRGDGRKHMVHWFRKGLRLHDNPSLREGLAGASTFRCVFVLDPWFAGSTN

VGINKWRFLLQCLEDLDCSLRKLNSRLFVIRGQPADALPKLFKEWGTTDLTFEEDPEPFGRVRDHNISALCKELGISVVQ

RISHTLYKLDEIIEKNAAKSPLTYHQFQNVVASMDPPEMPVPTVTSACIGSAYTPLKDDHDDHYGVPTLEELGFDTEGLL

PPVWVGGESEALARLERHLERKAWVASFGRPKMTPQSLLPSQTGLSPYLRFGCLSTRLFYYQLTDLYKKIKKAVPPLSLH

GQLLWREFFYCAATKNPNFDRMQGNPICVQIPWDKNVEALAKWANGQTGFPWIDAIMTQLREEGWIHHLARHAVACFLTR

GDLWISWEEGMKVFDELLLDADWSVNAGMWMWLSCSSFFQQFFHCYCPVRFGRKADPNGDYIRRYLPVLKNFPTRYIHEP

WNAPLSIQHAAKCIIGKEYSLPMVNHSKSSRINIERMKQVYQQLNKYRGNGASFKGETVGLLNALLAPSTKDTNEEKTKQ

DSPSQENEQKMETISNPTSQQQQQQQQQQQQP

>Odontotermes_form__QLJ82960.1 QLJ82960.1

MNAGSSEKHTVHWFRKGLRLHDNPSLREGLKSSTTFRCVFILDPWFAGSSNVGINKWRFLLQCLEDLDQNLRKLNSRLFV

IRGQPADALPKLFKEWGTTNLTFEEDPEPFGWVRDQNITAMCKELGITVISRVSHTLYQLETIIKKNGGKAPLTYHQFQT

IVASMETPPPTEPTINQHFLNGTYTPIGEDHDEKYGVPTLEELGFDTDGLLPPVWKGGESEALARLERHLERKAWVASFG

RPKMTPQSLLASQTGLSPYLRFGCLSTRLFYYQLTDLYKKIKKAYPPLSLHGQLLWREFFYCAATKNPNFDKMSGNPICV

QIPWDRNAEALAKWANGQTGFPWIDAIMTQLRQEGWIHHIARHAVACFLTRGDLWISWEEGMKVFEELLLDADWSVNAGM

WMWLSCSSFFQQFFHCYCPVRFGRKADPNGDYIRKYLPVLKNFPTRYIHEPWNAPEAVQKAAKCIIGKEYSLPMVNHAVA

SRINIERMKQVYQQLSKYRGPGLLATVPTSQSRTSNTLNPTLGQKDFQKSSETKWEALPGVDINGYSLVICDEDFKTGIQ

SYCYKSQNKEEYE

>Onthophagus_tau_CRY-1-_XP_022918997.1 XP_022918997.1

MSGLRINSGQRSDGRGHGKGHGRDKHMVHWFRKGLRLHDNPSLREGLKGATTFRCVFILDPWFAGSSNVGINKWRFLLQC

LEDLDRSLRKLNSRLFVIRGQPADALPKLVKEWGINTLSFEEDPEPFGRVRDHNITALCKELGITVIQKVSHTLYHLQHI

IDRNAGRAPLTYHQFLAIIACMAPPPLAESPVTQQTLNGATTPISEDHDEKYGVPTLEELGFDTDGLLPPVWQGGESVAL

ARLERHLERKAWVASFGRPKMTPQSLLPSQTGLSPYLRFGCLSTRLFYYQLTDLYKKIKKTFPPLSLHGQLLWREFFYCA

ATKNPNFDKMIGNPICVQIPWDKNSEALAKWASGQTGFPWIDAIMTQLREEGWIHHLARHAVACFLTRGDLWLSWEEGMK

VFEELLLDADWSVNAGMWMWLSCSSFFQQFFHCYCPVKFGRKADPNGDYIRKYLPVLKNMPVQYIHEPWTAPENVQRATK

CIIGKDYPLPMVNHAIASKINIQRMKQVYQQLAKYRRLETDSCKMQGNFKDGYQAQVVTVGNPDNLNDE

>Ooceraea_bir_CRY-1_XP_011338806.1 XP_011338806.1

MTGSSNNEMSQEMTSGVGGDGKKHTVHWFRKGLRLHDNPSLKEGLAGASTFRCVFVLDPWFAGSTNVGINKWRFLLQCLE

DLDCSLRKLNSRLFVIRGQPADALPKLFKEWGTTNLTFEEDSEPFGRIRDHNISALCKELGISVDQKISHTLYKLDEIIE

RNGGKPPLTYHQFQNIVAGMDPPEAPVSTVTATCIGSAYTPLKDDHDDHYGVPTLEELGFDTEGLLPPLWIGGESEALAR

LERHLERKAWVASFGKPKMTPQSLLPSQTGLSPYLRFGCLSTRLFYYQLTDLYKKIKKAMPTLSLHGQLLWREFFYCAAT

KNLNFDRMQGNPICVQIPWDKNIEALAKWANGQTGFPWIDAIMTQLREEGWIHHLARHAVACFLTRGDLWISWEEGMKVF

DELLLDADWSVNAGMWMWLSCSSFFQQFFHCYCPVRFGRKADPNGDYIRRYLPVLKNFPTRYIHEPWNAPLSIQHAAKCI

IGKEYSLPMVDHSKSSRINIERMKQVYQQLNKYRGNATSFKRKKYRLAERVAGTTGKEFR

>Operophtera_bru_6-4_PL_KOB69753.1 KOB69753.1

MSQKPTVIHWFRLDLRIHDNLALRNAINEAENRKHLLRPVYFIEPDIKRKVGVNRLRFLIQSLHDLDSSLRQLNSRLYVV

KGKAVDNLPKLFDNWAVKYLTSQVDIDPEVVQQHEAIEKIANEKDIFINSRVQHTVYDPNIVLKKNNGVVPLTYQKFLSL

VNDIQVKENIQISKQISNECKPPDETSQDYDVPTLHDVGLDESSMGPSKFPGGETEGIKRLNLYMAKKQWVCKFEKPKSS

PNSIEPSTTVLSPYISHGCLSAKHFYHKLKEAEHGMQHSLPPVSLMGQLMWREFYYTAGTGTENFDKMVGNSVCTQIPWG

RNDEHLKAWAEGRTGYPFVDAIMRQLKQEGWIHHLARHMVACFLTRGDLWISWEEGAKVFEDLLLDYDWSLNAGNWMWLS

ASAFFYKYFRVYSPIAFGKKTDKNGLYIRKYVPELKKYPAEYIYEPWKAPKSVQSAAGCVIGEGYPKRIVDHDTIHKENM

KKMSEAYKKNKEKKSLKRPRS

>Operophtera_bru_CRY_2__KOB73084.1 KOB73084.1

KGLRLHDNPSLREGLDNAATFRCVFLIDPWFASFSNVGIHKWRFLLQCLQDLDASLRKLNSRLFVIRGQPADALPKLFKE

WGTTSLTFEEDPEPYGRVRDHNIMTKCREAGIKVISRVSHTLYKLDKIIERNGGKAPLTYHQFQALIASMPAPPPAEPAV

CAESLNGAATPLDLNHDERFGVPTLEELGFDTEHLQAPVWIGGESEALARLERHLERKAFGCLSTRLFYYQLTELYKRVK

RVRPPLSLHGQILWREFFYCAATRNPNFDRSNRFPMDRRHHDPAETGRLDPSPREARGGVLPHQRRPVFDELLLDADWSV

NAGMWMWLSCSSFFQQFFHCYCPVRFGRKTDPNGDFIRRYIPALVNMPTRYIHEPWVAPESVQESAKCIIGRDYPMPIVD

HAKASQVNIERIKQVYAQLANPTSIIASINQSSYLCSASPAPPPAPYNDADLFQRPMKLAPPHRGDRHPKCKEIVIVHKV

HKSSVALANPANPQKTFVANGKIDSSFKHRSLPHGRQENCQFKNLAINNFVNGYSSQNVYPGATPNYQPNPNSEYQQVMK

INNYNFEQTKFYVPVYPQNVAPRPAHTAVPFIPDPIDGKRERKKEGTKDKSNMNADRSYKI

>Operophtera_bru_CRY__KOB58217.1 KOB58217.1

MLGGSVLWFRHGLRLHDNPSLHAALEDRSVPFFPVFIFDGETAGTKVVGYNRMRYLLEALDDLDSQFKKFGGRLIMVKGK

PKEVFRRLWEEFGIRKLCFEQDCEPVWRARDDSVKTACRETGVTVREHVSHTLWEPDTVIRANGGIPPLTYQMFLHTVAT

IGDPPRPVIDIDWHGVKFGTLPDCFKEEFTVFDKNDDIRMIRWVGGETTALKQMQQRLAVEYETFCRADIRMIRWVGGET

TALKQMQQRLAVEFYWGLQDLFTQVYQDRLNSSHFITDSVLGSVKNYPTITVSGDINIDIQPDNFDRHSLSYLELLATHG

LLPGHTLITRGEIFYDHIILKSKNECSTVILQTYLTDHCPALLSFTKTGK

>Operophtera_bru_DNA_PL_KOB77153.1 KOB77153.1
[truncated: 157,552 more chars]
