# Supplementary figures and images for: Contribution of cryptochromes and photolyases for insect life under sunlight
Source: J Comp Physiol A Neuroethol Sens Neural Behav Physiol. 2023 Jan 6;209(3):373–89. doi: 10.1007/s00359-022-01607-5 (PMC10102093; doi:10.1007/s00359-022-01607-5)

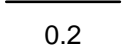

Supplement: Supplementary file 3 — Supplementary file3 (PDF 242 KB) [file 359_2022_1607_MOESM3_ESM.pdf]

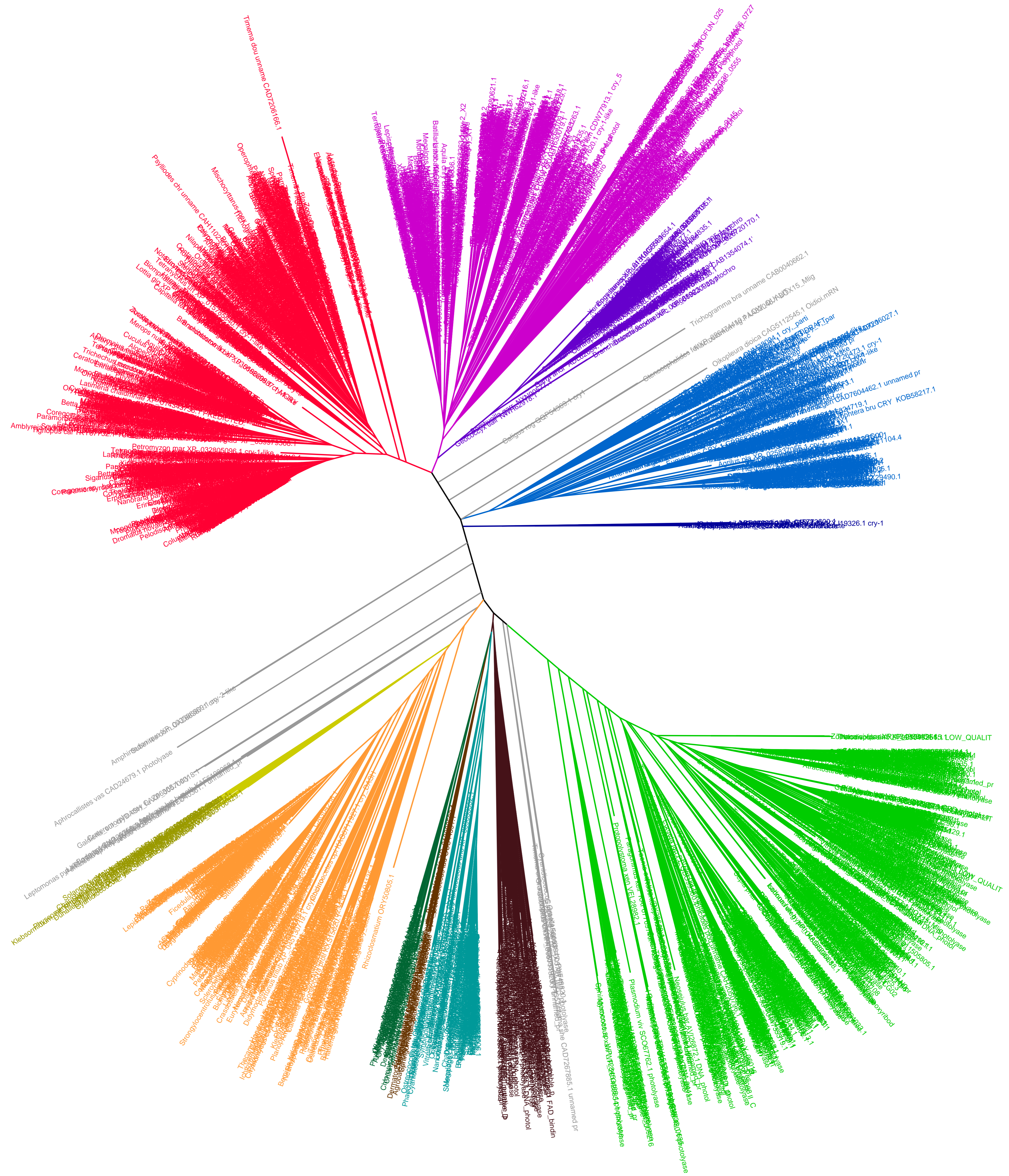

Supplement: Supplementary file 4 — Supplementary file4 (PDF 202 KB) [file 359_2022_1607_MOESM4_ESM.pdf]

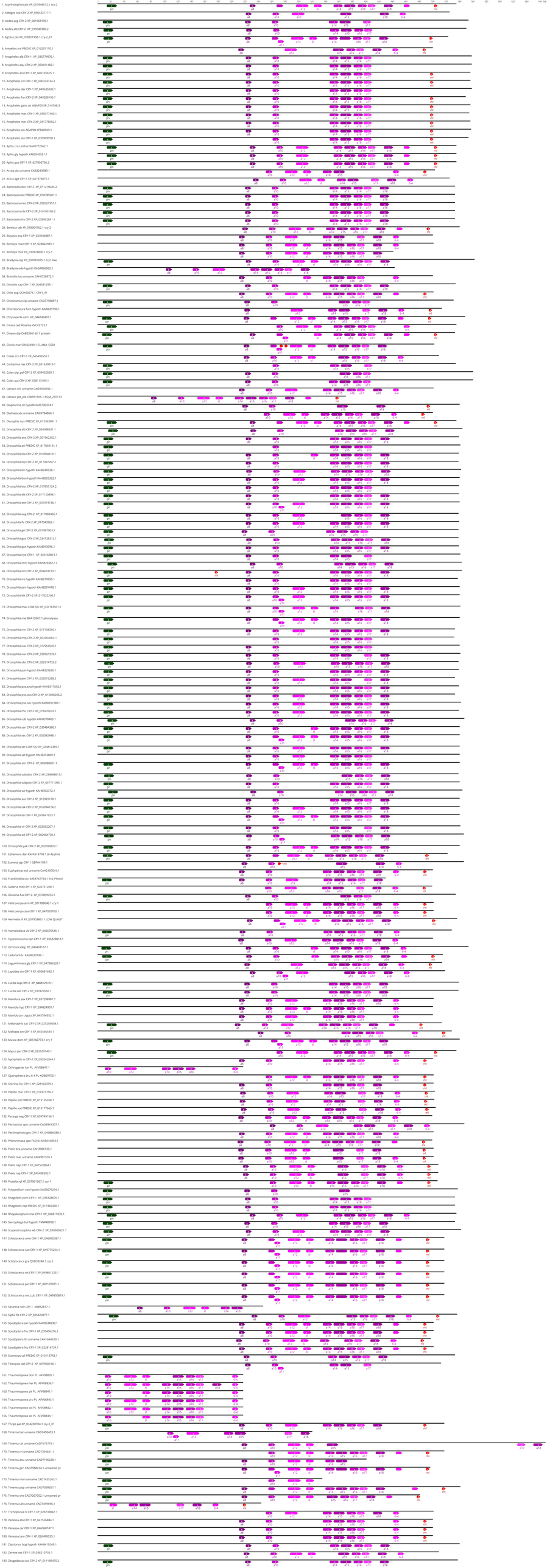

Supplement: Supplementary file 7 — Supplementary file7 (ZIP 16298 KB) [file 359_2022_1607_MOESM7_ESM.zip › 6-4 PLs.pdf]

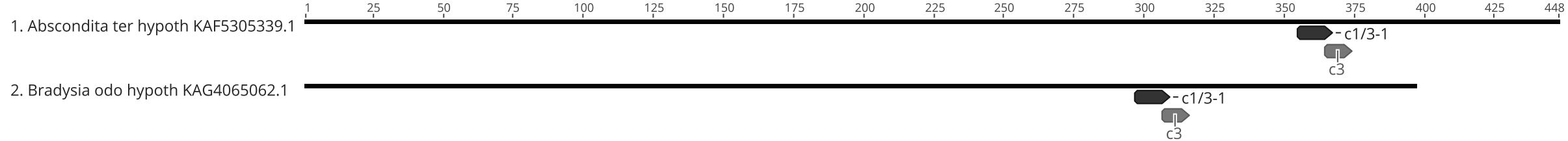

Supplement: Supplementary file 7 — Supplementary file7 (ZIP 16298 KB) [file 359_2022_1607_MOESM7_ESM.zip › CPDI PLs.pdf]

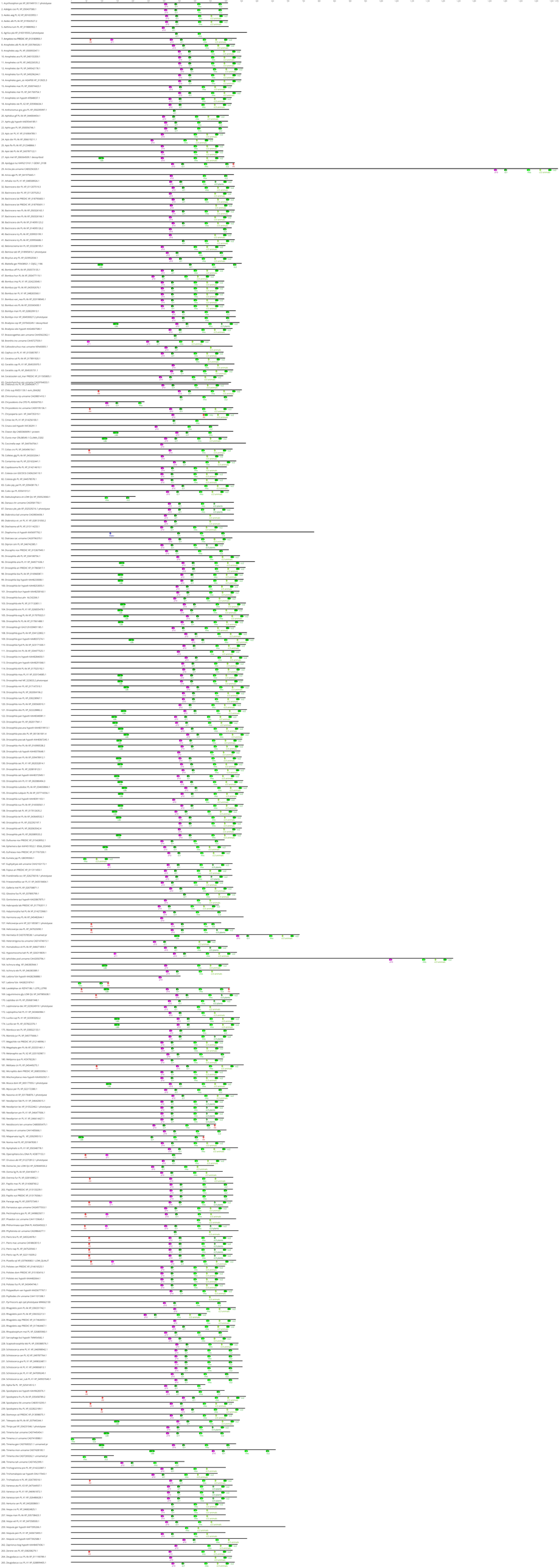

Supplement: Supplementary file 7 — Supplementary file7 (ZIP 16298 KB) [file 359_2022_1607_MOESM7_ESM.zip › CPDII PLs.pdf]

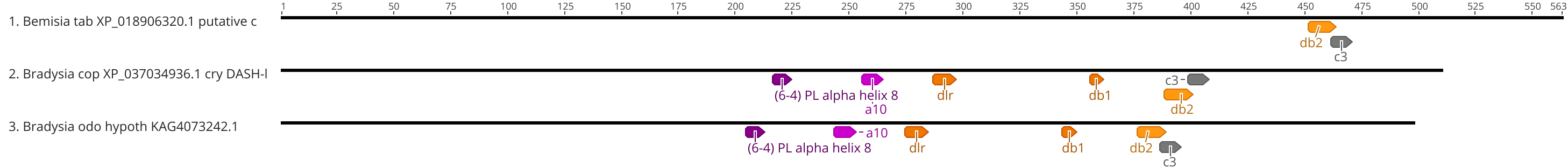

Supplement: Supplementary file 7 — Supplementary file7 (ZIP 16298 KB) [file 359_2022_1607_MOESM7_ESM.zip › DASH-CRYs.pdf]

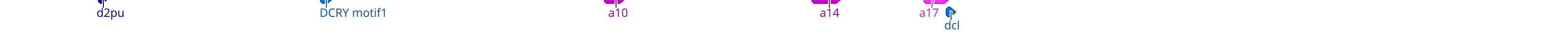

Supplement: Supplementary file 7 — Supplementary file7 (ZIP 16298 KB) [file 359_2022_1607_MOESM7_ESM.zip › DCRYs.pdf]

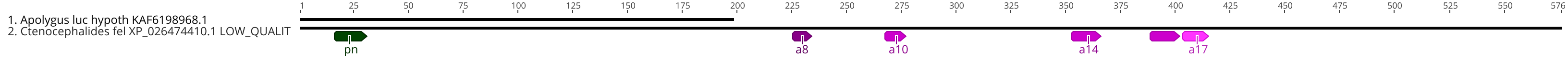

Supplement: Supplementary file 7 — Supplementary file7 (ZIP 16298 KB) [file 359_2022_1607_MOESM7_ESM.zip › other.pdf]
