# Supplementary material for: Contribution of cryptochromes and photolyases for insect life under sunlight
Source: J Comp Physiol A Neuroethol Sens Neural Behav Physiol. 2023 Jan 6;209(3):373–89. doi: 10.1007/s00359-022-01607-5 (PMC10102093; doi:10.1007/s00359-022-01607-5)

Supplementary file 8

1. Timema protein sequence alignment…………………………………………….1
2. Ladona & Ishnura protein sequence alignment…………………………………2
3. Tephritoidea protein sequence alignment………………………………………3
4. Bactrocera dor & Bactrocera ole detailed protein sequence alignment……...5
5. Bactrocera dor & Bactrocera ole nucleotide sequence alignment……………6

1. Timema protein sequence alignment

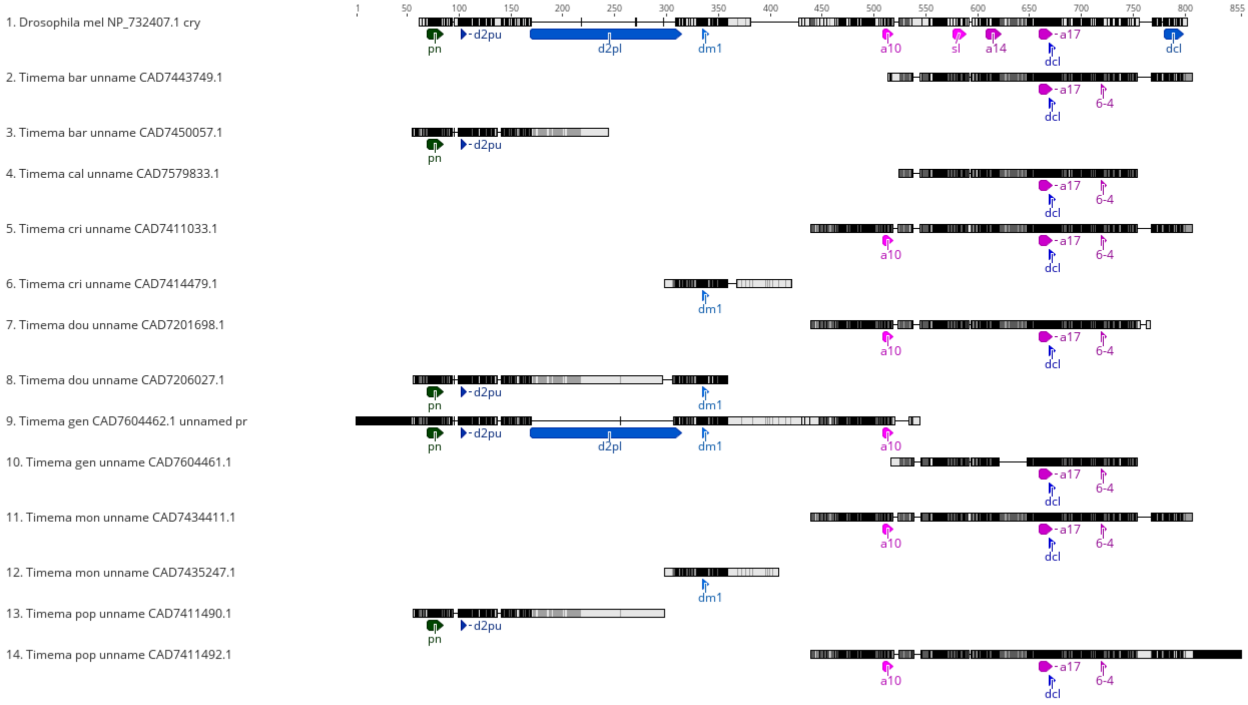

2. Ladona & Ishnura protein sequence alignment

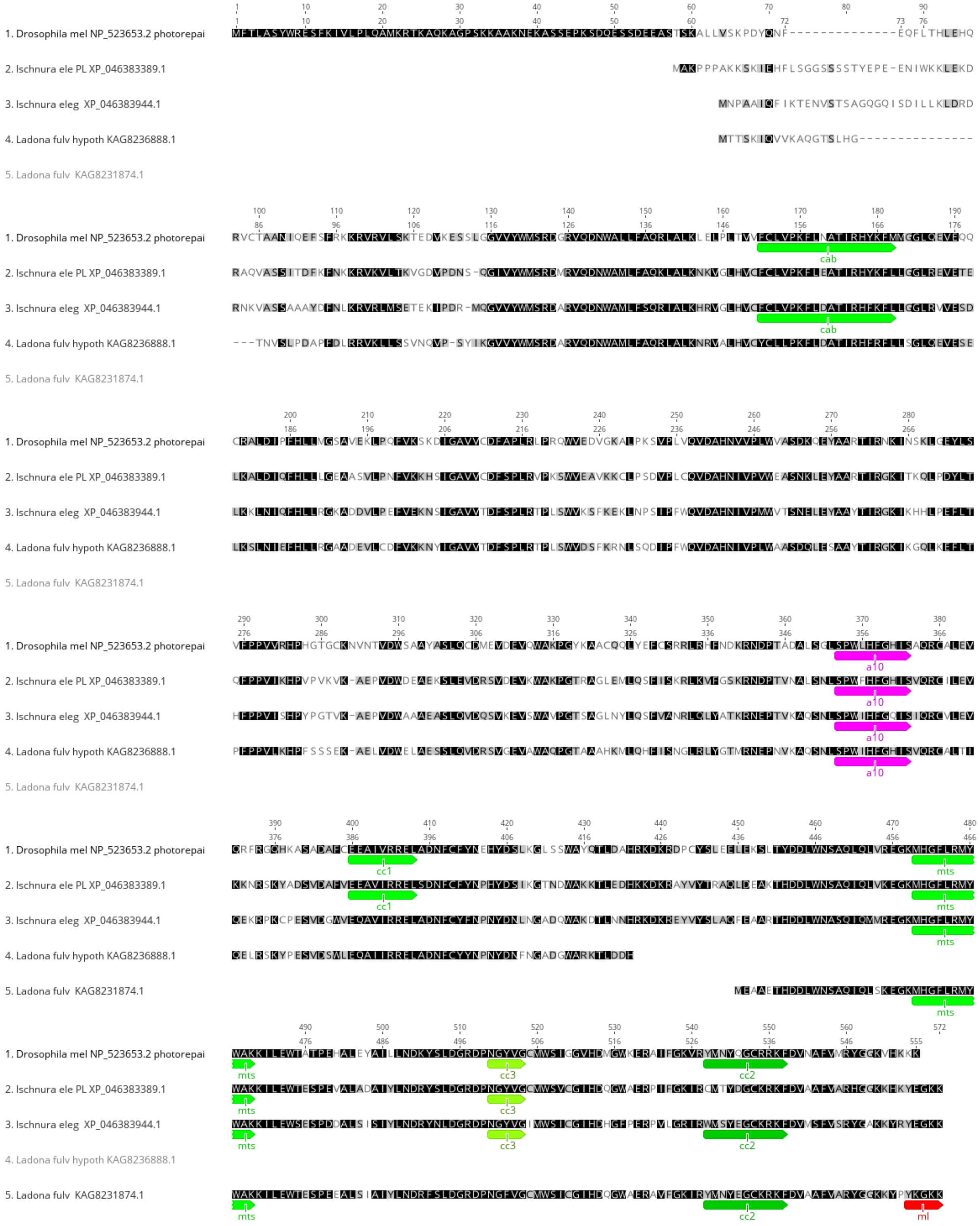

3. Tephritoidea protein sequence alignment

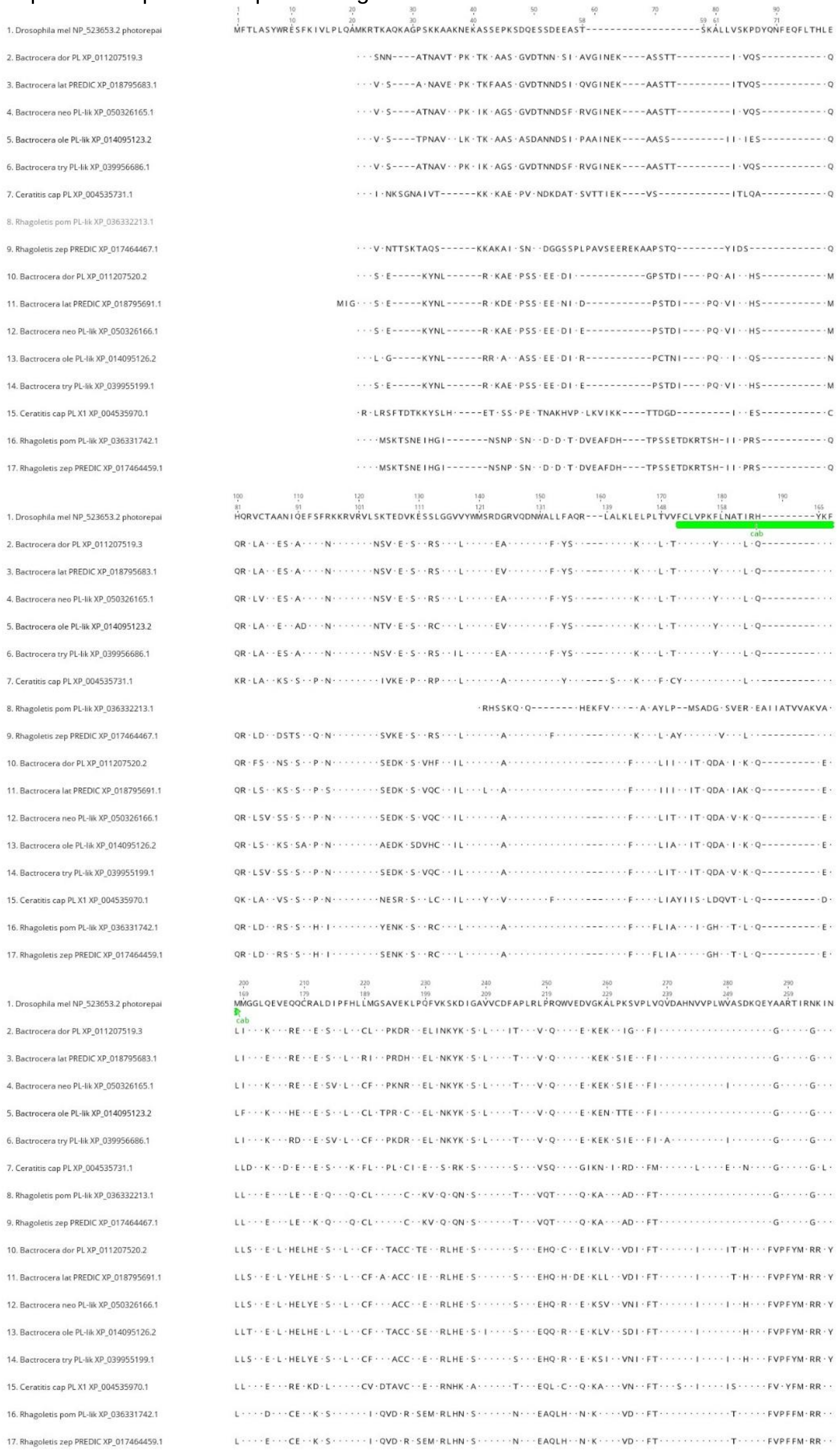

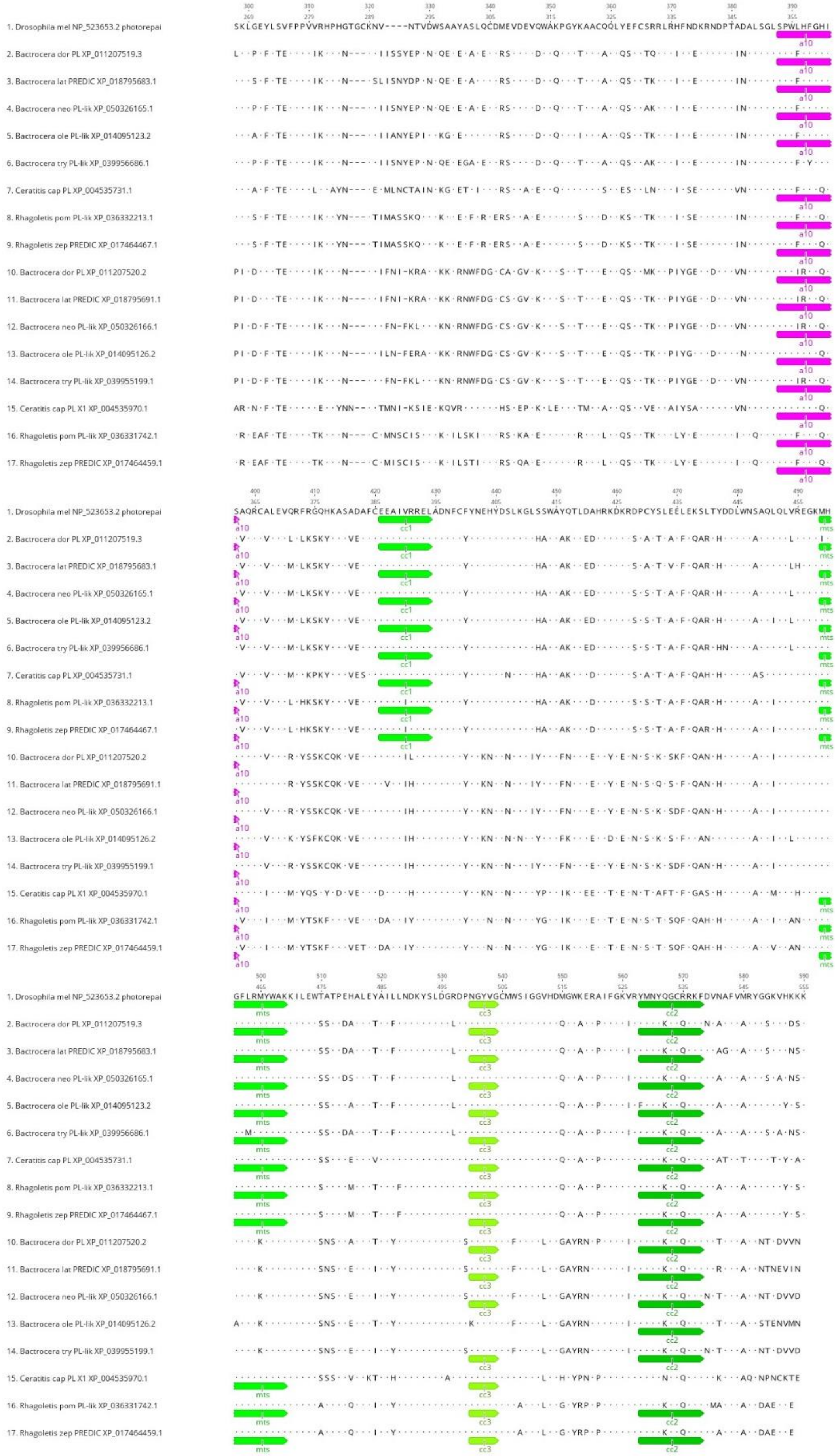
1. Drosophila mel NP_523653.2 photorepai
2. Bactrocera dor PL XP_011207519.3
3. Bactrocera lat PREDIC XP_018795683.1
4. Bactrocera neo PL-lik XP_050326165.1
5. Bactrocera ole PL-lik XP_014095123.2
6. Bactrocera try PL-lik XP_039956686.1
7. Ceratitis cap PL XP_004535731.1
8. Rhagoletis pom PL-lik XP_036332213.1
9. Rhagoletis zep PREDIC XP_017464467.1
10. Bactrocera dor PL XP_011207520.2
11. Bactrocera lat PREDIC XP_018795691.1
12. Bactrocera neo PL-lik XP_050326166.1
13. Bactrocera ole PL-lik XP_014095126.2
14. Bactrocera try PL-lik XP_039955199.1
15. Ceratitis cap PL X1 XP_004535970.1
16. Rhagoletis pom PL-lik XP_036331742.1
17. Rhagoletis zep PREDIC XP_017464459.1
a10
cc1
mts
cc3
cc2

4. Bactrocera dor & Bactrocera ole detailed protein sequence alignment

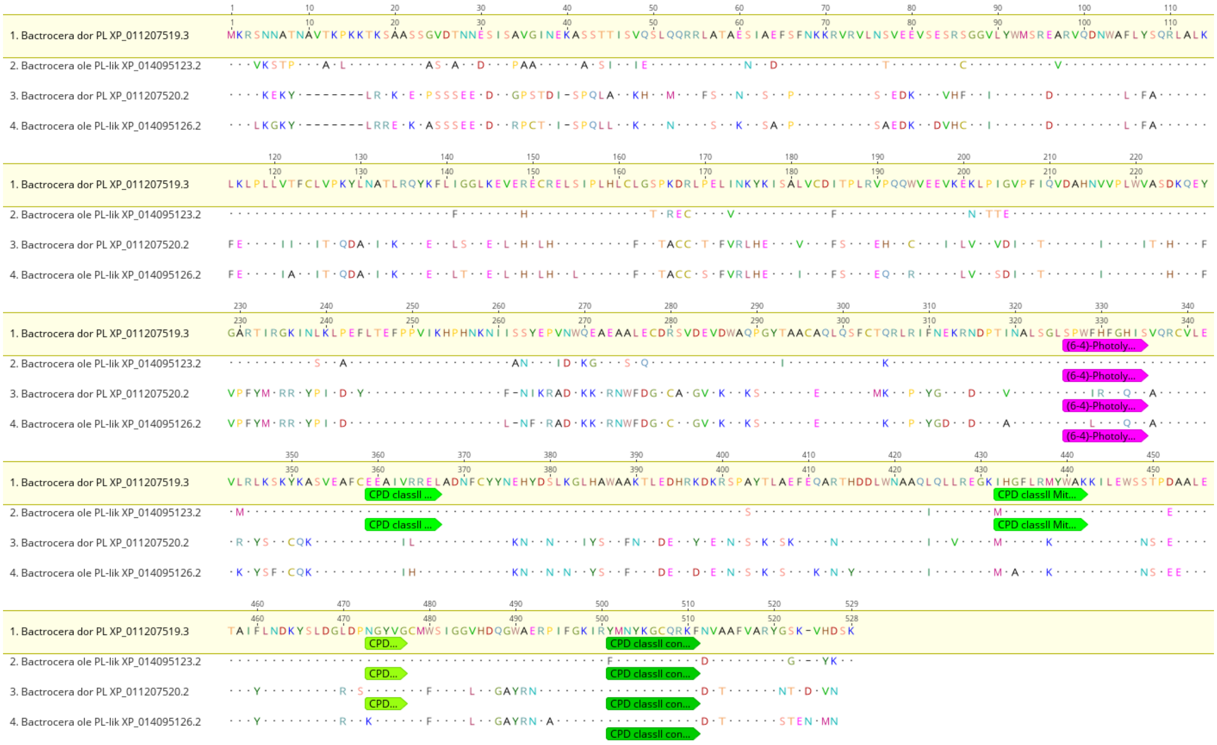

5. Bactrocera dor & Bactrocera ole nucleotide sequence alignment

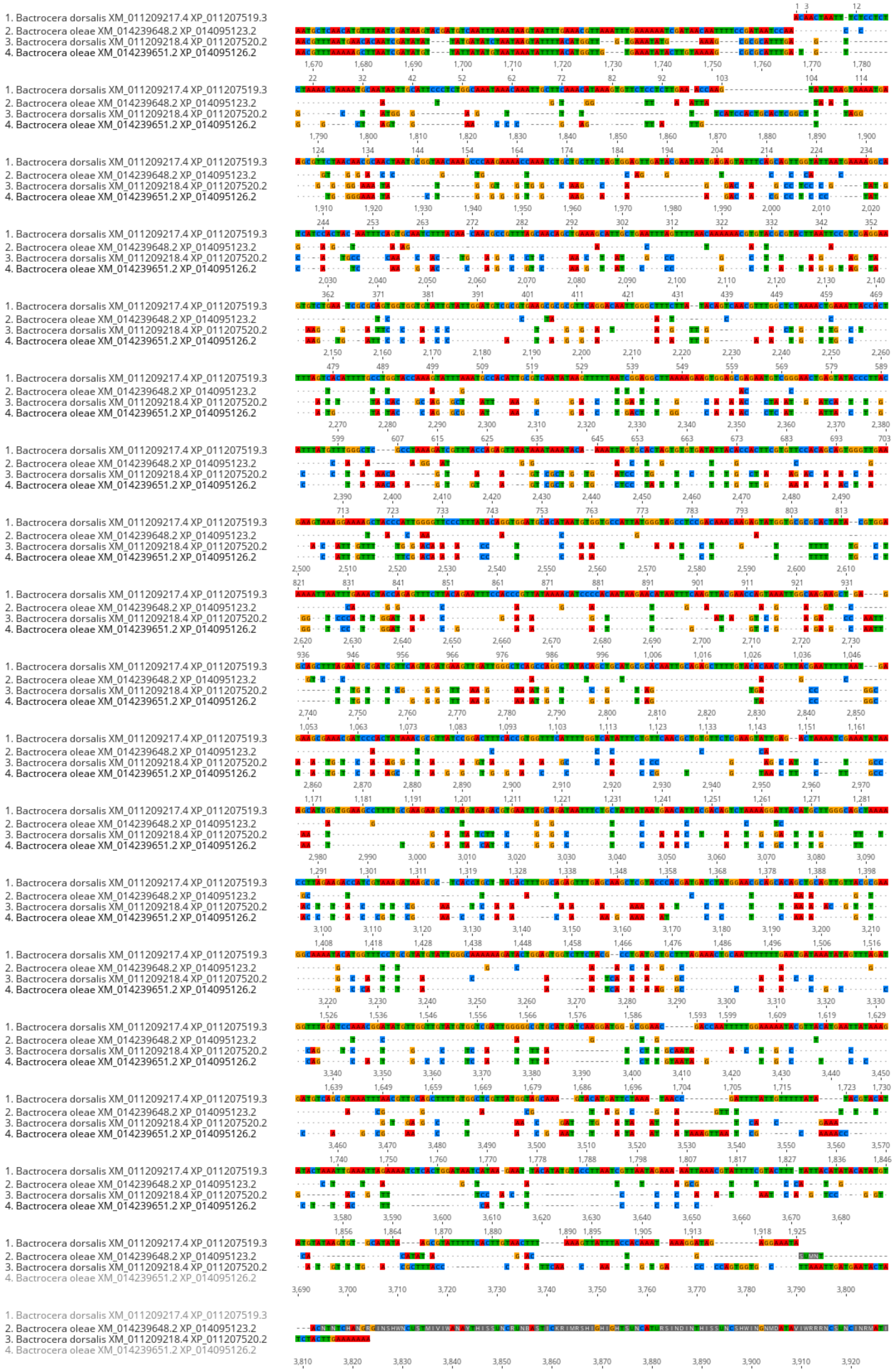

Supplement: Supplementary file 8 — Supplementary file8 (PDF 1261 KB) [file 359_2022_1607_MOESM8_ESM.pdf]
