# Supplementary material for: Contribution of cryptochromes and photolyases for insect life under sunlight
Source: J Comp Physiol A Neuroethol Sens Neural Behav Physiol. 2023 Jan 6;209(3):373–89. doi: 10.1007/s00359-022-01607-5 (PMC10102093; doi:10.1007/s00359-022-01607-5)
Supplement: Supplementary file 10 — Supplementary file10 (DOCX 762 KB) [file 359_2022_1607_MOESM10_ESM.docx]

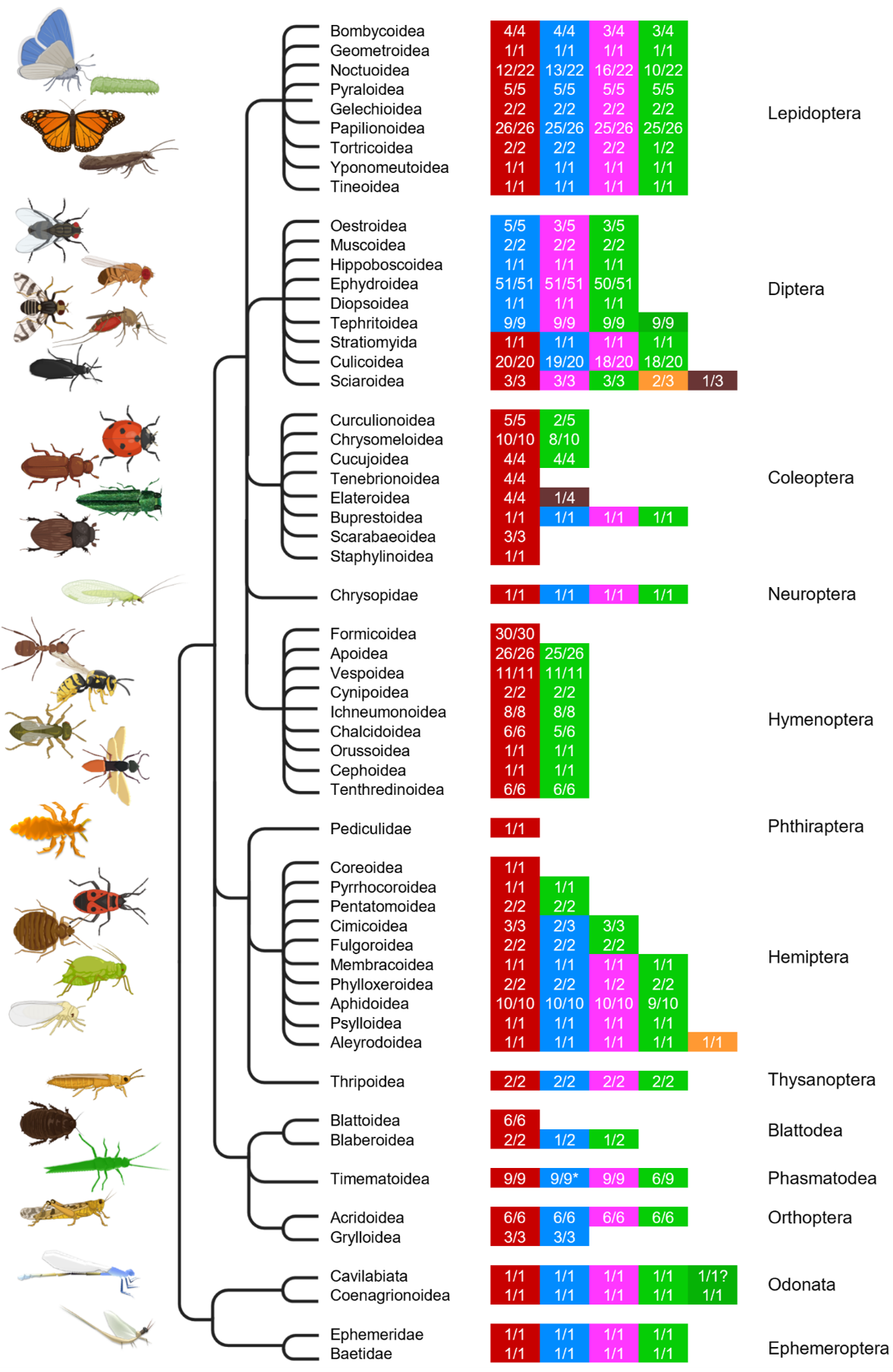


Distribution of CRY/PL subfamilies within insect orders and superfamilies without considering sequencing quality. MCRY is shown in dark red, DCRY in blue, 6-4 PL in light magenta, CPDII in light green, CPDII duplication in green, CPDI in brown, and DASH-CRY is shown in orange. The prevalence of a CRY/PL within an insect superfamily is shown as a fraction, with the denominator representing the total number of animals examined in a superfamily and the numerator representing the number of animals with the respective CRY/PL. MCRY, DCRY, 6-4 PL, and CPDII PL are the most common CRY/PLs in insects. All studied insects belonging to the orders Ephemeroptera, Odonata, Phasmatodea, Thysanoptera, and Neuroptera possess them. Most Lepidoptera and many insects belonging to Hemiptera (depending on the superfamily) also have all of them. In Hymenoptera, the CRY/PLs are greatly reduced, and some groups retain only MCRY. Such reductions are also observed in insects belonging to other orders. Many superfamilies of Diptera lack MCRY and possess only DCRY, 6-4 PL, and CPDII PL. Some individual insects also have DASH-CRY or CPDI photolyase and others possess a CPDII photolyase duplication. DCRY is only fragmentarily sequenced and annotated in most Timematoidea (*). But in all studied Timema species we could find DCRY fragments.
