# Supplementary material for: Contribution of cryptochromes and photolyases for insect life under sunlight
Source: J Comp Physiol A Neuroethol Sens Neural Behav Physiol. 2023 Jan 6;209(3):373–89. doi: 10.1007/s00359-022-01607-5 (PMC10102093; doi:10.1007/s00359-022-01607-5)

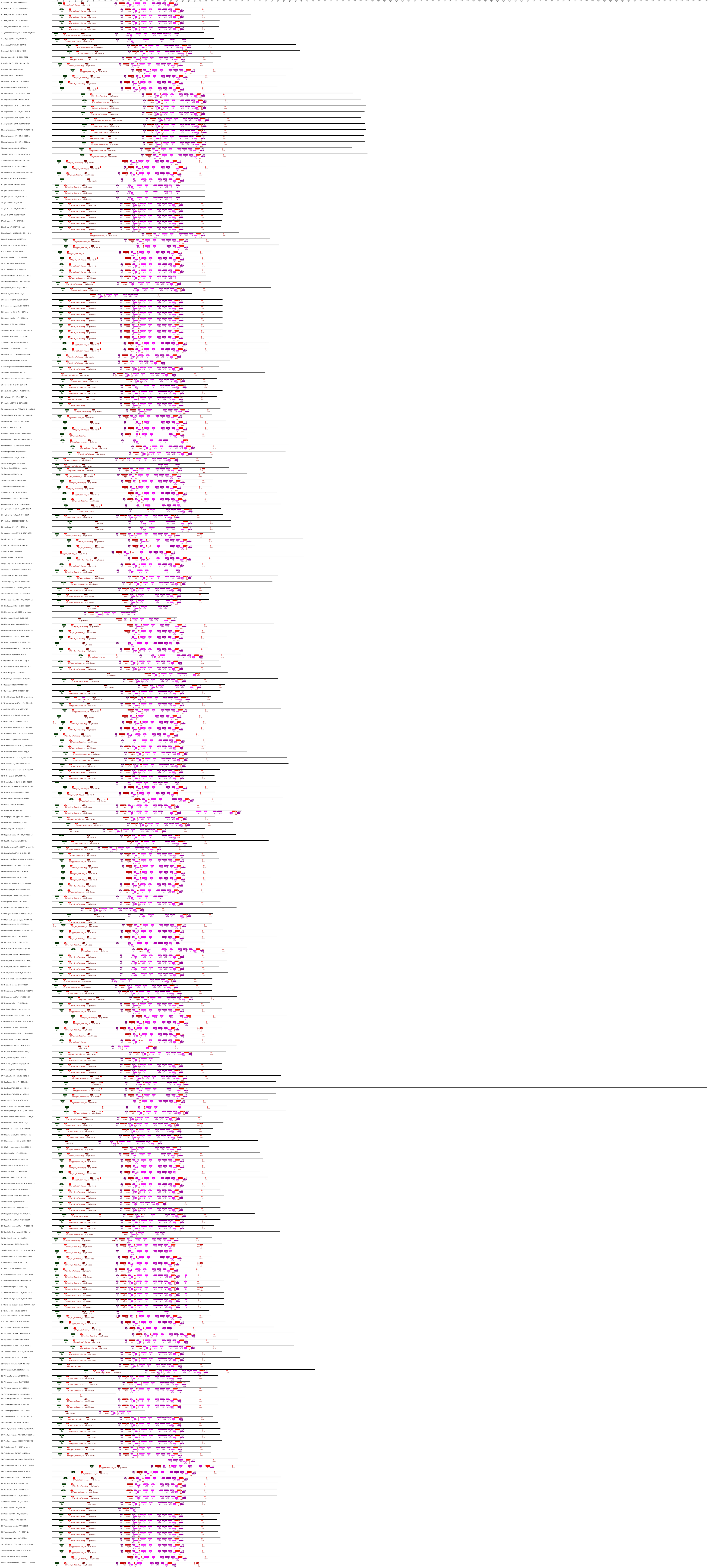

Supplement: Supplementary file 7 — Supplementary file7 (ZIP 16298 KB) [file MCRYs.pdf › MCRYs.pdf]
